# Global, regional, and national trends in haemoglobin concentration and prevalence of total and severe anaemia in children and pregnant and non-pregnant women for 1995–2011: a systematic analysis of population-representative data

**DOI:** 10.1016/S2214-109X(13)70001-9

**Published:** 2013-07

**Authors:** Gretchen A Stevens, Mariel M Finucane, Luz Maria De-Regil, Christopher J Paciorek, Seth R Flaxman, Francesco Branca, Juan Pablo Peña-Rosas, Zulfiqar A Bhutta, Majid Ezzati, on behalf of Nutrition Impact Model Study Group (Anaemia)

**Affiliations:** a Department of Health Statistics and Information Systems, WHO, Geneva, Switzerland; b Department of Nutrition for Health and Development, WHO, Geneva, Switzerland; c Gladstone Institutes, University of California, San Francisco, CA, USA; d Department of Statistics, University of California, Berkeley, CA, USA; e School of Computer Science and Heinz College, Carnegie Mellon University, Pittsburgh, PA, USA; f Division of Women and Child Health, Aga Khan University, Karachi, Pakistan; g Department of Epidemiology and Biostatistics, MRC-HPA Centre for Environment and Health, Imperial College London, London, UK

## Abstract

**Background:**

Low haemoglobin concentrations and anaemia are important risk factors for the health and development of women and children. We estimated trends in the distributions of haemoglobin concentration and in the prevalence of anaemia and severe anaemia in young children and pregnant and non-pregnant women between 1995 and 2011.

**Methods:**

We obtained data about haemoglobin and anaemia for children aged 6–59 months and women of childbearing age (15–49 years) from 257 population-representative data sources from 107 countries worldwide. We used health, nutrition, and household surveys; summary statistics from WHO's Vitamin and Mineral Nutrition Information System; and summary statistics reported by other national and international agencies. We used a Bayesian hierarchical mixture model to estimate haemoglobin distributions and systematically addressed missing data, non-linear time trends, and representativeness of data sources. We quantified the uncertainty of our estimates.

**Findings:**

Global mean haemoglobin improved slightly between 1995 and 2011, from 125 g/L (95% credibility interval 123–126) to 126 g/L (124–128) in non-pregnant women, from 112 g/L (111–113) to 114 g/L (112–116) in pregnant women, and from 109 g/L (107–111) to 111 g/L (110–113) in children. Anaemia prevalence decreased from 33% (29–37) to 29% (24–35) in non-pregnant women, from 43% (39–47) to 38% (34–43) in pregnant women, and from 47% (43–51) to 43% (38–47) in children. These prevalences translated to 496 million (409–595 million) non-pregnant women, 32 million (28–36 million) pregnant women, and 273 million (242–304 million) children with anaemia in 2011. In 2011, concentrations of mean haemoglobin were lowest and anaemia prevalence was highest in south Asia and central and west Africa.

**Interpretation:**

Children's and women's haemoglobin statuses improved in some regions where concentrations had been low in the 1990s, leading to a modest global increase in mean haemoglobin and a reduction in anaemia prevalence. Further improvements are needed in some regions, particularly south Asia and central and west Africa, to improve the health of women and children and achieve global targets for reducing anaemia.

**Funding:**

Bill & Melinda Gates Foundation, Grand Challenges Canada, and the UK Medical Research Council.

## Introduction

Anaemia, or low concentrations of haemoglobin, adversely affect cognitive and motor development and cause fatigue and low productivity.^1^, ^2^, ^3^ Low haemoglobin concentrations during pregnancy can be associated with an increased risk of maternal and perinatal mortality and low size or weight at birth.^2^, ^4^, ^5^, ^6^ Maternal and neonatal deaths are a major cause of mortality in developing countries, and together cause between 2·5 million and 3·4 million deaths worldwide.^7^, ^8^, ^9^ Although some adverse effects are associated with high haemoglobin concentrations,^10^ most take place along a continuum of low concentrations, with each decrement associated with worse outcomes. Other effects might be restricted to concentrations that correspond to moderate-to-severe anaemia.^2^, ^4^

Awareness about anaemia and its consequences for the health and development of women and children has increased in the past few decades. In 2012, the 65th World Health Assembly approved an action plan and global targets for maternal, infant, and child nutrition, with a commitment to halve anaemia prevalence in women of reproductive age by 2025, from 2011 levels. As such, attention to nutritional interventions, such as the Scaling Up Nutrition initiative, has increased. Furthermore, emphasis has been placed on the reduction of risk factors that adversely affect women and children, for example in the UN Secretary-General's Every Woman Every Child initiative and the accompanying Global Strategy for Women's and Children's Health. To plan for these programmes and prioritise interventions, information is needed about haemoglobin and anaemia in women and children, and how they have changed over time. We aimed to estimate trends in the complete distributions of haemoglobin concentration and anaemia prevalence by severity for young children and pregnant and non-pregnant women by country and region.

## Methods

### Study design

We estimated 1995–2011 trends in distributions of haemoglobin concentration for children aged 6–59 months and for women of reproductive age (15–49 years), by pregnancy status, in 190 countries and territories organised into 11 regions (appendix p 10). Our analysis comprised three steps: (1) identifying data sources, accessing and extracting data, and systematically assessing population representativeness of data; (2) adjusting haemoglobin for altitude; and (3) applying a statistical model to estimate trends in haemoglobin distributions and their uncertainties. The distributions estimated in the third step provide coherent and consistent estimates of mean haemoglobin and of the prevalences of total and severe anaemia. We defined total anaemia on the basis of WHO cutoff points of haemoglobin less than 110 g/L for children younger than 5 years and pregnant women, and less than 120 g/L for non-pregnant women.^11^ We defined severe anaemia as haemoglobin less than 70 g/L for children younger than 5 years and pregnant women, and less than 80 g/L for non-pregnant women.^11^ Because we estimated full distributions, prevalences based on other cutoffs (eg, <80 g/L, which is used as an indicator of malaria burden in children^12^) can be calculated and are available from the investigators by request.

### Data sources

We designed our data search and access strategy to obtain as many sources as possible while ensuring that the sources were representative of the population at the national level, or at least covered three regions within the country. To estimate complete distributions without overly restrictive assumptions, we accessed as many sources as possible with data available for individual participants. The appendix (pp 1–3) provides details of our data search and access and our inclusion criteria. In brief, our data sources were health examination, nutrition, and household surveys with anonymised individual records available through national and international agencies and survey databases; summary statistics, including mean haemoglobin concentration and anaemia prevalence, from WHO's Vitamin and Mineral Nutrition Information System; and summary statistics not in the WHO database, which were provided or reported by other national and international agencies.

Our statistical model used stratified data for pregnant and non-pregnant women when available, and accounted for the proportion of women who were pregnant from sources that reported combined summaries for both groups of women. Reporting of pregnancy status in household surveys tends to become consistent only after about week 6–10 of pregnancy. This period is also about the same as that during which the decline in haemoglobin concentrations of pregnant women was steepest (appendix p 21). To be consistent with reporting behaviours and to restrict pregnancy to periods when haemoglobin concentrations are consistently low, we limited our operational definition of pregnancy to after 8 weeks of gestation (appendix p 4).

### Adjustment of haemoglobin for altitude and smoking

Physiological haemoglobin needs are greater at high altitude because of the lower concentration of oxygen in the atmosphere, and smoking increases haemoglobin concentrations.^11^, ^13^ To depict the health and functional consequences of low haemoglobin in a comparable way, haemoglobin concentrations of people living more than 1000 m above sea level should be adjusted downwards to avoid underestimation of anaemia prevalence. Some sources provided data that had been adjusted for altitude with a formula from the US Centers for Disease Control and Prevention (CDC; appendix p 5).^13^ For data sources that had not been adjusted at the time of access, when individual-level data included information about the altitude of residence, we adjusted for altitude with use of the CDC formula. When individual-level data did not contain information about the altitude of residence or when data were available as summary statistics only, we used the data without adjustment in 144 countries where 5% or fewer of the population lived at elevations of 1500 m or higher (altitudes that correspond to at least a 3 g/L effect on mean haemoglobin). For 11 data sources from countries where more than 5% of people lived at high altitudes, we adjusted summarised data sources for altitude (appendix p 5). We used data adjusted for smoking status if available (eg, for all Demographic and Health Surveys [DHS]), but made no post-adjustment to data that were not adjusted for smoking status because the effects of smoking (a roughly 0·3 g/L effect) are substantially smaller than are those of altitude (roughly 8 g/L for those living above 2000 m).

### Modelling of trends in haemoglobin distributions

We aimed to estimate the complete distributions of haemoglobin for every country and year, which would then allow us to calculate any relevant summary statistic. We did all analyses separately for children and women of reproductive age. We pooled data from boys and girls because their mean haemoglobin concentrations tracked closely (appendix p 22) and because some sources did not separate data by sex.

As described in detail elsewhere,^14^ we used a Bayesian hierarchical mixture model, in which estimates made for each country-year were informed by data from that country-year itself, if available, and by data from other years in the same country and in other countries, especially those in the same region with data for similar time periods (appendix pp 7–8). The hierarchical model borrows information to a greater extent when data are non-existent or weakly informative (ie, have large uncertainty), and to a lesser degree in data-rich countries and regions. We modelled trends over time as a linear trend plus a smooth non-linear trend, at national, regional, and global levels. The estimates are also informed by covariates that help predict haemoglobin concentrations, including maternal education,^15^ proportion of population in urban areas, mean latitude, prevalence of sickle-cell disorders and thalassaemias,^16^ mean body-mass index (BMI)^17^ for women, and mean weight-for-age *Z* score^14^ for children. All covariates were available for every country and year, except the prevalences of sickle-cell disorders and thalassaemias, which we treated as constant over time during the analysis period for each country. The model included a variance term that accounted for unobserved design factors (eg, sample design, season, method of haemoglobin measurement), which led to additional variability in the data beyond that expected because of sample size. Finally, the model accounted for the fact that subnational data and data that did not precisely cover the age ranges of interest might have had larger variations than did national data and data that did cover those ranges. We fitted the model to data from 1990 to 2012 to restrict boundary effects, but results are reported for 1995–2011, because very few sources were available between 1990 and 1995, and in 2012.

The model uses a mixture (ie, a weighted-average) of various normal (bell-shaped) densities to estimate the full haemoglobin distributions, which might themselves be skewed. We used a mixture of five normal distributions for children. For adult women, we used two five-component mixtures, one for pregnant women and one for non-pregnant women. This approach used all data sources—ie, those that separate pregnant and non-pregnant women, those in which pregnant and non-pregnant women are reported together, and those in which only one group was measured—to make separate estimates by pregnancy status. Differences in haemoglobin distributions between pregnant and non-pregnant women could vary by country and year. In years and countries for which separate data by pregnancy status were scarce, the difference was informed on the basis of other sources, especially those in the same region with data for similar time periods.

The uncertainties of our estimates incorporated sampling error in each data source; non-sampling error (eg, because of issues in sample design and measurement); additional error associated with subnational data; uncertainty due to altitude adjustments; and uncertainty due to estimates being made by country and year when data were missing, when only summary statistics (*vs* individual-level data) were available, or when data were not available separately by pregnancy status.

We fitted the Bayesian model with the Markov chain Monte Carlo (MCMC) algorithm and obtained 2500 samples from the parameters' posterior, which we used to obtain 2500 posterior samples of the population haemoglobin distributions for each country-year. We used the distributions to calculate the population haemoglobin mean and total and severe anaemia prevalence for each country-year. The credibility intervals represent the 2·5th–97·5th centiles of these sampled distributions. We calculated distributions for regions and the world as population-weighted averages of the constituent countries. We calculated average changes in mean haemoglobin or anaemia prevalence over the 16 years of reporting (absolute for mean and proportional for prevalence), and report findings as change per decade. We report the posterior probability that an estimated increase or decrease represents a truly increasing or decreasing trend. The posterior probability would be 0·50 when an increase is statistically indistinguishable from a decrease, and a larger probability shows more certainty.

### Role of the funding source

The sponsors of the study had no role in study design, data collection, data analysis, data interpretation, or writing of the report. The Writing and Global Analysis Group had access to all data sources. The corresponding author is responsible for the content of the report and had final responsibility for the decision to submit for publication.

## Results

**Figure 1:**
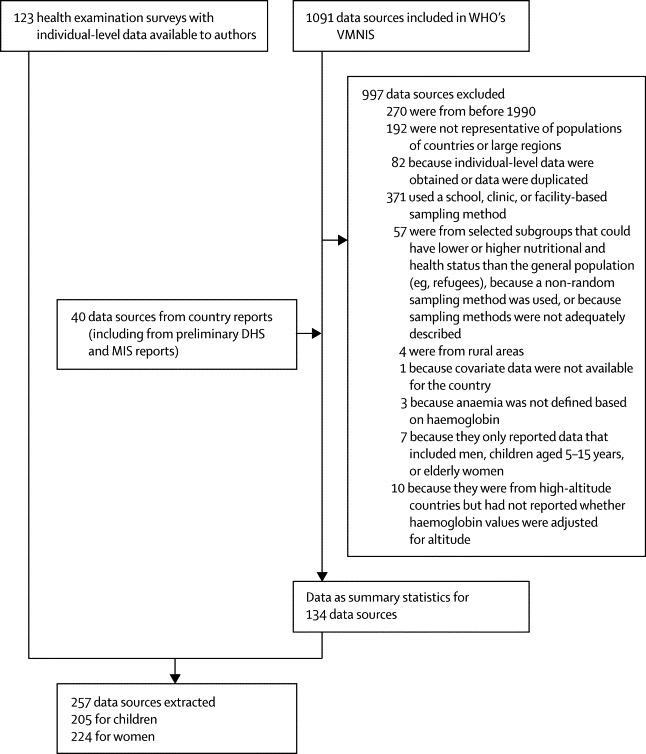
Flowchart of data identification, access, and extraction VMNIS=Vitamin and Mineral Nutrition Information System. DHS=Demographic and Health Surveys. MIS=Malaria Indicator Surveys.

Our final dataset included 257 surveys, comprising 232 (90%) nationally representative sources, with 1·9 million haemoglobin measurements (figure 1, appendix pp 11–20 and 23, 24). 205 (80%) sources had data for women (providing an average of 1·1 years of data per country or territory) and 224 (87%) had data for children (1·2 years of data per country or territory). Of 190 countries, 107 (56%), covering 85% of the global population of women and children, had at least one data source; 71 (37%) had at least two sources. Data were most sparse in high-income regions and in central and eastern Europe, with 43 sources in ten (20%) of 50 countries. By contrast, all countries in south Asia had at least one data source, with an average of 1·8 sources per country. At least one data source was available for 38 (79%) of 48 countries in sub-Saharan Africa.

**Figure 2:**
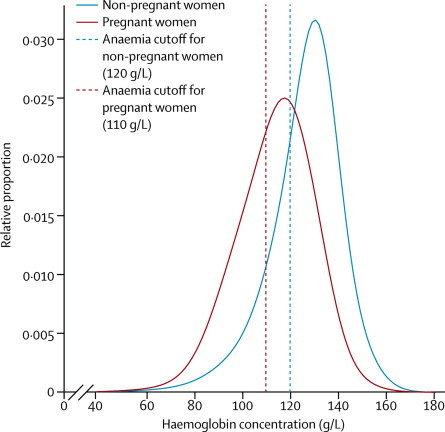
Global distributions of haemoglobin concentration for pregnant and non-pregnant women in 2011

In 1995, global mean haemoglobin concentrations were on average just below the thresholds for mild anaemia in children, and just above this threshold in non-pregnant and pregnant women (table). Corresponding values in 2011 were slightly improved (table), with posterior probabilities that the recorded increases were true increases of 0·95 for children, 0·87 for non-pregnant women, and 0·95 for pregnant women. Over this period, anaemia prevalence decreased by 4–5 percentage points in the three groups (table). Prevalence of severe anaemia, which is associated with substantially worse mortality, cognitive, and functional outcomes than is anaemia, also decreased (table). Anaemia prevalences of pregnant and non-pregnant women were only 9–10 percentage points apart in both 1995 and 2011 (table), even though the haemoglobin distribution in pregnant women was noticeably lower than that in non-pregnant women (figure 2). Relatively modest differences in prevalence despite noticeably shifted distributions happen because anaemia is defined on the basis of different cutoff values for these women (figure 2). The estimated anaemia prevalences translate to 273 million (95% credibility interval 242–304 million) children, 496 million (409–595 million) non-pregnant women, and 32 million (28–36 million) pregnant women with anaemia in 2011. Of these individuals, 10 million (7–14 million), 19 million (13–29 million), and 750 000 (520 000–1 100 000), respectively, had severe anaemia.

**Table:** Mean haemoglobin concentration and anaemia prevalence by region in 1995 and 2011

	**1995**			**2011**		
	Mean haemoglobin (g/L)	Anaemia (%)	Severe anaemia (%)	Mean haemoglobin (g/L)	Anaemia (%)	Severe anaemia (%)
**Children aged <5 years**						
High-income regions	123 (120–124)	11% (7–17)	0·3% (0·0–1·1)	123 (119–125)	11% (6–20)	0·1% (0·0–0·5)
Central and eastern Europe	116 (109–122)	29% (15–47)	1·4% (0·2–5·1)	117 (111–123)	26% (13–45)	0·2% (0·0–1·1)
East and southeast Asia	118 (115–120)	29% (22–37)	0·9% (0·5–1·5)	118 (113–123)	25% (16–38)	0·2% (0·1–0·6)
Oceania	111 (102–118)	42% (23–64)	2·0% (0·2–7·7)	112 (105–120)	43% (21–65)	0·5% (0·0–2·6)
South Asia	100 (96–105)	70% (59–78)	5·9% (3·0–9·1)	106 (102–111)	58% (44–69)	2·1% (0·8–4·4)
Central Asia, Middle East, and north Africa	111 (108–114)	43% (35–53)	1·5% (0·6–3·0)	114 (110–118)	38% (25–51)	0·4% (0·1–1·2)
Central and west Africa	95 (92–98)	80% (74–84)	9·7% (7·4–12·1)	100 (99–102)	71% (67–74)	4·9% (3·8–6·2)
East Africa	96 (93–100)	74% (65–81)	10·2% (7·7–12·6)	107 (105–108)	55% (50–59)	2·5% (1·8–3·6)
Southern Africa	116 (111–119)	30% (21–42)	1·1% (0·3–2·3)	110 (105–116)	46% (31–62)	0·9% (0·3–2·4)
Andean and central Latin America and Caribbean	113 (110–116)	38% (30–46)	1·4% (0·8–2·4)	116 (113–118)	33% (28–40)	0·4% (0·2–0·7)
Southern and tropical Latin America	117 (106–124)	28% (11–55)	1·3% (0·0–6·2)	119 (112–124)	23% (10–41)	0·2% (0·0–1·1)
Globe	109 (107–111)	47% (43–51)	3·7% (2·8–4·7)	111 (110–113)	43% (38–47)	1·5% (1·1–2·2)
**Non-pregnant women aged 15–49 years**						
High-income regions	131 (129–132)	14% (12–18)	0·6% (0·3–1·1)	130 (128–132)	16% (12–22)	0·5% (0·2–1·0)
Central and eastern Europe	129 (124–134)	23% (13–37)	0·9% (0·3–2·2)	128 (123–132)	22% (13–37)	0·5% (0·2–1·4)
East and southeast Asia	126 (123–128)	29% (22–39)	1·0% (0·6–1·7)	129 (124–133)	21% (12–36)	0·5% (0·2–1·2)
Oceania	123 (115–130)	37% (21–56)	2·8% (0·8–6·9)	126 (119–132)	28% (14–47)	1·8% (0·4–5·2)
South Asia	117 (113–121)	53% (42–64)	3·8% (2·3–5·8)	119 (115–124)	47% (33–59)	2·4% (1·0–4·6)
Central Asia, Middle East, and north Africa	123 (120–126)	38% (31–45)	2·0% (1·2–3·2)	125 (121–129)	33% (23–43)	1·0% (0·5–2·1)
Central and west Africa	118 (114–123)	52% (39–61)	2·8% (1·8–4·1)	119 (115–123)	48% (37–58)	2·2% (1·4–4·0)
East Africa	123 (120–127)	40% (33–47)	2·7% (1·8–3·8)	128 (126–131)	28% (23–34)	1·4% (1·0–1·9)
Southern Africa	124 (119–130)	33% (21–47)	2·0% (0·7–4·5)	128 (122–134)	28% (16–44)	1·2% (0·5–2·9)
Andean and central Latin America and Caribbean	126 (123–129)	30% (24–37)	1·7% (1·1–2·7)	131 (128–134)	19% (14–26)	0·7% (0·4–1·2)
Southern and tropical Latin America	129 (120–137)	22% (9–46)	1·2% (0·2–3·7)	130 (122–138)	18% (7–41)	0·7% (0·1–2·5)
Globe	125 (123–126)	33% (29–37)	1·8% (1·3–2·3)	126 (124–128)	29% (24–35)	1·1% (0·7–1·7)
**Pregnant women aged 15–49 years**						
High-income regions	119 (116–121)	23% (18–30)	0·5% (0·1–1·1)	119 (117–122)	22% (16–29)	0·2% (0·0–0·4)
Central and eastern Europe	117 (111–124)	30% (17–47)	0·9% (0·2–2·2)	119 (113–125)	24% (14–40)	0·3% (0·1–0·9)
East and southeast Asia	115 (112–117)	34% (28–43)	1·3% (0·7–2·0)	119 (114–123)	25% (17–38)	0·4% (0·1–1·0)
Oceania	110 (104–117)	48% (31–63)	2·8% (0·8–5·9)	115 (107–124)	36% (18–59)	1·1% (0·2–3·2)
South Asia	108 (104–111)	53% (43–63)	2·9% (1·8–4·4)	108 (105–113)	52% (40–63)	1·3% (0·7–2·4)
Central Asia, Middle East, and north Africa	114 (111–117)	37% (30–46)	1·1% (0·5–2·0)	117 (113–120)	31% (22–42)	0·4% (0·1–0·8)
Central and west Africa	105 (103–109)	61% (53–66)	3·3% (2·2–4·7)	108 (105–111)	56% (46–62)	1·8% (1·1–3·2)
East Africa	111 (109–114)	46% (41–52)	2·9% (1·9–4·1)	116 (113–118)	36% (30–41)	1·2% (0·8–1·7)
Southern Africa	117 (110–124)	34% (21–51)	1·2% (0·4–2·7)	118 (111–124)	31% (20–48)	0·4% (0·2–0·9)
Andean and central Latin America and Caribbean	115 (112–118)	37% (30–44)	1·4% (0·8–2·3)	119 (116–122)	27% (21–34)	0·3% (0·2–0·6)
Southern and tropical Latin America	115 (106–125)	37% (18–60)	1·3% (0·2–3·8)	117 (108–127)	31% (13–56)	0·5% (0·1–1·7)
Globe	112 (111–113)	43% (39–47)	2·0% (1·5–2·6)	114 (112–116)	38% (34–43)	0·9% (0·6–1·3)

Numbers in parentheses are 95% credibility intervals. Appendix pp 25–30 show mean haemoglobin and anaemia prevalence by country, and appendix pp 32–222 show estimated trends over time and comparisons with original country data.

**Figure 3:**
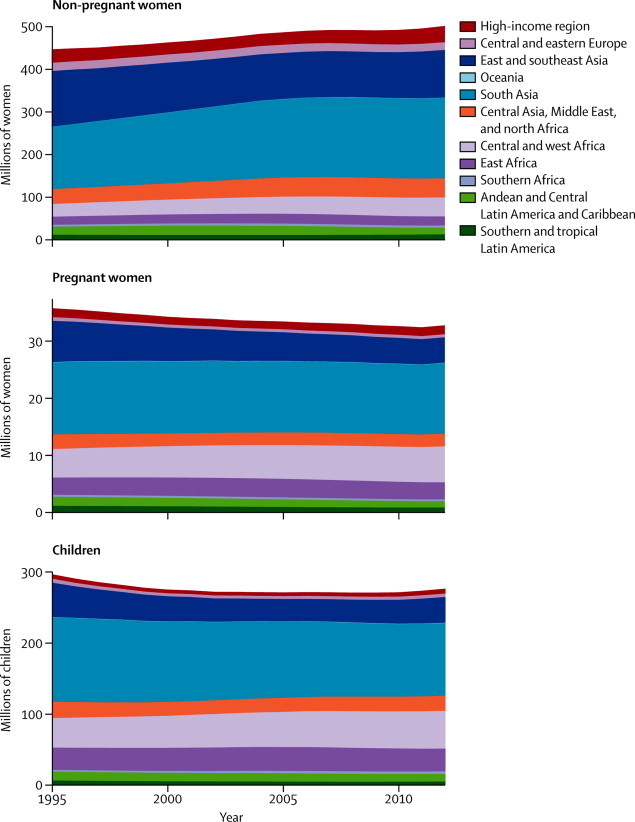
Number (millions) of children and pregnant and non-pregnant women with anaemia, by region Number with total anaemia includes all those below the relevant cutoff value, including those with severe anaemia.

Trends and levels varied substantially across regions and countries. In both 1995 and 2011, central and west Africa and south Asia had the lowest mean concentrations of haemoglobin and the highest anaemia prevalence; east Africa had low haemoglobin concentrations for children (table). Children in these three regions had mean haemoglobin concentrations of 100 g/L or less in 1995, with an anaemia prevalence of at least 70%; prevalence of severe anaemia was almost 10% in these two African regions and 6% in south Asia (table). Between 1995 and 2011, children in these three regions had the largest improvements of any region (table), with improvements in mean haemoglobin of 3·5 g/L or more per decade (posterior probability ≥0·95 for all three regions). Nonetheless, mean concentrations of haemoglobin remained lower than the cutoffs for anaemia in these regions, and anaemia prevalence remained at least 55% (table); however, prevalence of severe anaemia more than halved (table), with an overall shift to the right in haemoglobin distribution. Women in these regions had higher haemoglobin concentrations than children, but mean haemoglobin was less than 110 g/L for pregnant women and 120 g/L for non-pregnant women in central and west Africa and south Asia (table); anaemia prevalence was about 50% in non-pregnant women or above 50% in pregnant women in central and west Africa and south Asia (table). These regions also had the countries with the lowest haemoglobin concentrations and highest anaemia prevalences, including Benin, Burkina Faso, Côte d'Ivoire, Ghana, Guinea, Liberia, Mali, Niger, Senegal, and Togo (appendix pp 25–30). The lowest estimated mean haemoglobin concentrations in our analysis period were less than 90 g/L in children in Burkina Faso in the 1990s, with about 90% of children with anaemia and almost one in six children with severe anaemia (appendix p 59). By 2011, mean haemoglobin in Burkina Faso had improved to only 91 g/L (89–95) and anaemia prevalence in children remained more than 85% (appendix p 59). South Asia had the largest number of children with anaemia in 2011 (102 million [76–121 million]) followed by central and west Africa (53 million [50–55 million]; figure 3). Similarly, the largest number of pregnant women with anaemia lived in these regions (12 million [9–15 million] and 6 million [5–7 million], respectively; figure 3).

**Figure 4:**
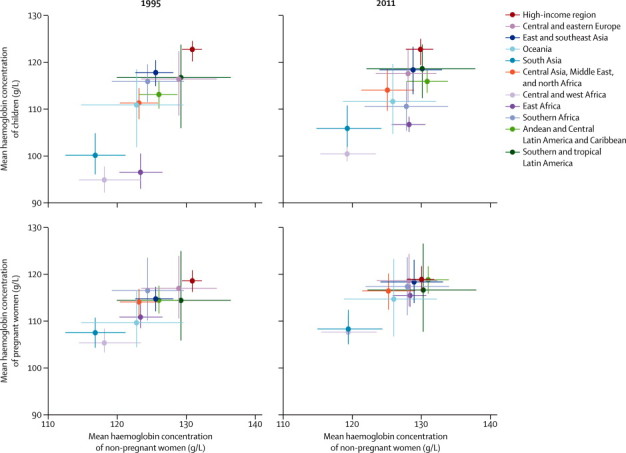
Comparison of mean haemoglobin concentrations of non-pregnant women with those of pregnant women and children in 1995 and 2011 Lines show the 95% credibility interval.

High-income regions, central and eastern Europe, and southern and tropical Latin America had the highest haemoglobin distributions throughout the analysis period. Mean haemoglobin concentrations in these regions were 6–13 g/L above the relevant cutoffs for anaemia, and anaemia prevalence was generally low (table, figure 4). Haemoglobin status did not change in an epidemiologically meaningful or statistically significant way in the regions where concentrations were initially high (table). Although this finding was partly because these regions had the least data, absence of a major trend was also noted in countries with several observations over time, such as Japan, South Korea, and the USA (appendix pp 119, 172, and 214). Other regions with little improvement in haemoglobin included Oceania for children; central and west Africa, and central Asia, Middle East, and north Africa for non-pregnant women; and south Asia for pregnant women (table). Mean haemoglobin concentrations might have declined and anaemia prevalence might have increased in children in southern Africa (posterior probability >0·95). By contrast, women's haemoglobin concentrations improved the most in Andean and central Latin America and Caribbean, east Africa, east and southeast Asia, and Oceania, irrespective of pregnancy status (table). In most of these regions, improvements were more than 2 g/L per decade, and posterior probabilities were greater than 0·85. In particular, because of large improvements, women's haemoglobin concentrations in east and southeast Asia in 2011 were among the highest in the world, similar to or higher than those for Latin America and Caribbean or high-income regions (table).

The concentrations (figure 4) and changes (appendix p 31) in mean haemoglobin concentrations were correlated between pregnant and non-pregnant women across regions, with mean haemoglobin concentrations 8–15 g/L lower during pregnancy in different regions. Trends were less correlated between children and women (appendix p 31).

## Discussion

Our results show that, by 2011, haemoglobin distributions in children and women had improved in some regions where they had been low in the 1990s, leading to a modest global improvement and somewhat smaller differences across regions. The prevalence of severe anaemia is now less than 2·5% in all regions, except among children from central, east, and west Africa. However, no improvement was shown in some groups with low haemoglobin and high anaemia in the 1990s, including in non-pregnant women in central and west Africa and central Asia, Middle East, and north Africa, and pregnant women in southern Africa and south Asia. Children's haemoglobin status might have deteriorated in southern Africa.

Global anaemia prevalence estimated by WHO with data from 1993 to 2005^18^ is consistent with our estimates, as are the broad regional patterns (panel). Our estimated global anaemia prevalence of roughly 30% in non-pregnant women in 2011 is lower than the 40% for 2007 in the 6th report of the UN Standing Committee on Nutrition (UNSCN),^19^ but our 95% credibility intervals for pregnant women (38%, 33–43) and children (43%, 38–47) include their estimates of 41% and 44%, respectively. Furthermore, the Standing Committee reported a smaller global decrease in anaemia prevalence than that noted in our study; they also labelled some countries as having a deteriorating change. The differences in results stem from differences in the scope of analysis, data, and methods. The UNSCN report did not include high-income regions and central and eastern Europe (16% of the global population), which have lower prevalences of anaemia than the global average. We did not include some data sources used in the UNSCN report, either because we did not have access to study meta-information to assess representativeness, or because we assessed these sources as non-representative. More importantly, recent DHS and other surveys have shown lower, and generally declining, prevalences in east African countries—eg, Ethiopia, Malawi, Madagascar, Rwanda, Tanzania, and Uganda—for which our estimates and trends differ the most from those of the UNSCN report. These surveys were not available at the time of the UNSCN analysis, but were used in our analysis.

Panel **Research in context**

**Systematic review**

We searched PubMed with search terms detailed in appendix pp 1–3 for studies published after Jan 1, 1990. In addition to indexed articles, many such comparisons are in national and international agency reports, which we also identified and accessed through requests to national and international organisations.

Some studies have reported anaemia prevalence in individual or several countries,^19^ with some also reporting change across repeated surveys.^20^, ^21^ The Global Burden of Disease Study 1990 quantified the global and regional prevalences of anaemia, with the estimates updated periodically by WHO.^2^, ^18^ These prevalence estimates have been extended to the country level with use of a regression model.^18^ These reports did not analyse the whole distribution of haemoglobin nor did they estimate severe anaemia, even though various cognitive and health effects take place along a continuum of haemoglobin concentrations, with some being greatest in the severe tail of the distribution. Furthermore, the only analysis of how anaemia has changed simply averaged the available surveys in 5 year intervals, but did not formally analyse trends.^19^

**Interpretation**

Our study advances the international reporting and comparison of children's and women's haemoglobin concentrations and anaemia prevalences. We estimated the full population distributions of haemoglobin, for children and women, and by pregnancy status, which allowed for coherent and consistent estimation of mean haemoglobin and of the prevalences of total and severe anaemia between 1995 and 2011 in all countries. In the past few decades, central and west Africa and south Asia have consistently had the lowest concentrations of mean haemoglobin and the highest anaemia prevalences of any region, with east Africa also having low haemoglobin concentrations for children. Haemoglobin distributions in children and women improved in some regions where they had been low in the 1990s, leading to a modest global improvement and somewhat smaller differences across regions. However, some places with low haemoglobin and high anaemia in the 1990s had no or little improvement. Further actions to improve haemoglobin are needed to improve women's and children's health and achieve global targets for reducing anaemia.

Low haemoglobin concentrations can be caused by genetic traits, such as sickle-cell anaemia and thalassaemia;^22^ inadequate bioavailability of dietary iron in foods that are low in iron, folate, or vitamin B12;^23^, ^24^, ^25^, ^26^ malaria;^12^, ^27^ schistosomiasis;^28^ hookworm infection;^29^ HIV infection;^30^ and some non-communicable diseases. Some of these risk factors have been associated with spatial variation in anaemia in west Africa in a cross-sectional analysis.^31^ Previous reports had estimated that about half of anaemia cases worldwide are due to iron deficiency; however, these studies did not assess the role of iron by region.^2^ Iron supplementation increased haemoglobin concentrations in those with anaemia at baseline by an average of 10·17 g/L for pregnant women,^32^ 8·64 g/L for non-pregnant women,^33^ and 8·0 g/L for children^25^ in randomised intervention studies. When these shifts were applied to the estimated haemoglobin concentrations, the proportion of all anaemia amenable to iron was about 50% in non-pregnant and pregnant women and 42% in children. The proportions of severe anaemia amenable to iron were larger: more than 50% for children and non-pregnant women, and more than 60% for pregnant women.

The iron-amenable share of anaemia was largest where fewer other causes of anaemia exist (eg, >55% in pregnant women and children in east and southeast Asia and southern and tropical Latin America, and roughly 70% in the same groups in high-income regions). The share was smallest in regions where other factors contribute to anaemia (eg, <45% in children and non-pregnant women in different parts of sub-Saharan Africa and south Asia); however, because these regions have a large absolute burden of anaemia, the number of people with iron-amenable anaemia is large compared with other regions. The share of severe anaemia amenable to iron was even greater than that for anaemia, ranging from 59% to 70% for pregnant women and from 55% to 70% for children. This finding emphasises the large burden associated with low iron in the most vulnerable groups—namely pregnant women and children. Some of the response to iron supplementation relates to low bioavailability of dietary iron, but attention should also be given to populations affected by hookworm and schistosomiasis, typically rural tropical regions with poor sanitation facilities, especially in east Asia, southeast Asia, and sub-Saharan Africa.^29^, ^34^

The role of malaria in haemoglobin status and anaemia prevalence is likely to be largest in east and west Africa; a systematic review reported that malaria control in endemic areas improved mean haemoglobin by 7·6 g/L in children, and reduced anaemia by 27% and severe anaemia by 60%.^12^ In our results, central and west Africa and east Africa—two of the regions with the lowest concentrations of mean haemoglobin in 2011 in children—contain the countries with the highest rate of *Plasmodium falciparum* infection.^35^ Some of the countries with the lowest concentrations of mean haemoglobin (eg, Burkina Faso and Mali) were also those with the highest rate of *P falciparum* infection.^35^ However, we noted little within-region correlation between the proportion of the population at risk and mean haemoglobin, possibly because other determinants of haemoglobin also vary both between and within countries. Isolation of the independent causal effects of these determinants would need data for various risk factors at fine geographical scale.^31^ Sickle-cell traits and thalassaemias might have a more important role than does malaria in sub-Saharan Africa, parts of central and south Asia, and the Mediterranean.^16^, ^36^

Isolation of the contributions of changes in the above factors to regional trends over time requires detailed data for each of these determinants; such data are rare and should ideally be included in future global databases. Furthermore, important interactions exist between iron and infections, which possibly makes the notion of independent roles irrelevant from clinical and public health perspectives.^37^ The frequency of thalassaemias and sickle-cell anaemia is unlikely to have changed sufficiently to account for trends. Reductions in malaria attributable to the introduction of insecticide-treated bednets^38^ is likely to have contributed to observed improvements in Africa, especially in children whose haemoglobin distribution improved in central, east, and west Africa, as might improved control of hookworm and schistosomiasis^39^ in sub-Saharan Africa and Asia. Improvements in haemoglobin concentrations in children could have been associated with improvements in overall nutritional and anthropometric status, which has improved in all regions except sub-Saharan Africa.^14^ The availability of animal products for human food has increased in east and southeast Asia, which is shown by increases in concentrations of serum cholesterol.^40^, ^41^ Programmes for intermittent iron and folic acid supplementation that target women of reproductive age might have contributed to improvements in some countries, including Laos, Cambodia, and Vietnam.^42^, ^43^, ^44^ However, coverage of iron supplementation during pregnancy in developing countries remains low, because many pregnant women do not attend antenatal clinics or receive sufficient doses of supplement, or because of insufficient emphasis on behavioural aspects of regular supplement use.^45^

Nutritional anaemia should ideally be addressed through dietary diversification and improved access to foods that have high iron bioavailability, including animal products. Other food-based approaches, such as fortification of staple foods and condiments, can also be used. Wheat flour fortification with folic acid and iron is mandated in more than 70 countries, but the extent of implementation varies.^46^ In addition to food-based programmes, daily or intermittent iron supplementation, alone or together with folic acid and other micronutrients, can be used for high-risk groups (ie, children, pregnant women, and women of childbearing age) to improve intakes in countries where they might be deficient in the diet and where losses might be increased—eg, where hookworms and schistosomiasis are prevalent. In some studies, iron supplementation in malaria-endemic settings exacerbated malarial disease and even increased mortality in young children.^47^, ^48^, ^49^ A meta-analysis of supplementation studies^50^ has led to a recommendation to integrate iron provision with malaria prevention and treatment,^51^, ^52^ which can together have synergistically beneficial effects on haemoglobin status. Preventive interventions, including insecticide-treated bednets and intermittent preventive treatment, also improve haemoglobin concentrations of children and pregnant women living in malaria-endemic areas,^1^, ^12^, ^27^ as do improved water and sanitation and deworming in regions where hookworms are common.

The strengths of our study are our extensive data search and rigorous criteria for inclusion of sources; consistent analysis for children and women, and by pregnancy status; estimation of trends by country and region; estimation of the full population distributions of haemoglobin, which is consistent with epidemiological evidence about the harms of low haemoglobin concentrations; and systematic estimation and reporting of uncertainty. The main limitation of our analysis is that, despite the extensive data search and access, fewer data were available for anaemia and haemoglobin than for other nutritional and physiological indicators, such as anthropometric status and blood pressure in women and children,^14^, ^17^, ^53^ especially in the early years of our analysis period. This restriction might have arisen from a scarcity of low-cost portable instruments for field measurement of haemoglobin concentrations. As a result, the estimates might not capture the full variation across countries and regions, and could tend to shrink towards global means when data are sparse. This outcome might have especially affected the estimates in high-income and upper-middle-income countries, where anaemia prevalence is low and typically addressed in a clinical setting. Because our analysis unit was age-country-year, we could use only covariates for which we had data for every country-year, and could not incorporate potentially important predictors of haemoglobin with scarce data, especially dietary iron and iron supplementation. Similarly, although we adjusted haemoglobin for altitude and used data adjusted for smoking when available, we could not do so for inflammation because most surveys did not obtain information about inflammation and because no standard adjustment exists. The adverse effects of low haemoglobin are largest in pregnant women and young children, but there are also effects in other groups, including adolescents, elderly people, and men.^18^ We did not include these groups in our analyses because substantially fewer representative data are available; these other groups could account for 45% of all anaemia cases. Finally, our study focused on national-level patterns of haemoglobin and anaemia; it would be ideal to have information about nutritional indicators at the subnational level—eg, by rural versus urban place of residence, province or state,^54^ or socioeconomic status.^55^

Reduction of anaemia is an important component of women's and children's health. Despite the modest improvements shown, haemoglobin concentrations remain low and anaemia prevalence remains high in the poorest regions of the world, presenting an obstacle to reducing maternal and neonatal mortality and to healthy early childhood development. If present trends are maintained, the probability of halving anaemia from 2011 levels by 2025 in women of reproductive age is less than 25% in all regions individually, and negligible at the global level. Further improvements are likely to need a combination of programmes that address the parasitic disease and nutritional determinants of low haemoglobin and those that scale up supplementation, especially during pregnancy. Beyond mortality outcomes, the cognitive benefits of increased haemoglobin will lead to improved school performance and work productivity, and contribute to better health and economic outcomes throughout the life course.^1^, ^3^, ^56^
