# Supplementary material for: Global, regional, and national trends in haemoglobin concentration and prevalence of total and severe anaemia in children and pregnant and non-pregnant women for 1995–2011: a systematic analysis of population-representative data
Source: Lancet Glob Health. 2013 Jul;1(1):e16–25. doi: 10.1016/S2214-109X(13)70001-9 (PMC4547326; doi:10.1016/S2214-109X(13)70001-9)
Supplement: Supplementary appendix [file mmc1.pdf]

THE LANCET Global Health

## Supplementary appendix

This appendix formed part of the original submission and has been peer reviewed. We post it as supplied by the authors.

Supplement to: Stevens GA, Finucane MM, De-Regil LM, et al. Global, regional, and national trends in haemoglobin concentration and prevalence of total and severe anaemia in children and pregnant and non-pregnant women for 1995–2011: a systematic analysis of population-representative data. *Lancet Glob Health* 2013; **1:** e16–25.

# Webappendix 1. Data identification, access and inclusion

The distribution of blood haemoglobin concentration in a population is commonly summarized as a percentage below a threshold, or a prevalence of anaemia. Mean haemoglobin and its standard deviation may also be reported. Anaemia thresholds typically vary by age, sex, and pregnancy status. Studies may also use different haemoglobin thresholds to define anaemia, and may report multiple anaemia severities, such as mild, moderate and severe anaemia. We accessed data in two forms: 1) anonymised individual-level haemoglobin data when available to the authors, and 2) summary statistics, including mean haemoglobin and anaemia prevalences below specific thresholds. We used anaemia prevalences with any definition in our statistical model described in Webappendix 5, which accounts for the specific thresholds used to define anaemia when using the data.

We included data sources if:

- blood haemoglobin was measured;
- the study reported anaemia or mean haemoglobin for pre-school-aged children or women of reproductive age;
- a probabilistic sampling method with a defined sampling frame was used and data were representative of at least three areas within a country;
- the sample size of the survey or study was at least 100 individuals;
- data were collected in or after 1990; and
- data were from the 190 countries and territories listed in Webtable 1.

Measurement of haemoglobin for children younger than 6 months of age is rare in surveys because it requires a puncture to get blood drops. For this reason, our age group of interest for children was 6-59 months of age. We included data for all pre-school children because some sources did not report data separately for children aged 6-59 from those who were below 6 months or between 60 and 71 months of age. Data sources that did not cover the exact age groups of interest were given smaller weights, as described in Webappendix 5.

We excluded data sources if a facility-based sampling scheme was used. We also excluded women's data if only mothers of children $<$ 5 years of age were included in the sample (e.g., Reproductive Health Surveys) because women who have not had a child in the past 5 years and may have systematically different haemoglobin levels. Finally, we excluded subnational datasources if the subnational area was selected on a variable causally related to anaemia prevalence, e.g., malaria endemicity. We manually identified and removed duplicated data accessed from more than one source. If both individual-level and summarized data were available, we used individual-level data. Our last update date was in the first quarter of 2013.

## 1.1 Individual-level data

We obtained anonymised individual-level data from health-examination surveys and household surveys with haemoglobin measurements. Most of these sources were multi-country surveys including the Demographic and Health Surveys (DHS), Multiple Indicator Cluster Surveys (MICS), Reproductive Health Surveys (RHS) and the Malaria Indicator Surveys (MIS). We also searched websites that archive survey data for health examination surveys that met our inclusion criteria; the websites included the Inter-University Consortium for Political and Social Research (ICPSR), the Institute for Health Metrics and Evaluation's Global Health Data Exchange (GHDx), and the RAND corporation. Finally, we used health examination survey data available to the authors through the Global Burden of Metabolic Risk Factors of Chronic Diseases Collaborating

Group[1,2,3,4]. From each source, we extracted the following variables: age, sex, haemoglobin concentration, pregnancy status, urban or rural residence, altitude, and survey sample weight, stratum, and primary sampling unit.

We only used data for children aged 6 to 59 months and women aged 15 to 49 years. Some haemoglobin concentrations recorded in survey datasets are biologically implausible. We excluded haemoglobin measurements that were less than 25 g/L or greater than 200 g/L. Finally, we adjusted all haemoglobin data for altitude, as described in Webappendix 3.

## 1.2 Data accessed as summary statistics

WHO maintains a database on anaemia prevalence. Data are identified via periodic MEDLINE searches and an international network of collaborators, who uncover data sources not reported in routine databases. The search is limited to humans, and the following search terms are used:

```
(((national) AND (survey)) OR ((population) AND (prevalence))) AND
((iron status) OR (iron deficiency) OR (anemia) OR (anaemia) OR
(ferritin) OR (hemoglobin) OR (haemoglobin) OR (low iron level) OR
(transferrin receptor) OR (insufficient iron))
```

Studies are included in the WHO database if there is a defined population-based sampling frame; a probabilistic sampling procedure is used; and sample size is at least 100 individuals.

We accessed and further screened these summary data using our exclusion criteria (Webappendix 1; Figure 1 in the main paper). Consistent with our inclusion and exclusion criteria, we excluded summarized data sources if:

- blood haemoglobin concentration was not measured, and another measure such as serum ferritin, haematocrit, or previously diagnosed anaemia was reported;
- data were collected prior to 1990 or in a country or territory not listed in Webtable 1;
- we had access to the same data as individual-level records;
- they were not representative of the general population (e.g., were refugees), or non-random sampling methods were used, or sampling methods were not adequately described;
- they used a facility-based surveillance method;
- they were representative of fewer than three areas within a country;
- we were unable to determine whether the data were adjusted for altitude and the data were collected in a high-altitude country;
- data for pre-school aged children were summarized together with data for children older than six years of age without reporting summary statistics in smaller age bands; or data for women of reproductive age were combined with data for children under 10 years of age without reporting summary statistics in smaller age bands; or
- the study did not have data on haemoglobin concentration or anaemia prevalence in children aged 6-59 months or women aged 15-49 years.

In some cases, the sample size was not reported in the WHO anaemia database. We checked all data sources for which sample size was not reported. If we could not find any information on the sample size, we conservatively assumed a sample size of 100.

We also provided a list of data sources in the WHO anaemia database to WHO country offices and to WHO's network of international collaborators, and requested that additional data to be provided. We extracted additional data from reports obtained through this data request, from preliminary DHS reports for surveys that were not yet available as individual-level records, and from reports archived on the Malaria Indicator Surveys.

## Webappendix 2. Pregnancy status in data from household surveys

Blood haemoglobin concentration decreases, initially sharply, during the first trimester of pregnancy as part of a physiological process that occurs in both well-nourished and undernourished women (Webfigure 1).

Most household surveys ask women whether they are pregnant. Data on self-reported pregnancy and reported duration of the pregnancy show that many women do not report pregnancy during the first trimester. Specifically, we would expect more 6-week pregnancies than 8-month pregnancies, since not all pregnancies progress. However, comparison of reported pregnancies of different durations in 43 DHS surveys indicates that approximately 25% of women with a 6-week pregnancy report that they are pregnant, vs. 75% of women with a 10-week pregnancy. In addition to this reporting behaviour, the final 32 weeks of pregnancy coincide with the time during which blood haemoglobin has had its sharpest decline, in low-and-middle-income as well as high-income countries (Webfigure 1). Therefore, our operational definition of pregnancy status was one that began in the eighth week, which minimizes bias in self-reported status with relatively small effect on the role of pregnancy in haemoglobin level. This also meant that in calculating the proportion of women in a country who were pregnant at any time, as below, we used an average pregnancy duration of 32 weeks (gestational weeks 8-40).

We excluded all individual-record observations for women of reproductive age who reported that they did not know their pregnancy status (0.5% of all observations). Five surveys with individual-level data did not record pregnancy status as a part of their design. In these cases, we did not use individual-level data; rather, we calculated summary statistics for the whole sample and used the source in the same manner as data accessed directly as summary statistics, as described below.

When available, we used summary data separately by pregnancy status. Some summarized data were reported for pregnant and non-pregnant women together. Our statistical model used such data as a combination of pregnant and non-pregnant women, as described in Webappendix 5, with the proportion of pregnant women approximated by that of the national population. We calculated the proportion of women in the population of the given country and in a given year who were pregnant at the time of survey, using country- and age-specific data on live births (from the UN Population Division's 2010 Revision) and stillbirths[5], using our operational definition of pregnancy above.

## Webappendix 3. Methods for adjusting haemoglobin for altitude

Haemoglobin needs are greater for those living at high altitudes due to the lower concentration of oxygen in the atmosphere[6]. When altitude measurements corresponding to individual-level observations were available us, we adjusted haemoglobin concentrations using a formula developed by the US Centers for Disease Control and Prevention[6,7] and commonly used in studies worldwide:

$$Hb_{adjusted} = Hb_{unadjusted} + 0.32 \cdot (altitude \cdot 0.0033) - 0.22 \cdot (altitude \cdot 0.0033)^2 \tag{1}$$

where haemoglobin is measured in g/L and altitude is measured in meters above sea level (m.a.s.l.). The adjustment is only applied to individuals living at altitudes over 1,000 m.a.s.l.

We were unable to obtain altitude information for individual subjects for some surveys with individual record data. When the proportion of population living at altitudes above 1500 m.a.s.l. (an altitude at which there is 3 g/L effect on haemoglobin concentration) was less than 5% of total population (hereafter termed low-altitude countries), we included the source. Data sources in this category were from the United States, United Kingdom, Thailand, El Salvador, and Honduras. Data from one individual-record data source without individual-level altitude in a country where more than than 5% of total population lived above 1500 m.a.s.l (2011 Angola Malaria Indicators Survey) were converted to summary statistics and adjusted as described below.

For data available as summary statistics, we determined whether the data were from a low- or high-altitude country. If data were from a low-altitude country, we used the data regardless of adjustment for altitude. We developed regression equations to correct unadjusted summary statistics from high-altitude countries. We pooled data from 31 DHS surveys in countries with some population living over 1000 m.a.s.l. We extracted both adjusted and unadjusted haemoglobin data for all survey participants (both women and children). We calculated mean haemoglobin and prevalence of anaemia (using each of the cutoffs 70, 100, 110, 120, and 130 g/L) using both adjusted and unadjusted data for each of the surveys. We then related altitude-adjusted mean haemoglobin concentration and prevalence of anaemia to the corresponding unadjusted values using separate regressions for each of the six metrics. In particular, for each metric we regressed the adjusted values against the unadjusted values, including as additional covariates the percent of population living over 1,000 m.a.s.l., the percent of population living over 2,000 m.a.s.l., and an interaction between the two. This regression specification was intended to mimic the quadratic relationship in the CDC adjustment (Eq. 1). In the regression for mean haemoglobin, we fixed the coefficient of unadjusted haemoglobin at one to reflect the relationship in (Eq. 1).

We used these regression relationships to predict adjusted mean haemoglobin concentration and adjusted prevalence of anaemia based on unadjusted summary statistics from high-altitude countries. We accounted for uncertainty of this step by calculating the standard regression prediction variance, which accounts for both uncertainty in estimating the regression relationship and variability of individual values around the regression line. This variability from the effect of predicting adjusted country-level metrics was then included in the statistical modeling as an added variance in the likelihood for each summary statistic from these sources.

# Webappendix 4. Accounting for complex survey design

As described in the main paper and Webappendix 5, our statistical model used individual-level data when available and summary statistics when not to estimate the full distributions of blood haemoglobin concentration by country and year.

All of the individual-level data in our analysis came from surveys that used complex survey designs. Specifically, in designing a representative survey, the target populations were usually divided into strata based on geographical regions within the country, whether place of residence was rural or urban, and/or the socioeconomic characteristics of the place of residence; within each stratum, a number of clusters were randomly selected. Clusters may be villages, administrative units, or census units. Households or participants were then randomly sampled within each cluster. Because total population may differ among strata and clusters, individuals or households in smaller units have a higher probability of being selected than those in larger units. To account for the differences in probability of being sampled, each observation is assigned a sample weight. These weights are calculated to make the survey data representative of the total population.

An implication of the sampling method is that the so-called effective sample size of the survey (ESS) is different from its actual sample size. This occurs primarily because the sampled individuals are from a clusters that are representative but do not cover the entire country, and hence contain less information than they would, had they been a true random sample of the population.

To reflect the true availability of information in each survey and in the individual level data that it provided to the statistical model, we estimated ESS based on the `estat effects` command of the Stata version 10.1 `svy` suite of commands (StataCorp, 2009). In particular, this command generates the design effect (DEFF), which is the ratio between the (usually smaller) ESS and the real sample size, e.g. a survey with 1000 subjects with a DEFF of 2.0 has an ESS of 500. The DEFF may differ by summary statistic metrics (mean vs. prevalence below 100 g/L vs. prevalence below 120 g/L) depending on how these indicators, and the metrics are distributed across the strata and clusters. Following previous work[8], for each survey, we calculated the DEFF as the median of those from a range of metrics, specifically, mean haemoglobin concentration and prevalence below $90, 100, 110, 120, 130$ g/L. ESS was then calculated as sample size divided by DEFF.

In our statistical model, we accounted for the difference between the real and effective sample sizes and for the difference in weights for each observation by scaling the weights across all observations in a study to sum to the ESS. These scaled weights were then used to weight the likelihood contributions from each individual. In addition, surveys may over- or under-sample pregnant women relative to their fraction of the population. To account for this, in the statistical model for women, we scaled the weights for each individual such that the sum of the weights for pregnant women was equal to the total ESS for the study multiplied by the proportion of pregnant women in the study; we did the same for and non-pregnant women. This ensured that the sum of weights across all women was equal to the ESS for the study and that the relative weighting of pregnant and non-pregnant women reflected the number of women in each category in the study.

Data sources providing only summary statistics were also from surveys that used complex survey designs, but sample sizes recorded for these data sources are actual sample sizes and not the effective sample sizes. To ensure that the sample sizes used for these sources in the statistical modeling also reflect the complex survey design, we estimated ESS for each study as the actual sample size multiplied by an estimate of the DEFF. Calculating the DEFF requires individual-level data, which by definition is not available for these data sources. Following previous work[8], we used the median DEFF from all surveys with individual-level data. We then used the estimated ESS for each study in deriving the joint normal likelihood for the summary statistics from each study.

# Webappendix 5. Bayesian hierarchical mixture model

## 5.1 Overview of the statistical model

We estimated the complete distributions of blood haemoglobin concentration in each country-year for pregnant women, non-pregnant women, and children, taking a population-based (vs. high-risk-only) approach to risk factors. This approach allows making coherent inference on mean haemoglobin and on the prevalence of anaemia at all levels of severity.

The methods used followed the estimation of the distributions of other nutritional indicators, reported elsewhere[8] with two modifications. First, we used the individual-level data in a weighted likelihood, with weights determined as described in Webappendix 4. This approach accounted for complex survey design while avoiding additional uncertainty introduced by resampling as done in previous work[8]. Second, for women, we fitted a model that stratifies based on pregnancy status. The differences in haemoglobin distributions between pregnant and non-pregnant women are specified by a country-level intercept that is allowed to change linearly over time, reflecting the fact that the difference can changed based on nutrition and antenatal care. We also included an additional study-specific error term for the difference in haemoglobin between pregnant and non-pregnant women, which accounted for the deviation of this difference in each study from the country pattern, for example due to specific design and measurement features of a specific survey. Using this specification, country- and year-specific data on haemoglobin concentrations stratified by pregnancy status inform estimation of the difference between pregnant and non-pregnant women. In years and countries where separate data by pregnancy status were lacking, the difference was informed based on other sources, especially those in the same country or in the same region, with data in similar time periods.

This gives us the following model for women, with $g$ an indicator differentiating pregnant and non-pregnant strata within a study:

$$f_{gi}(z) = \sum_{m=1}^{M+1} w_{mgi}\, \mathcal{N}\left(z \mid \theta_m, \sigma_m^2\right) \tag{2}$$

$$w_{mgi} = \begin{cases} \Phi(\alpha_{mgi}) \prod_{u=1}^{m-1}(1-\Phi(\alpha_{ugi})) & \text{if } m \le M \\ \prod_{u=1}^{M}(1-\Phi(\alpha_{mgi})) & \text{if } m = M+1 \end{cases} \tag{3}$$

$$\alpha_{mgi} = \delta^c_{mj[i]} + (\varphi\delta^c)_{mj[i]} t_i + u_{mj[i]t_i} + \beta_m x_i + a_{mi} + b_{mi} + I_{gi}(\gamma^c_{mj[i]} + (\varphi\gamma^c)_{mj[i]} t_i + c_{mi}) \tag{4}$$

Details on the model specifications and features are provided elsewhere[8]. Briefly, equation 2 describes a finite mixture of $M+1$ normal ($\mathcal{N}$) distributions (or mixture components), where the weights ($w$) on the constituent normal distributions vary across studies. We specified a probit stick-breaking model for the $w$'s in equation 3. This transformation uses the standard normal cumulative distribution function ($\Phi$) to transform $\alpha$'s that range between -$\infty$ and $\infty$ to $w$'s that range between 0 and 1. Specifically, the $\alpha$'s determine the relative weights assigned to each cluster in the following manner: starting with a 'stick' of length one, $\Phi(\alpha_{1gi})$ is the proportion of the stick that we break off and assign to $w_{1gi}$; $\Phi(\alpha_{2gi})$ is the proportion of the remaining stick of length $(1-w_{1gi})$ that we break off and allocate to $w_{2i}$; and so on. Larger values of $\alpha_{mgi}$ thus correspond to higher weights on the $m^{\text{th}}$ mixture component for stratum $g$ in study $i$. The probit stick-breaking transformation therefore allows placing a flexible model on the $\alpha$'s, while ensuring that the $w$'s still add to one, in such a way that large mass in one part of the haemoglobin distribution is balanced by smaller mass in others parts, and vice versa, through exchanges among the constituent mixture components.

In equation 4, $\alpha_{mgi}$ is defined to leverage all available information in making estimates for each country-year-stratum. $\delta^c_{mj[i]}$ is a country-by-component interaction term, determining the baseline weight placed on each of the $M+1$ normal distributions in country $j$. $(\varphi\delta^c)_{mj[i]}$ is a country- and component-specific linear

time effect, determining the linear parts of country $j$'s time trend. Letting $T = 22$ be the total number of analysis years $(1990, 1991, \cdots, 2011)$, the $T$-vector $u_{mj[i]}$ captures smooth nonlinear change over time in country $j$ and mixture component $m$. $\beta_m$ is the effect of time-varying country-level covariates $x$ (described in the main paper) in mixture component $m$. The $a$'s are study-specific random effects, and the $b$'s capture the extra variance of studies that included women under age 15 or over age 50 (or those that did not cover exactly 6-59 months of age in the model for children). The difference between the models for women and children is that for the former, the model includes the additional terms that are multiplied by $I_{gi}$, which is an indicator variable that takes the value one when stratum $g$ contains pregnant women and -1 when stratum $g$ contains non-pregnant women. This indicator multiples a country- and component-specific term, $\gamma^c_{mj[i]}$, that quantifies the overall difference between pregnant and non-pregnant women, a linear time effect for the pregnant/non-pregnant difference, $(\varphi\gamma^c)_{mj[i]}$, and study-specific errors, $c_{mi}$, in the difference. The difference in haemoglobin between pregnant and non-pregnant women was modelled as linear for simplicity and because there are insufficient data to reliably estimate more complex trends in difference.

The hierarchical prior distributions for the country-specific terms and specifications of the study-specific error terms are described in detail in Stevens et al.[8], with the additional terms introduced here, $\gamma^c_{mj[i]}$, $(\varphi\gamma^c)_{mj[i]}$, and $c_{mi}$, treated analogously to $\delta^c_{mj}$, $(\varphi\delta^c)_{mj}$, and $a_{mi}$, respectively.

For data accessed as summary statistics for which pregnant and non-pregnant women are not distinguished, we took the mixture densities for pregnant and for non-pregnant women and combined them into a $(2M + 2)$-component mixture, weighting by the proportion of pregnant women estimated for that country-year, as described earlier.

## 5.2 Computation and inference

We fitted the models via Markov chain Monte Carlo (MCMC), programming the sampler using the statistical computing language R. For parameters without standard conditional distributions, we implemented an adaptive Metropolis within Gibbs algorithm due to Shaby and Wells[9] that tunes proposal covariances automatically to mimic scaled posterior covariances and that scales adapted proposal covariance matrices to obtain theoretically-optimal acceptance rates. To make estimates at the region and global levels for a given year, we calculated population-weighted averages of each region's constituent country-level values. For each model, we ran 10 chains in parallel for 100,000 iterations each, starting from dispersed starting values, discarding the first 40,000 iterations from each chain for burn-in and retaining one in every 240 of the remaining iterations (to reduce storage needs) from each chain for a total of 2,500 iterations with which to generate results. We assessed convergence to the target distribution and mixing of the samplers using standard MCMC diagnostics applied to the primary inferential quantities of interest (the country-year draws of mean haemoglobin and anaemia and severe anaemia prevalences), including estimation of the effective sample size (ESS), a measure of the number of effectively-independent draws from the posterior. 98% of ESS values were above 500. Of those below 500, 67 of the 39,330 values were between 124 and 200, all for pregnant women and mostly for severe anaemia prevalence. The 95% uncertainty intervals for each quantity of interest were calculated as the 2.5th-97.5th percentiles of the 2,500 draws of the quantity.

## References

1. M.M. Finucane, G.A. Stevens, M.J. Cowan, G. Danaei, J.K. Lin, C.J. Paciorek, G.M. Singh, H.R. Gutierrez, Y. Lu, A.N. Bahalim, et al. National, regional, and global trends in body-mass index since 1980: systematic analysis of health examination surveys and epidemiological studies with 960 country-years and 9· 1 million participants. *The Lancet*, 377(9765):557–567, 2011.

2. G. Danaei, M.M. Finucane, Y. Lu, G.M. Singh, M.J. Cowan, C.J. Paciorek, J.K. Lin, F. Farzadfar, Y.H. Khang, G.A. Stevens, et al. National, regional, and global trends in fasting plasma glucose and diabetes prevalence since 1980: systematic analysis of health examination surveys and epidemiological studies with 370 country-years and 2· 7 million participants. *The Lancet*, 378(9785):31–40, 2011.

3. G. Danaei, M.M. Finucane, J.K. Lin, G.M. Singh, C.J. Paciorek, M.J. Cowan, F. Farzadfar, G.A. Stevens, S.S. Lim, L.M. Riley, et al. National, regional, and global trends in systolic blood pressure since 1980: systematic analysis of health examination surveys and epidemiological studies with 786 country-years and 5· 4 million participants. *The Lancet*, 377(9765):568–577, 2011.

4. F. Farzadfar, M.M. Finucane, G. Danaei, P.M. Pelizzari, M.J. Cowan, C.J. Paciorek, G.M. Singh, J.K. Lin, G.A. Stevens, L.M. Riley, et al. National, regional, and global trends in serum total cholesterol since 1980: systematic analysis of health examination surveys and epidemiological studies with 321 country-years and 3· 0 million participants. *The Lancet*, 377(9765):578–586, 2011.

5. S. Cousens, H. Blencowe, C. Stanton, D. Chou, S. Ahmed, L. Steinhardt, A.A. Creanga, Ö. Tunçalp, Z.P. Balsara, S. Gupta, et al. National, regional, and worldwide estimates of stillbirth rates in 2009 with trends since 1995: a systematic analysis. *The Lancet*, 377(9774):1319–1330, 2011.

6. W. Dowdle. CDC criteria for anemia in children and childbearing-aged women. *Morbidity and Mortality Weekly Report*, 38(22):400–404, 1989.

7. World Health Organization. *Haemoglobin concentrations for the diagnosis of anaemia and assessment of severity*. World Health Organization, 2011. URL `http://www.who.int/vmnis/indicators/haemoglobin.pdf`.

8. G.A. Stevens, M.M. Finucane, C.J. Paciorek, S.R. Flaxman, R.A. White, A.J. Donner, and M. Ezzati. Trends in mild, moderate, and severe stunting and underweight, and progress towards MDG 1 in 141 developing countries: a systematic analysis of population representative data. *The Lancet*, 378:31–40, 2012.

9. B. Shaby and M.T Wells. Exploring an adaptive metropolis algorithm. Technical Report 11-14, Duke University, Department of Statistical Science, 2011.

**Webtable 1. Countries and territories in analysis regions.**

| Region | Countries |
|---|---|
| High-income region | Andorra, Australia, Austria, Belgium, Brunei Darussalam, Canada, Cyprus, Denmark, Finland, France, Germany, Greece, Iceland, Ireland, Israel, Italy, Japan, Luxembourg, Malta, Netherlands, New Zealand, Norway, Portugal, Republic of Korea, Singapore, Spain, Sweden, Switzerland, United Kingdom, United States of America |
| Central and Eastern Europe | Albania, Belarus, Bosnia and Herzegovina, Bulgaria, Croatia, Czech Republic, Estonia, Hungary, Latvia, Lithuania, Macedonia (Former Yugoslav Republic of), Moldova, Montenegro, Poland, Romania, Russian Federation, Serbia, Slovakia, Slovenia, Ukraine |
| East and Southeast Asia | Cambodia, China, China (Hong Kong SAR), China (Macao SAR), Democratic People's Republic of Korea, Indonesia, Lao People's Democratic Republic, Malaysia, Maldives, Myanmar, Philippines, Sri Lanka, Taiwan, Thailand, Timor-Leste, Viet Nam |
| Oceania | Fiji, Kiribati, Marshall Islands, Micronesia (Federated States of), Papua New Guinea, Samoa, Solomon Islands, Tonga, Vanuatu |
| South Asia | Afghanistan, Bangladesh, Bhutan, India, Nepal, Pakistan |
| Central Asia, Middle East, and North Africa | Algeria, Armenia, Azerbaijan, Bahrain, Egypt, Georgia, Iran (Islamic Republic of), Iraq, Jordan, Kazakhstan, Kuwait, Kyrgyzstan, Lebanon, Libya, Mongolia, Morocco, Occupied Palestinian Territory, Oman, Qatar, Saudi Arabia, Syrian Arab Republic, Tajikistan, Tunisia, Turkey, Turkmenistan, United Arab Emirates, Uzbekistan, Yemen |
| Central and West Africa | Angola, Benin, Burkina Faso, Cameroon, Cape Verde, Chad, Central African Republic, Congo, Côte d'Ivoire, Democratic Republic of the Congo, Equatorial Guinea, Gabon, Gambia, Ghana, Guinea, Guinea-Bissau, Liberia, Mali, Mauritania, Niger, Nigeria, Senegal, Sierra Leone, São Tomé and Príncipe, Togo |
| East Africa | Burundi, Comoros, Djibouti, Eritrea, Ethiopia, Kenya, Madagascar, Malawi, Mauritius, Mozambique, Rwanda, Somalia, Sudan, Uganda, United Republic of Tanzania, Zambia |
| Southern Africa | Botswana, Lesotho, Namibia, South Africa, Swaziland, Zimbabwe |
| Andean and Central Latin America and Caribbean | Antigua and Barbuda, Bahamas, Barbados, Belize, Bermuda, Bolivia, Colombia, Costa Rica, Cuba, Dominica, Dominican Republic, Ecuador, El Salvador, Grenada, Guatemala, Guyana, Haiti, Honduras, Jamaica, Mexico, Nicaragua, Panama, Peru, Puerto Rico, Saint Lucia, Saint Vincent and the Grenadines, Suriname, Trinidad and Tobago, Venezuela (Bolivarian Republic of) |
| Southern and Tropical Latin America | Argentina, Brazil, Chile, Paraguay, Uruguay |

**Webtable 2.** Characteristics of data sources.

| Country | Year | Administrative level | Sample size (women and children) | Notes | Age range (children) | Age range (women) | Survey (when individual level data available) or source (when summary statistics available) |
|---|---|---|---|---|---|---|---|
| Afghanistan | 2004 | National | 2012 | 2 | 6-59 | 15-50 | Ministry of Public Health of the Islamic Republic of Afghanistan, UNICEF, Centers for Disease Control and Prevention, National Institute for Research on Food and Nutrition. Summary Report of the National Nutrition Survey, Afghanistan, 2004. Afghanistan, Ministry of Public Health of the Islamic Republic of Afghanistan, 2005 |
| Albania | 2008-2009 | National | 9010 | 1 | 6-59 | 15-49 | DHS |
| Angola | 2006-2007 | National | 5262 | 1 | 6-59 | 15-49 | MIS |
| Angola | 2011 | National | 3485 | 3, 4 | 6-59 | | Cosep Consultoria, Consaúde e ICF Macro. Inquérito de Indicadores de Malária em Angola de 2011. Calverton MD, Cosep Consultoria, Consaúde e ICF Macro, 2011 |
| Argentina | 2007 | National | 40781 | 3 | 6-72 | 10-49 | Ministerio de Salud. Encuesta Nacional de Nutrición y Salud: Documento de Resultados. Buenos Aires, Ministerio de Salud, 2007 |
| Armenia | 2000 | National | 7471 | 2 | 6-59 | 15-50 | National Statistical Service [Armenia], Ministry of Health [Armenia], ORC Macro Inc. Armenia Demographic and Health Survey 2000. Calverton, MD, National Statistical Service, 2001 |
| Armenia | 2005 | National | 7224 | 1 | 6-59 | 15-49 | DHS |
| Azerbaijan | 2006 | National | 10049 | 1 | 6-59 | 15-49 | DHS |
| Bahrain | 2002 | National | 384 | 3 | | 14-50 | Al-Dallal ZS, Hussain KM. Impact of the national flour fortification program on the prevalence of iron deficiency and anemia among women at reproductive age in the Kingdom of Bahrain. Kingdom of Bahrain, Ministry of Health, Public Health Directorate, Nutrition Section, 2003 |
| Bangladesh | 2011 | National | 8285 | 1 | 6-59 | 15-49 | DHS |
| Benin | 2001 | National | 5475 | 1 | 6-59 | 15-49 | DHS |
| Benin | 2006 | National | 9516 | 1 | 6-59 | 15-49 | DHS |
| Benin | 2011-2012 | National | 8724 | 2 | 6-59 | 15-49 | Enquête Démographique et de Santé et à Indicateurs Multiples du Benin EDS-MICS-IV 2011-2012, Rapport Préliminaire |
| Bhutan | 2002 | National | 3600 | 2 | 6-61 | 16-46 | Royal Government of Bhutan- Ministry of Health and Education. Anemia among men, women and children in Bhutan: How big is the problem?. Bhutan, Ministry of Health and Education, 2003 |
| Bolivia | 1998 | National | 5379 | 1 | 6-59 | 15-49 | DHS |
| Bolivia | 2003 | National | 9073 | 1 | 6-59 | 15-49 | DHS |
| Bolivia | 2008 | National | 8443 | 1 | 6-59 | 15-49 | DHS |
| Botswana | 1994 | National | 464 | 3 | 6-59 | 15-50 | Ministry of Health [Botswana], UNICEF. Micronutrient malnutrition in Botswana. A national survey to assess the status of iodine, iron, and vitamin A. Gaborone, Ministry of Health, 1996 |
| Brazil | 2006 | National | 3455 | 2 | 6-59 | | MINISTÉRIO DA SAÚDE CENTRO BRASILEIRO DE ANÁLISE E PLANEJAMENTO |
| Brunei Darussalam | 1996-1997 | National | 668 | 2 | | 15-50 | Ministry of Health. National Nutritional Status Survey - 1997. Negara, Ministry of Health, 1997 |
| Burkina Faso | 2003 | National | 7019 | 1 | 6-59 | 15-49 | DHS |
| Burkina Faso | 2010 | National | 14782 | 1 | 6-59 | 15-49 | DHS |
| Burundi | 2003 | National | 2276 | 2, 4 | 0-59 | 15+ | Kimboka S. Burundi National Anaemia Survey. Bujumbura, Burundi, Minstere de la Sante Publique, 2004 |
| Burundi | 2010 | National | 7857 | 1 | 6-59 | 15-49 | DHS |
| Cambodia | 2000 | National | 5211 | 1 | 6-59 | 15-49 | DHS |
| Cambodia | 2005 | National | 11903 | 1 | 6-59 | 15-49 | DHS |
| Cambodia | 2010 | National | 13231 | 1 | 6-59 | 15-49 | DHS |
| Cameroon | 2004 | National | 8933 | 1 | 6-59 | 15-49 | DHS |
| Cameroon | 2011 | National | 13503 | 1 | 6-59 | 15-49 | DHS |
| Central African Republic | 1999 | National | 3781 | 3 | 6-36 | 15-50 | Ministere Delegue a l'Economie au Plan et a la Cooperation Internationale, Ministere de la Sante et de la Population, UNICEF. Enquête nationale sur l'avitaminose A, la carence en fer et la consommation du sel iode [rapport final]. Republique Centrafricaine, 2000 |
| Chile | 2003 | National | 910 | 1 | | 16-49 | Encuesta Nacional de Salud |
| China | 1992 | National | 16898 | 3, 4 | 0-59 | 15-44 | China National Health Survey |

| Country | Year | Administrative level | Sample size (women and children) | Notes | Age range (children) | Age range (women) | Survey (when individual level data available) or source (when summary statistics available) |
|---|---|---|---|---|---|---|---|
| China | 2002 | National | 62429 | 3 | 0-59 | 15-44 | China National Health Survey |
| Colombia | 2005 | National | 9720 | 1 | 12-48 | 15-49 | Encuesta Nacional de Salud |
| Colombia | 2009-2010 | National | 20927 | 3 | 6-59 | 13-49 | Encuesta Nacional de la Situacion Nutricional en Colombia 2010 |
| Congo | 2005 | National | 5347 | 1 | 6-59 | 15-49 | DHS |
| Congo | 2011-2012 | National | 9458 | 2 | 6-59 | 15-49 | Enquête Démographique et de Santé du Congo EDSC-II 2011-2012, Rapport Préliminaire |
| Costa Rica | 1996 | National | 820 | 3 | | 15-45 | Rodriguez S, Blanco A, Cunningham L, Ascencio M, Chavez M, Munoz L. Prevalencia de las anemias nutricionales de mujeres en edad fértil, Costa Rica: encuesta nacional de nutrición, 1996 [Prevalence of nutritional anemia in women of reproductive age, Costa Rica: national nutrition survey, 1996]. Archivos Latinoamericanos de Nutrición, 2001, 51 -1 :19-24. |
| Costa Rica | 1996 | National | 590 | 3 | 12-59 | | Cunningham L, Blanco A, Rodriguez S, Ascencio M. Prevalencia de anemia, deficiencia de hierro y folatos en niños menores de siete años: Costa Rica, 1996 [Prevalence of anemia, iron and folate deficiency in children smaller than seven years : Costa Rica, 1996]. Archivos Latinoamericanos de Nutrición, 2001, 51 -1 :37-43. |
| Côte d'Ivoire | 2009 | National | 1071 | 2 | 6-59 | 15-49 | Enquête nationale sur l’anémie et les carences en vitamine A et fer en Côte d’Ivoire. “Projet Ivoirien de Promotion des Aliments Fortifiés » PIPAF. Rapport final |
| Côte d'Ivoire | 2011-2012 | National | 8034 | 2 | 6-59 | 15-49 | Enquête Démographique et de Santé et à Indicateurs Multiples EDSCI-III, Côte d’Ivoire 2011-2012, Rapport Préliminaire |
| Democratic People's Republic of Korea | 1998 | National | 72 | 3 | | 15-50 | UNICEF, DPRK. The Multiple Indicator Cluster Survey in the Democratic People's Republic of Korea, 1998. Pyongyang, United Nations Children's Fund, 1998 |
| Democratic People's Republic of Korea | 2004 | National | 1185 | 2 | | 20-35 | Central Bureau of Statistics, Institute of Child Nutrition. DPRK 2004 Nutrition assessment report of survey results. Democratic People's Republic of Korea, Central Bureau of Statistics, Institute of Child Nutrition, 2005 |
| Democratic Republic of the Congo | 2007 | National | 8275 | 1 | 6-59 | 15-49 | DHS |
| Egypt | 1997 | National | 276 | 2 | | 16-20 | El-Sahn F, Sallam S, Mandil A, Galal O. Anaemia among Egyptian adolescents: prevalence and determinants. Eastern Mediterranean Health Journal, 2000, 6 (5/6) :1017-1025. |
| Egypt | 2000 | National | 12440 | 1 | 6-59 | 15-49 | DHS |
| Egypt | 2005 | National | 10284 | 1 | 6-59 | 15-49 | DHS |
| El Salvador | 1998 | National | 5069 | 1 | 7-59 | | RHS |
| El Salvador | 2002-2003 | National | 4261 | 1 | 6-59 | | RHS |
| El Salvador | 2008 | National | 3837 | 1 | 6-59 | | RHS |
| Equatorial Guinea | 2004 | National | 523 | 2 | 0-59 | | Nutritional status and its correlates in Equatorial Guinean preschool children: Results form a nationally representative survey. |
| Equatorial Guinea | 2011 | National | NR | 2 | 6-59 | 15-49 | Guinée Équatoriale Enquête Démographique et de Santé 2011, Rapport de synthese |
| Ethiopia | 1990-1994 | Subnational | 1449 | 2, 4 | | 15-50 | Haidar J, Nekatibeb H, Urga K. Iron deficiency anaemia in pregnant and lactating mothers in rural Ethiopia. East African Medical Journal, 1999, 76 -11 :618-622. |
| Ethiopia | 2005 | National | 9606 | 1 | 6-59 | 15-49 | DHS |
| Ethiopia | 2011 | National | 25244 | 1 | 6-59 | 15-49 | DHS |
| Fiji | 1993 | National | 1605 | 3 | 6-59 | 15-50 | Saito S. 1993 national nutrition survey [main report]. Suva, National Food and Nutrition Committee, 1995 |
| Fiji | 2004 | National | 749 | 3 | | 15-44 | 2004 National Nutrition Survey |

| Country | Year | Administrative level | Sample size (women and children) | Notes | Age range (children) | Age range (women) | Survey (when individual level data available) or source (when summary statistics available) |
|---|---|---|---|---|---|---|---|
| Fiji | 2010 | National | 869 | 3 | | 15-44 | Impact of iron fortified flour in child bearing age women in Fiji 2010 report |
| Gabon | 2012 | National | 8837 | 2 | 6-59 | 15-49 | Enquête Démographique et de Santé, Gabon 2012, Rapport Préliminaire |
| Gambia | 1999 | National | 2084 | 2 | 12-72 | 15-50 | Bah A, Semega-Janneh I, Prentice A, Bates C. Nationwide survey on the prevalence of vitamin A and iron deficiency in women and children in the Gambia. Banjul, National Nutrition Agency, 2001 |
| Georgia | 2005 | National | 2651 | 1 | | 15-49 | MICS |
| Ghana | 2003 | National | 8628 | 1 | 6-59 | 15-49 | DHS |
| Ghana | 2008 | National | 7292 | 1 | 6-59 | 15-49 | DHS |
| Guatemala | 1995 | National | 3211 | 2 | 12-59 | 15-45 | Ministerio de Salud Publica y Asistencia Social. Encuesta Nacional de Micronutrientes. Guatemala City, Ministerio de Salud Publica y Asistencia Social, 1996 |
| Guatemala | 2002 | National | 5545 | 1 | 6-59 | | RHS |
| Guatemala | 2008-2009 | National | 8952 | 1 | 6-59 | | RHS |
| Guinea | 2000 | National | 3624 | 3 | 6-59 | 15-50 | Ministère de la Santé Publique [Guinee]. Enquête nationale sur l'anémie ferriprive en Guinée. Rapport Final: résumé. 2001 |
| Guinea | 2005 | National | 6421 | 1 | 6-59 | 15-49 | DHS |
| Guinea | 2012 | National | 7972 | 2 | 6-59 | 15-49 | Enquête Démographique et de Santé et à Indicateurs Multiples EDS-MICS-IV, GUINÉE 2012, Rapport Préliminaire |
| Guyana | 1996-1997 | National | 403 | 3 | 0-59 | 15-31 | Ministry of Health [Guyana], WHO Pan American Health Organization, Caribbean Food and Nutrition Institute. Executive summary micronutrient study report - Guyana. An assessment of the vitamin A, E, beta-carotene, iron and iodine status in the population. Georgetown, Ministry of Health, 1997 |
| Guyana | 2009 | National | 6233 | 1 | 6-59 | 15-49 | DHS |
| Haiti | 2000 | National | 7656 | 1 | 6-59 | 15-49 | DHS |
| Haiti | 2005-2006 | National | 7883 | 1 | 6-59 | 15-49 | DHS |
| Haiti | 2012 | National | 13385 | 2 | 6-59 | 15-49 | Enquête Mortalité, Morbidité et Utilisation des Services EMMUS-V, Haiti 2012, Rapport Préliminaire |
| Honduras | 2001 | National | 4609 | 1 | 11-59 | | RHS |
| Honduras | 2005 | National | 28192 | 1 | 6-59 | 15-49 | DHS |
| India | 1998-1999 | National | 99910 | 1 | 6-35 | 15-49 | DHS |
| India | 2001-2003 | Subnational | 9480 | 3 | 12-59 | 15-50 | National Institute of Nutrition, Indian Council of Medical Research. Prevalence of Micronutrient Deficiencies. National Nutrition Monitoring Bureau (NNMB) Technical Report No. 22. Hyderabad, India, National Institute of Nutrition, 2003 |
| India | 2005-2006 | National | 152434 | 1 | 6-59 | 15-49 | DHS |
| Indonesia | 1997 | Subnational | 9713 | 1 | 6-59 | 15-49 | IFLS 2 |
| Indonesia | 2000 | Subnational | 12919 | 1 | 8-59 | 15-49 | IFLS 3 |
| Indonesia | 2008 | Subnational | 14472 | 1 | 6-59 | 15-49 | IFLS 4 |
| Iran (Islamic Republic of) | 1994-1995 | National | 1430 | 2 | | 15-50 | Ministry of Health and Medical Education, UNICEF. Multi-centre study on iron deficiency anemia among 15 to 49 year old women in the Islamic Republic of Iran. Nutrition Department, Ministry of Health and Medical Education, 1995 |
| Iran (Islamic Republic of) | 2001 | National | 8765 | 1 | 13-59 | 15-49 | Iran Micronutrient Survey 2001 |
| Japan | 1992 | National | NR | 3 | | 30-50 | The National Nutrition Survey (1992, 1993, 1994, 1995) Japan., National Institute of Health and Nutrition. |
| Japan | 1993 | National | NR | 3 | | 30-50 | The National Nutrition Survey (1992, 1993, 1994, 1995) Japan., National Institute of Health and Nutrition. |
| Japan | 1994 | National | NR | 3 | | 20-50 | The National Nutrition Survey (1992, 1993, 1994, 1995) Japan., National Institute of Health and Nutrition. |
| Japan | 1995 | National | NR | 3 | | 20-50 | The National Nutrition Survey (1992, 1993, 1994, 1995) Japan., National Institute of Health and Nutrition. |
| Japan | 2001 | National | 1398 | 3 | | 20-50 | National Nutrition Survey of Japan in 2001 and 2002., National Institute of Health and Nutrition. |

| Country | Year | Administrative level | Sample size (women and children) | Notes | Age range (children) | Age range (women) | Survey (when individual level data available) or source (when summary statistics available) |
|---|---|---|---|---|---|---|---|
| Japan | 2002 | National | 1164 | 3 | | 20-50 | National Nutrition Survey of Japan in 2001 and 2002., National Institute of Health and Nutrition. |
| Japan | 2007 | National | 1028 | 3 | | 20-54 | National Health and Nutrition Survey |
| Jordan | 2002 | National | 2481 | 3 | 12-59 | 15-50 | Ministry of Health [Jordan], WHO, UNICEF, Centers for Disease Control and Prevention. National baseline survey on iron deficiency anemia and vitamin A deficiency. Amman, Ministry of Health, 2002 |
| Jordan | 2002 | National | 4424 | 1 | 6-59 | 15-49 | DHS |
| Jordan | 2007 | National | 13512 | 1 | 6-59 | 15-49 | DHS |
| Jordan | 2009 | National | 11320 | 1 | 6-59 | 15-49 | DHS |
| Jordan | 2010 | National | 2932 | 2 | 12-59 | 15-49 | Jordan Ministry of Health. National Micronutrient Survey Jordan 2010. Amman, Jordan Ministry of Health, 2011 |
| Kazakhstan | 1995 | National | 4398 | 2, 4 | 6-36 | 15-50 | National Institute of Nutrition [Kazakstan], Academy of Preventive Medicine [Kazakstan], Macro International Inc. Kazakhstan Demographic and Health Survey, 1995. Calverton, MD, National Institute of Nutrition/Macro International Inc, 1996 |
| Kazakhstan | 1999 | National | 2738 | 1 | 6-59 | 15-49 | DHS |
| Kenya | 1999 | National | 6253 | 3 | 2-72 | 15-51 | Mwaniki DL, Omwega AM, Muniu EM, Mutunga JN, Akelola R, Shako BR, Gotink MH, Pertet AM. Anaemia and status of iron, vitamin A and zinc in Kenya. The 1999 National Survey. Nairobi, Ministry of Health, 2002 |
| Kenya | 2010 | National | 3940 | 3 | 3-59 | | Kenya Malaria Indicator Survey 2010. Preliminary Report |
| Kuwait | 1995 | National | 980 | 2 | | 14-46 | Al-Awadi F, Amine EK, Goulam Z. Assessment of the nutritional status of vulnerable groups in Kuwait, part 4: anaemia among adult females in Kuwait. Kuwait, Ministry of Health, Food and Nutrition Administration, 1995 |
| Kyrgyzstan | 1997 | National | 4582 | 1 | 6-35 | 15-49 | DHS |
| Lao People's Democratic Republic | 2000 | National | NR | 2 | 0-72 | | Ministry of Health [Lao People's Democratic Republic]. Report on national health survey: health status of the People of LAO PDR. Vientiane, Ministry of Health, 2001 |
| Lao People's Democratic Republic | 2005 | National | 803 | 1 | | 15-49 | MICS |
| Lebanon | 1997-1998 | National | 828 | 3 | 12-72 | 15-50 | Hwalla N, Adra N. Prevalence and selected determinant of iron deficiency anemia in women and under five children in Lebanon. 1998 |
| Lesotho | 2004 | National | 4430 | 1 | 6-59 | 15-49 | DHS |
| Lesotho | 2009 | National | 6021 | 1 | 6-59 | 15-49 | DHS |
| Liberia | 1999 | National | 2283 | 3 | 6-36 | 14-50 | Mulder-Sibanda M, Dahn B, Duworko M, Flomo-Hall M, Benson A, Ortiz J. National Micronutrient Survey. A national prevalence study on vitamin A deficiency, iron deficiency anemia, iodine deficiency. Monrovia, Ministry of Health and Social Welfare, Family Health Division, United Nations Children's Fund, 1999 |
| Liberia | 2009 | National | 4057 | 1 | 6-59 | | MIS |
| Liberia | 2011 | National | 3207 | 1 | 6-59 | | MIS |
| Macedonia (Former Yugoslav Republic of) | 1999 | National | 2097 | 3 | 6-59 | 15-46 | Branca F, Pastore G, Rossi L, Sette S, Stojanovska Ancevska B, Janeva N, Kolevska L, Peova S, Muratovska O, Venovska K. Multiple indicator cluster survey in FYR Macedonia with micronutrient component. Rome, National Institute of Nutrition, 2000 |
| Macedonia (Former Yugoslav Republic of) | 2011 | National | 5195 | 2 | 6-59 | 15-49 | Institute for Public Health. Macedonia National Nutrition Survey 2011. Skopje, Institute for Public Health, 2011 |
| Madagascar | 1997 | National | 5389 | 1 | 6-35 | 15-49 | DHS |
| Madagascar | 2003-2004 | National | 4202 | 1 | 6-59 | 15-49 | DHS |
| Madagascar | 2008-2009 | National | 13842 | 1 | 6-59 | 15-49 | DHS |
| Madagascar | 2011 | National | 6226 | 1 | 6-59 | | MIS |

| Country | Year | Administrative level | Sample size (women and children) | Notes | Age range (children) | Age range (women) | Survey (when individual level data available) or source (when summary statistics available) |
|---|---|---|---|---|---|---|---|
| Malawi | 2004 | National | 5033 | 1 | 6-59 | 15-49 | DHS |
| Malawi | 2010 | National | 2161 | 3 | 0-59 | | Malawi MIS 2010 |
| Malawi | 2010 | National | 11856 | 1 | 6-59 | 15-49 | DHS |
| Maldives | 1994 | National | 3666 | 3 | 6-59 | 15+ | Ministry of Health and Welfare, Department of Public Health. Nutritional status and child feeding practices of Maldivian children - Report of the National Nutrition Survey. 1994 |
| Maldives | 2001 | National | 1361 | 2 | | 15-50 | Minister of Health, Republic of Maldives. Multiple Indicator Cluster Survey (MICS 2), Maldives. Malé, Ministry of Health, 2001 |
| Maldives | 2007 | National | 2504 | 3 | 6-59 | 15-49 | Ministry of Health & Family and UNICEF Maldives. Project Report: National Micronutrient Survey 2007. Male', Ministry of Health & Family, |
| Mali | 2001 | National | 6474 | 1 | 6-59 | 15-49 | DHS |
| Mali | 2006 | National | 8296 | 1 | 6-59 | 15-49 | DHS |
| Mali | 2010 | National | 1760 | 1 | 6-59 | | DHS |
| Marshall Islands | 1994-1995 | Subnational | 904 | 2 | 12-72 | | Palafox NA, Gamble MV, Dancheck B, Ricks MO, Briand K, Semba RD. Vitamin A deficiency, iron deficiency, and anemia among preschool children in the Republic of the Marshall Islands. Nutrition, 2003, 19 -5 :405-408. |
| Mauritius | 1995 | National | 628 | 3 | | 15-51 | Ministry of Health [Mauritius]. A survey of nutrition in Mauritius and Rodrigues (1995) [final report]. Port Louis, Ministry of Health, 1995 |
| Mexico | 1999 | National | 20623 | 1 | 6-59 | 15-49 | Mexico ENN 1999 |
| Mexico | 2002 | National | 10022 | 1 | 12-48 | 15-49 | Mexican Family Life Survey |
| Mexico | 2005-2006 | National | 24191 | 1 | 12-59 | 15-49 | ENSANUT |
| Mexico | 2012 | National | 26323 | 2 | 12-59 | 12-49 | Ensanut 2012 |
| Micronesia (Federated States of) | 1993 | Subnational | 355 | 3 | 24-48 | | Auerbach SB. Maternal-Child Health Survey: Pohnpei, Federated States of Micronesia, 1993 [summary table]. Palikir, Pohnpei, US Public Health Service/Department of Health Services [Federated States of Micronesia], 1993 |
| Micronesia (Federated States of) | 2000 | Subnational | 849 | 3 | 24-59 | 15-50 | Socorro P, Gonzaga C. Results of vitamin A, anemia and blood lead survey among 2-4 year old children and reproductive-aged women in Yap proper and Kosrae State, Federated States of Micronesia. Atlanta, Centers for Disease Control and Prevention, 2000 |
| Moldova | 2005 | National | 8467 | 1 | 6-59 | 15-49 | DHS |
| Mongolia | 2004 | National | 1462 | 3 | 6-59 | 15-50 | Enkhbat S. Third National Nutrition Survey 2004 [personal communication]. Mongolia, Ministry of Health, 2004 |
| Morocco | 2000 | National | 3732 | 3, 4 | 6-59 | 15-50 | Ministère de la Santé [Maroc]. Enquête nationale sur la carence en fer l'utilisation du sel iodé et la supplémentation par la vitamine A, 2000. 2000 |
| Mozambique | 1998 | Subnational | 3611 | 2 | 12-72 | 15-50 | Fildalgo L, Ismael C, Khan S, Ministerio de Saude. Avaliação da deficiencia em micronutrientes a nivel das provincias de C. Delgado, Manica, Gaza e Maputo [Evaluation of micronutrient deficiency in the provinces of C. Delgado, Manica, Gaza and Maputo]. Maputo, Ministerio de Saude, 1999 |
| Mozambique | 2001-2002 | National | 1414 | 2 | 6-59 | 15+ | Ministério da Saúde, Direcção Nacional de Saúde. Inquérito nacional seovre a deficiência de vitamina A, prevalência de anemia e malária em crianças dos 6-59 meses e respectivas mães. Maputo, Instituto Nacional de Saúde, 2003 |
| Mozambique | 2007 | National | 4285 | 3 | 0-59 | 15-49 | Mozambique MIS 2007 |
| Mozambique | 2011 | National | 18459 | 2 | 6-59 | 15-49 | Mozambique Demographic and Health Survey 2011. Preliminary Report. |
| Myanmar | 2001 | National | 1200 | 2 | | 15-45 | National Nutrition Center, Ministry of Health. A study on hemoglobin status and food practices of Myanmar women. Myanmar, National Nutrition Center, Department of Health, 2001 |
| Nepal | 1997-1998 | National | 7518 | 2 | 6-59 | 15-50 | Ministry of Health [Nepal], Child Health Division, New ERA, The Micronutrient Initiative, UNICEF [Nepal], WHO. Nepal Micronutrient Status Survey 1998. Kathmandu, Ministry of Health, 1999 |
| Nepal | 2006 | National | 15961 | 1 | 6-59 | 15-49 | DHS |
| Nepal | 2011 | National | 8490 | 1 | 6-59 | 15-49 | DHS |

| Country | Year | Administrative level | Sample size (women and children) | Notes | Age range (children) | Age range (women) | Survey (when individual level data available) or source (when summary statistics available) |
|---|---|---|---|---|---|---|---|
| New Zealand | 1996-1997 | National | 1088 | 3 | | 15-45 | Russell D, Parnell W, Wilson N, Faed J, Ferguson E, Herbison P, Horwath C, Nye T, Reid P, Walker R, Wilson B, Tukuitonga C. NZ Food: NZ People: key results of the 1997 National Nutrition Survey. New Zealand, Ministry of Health, 1999 |
| Nicaragua | 1993 | National | 3506 | 3 | 12-59 | 15+ | Ministerio de Salud, Direccion General de Promocion de la Salud, Direccion de Nutricion. Encuesta nacional sobre deficiencia de micronutrientes en Nicaragua 1993: resumen ejecutivo [National survey of micronutrient deficiencies in Nicaragua 1993: executive summary]. Managua, Ministerio de Salud, 1994 |
| Nicaragua | 2000 | National | 4453 | 3 | 6-59 | 15-50 | Ministerio de Salud. Encuesta nacional de micronutrientes (ENM 2000) [National survey of micronutrients (ENM 2000)]. Managua, Ministerio de Salud, 2002 |
| Nicaragua | 2003-2005 | National | 2712 | 3 | 6-59 | 15-49 | Sistema integrado de vigilancia de intervenciones nutricionales (SIVIN) |
| Niger | 2006 | National | 7590 | 1 | 6-59 | 15-49 | DHS |
| Niger | 2012 | National | 10025 | 2 | 6-59 | 15-49 | Enquête Démographique et de Santé et à Indicateurs Multiples du NIger EDSN-MICS-IV 2012, Rapport Préliminaire |
| Nigeria | 1992-1993 | National | 1316 | 2 | | 15-46 | Federal Government of Nigeria, UNICEF. The nutritional status of women and children in Nigeria. Lagos, 1994 |
| Nigeria | 1993 | National | 5013 | 3 | 6-72 | 15-46 | Federal Ministry of Health and Social Services, United States Agency for International Development, Vitamin A Field Support Project, Opportunities for Micronutrient Interventions. Nigeria National Micronutrient Survey, 1993. Nigeria, Federal Ministry of Health and Social Services, 1996 |
| Nigeria | 2010 | National | 4930 | 1 | 6-59 | | MIS |
| Occupied Palestinian Territory | 2003 | National | 1106 | 2 | 12-59 | | Salman R. Prevalence of vitamin A deficiency among children, aged 12-59 months, in the West Bank and Gaza Strip. 2004 |
| Oman | 1992 | National | 1691 | 2 | 6-72 | 16+ | Musaiger AO. Health and nutritional status of Omani families. 1992 |
| Oman | 1995 | National | 5015 | 3 | 0-59 | | Al-Riyami A, Ebrahim GJ. Genetic Blood Disorders Survey in the Sultanate of Oman. Journal of Tropical Pediatrics, 2003, 49 :i1-20. |
| Oman | 2000 | National | 1025 | 2 | | 15-20 | Al-Riyami A, Afifi M, Al-Kharusi H, Morsi M. National Health Survey, 2000. Volume II-Reproductive Health Study. Ministry of Health the Sultanate of Oman, 2000 |
| Oman | 2004 | National | 623 | 3 | 6-59 | 15-50 | Ministry of Health of the Sultanate of Oman, UNICEF Muscat, WHO Eastern Mediterranean Regional Office. National micronutrient status and fortified food coverage survey, Oman, 2004. Muscat, Oman, Department of Nutrition, Ministry of Health of the Sultanate of Oman, 2006 |
| Pakistan | 2001 | National | 8216 | 2, 4 | 6-59 | 15+ | Pakistan Institute of Development Economics, Micronutrient Laboratories Aga Khan University, Medical Centre. National Nutrition Survey 2001-2002. Islamabad, Government of Pakistan, Planning Commission, 2003 |
| Pakistan | 2011 | National | 19405 | 1 | 6-59 | 16-49 | Pakistan Nutritional Survey 2011 |
| Panama | 1992 | National | 999 | 2 | 12-59 | | Ministerio de Salud, Departamento de Nutricion y Dietetica. Encuesta nacional de vitamina A 1992 [National survey on vitamin A 1992]. Panamá City, Ministerio de Salud, 1992 |
| Panama | 1999 | National | 2676 | 3 | 12-59 | 15+ | Ministerio de Salud, UNICEF. Encuesta nacional de vitamina A y anemia por deficiencia de hierro [National survey of vitamin A and iron deficiency anemia]. Panama City, Ministerio de Salud, 2000 |
| Peru | 1996 | National | 3377 | 1 | 6-59 | 15-49 | DHS |
| Peru | 2000 | National | 8545 | 1 | 6-59 | 15-49 | DHS |
| Peru | 2003 | National | 5698 | 3 | 12-36 | 15+ | Informe nacional de niveles de hemoglobina y prevalencia de anemia en niños de 12 a 36 meses y mujeres en edad fertil 2003., Instituto Nacional de Salud, Centro Nacional de Alimentación y Nutritción, Dirección Ejecutiva de Vigilancia Alimentar |
| Peru | 2004 | National | 30808 | 2 | 0-59 | 15-50 | Ministerio de Salud Publica, Instituto Nacional de Salud. Monitoreo nacional de indicadores nutricionales 2004. Lima, Peru, Ministerio de Salud Publica, Instituto Nacional de Salud., 2004 |

| Country | Year | Administrative level | Sample size (women and children) | Notes | Age range (children) | Age range (women) | Survey (when individual level data available) or source (when summary statistics available) |
|---|---|---|---|---|---|---|---|
| Peru | 2004-2005 | National | 1505 | 3 | | 20-50 | Cárdenas de Jurado HG, Gutiérrez PAM, Arbieto LR, Tasayco FM. Encuesta Nacional de indicadores nutricionales, bioquimicos, socioeconómicos y culturales rilacionados con las enfermedades crónico degenerativas. Lima, Peru, Ministerio de Salud, 2006 |
| Peru | 2004-2005 | National | 7502 | 1 | 6-59 | 15-49 | DHS |
| Peru | 2006-2008 | National | 26950 | 1 | 6-59 | 15-49 | DHS |
| Peru | 2009 | National | 29588 | 2 | 6-59 | 15-49 | Instituto Nacional de Estadística e Informática. Encuesta Demografica y de Salud Familiar-ENDES Continua 2009: Informe principal. Lima, Instituto Nacional de Estadística e Informática, 2010 |
| Peru | 2010 | National | 29484 | 2 | 6-59 | 15-49 | Instituto Nacional de Estadística e Informática. Encuesta Demografica y de Salud Familiar-ENDES Continua 2010: Informe principal. Lima, Instituto Nacional de Estadística e Informática, 2011 |
| Peru | 2011 | National | 29523 | 2 | 6-59 | 15-49 | Encuesta Demografica y de Salud Familiar-ENDES Continua 2011. Informe principal |
| Philippines | 1993 | National | 3916 | 3 | 6-12 | 15-60 | Fourth National Nutrition Survey, Food and Nutrition Research Institute, Department of Science and Technology |
| Philippines | 1998 | National | 22737 | 2 | 6-72 | 15-50 | Food and Nutrition Research Institute, Department of Science and Technology, UNICEF. Philippine nutrition facts & figures. Manila, Food and Nutrition Research Institute, 2001 |
| Philippines | 2003 | National | 4736 | 3 | 6-59 | 15-50 | Food and Nutrition Research Institute, Philippines. The Sixth National Survey 2003 [personal communication]. Manila, 2003 |
| Qatar | 1995 | National | 1195 | 3 | 6-24 | | Amine EK. Nutritional assessment in Qatar; 1995 Oct 20-Nov 3 [assignment report]. Qatar, WHO Regional Office for the Eastern Mediterranean, 1995 |
| Republic of Korea | 1993 | National | 3172 | 2 | 6-72 | 18+ | Ministry of Health and Welfare. 1993 National Nutrition Survey Report. 1995 |
| Republic of Korea | 1995 | National | 1835 | 3 | | 15-50 | Ministry of Health and Welfare. 1995 National Nutrition Survey Report. Republic of Korea, Ministry of Health and Welfare, 1997 |
| Republic of Korea | 1998 | National | 3228 | 1 | | 15-49 | Korean NHANES 1998 |
| Republic of Korea | 2001 | National | 4430 | 2 | | 15-50 | Korean Ministry of Health and Welfare. The Second Korea National Health and Nutrition Examination Survey, 2001. Seoul, Korean Ministry of Health and Welfare, 2003 |
| Republic of Korea | 2005 | National | 2038 | 1 | | 15-49 | Korean NHANES 2005 |
| Republic of Korea | 2007 | National | 994 | 1 | | 15-49 | Korean NHANES 2007 |
| Republic of Korea | 2008 | National | 2201 | 1 | | 15-49 | Korean NHANES 2008 |
| Republic of Korea | 2009 | National | 2393 | 1 | | 15-49 | Korean NHANES 2009 |
| Rwanda | 1996 | National | 1130 | 2, 4 | 6-59 | 15-50 | Ministère de la Santé, UNICEF, OMS. National Nutrition Survey of Women and Children in Rwanda in 1996 [final report]. Kigali, Ministère de la Santé, 1997 |
| Rwanda | 2005 | National | 9089 | 1 | 6-59 | 15-49 | DHS |
| Rwanda | 2008 | National | 11856 | 1 | 6-59 | 15-49 | DHS |
| Rwanda | 2010 | National | 11122 | 1 | 6-59 | 15-49 | DHS |
| Samoa | 1999 | National | 486 | 3 | 0-59 | 20-50 | Mackerras D, Kiernan DM. Samoa national nutritional survey 1999, part 1: anaemia survey [technical report]. Apia, Department of Health, 2002 |
| Senegal | 2005 | National | 6986 | 1 | 6-59 | 15-49 | DHS |
| Senegal | 2008 | National | 10184 | 1 | 6-59 | 15-49 | MIS |
| Senegal | 2010-2011 | National | 9642 | 1 | 6-59 | 15-49 | DHS |
| Serbia | 2000 | National | 1665 | 2 | 6-59 | 15-50 | Petrovic O, Popovic D, Simic S, Bjeloglav D, Peart G. Multiple Indicator Cluster Survey II. The report for the Federal Republic of Yugoslavia. Belgrade, United Nations Children's Fund, 2000 |
| Sierra Leone | 2008 | National | 5916 | 1 | 6-59 | 15-49 | DHS |
| Somalia | 2001 | Subnational | 784 | 2 | 6-59 | | UNICEF. Anemia survey in Somaliland [report]. 2001 |

| Country | Year | Administrative level | Sample size (women and children) | Notes | Age range (children) | Age range (women) | Survey (when individual level data available) or source (when summary statistics available) |
|---|---|---|---|---|---|---|---|
| Somalia | 2009 | National | 1634 | 3 | 6-59 | 15-49 | National Micronutrient and Anthropometric Nutrition Survey Somalia 2009 |
| South Africa | 1994 | National | 4494 | 3, 4 | 6-72 | | South African Vitamin A Consultation Group (SAVACG). Children aged 6 to 71 months in South Africa, 1994: their anthropometric, vitamin A, iron and immunisation coverage status. Johannesburg, South African Vitamin A Consultative Group, 1995 |
| South Africa | 2005 | National | 2744 | 3, 4 | 12-47 | 15-49 | National Food Consumption Survey-Fortification Baseline (NFCS-FB). Department of Health, Republic of South Africa. |
| Sri Lanka | 1994-1995 | National | 3350 | 3 | 3-59 | 15+ | Mudalige R, Nestel P. Prevalence of anaemia in Sri Lanka. Ceylon Journal of Medical Science, 1996, 39 -1 :9-16. |
| Sri Lanka | 2001 | National | 7849 | 3 | 6-59 | 15-50 | Piyasena C, Mahamithawa AMASB. Assessment of anaemia status in Sri Lanka 2001 [survey report]. Colombo, Ministry of Health, Nutrition and Welfare, Department of Health Services, Medical Research Institute, 2003 |
| Sudan | 1995 | Subnational | 2700 | 2 | 6-59 | 15+ | Federal Ministry of Health, National Nutrition Department, WHO, Ministries of Health, Nutrition Departments Kassala - S. Darfur - N. Kordofan - Red Sea -Gezira -& Nahr El Neil States. Comprehensive Nutrition Survey. Khartoum, Federal Ministry of Health, National Nutrition Department, 1997 |
| Swaziland | 2006 | National | 8446 | 1 | 6-59 | 15-49 | DHS |
| São Tomé and Príncipe | 2008 | National | 4385 | 1 | 6-59 | 15-49 | DHS |
| Taiwan | 1993-1996 | National | 605 | 3, 4 | | 19-44 | NAHSIT 1993-1996 |
| Taiwan | 2005-2008 | National | 261 | 3, 4 | | 19-44 | NAHSIT 2005-2008 |
| Tajikistan | 2003 | National | 3952 | 3 | 6-59 | 15-50 | Branca F, Ferrari M, Rossi L. Micro-nutrient status survey in Tajikistan. Rome, National Institute for Research on Food and Nutrition, Kazakh Academy of Nutrition, 2004 |
| Tajikistan | 2009 | National | 4313 | 2 | 6-59 | 15-49 | Micronutrient Status Survey in Tajikistan, 2009 |
| Thailand | 1995 | National | 5271 | 3 | 0-72 | 15-50 | Ministry of Public Health, Department of Health. The Fourth National Nutrition Survey of Thailand 1995. Bangkok, Ministry of Public Health, Department of Health, 1998 |
| Thailand | 1997 | National | 1405 | 1 | | 15-49 | Thailand NHES 2 |
| Timor-Leste | 2003 | National | 9323 | 2 | 0-59 | 15-50 | Ministry of Health [Timor Leste], University of Newcastle, Australian National University, ACIL. Timor Leste 2003 Demographic and Health Survey. Newcastle, Australia, Ministry of Health/University of Newcastle, 2003 |
| Timor-Leste | 2009 | National | 6735 | 1 | 6-59 | 15-49 | DHS |
| Tunisia | 1996-1997 | National | 2743 | 2 | 0-72 | 20-60 | Ministère de la Santé Publique, Institut National de Nutrition. Rapport national: évaluation de l'état nutritionnel de la population tunisienne [National report: evaluation of the nutritional status of the Tunisian population]. Tunis, Ministère de la Santé Publique, 1996 |
| Turkmenistan | 2000 | National | 10664 | 2 | 0-59 | 15-50 | Gurbansoltan Eje Clinical Research Center for Maternal and Child Health, Ministry of Health and Medical Industry [Turkmenistan], ORC Macro. Turkmenistan Demographic and Health Survey 2000. Calverton, MD, Gurbansoltan Eje Clinical Research Center for Maternal and Child Health, ORC Macro, 2001 |
| Uganda | 2000-2001 | National | 11941 | 1 | 6-59 | 15-49 | DHS |
| Uganda | 2006 | National | 5310 | 1 | 6-59 | 15-49 | DHS |
| Uganda | 2009 | National | 3623 | 1 | 6-59 | | MIS |
| Uganda | 2011 | National | 4880 | 1 | 6-59 | 15-49 | DHS |
| United Kingdom | 1991 | Subnational | 1396 | 1 | | 16-49 | UK HSE 1991-1992 |
| United Kingdom | 1992-1993 | Subnational | 951 | 3 | 18-55 | | Gregory JR, Collins DL, Davies PSW, Hughes JM, Clarke PC. National Diet and Nutrition Survey: children aged 1½ to 4½ years. Volume 1: report of the diet and nutrition survey. London, Her Majesty's Stationery Office, 1995 |
| United Kingdom | 1993 | Subnational | 3269 | 1 | | 16-49 | UK HSE 1993 |
| United Kingdom | 1994 | Subnational | 3175 | 1 | | 16-49 | UK HSE 1994 |
| United Kingdom | 1995 | Subnational | 2034 | 1 | | 20-49 | Scottish Health Survey 1995 |
| United Kingdom | 1997 | Subnational | 169 | 3 | | 15-19 | Gregory J, Lowe S, Bates CJ, Prentice A, Jackson LV, Smithers G, Wenlock R, Farron M. National Diet and Nutrition Survey: young people aged 4 to 18 years. Volume 1: report of the diet and nutrition survey. London, Her Majesty's Stationery Office, 2000 |

| Country | Year | Administrative level | Sample size (women and children) | Notes | Age range (children) | Age range (women) | Survey (when individual level data available) or source (when summary statistics available) |
|---|---|---|---|---|---|---|---|
| United Kingdom | 1997 | Subnational | 212 | 1 | | 18-24 | UK HSE 1997 |
| United Kingdom | 1998 | Subnational | 3034 | 1 | | 15-49 | UK HSE 1998 |
| United Kingdom | 1998 | Subnational | 1876 | 1 | | 15-49 | Scottish Health Survey 1998 |
| United Kingdom | 2000-2001 | Subnational | 486 | 3 | | 19-50 | Ruston D, Hoare J, Henderson L, Gregory J, Bates CJ, Prentice A, Birch M, Swan G, Farron M. The National Diet & Nutrition Survey: adults aged 19 to 64 years. Volume 4: nutritional status (anthropometry and blood analytes), blood pressure and physical activity. London, Her Majesty's Stationery Office, 2004 |
| United Kingdom | 2001 | Subnational | 390 | 1 | | 15-24 | UK HSE 2001 |
| United Kingdom | 2002 | Subnational | 946 | 1 | | 15-24 | UK HSE 2002 |
| United Kingdom | 2006 | Subnational | 1990 | 1 | | 16-49 | UK HSE 2006 |
| United Republic of Tanzania | 2004 | National | 17658 | 1 | 6-59 | 15-49 | DHS |
| United Republic of Tanzania | 2007 | National | 6492 | 1 | 6-59 | | AIS/MIS |
| United Republic of Tanzania | 2010 | National | 16995 | 1 | 6-59 | 15-49 | DHS |
| United States of America | 1988-1991 | National | 3878 | 1 | 12-59 | 15-49 | NHANES III |
| United States of America | 1991-1994 | National | 4804 | 1 | 12-59 | 15-49 | NHANES III |
| United States of America | 1999-2000 | National | 2368 | 1 | 12-59 | 15-49 | NHANES 1999-2000 |
| United States of America | 2001-2002 | National | 2754 | 1 | 12-59 | 15-49 | NHANES 2001-2002 |
| United States of America | 2003-2004 | National | 2514 | 1 | 12-59 | 15-49 | NHANES 2003-2004 |
| United States of America | 2005-2006 | National | 2740 | 1 | 12-59 | 15-49 | NHANES 2005-2006 |
| United States of America | 2007-2008 | National | 2255 | 1 | 12-59 | 15-49 | NHANES 2007-2008 |
| United States of | 2009-2010 | National | 2564 | 1 | 12-59 | 15-49 | NHANES 2009-2010 |
| Uzbekistan | 1996 | National | 5180 | 1 | 6-35 | 15-49 | DHS |
| Uzbekistan | 2002 | National | 1207 | 2 | 6-36 | | Ministry of Health, Analytical and Information Center [Uzbekistan], Ministry of Macroeconomics and Statistics, State Department of Statistics [Uzbekistan], ORC Macro. Uzbekistan Health Examination Survey 2002. Calverton, MD, ORC Macro, 2004 |
| Vanuatu | 1996 | National | 1919 | 2 | | 15-50 | Harvey P, Carlot M, Menere R. Report of the second national nutrition survey 1996. Port Vila, Department of Health, 1998 |
| Vanuatu | 2007 | National | 904 | 1 | 6-59 | 15-49 | MICS |
| Viet Nam | 1995 | National | NR | 2 | 26451 | 15+ | Yip R. Final report of the 1995 Viet Nam National Nutrition Anemia and Intestinal Helminth Survey: a recommended plan of action for the control of iron deficiency for Viet Nam. Jakarta, United Nations Children's Fund [Indonesia], 1996 |
| Viet Nam | 2000-2001 | National | 16903 | 3 | 0-59 | 15-50 | Khoi HH, Khan NC, Tam NC, Mai LB, Hao LQ, Thuy PV, Ninh NX, Do TT, Quang ND. Report on Vietnam National anemia Survey, 2000. Hanoi, National Institute of Nutrition, 2001 |
| Viet Nam | 2010 | National | 1523 | 2 | | 15-49 | Laillou, A. et al. 2012. Micronutrient deficits are still publich health issues among women and young children in Vietnam. Plos One 7(4):e34906. |
| Zambia | 1998 | National | 2925 | 2 | 6-59 | 15+ | Luo C, Mwela CM, Campbell J. National baseline survey on prevalence and aetiology of anaemia in Zambia: a random cluster community survey involving children, women and men. Lusaka, National Food and Nutrition Commission., 1999 |

| Country | Year | Administrative level | Sample size (women and children) | Notes | Age range (children) | Age range (women) | Survey (when individual level data available) or source (when summary statistics available) |
|---|---|---|---|---|---|---|---|
| Zambia | 2003 | National | 1347 | 3 | 6-59 | 15-50 | Micronutrient Operational Strategies and Technologies (MOST), UNICEF, Centers for Disease Control and Prevention, Food and Nutrition Commision of Zambia, University of Zambia. Report of the national survery to evaluate the impact of vitamin A interventions in Zambia, July and November 2003. Zambia, Micronutrient Operational Strategies and Technologies, United States Agency for International Development (USAID) Micronutrient Program, 2003 |
| Zambia | 2006 | National | 1927 | 3 | 0-59 | | Zambia MIS 2006 |
| Zambia | 2008 | National | 3086 | 3 | 0-59 | | Zambia MIS 2008 |
| Zambia | 2010 | National | 3162 | 3 | 0-59 | | Zambia MIS 2010 |
| Zimbabwe | 1997 | Subnational | 2345 | 2 | 12-59 | 15-50 | Sikosana PLN, Bhebhe S, Katuli S. A prevalence survey of iron deficiency and iron deficiency anaemia in pregnant and lactating women, adult males and pre-school children in Zimbabwe. Central African Journal of Medicine, 1998, 44 -12 :297-305. |
| Zimbabwe | 1999 | National | 1197 | 2 | 12-72 | 15-50 | Zimbabwe National Micronutrient Survey: 1999, Ministry of Health and Child Welfare, Nutrition Unit |
| Zimbabwe | 2005-2006 | National | 11980 | 1 | 6-59 | 15-49 | DHS |
| Zimbabwe | 2010-2011 | National | 12715 | 1 | 6-59 | 15-49 | DHS |

For the indicator column: 1 = individual record data available, 2 = prevalence of anaemia reported, 3 = mean haemoglobin and prevalence of anaemia reported, 4 = Adjustment for altitude was done on summary statistics (vs. on individual-level data)

**Webfigure 1: The relationship between gestational month and mean haemoglobin concentration. Median haemoglobin concentration and its interquartile range are shown by month of pregnancy, for Demographic and Health Surveys (DHS, from low- and middle-income countries) and the US National Health and Nutrition Examination Survey (NHANES, a high-income country). The graph shows that the shape of the decline in haemoglobin with gestational age is similar between the two populations, even though the NHANES sample has higher haemoglobin than the DHS sample.**

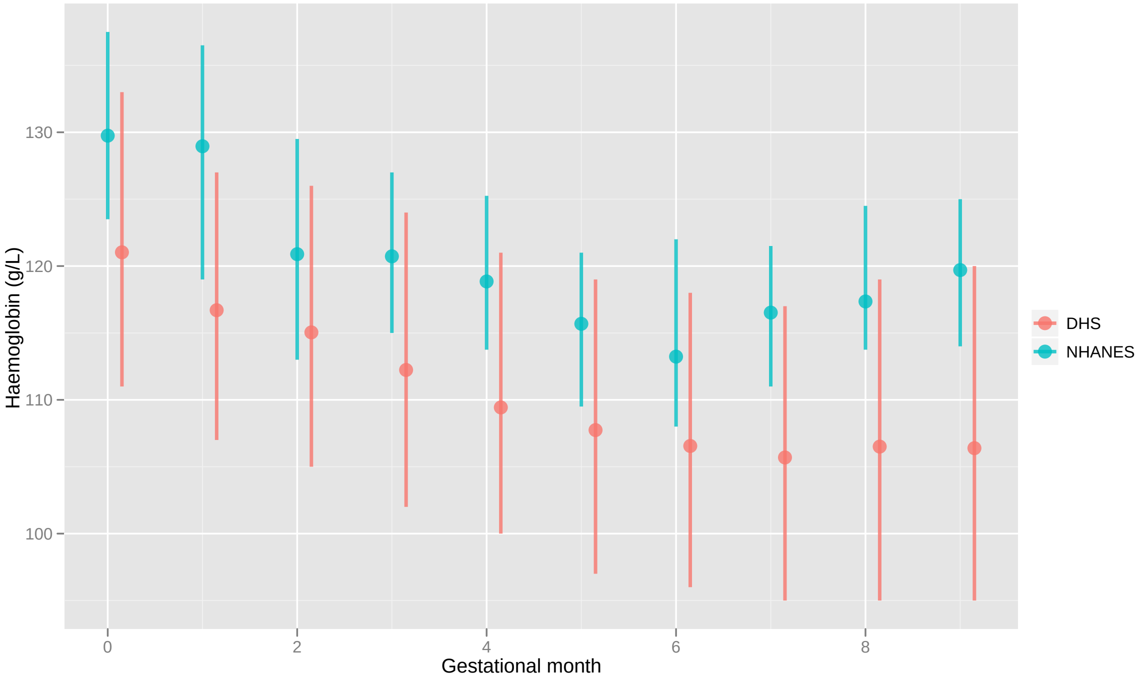

**Webfigure 2: Comparison of mean haemoglobin concentration in male vs. female children under 5 years of age.**

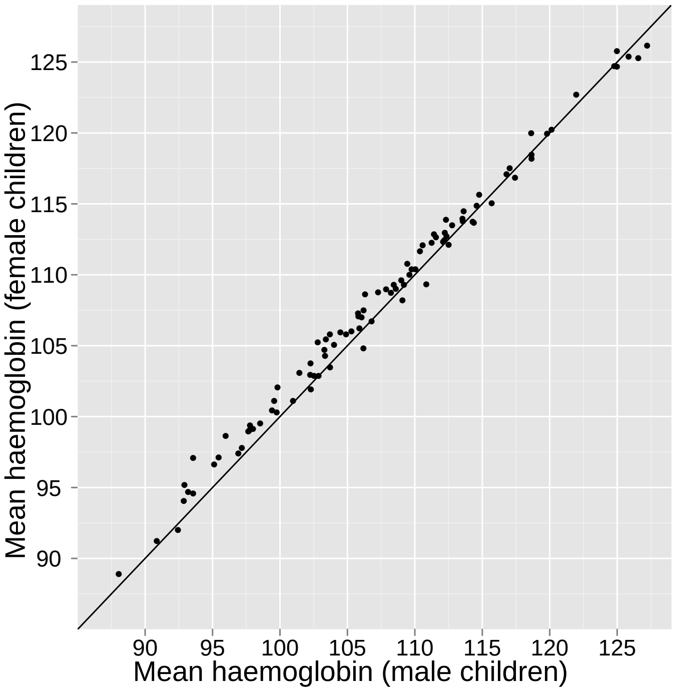

**Webfigure 3: Data sources available by region, year, and the level of representativeness (national vs. regional) for children.**

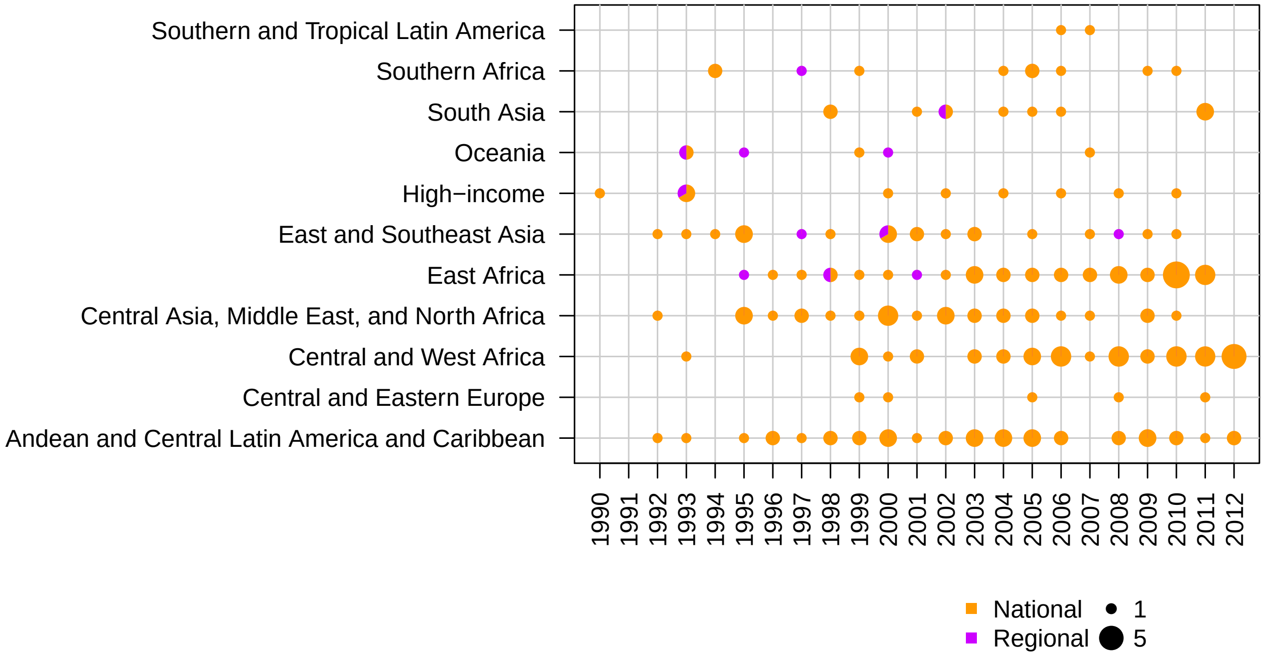

**Webfigure 4: Data sources available by region, year, and the level of representativeness (national vs. regional) for women of reproductive age.**

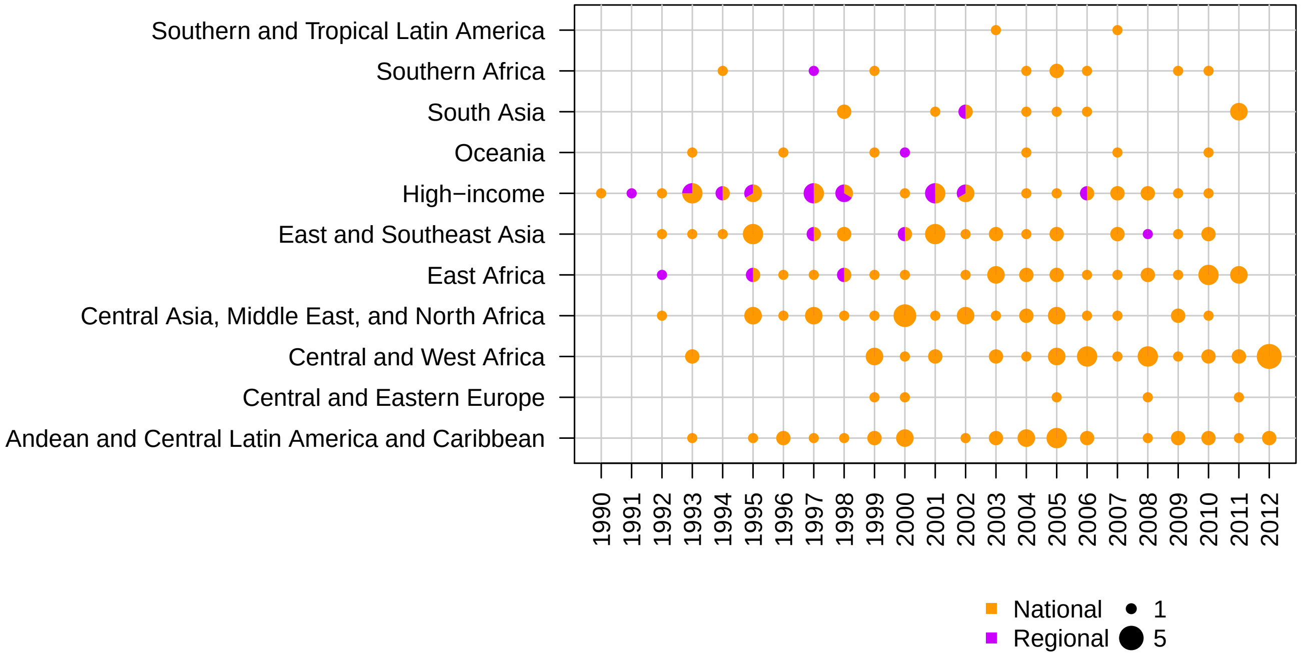

**Webfigure 5: Mean haemoglobin concentration and prevalences of anaemia and of severe anaemia by country, children 6-59 months, 2011. The horizontal line shows the uncertainty interval; as defined in Methods, the uncertainty intervals represent the 2.5th-97.5th percentiles of the 2,500 posterior MCMC draws. *Countries with no data for which the estimates are based on data in all other countries and on the country-specific covariates that are used in the model.**

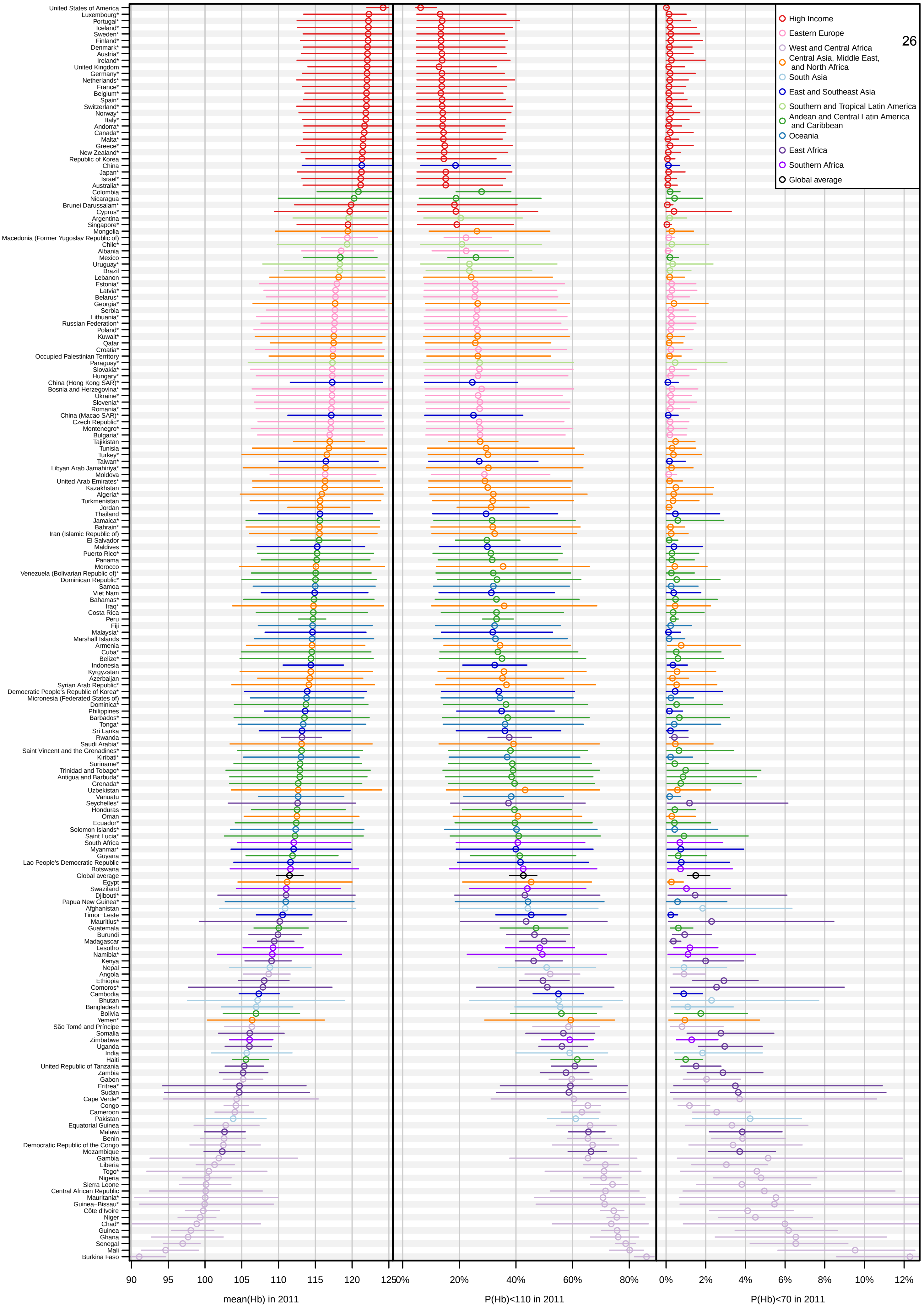
United States of America
Luxembourg*
Portugal*
Iceland*
Sweden*
Finland*
Denmark*
Austria*
Ireland*
United Kingdom
Germany*
Netherlands*
France*
Belgium*
Spain*
Switzerland*
Norway*
Italy*
Andorra*
Canada*
Malta*
Greece*
New Zealand*
Republic of Korea
China
Japan*
Israel*
Australia*
Colombia
Nicaragua
Brunei Darussalam*
Cyprus*
Argentina
Singapore*
Mongolia
Macedonia (Former Yugoslav Republic of)
Chile*
Albania
Mexico
Uruguay*
Brazil
Lebanon
Estonia*
Latvia*
Belarus*
Georgia*
Serbia
Lithuania*
Russian Federation*
Poland*
Kuwait*
Qatar
Croatia*
Occupied Palestinian Territory
Paraguay*
Slovakia*
Hungary*
China (Hong Kong SAR)*
Bosnia and Herzegovina*
Ukraine*
Slovenia*
Romania*
China (Macao SAR)*
Czech Republic*
Montenegro*
Bulgaria*
Tajikistan
Tunisia
Turkey*
Taiwan*
Libyan Arab Jamahiriya*
Moldova
United Arab Emirates*
Kazakhstan
Algeria*
Turkmenistan
Jordan
Thailand
Jamaica*
Bahrain*
Iran (Islamic Republic of)
El Salvador
Maldives
Puerto Rico*
Panama
Morocco
Venezuela (Bolivarian Republic of)*
Dominican Republic*
Samoa
Viet Nam
Bahamas*
Iraq*
Costa Rica
Peru
Fiji
Malaysia*
Marshall Islands
Armenia
Cuba*
Belize*
Indonesia
Kyrgyzstan
Azerbaijan
Syrian Arab Republic*
Democratic People's Republic of Korea*
Micronesia (Federated States of)
Dominica*
Philippines
Barbados*
Tonga*
Sri Lanka
Rwanda
Saudi Arabia*
Saint Vincent and the Grenadines*
Kiribati*
Suriname*
Trinidad and Tobago*
Antigua and Barbuda*
Grenada*
Uzbekistan
Vanuatu
Seychelles*
Honduras
Oman
Ecuador*
Solomon Islands*
Saint Lucia*
South Africa
Myanmar*
Guyana
Lao People's Democratic Republic
Botswana
Global average
Egypt
Swaziland
Djibouti*
Papua New Guinea*
Afghanistan
Timor−Leste
Mauritius*
Guatemala
Burundi
Madagascar
Lesotho
Namibia*
Kenya
Nepal
Angola
Ethiopia
Comoros*
Cambodia
Bhutan
Bangladesh
Bolivia
Yemen*
São Tomé and Príncipe
Somalia
Zimbabwe
Uganda
India
Haiti
United Republic of Tanzania
Zambia
Gabon
Eritrea*
Sudan
Cape Verde*
Congo
Cameroon
Pakistan
Equatorial Guinea
Malawi
Benin
Democratic Republic of the Congo
Mozambique
Gambia
Liberia
Togo*
Nigeria
Sierra Leone
Central African Republic
Mauritania*
Guinea−Bissau*
Côte d'Ivoire
Niger
Chad*
Guinea
Ghana
Senegal
Mali
Burkina Faso
High Income
Eastern Europe
West and Central Africa
Central Asia, Middle East, and North Africa
South Asia
East and Southeast Asia
Southern and Tropical Latin America
Andean and Central Latin America and Caribbean
Oceania
East Africa
Southern Africa
Global average
90 95 100 105 110 115 120 125
mean(Hb) in 2011
0% 20% 40% 60% 80%
P(Hb)<110 in 2011
0% 2% 4% 6% 8% 10% 12%
P(Hb)<70 in 2011

**Webfigure 6a: Mean haemoglobin concentration and prevalences of anaemia and of severe anaemia by country, non-pregnant women 15-49 years, 2011. The horizontal line shows the uncertainty interval; as defined in Methods, the uncertainty intervals represent the 2.5th-97.5th percentiles of the 2,500 posterior MCMC draws. *Countries with no data for which the estimates are based on data in all other countries and on the country-specific covariates that are used in the model.**

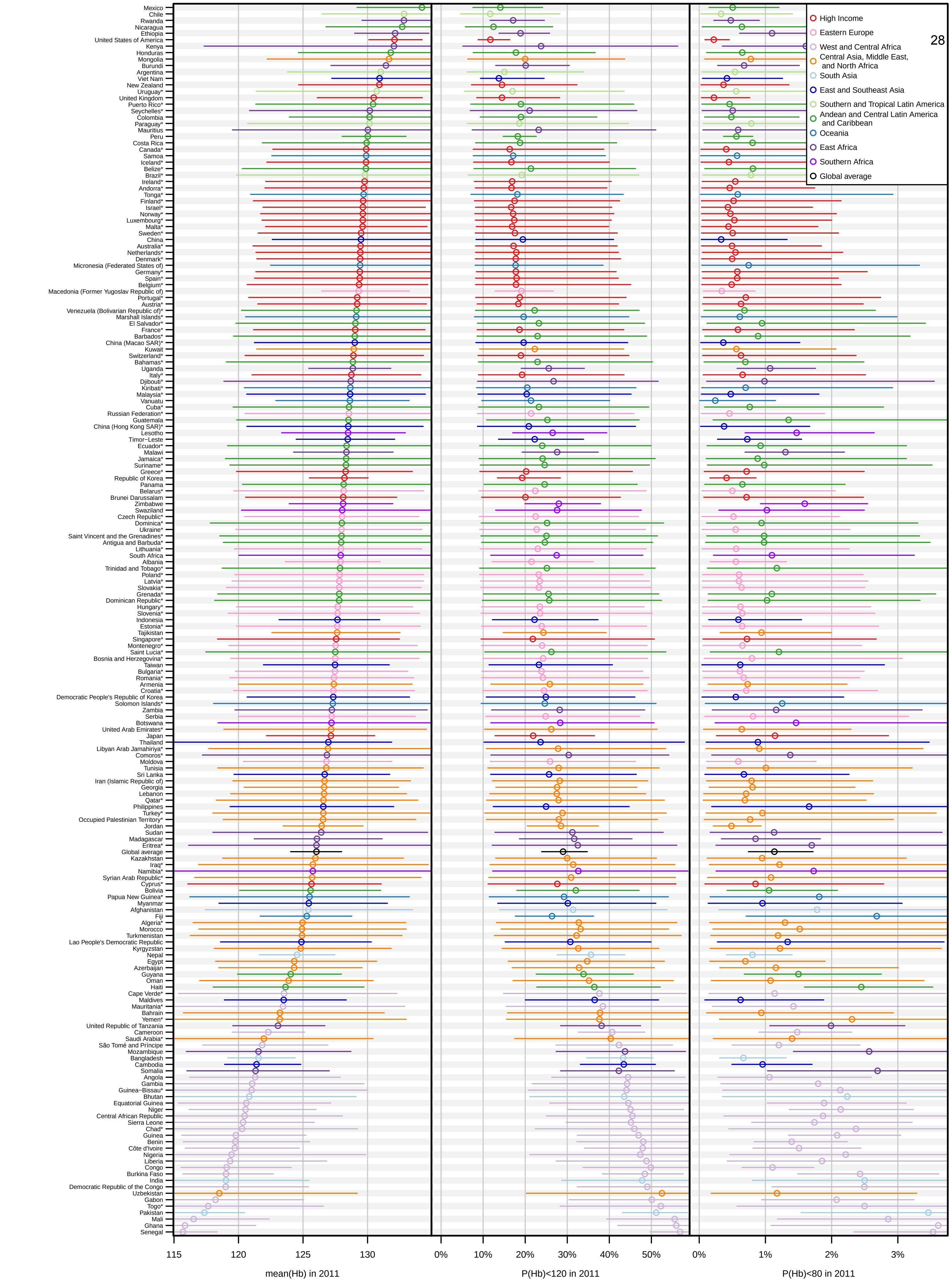

Mexico
Chile
Rwanda
Nicaragua
Ethiopia
United States of America
Kenya
Honduras
Mongolia
Burundi
Argentina
Viet Nam
New Zealand
Uruguay*
United Kingdom
Puerto Rico*
Seychelles*
Colombia
Paraguay*
Mauritius
Peru
Costa Rica
Canada*
Samoa
Iceland*
Belize*
Brazil*
Ireland*
Andorra*
Tonga*
Finland*
Israel*
Norway*
Luxembourg*
Malta*
Sweden*
China
Australia*
Netherlands*
Denmark*
Micronesia (Federated States of)
Germany*
Spain*
Belgium*
Macedonia (Former Yugoslav Republic of)
Portugal*
Austria*
Venezuela (Bolivarian Republic of)*
Marshall Islands*
El Salvador*
France*
Barbados*
China (Macao SAR)*
Kuwait
Switzerland*
Bahamas*
Uganda
Italy*
Djibouti*
Kiribati*
Malaysia*
Vanuatu
Cuba*
Russian Federation*
Guatemala
China (Hong Kong SAR)*
Lesotho
Timor−Leste
Ecuador*
Malawi
Jamaica*
Suriname*
Greece*
Republic of Korea
Panama
Belarus*
Brunei Darussalam
Zimbabwe
Swaziland
Czech Republic*
Dominica*
Ukraine*
Saint Vincent and the Grenadines*
Antigua and Barbuda*
Lithuania*
South Africa
Albania
Trinidad and Tobago*
Poland*
Latvia*
Slovakia*
Grenada*
Dominican Republic*
Hungary*
Slovenia*
Indonesia
Estonia*
Tajikistan
Singapore*
Montenegro*
Saint Lucia*
Bosnia and Herzegovina*
Taiwan
Bulgaria*
Romania*
Armenia
Croatia*
Democratic People's Republic of Korea
Solomon Islands*
Zambia
Serbia
Botswana
United Arab Emirates*
Japan
Thailand
Libyan Arab Jamahiriya*
Comoros*
Moldova
Tunisia
Sri Lanka
Iran (Islamic Republic of)
Georgia
Lebanon
Qatar*
Philippines
Turkey*
Occupied Palestinian Territory*
Jordan
Sudan
Madagascar
Eritrea*
Global average
Kazakhstan
Iraq*
Namibia*
Syrian Arab Republic*
Cyprus*
Bolivia
Papua New Guinea*
Myanmar
Afghanistan
Fiji
Algeria*
Morocco
Turkmenistan
Lao People's Democratic Republic
Kyrgyzstan
Nepal
Egypt
Azerbaijan
Guyana
Oman
Haiti
Cape Verde*
Maldives
Mauritania*
Bahrain
Yemen*
United Republic of Tanzania
Cameroon
Saudi Arabia*
São Tomé and Príncipe
Mozambique
Bangladesh
Cambodia
Somalia
Angola
Gambia
Guinea−Bissau*
Bhutan
Equatorial Guinea
Niger
Central African Republic
Sierra Leone
Chad*
Guinea
Benin
Côte d'Ivoire
Nigeria
Liberia
Congo
Burkina Faso
India
Democratic Republic of the Congo
Uzbekistan
Gabon
Togo*
Pakistan
Mali
Ghana
Senegal
High Income
Eastern Europe
West and Central Africa
Central Asia, Middle East, and North Africa
South Asia
East and Southeast Asia
Southern and Tropical Latin America
Andean and Central Latin America and Caribbean
Oceania
East Africa
Southern Africa
Global average
115
120
125
130
mean(Hb) in 2011
0%
10%
20%
30%
40%
50%
P(Hb)<120 in 2011
0%
1%
2%
3%
P(Hb)<80 in 2011

**Webfigure 6b: Mean haemoglobin concentration and prevalences of anaemia and of severe anaemia by country, pregnant women 15-49 years, 2011. The horizontal line shows the uncertainty interval; as defined in Methods, the uncertainty intervals represent the 2.5th-97.5th percentiles of the 2,500 posterior MCMC draws. *Countries with no data for which the estimates are based on data in all other countries and on the country-specific covariates that are used in the model.**

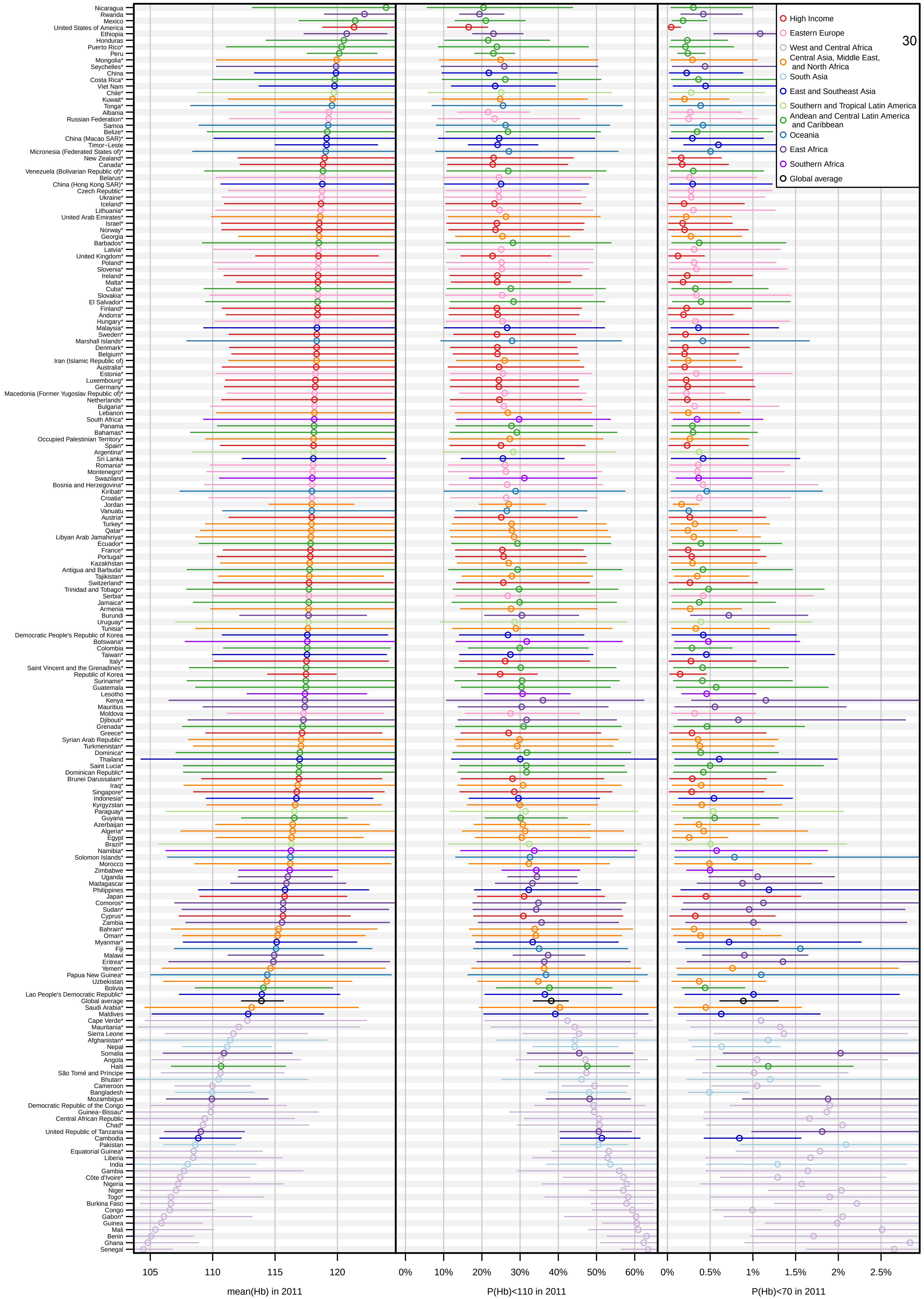
Nicaragua
Rwanda
Mexico
United States of America
Ethiopia
Honduras
Puerto Rico*
Peru
Mongolia*
Seychelles*
China
Costa Rica*
Viet Nam
Chile*
Kuwait*
Tonga*
Albania
Russian Federation*
Samoa
Belize*
China (Macao SAR)*
Timor−Leste
Micronesia (Federated States of)*
New Zealand*
Canada*
Venezuela (Bolivarian Republic of)*
Belarus*
China (Hong Kong SAR)*
Czech Republic*
Ukraine*
Iceland*
Lithuania*
United Arab Emirates*
Israel*
Norway*
Georgia
Barbados*
Latvia*
United Kingdom*
Poland*
Slovenia*
Ireland*
Malta*
Cuba*
Slovakia*
El Salvador*
Finland*
Andorra*
Hungary*
Malaysia*
Sweden*
Marshall Islands*
Denmark*
Belgium*
Iran (Islamic Republic of)
Australia*
Estonia*
Luxembourg*
Germany*
Macedonia (Former Yugoslav Republic of)*
Netherlands*
Bulgaria*
Lebanon
South Africa*
Panama
Bahamas*
Occupied Palestinian Territory*
Spain*
Argentina*
Sri Lanka
Romania*
Montenegro*
Swaziland
Bosnia and Herzegovina*
Kiribati*
Croatia*
Jordan
Vanuatu
Austria*
Turkey*
Qatar*
Libyan Arab Jamahiriya*
Ecuador*
France*
Portugal*
Kazakhstan
Antigua and Barbuda*
Tajikistan*
Switzerland*
Trinidad and Tobago*
Serbia*
Jamaica*
Armenia
Burundi
Uruguay*
Tunisia*
Democratic People's Republic of Korea
Botswana*
Colombia
Taiwan*
Italy*
Saint Vincent and the Grenadines*
Republic of Korea
Suriname*
Guatemala
Lesotho
Kenya
Mauritius
Moldova
Djibouti*
Grenada*
Greece*
Syrian Arab Republic*
Turkmenistan*
Dominica*
Thailand
Saint Lucia*
Dominican Republic*
Brunei Darussalam*
Iraq*
Singapore*
Indonesia*
Kyrgyzstan
Paraguay*
Guyana
Azerbaijan
Algeria*
Egypt
Brazil*
Namibia*
Solomon Islands*
Morocco
Zimbabwe
Uganda
Madagascar
Philippines
Japan
Comoros*
Sudan*
Cyprus*
Zambia
Bahrain*
Oman*
Myanmar*
Fiji
Malawi
Eritrea*
Yemen*
Papua New Guinea*
Uzbekistan
Bolivia
Lao People's Democratic Republic*
Global average
Saudi Arabia*
Maldives
Cape Verde*
Mauritania*
Sierra Leone
Afghanistan*
Nepal
Somalia
Angola
Haiti
São Tomé and Príncipe
Bhutan*
Cameroon
Bangladesh
Mozambique
Democratic Republic of the Congo
Guinea−Bissau*
Central African Republic
Chad*
United Republic of Tanzania
Cambodia
Pakistan
Equatorial Guinea*
Liberia
India
Gambia
Côte d'Ivoire*
Nigeria
Niger
Togo*
Burkina Faso
Congo
Gabon*
Guinea
Mali
Benin
Ghana
Senegal
105
110
115
120
mean(Hb) in 2011
0%
10%
20%
30%
40%
50%
60%
P(Hb)<110 in 2011
0%
0.5%
1%
1.5%
2%
2.5%
P(Hb)<70 in 2011
High Income
Eastern Europe
West and Central Africa
Central Asia, Middle East, and North Africa
South Asia
East and Southeast Asia
Southern and Tropical Latin America
Andean and Central Latin America and Caribbean
Oceania
East Africa
Southern Africa
Global average

**Webfigure 7: Comparison of change in mean haemoglobin of non-pregnant women, with those of pregnant women and children. The lines show the uncertainty interval; as defined in Methods, the uncertainty intervals represent the 2.5th-97.5th percentiles of the 2,500 posterior MCMC draws.**

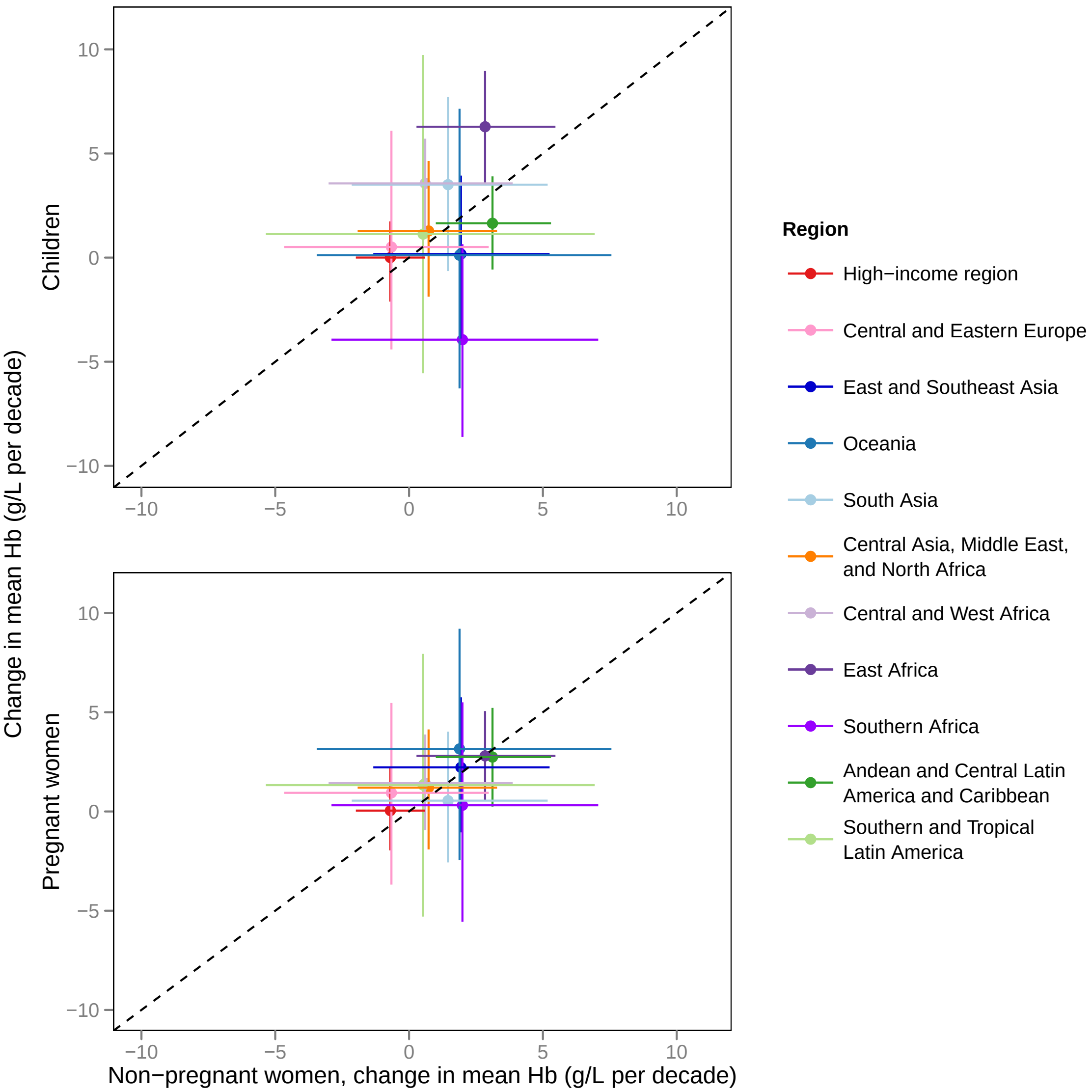

**Webfigure 8: Trends in mean haemoglobin concentration and anaemia prevalence by country between 1990 and 2011. Trends are shown in relation to the original data. The shaded area shows the uncertainty intervals, defined and estimated as described in Methods. Only mean haemoglobin and WHO-defined anaemia and severe anaemia prevalence are shown; data using other definitions of anaemia were used in this analysis, accounting for the specific threshold as described in Methods, but are not shown. The number of observations used but not shown is reported in each panel. Observed anaemia and severe anaemia prevalences for combined groups of pregnant and nonpregnant women are based on cutoffs of 120 g/L and 80 g/L, respectively.**

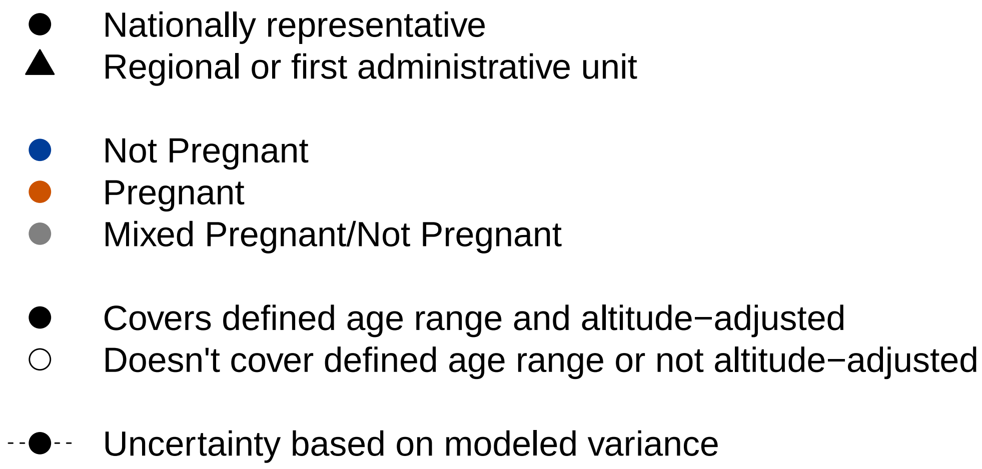

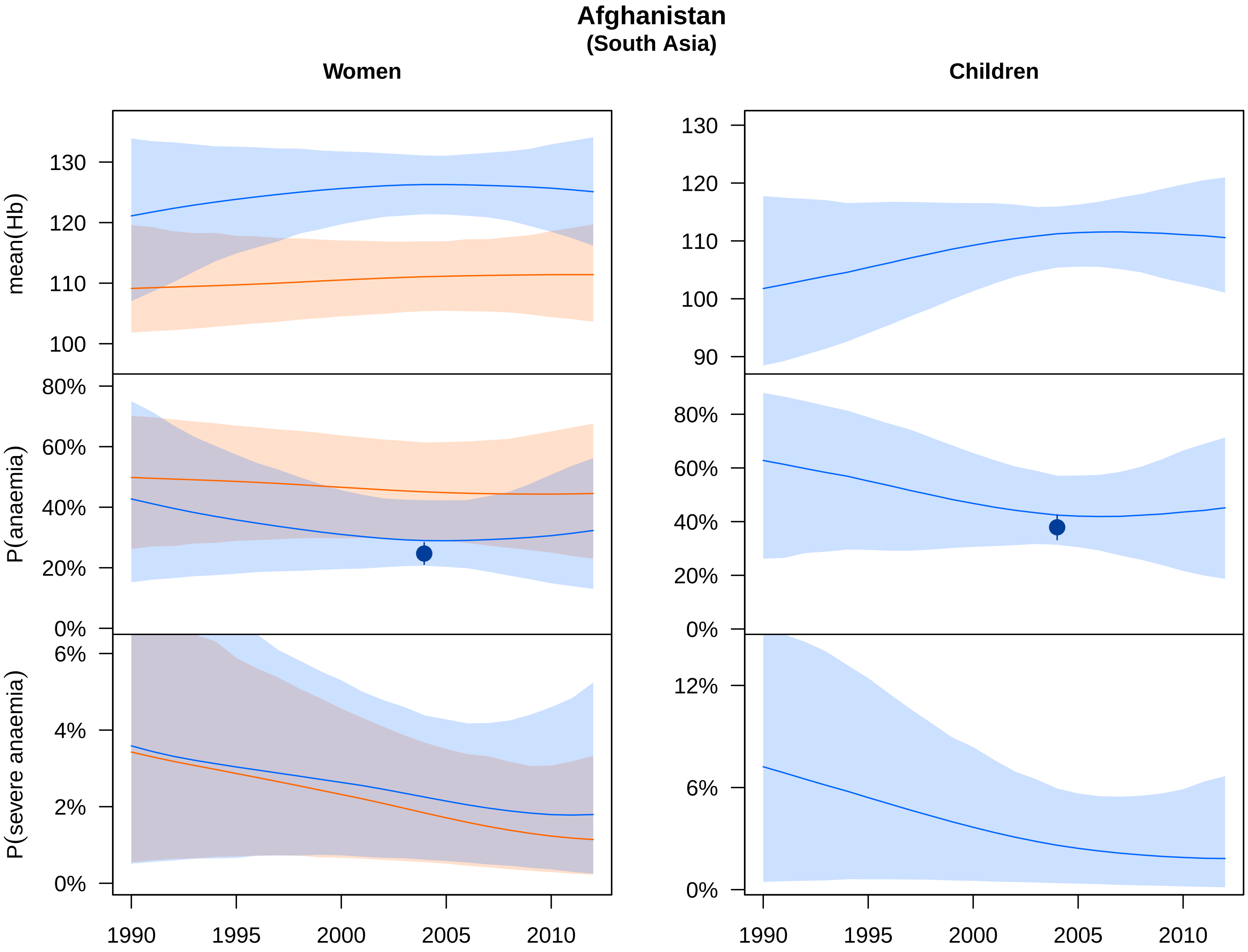
Afghanistan
(South Asia)
Women
Children
mean(Hb)
P(anaemia)
P(severe anaemia)
130
120
110
100
90
80%
60%
40%
20%
0%
6%
4%
2%
12%
1990
1995
2000
2005
2010

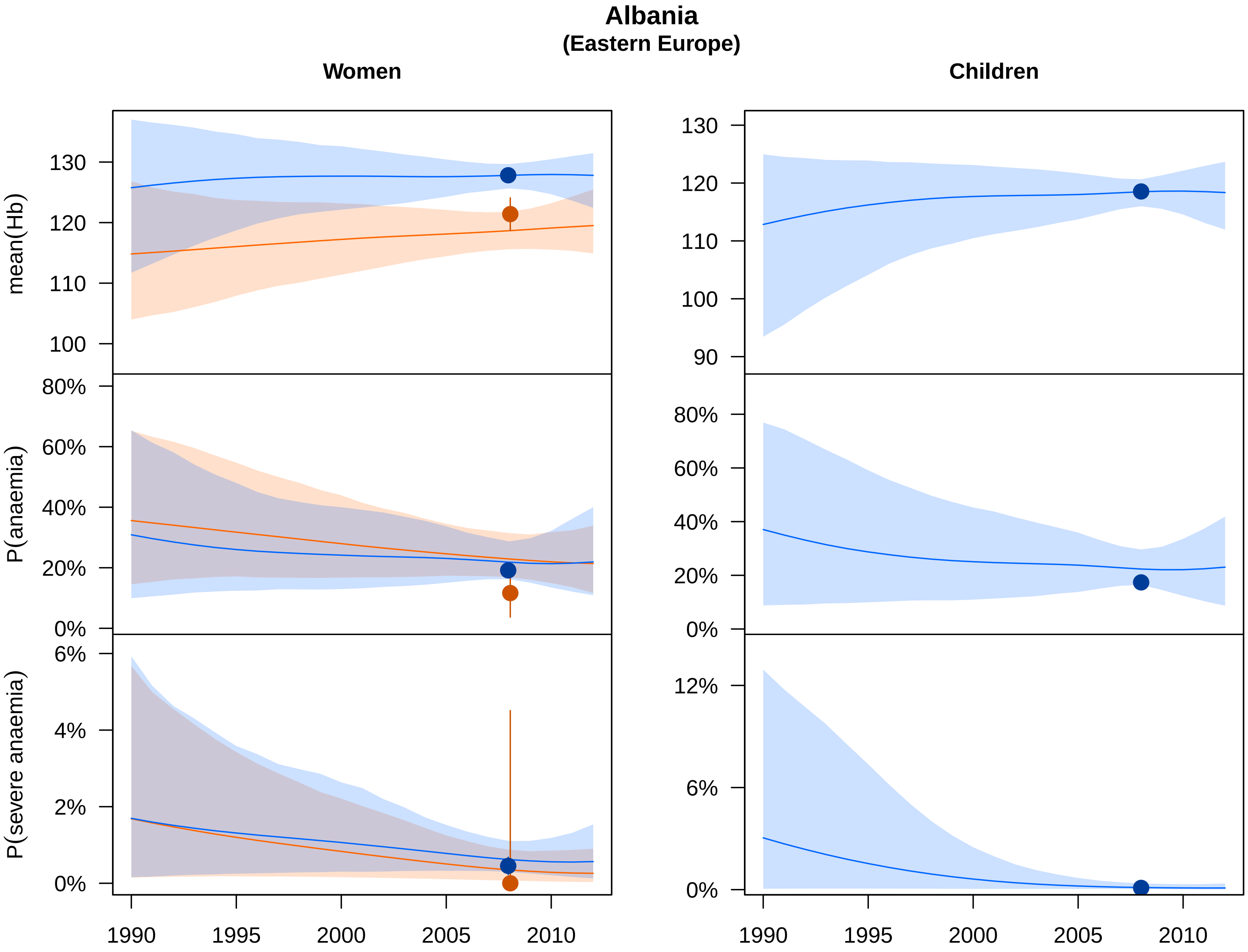

Albania
(Eastern Europe)
Women
Children
mean(Hb)
P(anaemia)
P(severe anaemia)
130
120
110
100
90
80%
60%
40%
20%
0%
6%
4%
2%
12%
1990
1995
2000
2005
2010

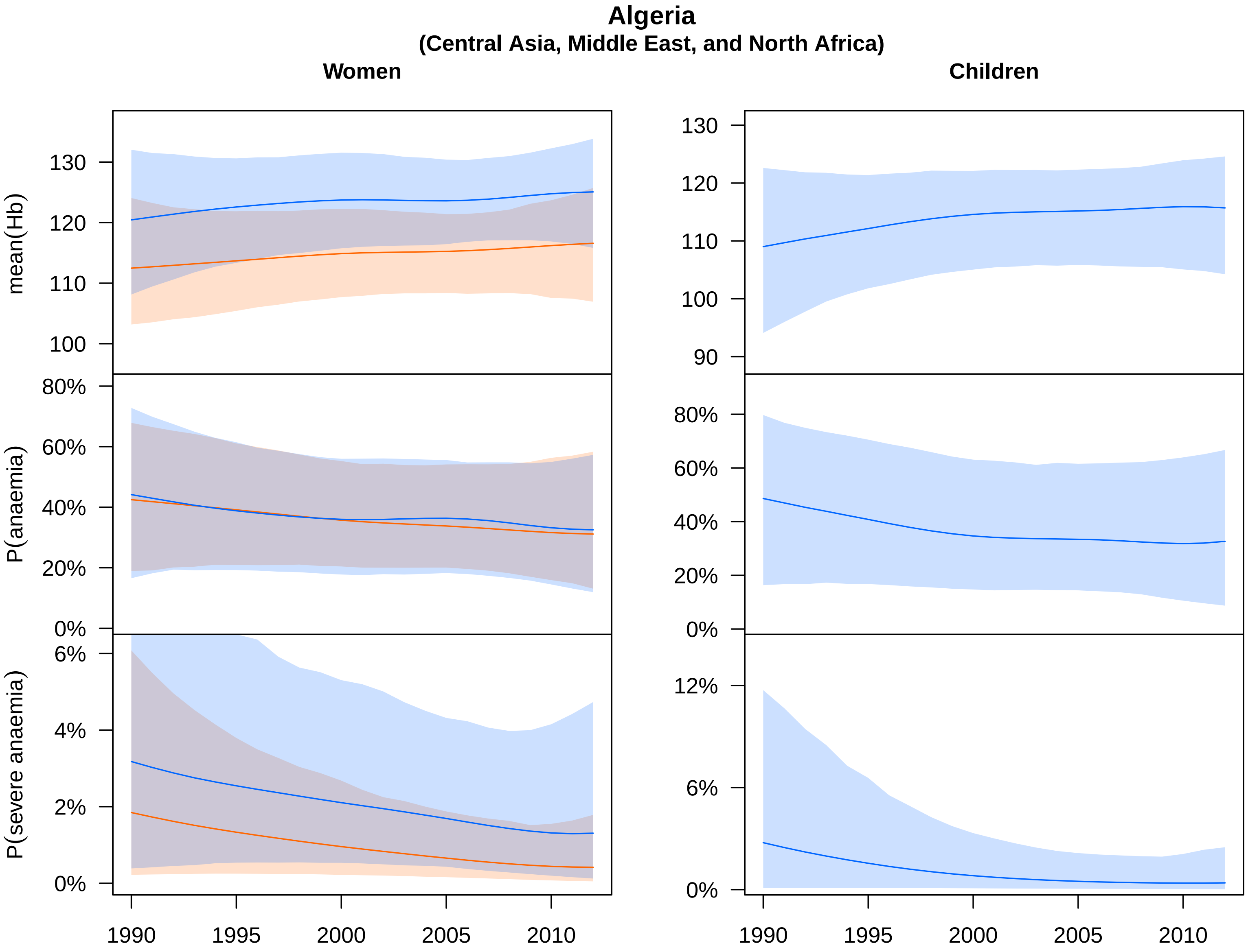

Algeria
(Central Asia, Middle East, and North Africa)
Women
Children
mean(Hb)
P(anaemia)
P(severe anaemia)
130
120
110
100
90
80%
60%
40%
20%
0%
6%
4%
2%
12%
1990
1995
2000
2005
2010

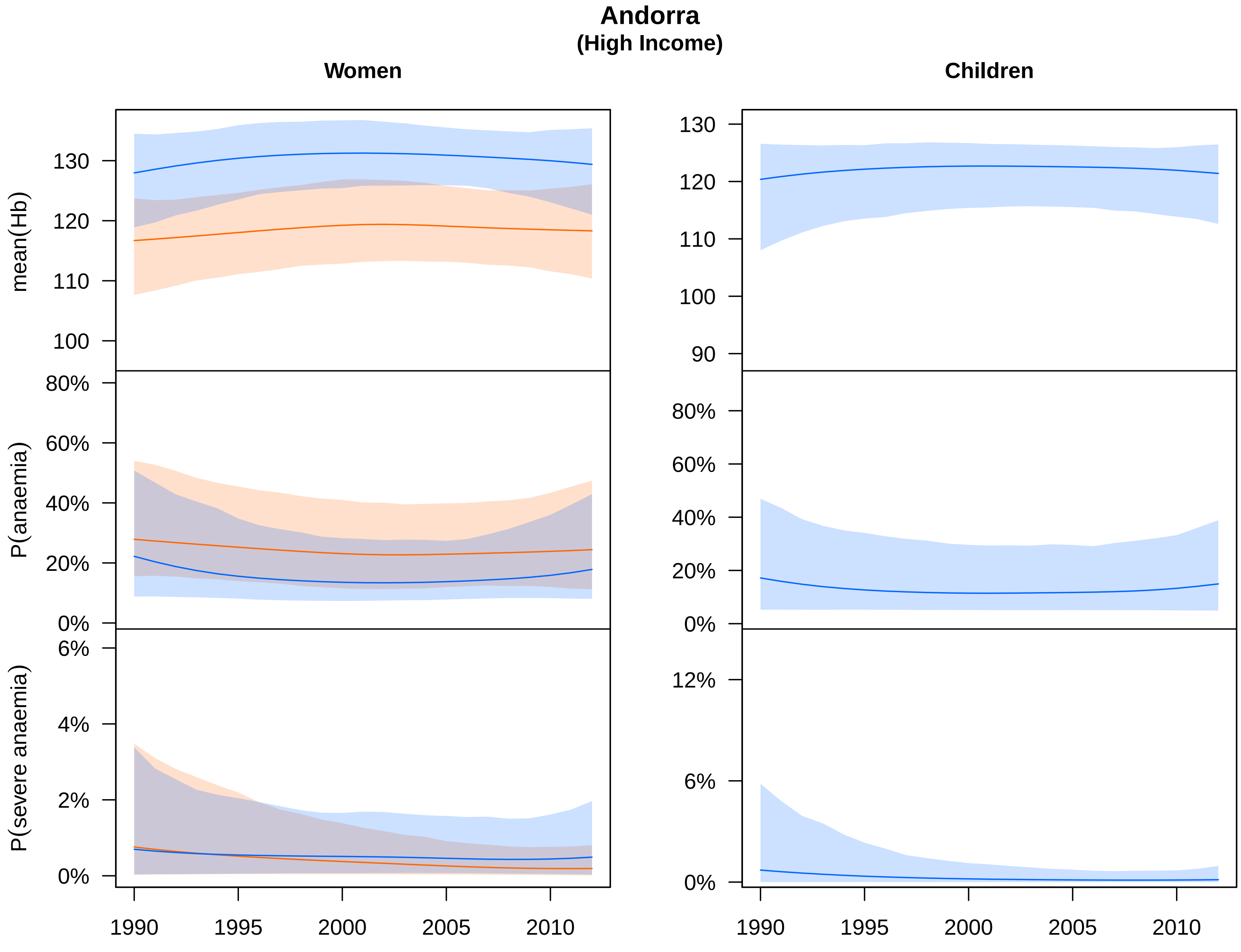

Andorra
(High Income)
Women
Children
mean(Hb)
P(anaemia)
P(severe anaemia)
130
120
110
100
90
80%
60%
40%
20%
0%
6%
4%
2%
12%
1990
1995
2000
2005
2010

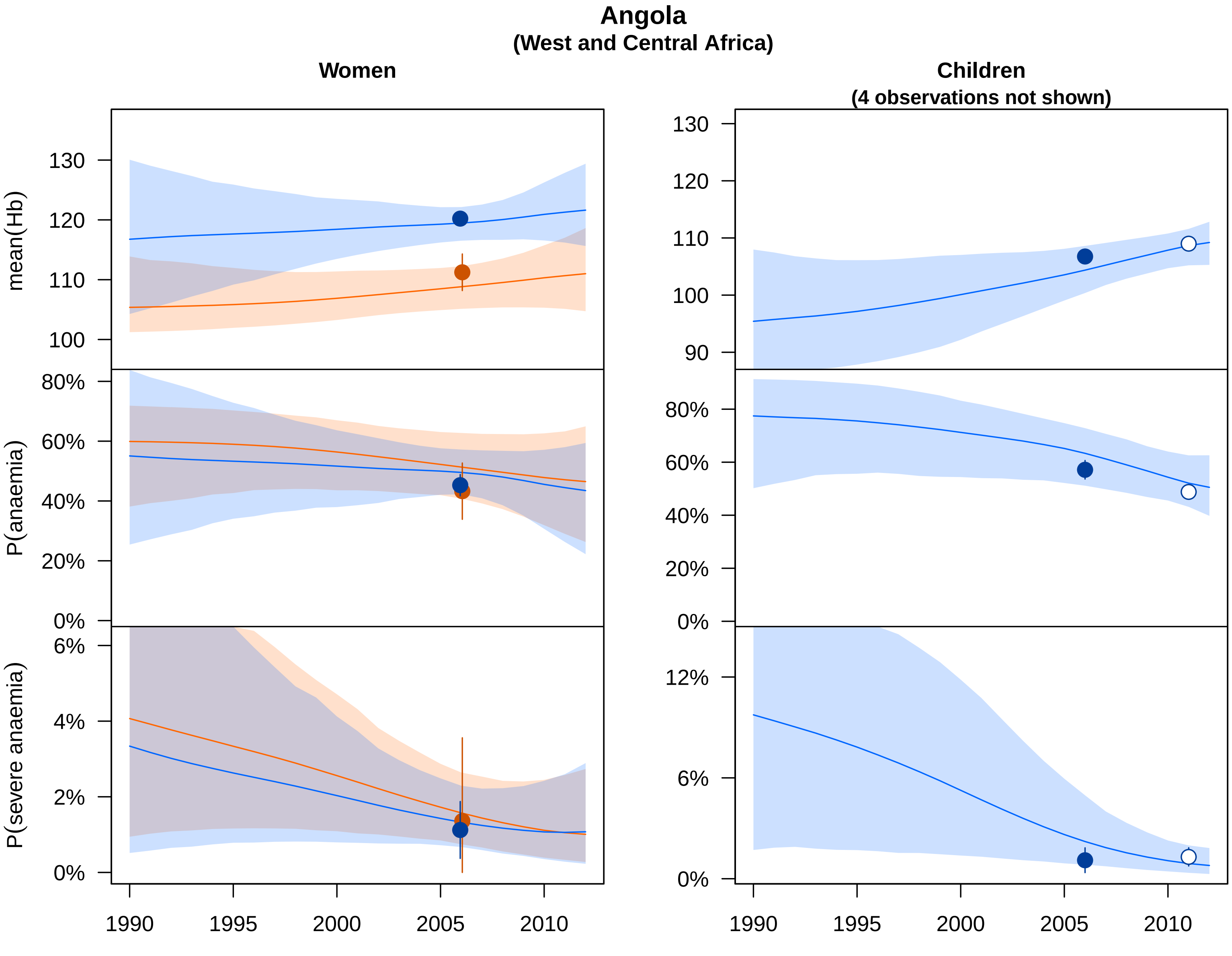

Angola
(West and Central Africa)
Women
Children
(4 observations not shown)
mean(Hb)
P(anaemia)
P(severe anaemia)
130
120
110
100
90
80%
60%
40%
20%
0%
6%
4%
2%
12%
1990
1995
2000
2005
2010

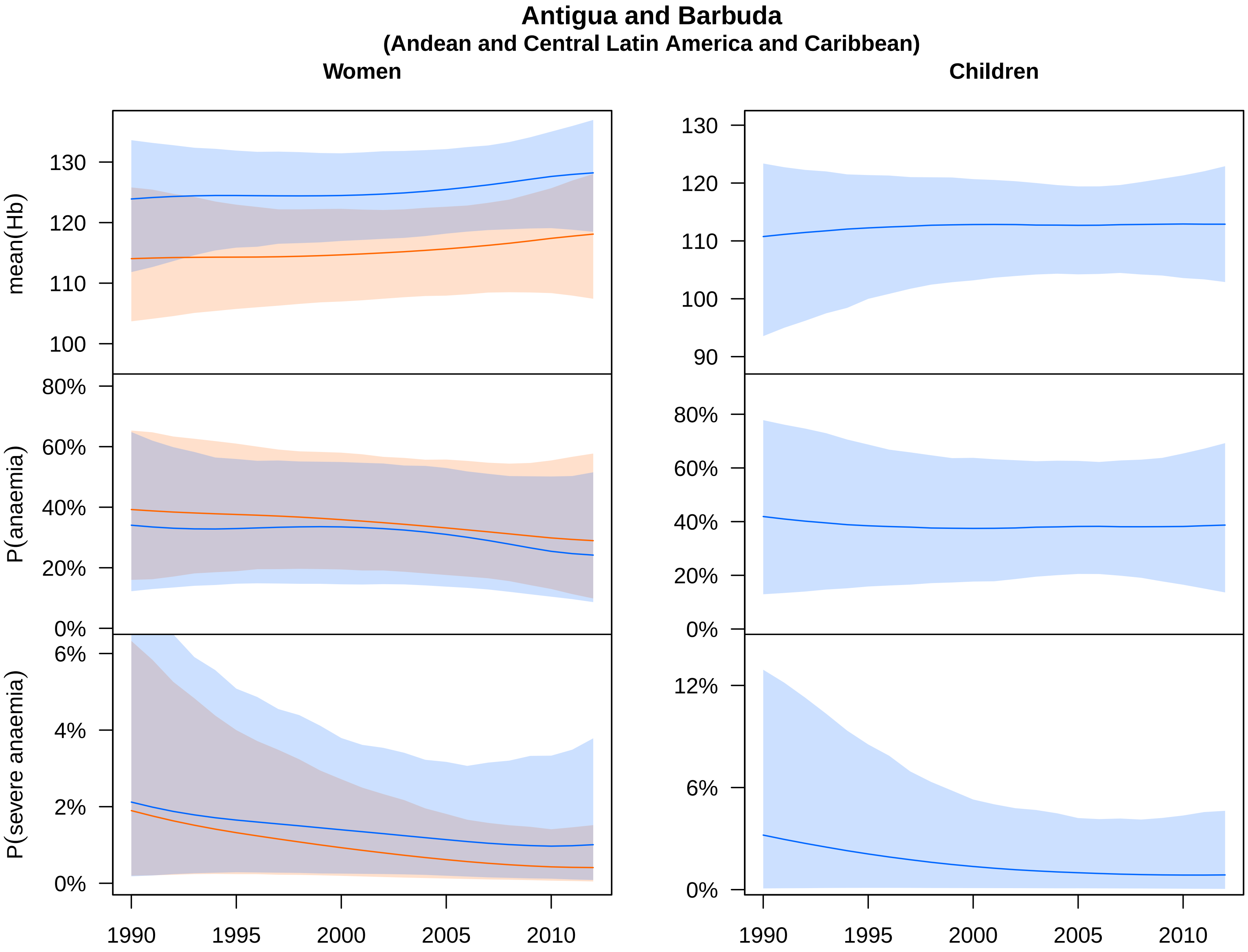
Antigua and Barbuda
(Andean and Central Latin America and Caribbean)
Women
Children
mean(Hb)
P(anaemia)
P(severe anaemia)
100
110
120
130
90
0%
20%
40%
60%
80%
2%
4%
6%
12%
1990
1995
2000
2005
2010

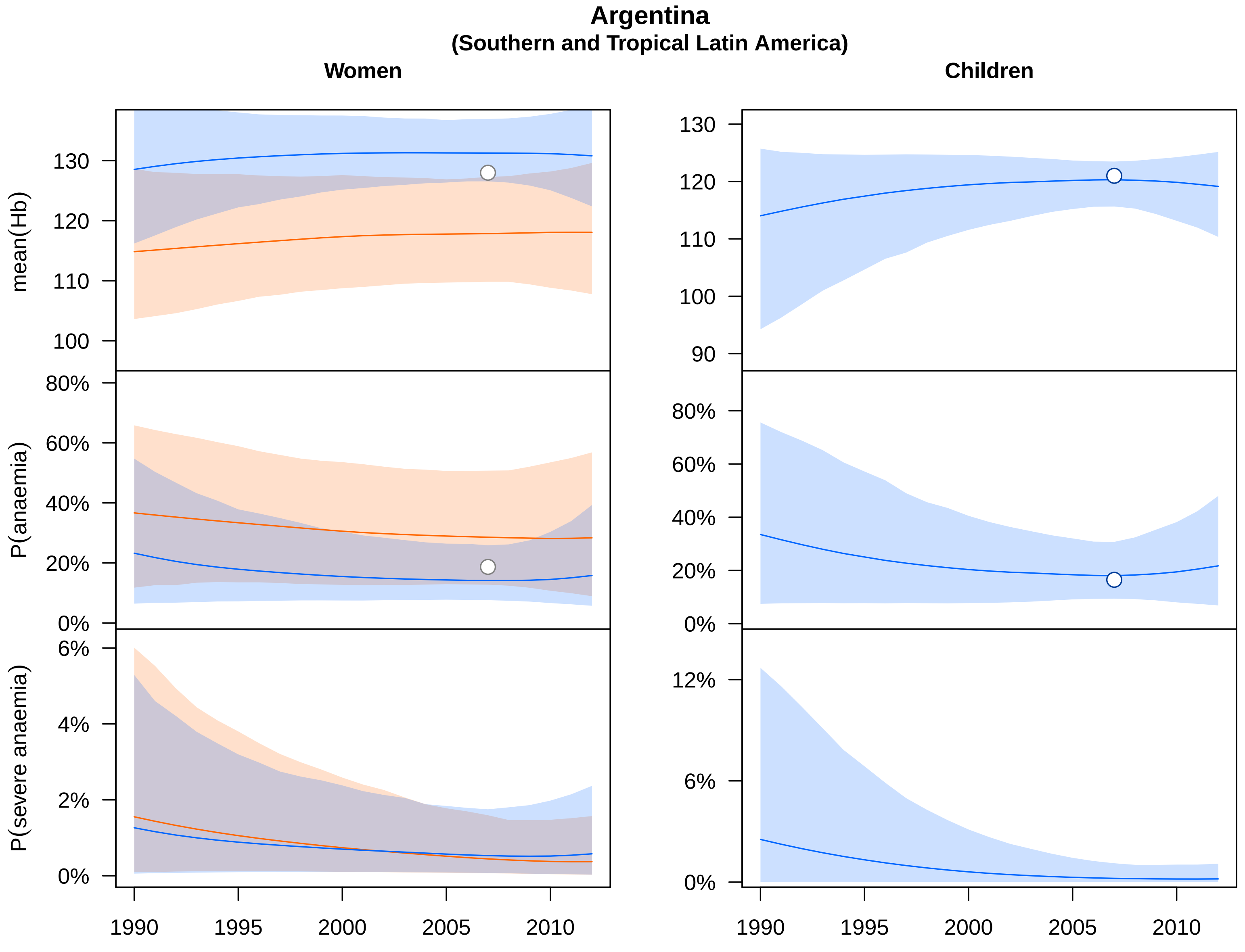

Argentina
(Southern and Tropical Latin America)
Women
Children
mean(Hb)
P(anaemia)
P(severe anaemia)
130
120
110
100
90
80%
60%
40%
20%
0%
6%
4%
2%
12%
1990
1995
2000
2005
2010

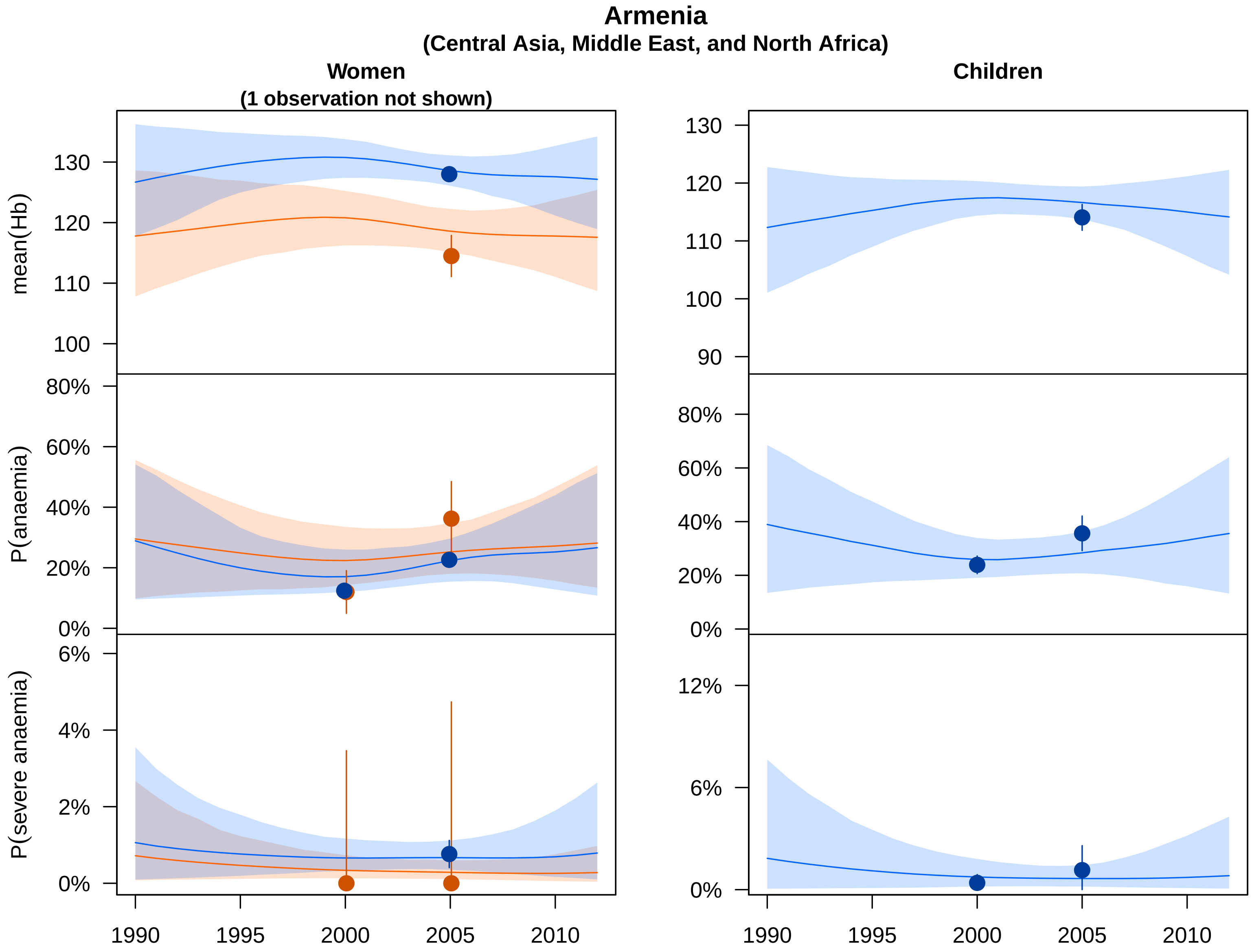
Armenia
(Central Asia, Middle East, and North Africa)
Women
(1 observation not shown)
Children
mean(Hb)
P(anaemia)
P(severe anaemia)
130
120
110
100
90
80%
60%
40%
20%
0%
6%
4%
2%
12%
1990
1995
2000
2005
2010

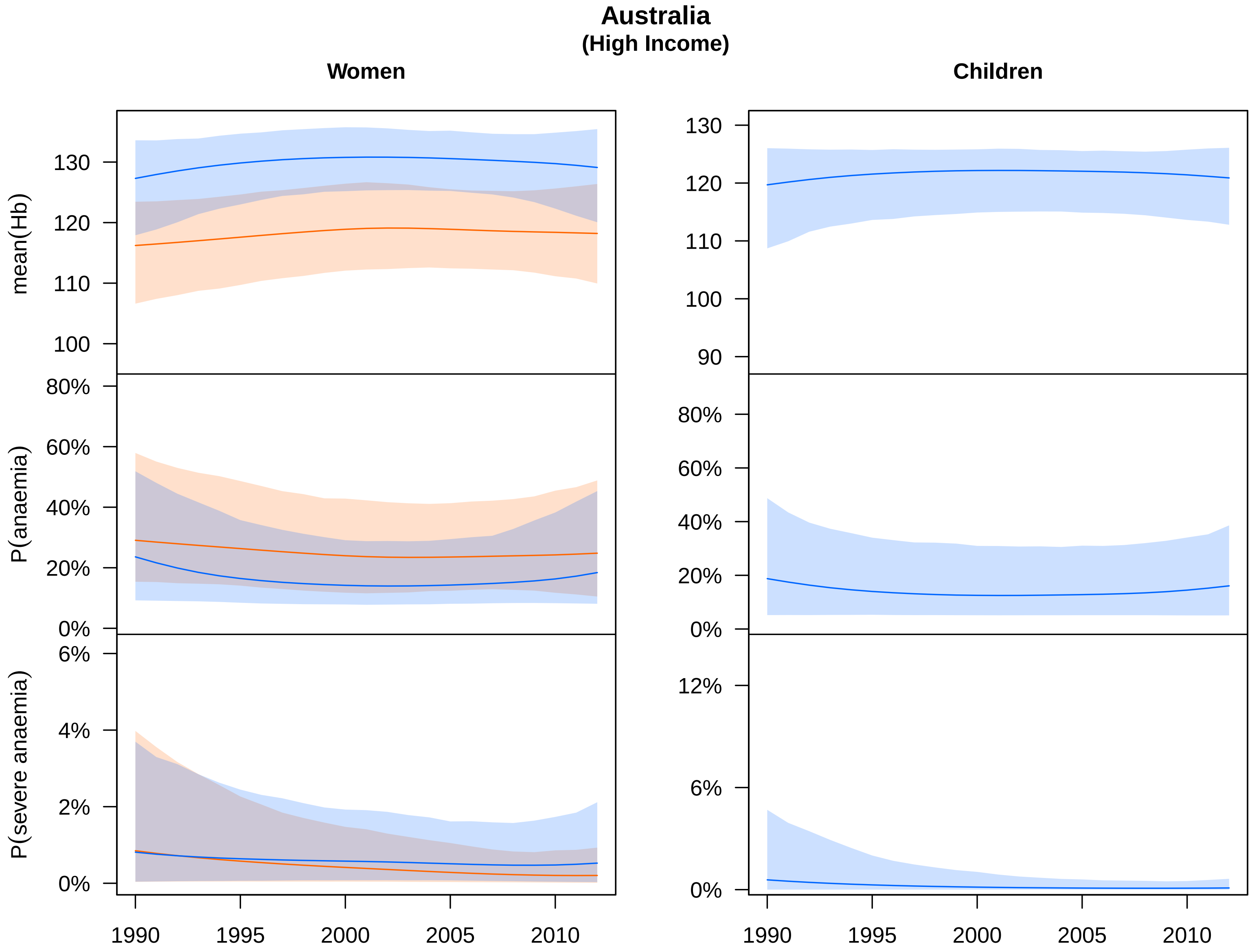
Australia
(High Income)
Women
Children
mean(Hb)
P(anaemia)
P(severe anaemia)
130
120
110
100
90
80%
60%
40%
20%
0%
6%
4%
2%
12%
1990
1995
2000
2005
2010

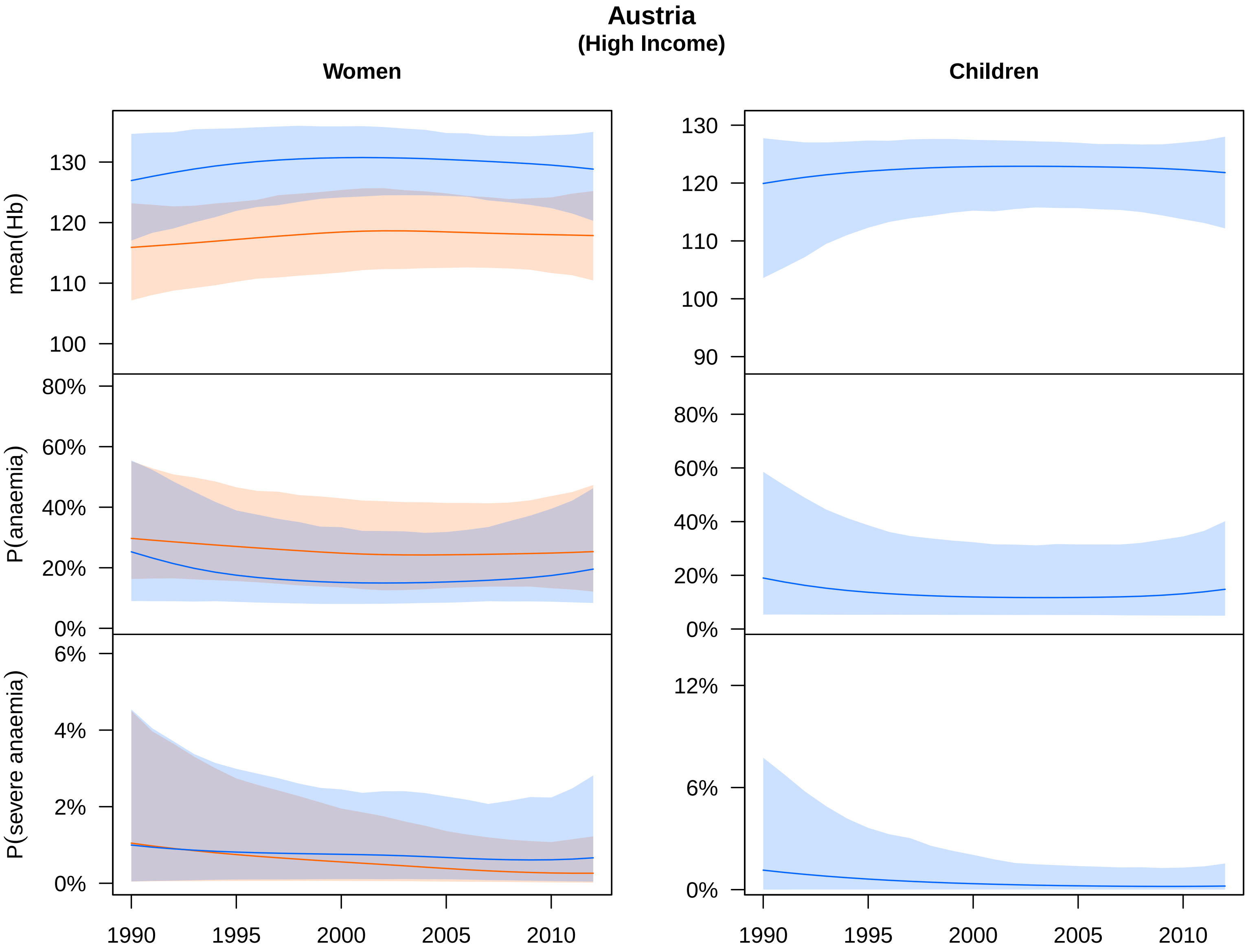

Austria
(High Income)
Women
Children
mean(Hb)
P(anaemia)
P(severe anaemia)
130
120
110
100
90
80%
60%
40%
20%
0%
6%
4%
2%
12%
1990
1995
2000
2005
2010

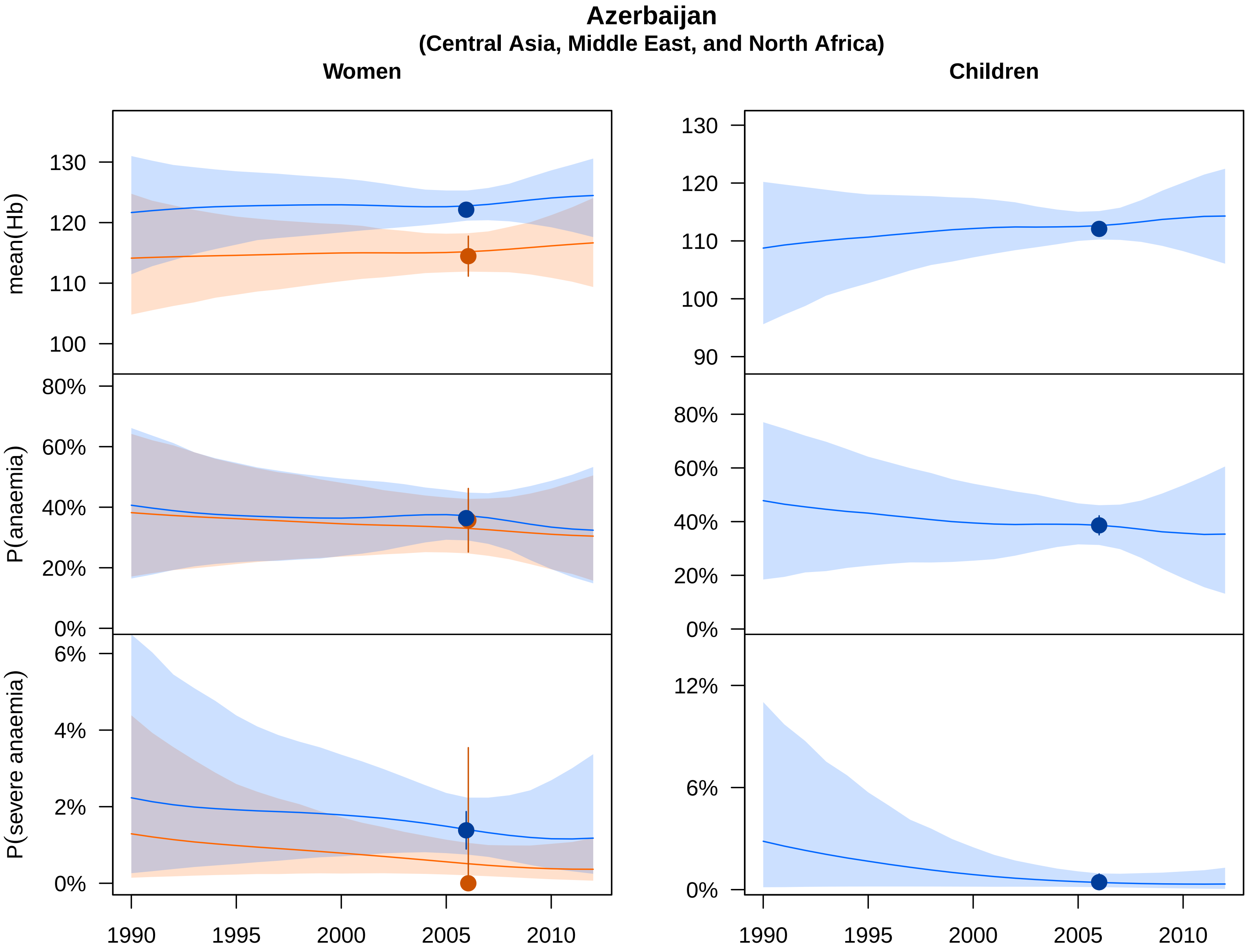
Azerbaijan
(Central Asia, Middle East, and North Africa)
Women
Children
mean(Hb)
P(anaemia)
P(severe anaemia)
130
120
110
100
90
80%
60%
40%
20%
0%
6%
4%
2%
12%
1990
1995
2000
2005
2010

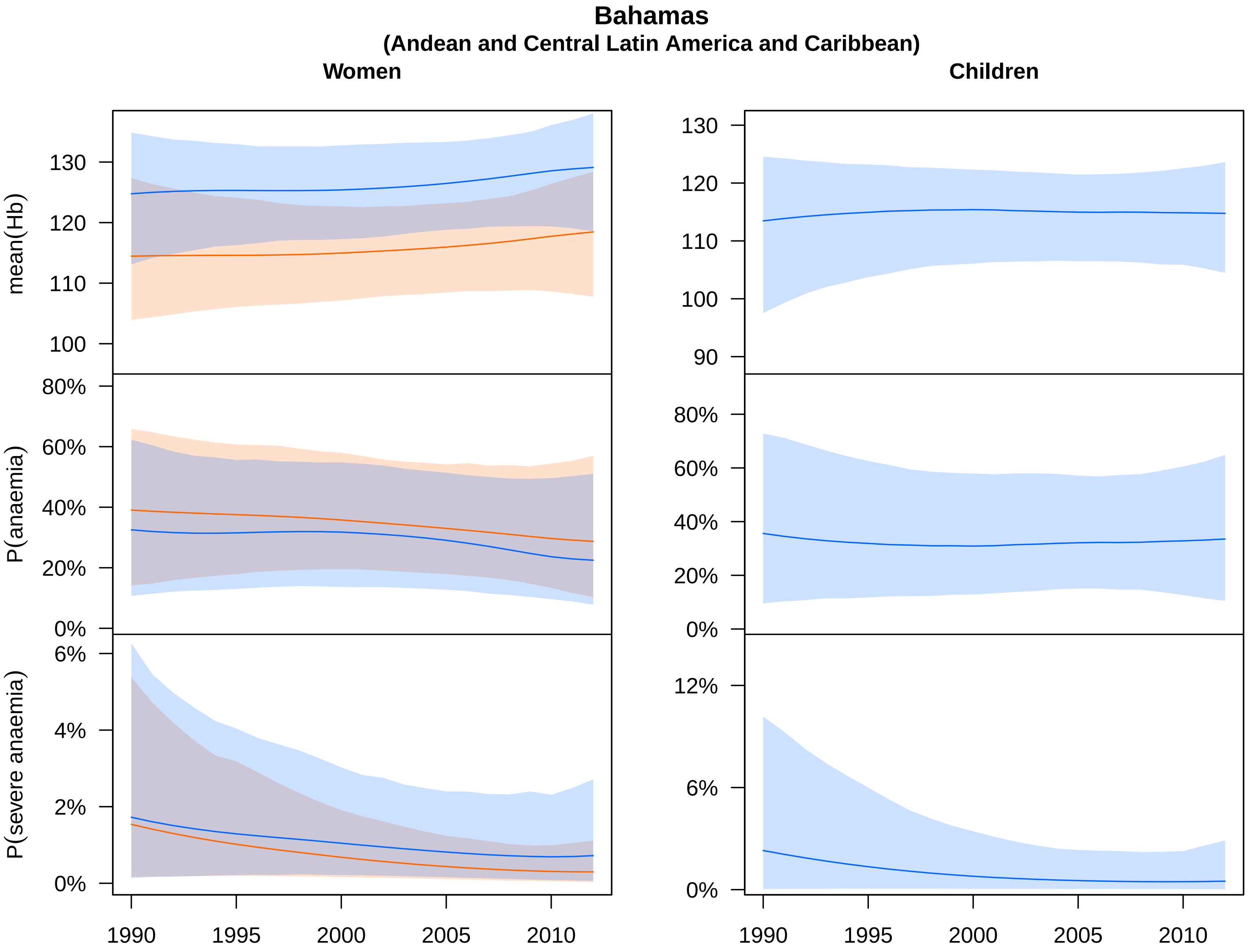

Bahamas
(Andean and Central Latin America and Caribbean)
Women
Children
mean(Hb)
P(anaemia)
P(severe anaemia)
100
110
120
130
90
0%
20%
40%
60%
80%
2%
4%
6%
12%
1990
1995
2000
2005
2010

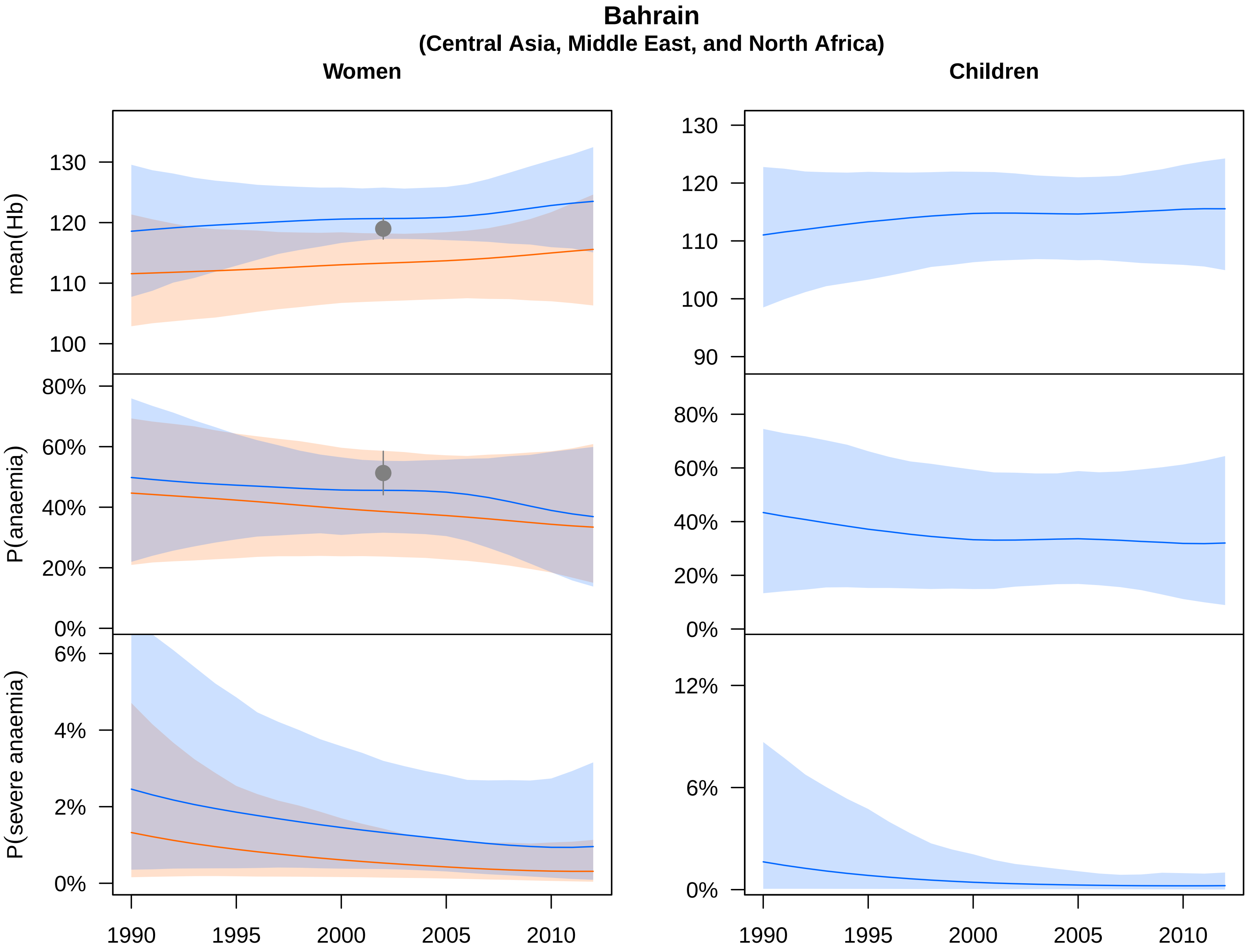
Bahrain
(Central Asia, Middle East, and North Africa)
Women
Children
mean(Hb)
P(anaemia)
P(severe anaemia)
130
120
110
100
90
80%
60%
40%
20%
0%
6%
4%
2%
12%
1990
1995
2000
2005
2010

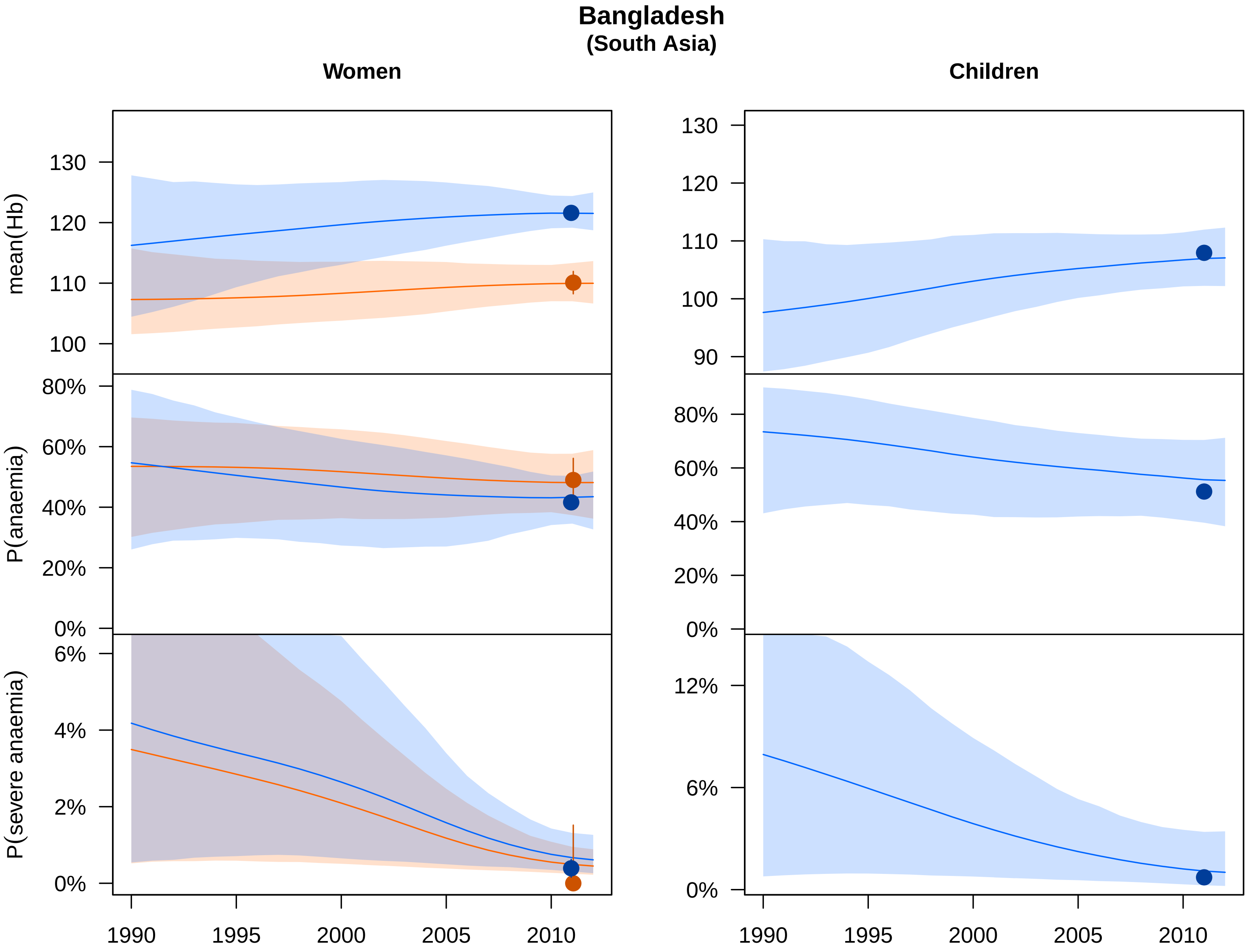
Bangladesh
(South Asia)
Women
Children
mean(Hb)
P(anaemia)
P(severe anaemia)
130
120
110
100
90
80%
60%
40%
20%
0%
6%
4%
2%
12%
1990
1995
2000
2005
2010

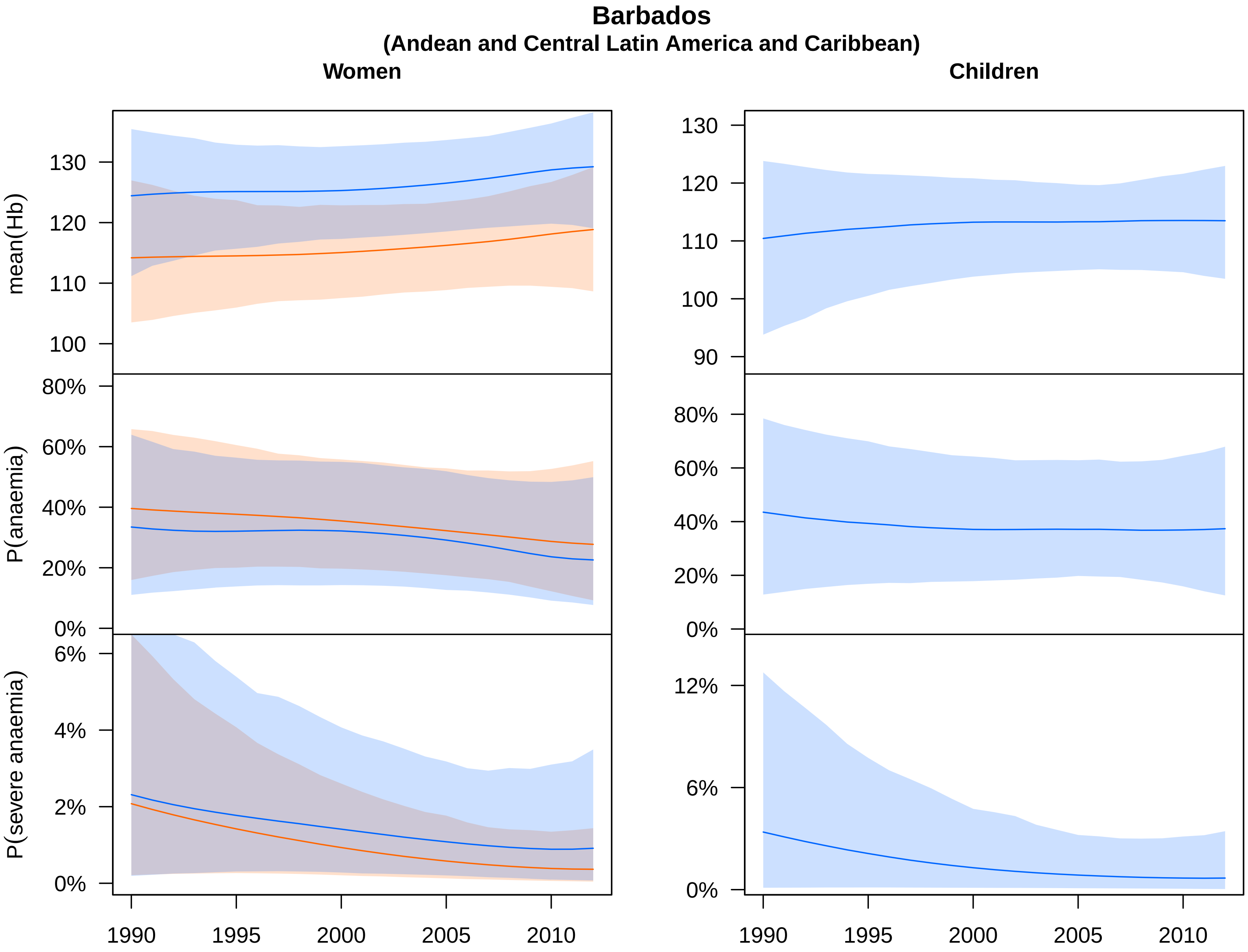
Barbados
(Andean and Central Latin America and Caribbean)
Women
Children
mean(Hb)
P(anaemia)
P(severe anaemia)
100
110
120
130
90
100
110
120
130
0%
20%
40%
60%
80%
0%
20%
40%
60%
80%
0%
2%
4%
6%
0%
6%
12%
1990
1995
2000
2005
2010
1990
1995
2000
2005
2010

**Belarus**
**(Eastern Europe)**

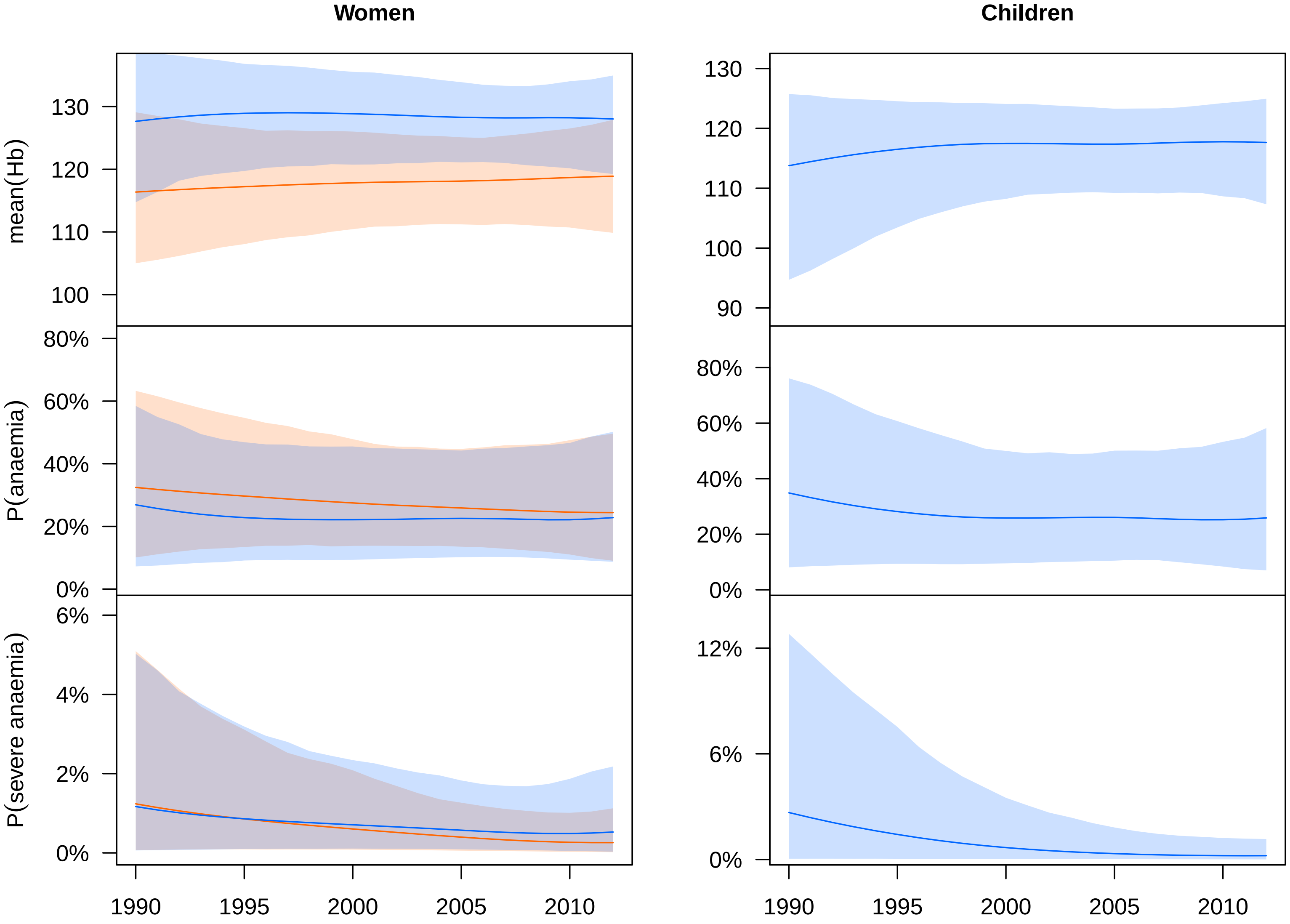

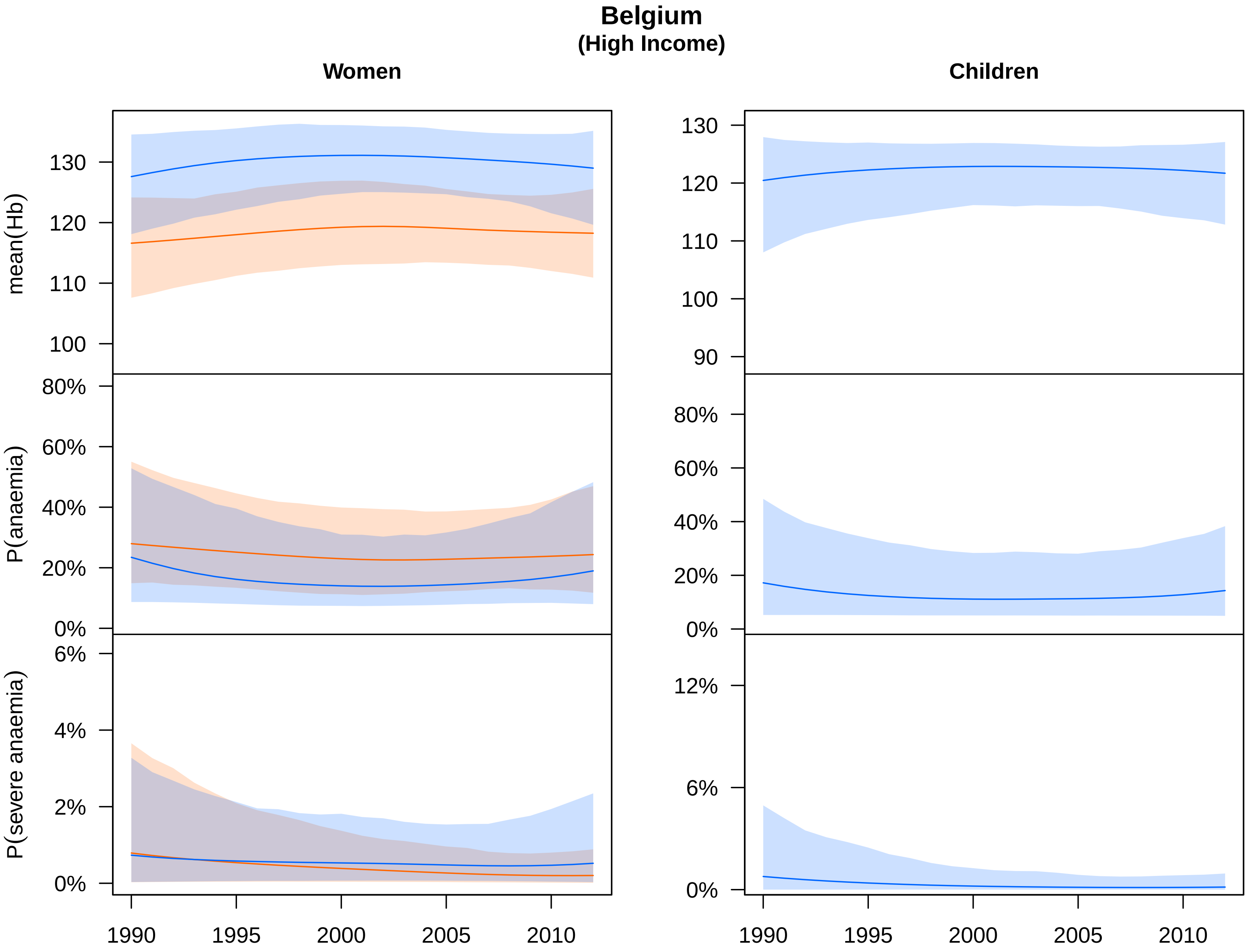

Belgium
(High Income)
Women
Children
mean(Hb)
P(anaemia)
P(severe anaemia)
130
120
110
100
90
80%
60%
40%
20%
0%
6%
4%
2%
12%
1990
1995
2000
2005
2010

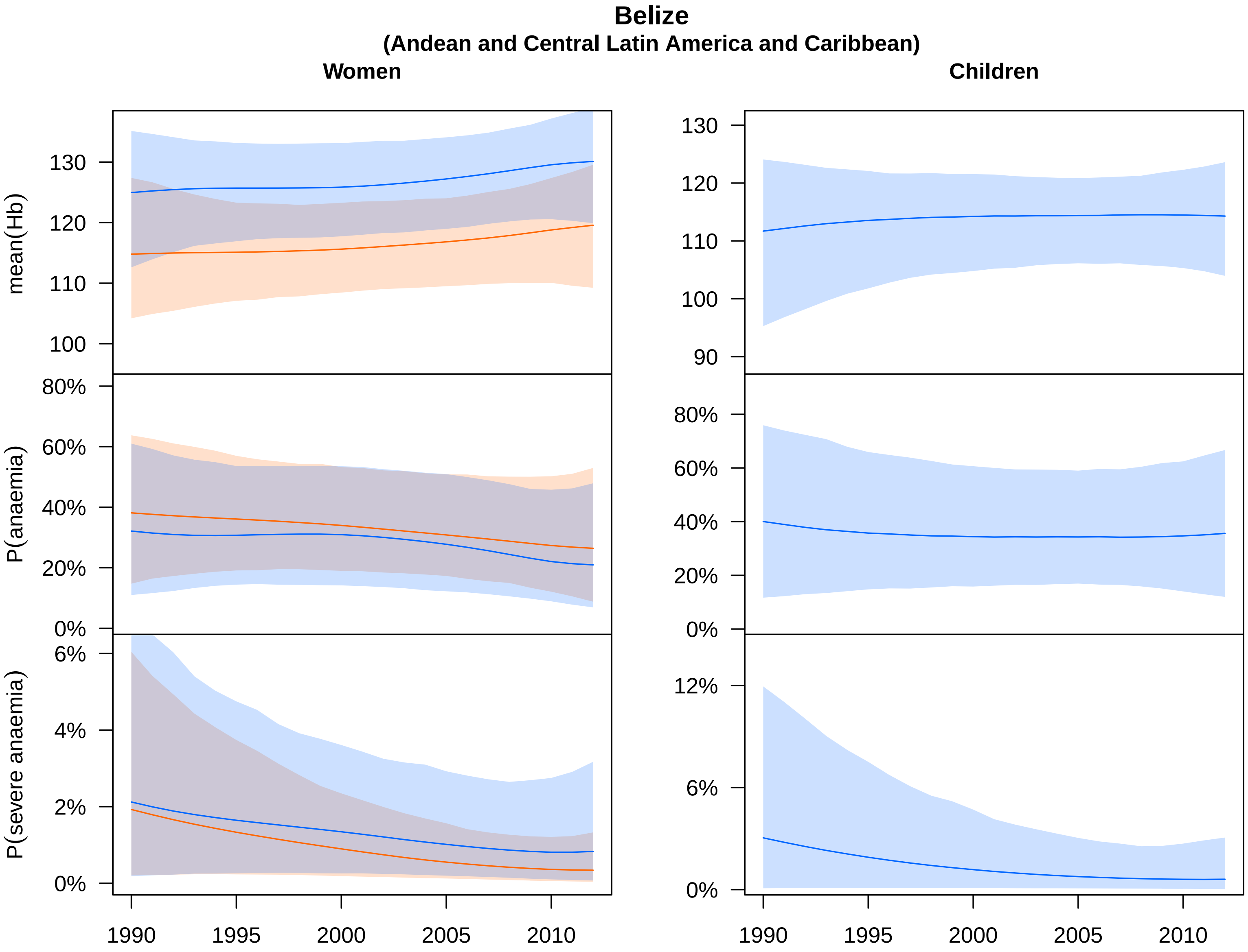
Belize
(Andean and Central Latin America and Caribbean)
Women
Children
mean(Hb)
P(anaemia)
P(severe anaemia)
130
120
110
100
90
80%
60%
40%
20%
0%
6%
4%
2%
12%
1990
1995
2000
2005
2010

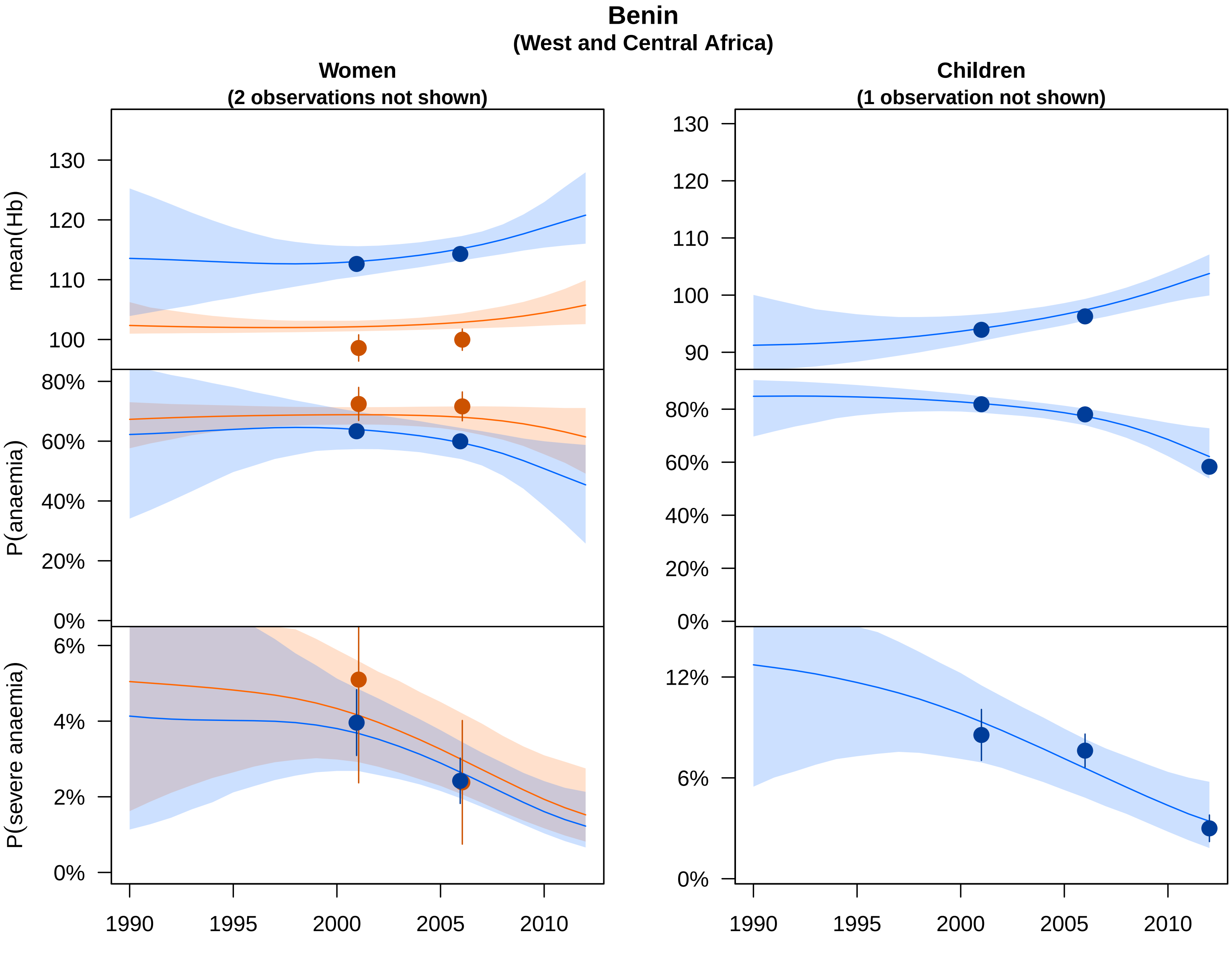
Benin
(West and Central Africa)
Women
(2 observations not shown)
Children
(1 observation not shown)
mean(Hb)
P(anaemia)
P(severe anaemia)
130
120
110
100
90
80%
60%
40%
20%
0%
6%
4%
2%
12%
1990
1995
2000
2005
2010

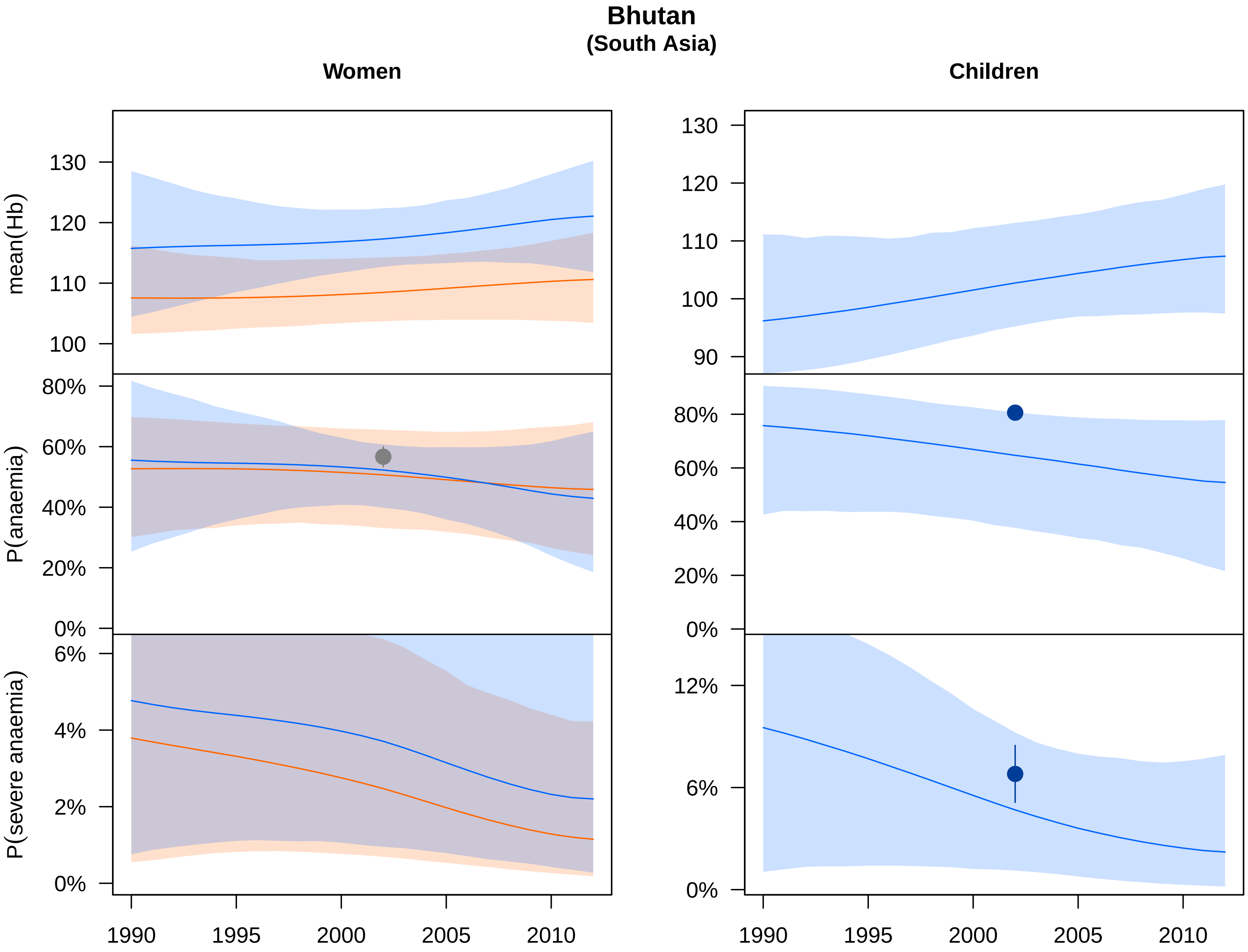
Bhutan
(South Asia)
Women
Children
mean(Hb)
P(anaemia)
P(severe anaemia)
130
120
110
100
90
80%
60%
40%
20%
0%
6%
4%
2%
12%
1990
1995
2000
2005
2010

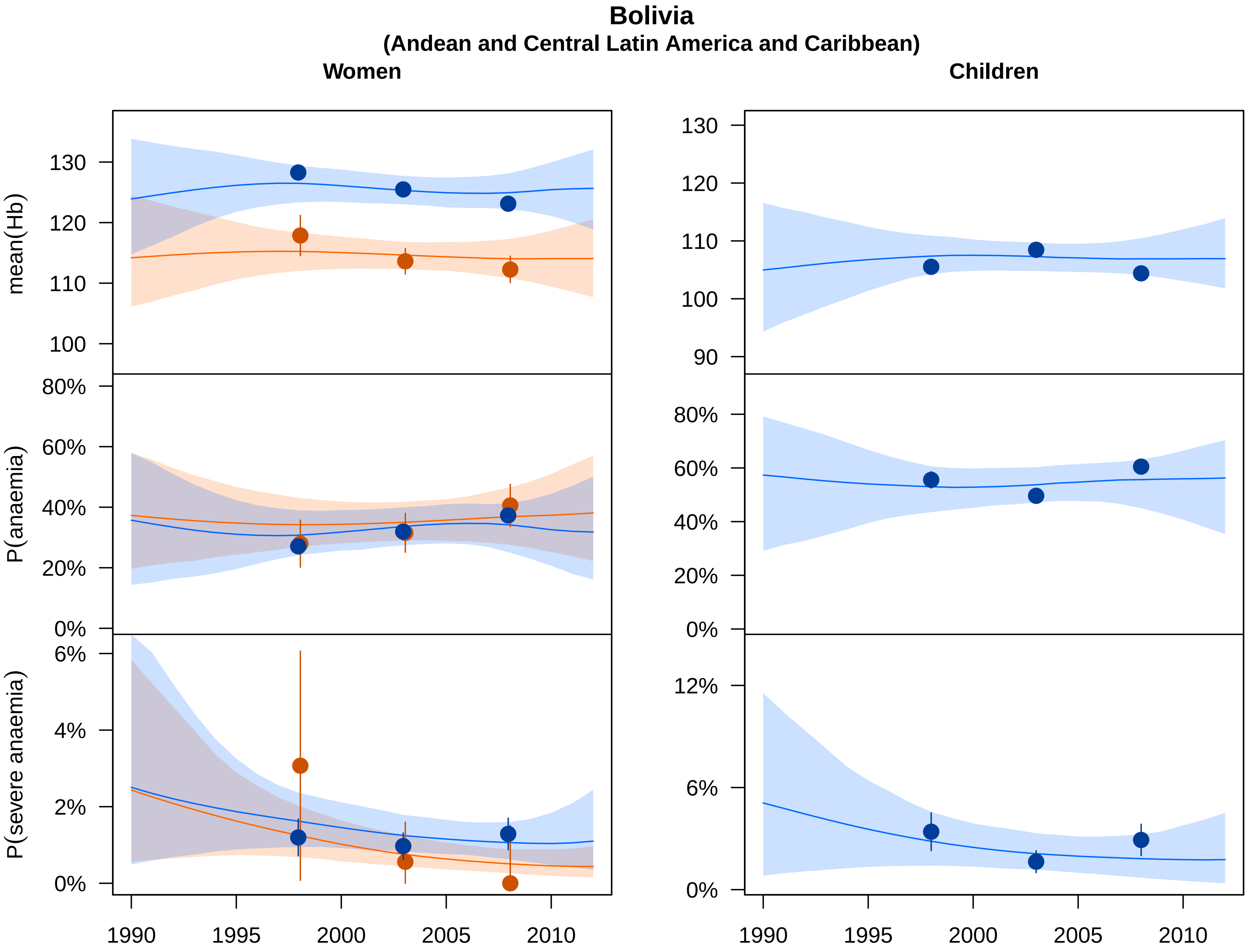
Bolivia
(Andean and Central Latin America and Caribbean)
Women
Children
mean(Hb)
P(anaemia)
P(severe anaemia)
130
120
110
100
90
80%
60%
40%
20%
0%
6%
4%
2%
12%
1990
1995
2000
2005
2010

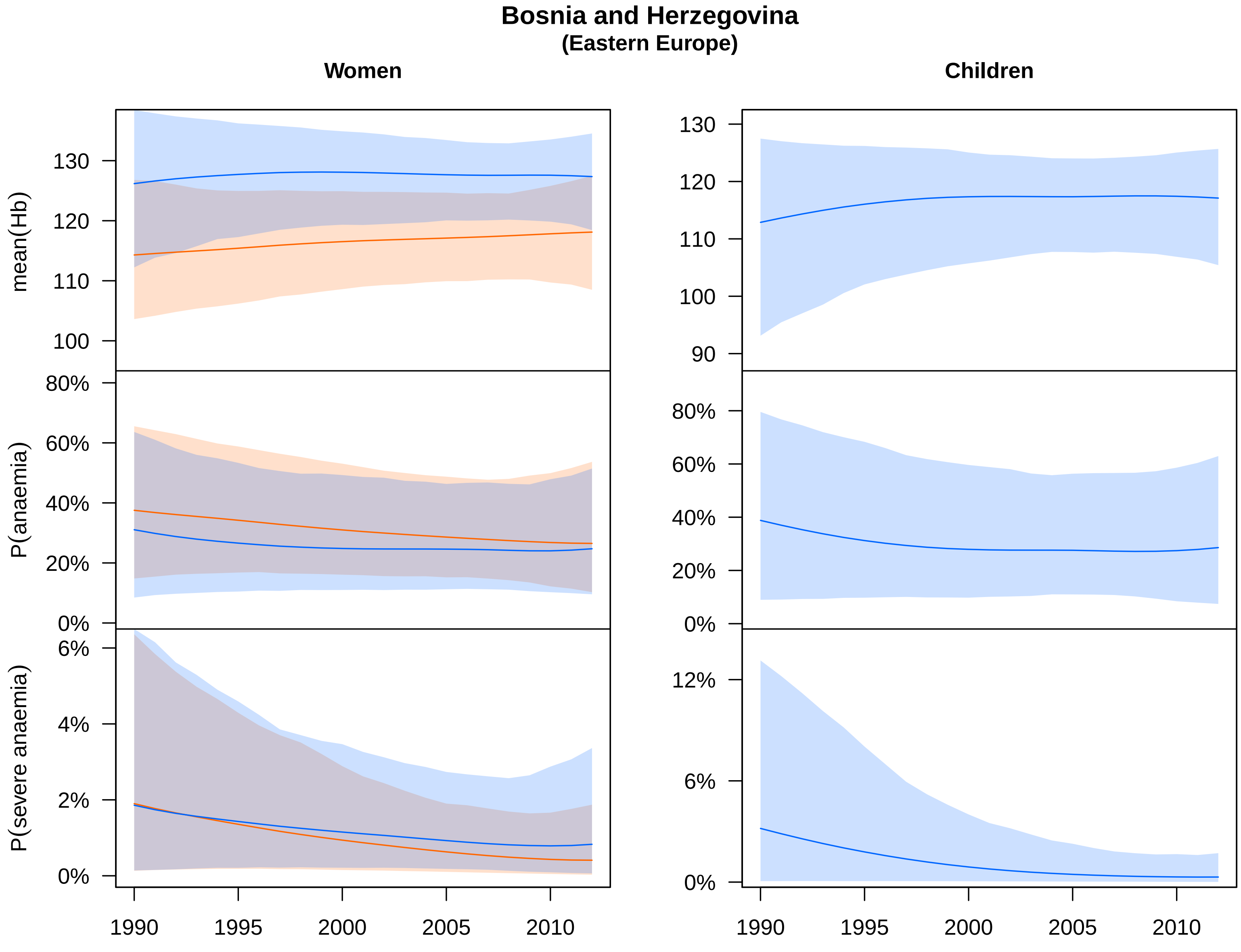
Bosnia and Herzegovina
(Eastern Europe)
Women
Children
mean(Hb)
P(anaemia)
P(severe anaemia)
130
120
110
100
90
80%
60%
40%
20%
0%
6%
4%
2%
12%
1990
1995
2000
2005
2010

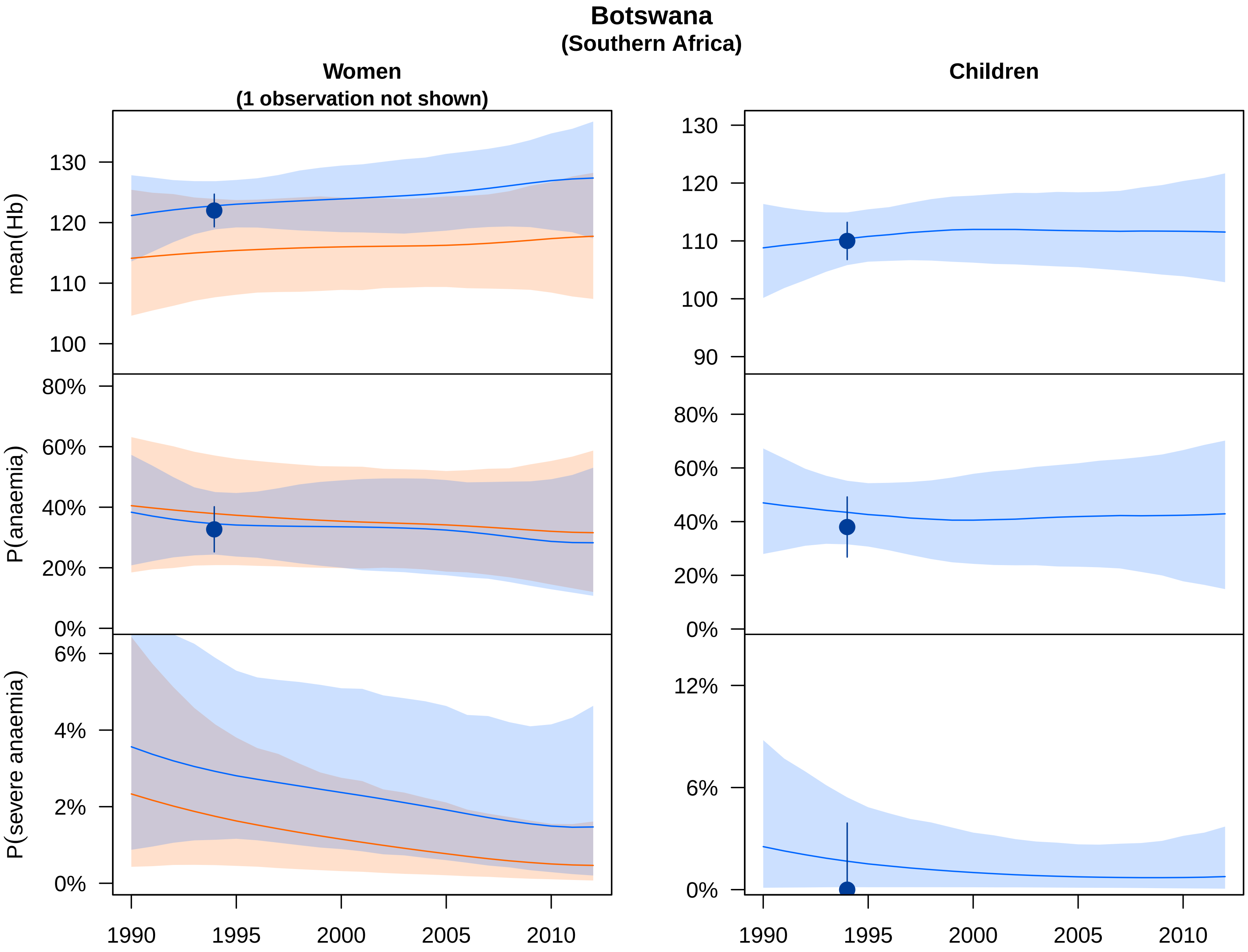
Botswana
(Southern Africa)
Women
(1 observation not shown)
Children
mean(Hb)
P(anaemia)
P(severe anaemia)
130
120
110
100
90
80%
60%
40%
20%
0%
6%
4%
2%
12%
1990
1995
2000
2005
2010

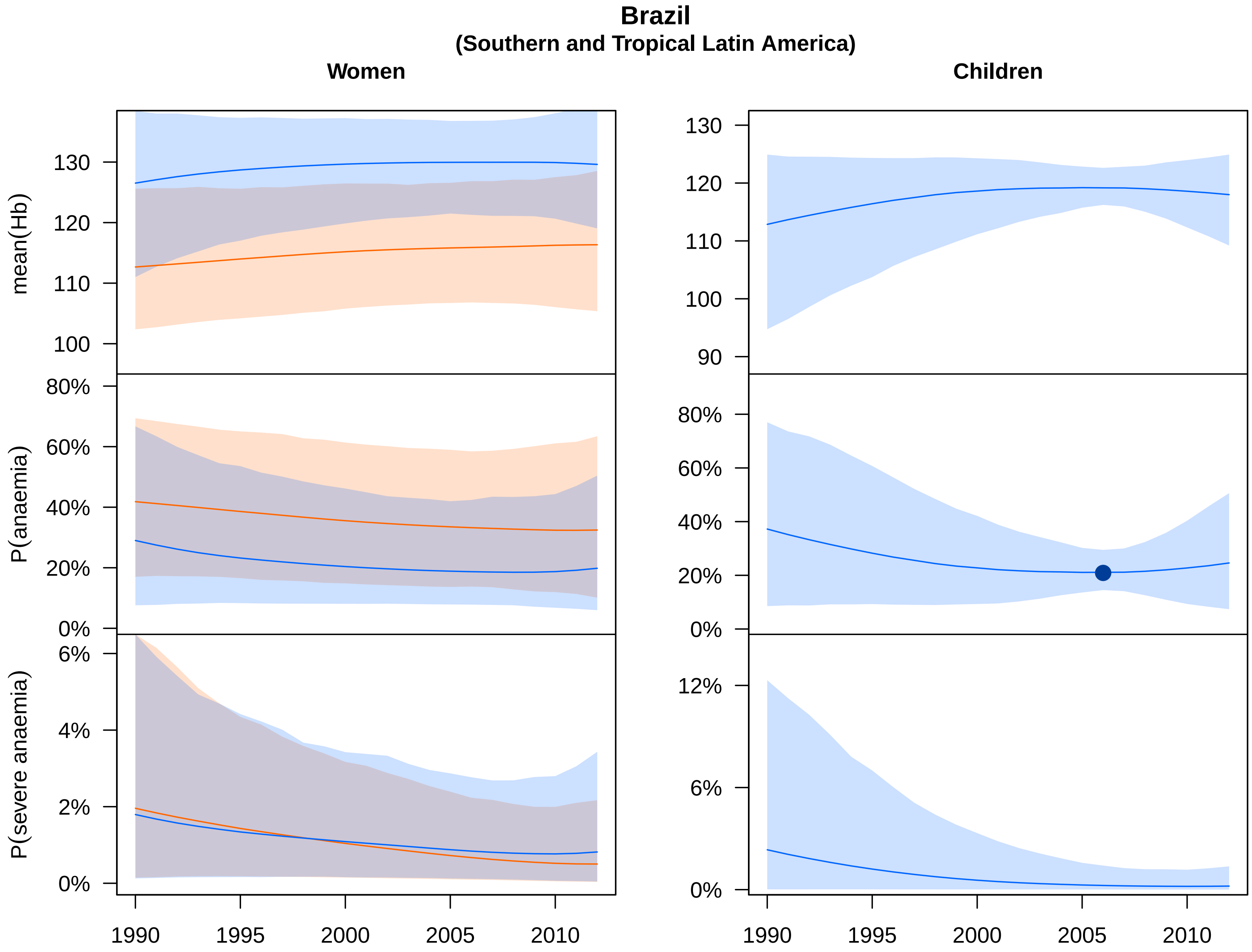
Brazil
(Southern and Tropical Latin America)
Women
Children
mean(Hb)
P(anaemia)
P(severe anaemia)
130
120
110
100
90
80%
60%
40%
20%
0%
6%
4%
2%
12%
1990
1995
2000
2005
2010

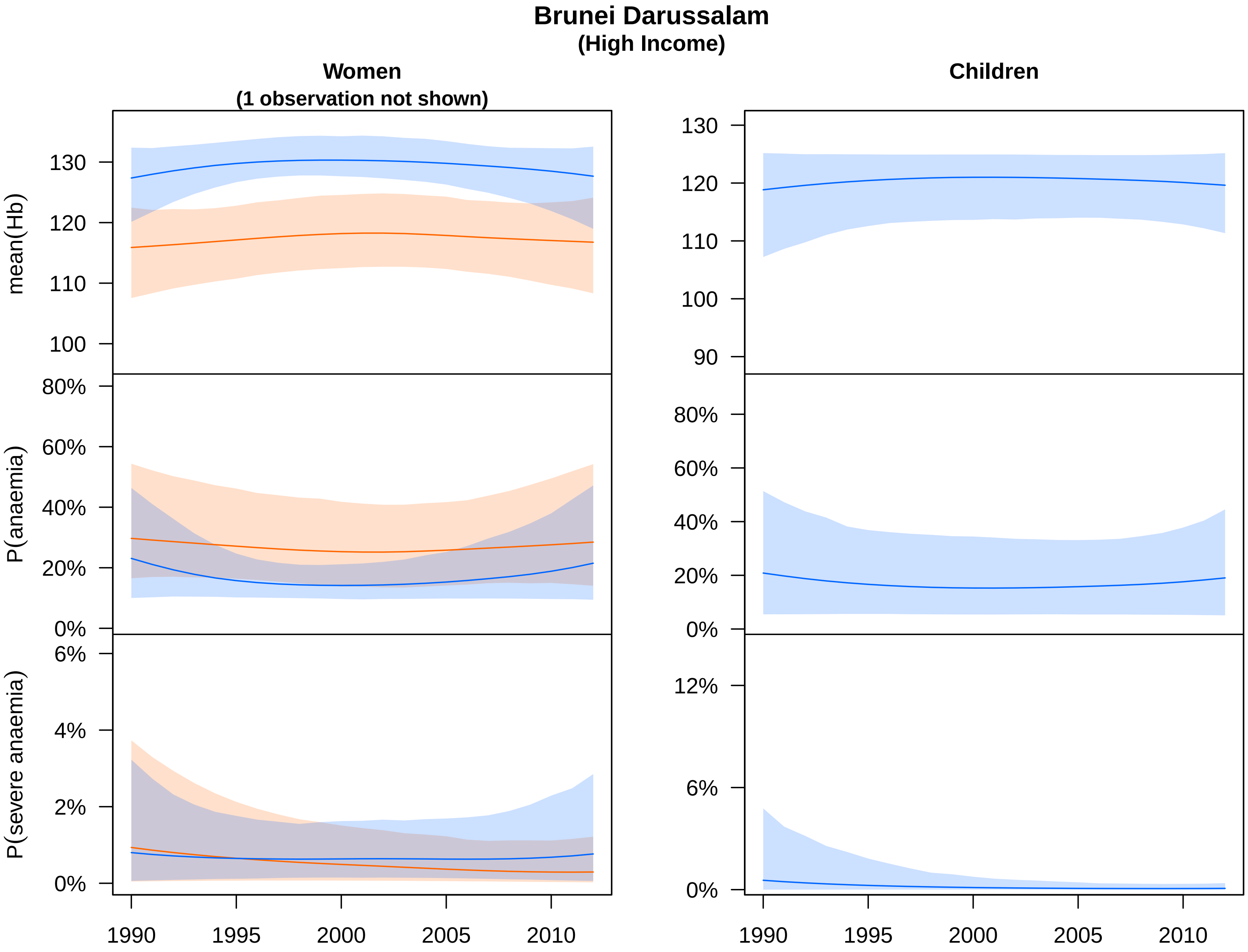
Brunei Darussalam
(High Income)
Women
(1 observation not shown)
Children
mean(Hb)
P(anaemia)
P(severe anaemia)
130
120
110
100
90
80%
60%
40%
20%
0%
6%
4%
2%
12%
6%
1990
1995
2000
2005
2010

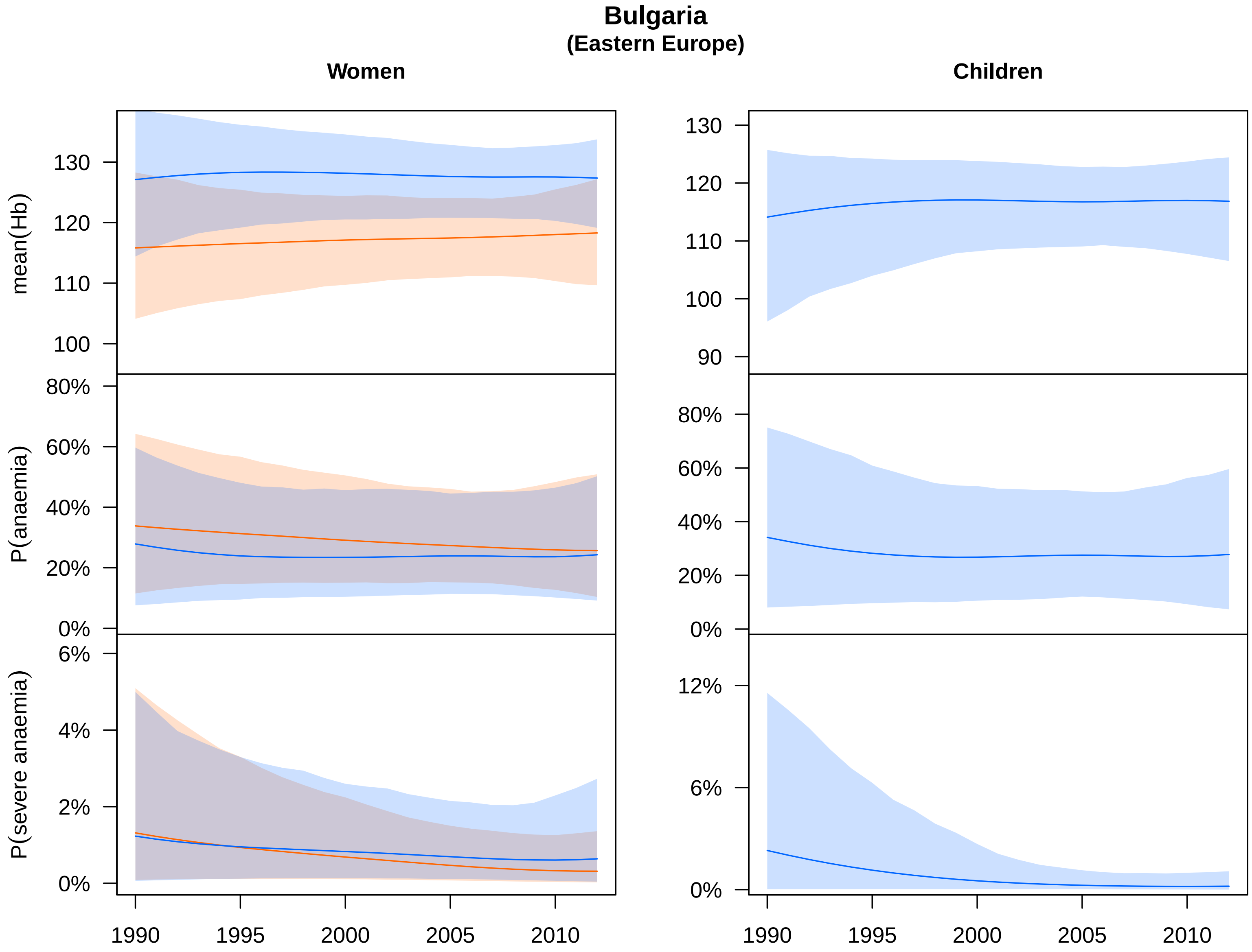
Bulgaria
(Eastern Europe)
Women
Children
mean(Hb)
P(anaemia)
P(severe anaemia)
130
120
110
100
90
80%
60%
40%
20%
0%
6%
4%
2%
12%
1990
1995
2000
2005
2010

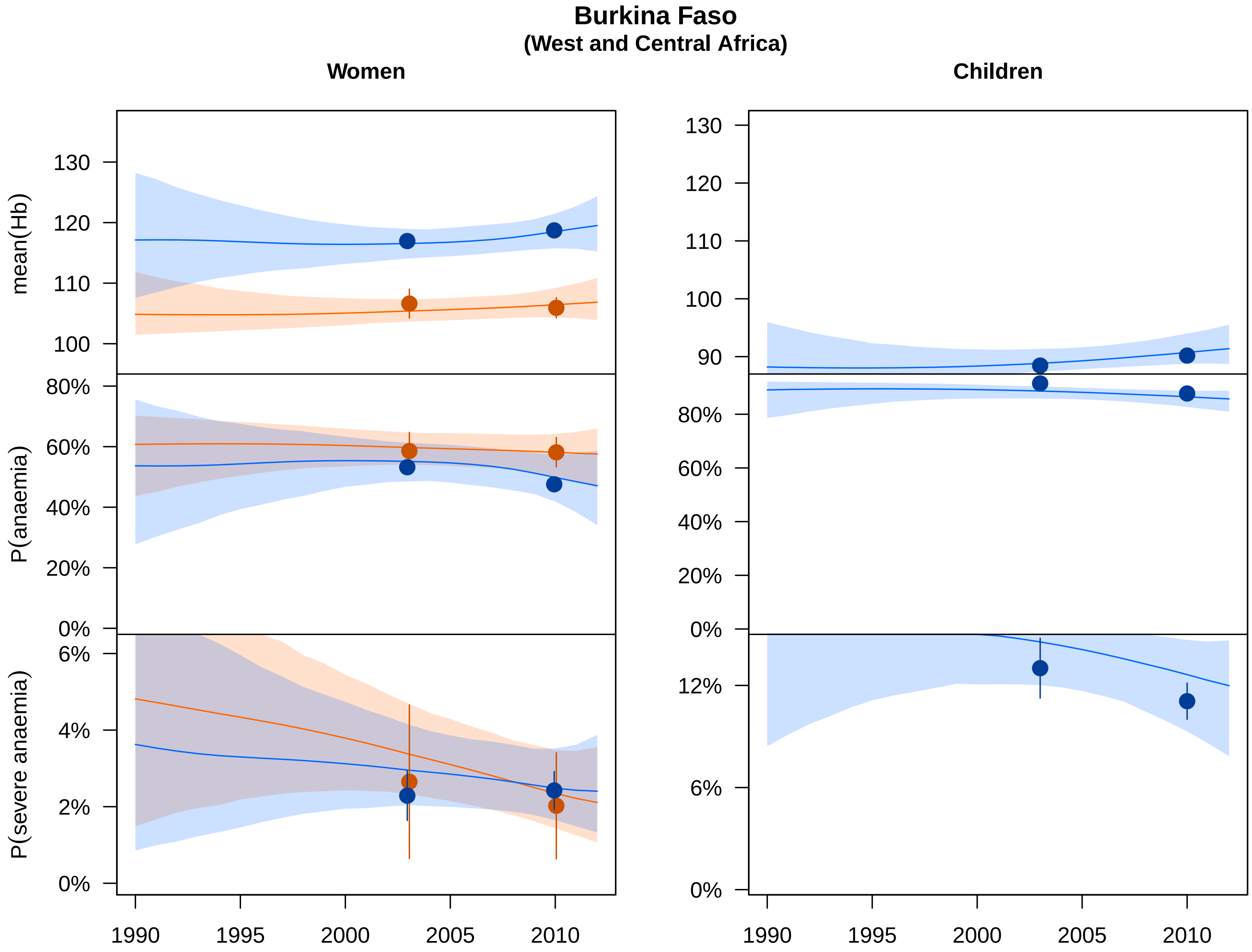
Burkina Faso
(West and Central Africa)
Women
Children
mean(Hb)
P(anaemia)
P(severe anaemia)
130
120
110
100
90
80%
60%
40%
20%
0%
6%
4%
2%
12%
1990
1995
2000
2005
2010

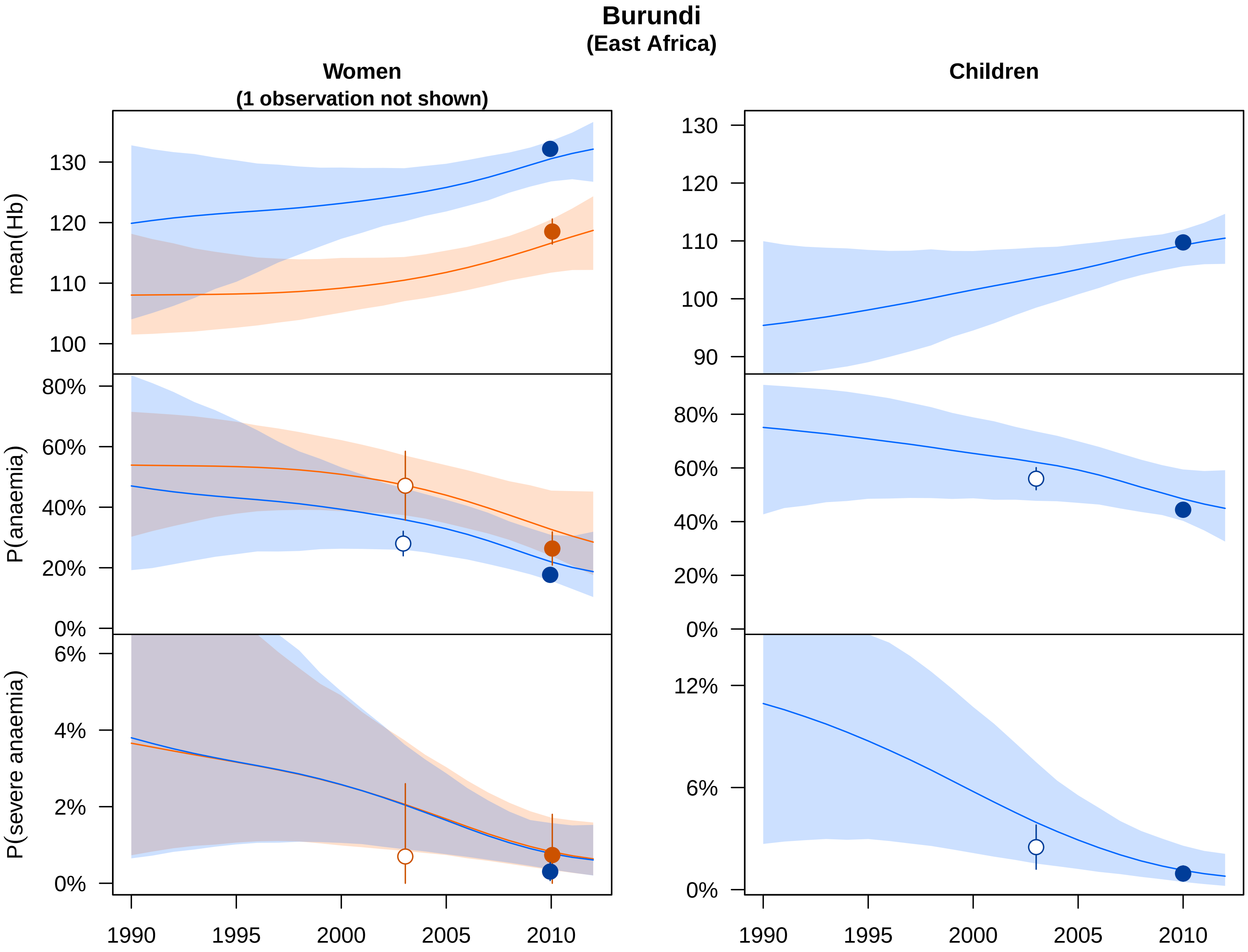
Burundi
(East Africa)
Women
(1 observation not shown)
Children
mean(Hb)
P(anaemia)
P(severe anaemia)
130
120
110
100
90
80%
60%
40%
20%
0%
6%
4%
2%
12%
1990
1995
2000
2005
2010

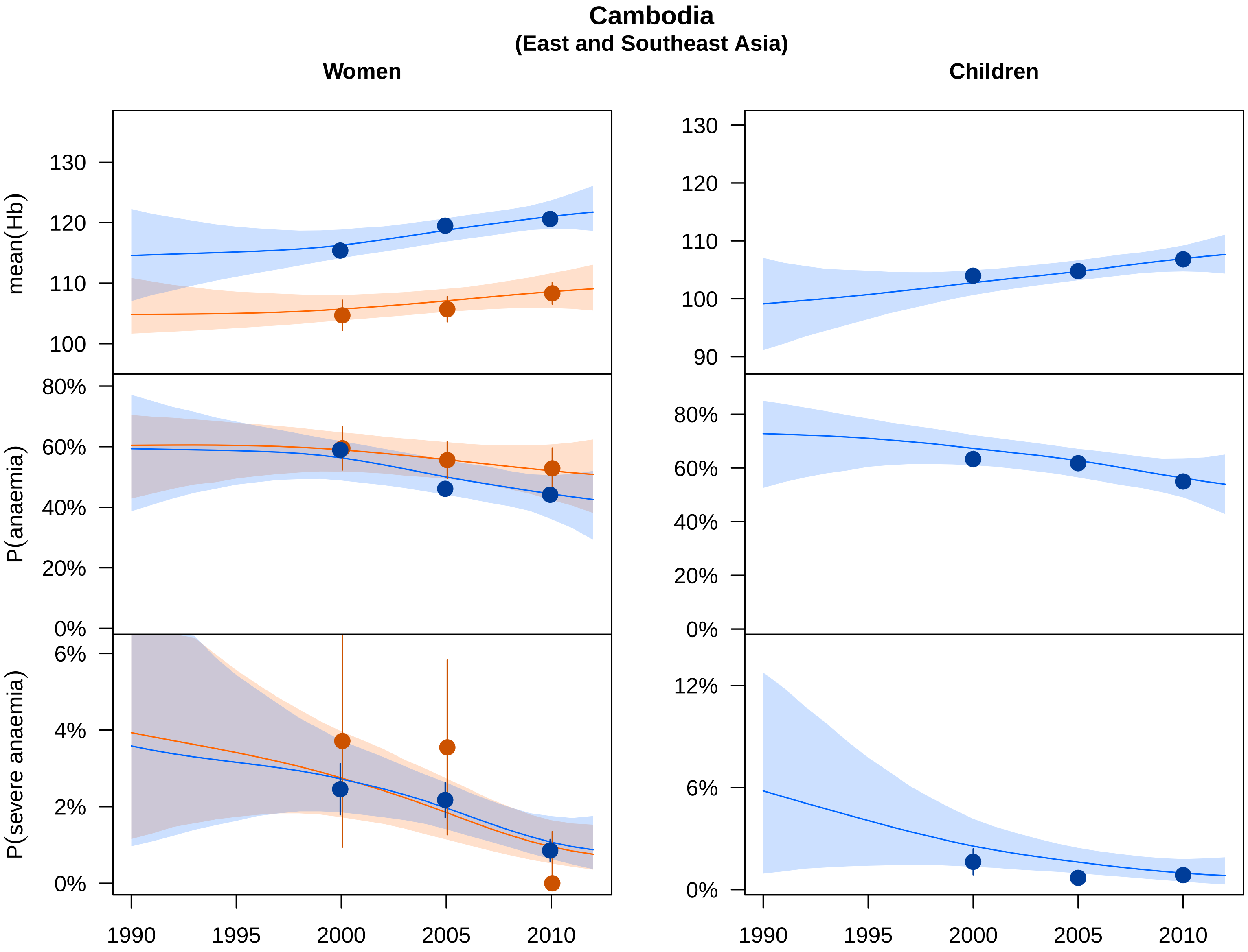

Cambodia
(East and Southeast Asia)
Women
Children
mean(Hb)
P(anaemia)
P(severe anaemia)
130
120
110
100
90
80%
60%
40%
20%
0%
6%
4%
2%
12%
1990
1995
2000
2005
2010

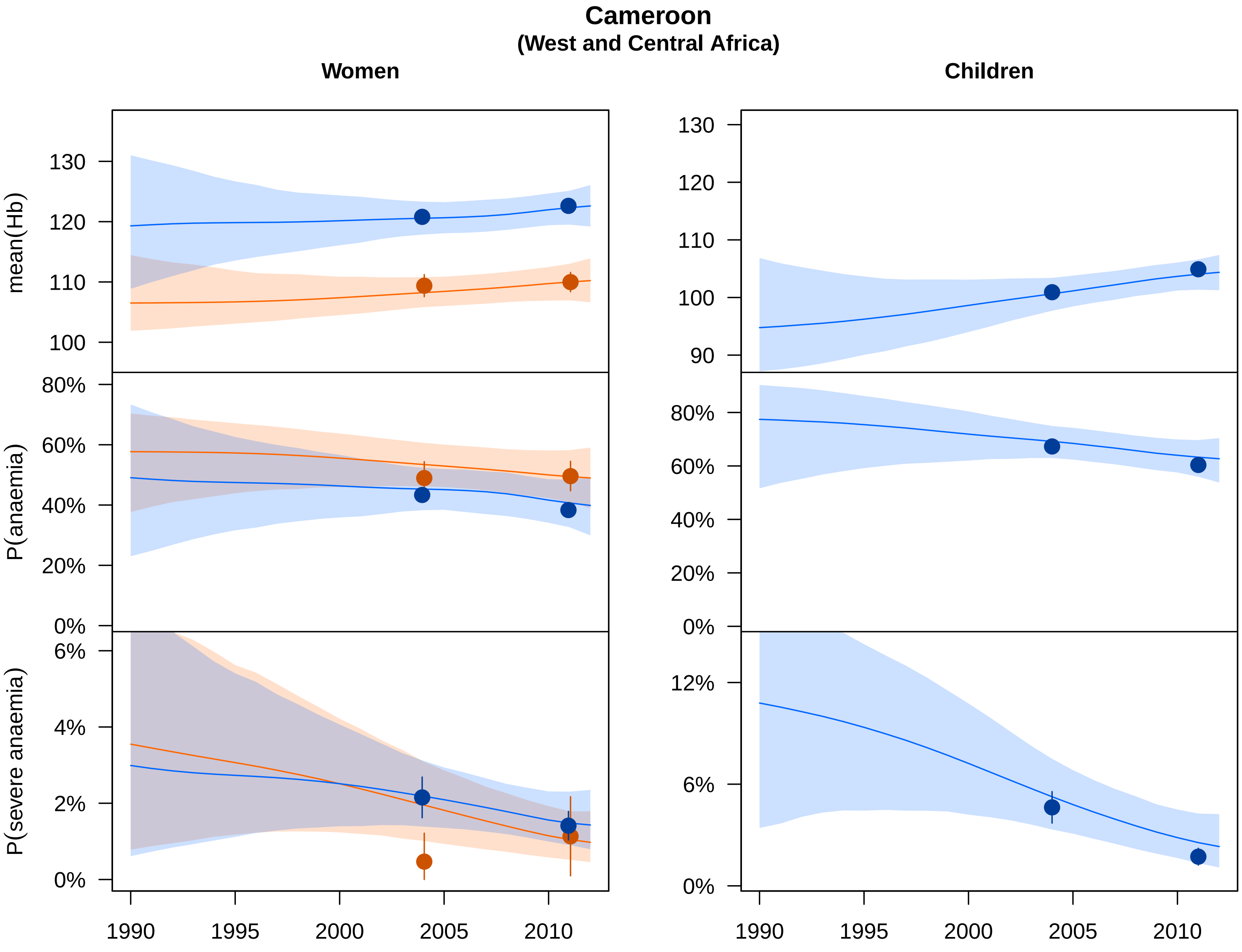
Cameroon
(West and Central Africa)
Women
Children
mean(Hb)
P(anaemia)
P(severe anaemia)
130
120
110
100
90
80%
60%
40%
20%
0%
6%
4%
2%
12%
1990
1995
2000
2005
2010

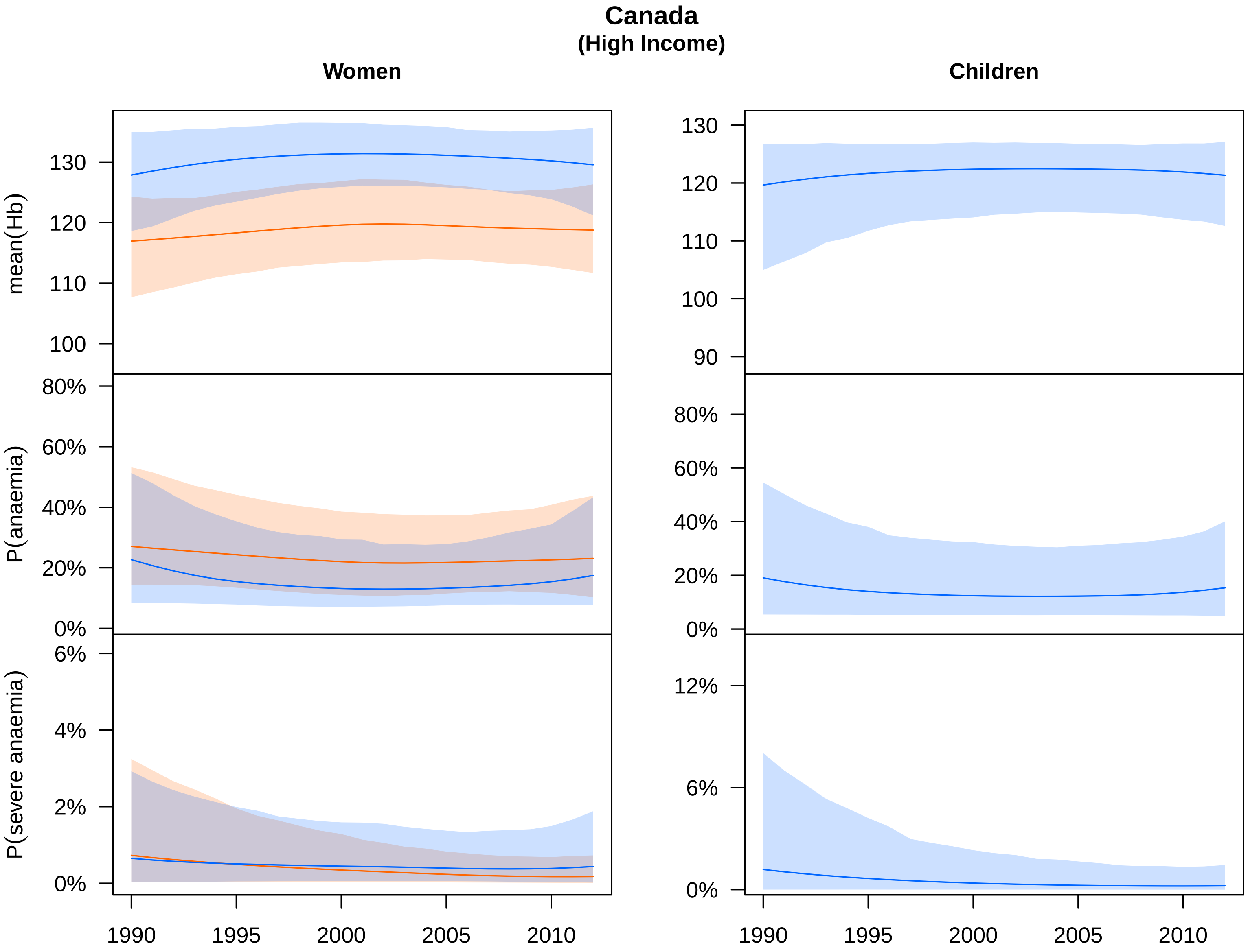

Canada
(High Income)
Women
Children
mean(Hb)
P(anaemia)
P(severe anaemia)
130
120
110
100
90
80%
60%
40%
20%
0%
6%
4%
2%
12%
1990
1995
2000
2005
2010

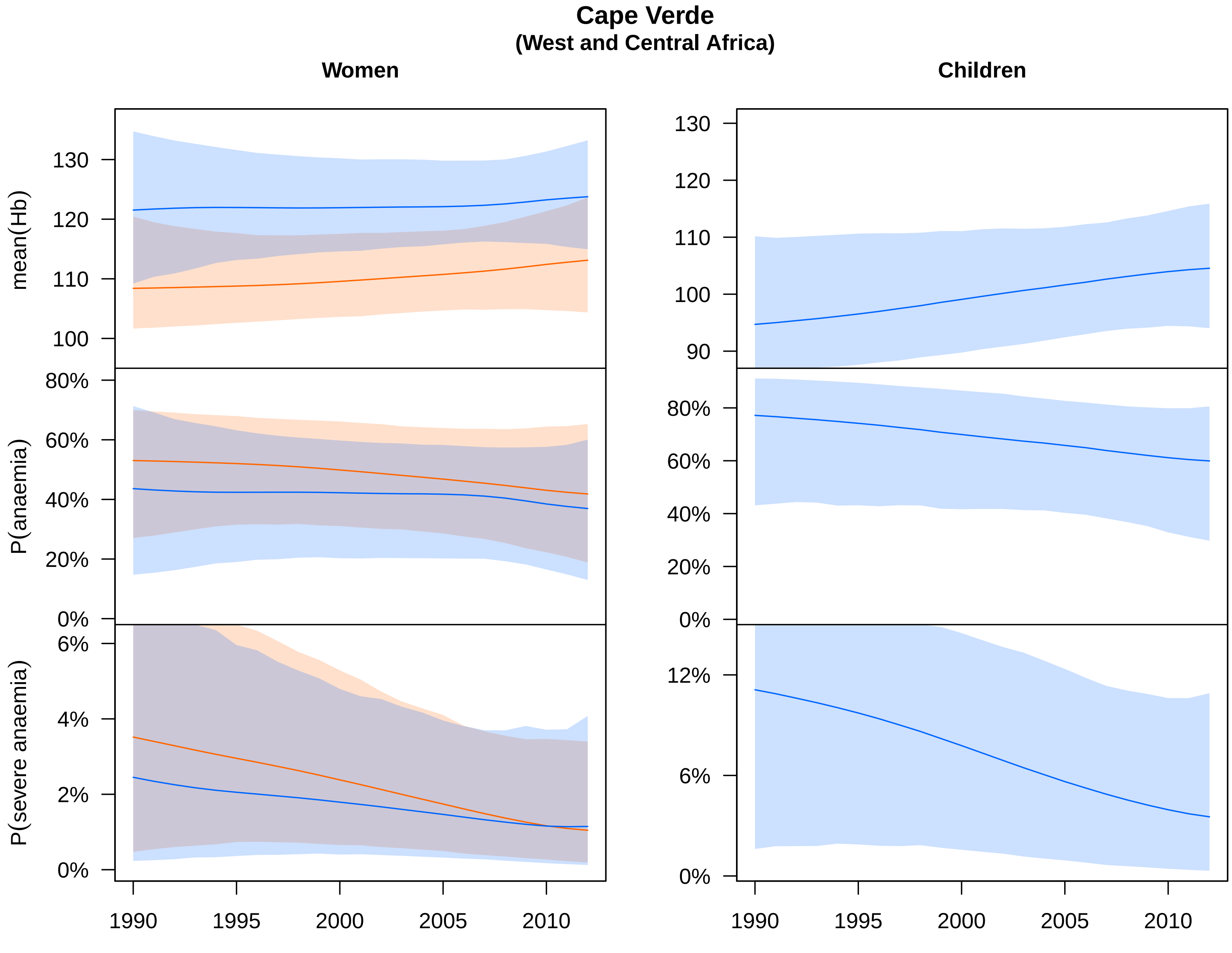
Cape Verde
(West and Central Africa)
Women
Children
mean(Hb)
P(anaemia)
P(severe anaemia)
130
120
110
100
90
80%
60%
40%
20%
0%
6%
4%
2%
12%
1990
1995
2000
2005
2010

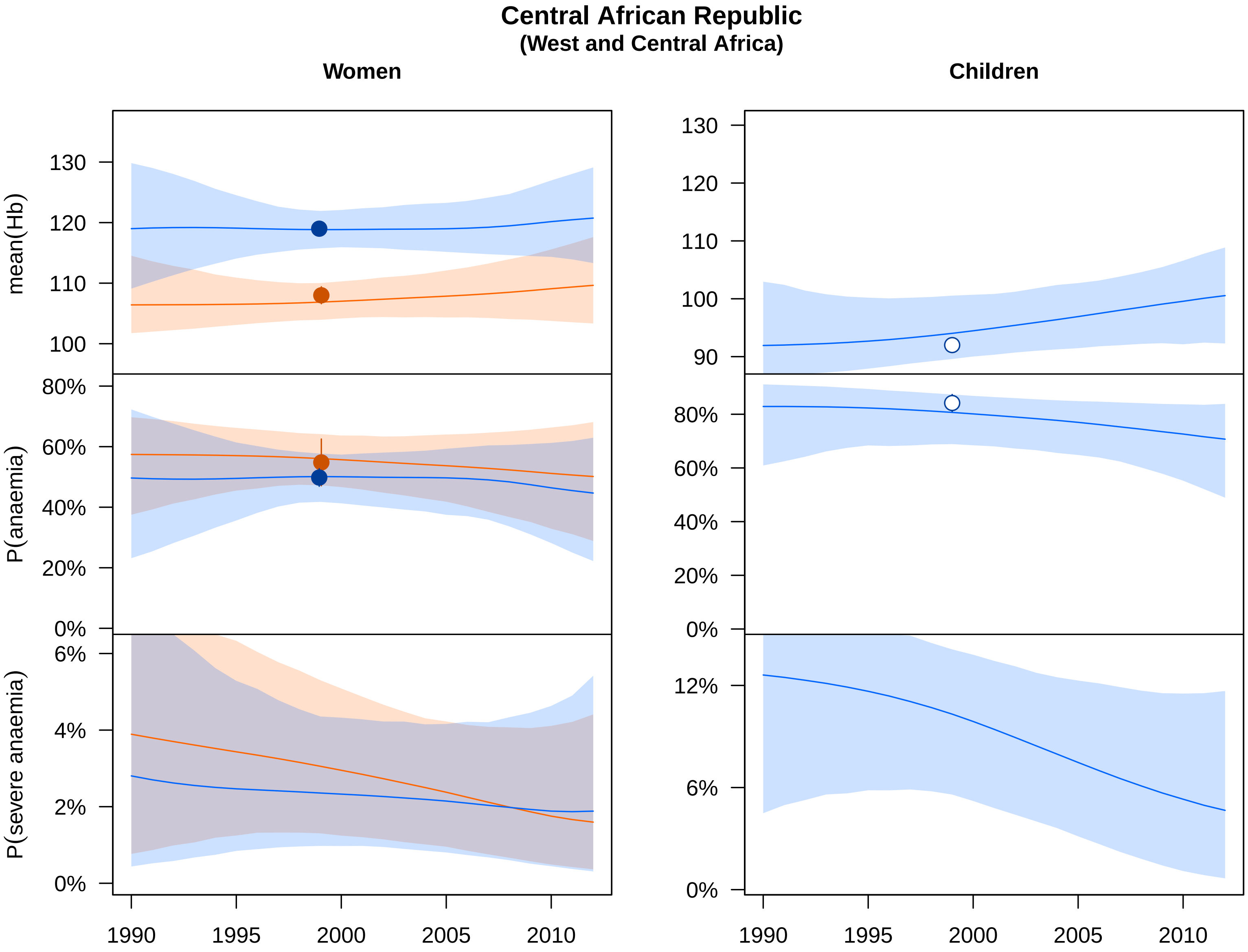

Central African Republic
(West and Central Africa)
Women
Children
mean(Hb)
P(anaemia)
P(severe anaemia)
130
120
110
100
90
80%
60%
40%
20%
0%
6%
4%
2%
12%
1990
1995
2000
2005
2010

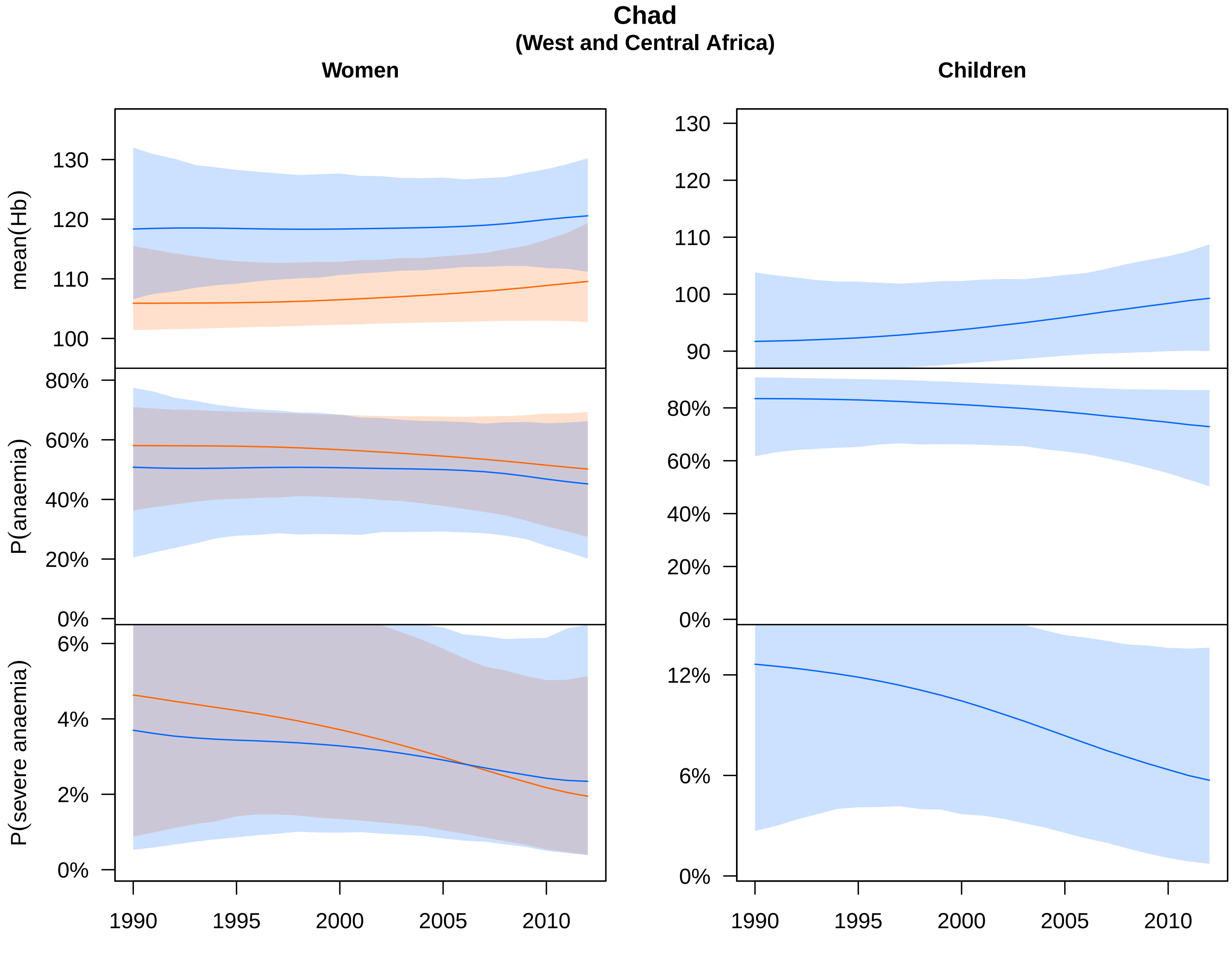

Chad
(West and Central Africa)
Women
Children
mean(Hb)
P(anaemia)
P(severe anaemia)
130
120
110
100
90
80%
60%
40%
20%
0%
6%
4%
2%
12%
1990
1995
2000
2005
2010

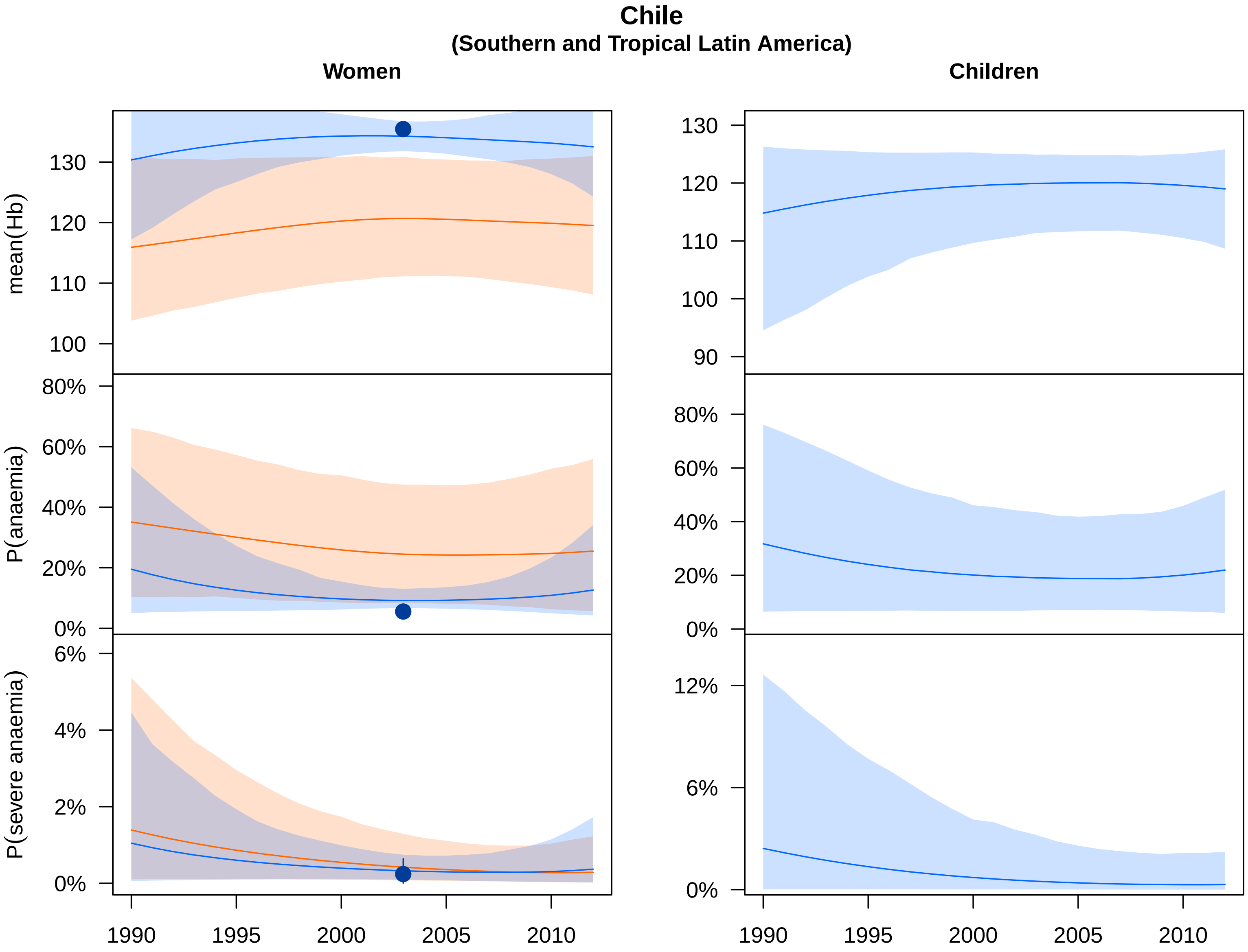
Chile
(Southern and Tropical Latin America)
Women
Children
mean(Hb)
P(anaemia)
P(severe anaemia)
130
120
110
100
90
80%
60%
40%
20%
0%
6%
4%
2%
12%
1990
1995
2000
2005
2010

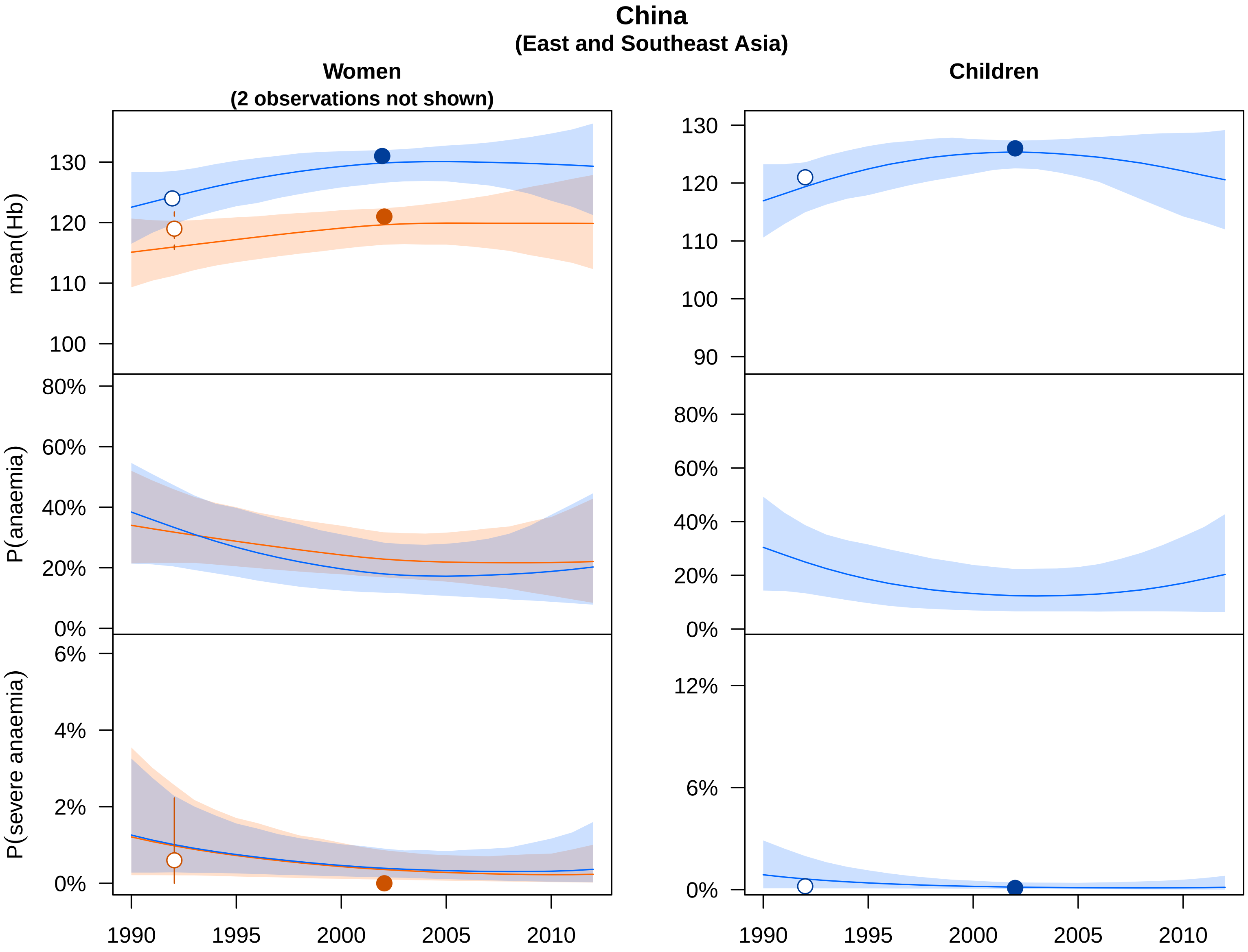
China
(East and Southeast Asia)
Women
(2 observations not shown)
Children
mean(Hb)
P(anaemia)
P(severe anaemia)
130
120
110
100
90
80%
60%
40%
20%
0%
6%
4%
2%
12%
1990
1995
2000
2005
2010

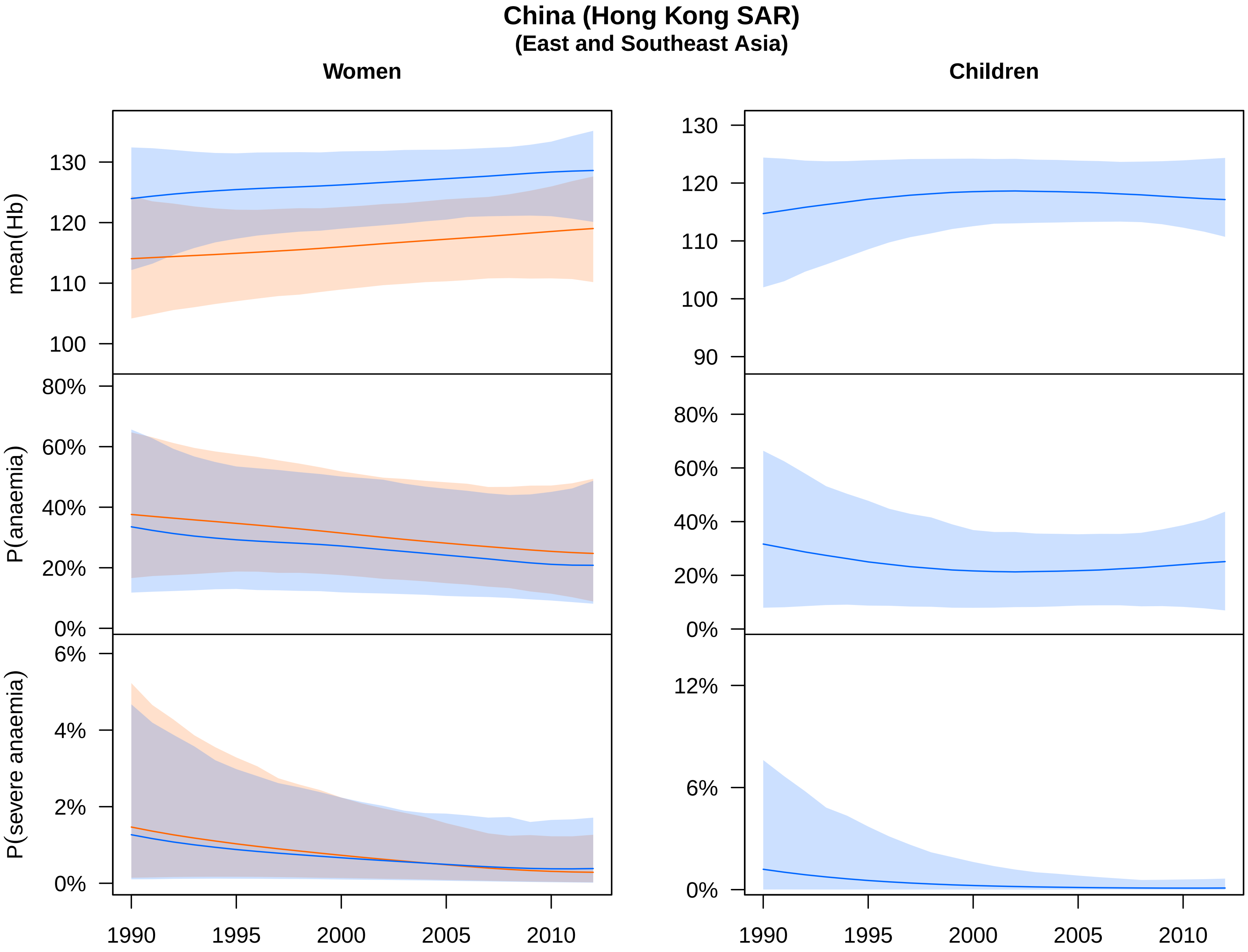
China (Hong Kong SAR)
(East and Southeast Asia)
Women
Children
mean(Hb)
P(anaemia)
P(severe anaemia)
100
110
120
130
90
0%
20%
40%
60%
80%
2%
4%
6%
12%
1990
1995
2000
2005
2010

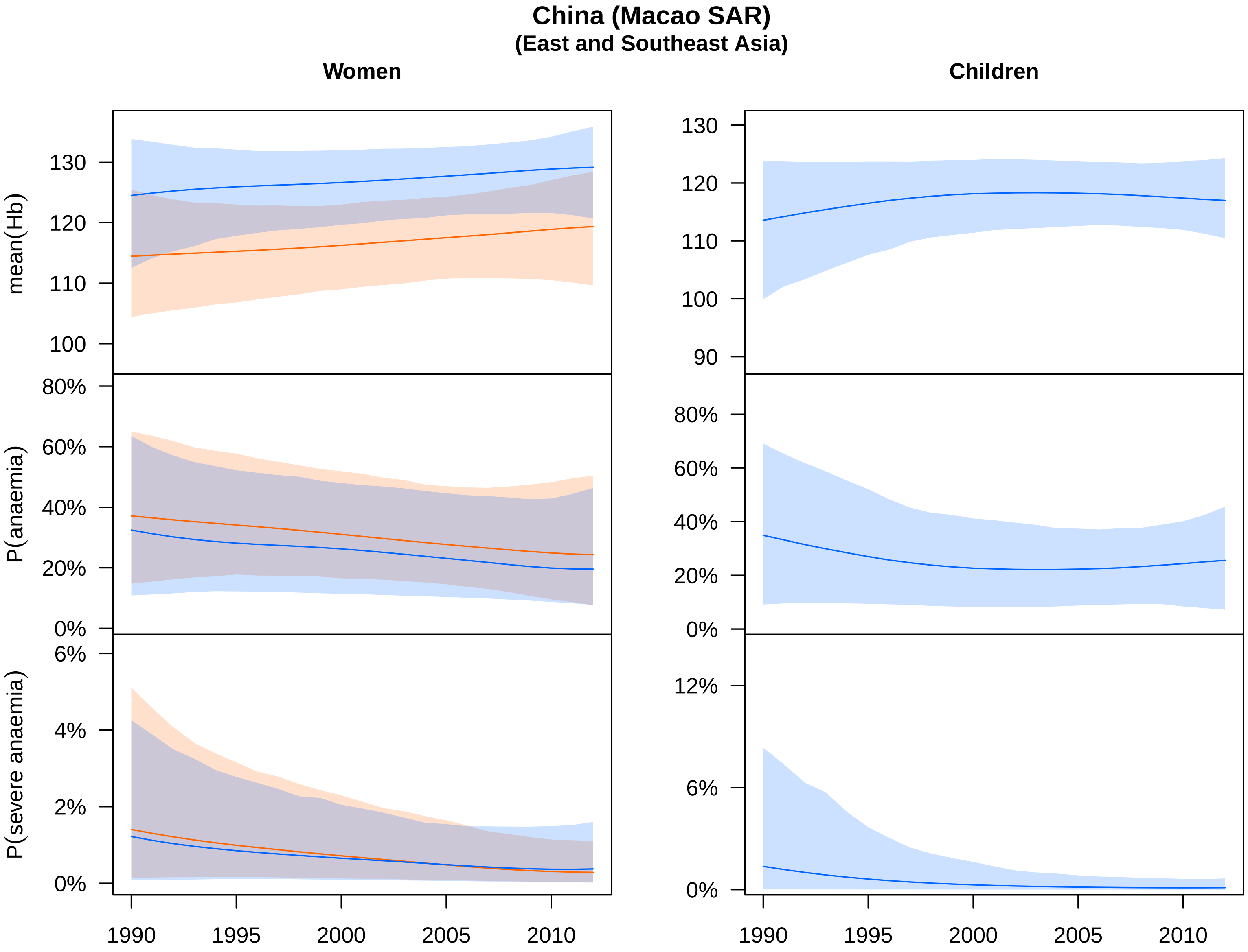

China (Macao SAR)
(East and Southeast Asia)
Women
Children
mean(Hb)
P(anaemia)
P(severe anaemia)
130
120
110
100
90
80%
60%
40%
20%
0%
6%
4%
2%
12%
1990
1995
2000
2005
2010

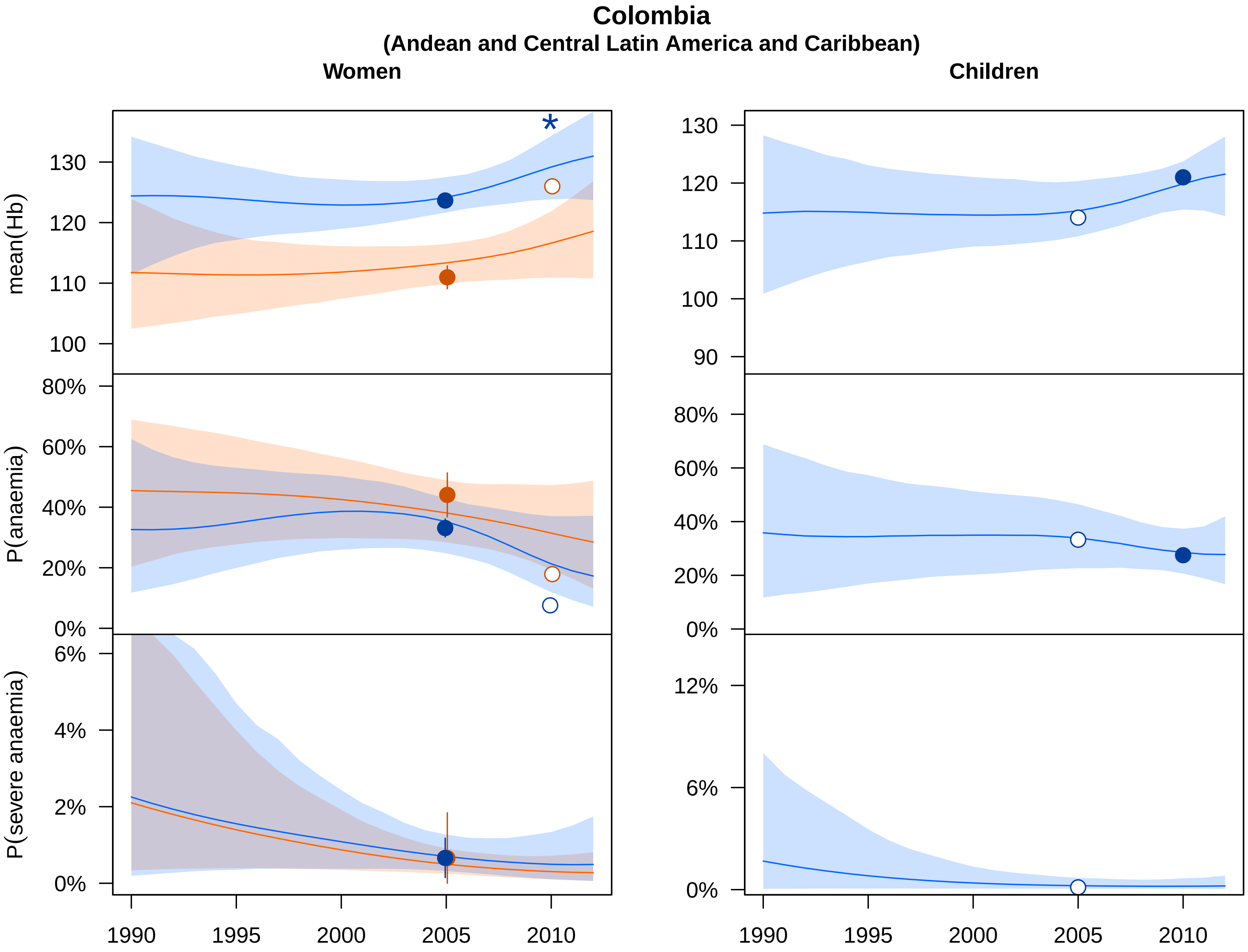
Colombia
(Andean and Central Latin America and Caribbean)
Women
Children
mean(Hb)
P(anaemia)
P(severe anaemia)
130
120
110
100
90
80%
60%
40%
20%
0%
6%
4%
2%
12%
1990
1995
2000
2005
2010

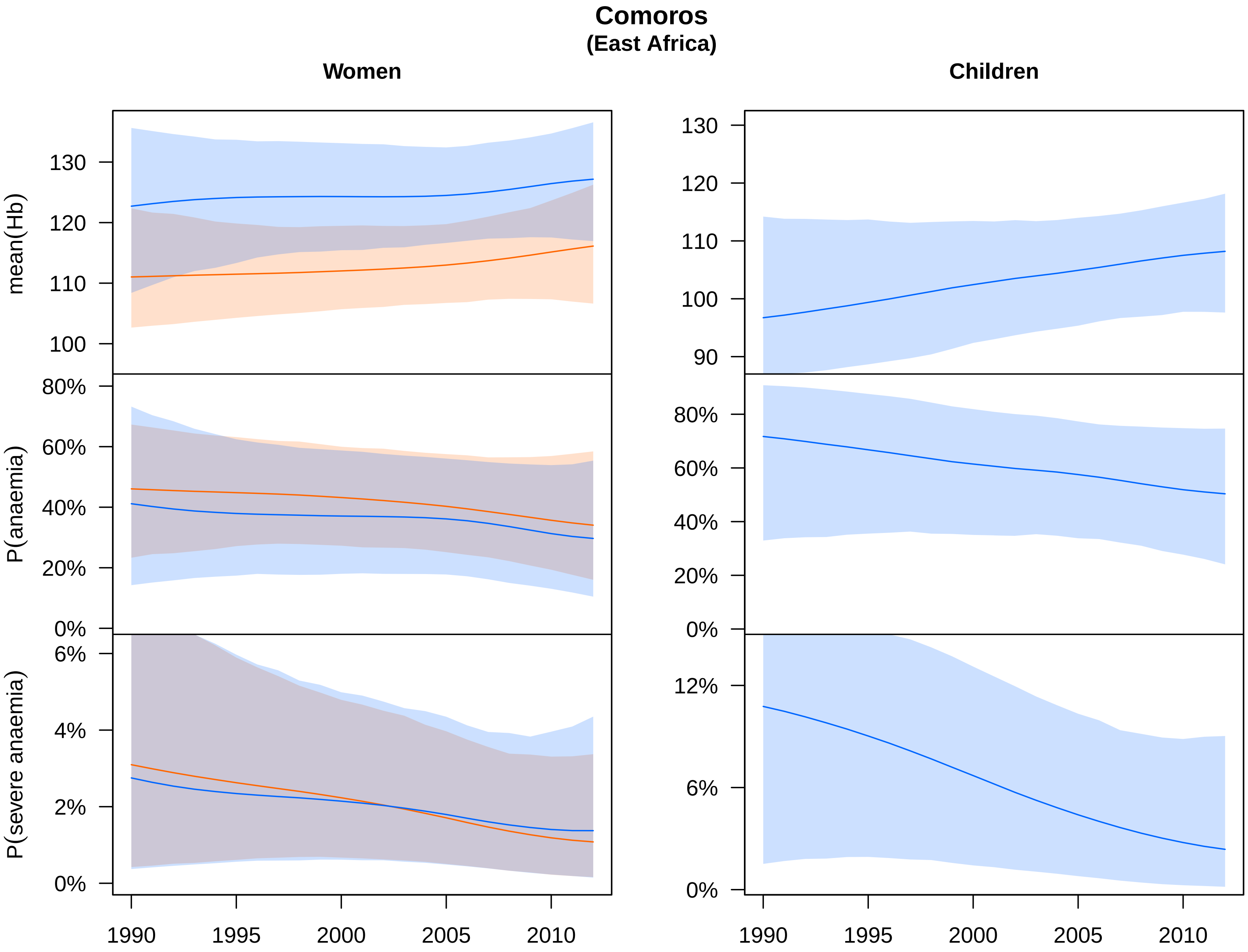
Comoros
(East Africa)
Women
Children
mean(Hb)
P(anaemia)
P(severe anaemia)
130
120
110
100
90
80%
60%
40%
20%
0%
6%
4%
2%
12%
1990
1995
2000
2005
2010

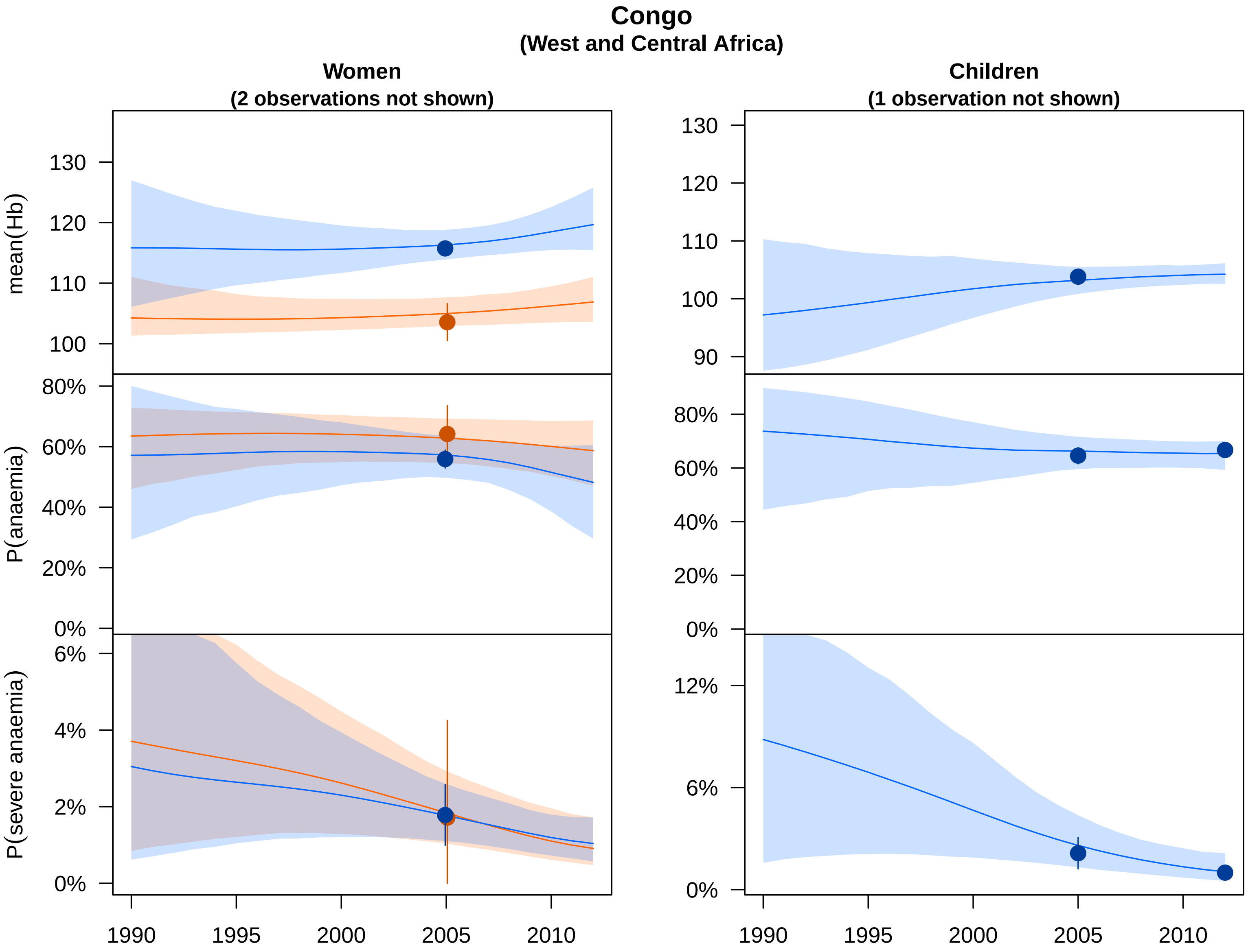
Congo
(West and Central Africa)
Women
(2 observations not shown)
Children
(1 observation not shown)
mean(Hb)
P(anaemia)
P(severe anaemia)
130
120
110
100
90
80%
60%
40%
20%
0%
6%
4%
2%
12%
1990
1995
2000
2005
2010

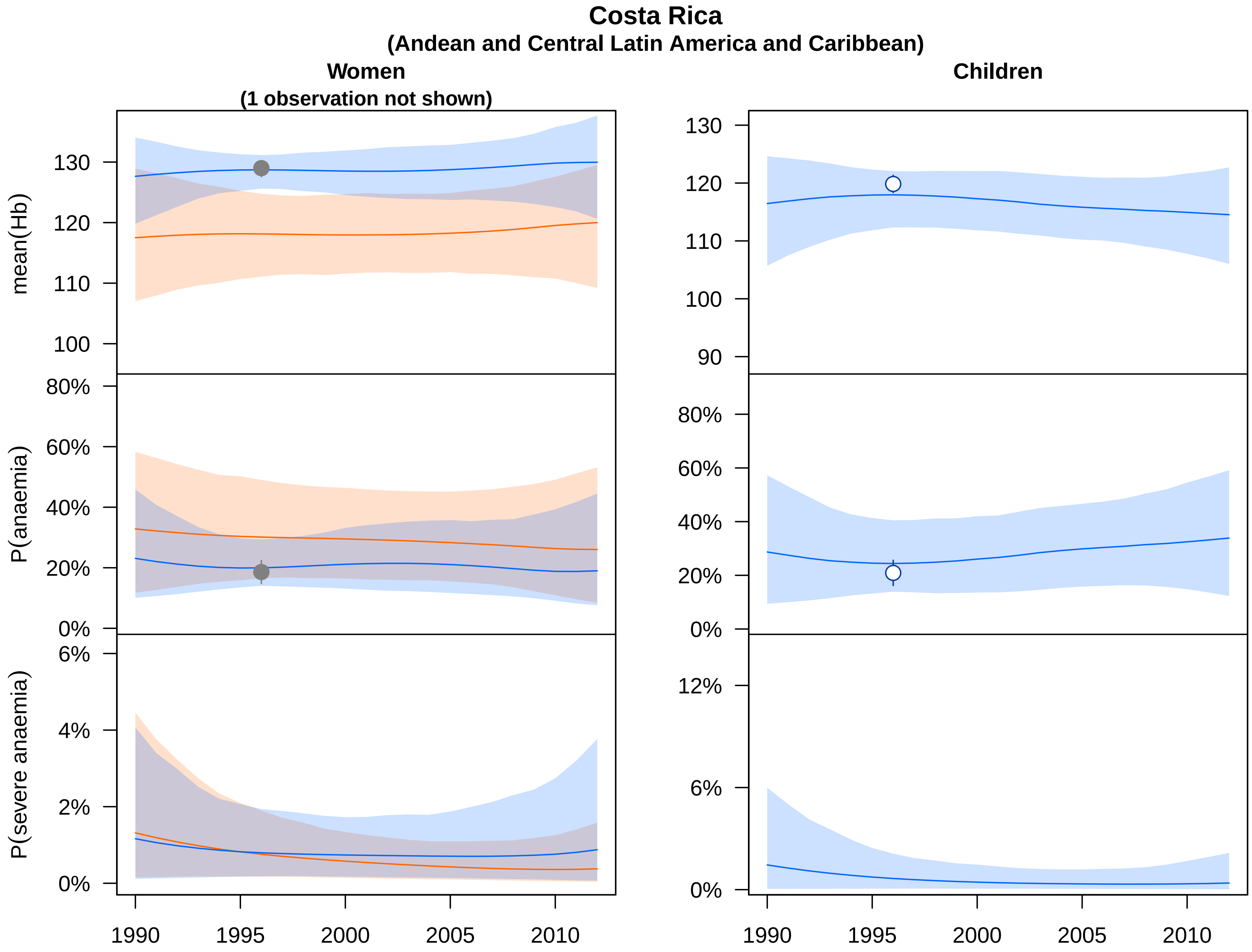
Costa Rica
(Andean and Central Latin America and Caribbean)
Women
(1 observation not shown)
Children
mean(Hb)
P(anaemia)
P(severe anaemia)
130
120
110
100
90
80%
60%
40%
20%
0%
6%
4%
2%
12%
1990
1995
2000
2005
2010

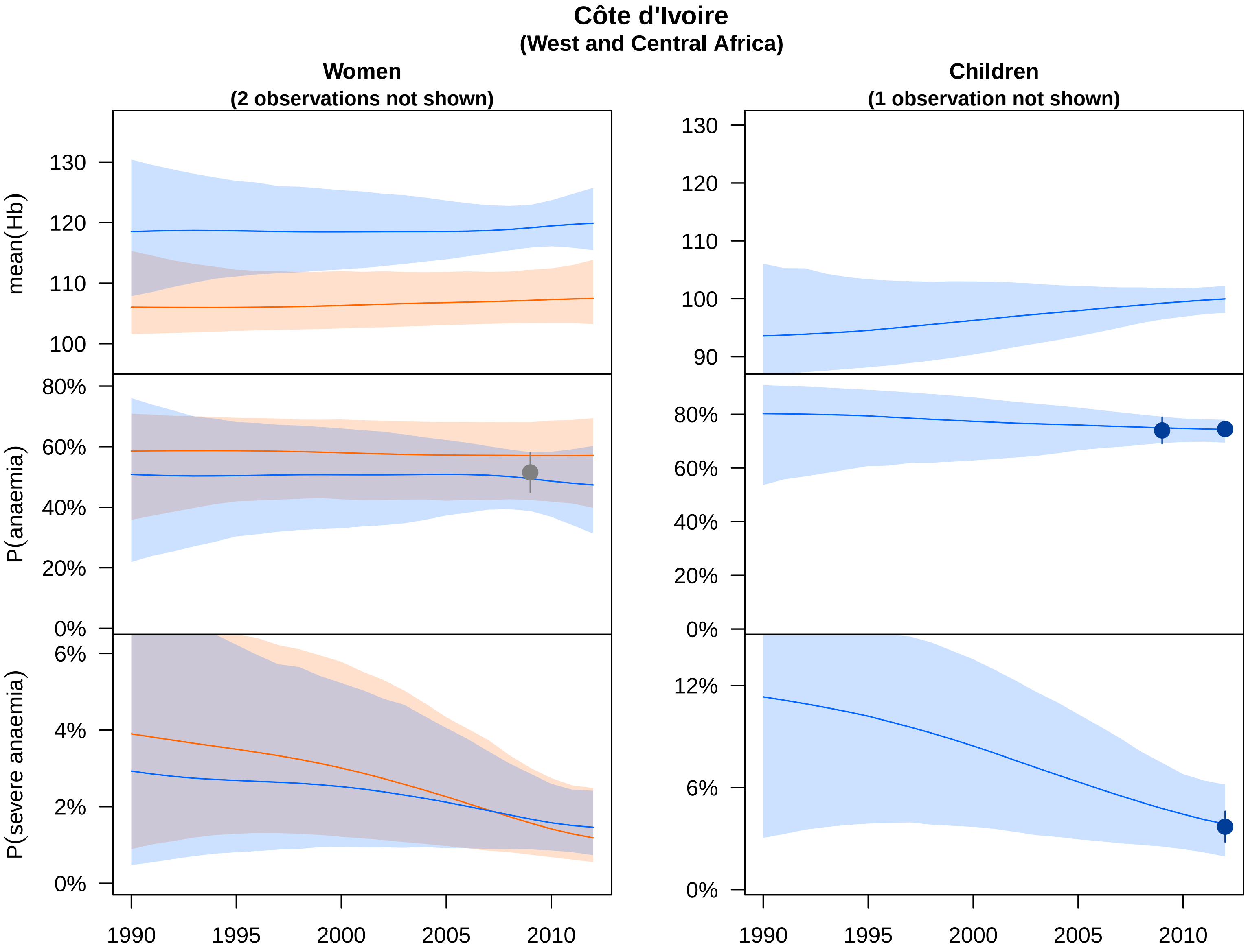

Côte d'Ivoire
(West and Central Africa)
Women
(2 observations not shown)
Children
(1 observation not shown)
mean(Hb)
P(anaemia)
P(severe anaemia)
130
120
110
100
90
80%
60%
40%
20%
0%
6%
4%
2%
12%
1990
1995
2000
2005
2010

# Croatia
## (Eastern Europe)

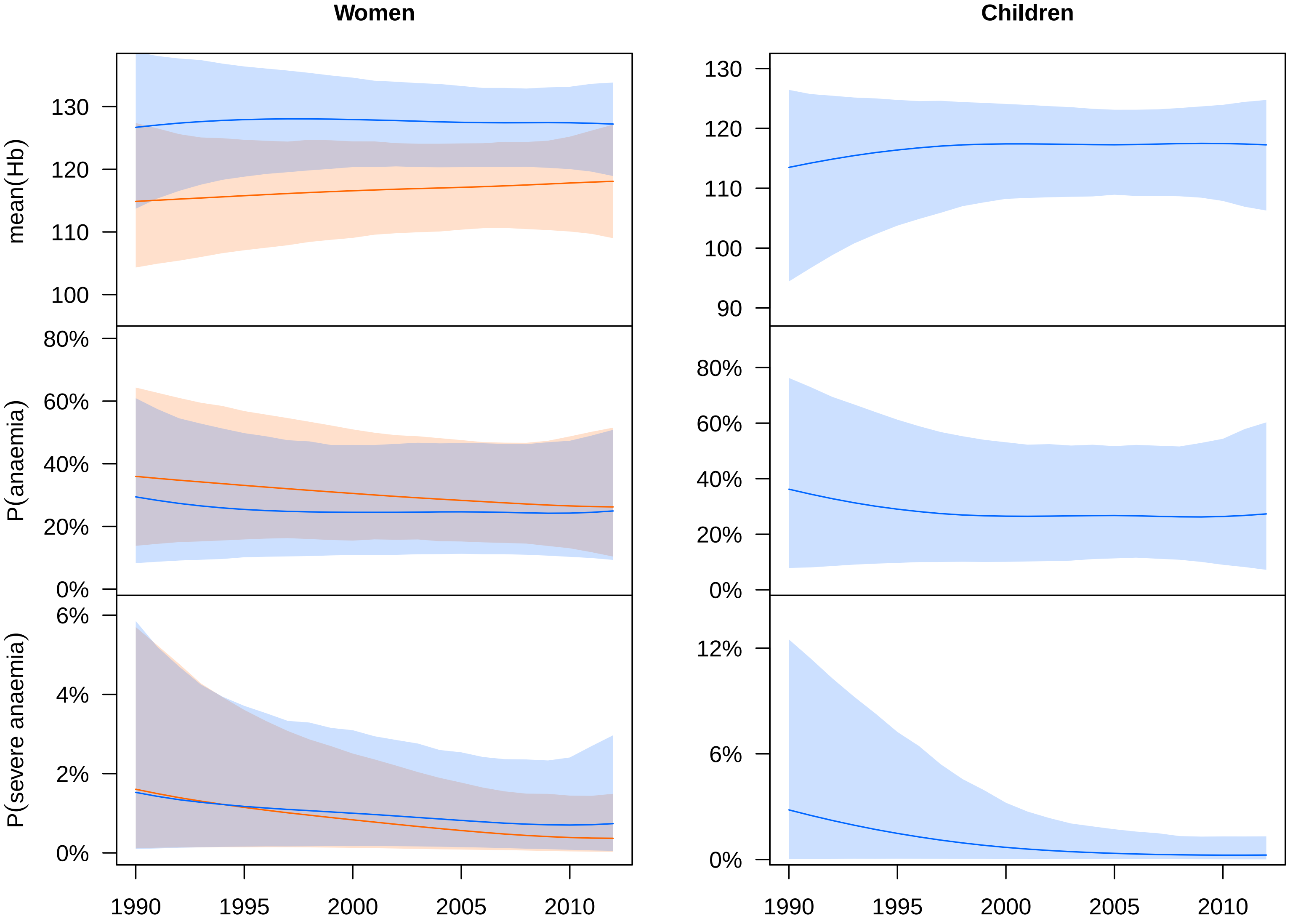

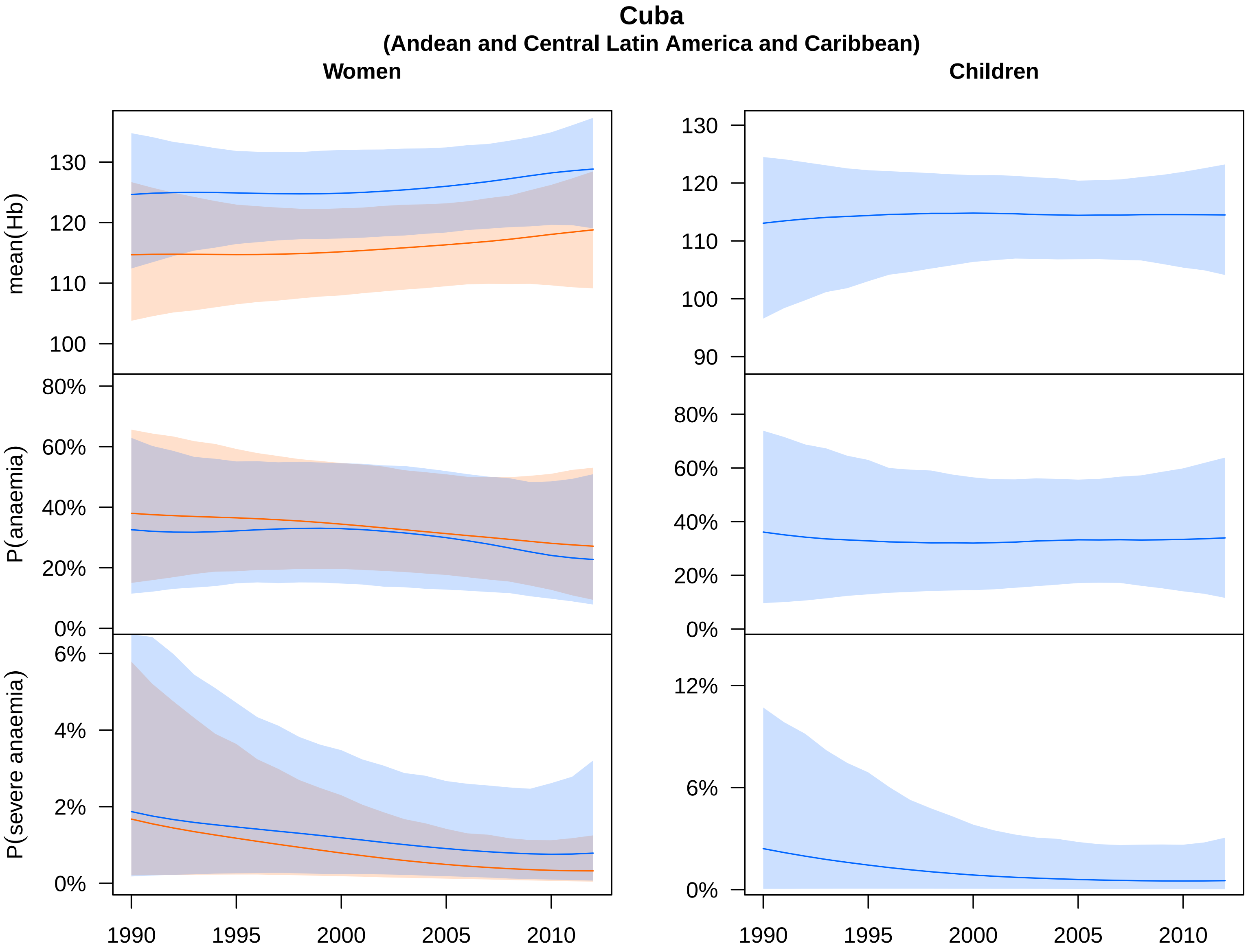

Cuba
(Andean and Central Latin America and Caribbean)
Women
Children
mean(Hb)
P(anaemia)
P(severe anaemia)
130
120
110
100
90
80%
60%
40%
20%
0%
6%
4%
2%
12%
1990
1995
2000
2005
2010

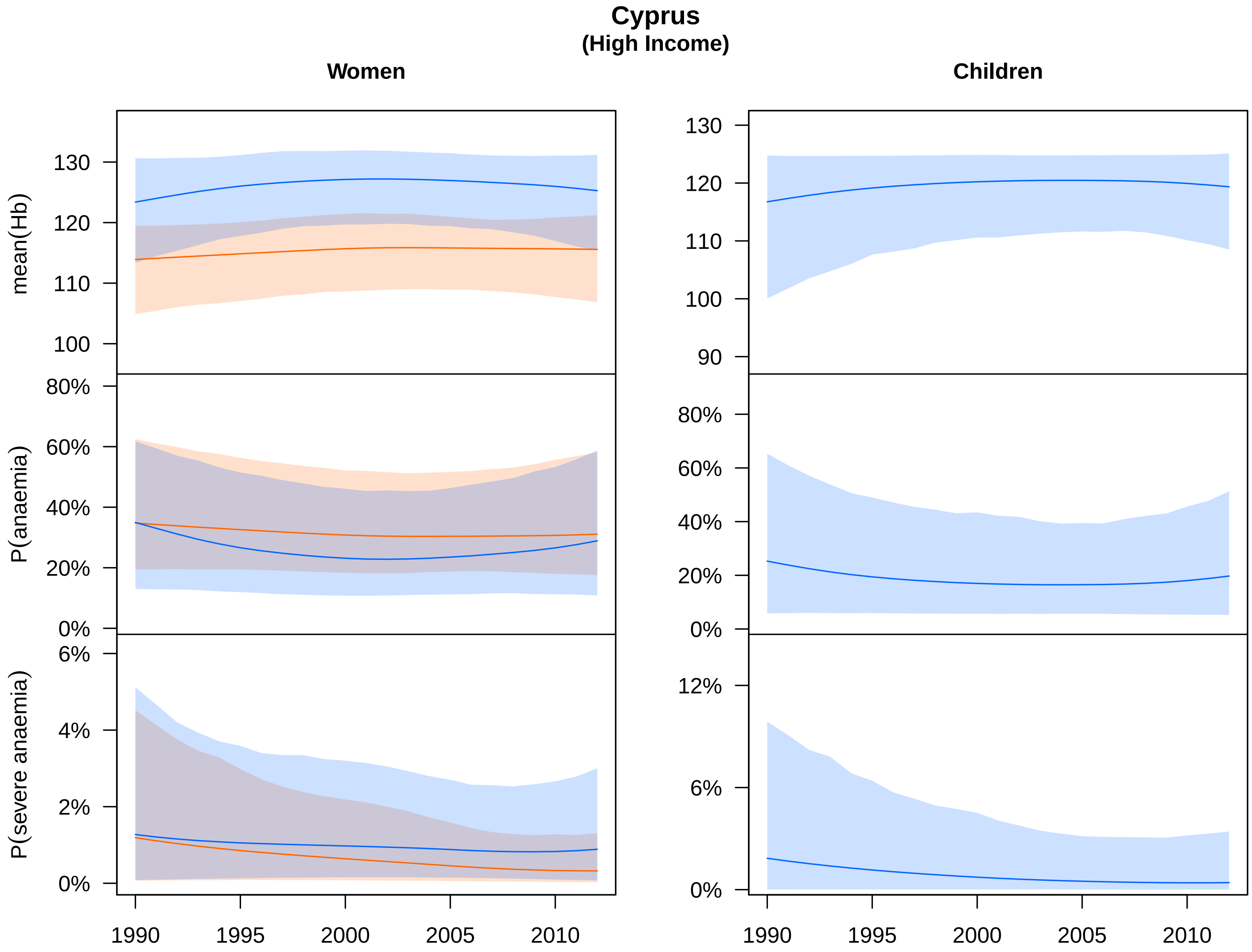
Cyprus
(High Income)
Women
Children
mean(Hb)
P(anaemia)
P(severe anaemia)
130
120
110
100
90
80%
60%
40%
20%
0%
6%
4%
2%
12%
1990
1995
2000
2005
2010

**Czech Republic**
**(Eastern Europe)**

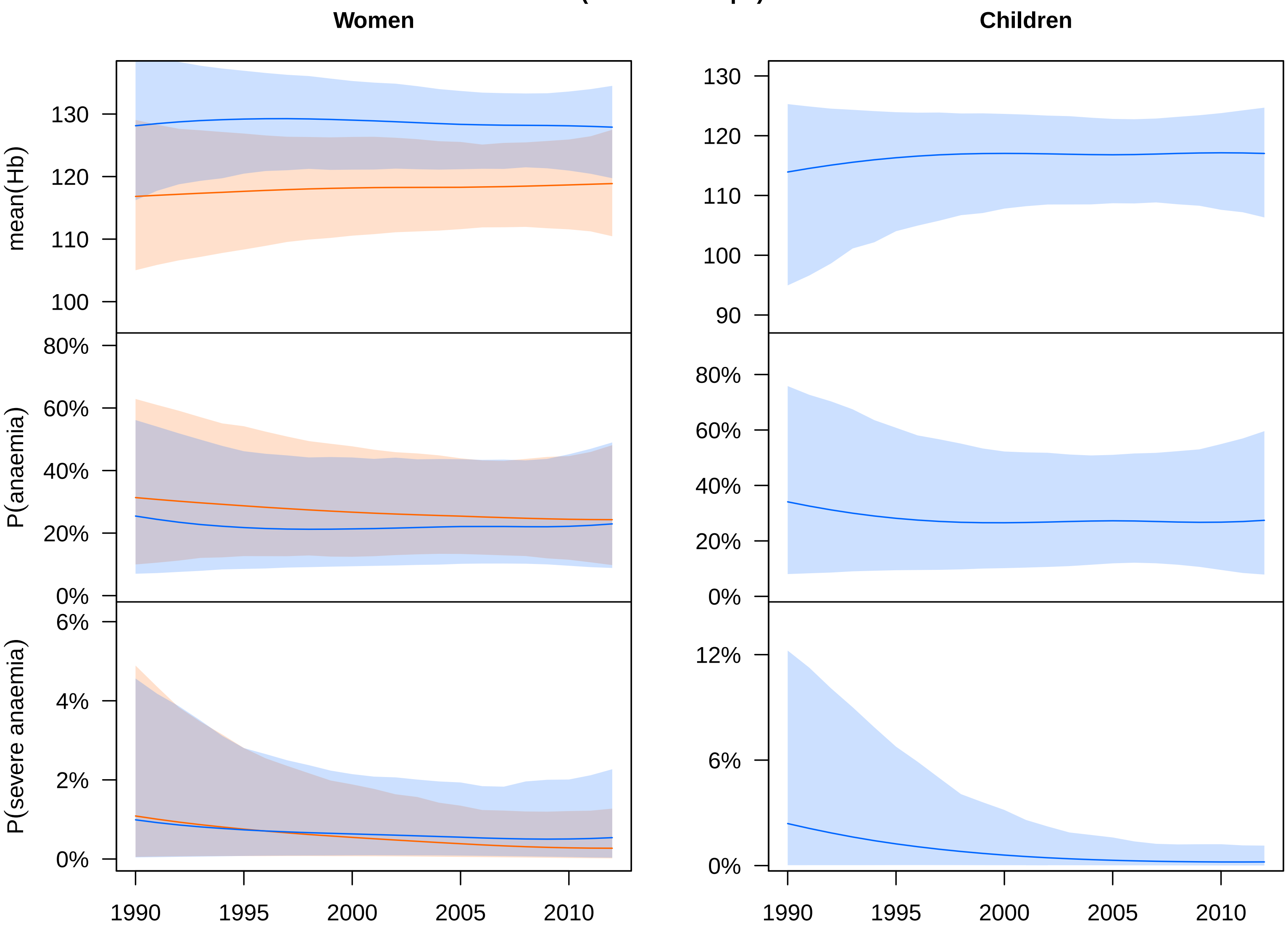

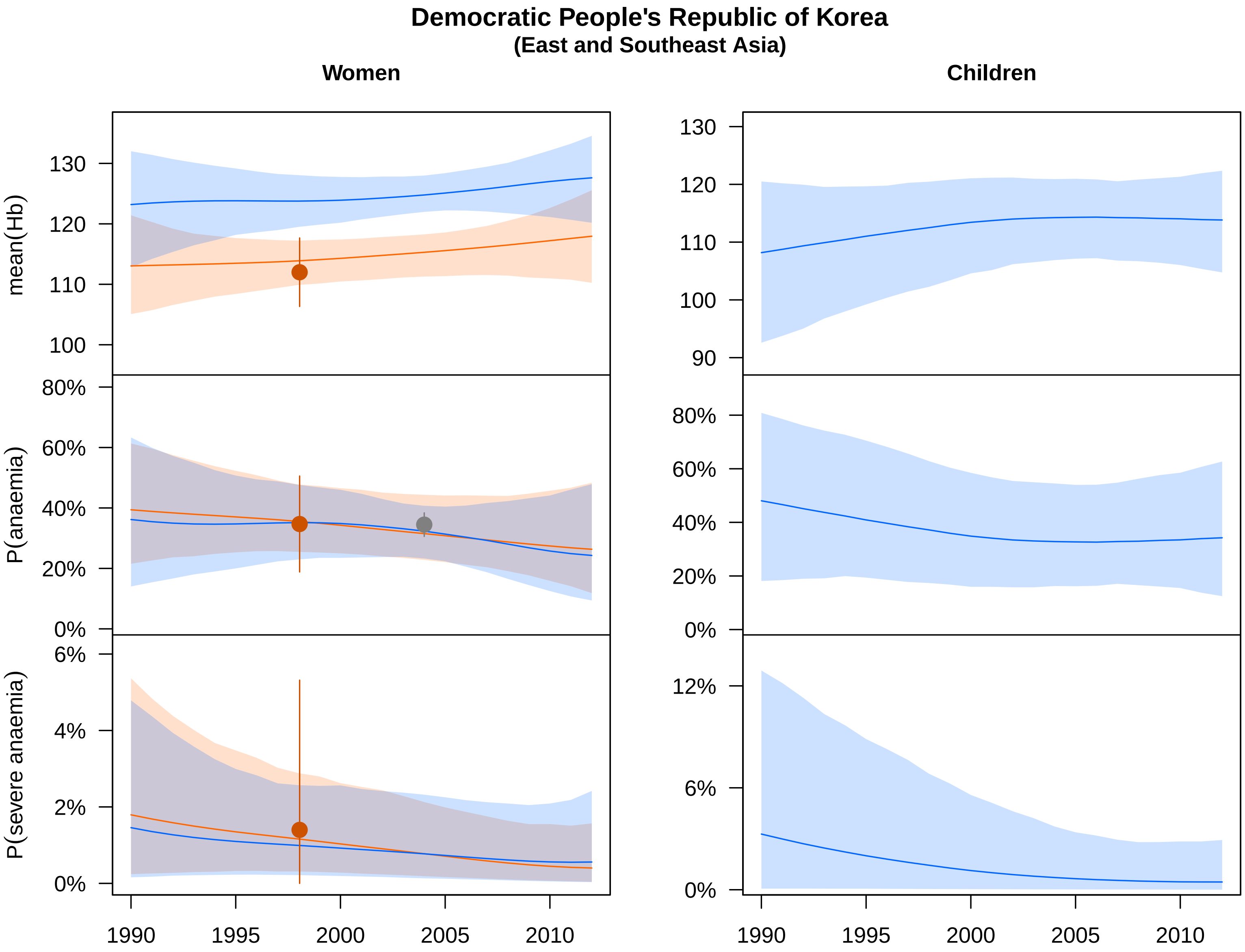
Democratic People's Republic of Korea
(East and Southeast Asia)
Women
Children
mean(Hb)
P(anaemia)
P(severe anaemia)
130
120
110
100
90
80%
60%
40%
20%
0%
6%
4%
2%
12%
1990
1995
2000
2005
2010

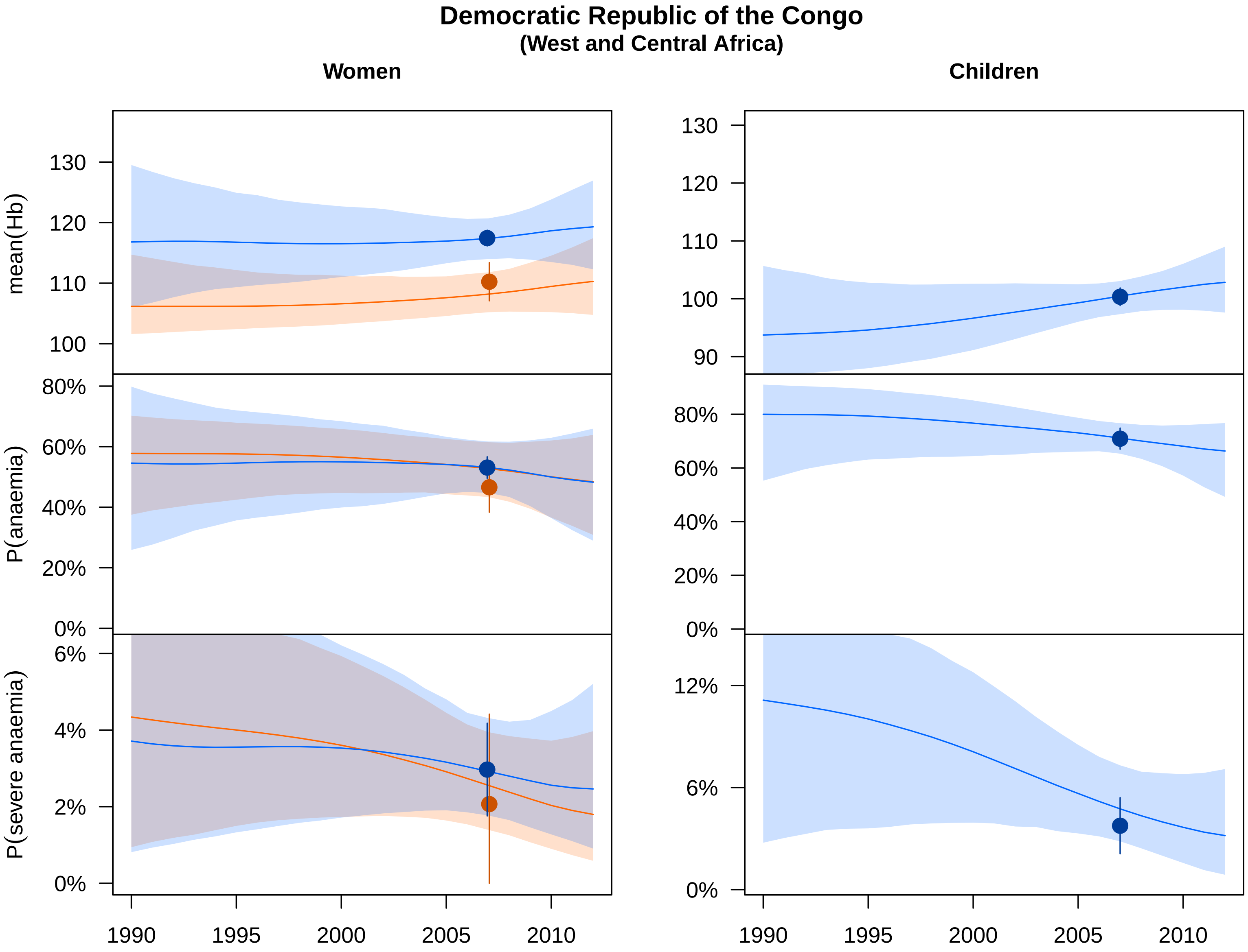
Democratic Republic of the Congo
(West and Central Africa)
Women
Children
mean(Hb)
P(anaemia)
P(severe anaemia)
130
120
110
100
90
80%
60%
40%
20%
0%
6%
4%
2%
12%
1990
1995
2000
2005
2010

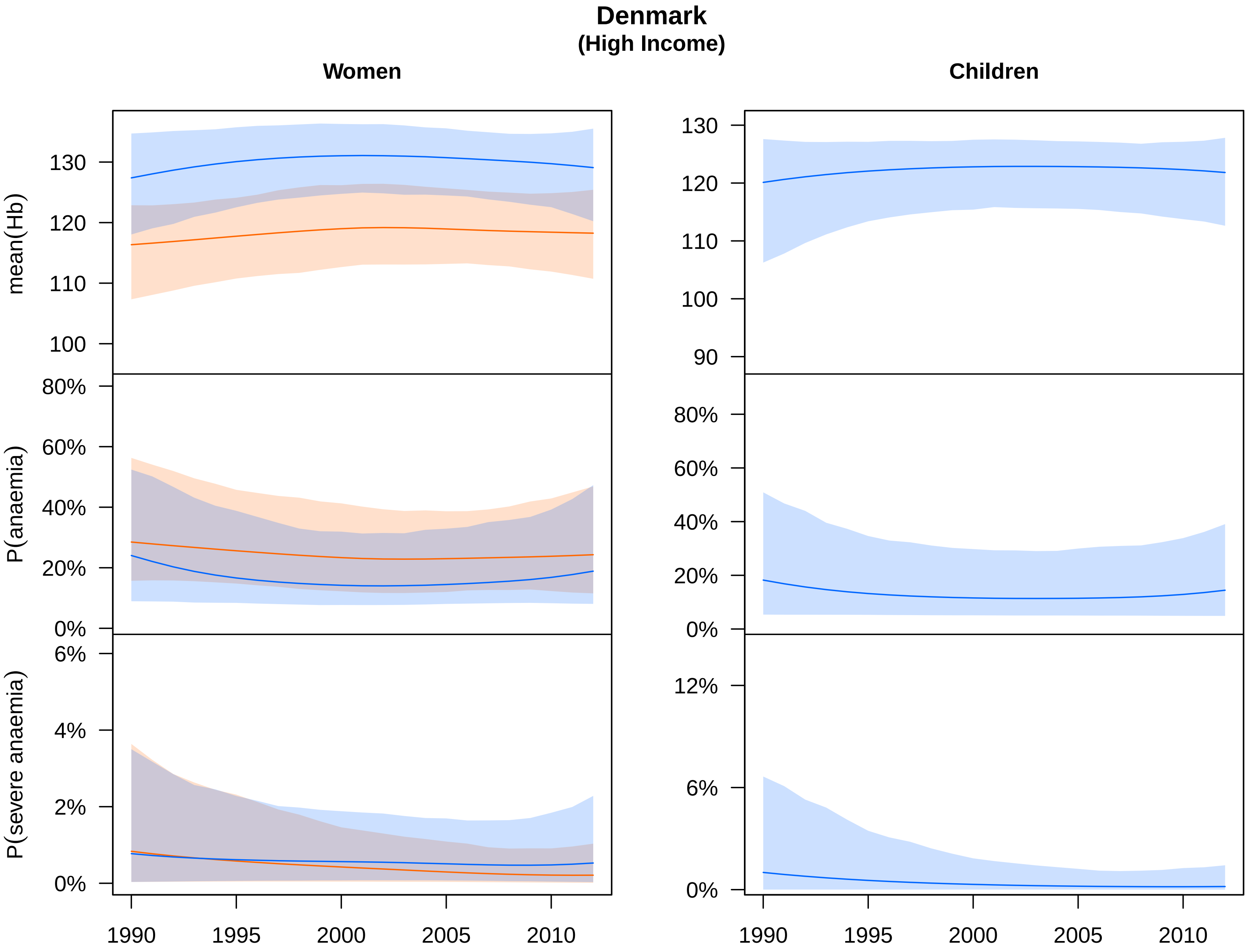
Denmark
(High Income)
Women
Children
mean(Hb)
P(anaemia)
P(severe anaemia)
130
120
110
100
90
80%
60%
40%
20%
0%
6%
4%
2%
12%
1990
1995
2000
2005
2010

## Djibouti
### (East Africa)

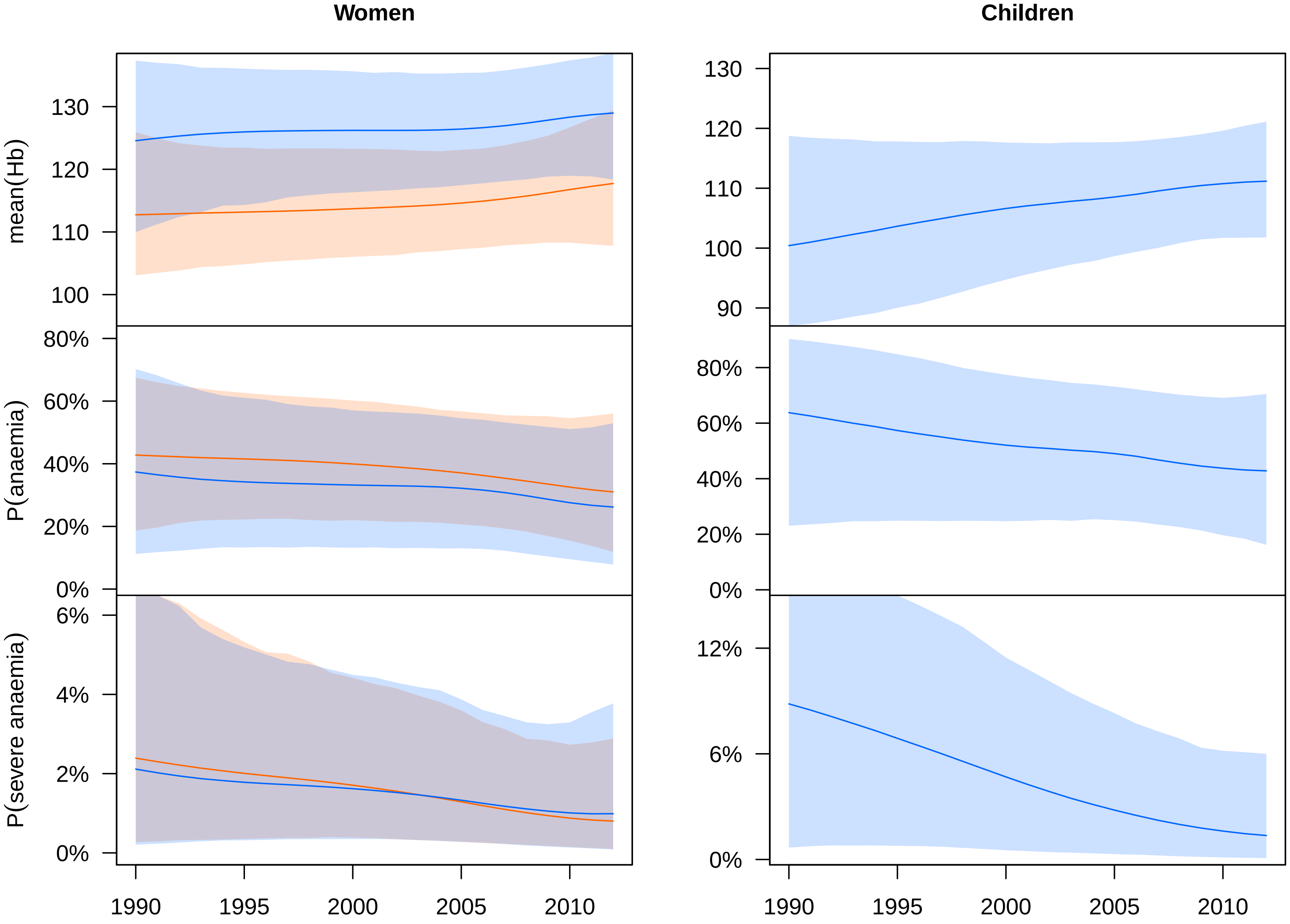

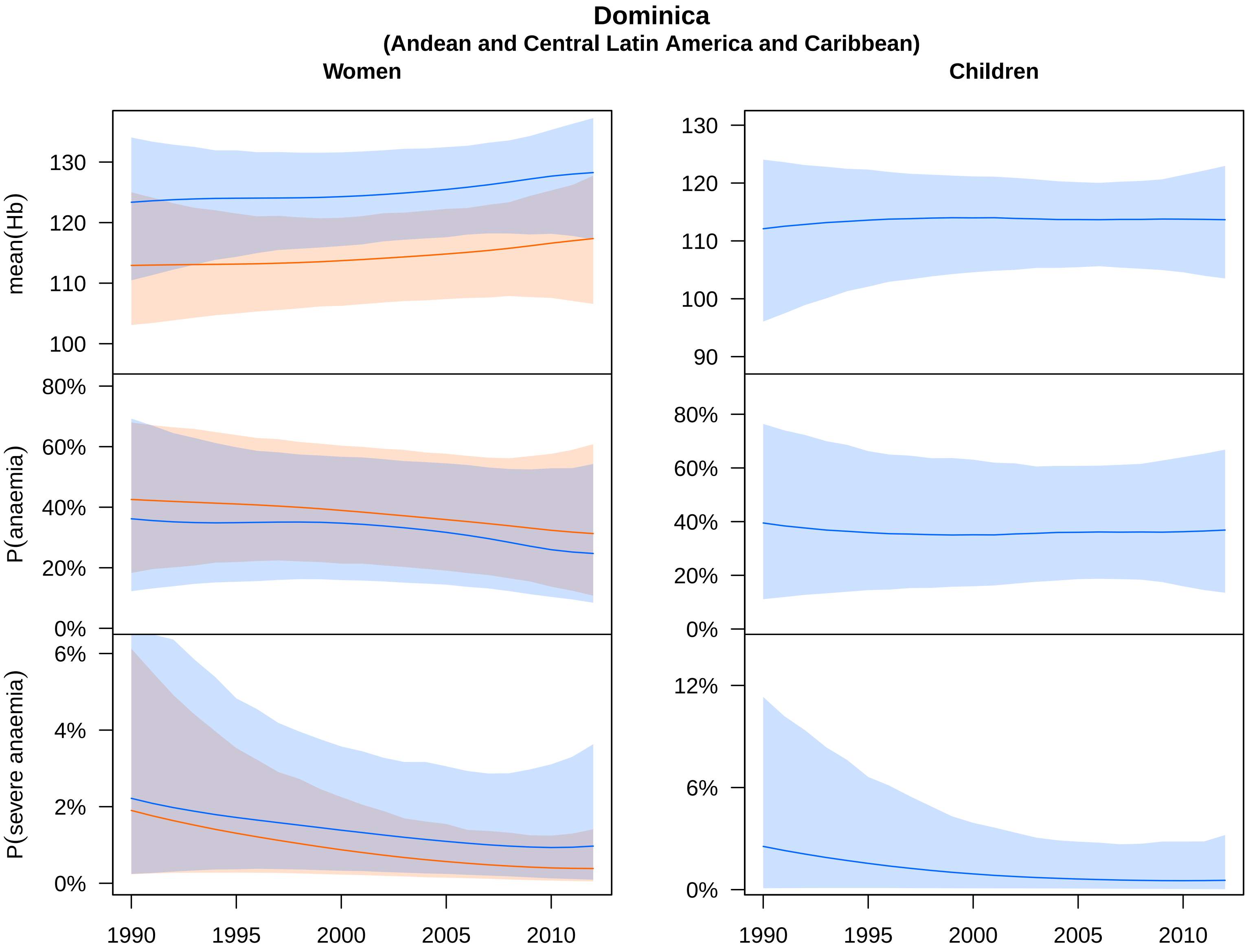

Dominica
(Andean and Central Latin America and Caribbean)
Women
Children
mean(Hb)
P(anaemia)
P(severe anaemia)
130
120
110
100
90
80%
60%
40%
20%
0%
6%
4%
2%
12%
1990
1995
2000
2005
2010

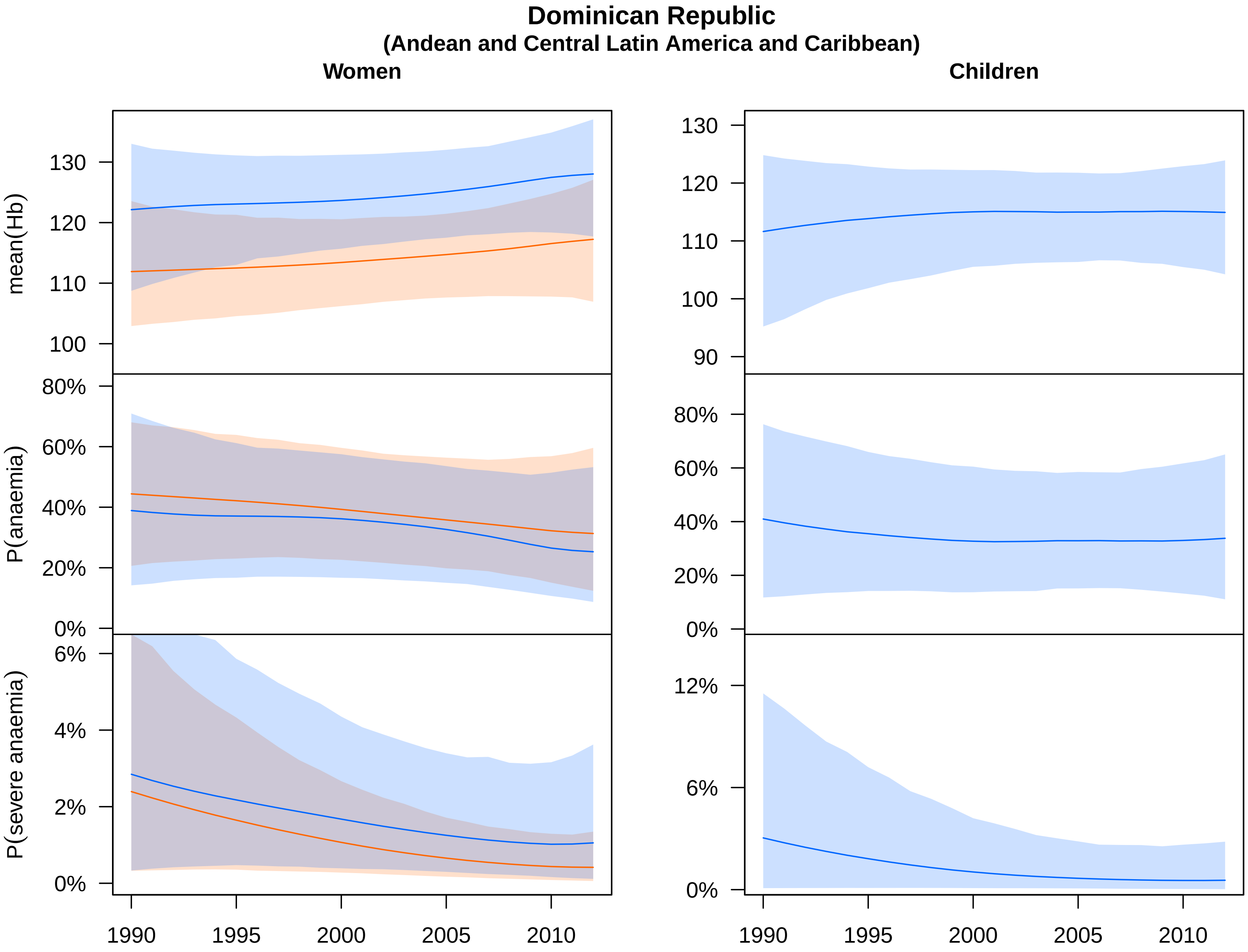
Dominican Republic
(Andean and Central Latin America and Caribbean)
Women
Children
mean(Hb)
P(anaemia)
P(severe anaemia)
130
120
110
100
90
80%
60%
40%
20%
0%
6%
4%
2%
12%
1990
1995
2000
2005
2010

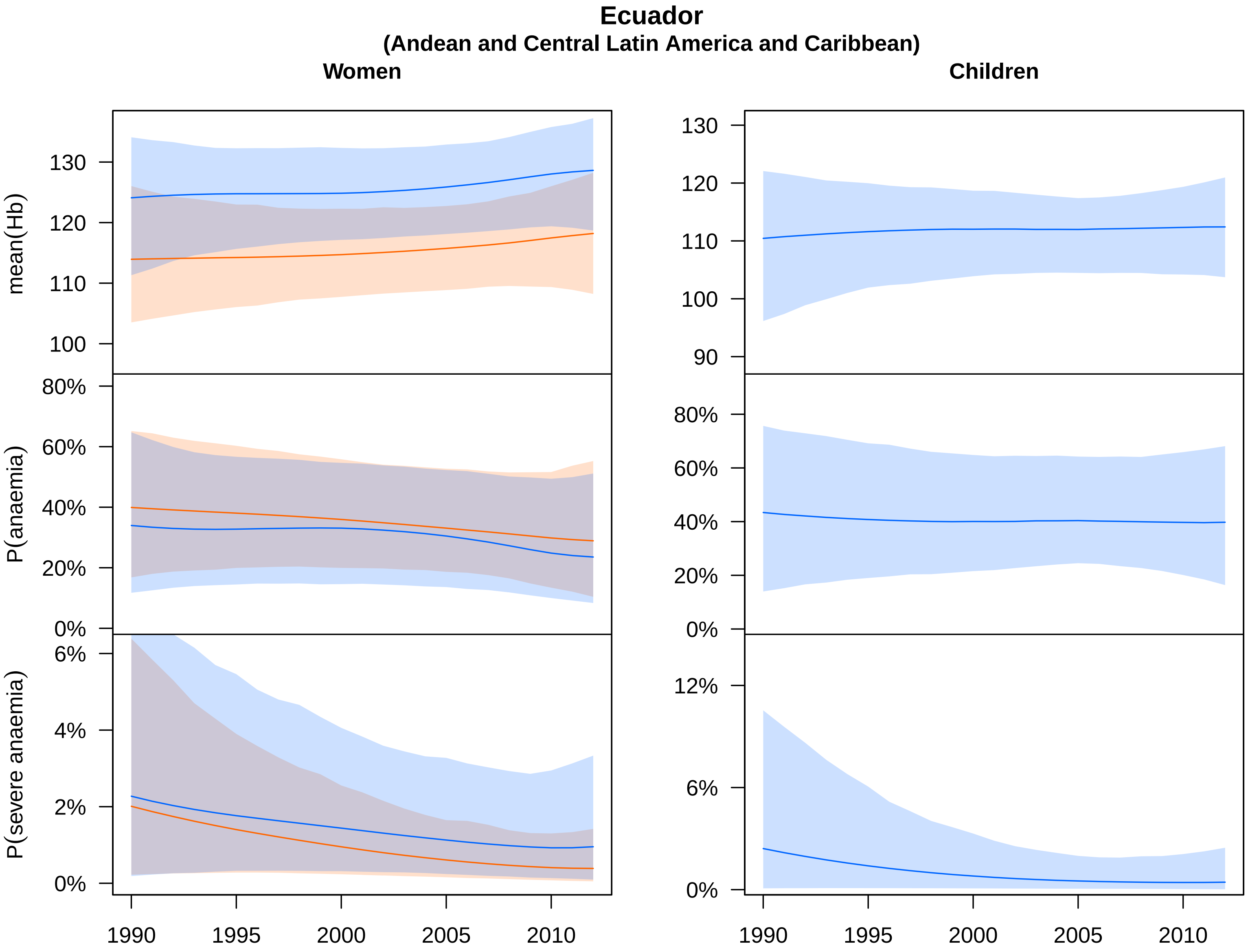
Ecuador
(Andean and Central Latin America and Caribbean)
Women
Children
mean(Hb)
P(anaemia)
P(severe anaemia)
130
120
110
100
90
80%
60%
40%
20%
0%
6%
4%
2%
12%
1990
1995
2000
2005
2010

**Egypt**
**(Central Asia, Middle East, and North Africa)**

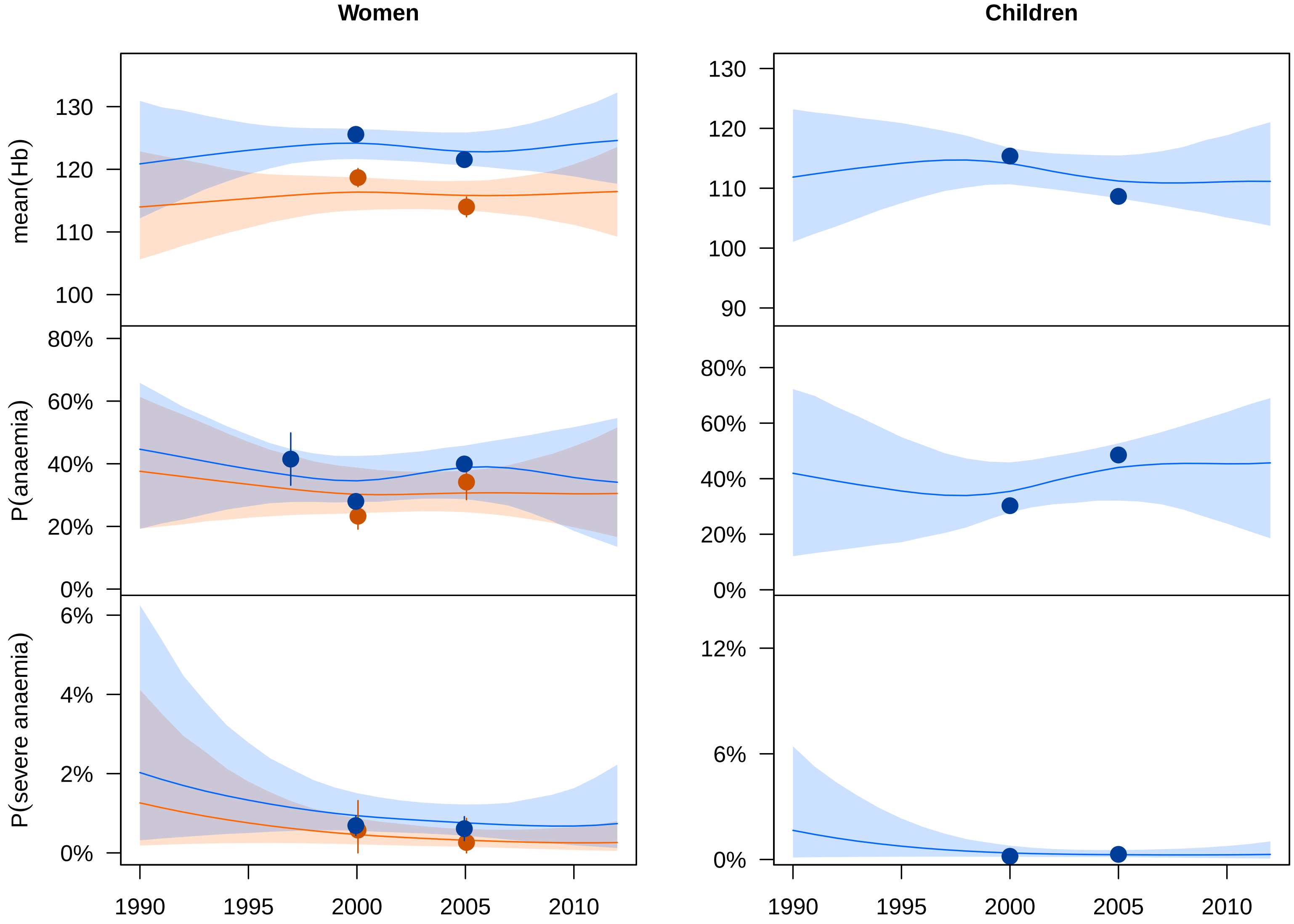

# El Salvador

## (Andean and Central Latin America and Caribbean)

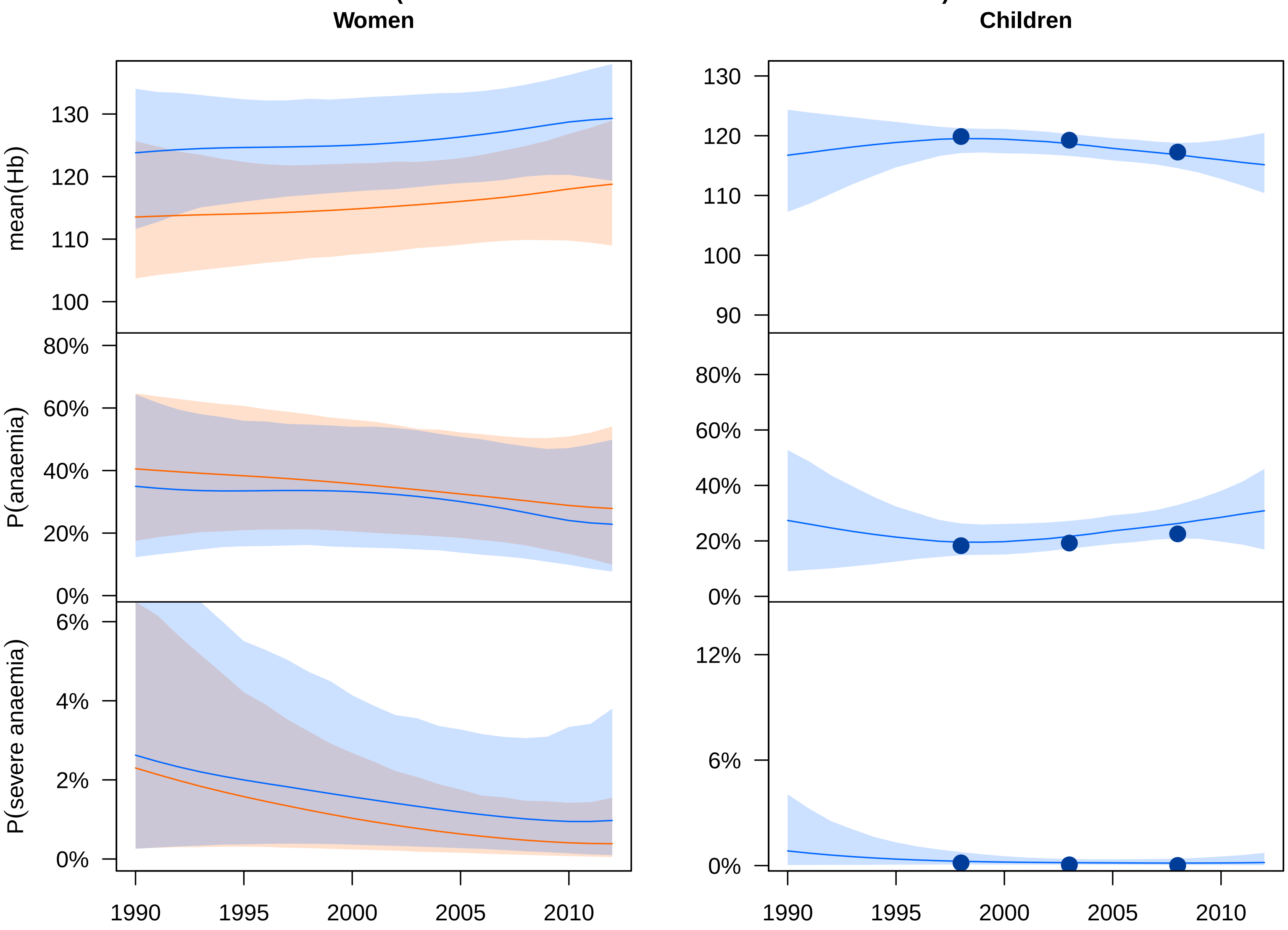

# Equatorial Guinea
**(West and Central Africa)**

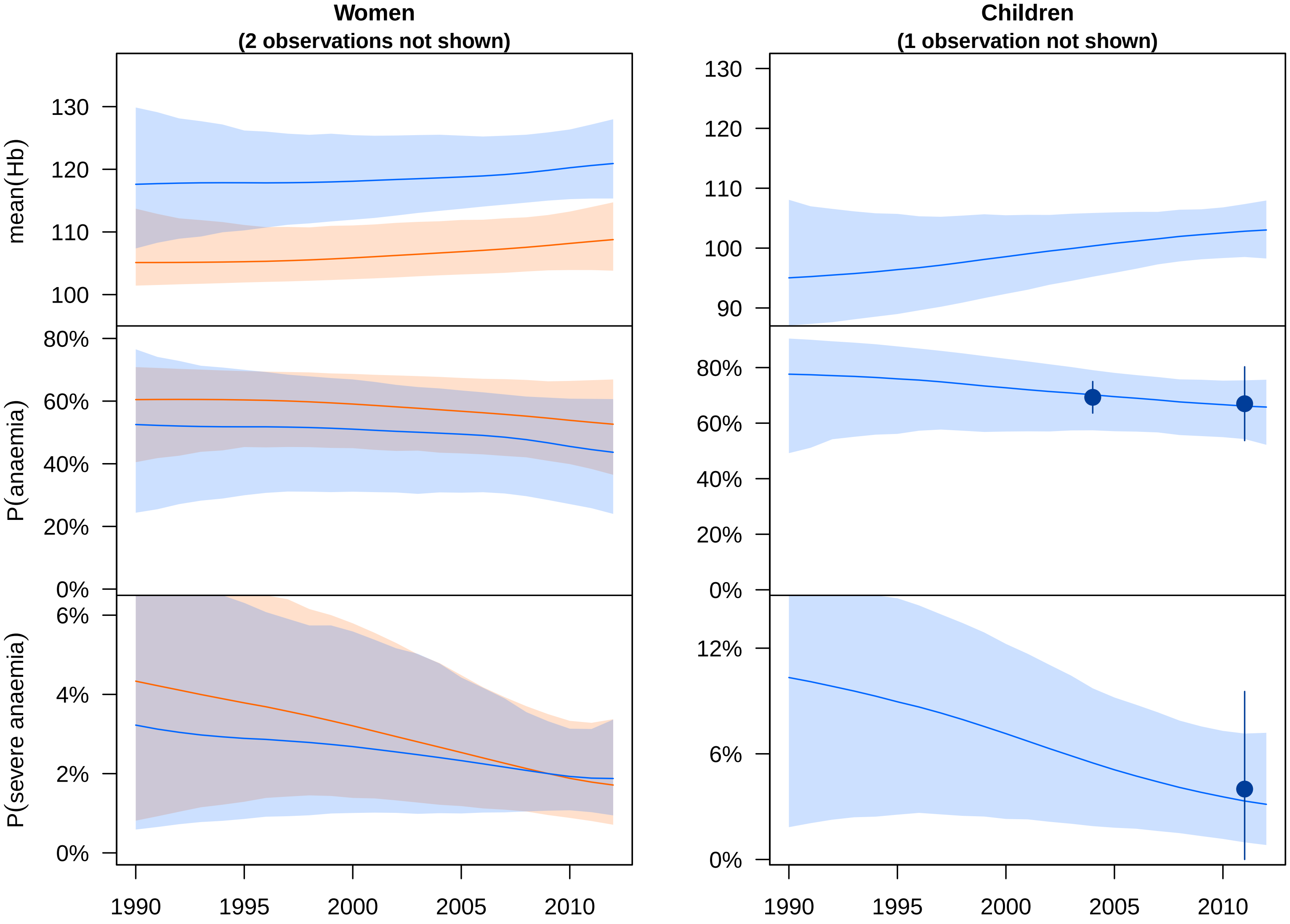

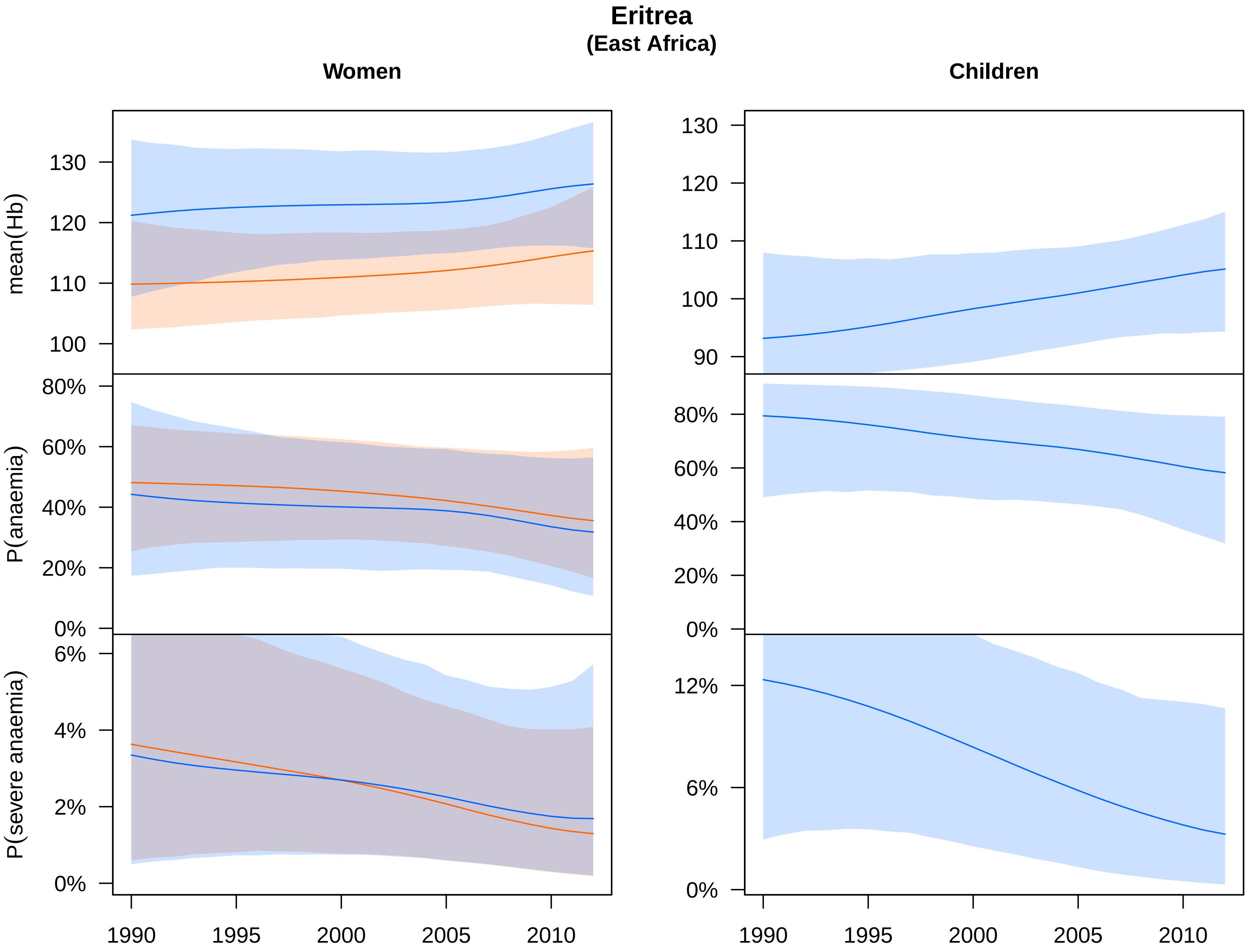

Eritrea
(East Africa)
Women
Children
mean(Hb)
P(anaemia)
P(severe anaemia)
130
120
110
100
90
80%
60%
40%
20%
0%
6%
4%
2%
12%
1990
1995
2000
2005
2010

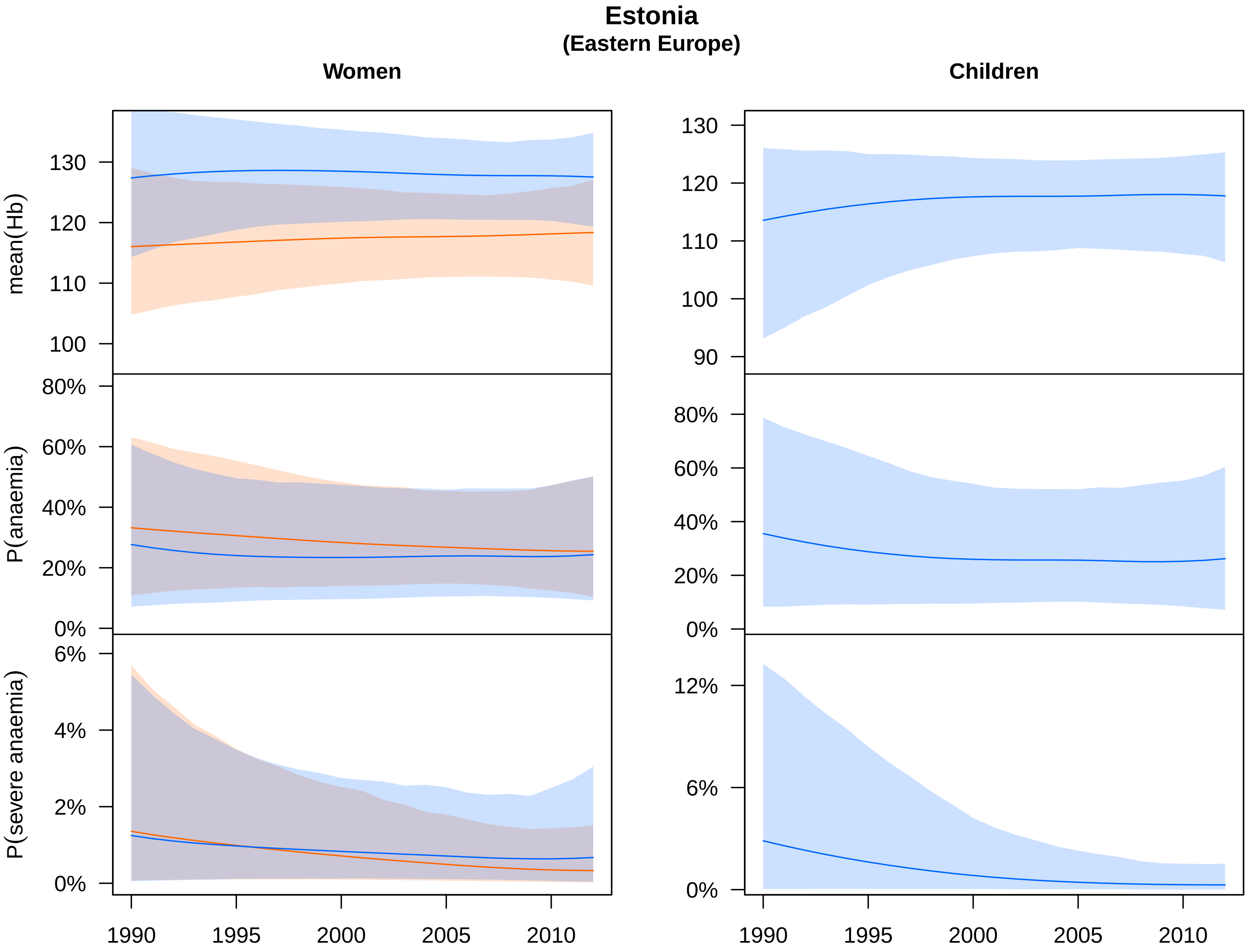

Estonia
(Eastern Europe)
Women
Children
mean(Hb)
130
120
110
100
90
P(anaemia)
80%
60%
40%
20%
0%
P(severe anaemia)
6%
4%
2%
0%
12%
6%
0%
1990
1995
2000
2005
2010

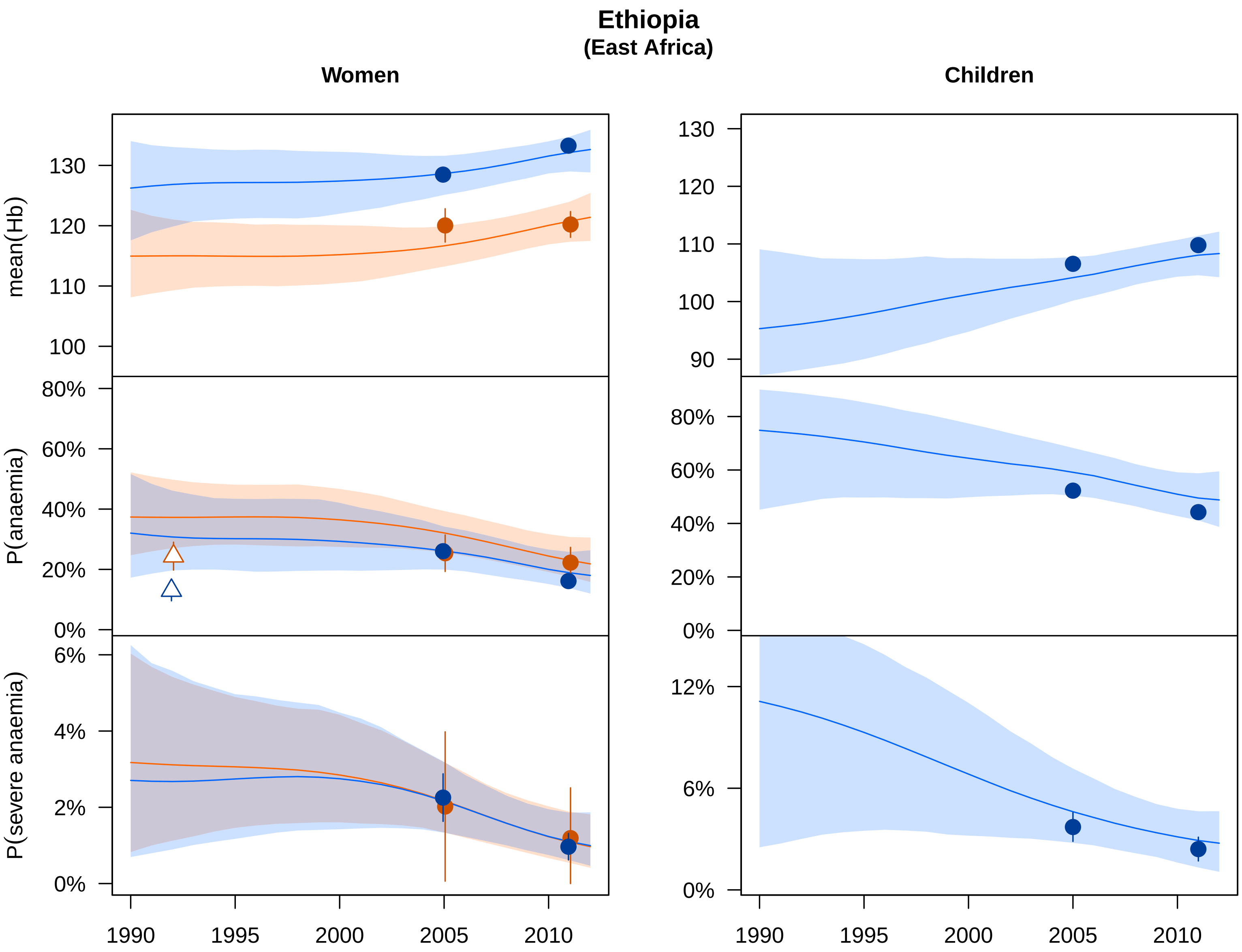
Ethiopia
(East Africa)
Women
Children
mean(Hb)
P(anaemia)
P(severe anaemia)
130
120
110
100
90
80%
60%
40%
20%
0%
6%
4%
2%
12%
1990
1995
2000
2005
2010

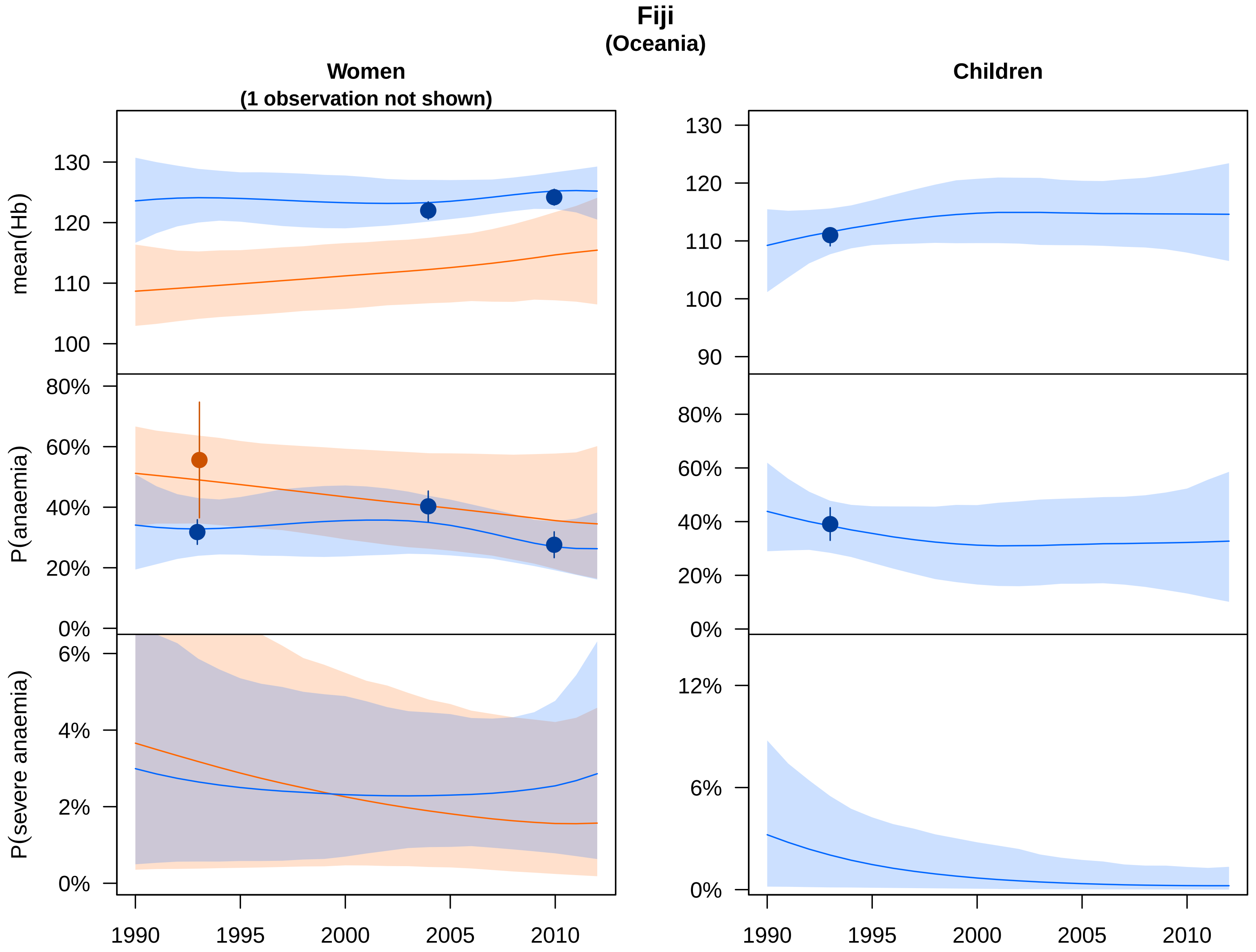
Fiji
(Oceania)
Women
(1 observation not shown)
Children
mean(Hb)
P(anaemia)
P(severe anaemia)
130
120
110
100
90
80%
60%
40%
20%
0%
6%
4%
2%
12%
1990
1995
2000
2005
2010

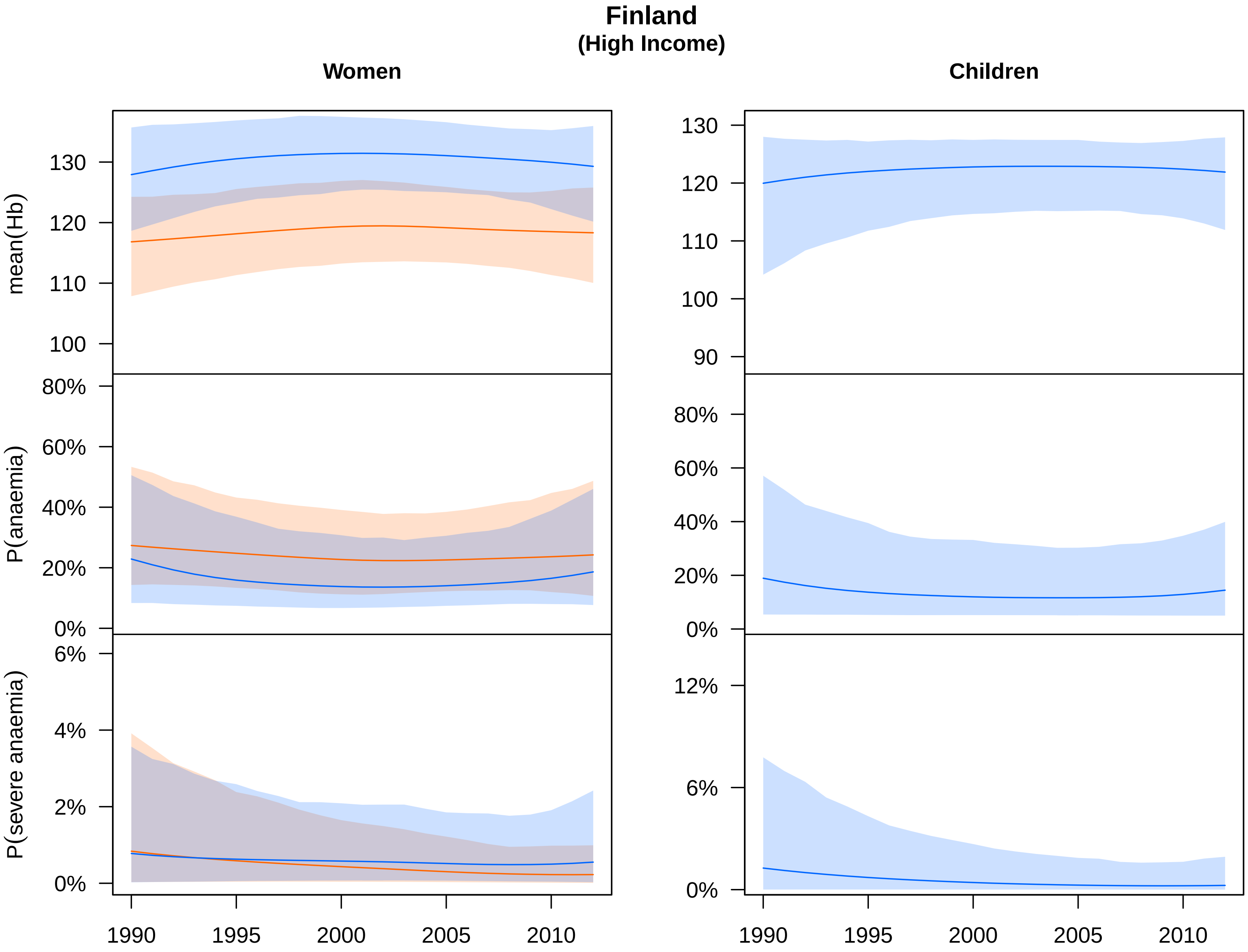
Finland
(High Income)
Women
Children
mean(Hb)
P(anaemia)
P(severe anaemia)
130
120
110
100
90
80%
60%
40%
20%
0%
6%
4%
2%
12%
6%
1990
1995
2000
2005
2010

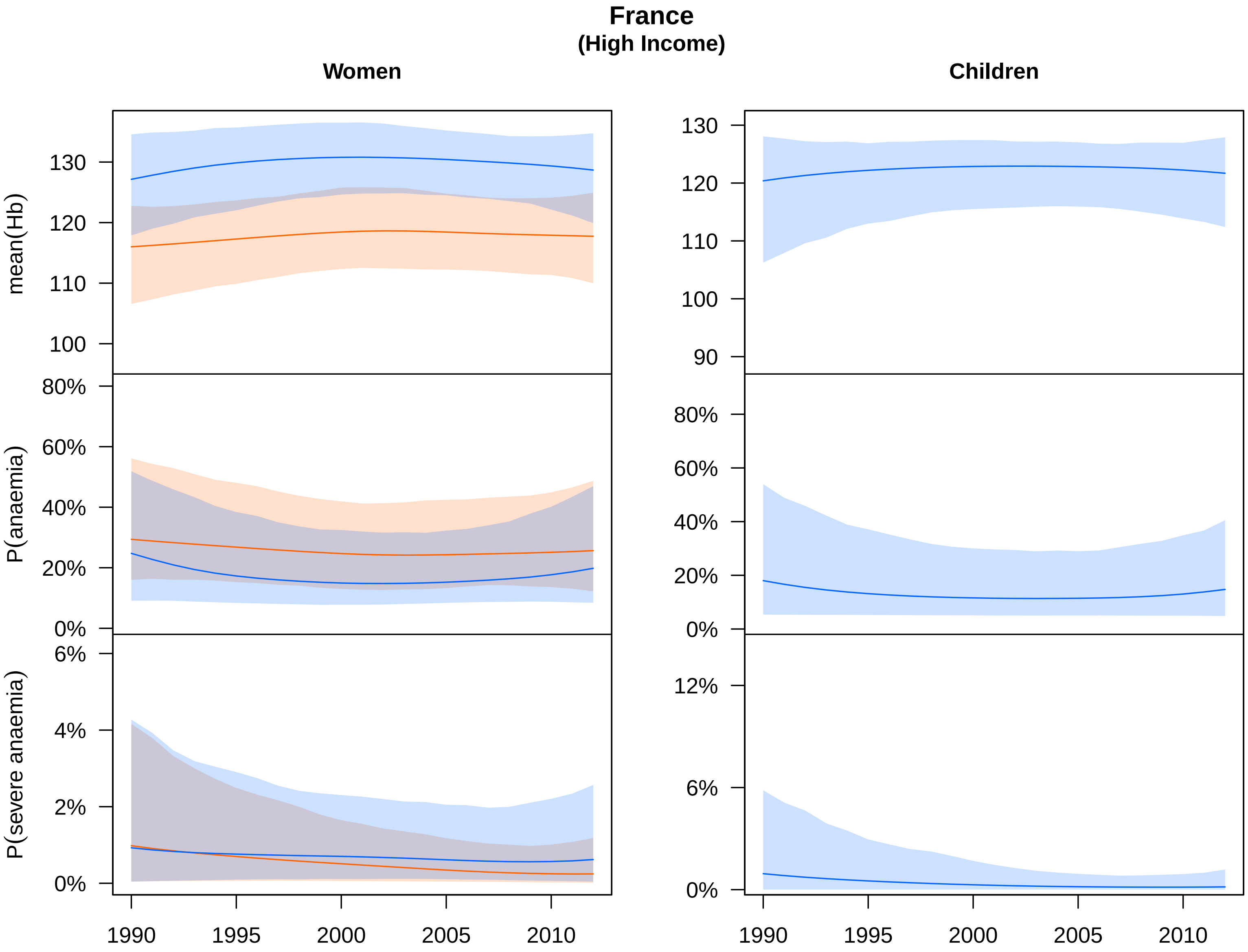
France
(High Income)
Women
Children
mean(Hb)
130
120
110
100
90
P(anaemia)
80%
60%
40%
20%
0%
P(severe anaemia)
6%
4%
2%
12%
1990
1995
2000
2005
2010

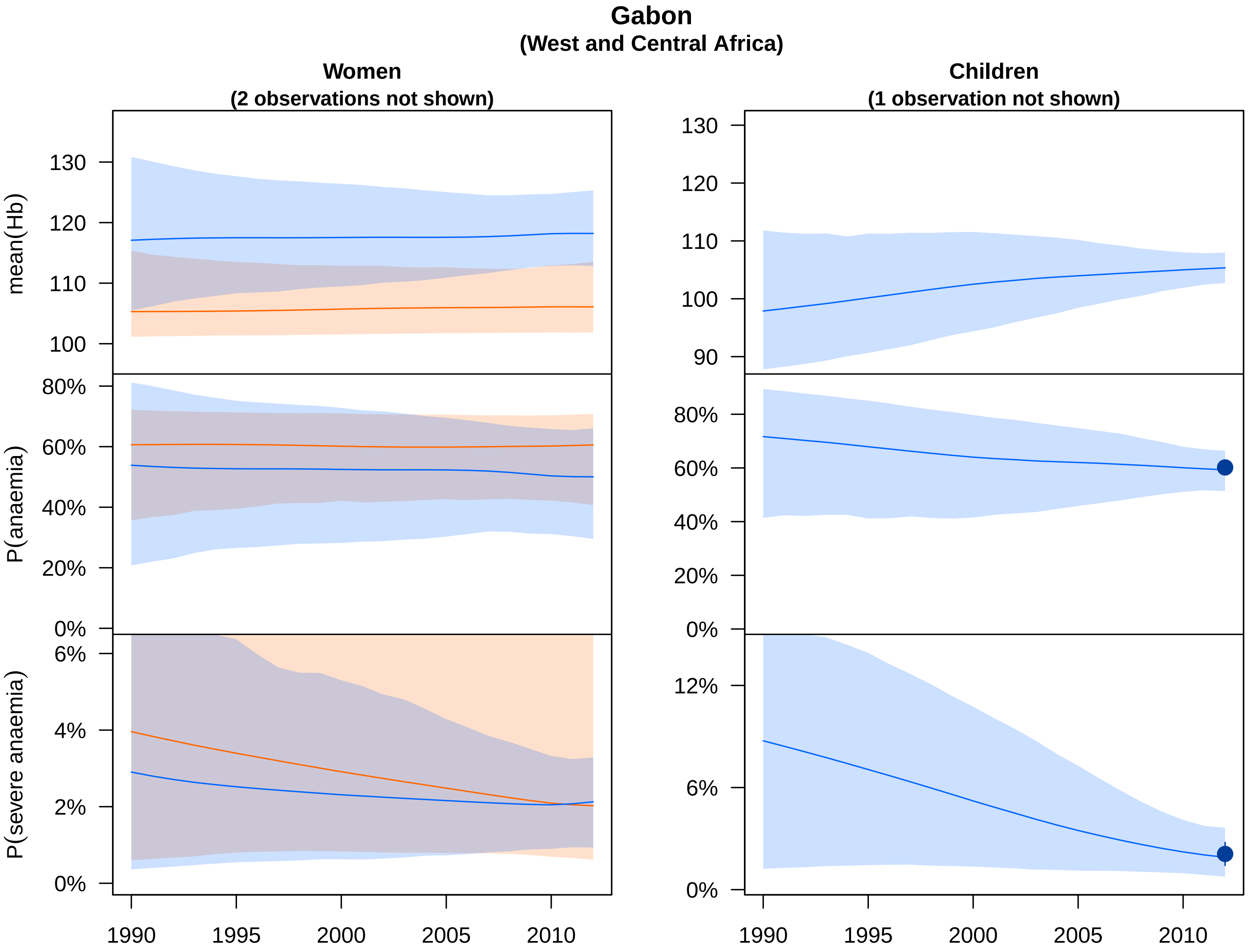
Gabon
(West and Central Africa)
Women
(2 observations not shown)
Children
(1 observation not shown)
mean(Hb)
P(anaemia)
P(severe anaemia)
130
120
110
100
90
80%
60%
40%
20%
0%
6%
4%
2%
12%
1990
1995
2000
2005
2010

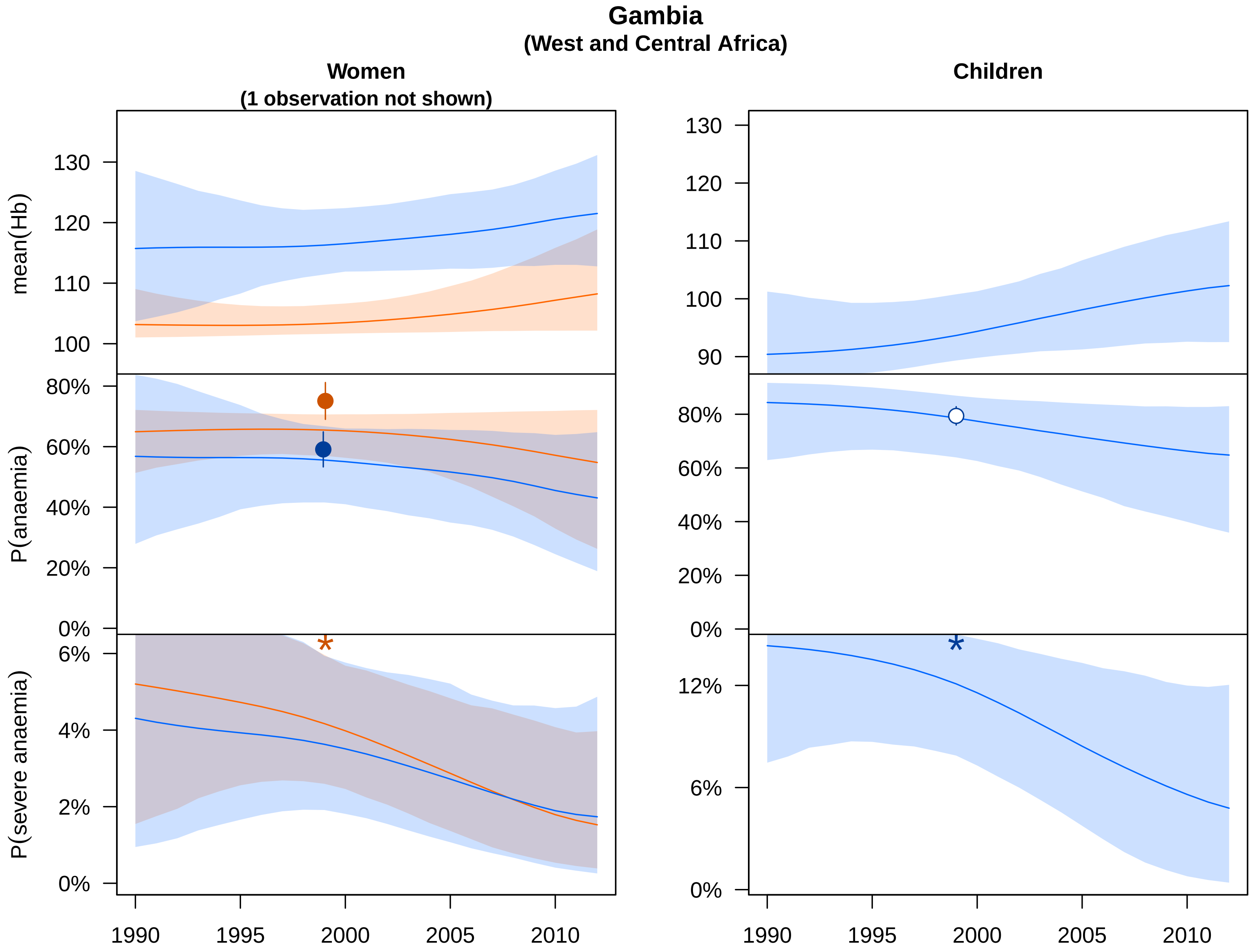

Gambia
(West and Central Africa)
Women
(1 observation not shown)
Children
mean(Hb)
P(anaemia)
P(severe anaemia)
130
120
110
100
90
80%
60%
40%
20%
0%
6%
4%
2%
12%
1990
1995
2000
2005
2010

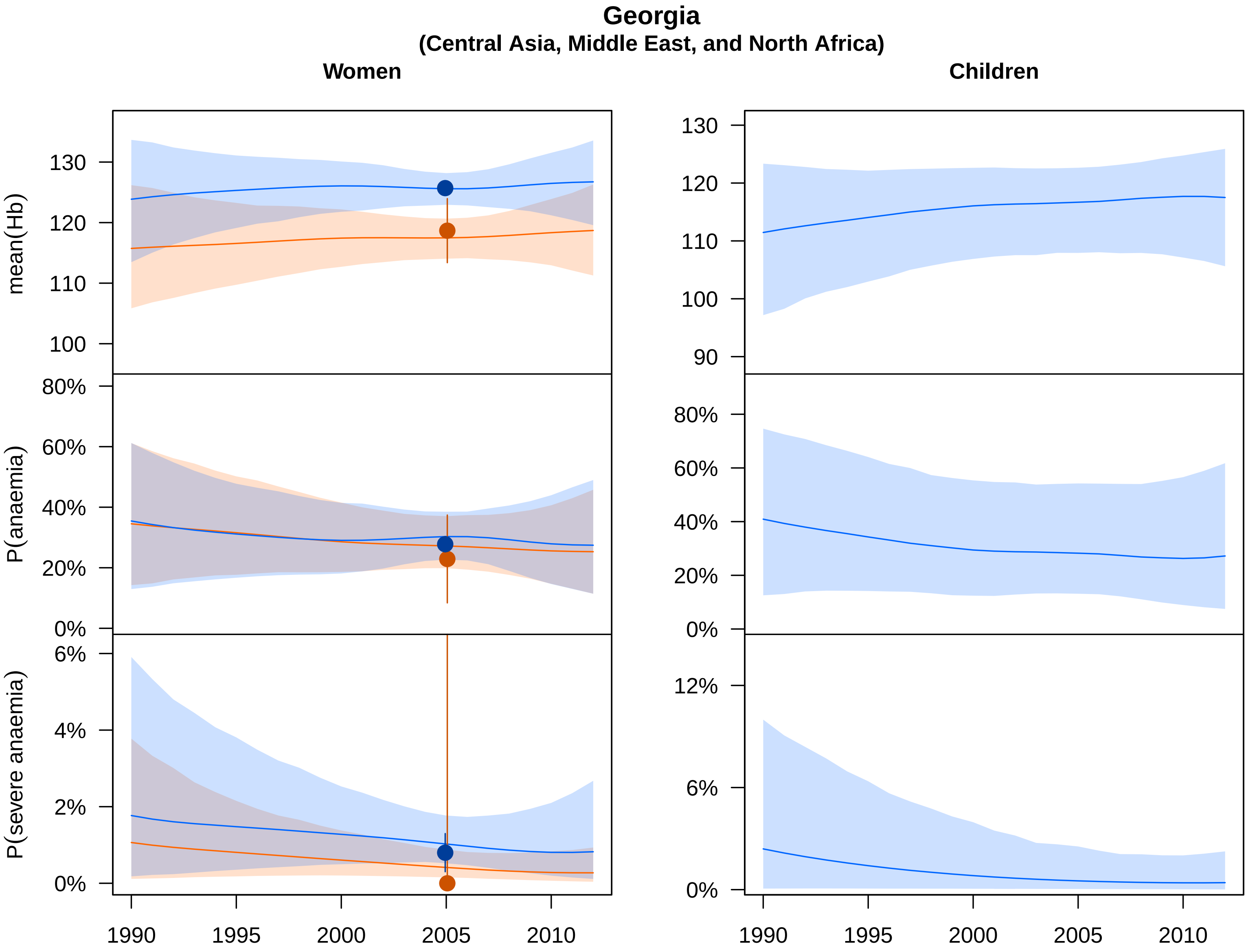
Georgia
(Central Asia, Middle East, and North Africa)
Women
Children
mean(Hb)
P(anaemia)
P(severe anaemia)
130
120
110
100
90
80%
60%
40%
20%
0%
6%
4%
2%
12%
1990
1995
2000
2005
2010

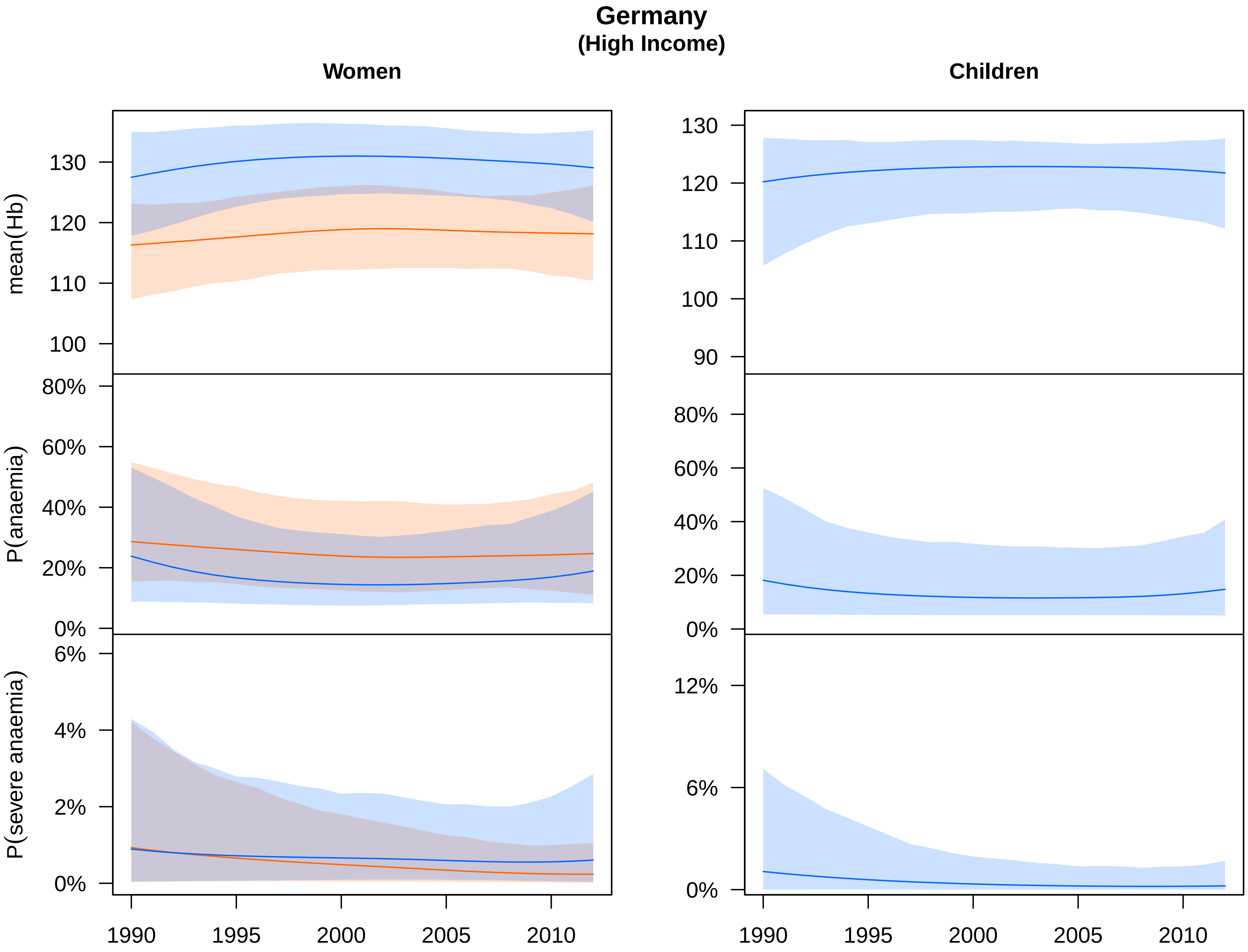
Germany
(High Income)
Women
Children
mean(Hb)
P(anaemia)
P(severe anaemia)
130
120
110
100
90
80%
60%
40%
20%
0%
6%
4%
2%
12%
1990
1995
2000
2005
2010

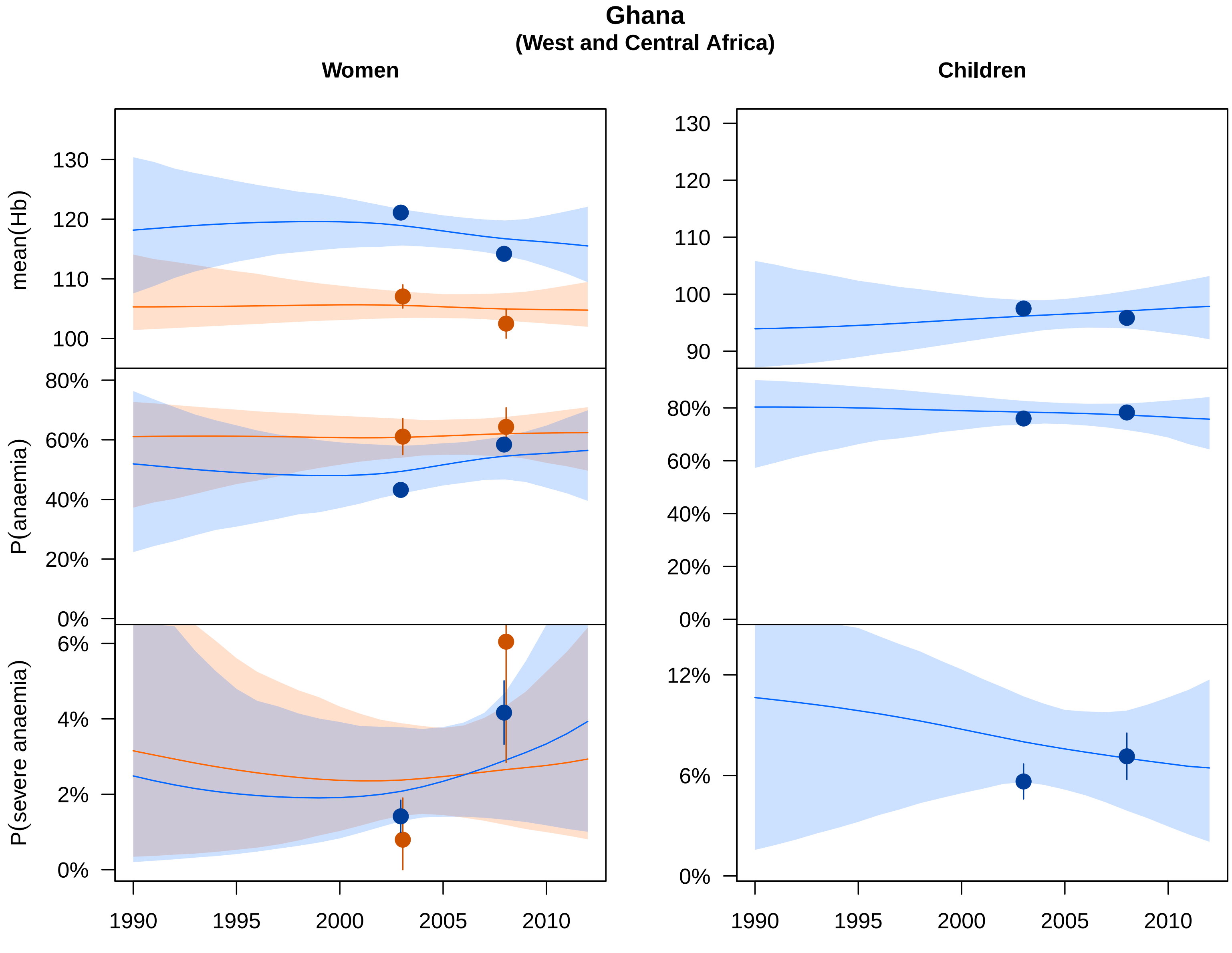

Ghana
(West and Central Africa)
Women
Children
mean(Hb)
P(anaemia)
P(severe anaemia)
130
120
110
100
90
80%
60%
40%
20%
0%
6%
4%
2%
12%
1990
1995
2000
2005
2010

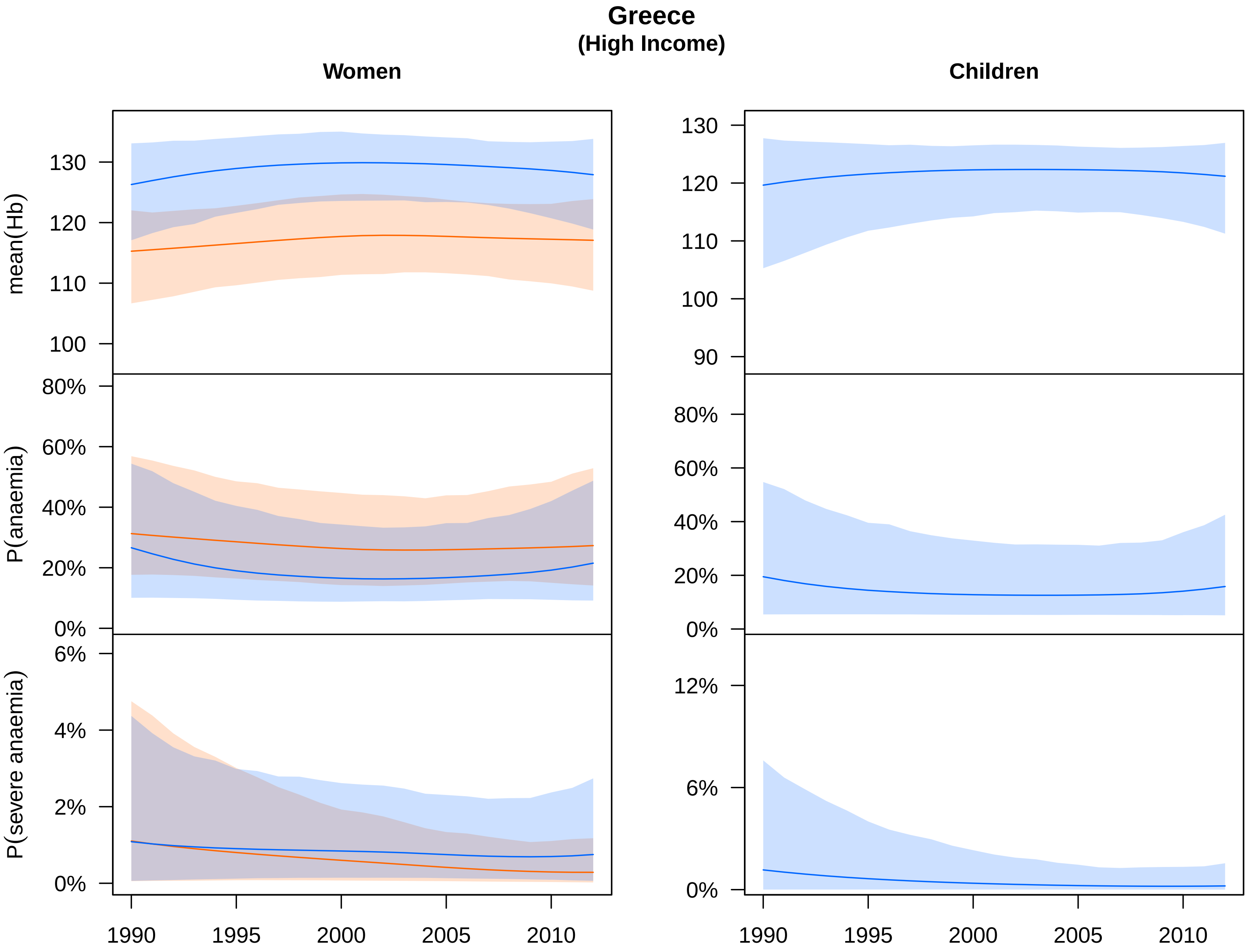
Greece
(High Income)
Women
Children
mean(Hb)
P(anaemia)
P(severe anaemia)
130
120
110
100
90
80%
60%
40%
20%
0%
6%
4%
2%
12%
1990
1995
2000
2005
2010

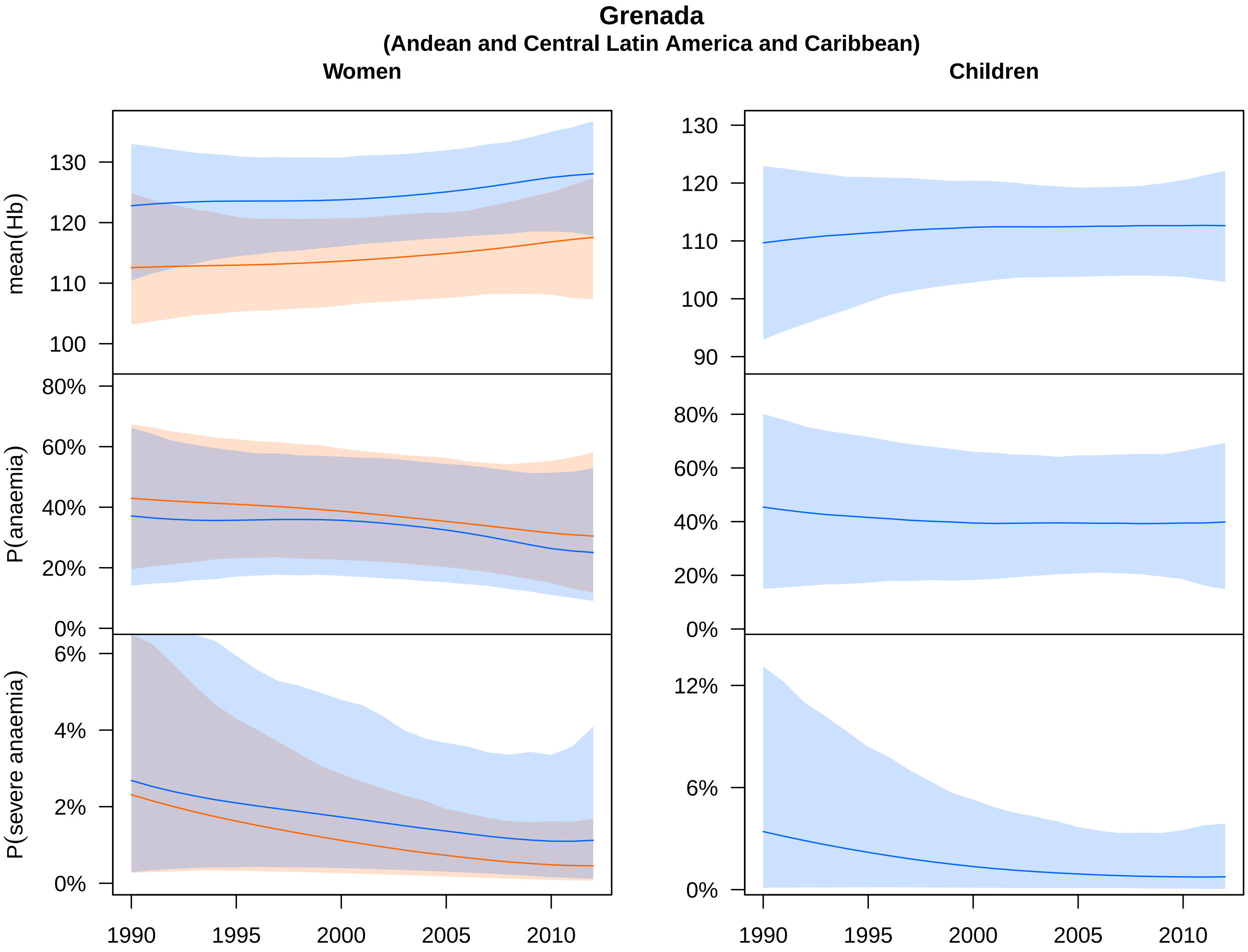
Grenada
(Andean and Central Latin America and Caribbean)
Women
Children
mean(Hb)
P(anaemia)
P(severe anaemia)
130
120
110
100
90
80%
60%
40%
20%
0%
6%
4%
2%
12%
1990
1995
2000
2005
2010

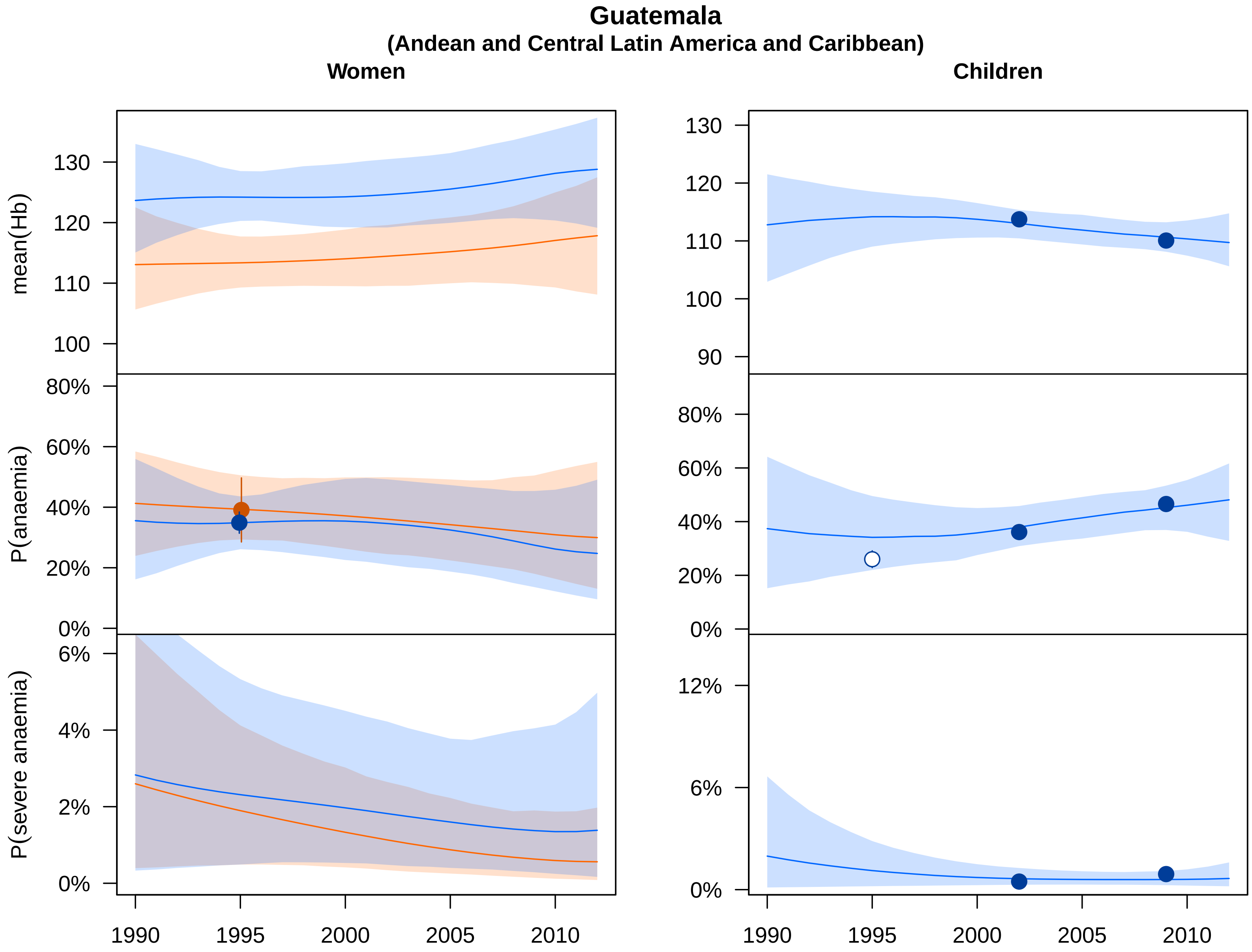
Guatemala
(Andean and Central Latin America and Caribbean)
Women
Children
mean(Hb)
P(anaemia)
P(severe anaemia)
130
120
110
100
90
80%
60%
40%
20%
0%
6%
4%
2%
12%
1990
1995
2000
2005
2010

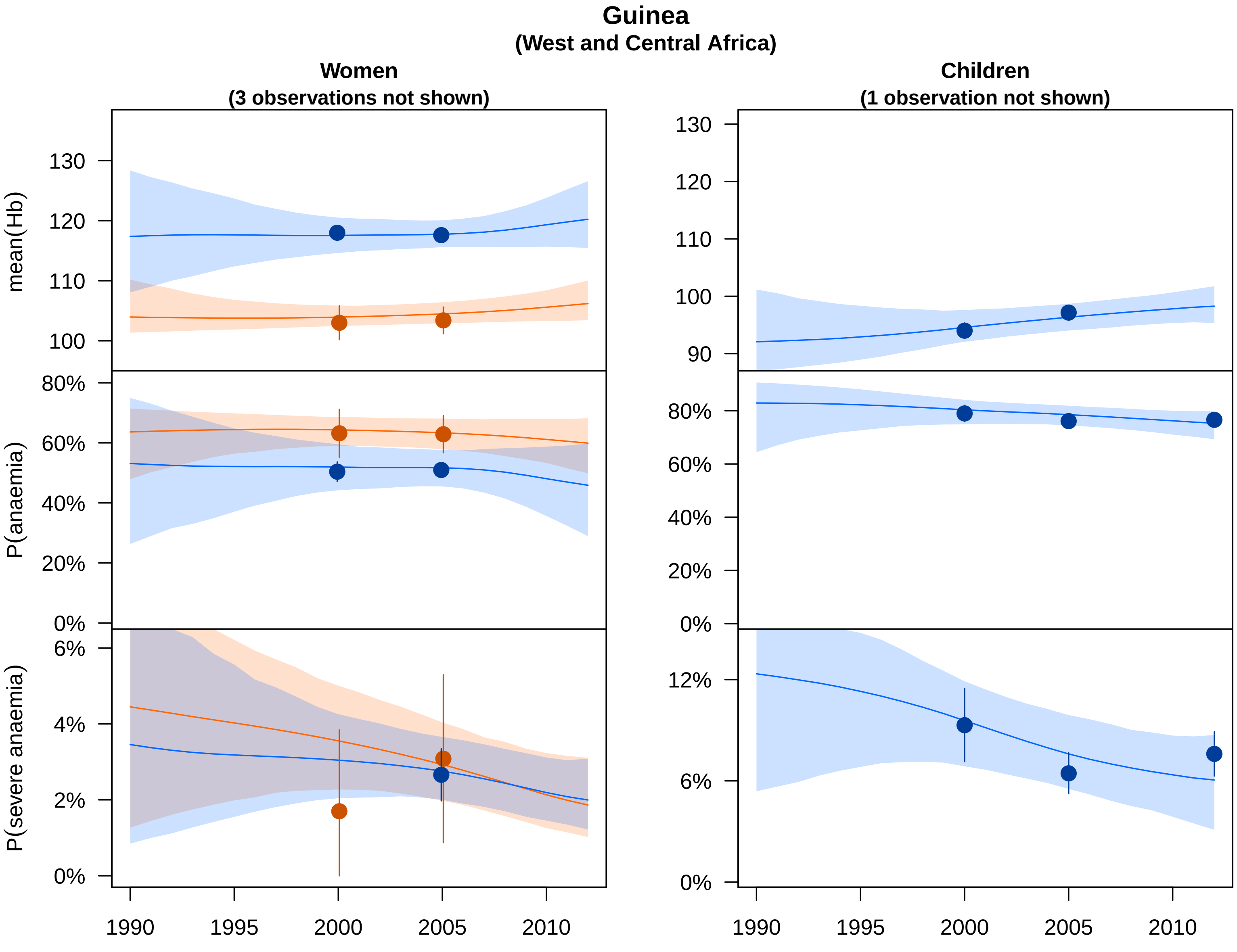

Guinea
(West and Central Africa)
Women
(3 observations not shown)
Children
(1 observation not shown)
mean(Hb)
P(anaemia)
P(severe anaemia)
130
120
110
100
90
80%
60%
40%
20%
0%
6%
4%
2%
12%
1990
1995
2000
2005
2010

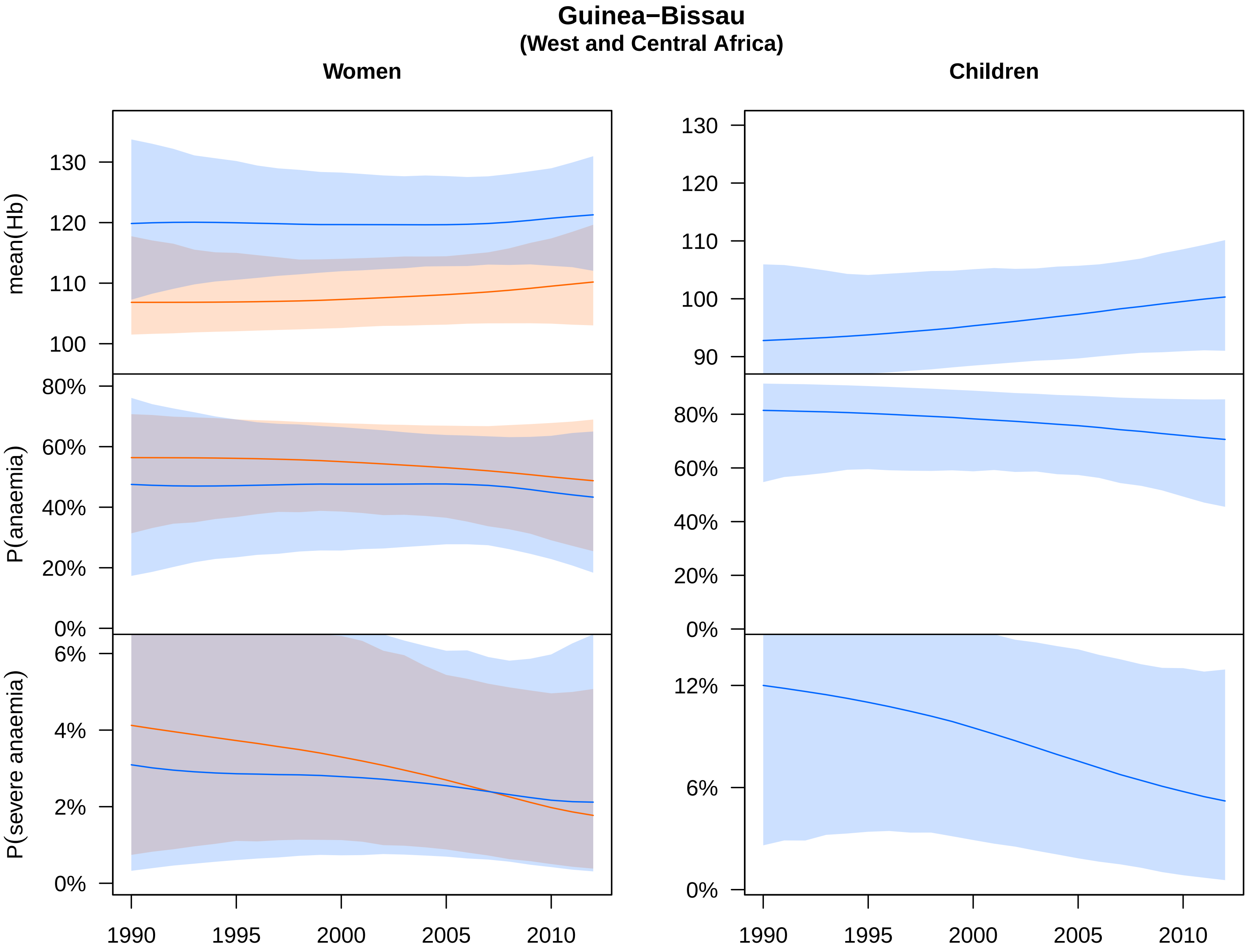
Guinea−Bissau
(West and Central Africa)
Women
Children
mean(Hb)
P(anaemia)
P(severe anaemia)
130
120
110
100
90
80%
60%
40%
20%
0%
6%
4%
2%
12%
1990
1995
2000
2005
2010

**Guyana**
**(Andean and Central Latin America and Caribbean)**

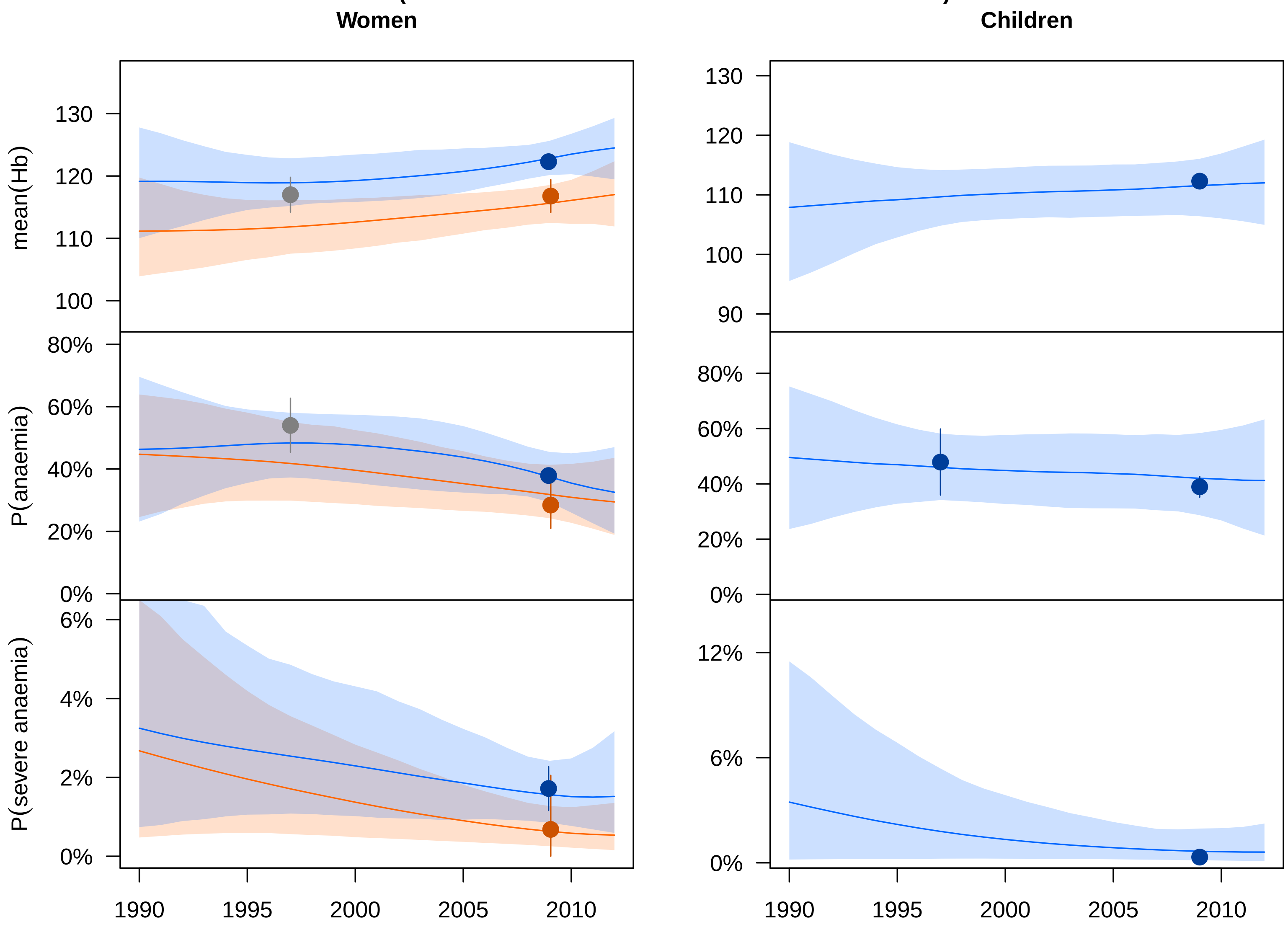

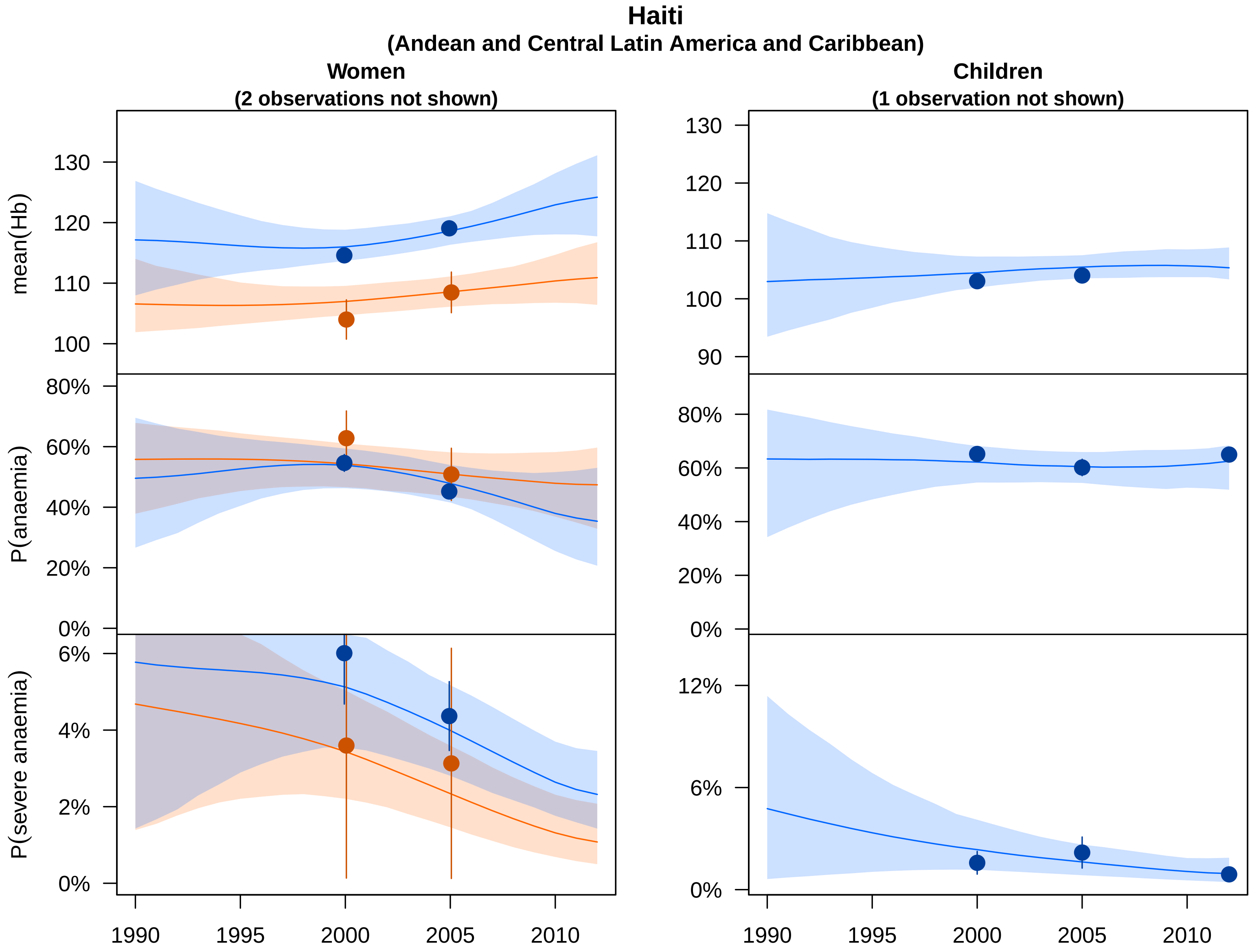
Haiti
(Andean and Central Latin America and Caribbean)
Women
(2 observations not shown)
Children
(1 observation not shown)
mean(Hb)
P(anaemia)
P(severe anaemia)
130
120
110
100
90
80%
60%
40%
20%
0%
6%
4%
2%
12%
1990
1995
2000
2005
2010

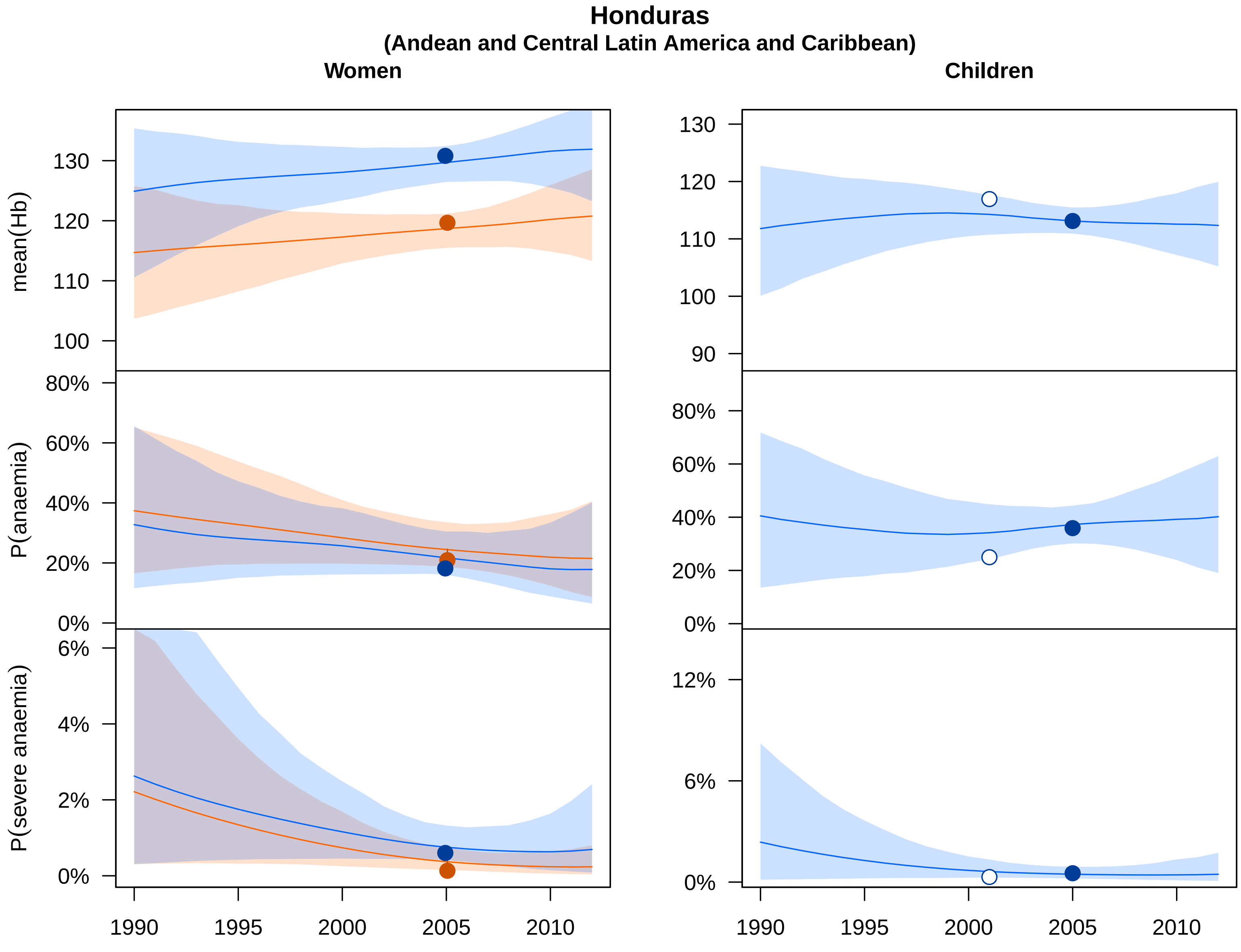
Honduras
(Andean and Central Latin America and Caribbean)
Women
Children
mean(Hb)
P(anaemia)
P(severe anaemia)
130
120
110
100
90
80%
60%
40%
20%
0%
6%
4%
2%
12%
1990
1995
2000
2005
2010

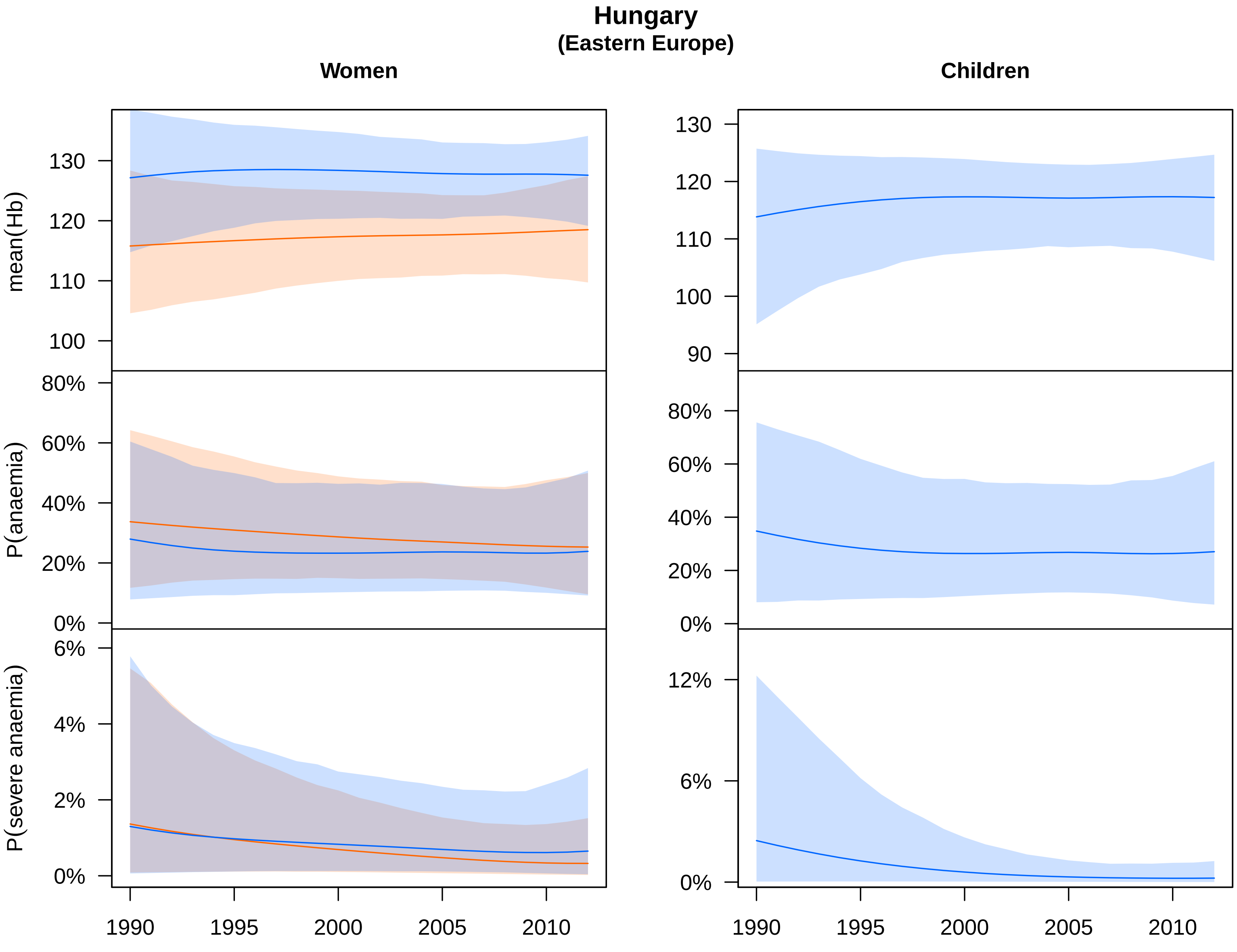
Hungary
(Eastern Europe)
Women
Children
mean(Hb)
P(anaemia)
P(severe anaemia)
130
120
110
100
90
80%
60%
40%
20%
0%
6%
4%
2%
12%
1990
1995
2000
2005
2010

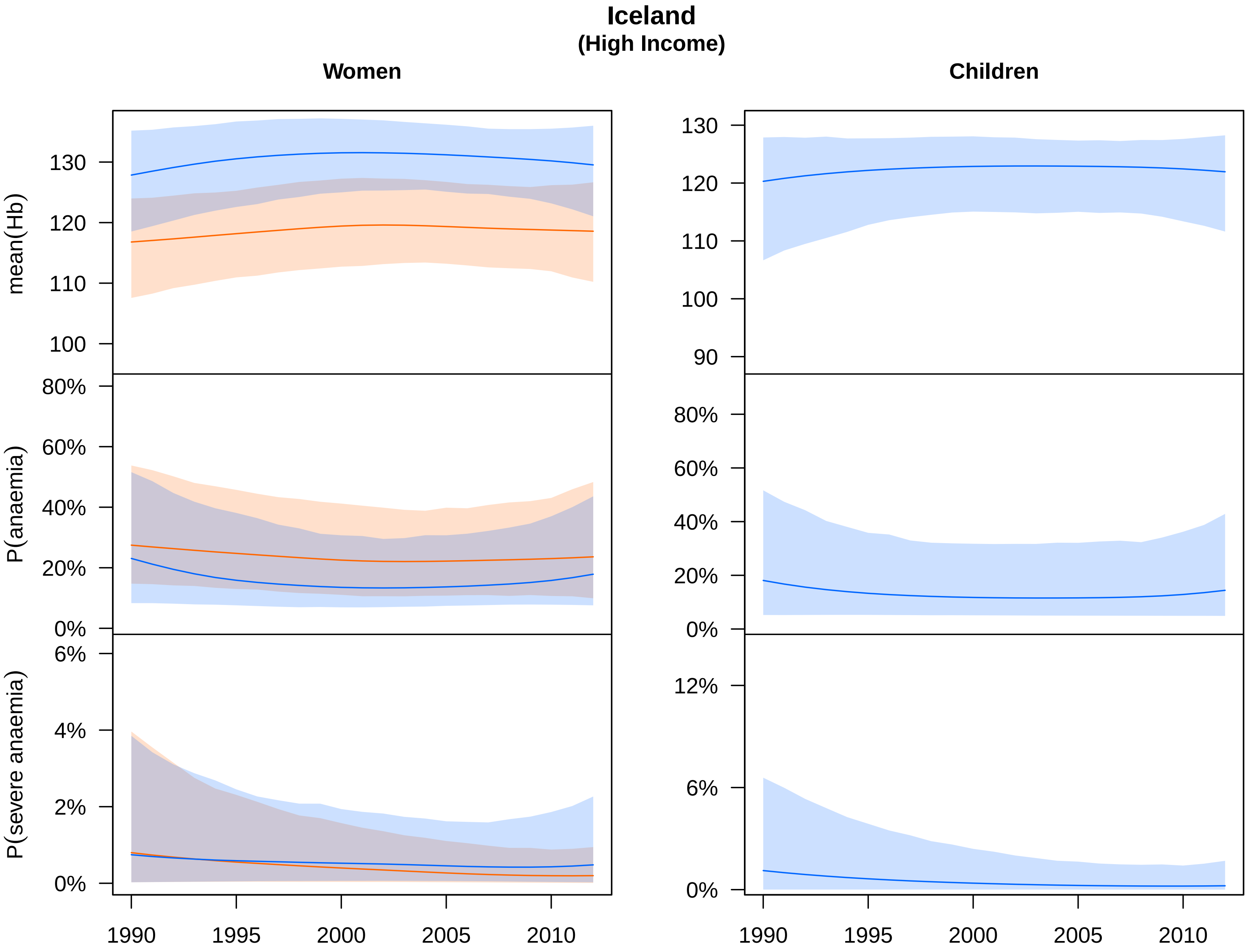

Iceland
(High Income)
Women
Children
mean(Hb)
P(anaemia)
P(severe anaemia)
130
120
110
100
90
80%
60%
40%
20%
0%
6%
4%
2%
12%
1990
1995
2000
2005
2010

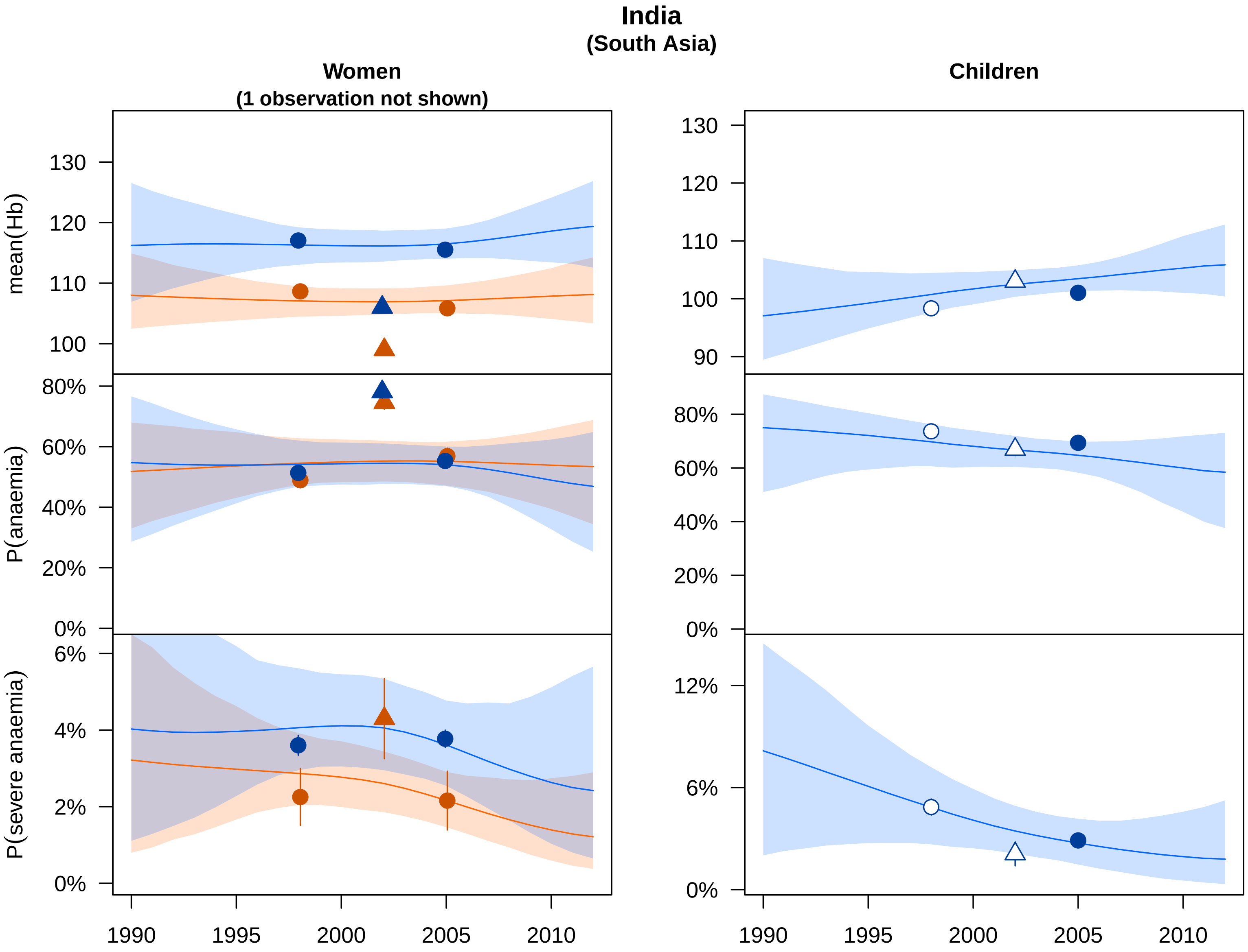
India
(South Asia)
Women
(1 observation not shown)
Children
mean(Hb)
P(anaemia)
P(severe anaemia)
130
120
110
100
90
80%
60%
40%
20%
0%
6%
4%
2%
12%
1990
1995
2000
2005
2010

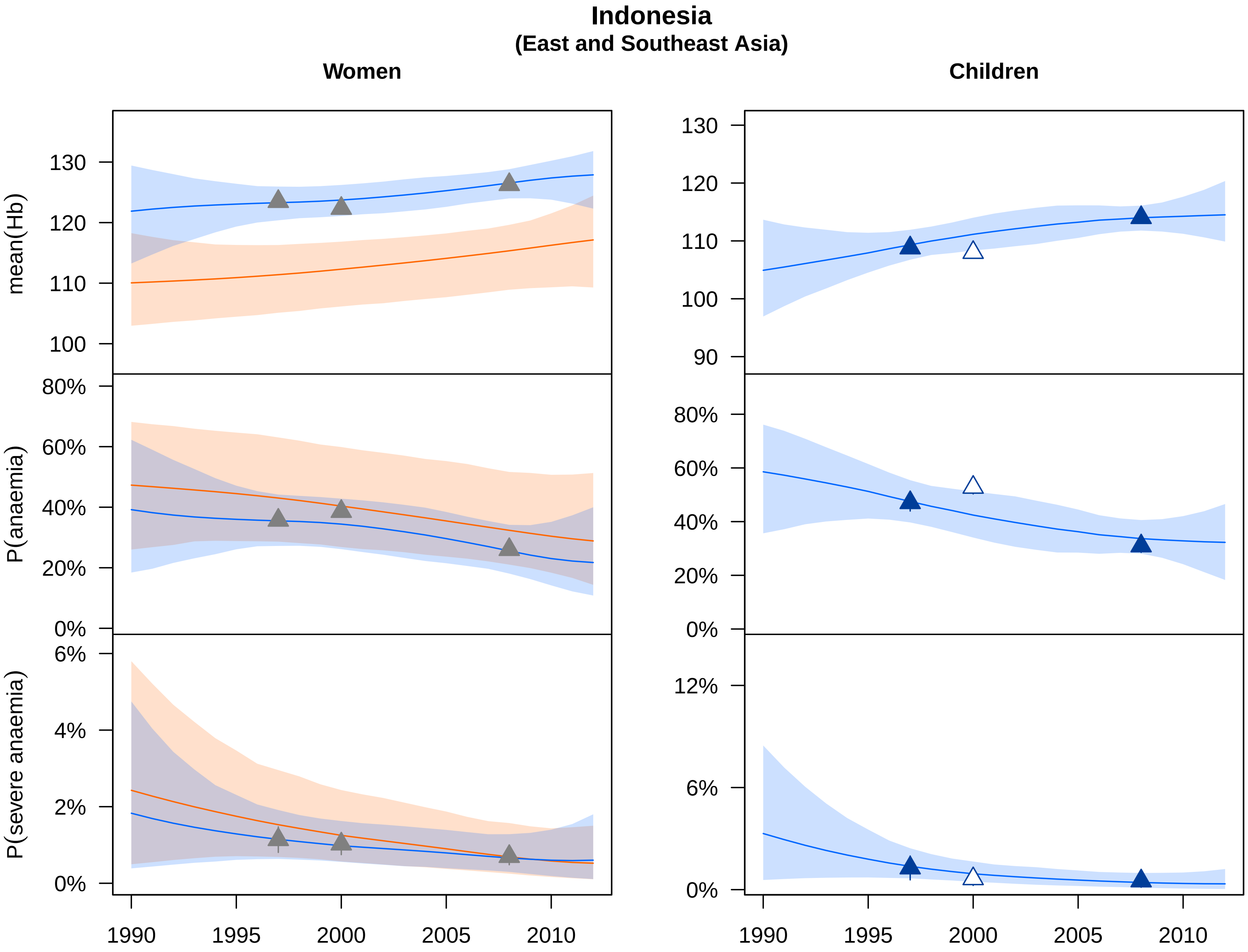

Indonesia
(East and Southeast Asia)
Women
Children
mean(Hb)
P(anaemia)
P(severe anaemia)
130
120
110
100
90
80%
60%
40%
20%
0%
6%
4%
2%
12%
1990
1995
2000
2005
2010

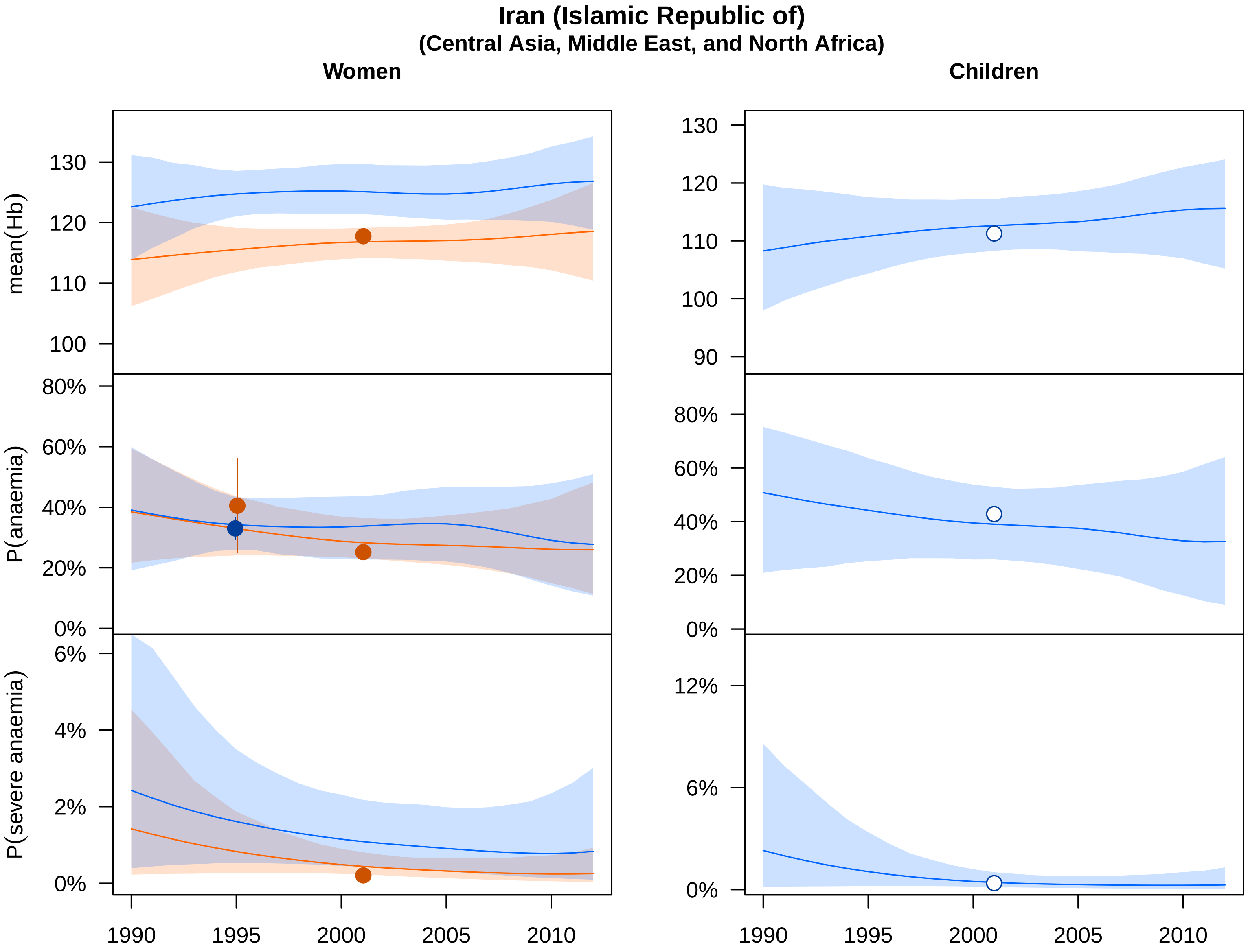

Iran (Islamic Republic of)
(Central Asia, Middle East, and North Africa)
Women
Children
mean(Hb)
P(anaemia)
P(severe anaemia)
130
120
110
100
90
80%
60%
40%
20%
0%
6%
4%
2%
12%
1990
1995
2000
2005
2010

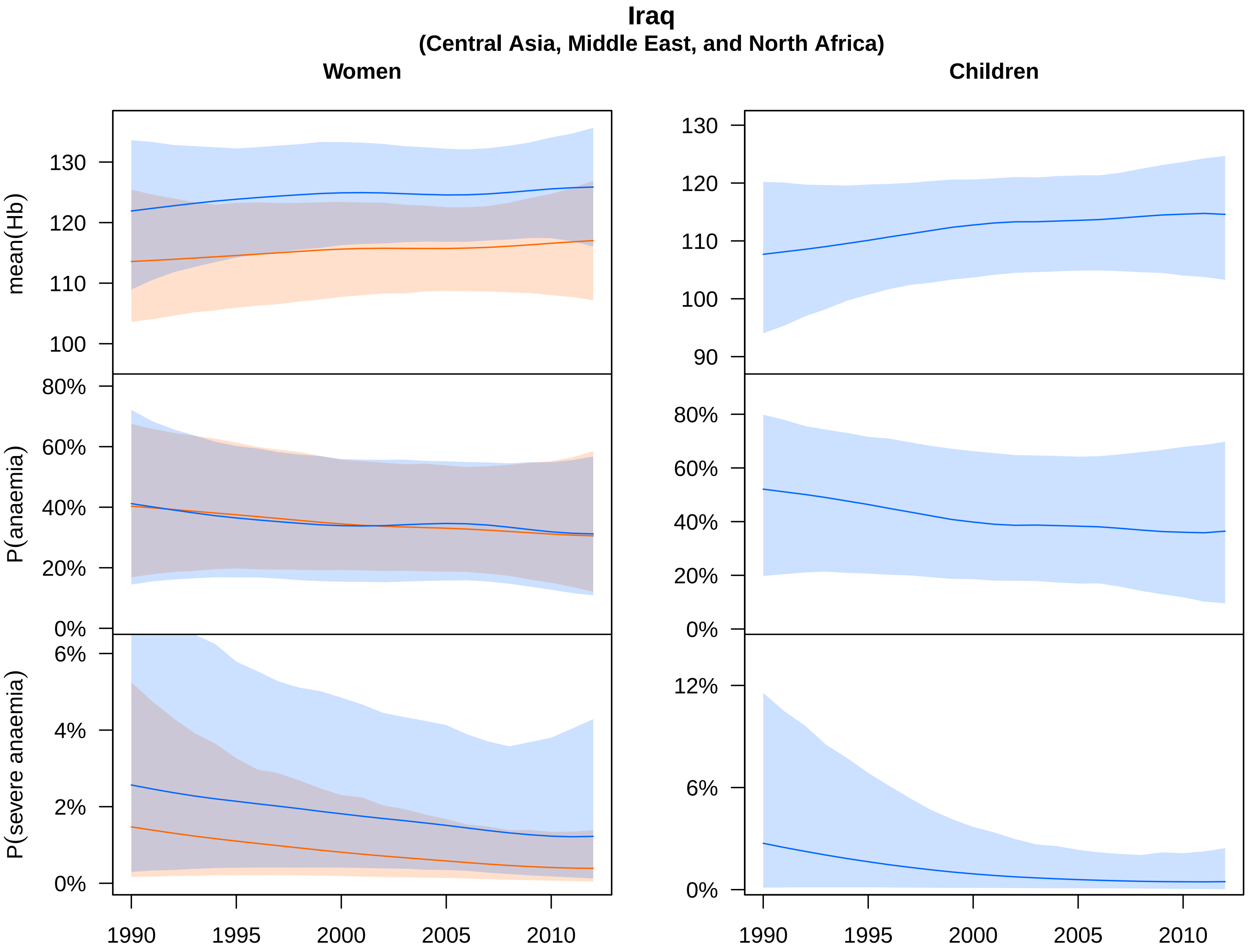
Iraq
(Central Asia, Middle East, and North Africa)
Women
Children
mean(Hb)
P(anaemia)
P(severe anaemia)
130
120
110
100
90
80%
60%
40%
20%
0%
6%
4%
2%
12%
1990
1995
2000
2005
2010

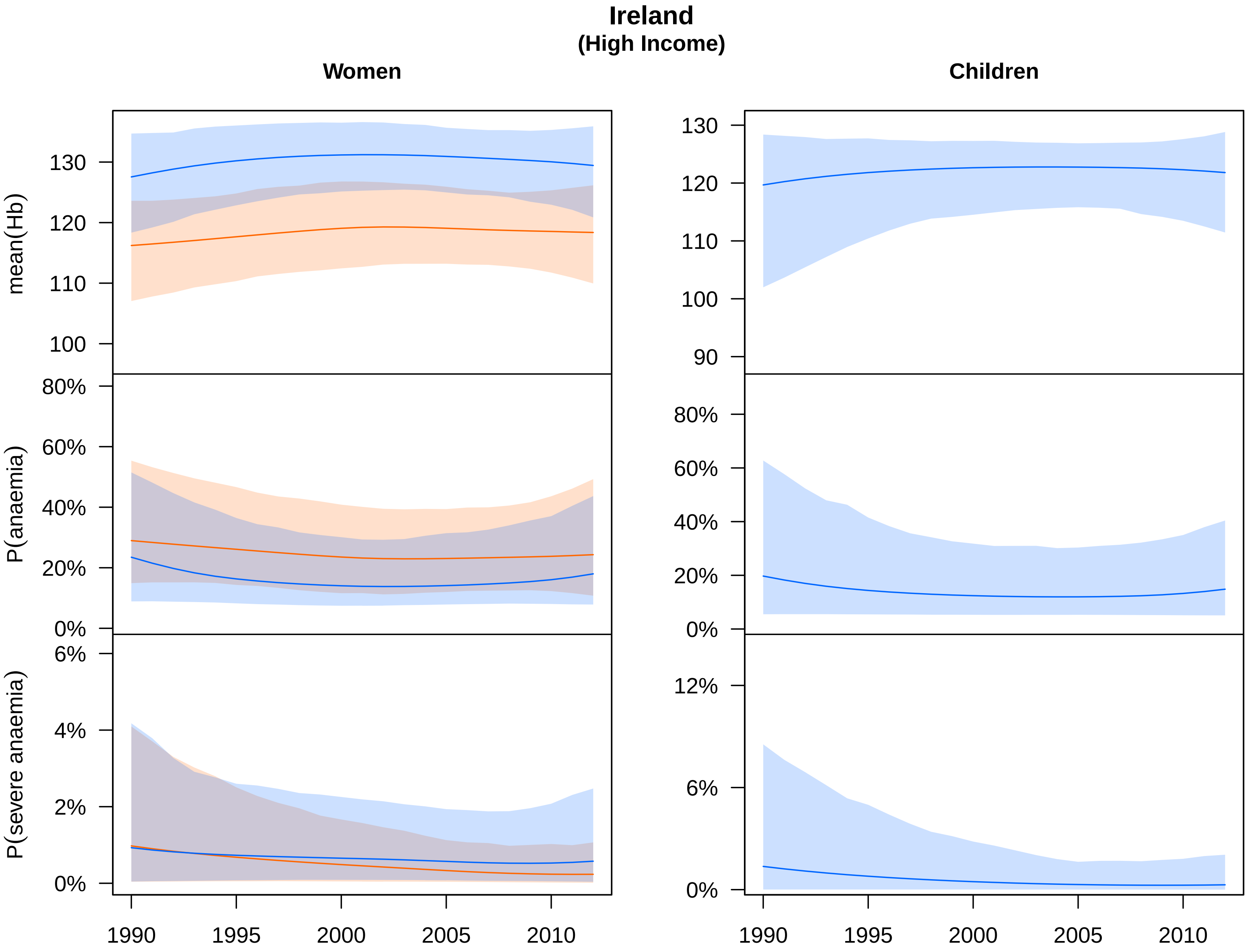

Ireland
(High Income)
Women
Children
mean(Hb)
P(anaemia)
P(severe anaemia)
130
120
110
100
90
80%
60%
40%
20%
0%
6%
4%
2%
12%
1990
1995
2000
2005
2010

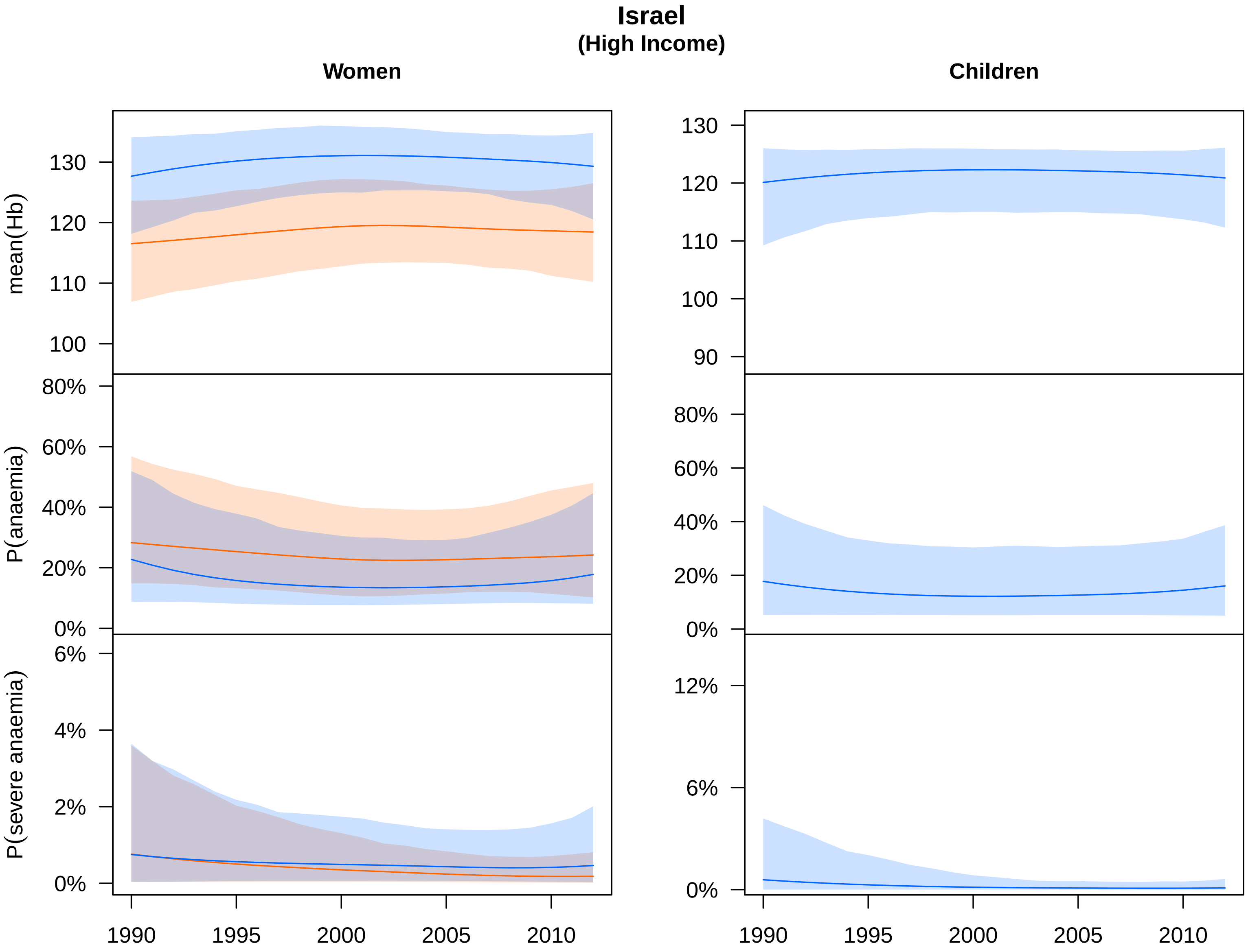
Israel
(High Income)
Women
Children
mean(Hb)
P(anaemia)
P(severe anaemia)
130
120
110
100
90
80%
60%
40%
20%
0%
6%
4%
2%
12%
1990
1995
2000
2005
2010

# Italy
**(High Income)**

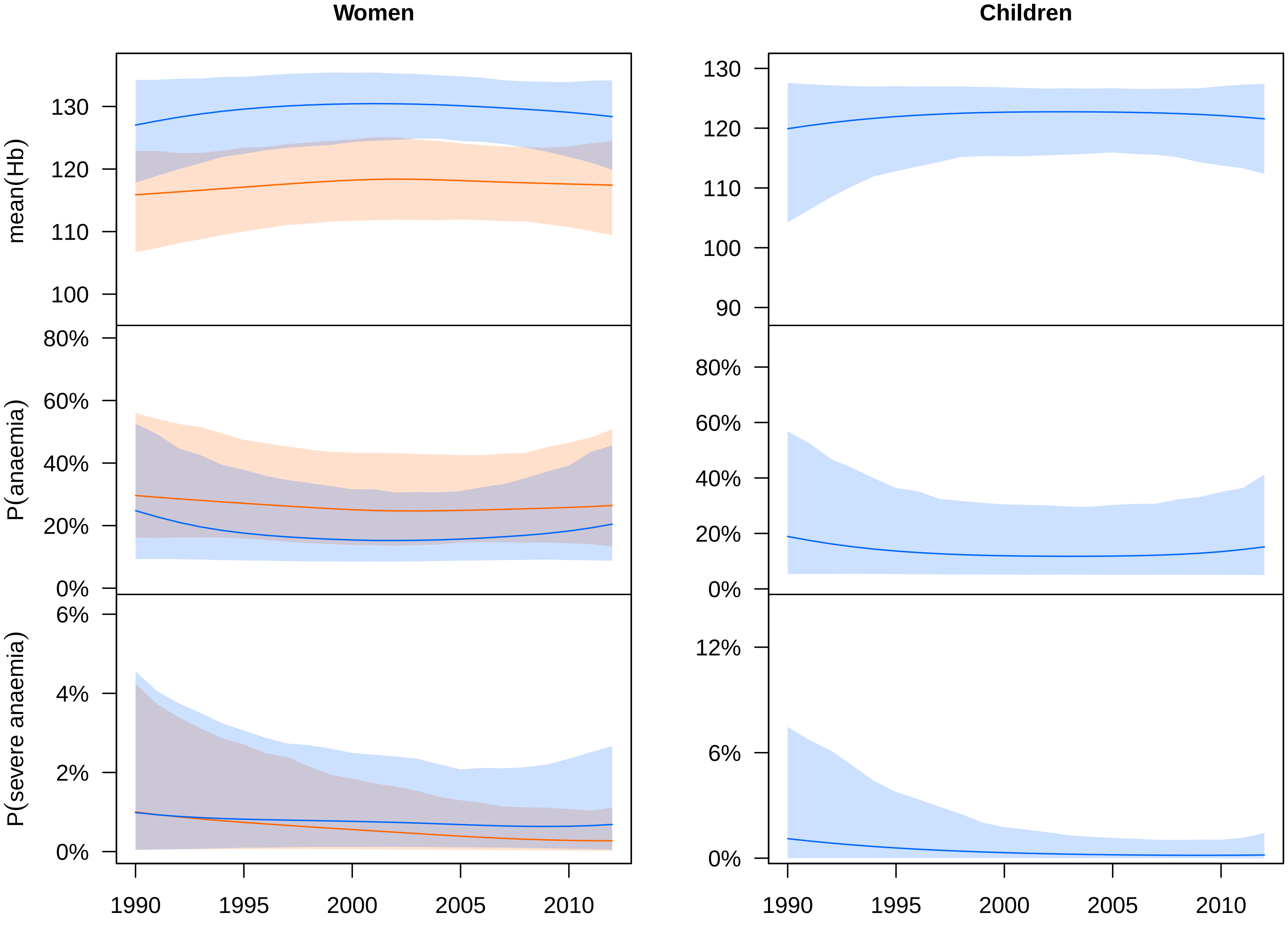

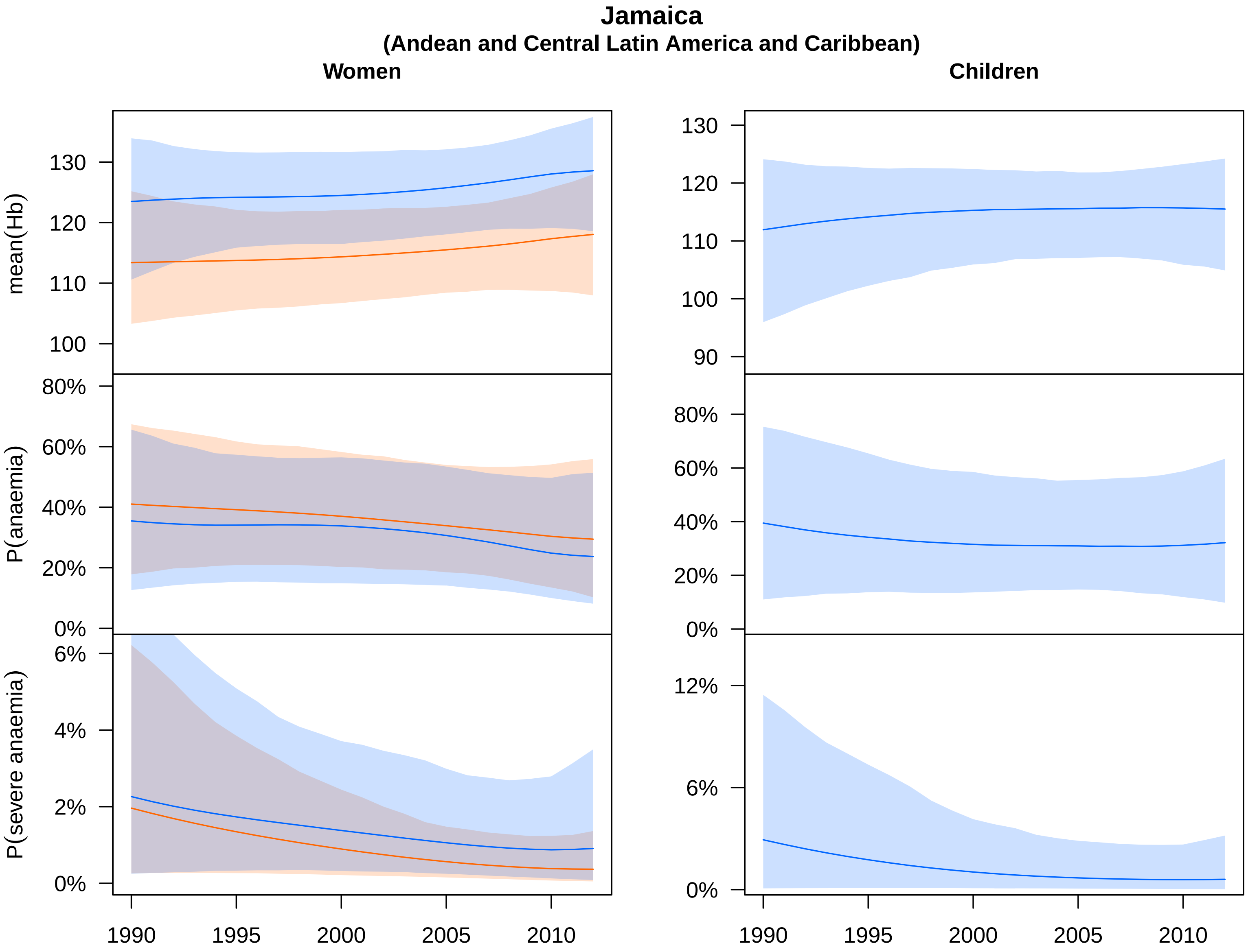
Jamaica
(Andean and Central Latin America and Caribbean)
Women
Children
mean(Hb)
P(anaemia)
P(severe anaemia)
130
120
110
100
90
80%
60%
40%
20%
0%
6%
4%
2%
12%
1990
1995
2000
2005
2010

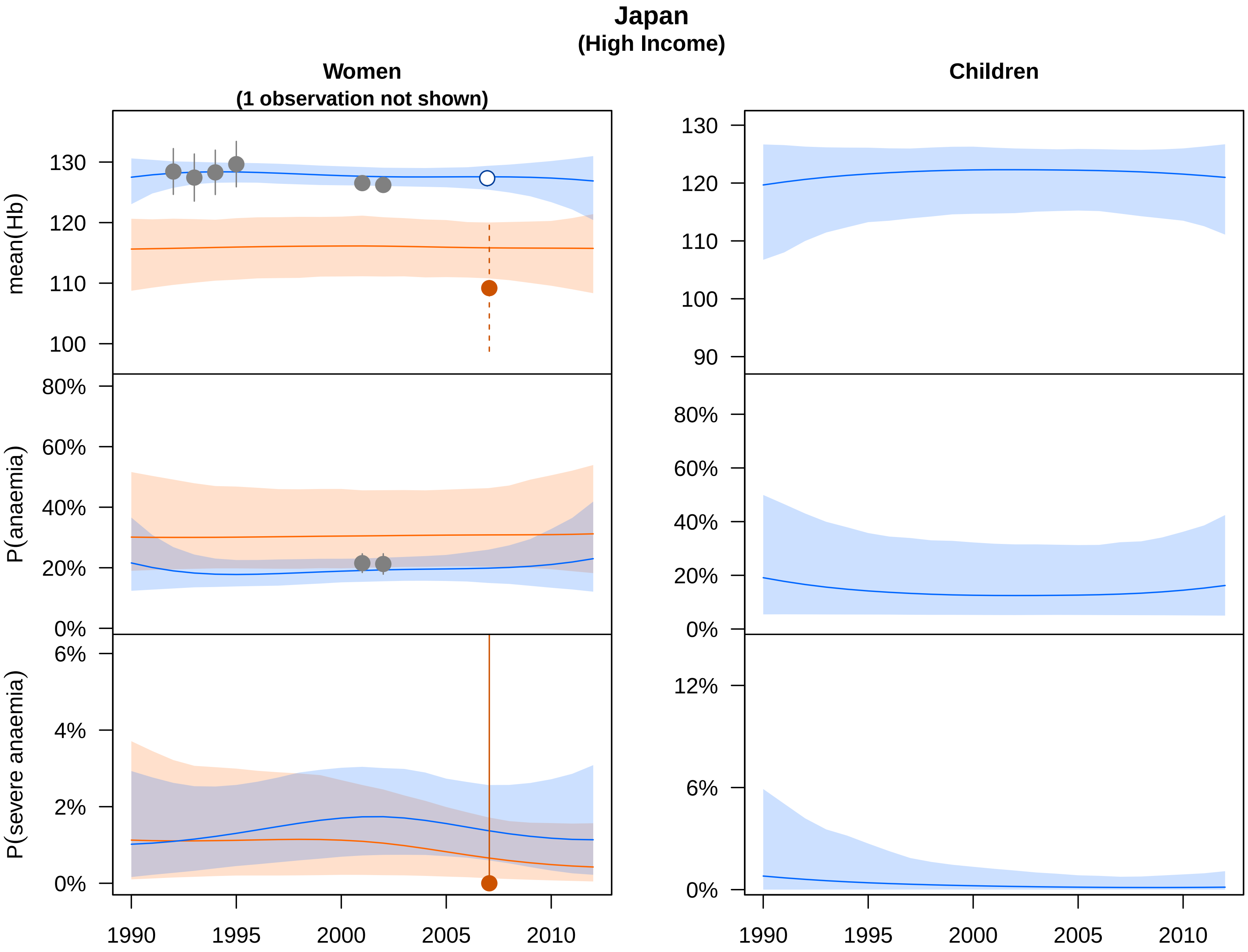
Japan
(High Income)
Women
(1 observation not shown)
Children
mean(Hb)
P(anaemia)
P(severe anaemia)
130
120
110
100
90
80%
60%
40%
20%
0%
6%
4%
2%
12%
1990
1995
2000
2005
2010

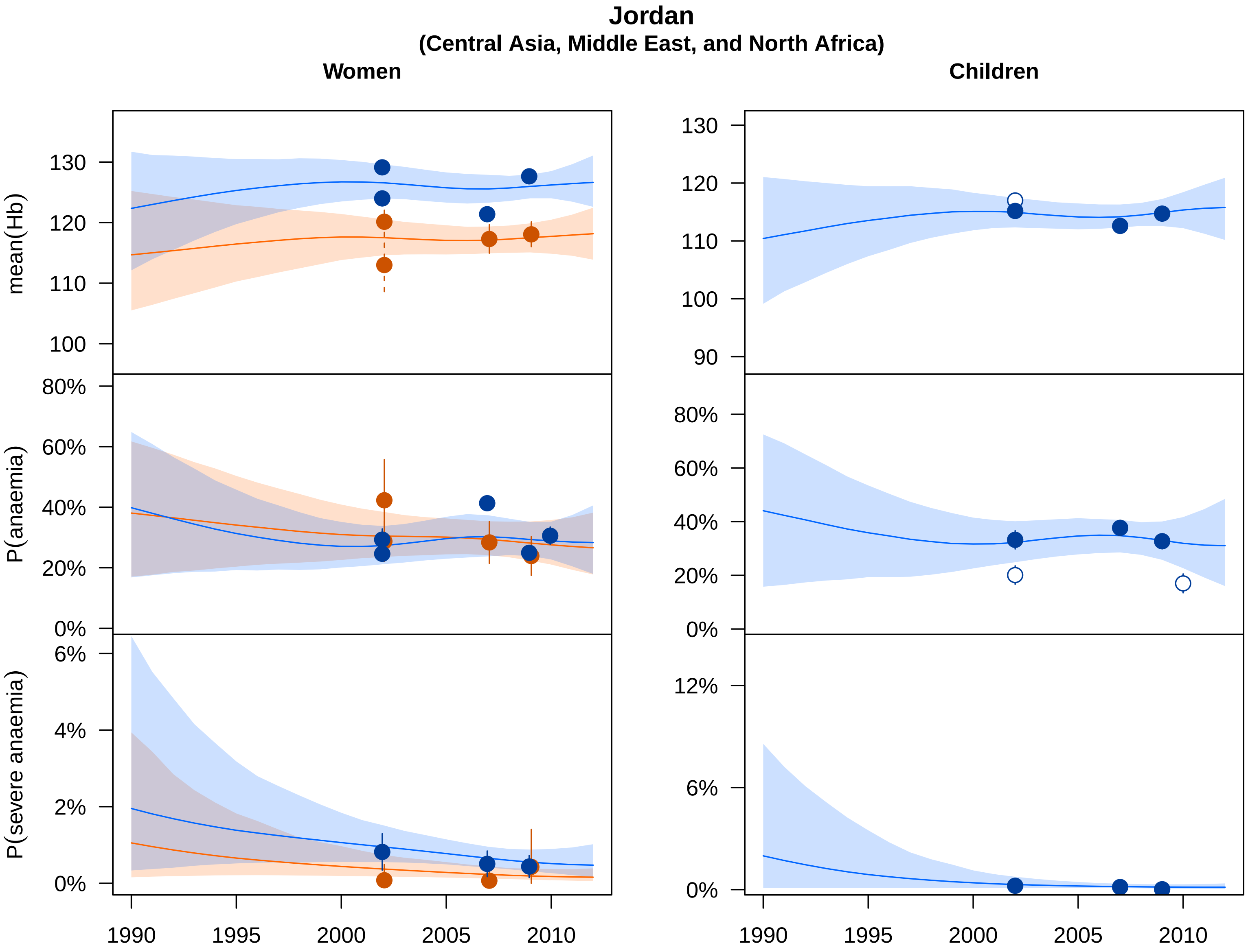
Jordan
(Central Asia, Middle East, and North Africa)
Women
Children
mean(Hb)
P(anaemia)
P(severe anaemia)
130
120
110
100
90
80%
60%
40%
20%
0%
6%
4%
2%
12%
1990
1995
2000
2005
2010

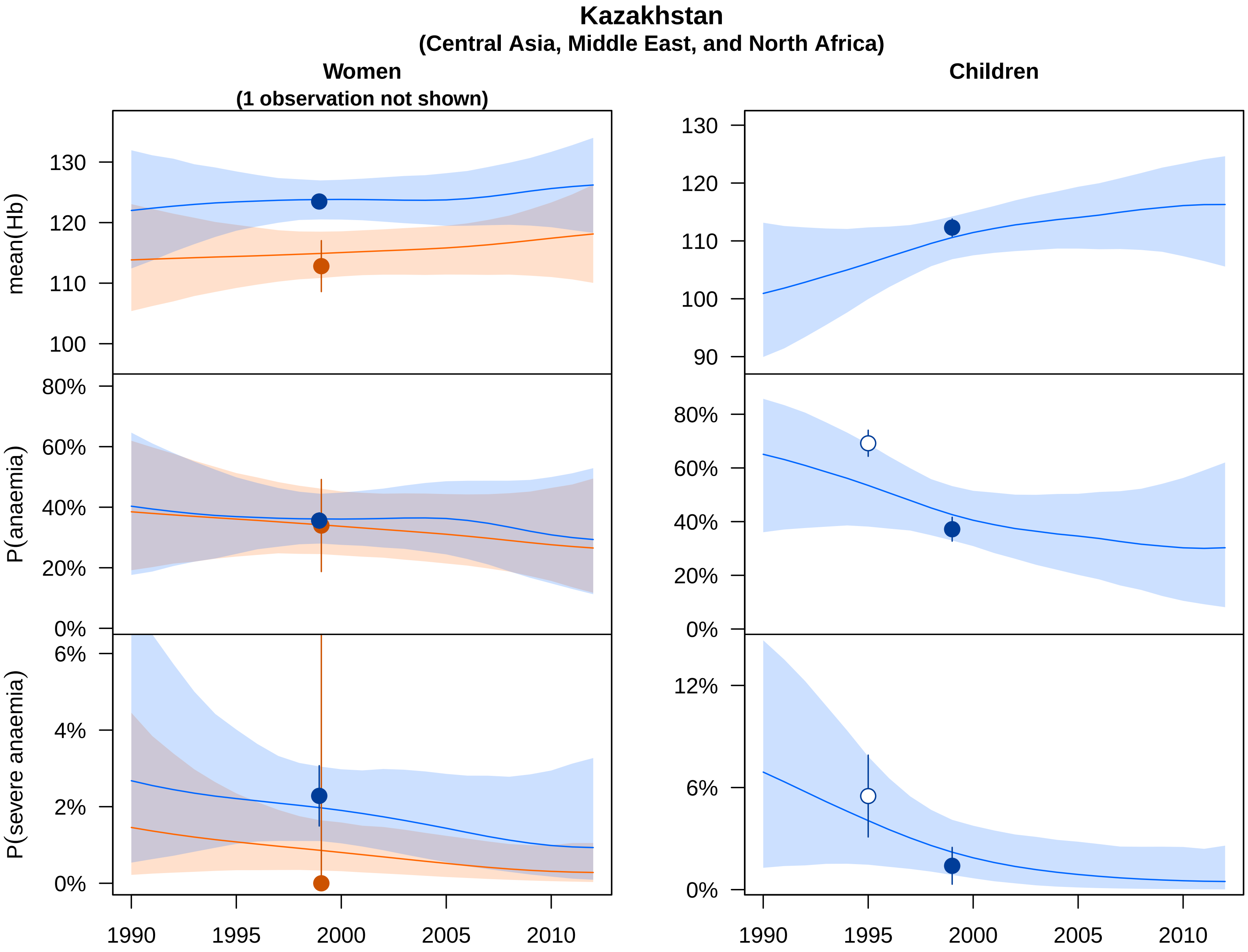
Kazakhstan
(Central Asia, Middle East, and North Africa)
Women
(1 observation not shown)
Children
mean(Hb)
P(anaemia)
P(severe anaemia)
130
120
110
100
90
80%
60%
40%
20%
0%
6%
4%
2%
12%
1990
1995
2000
2005
2010

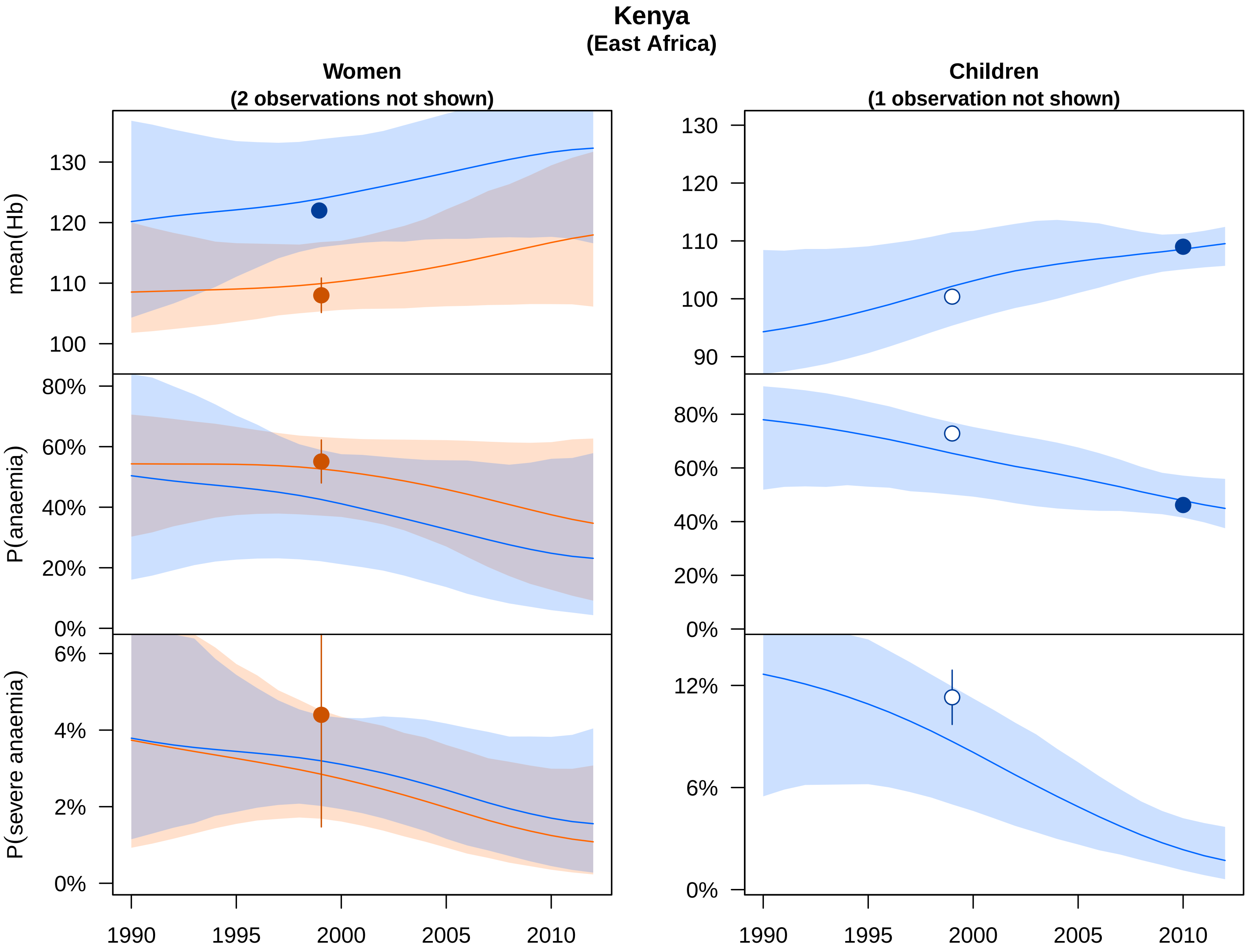
Kenya
(East Africa)
Women
(2 observations not shown)
Children
(1 observation not shown)
mean(Hb)
P(anaemia)
P(severe anaemia)
130
120
110
100
90
80%
60%
40%
20%
0%
6%
4%
2%
12%
1990
1995
2000
2005
2010

## Kiribati
### (Oceania)

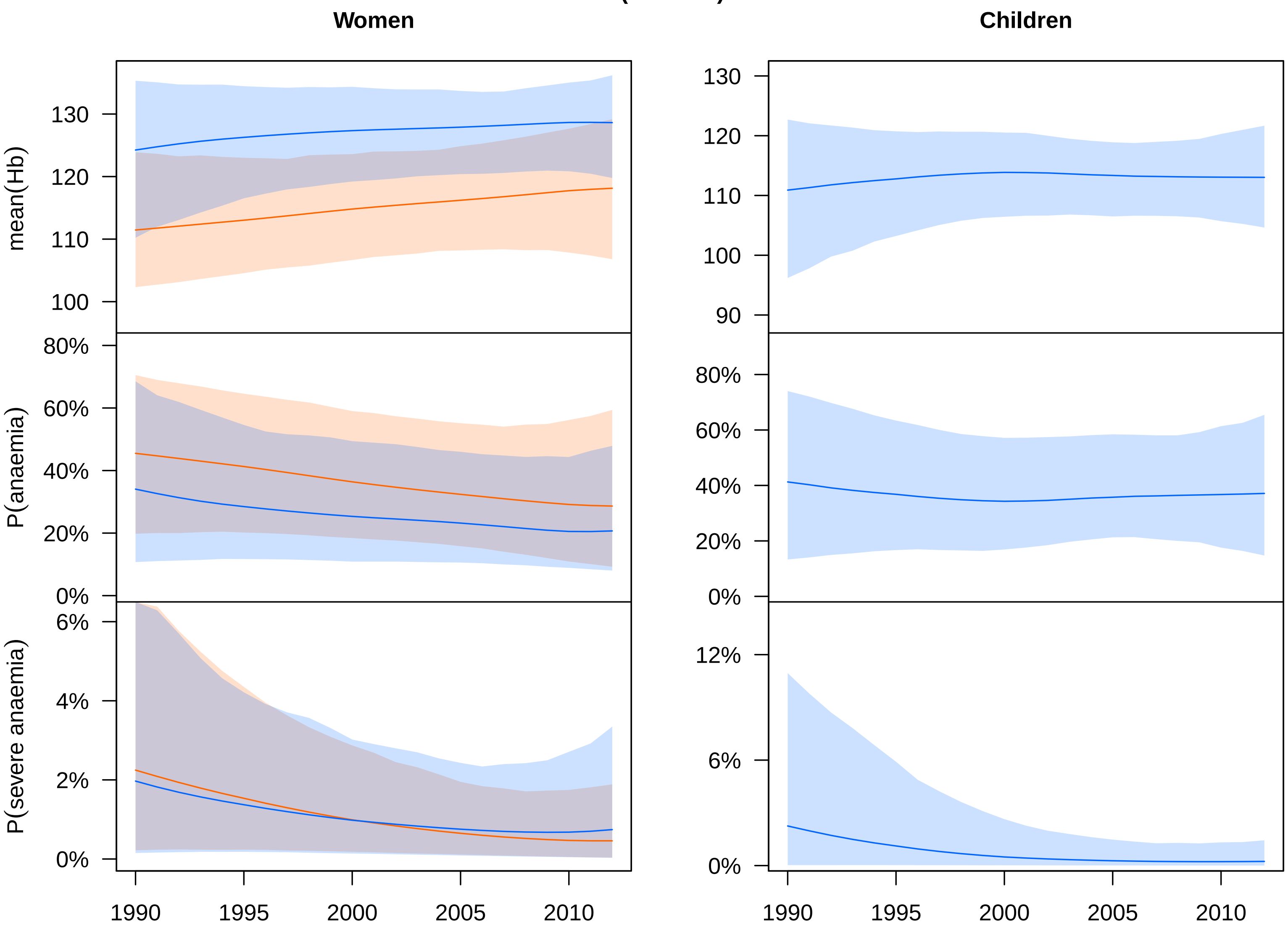

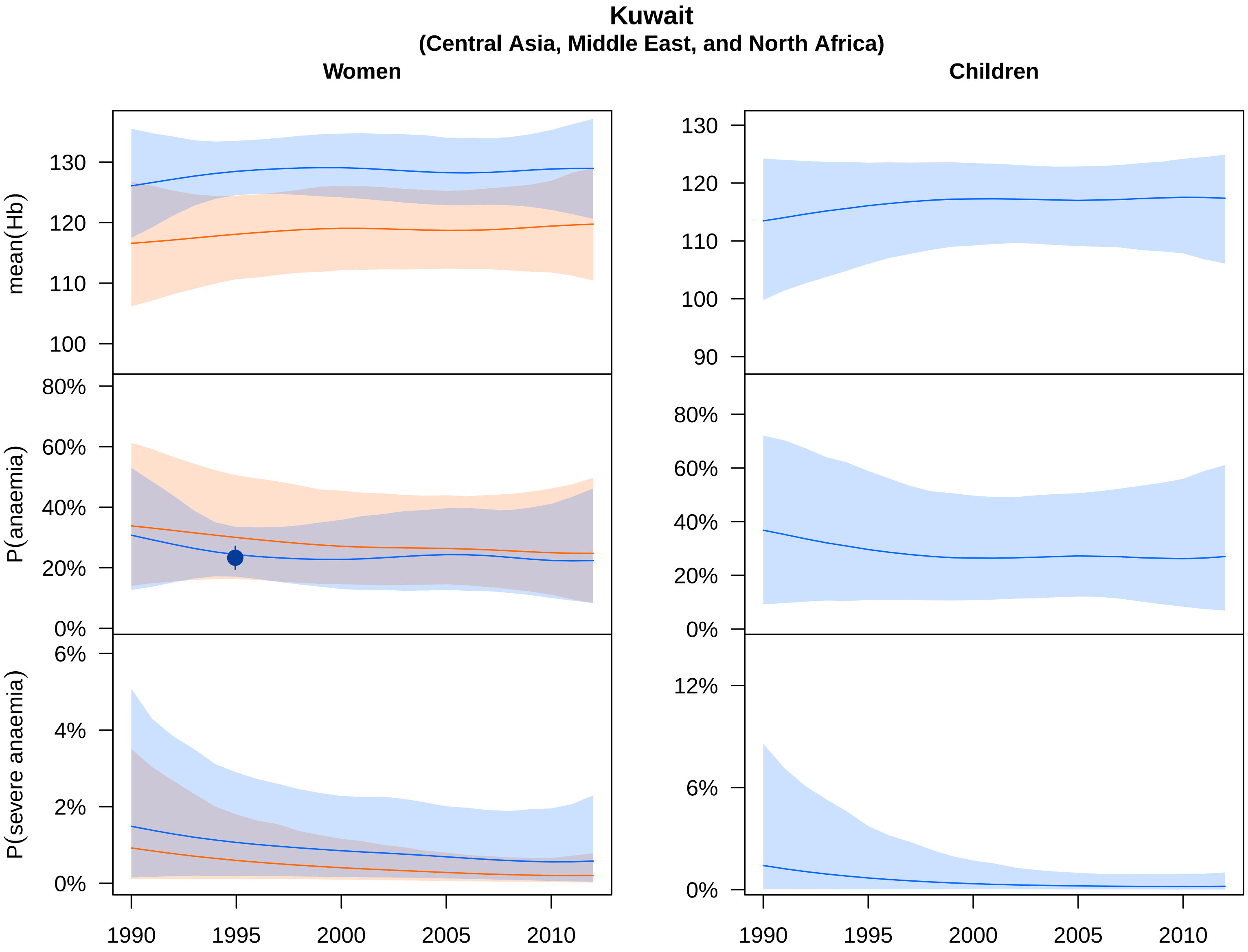
Kuwait
(Central Asia, Middle East, and North Africa)
Women
Children
mean(Hb)
P(anaemia)
P(severe anaemia)
130
120
110
100
90
80%
60%
40%
20%
0%
6%
4%
2%
12%
1990
1995
2000
2005
2010

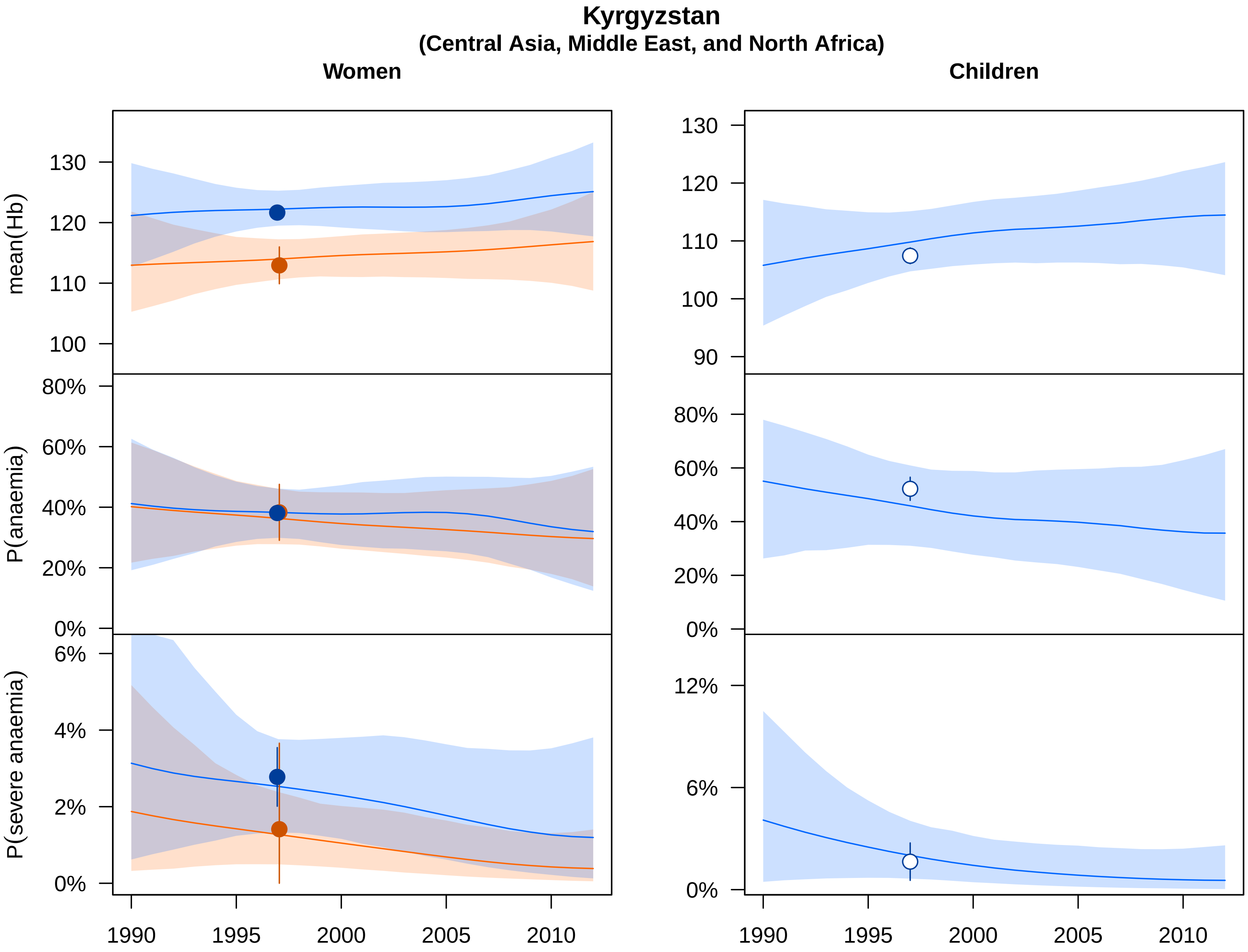
Kyrgyzstan
(Central Asia, Middle East, and North Africa)
Women
Children
mean(Hb)
P(anaemia)
P(severe anaemia)
130
120
110
100
90
80%
60%
40%
20%
0%
6%
4%
2%
12%
1990
1995
2000
2005
2010

# Lao People's Democratic Republic
**(East and Southeast Asia)**

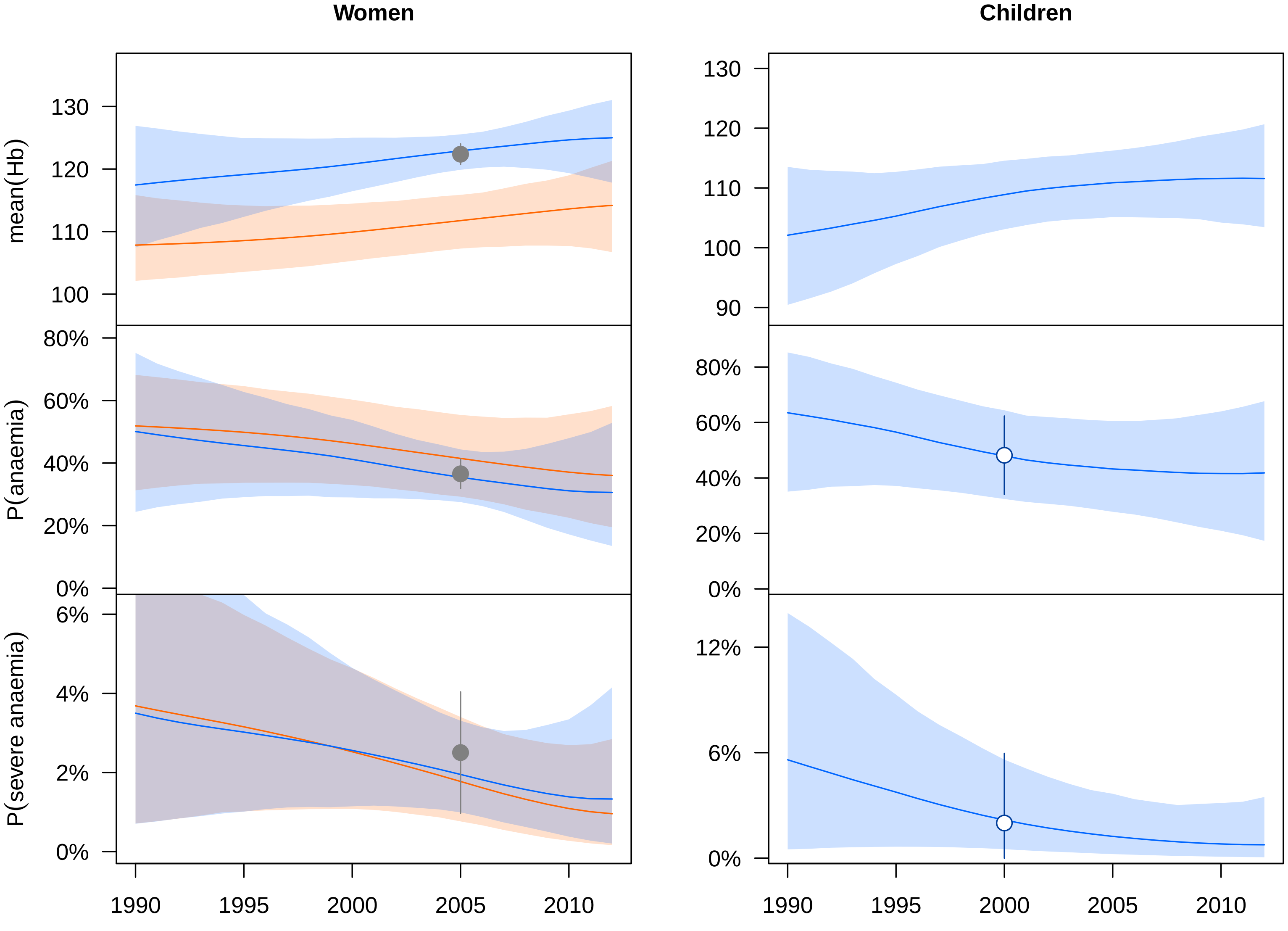

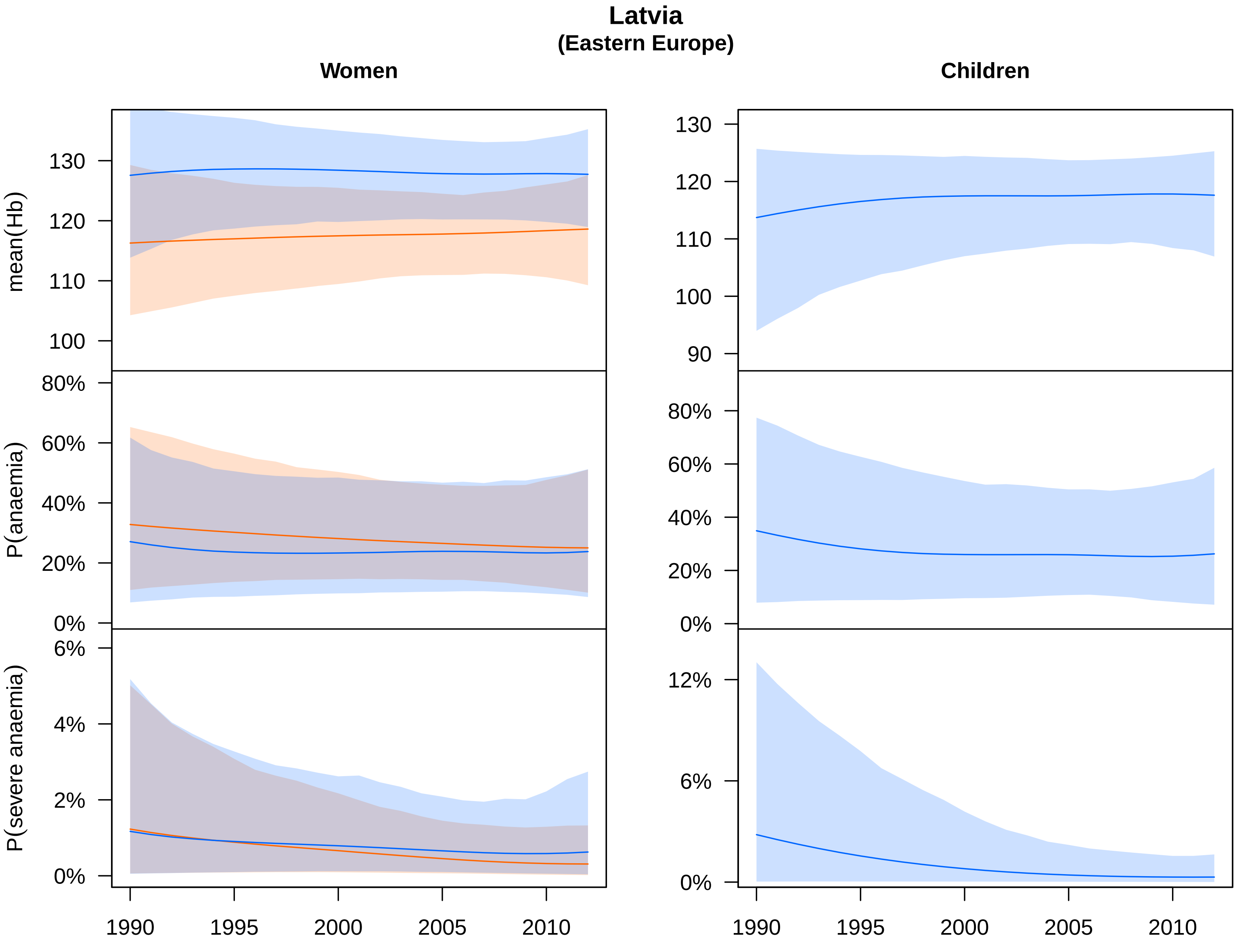
Latvia
(Eastern Europe)
Women
Children
mean(Hb)
P(anaemia)
P(severe anaemia)
130
120
110
100
90
80%
60%
40%
20%
0%
6%
4%
2%
12%
1990
1995
2000
2005
2010

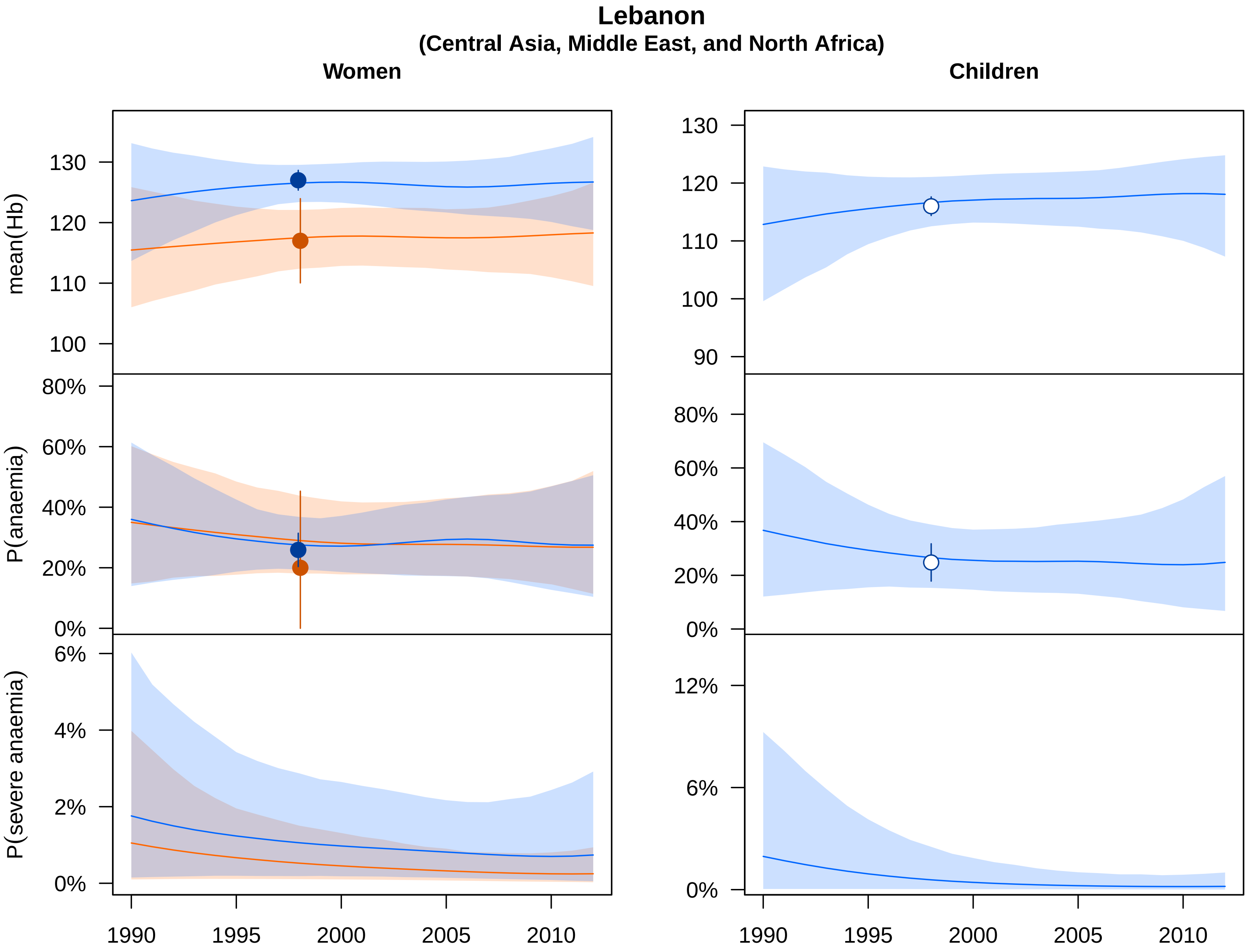
Lebanon
(Central Asia, Middle East, and North Africa)
Women
Children
mean(Hb)
P(anaemia)
P(severe anaemia)
130
120
110
100
90
80%
60%
40%
20%
0%
6%
4%
2%
12%
1990
1995
2000
2005
2010

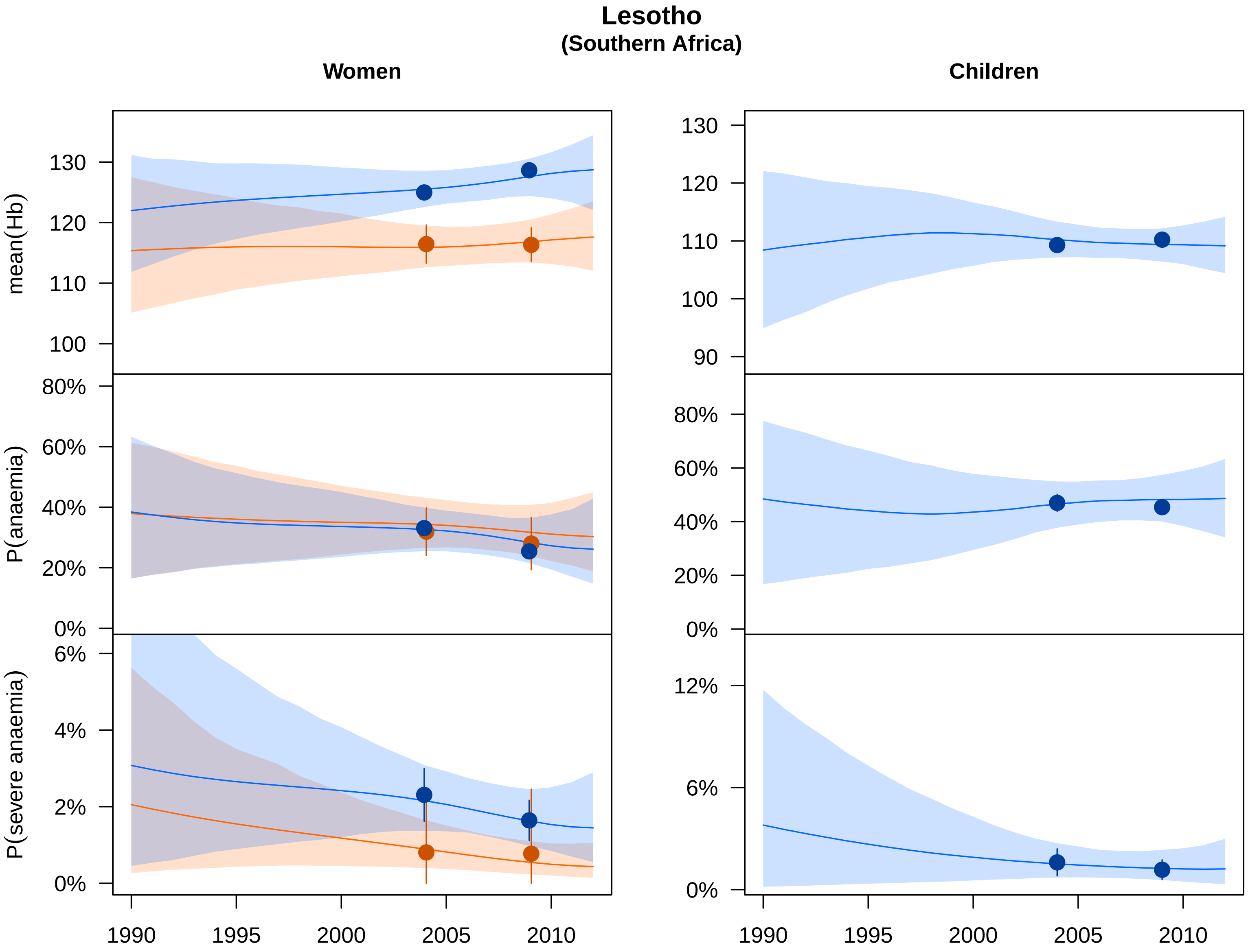
Lesotho
(Southern Africa)
Women
Children
mean(Hb)
P(anaemia)
P(severe anaemia)
130
120
110
100
90
80%
60%
40%
20%
0%
6%
4%
2%
12%
1990
1995
2000
2005
2010

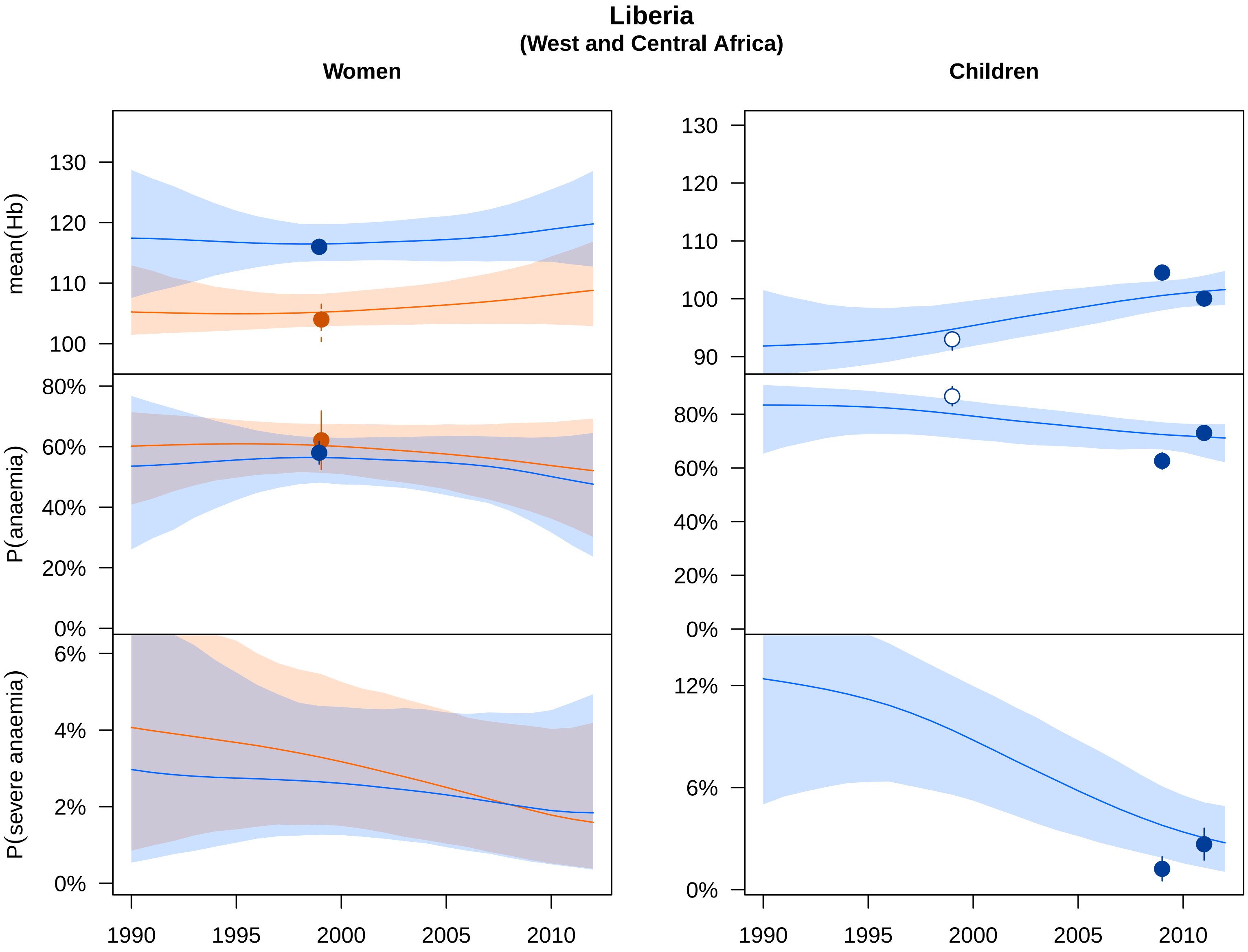
Liberia
(West and Central Africa)
Women
Children
mean(Hb)
P(anaemia)
P(severe anaemia)
130
120
110
100
90
80%
60%
40%
20%
0%
6%
4%
2%
12%
1990
1995
2000
2005
2010

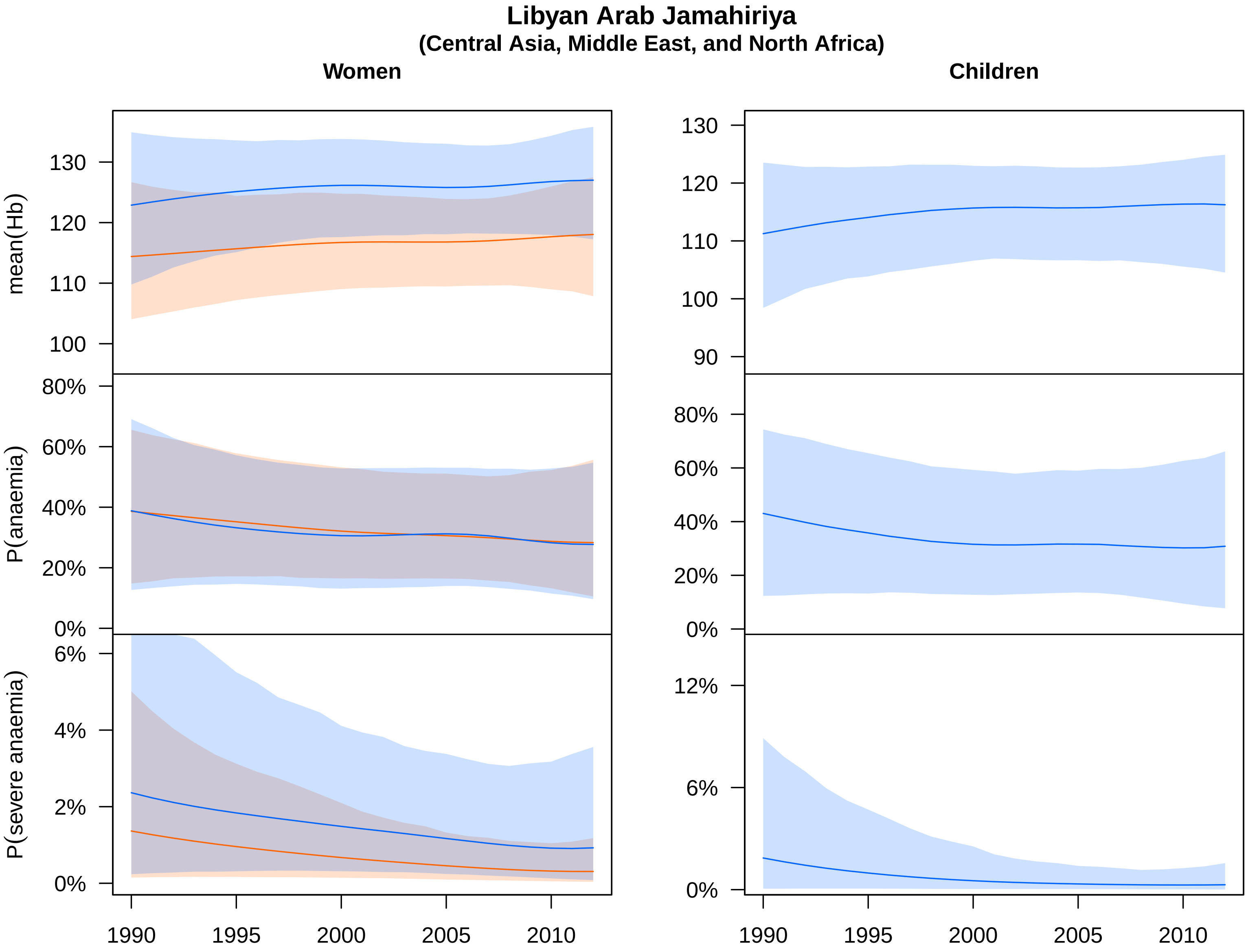
Libyan Arab Jamahiriya
(Central Asia, Middle East, and North Africa)
Women
Children
mean(Hb)
P(anaemia)
P(severe anaemia)
130
120
110
100
90
80%
60%
40%
20%
0%
6%
4%
2%
12%
1990
1995
2000
2005
2010

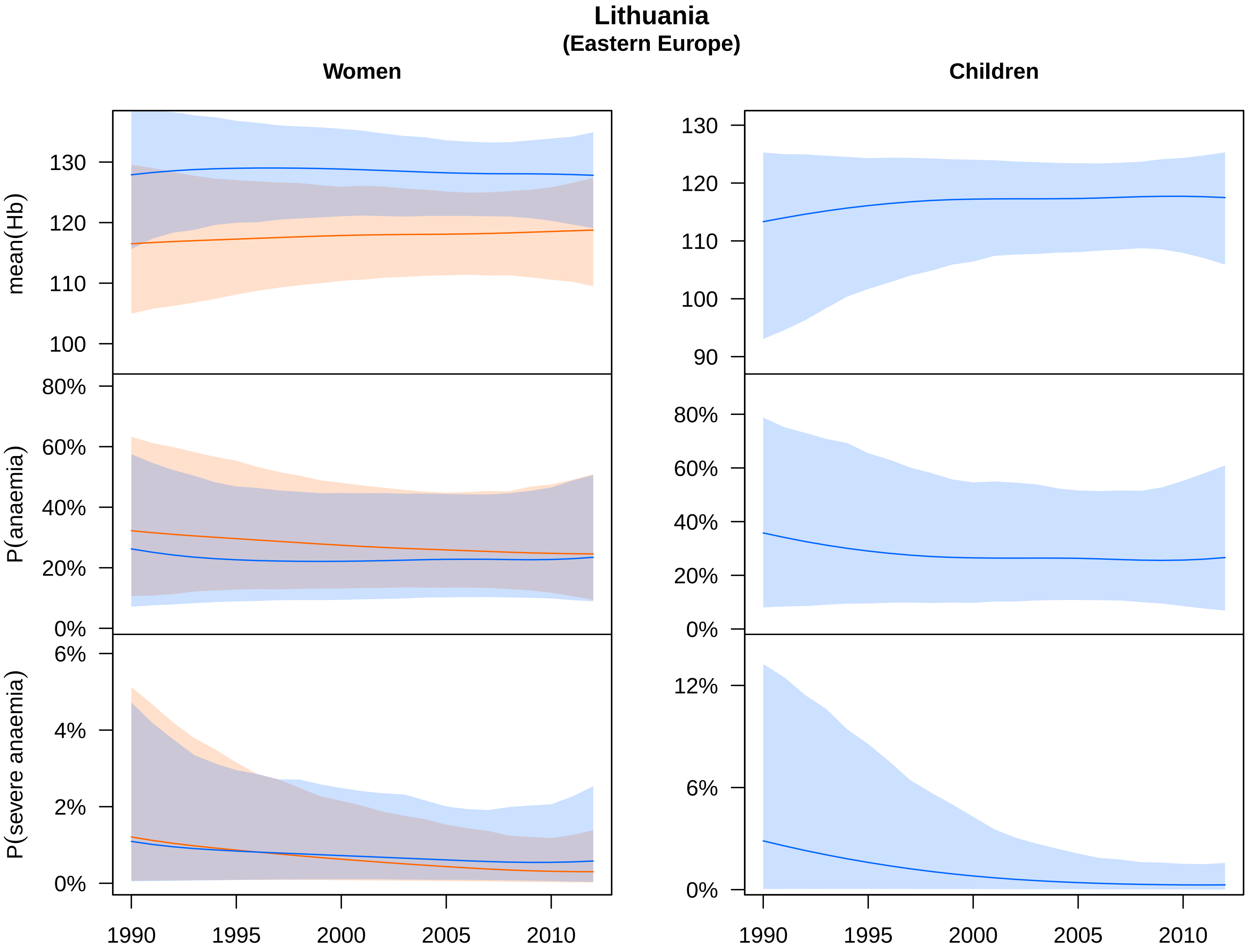
Lithuania
(Eastern Europe)
Women
Children
mean(Hb)
P(anaemia)
P(severe anaemia)
130
120
110
100
90
80%
60%
40%
20%
0%
6%
4%
2%
12%
1990
1995
2000
2005
2010

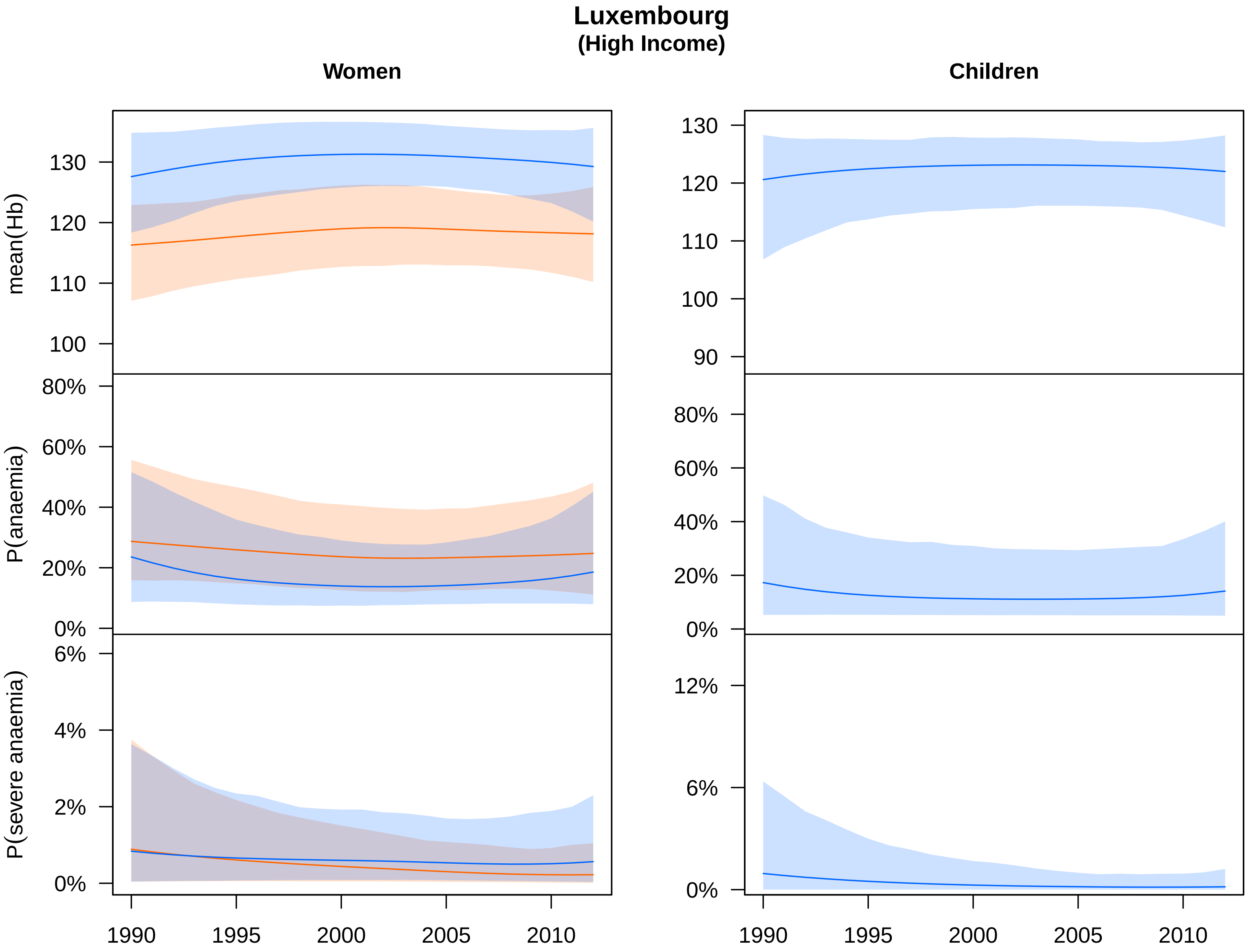
Luxembourg
(High Income)
Women
Children
mean(Hb)
P(anaemia)
P(severe anaemia)
130
120
110
100
90
80%
60%
40%
20%
0%
6%
4%
2%
12%
1990
1995
2000
2005
2010

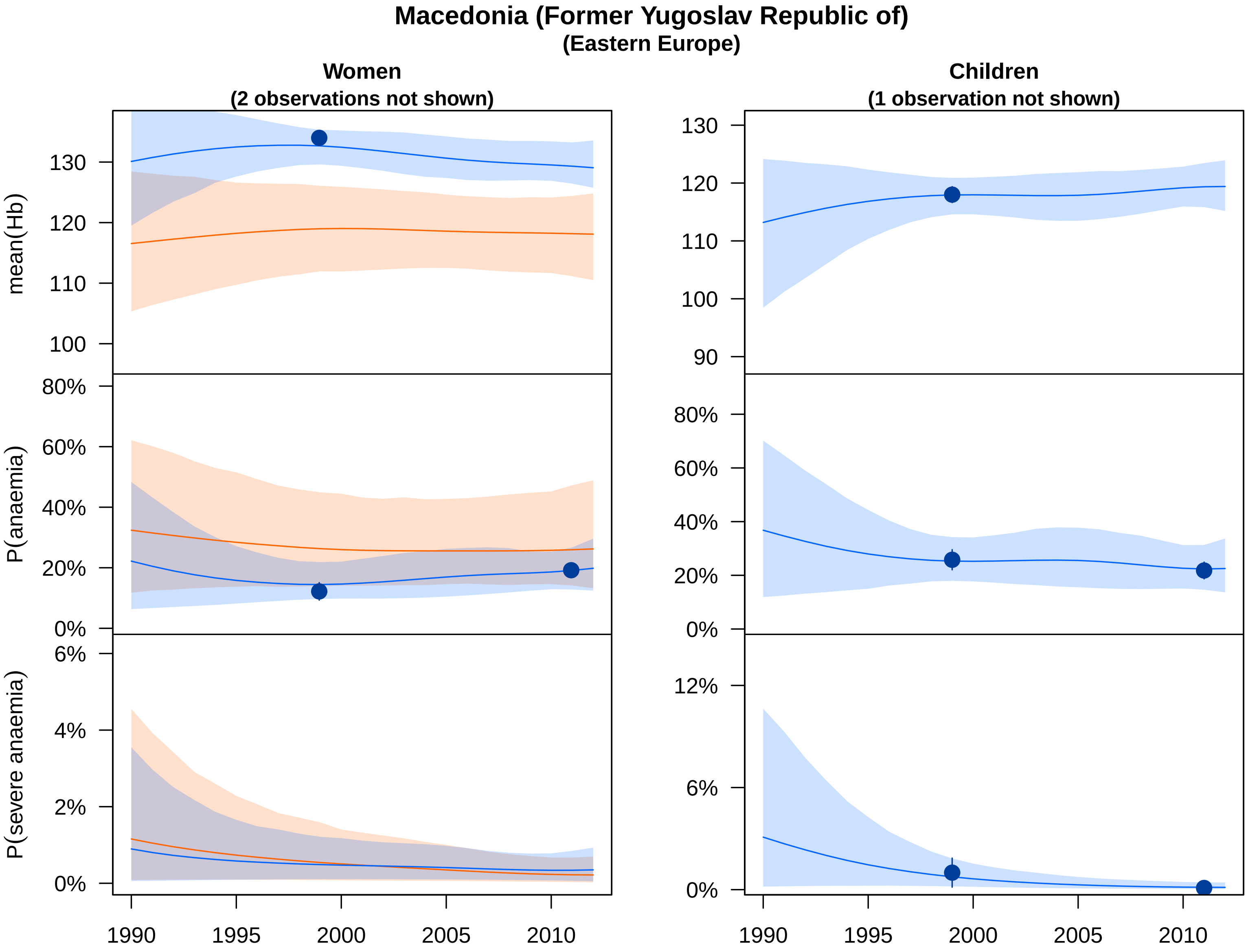
Macedonia (Former Yugoslav Republic of)
(Eastern Europe)
Women
(2 observations not shown)
Children
(1 observation not shown)
mean(Hb)
P(anaemia)
P(severe anaemia)
130
120
110
100
90
80%
60%
40%
20%
0%
6%
4%
2%
12%
1990
1995
2000
2005
2010

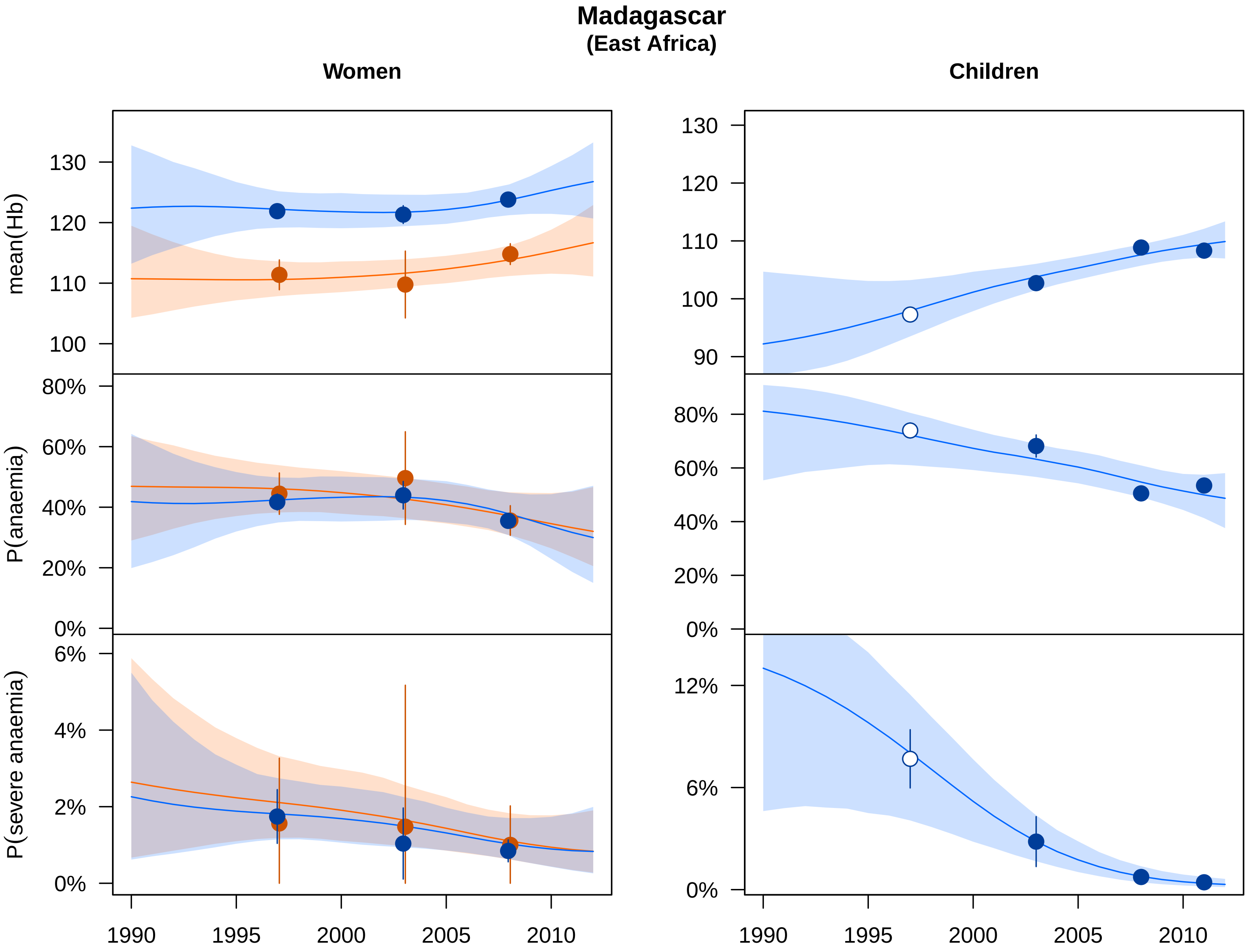

Madagascar
(East Africa)
Women
Children
mean(Hb)
P(anaemia)
P(severe anaemia)
130
120
110
100
90
80%
60%
40%
20%
0%
6%
4%
2%
12%
1990
1995
2000
2005
2010

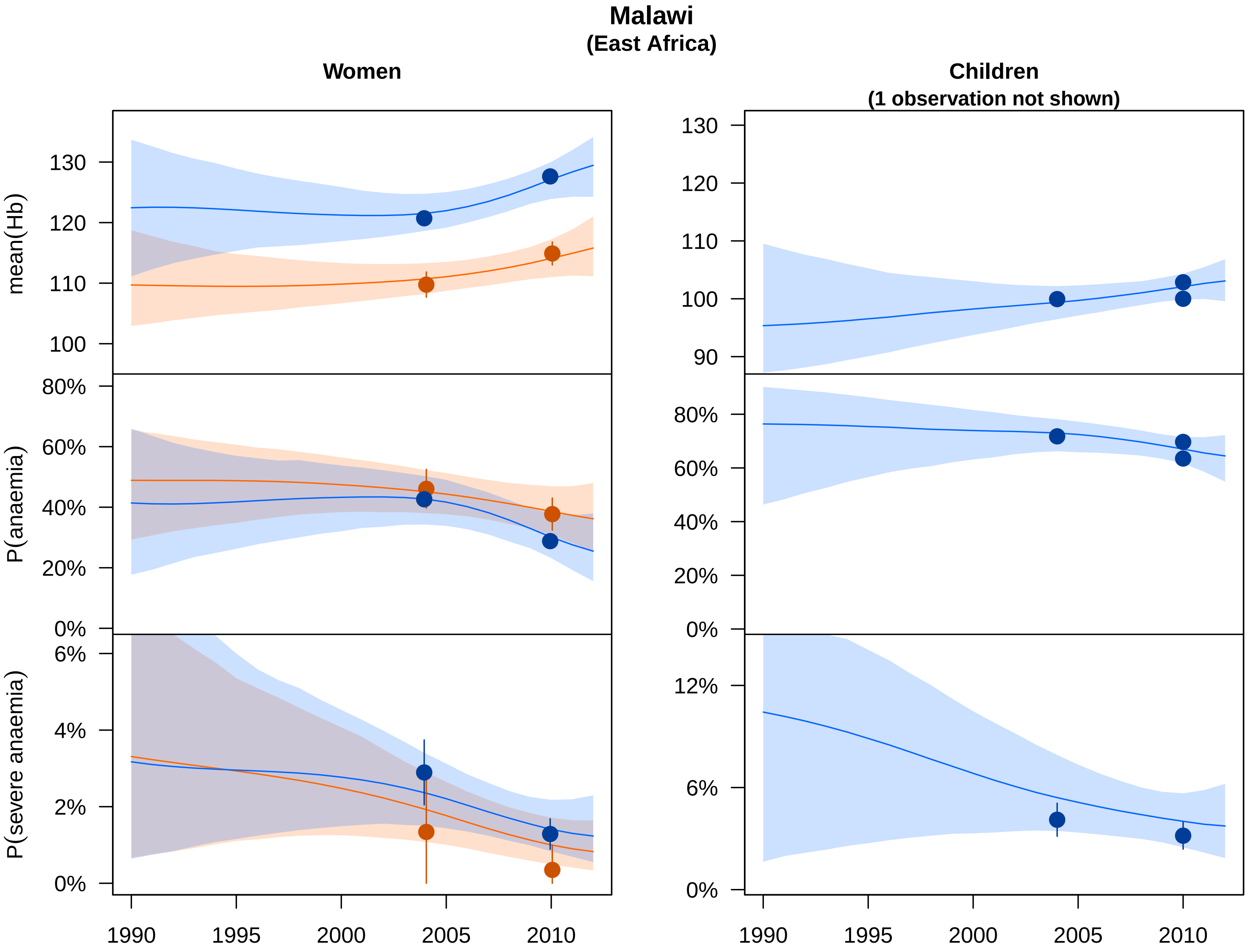
Malawi
(East Africa)
Women
Children
(1 observation not shown)
mean(Hb)
P(anaemia)
P(severe anaemia)
130
120
110
100
90
80%
60%
40%
20%
0%
6%
4%
2%
12%
1990
1995
2000
2005
2010

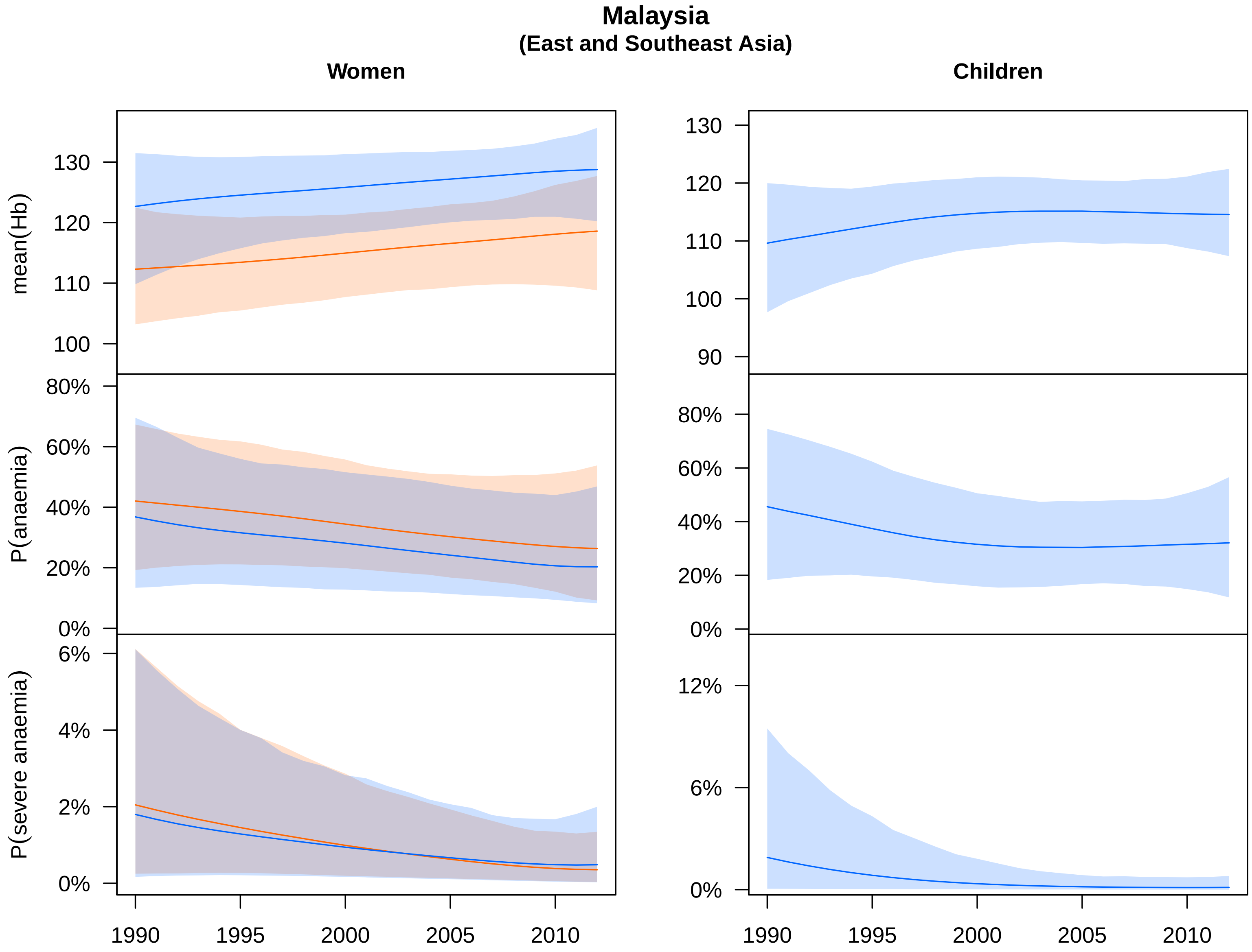
Malaysia
(East and Southeast Asia)
Women
Children
mean(Hb)
P(anaemia)
P(severe anaemia)
130
120
110
100
90
80%
60%
40%
20%
0%
6%
4%
2%
12%
1990
1995
2000
2005
2010

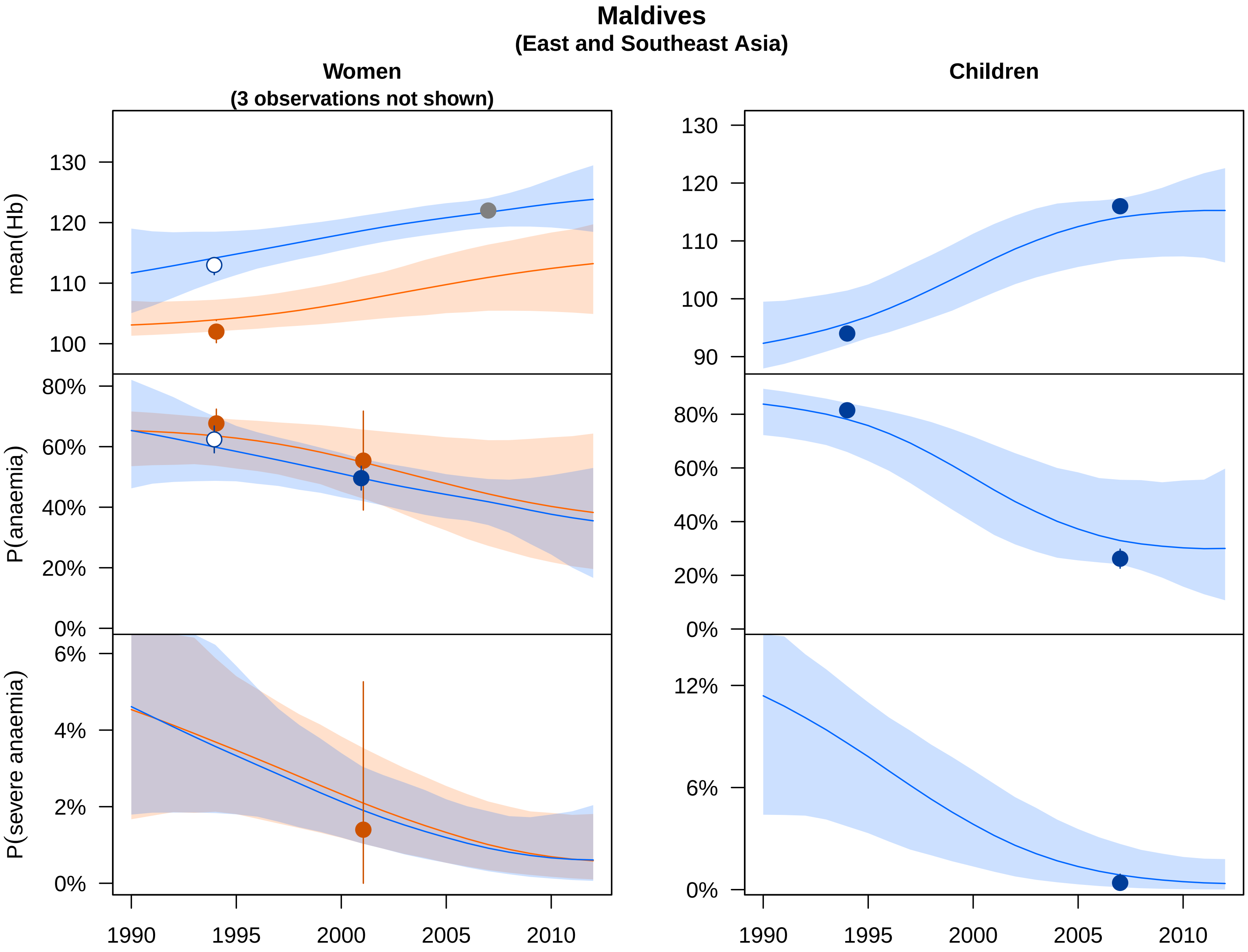
Maldives
(East and Southeast Asia)
Women
(3 observations not shown)
Children
mean(Hb)
P(anaemia)
P(severe anaemia)
130
120
110
100
90
80%
60%
40%
20%
0%
6%
4%
2%
12%
1990
1995
2000
2005
2010

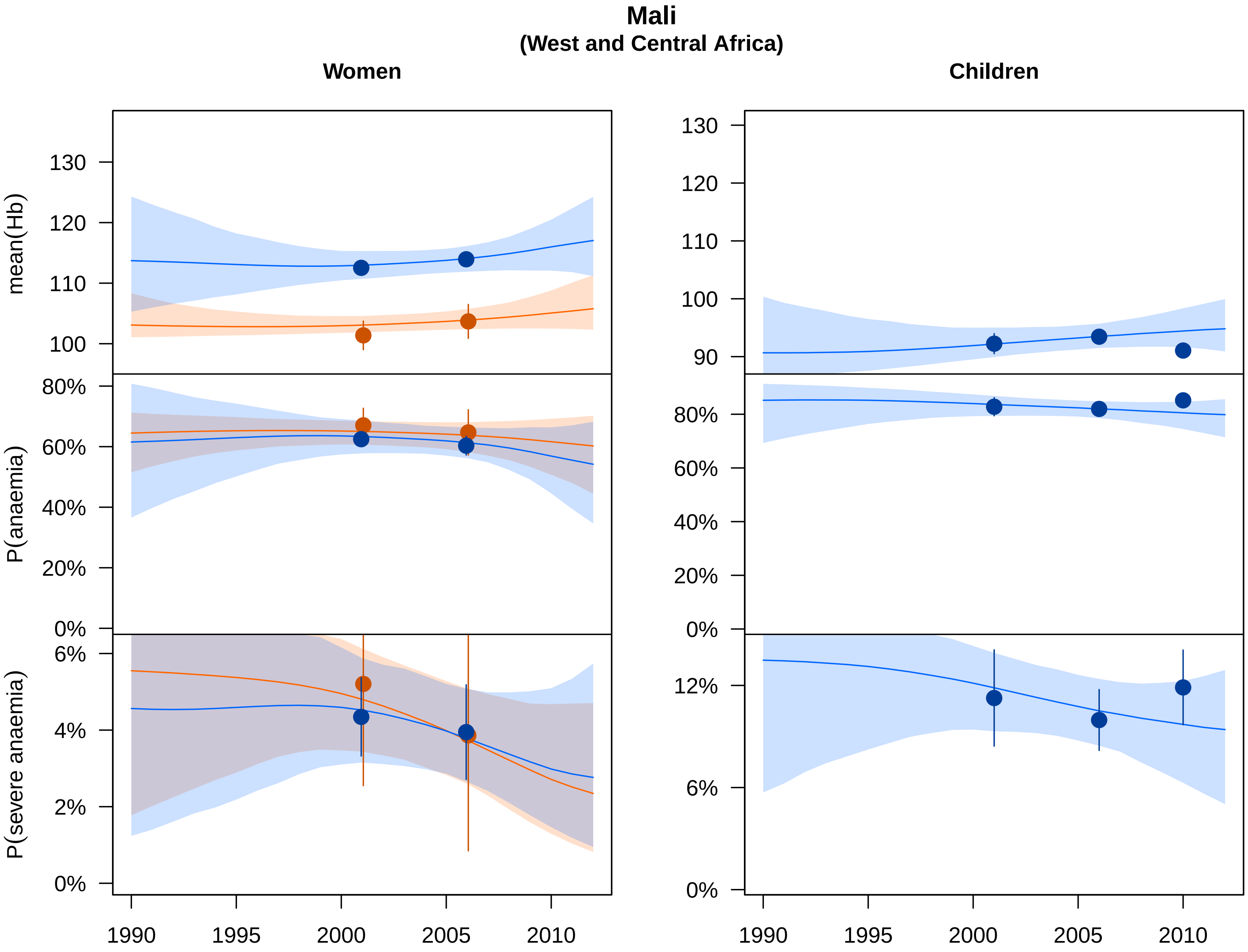
Mali
(West and Central Africa)
Women
Children
mean(Hb)
P(anaemia)
P(severe anaemia)
130
120
110
100
90
80%
60%
40%
20%
0%
6%
4%
2%
12%
1990
1995
2000
2005
2010

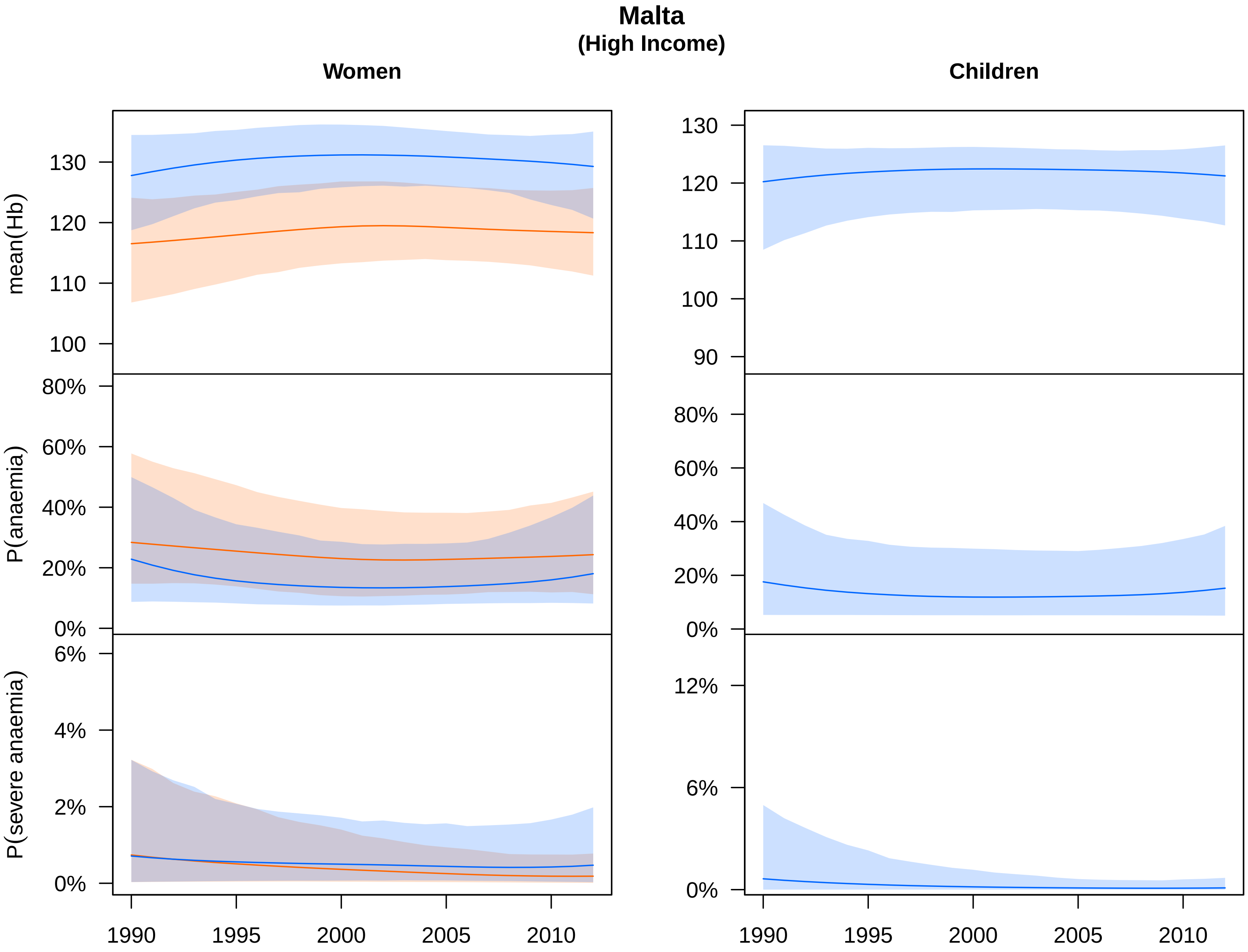
Malta
(High Income)
Women
Children
mean(Hb)
130
120
110
100
90
P(anaemia)
80%
60%
40%
20%
0%
P(severe anaemia)
6%
4%
2%
12%
1990
1995
2000
2005
2010

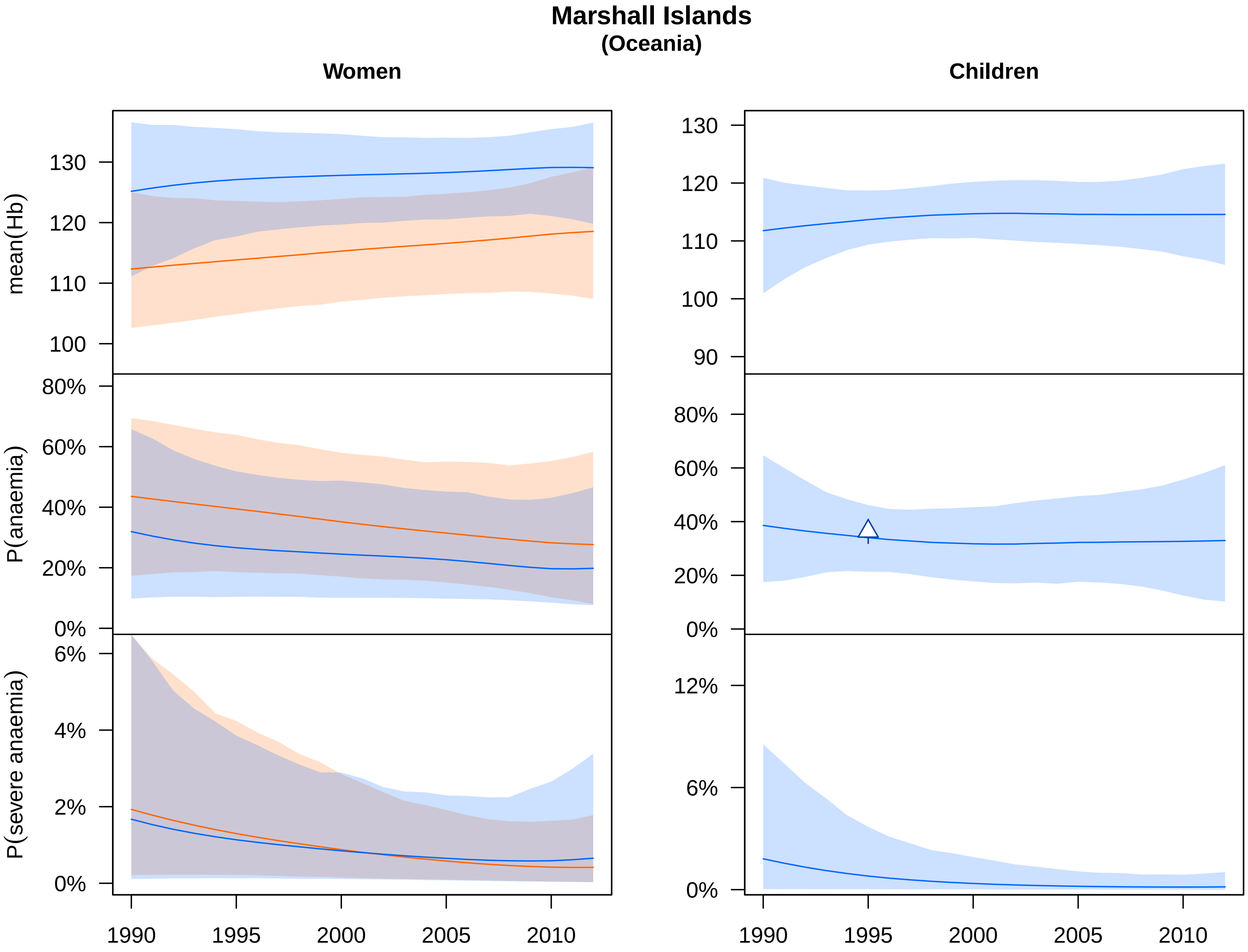
Marshall Islands
(Oceania)
Women
Children
mean(Hb)
P(anaemia)
P(severe anaemia)
130
120
110
100
90
80%
60%
40%
20%
0%
6%
4%
2%
12%
1990
1995
2000
2005
2010

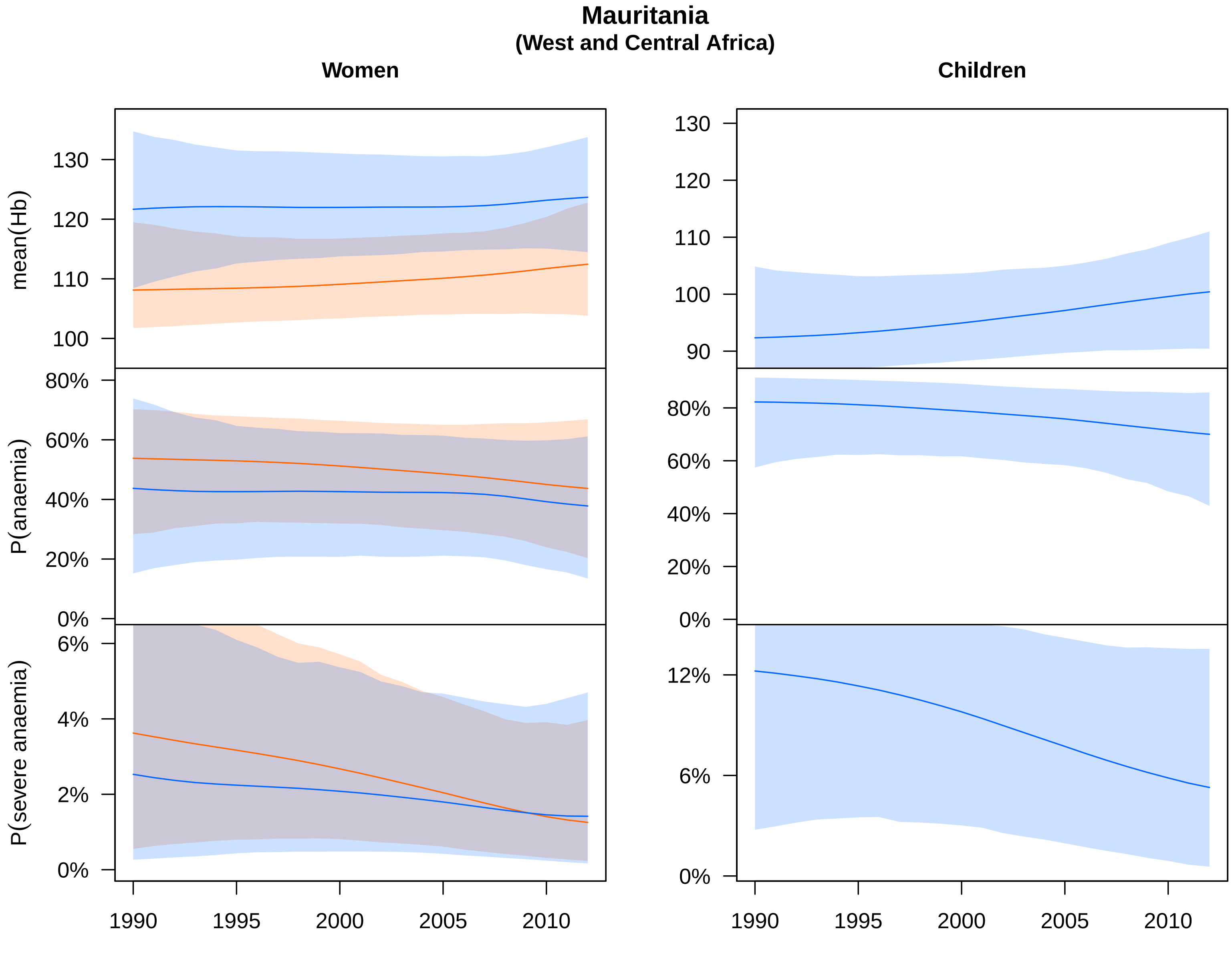
Mauritania
(West and Central Africa)
Women
Children
mean(Hb)
P(anaemia)
P(severe anaemia)
130
120
110
100
90
80%
60%
40%
20%
0%
6%
4%
2%
12%
1990
1995
2000
2005
2010

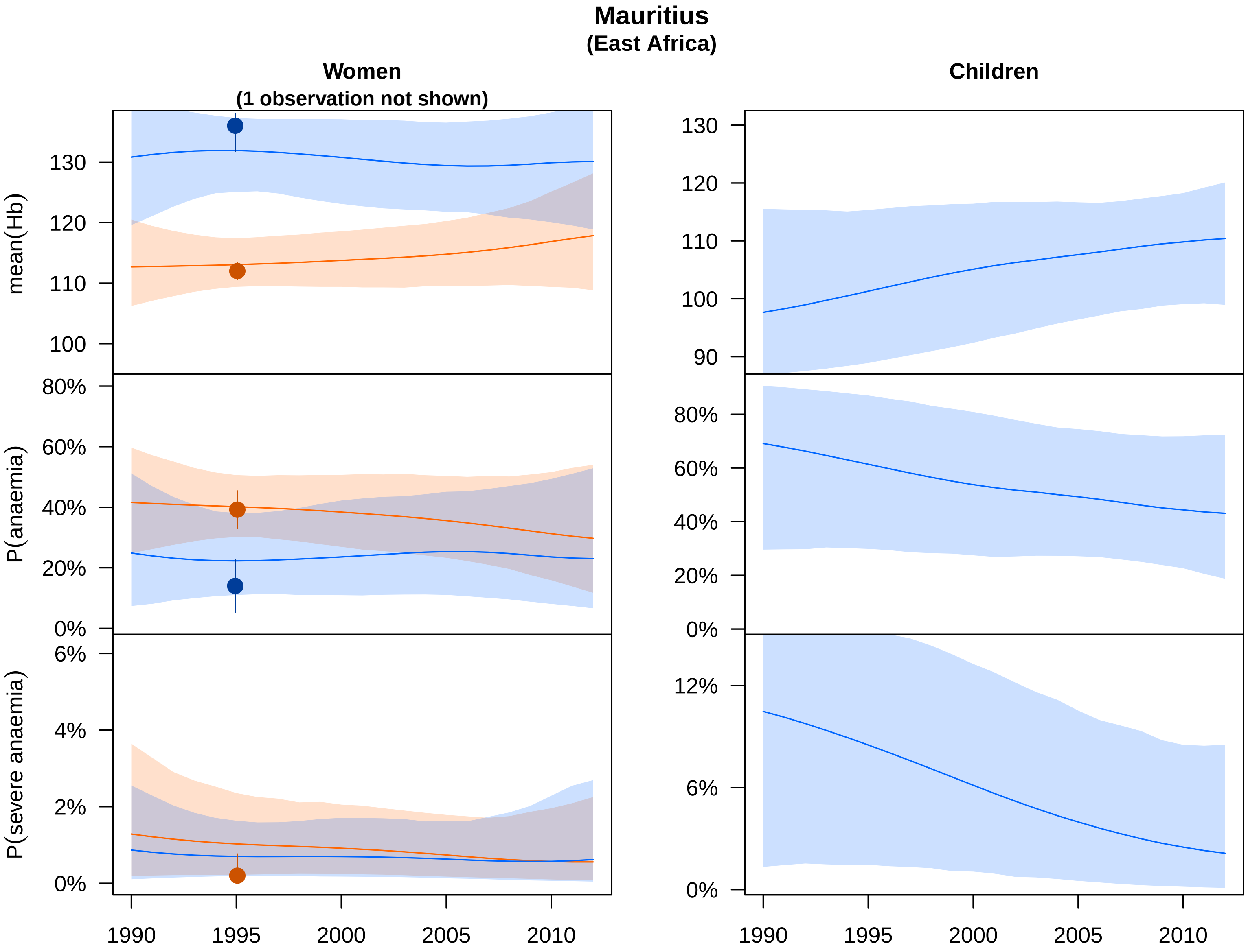
Mauritius
(East Africa)
Women
(1 observation not shown)
Children
mean(Hb)
P(anaemia)
P(severe anaemia)
130
120
110
100
90
80%
60%
40%
20%
0%
6%
4%
2%
12%
1990
1995
2000
2005
2010

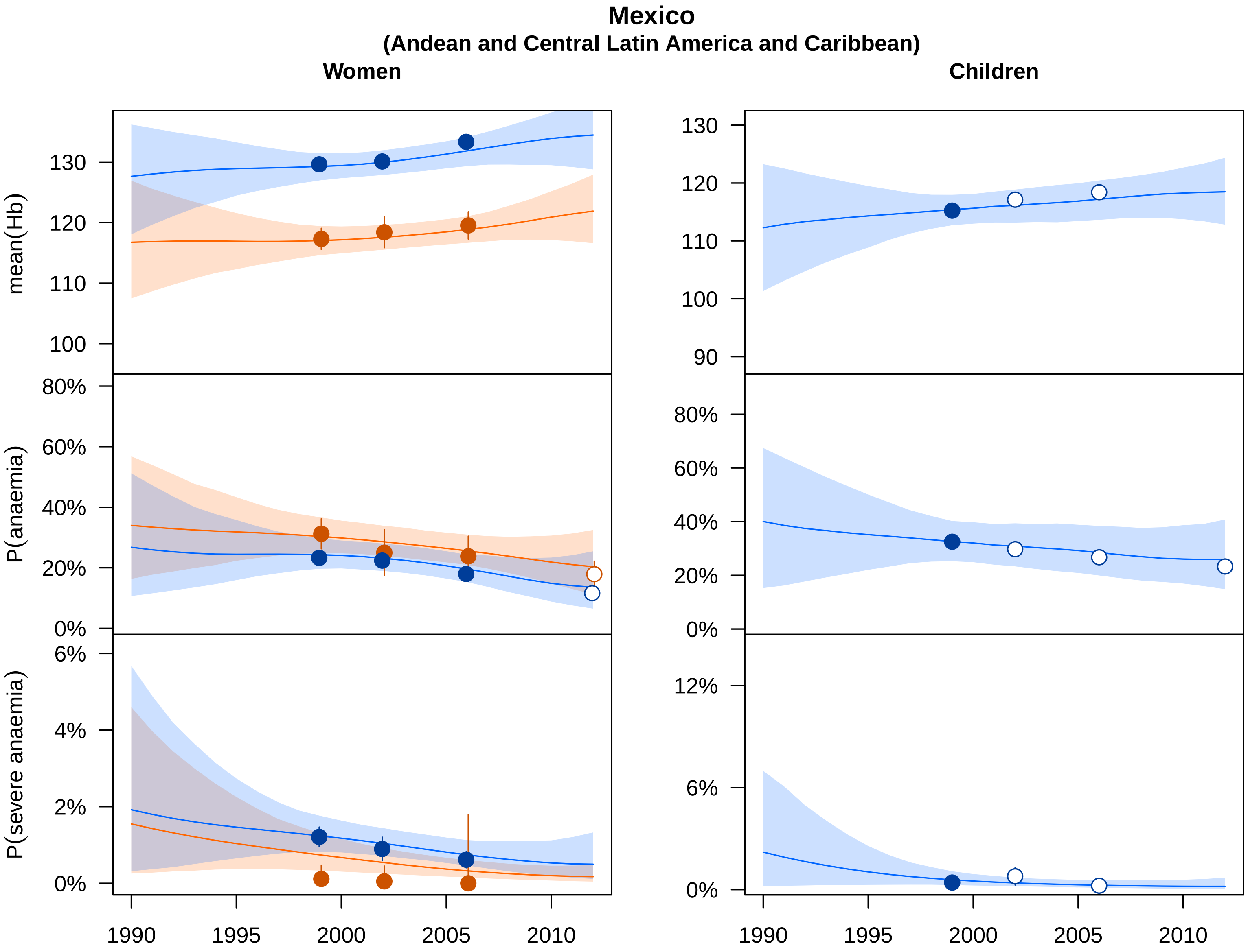
Mexico
(Andean and Central Latin America and Caribbean)
Women
Children
mean(Hb)
P(anaemia)
P(severe anaemia)
130
120
110
100
90
80%
60%
40%
20%
0%
6%
4%
2%
12%
1990
1995
2000
2005
2010

# Micronesia (Federated States of)
## (Oceania)

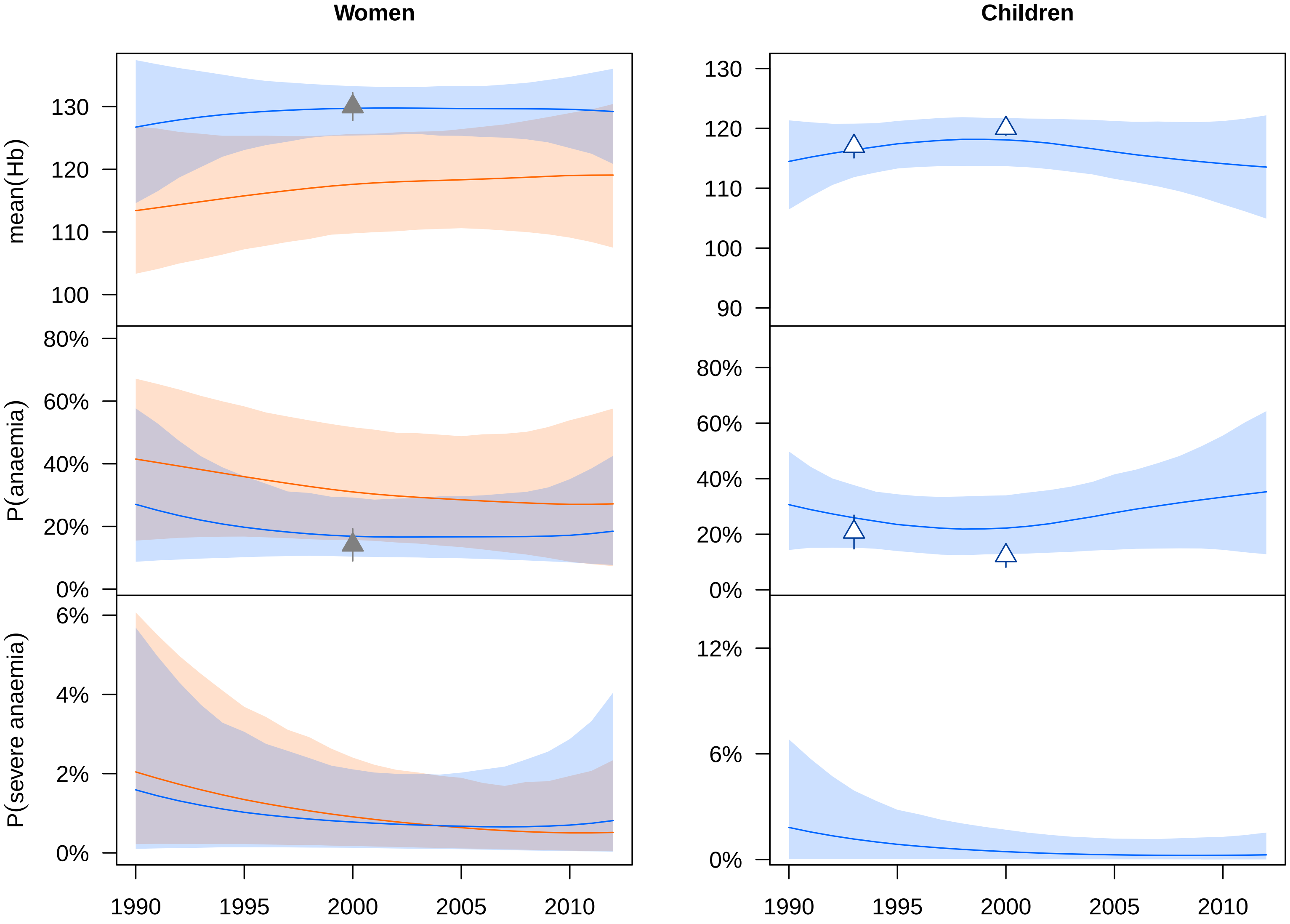

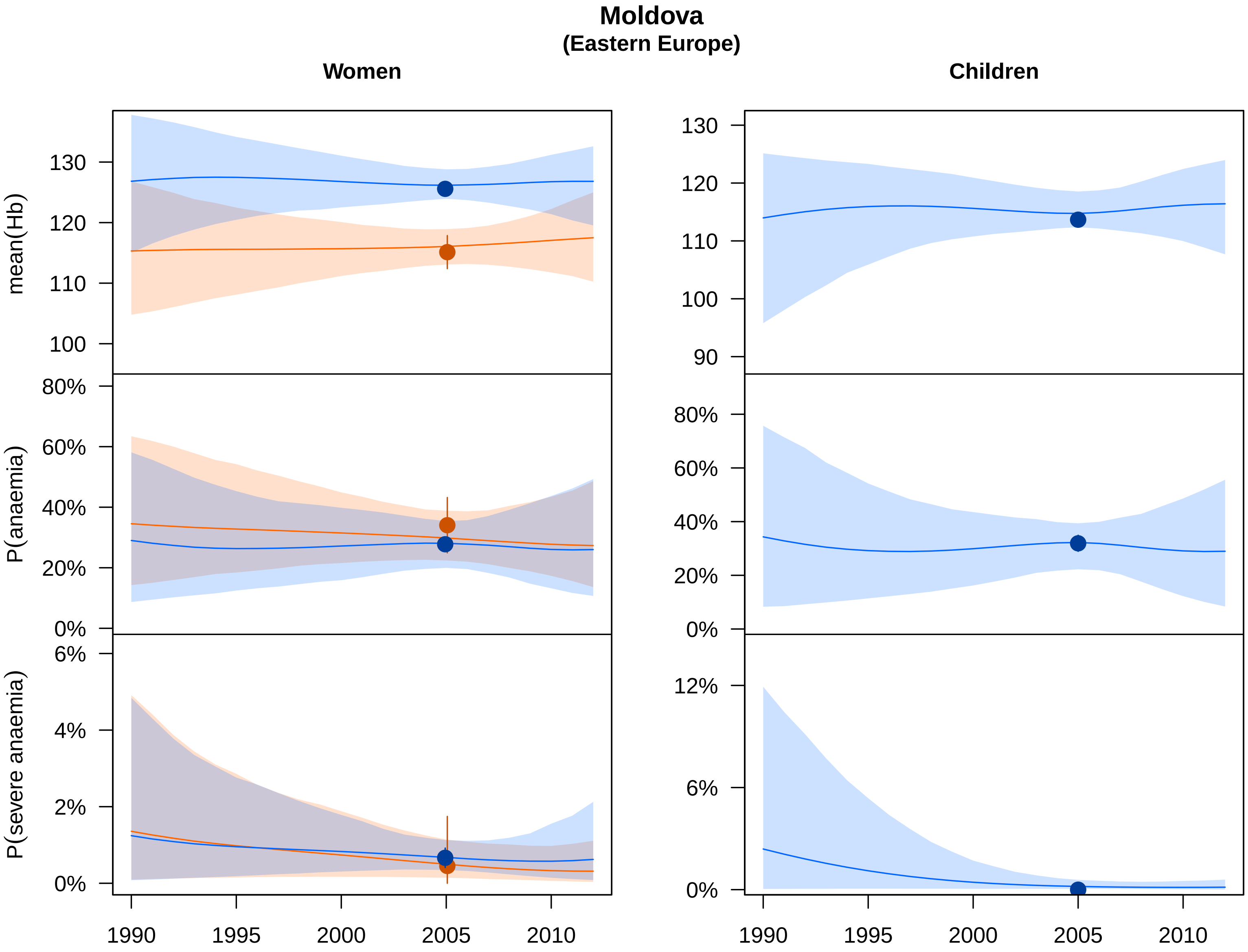
Moldova
(Eastern Europe)
Women
Children
mean(Hb)
P(anaemia)
P(severe anaemia)
130
120
110
100
90
80%
60%
40%
20%
0%
6%
4%
2%
12%
1990
1995
2000
2005
2010

# Mongolia
## (Central Asia, Middle East, and North Africa)

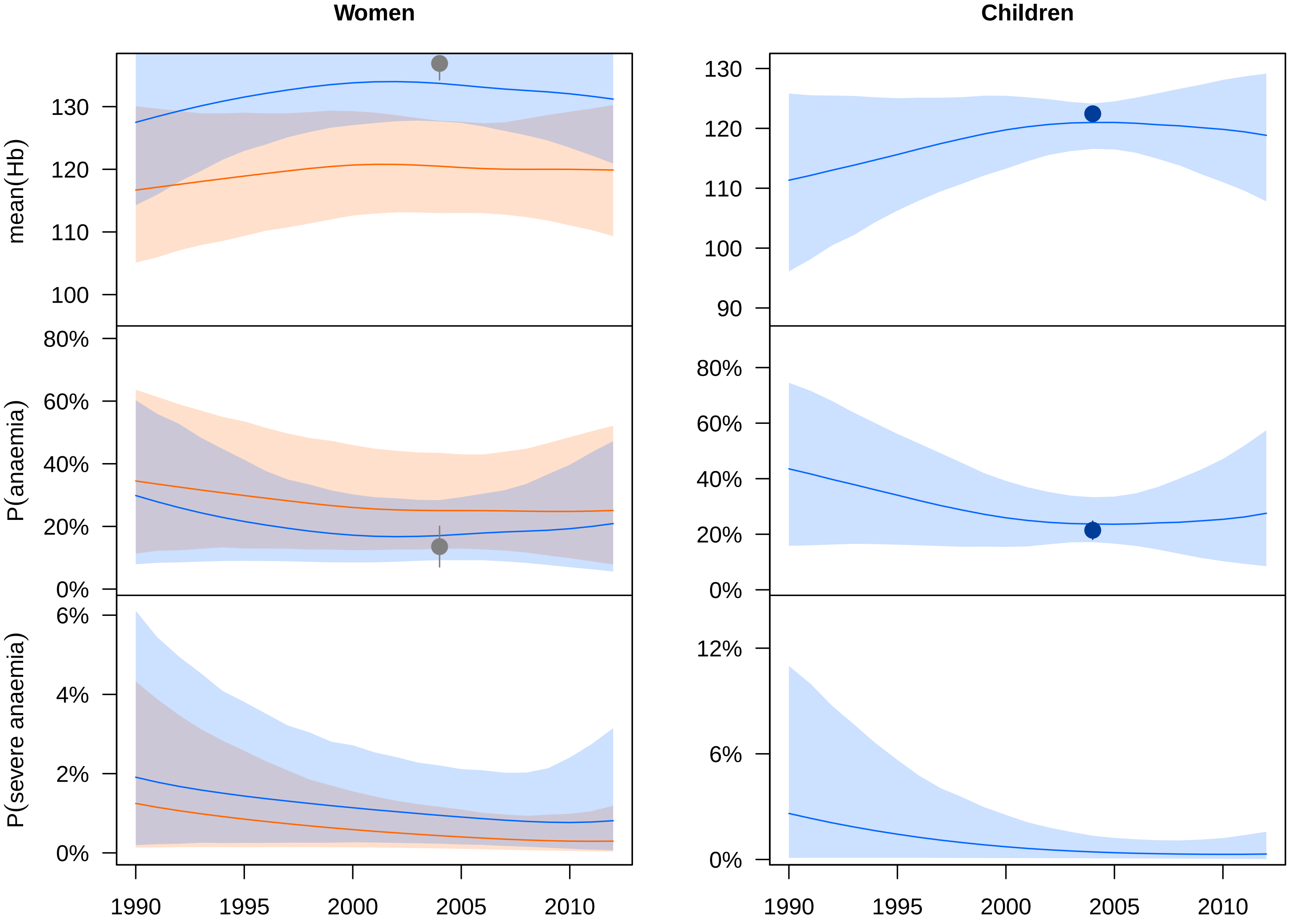

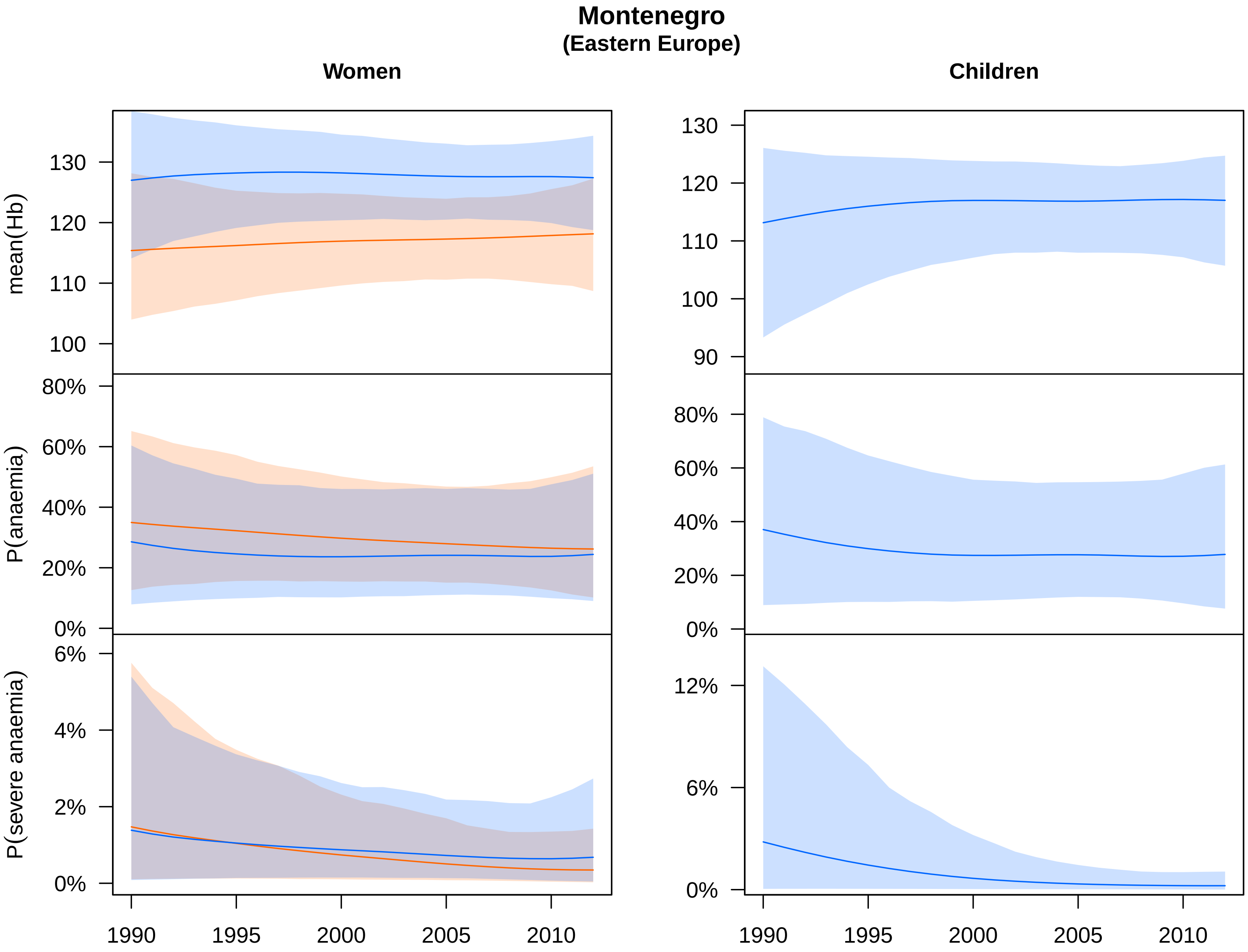
Montenegro
(Eastern Europe)
Women
Children
mean(Hb)
P(anaemia)
P(severe anaemia)
130
120
110
100
90
80%
60%
40%
20%
0%
6%
4%
2%
12%
1990
1995
2000
2005
2010

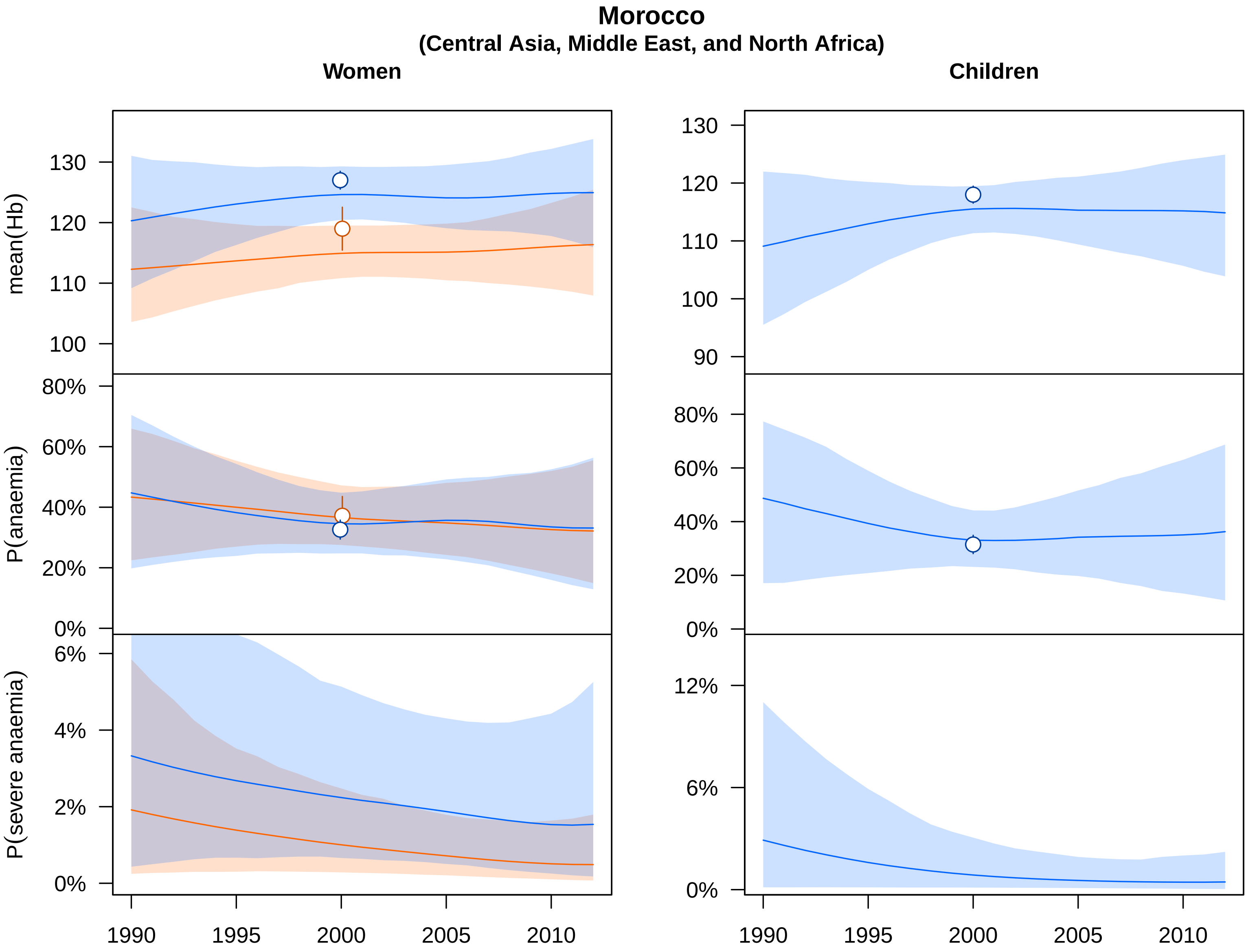

Morocco
(Central Asia, Middle East, and North Africa)
Women
Children
mean(Hb)
P(anaemia)
P(severe anaemia)
130
120
110
100
90
80%
60%
40%
20%
0%
6%
4%
2%
12%
1990
1995
2000
2005
2010

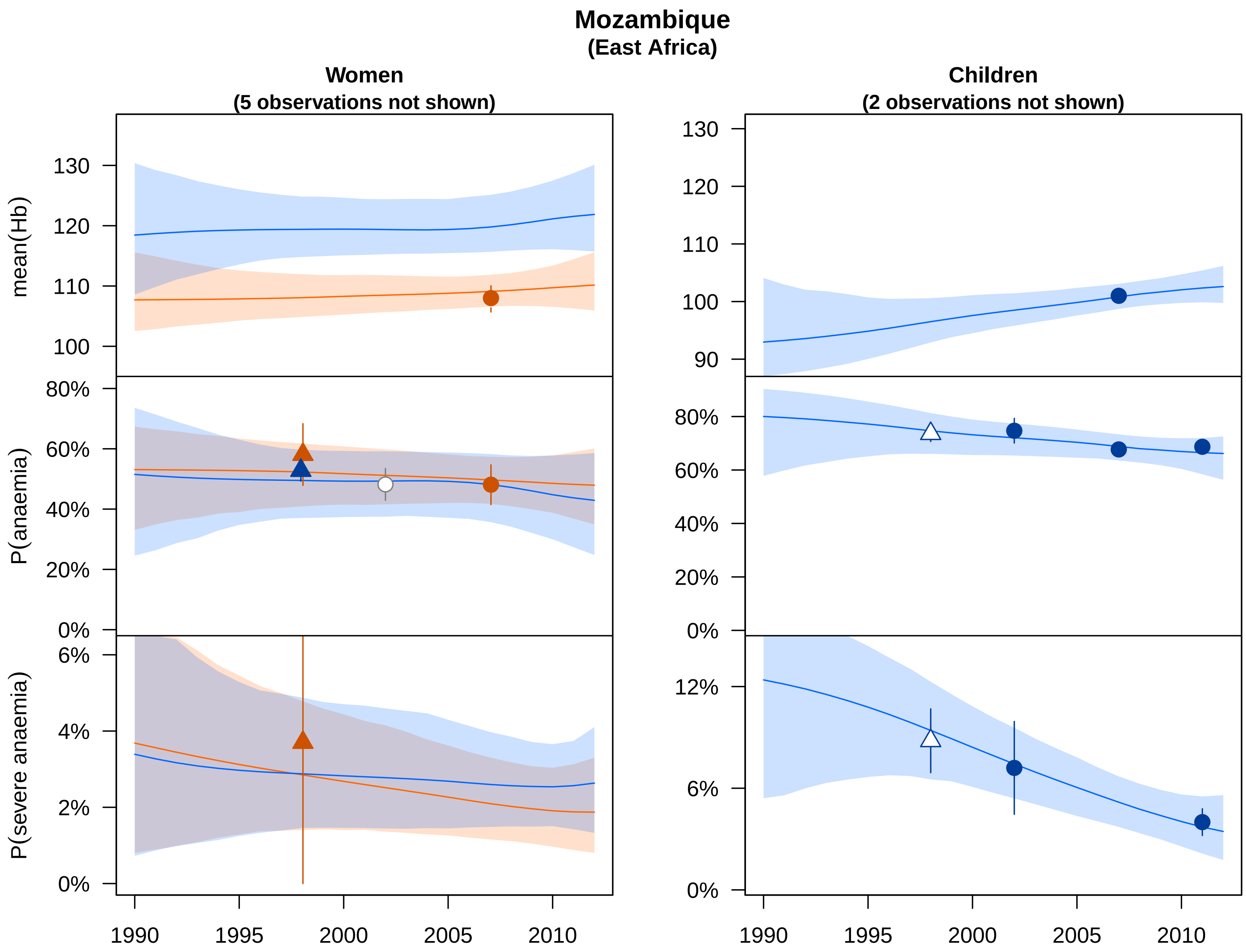
Mozambique
(East Africa)
Women
(5 observations not shown)
Children
(2 observations not shown)
mean(Hb)
P(anaemia)
P(severe anaemia)
130
120
110
100
90
80%
60%
40%
20%
0%
6%
4%
2%
12%
1990
1995
2000
2005
2010

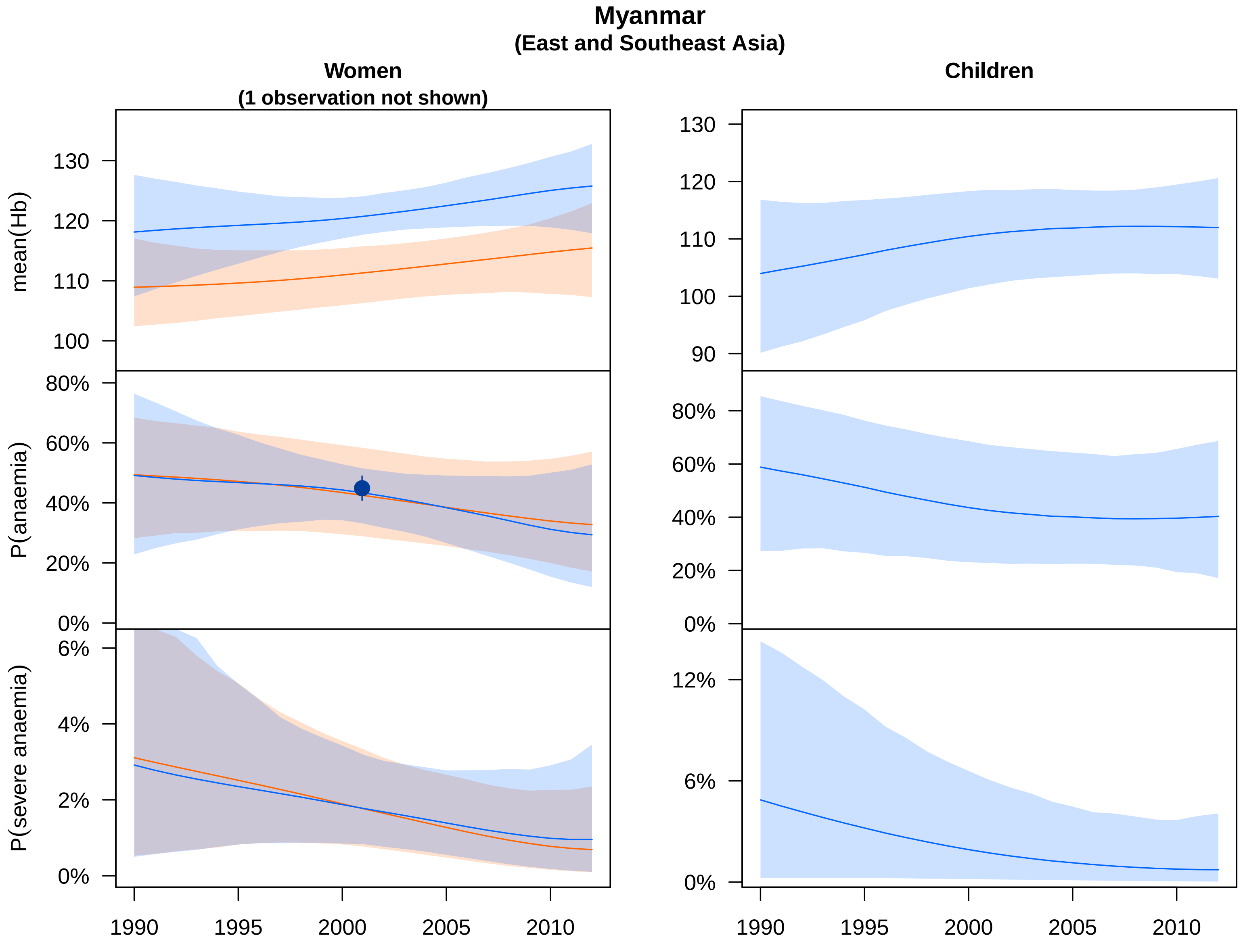
Myanmar
(East and Southeast Asia)
Women
(1 observation not shown)
Children
mean(Hb)
P(anaemia)
P(severe anaemia)
130
120
110
100
90
80%
60%
40%
20%
0%
6%
4%
2%
12%
1990
1995
2000
2005
2010

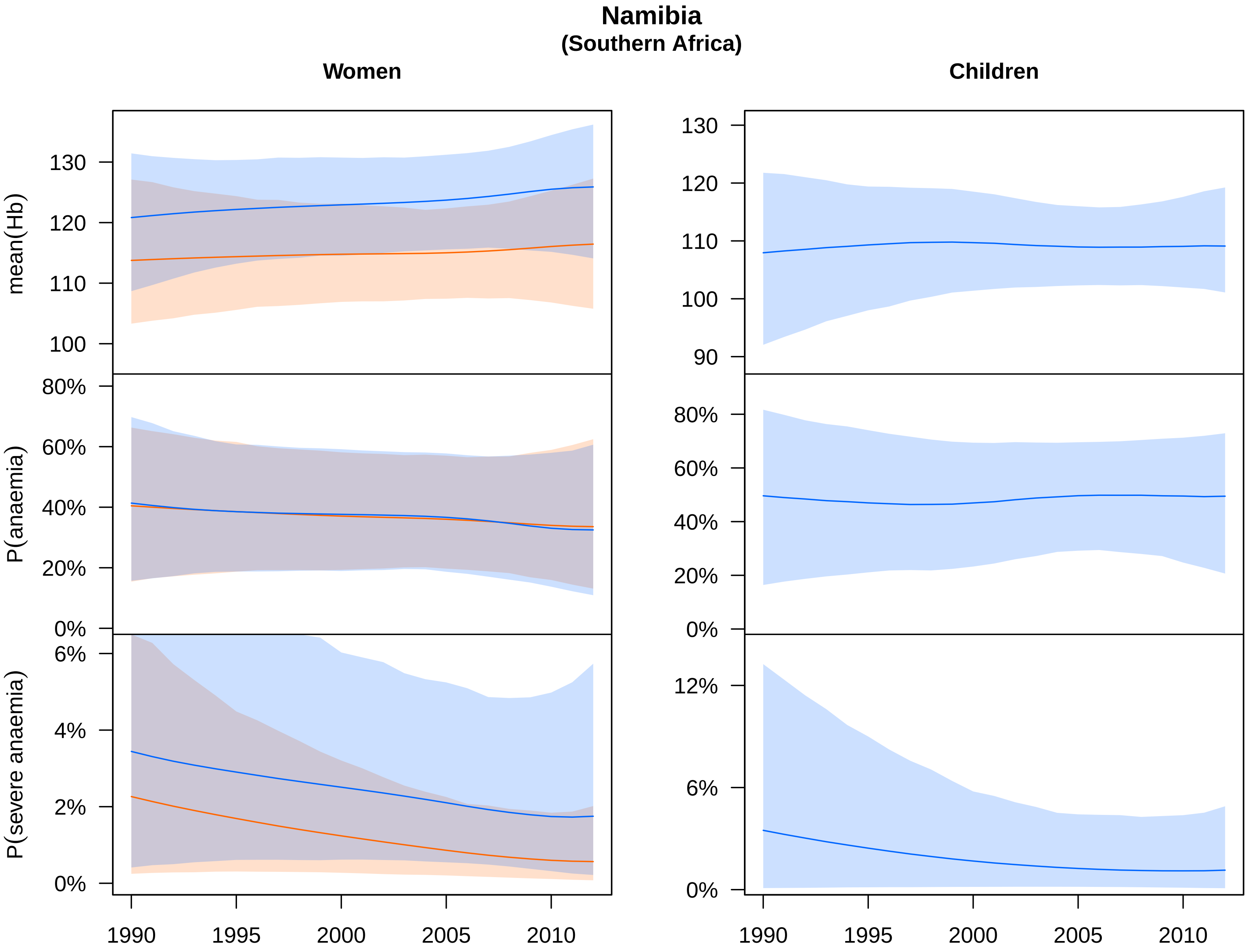

Namibia
(Southern Africa)
Women
Children
mean(Hb)
P(anaemia)
P(severe anaemia)
130
120
110
100
90
80%
60%
40%
20%
0%
6%
4%
2%
12%
1990
1995
2000
2005
2010

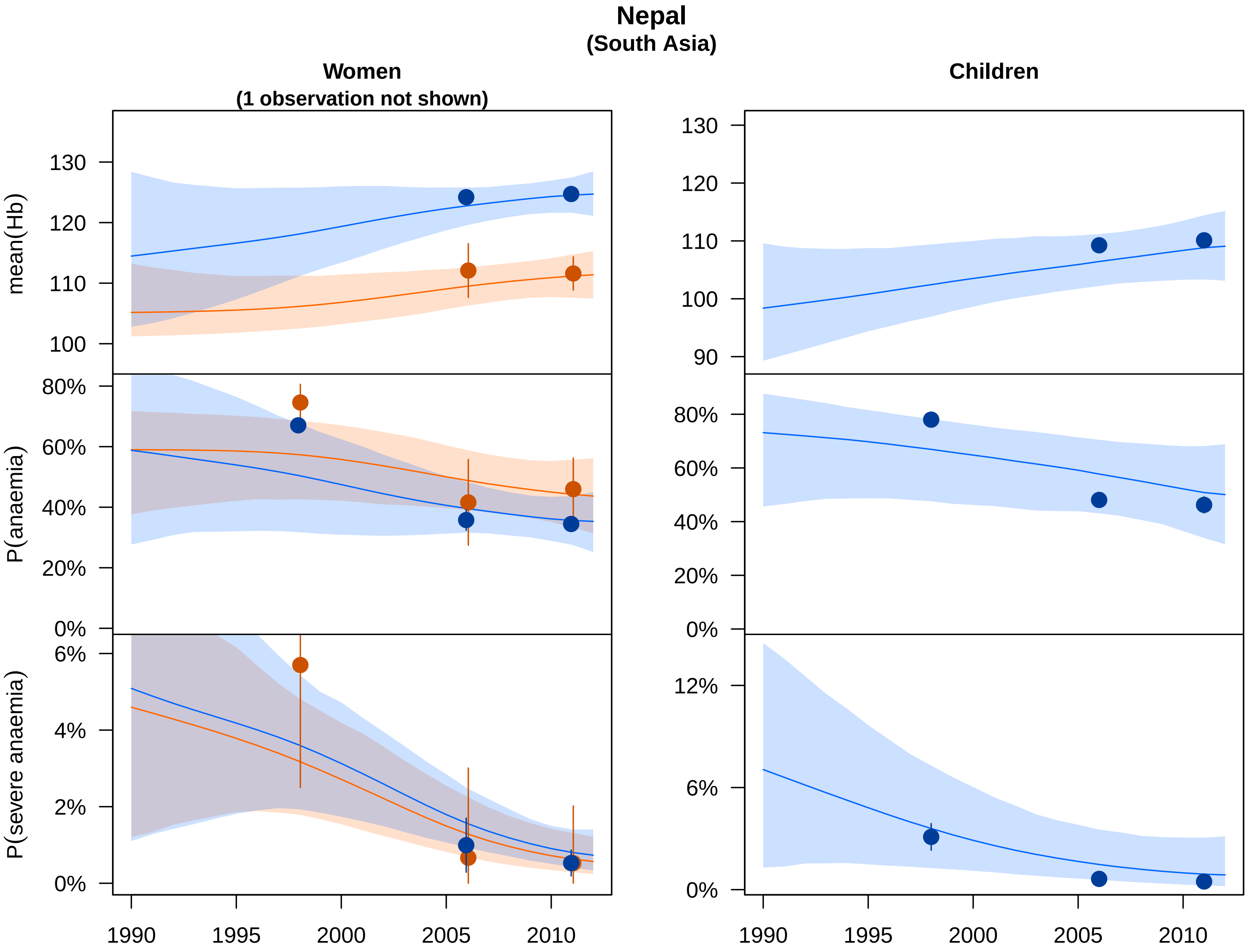
Nepal
(South Asia)
Women
(1 observation not shown)
Children
mean(Hb)
P(anaemia)
P(severe anaemia)
130
120
110
100
90
80%
60%
40%
20%
0%
6%
4%
2%
12%
1990
1995
2000
2005
2010

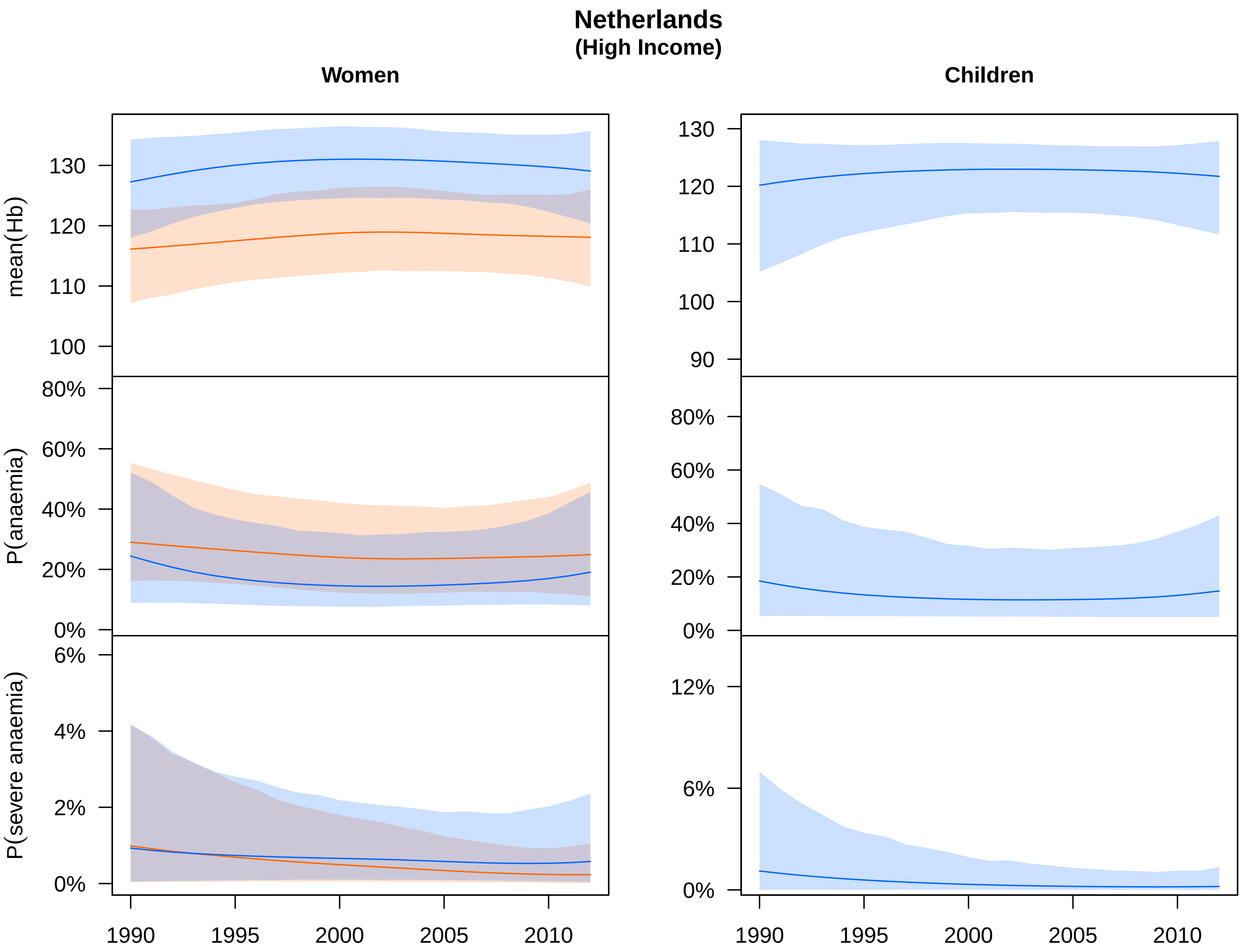
Netherlands
(High Income)
Women
Children
mean(Hb)
P(anaemia)
P(severe anaemia)
130
120
110
100
90
80%
60%
40%
20%
0%
6%
4%
2%
12%
1990
1995
2000
2005
2010

# New Zealand
## (High Income)

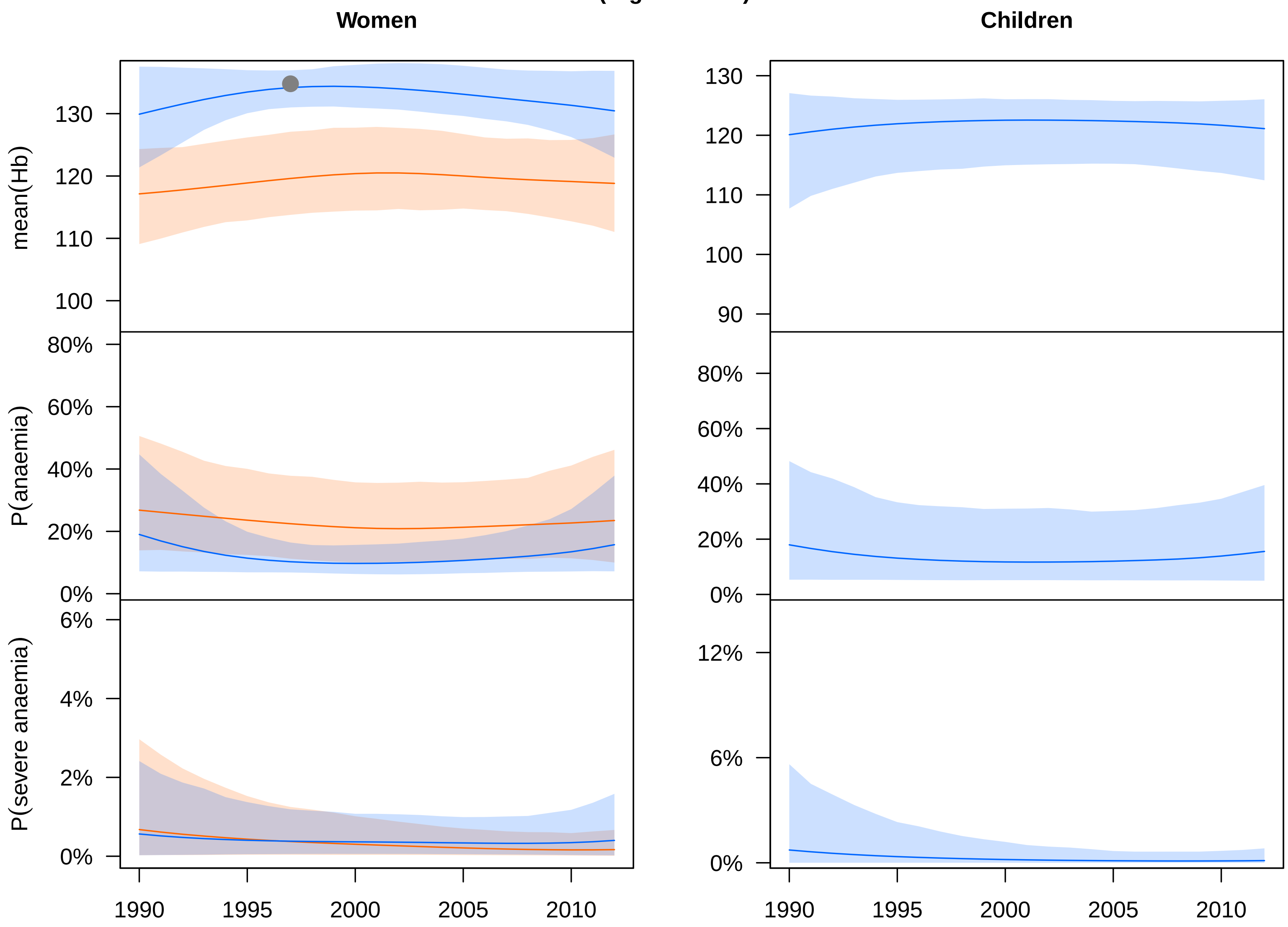

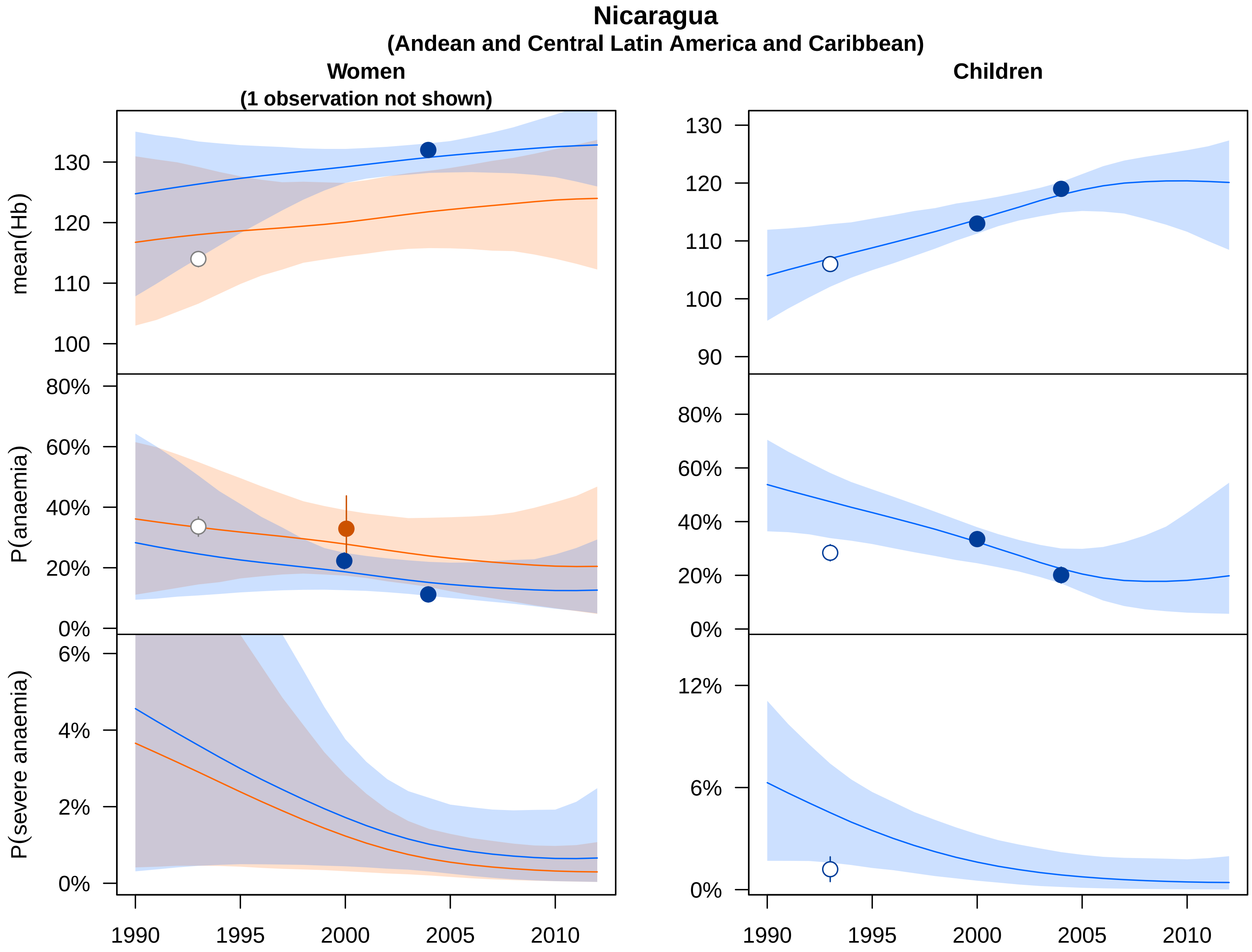
Nicaragua
(Andean and Central Latin America and Caribbean)
Women
(1 observation not shown)
Children
mean(Hb)
P(anaemia)
P(severe anaemia)
130
120
110
100
90
80%
60%
40%
20%
0%
6%
4%
2%
12%
1990
1995
2000
2005
2010

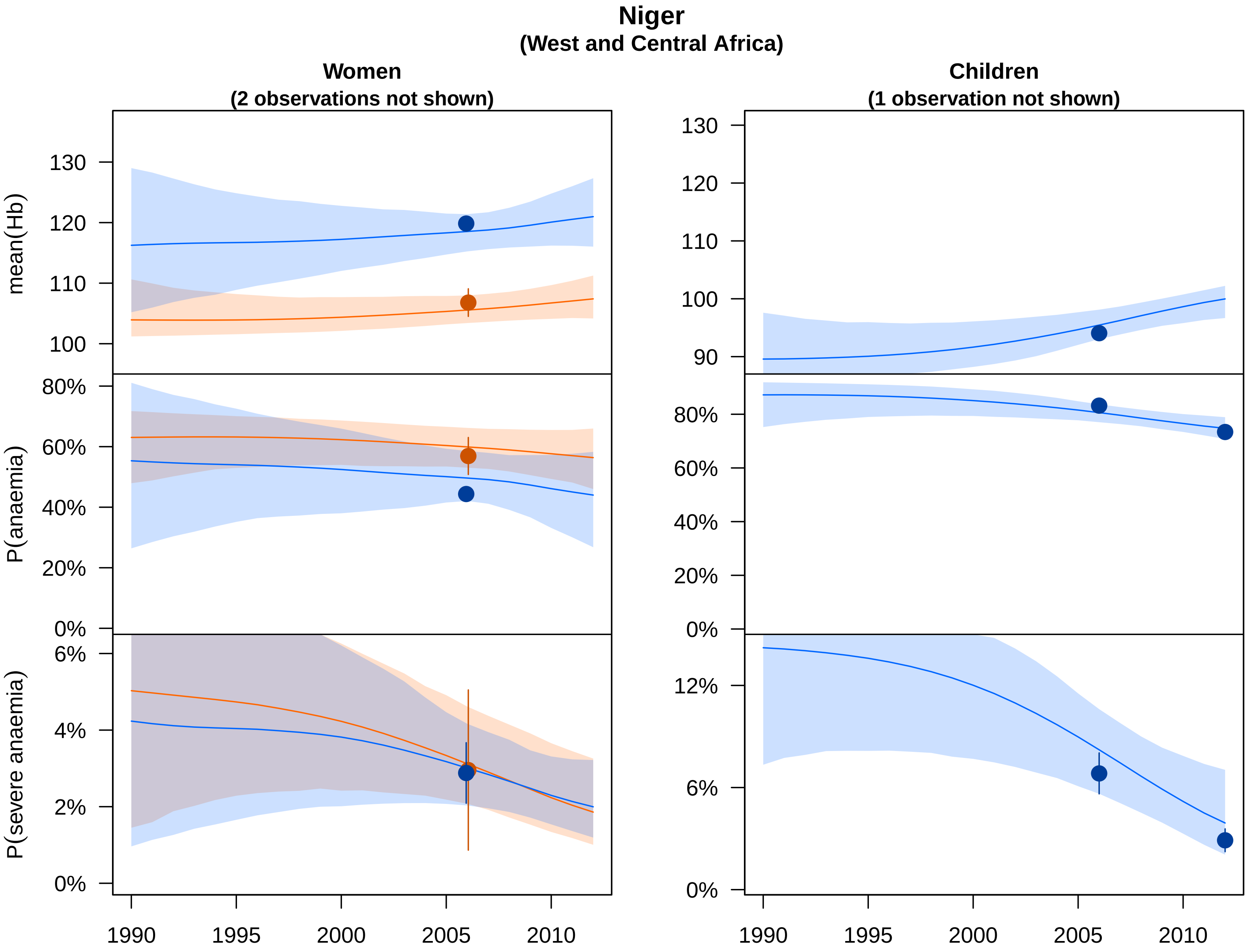
Niger
(West and Central Africa)
Women
(2 observations not shown)
Children
(1 observation not shown)
mean(Hb)
P(anaemia)
P(severe anaemia)
130
120
110
100
90
80%
60%
40%
20%
0%
6%
4%
2%
12%
1990
1995
2000
2005
2010

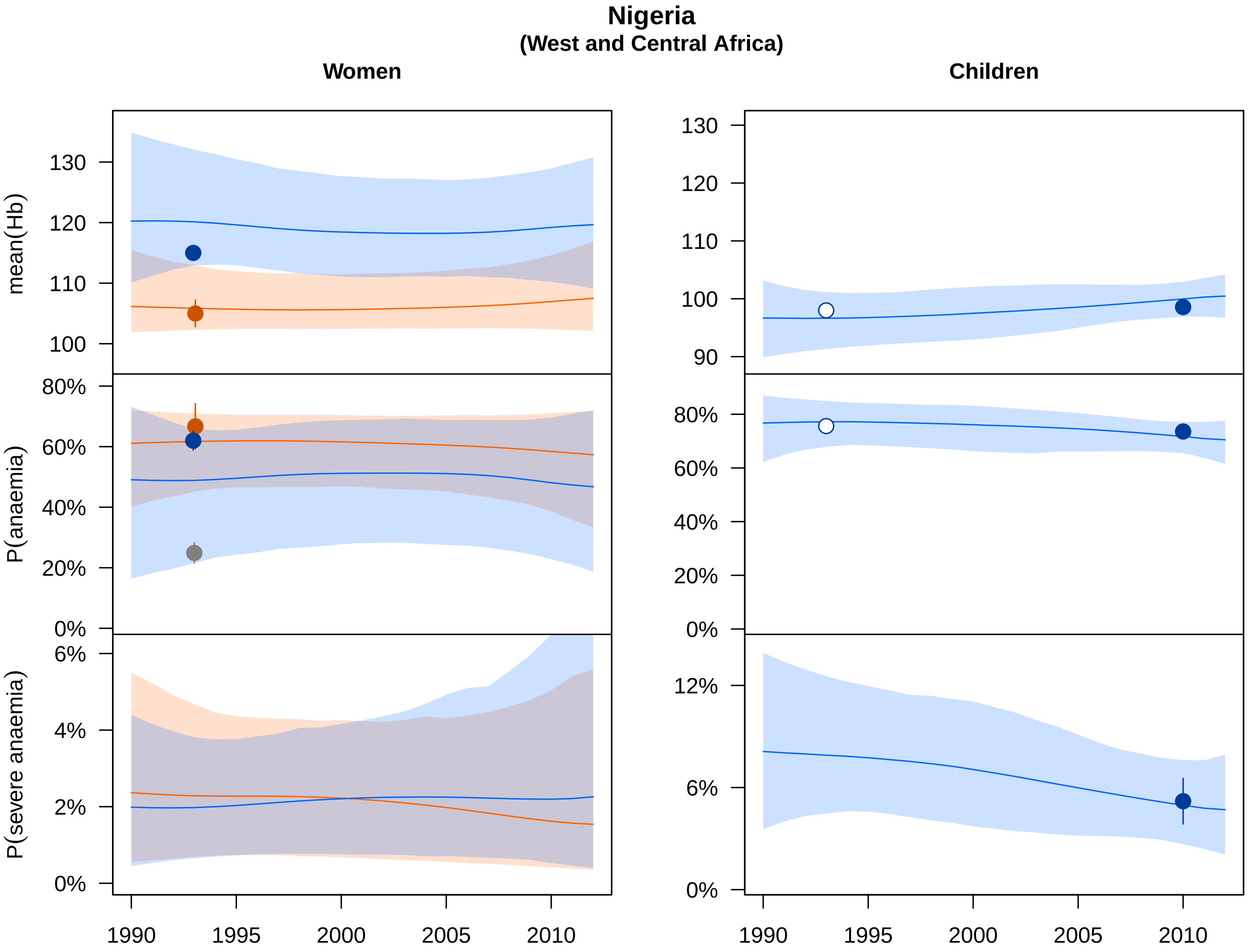
Nigeria
(West and Central Africa)
Women
Children
mean(Hb)
P(anaemia)
P(severe anaemia)
130
120
110
100
90
80%
60%
40%
20%
0%
6%
4%
2%
12%
1990
1995
2000
2005
2010

**Norway**
**(High Income)**

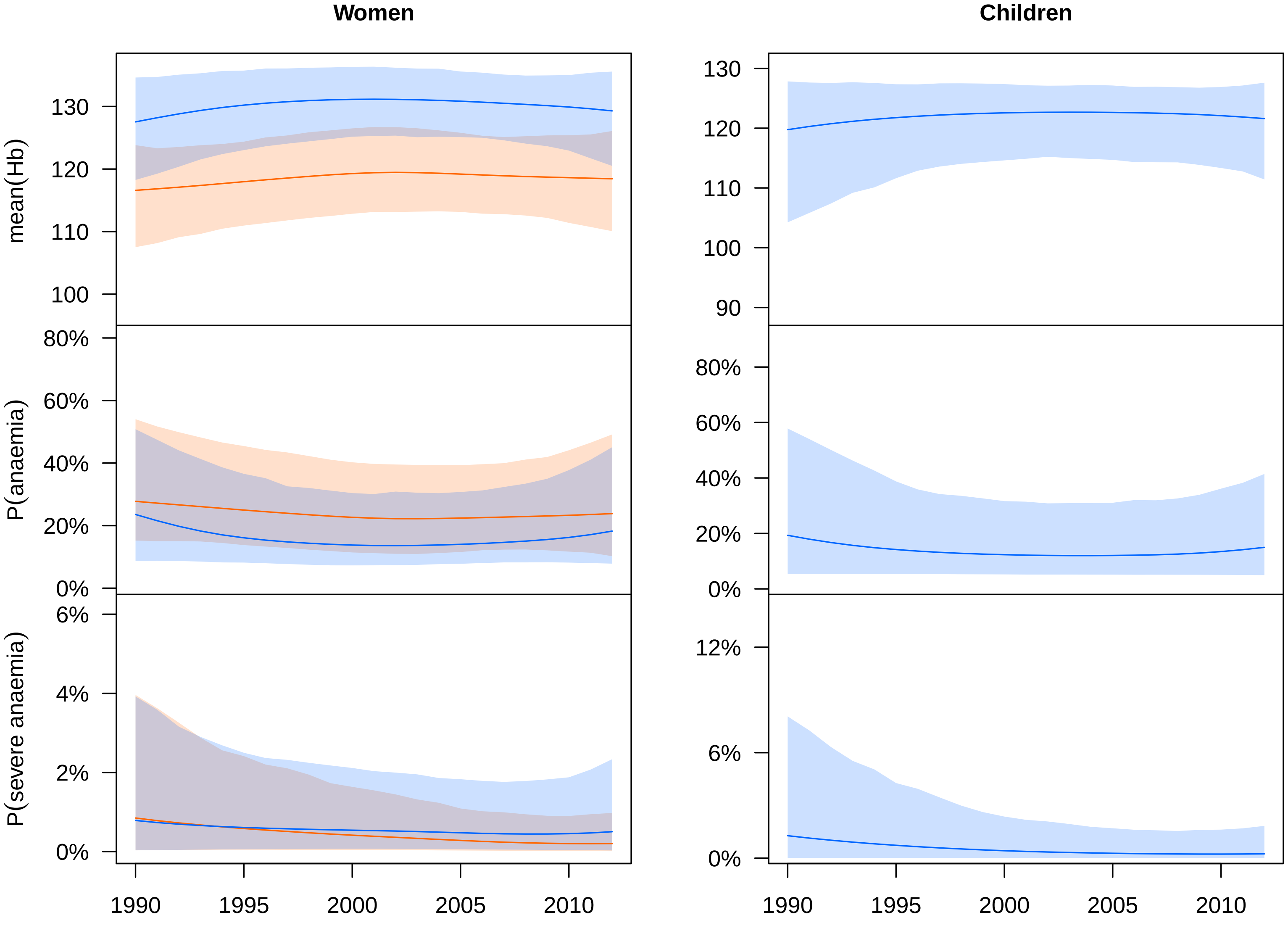

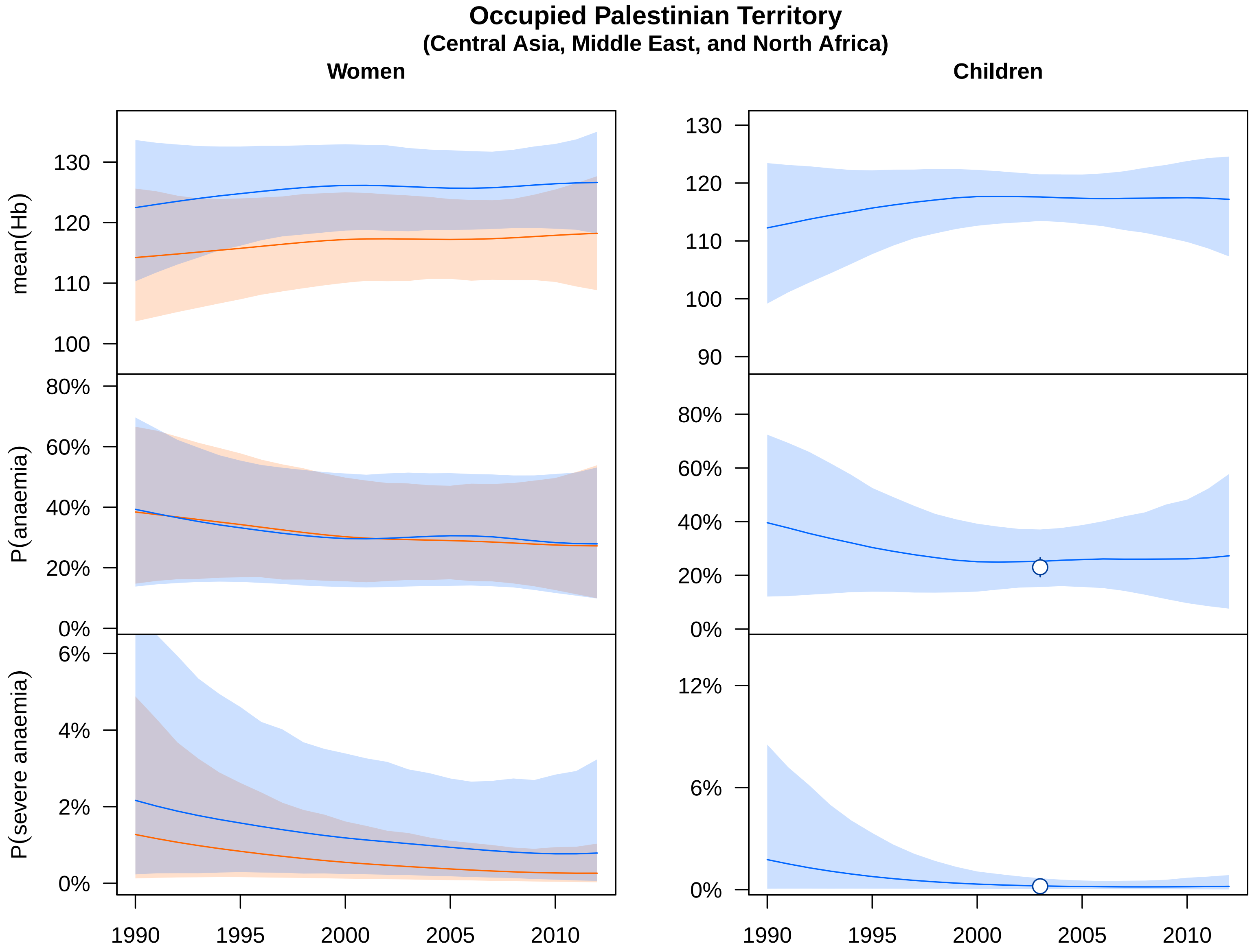
Occupied Palestinian Territory
(Central Asia, Middle East, and North Africa)
Women
Children
mean(Hb)
P(anaemia)
P(severe anaemia)
100
110
120
130
90
0%
20%
40%
60%
80%
2%
4%
6%
12%
1990
1995
2000
2005
2010

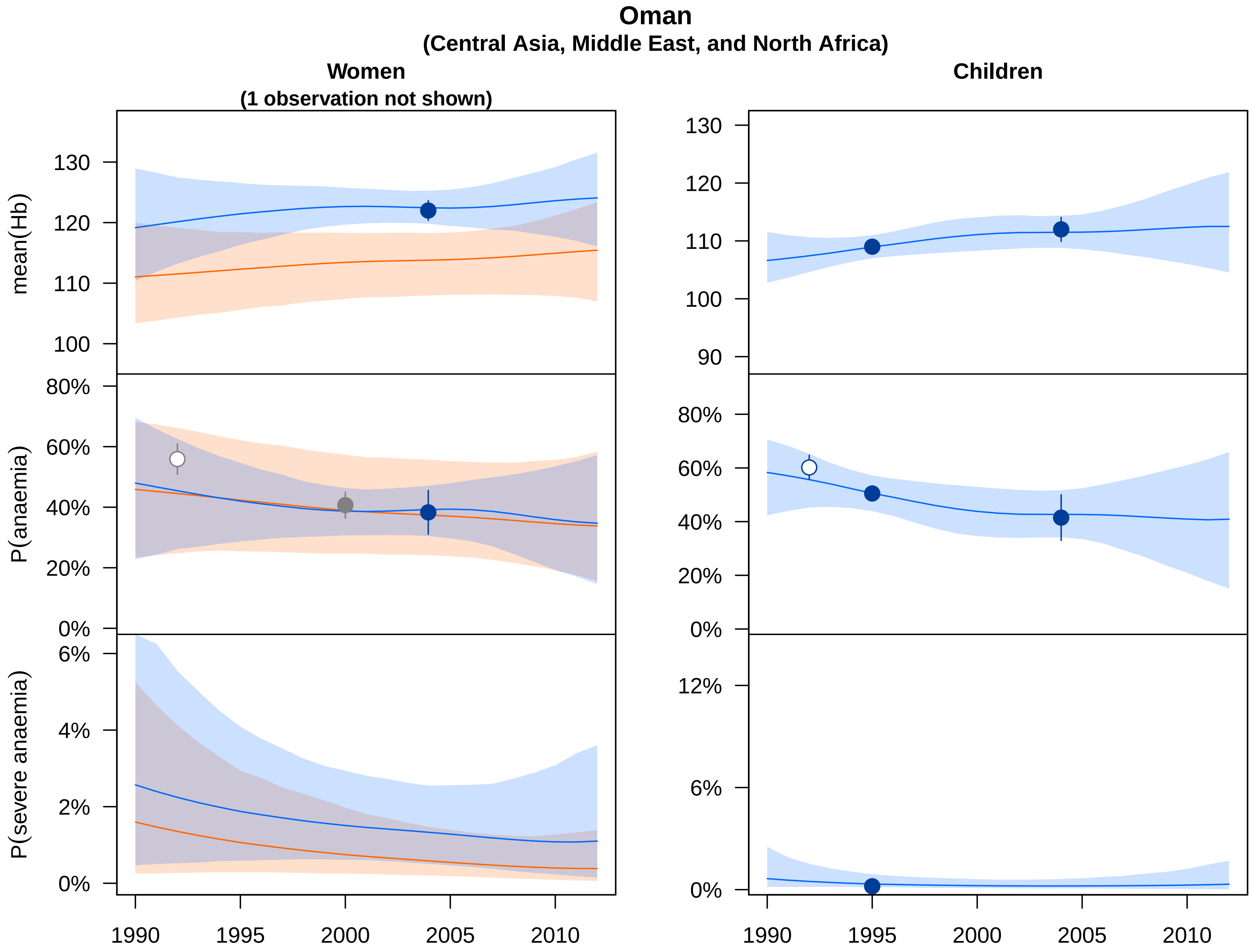
Oman
(Central Asia, Middle East, and North Africa)
Women
(1 observation not shown)
Children
mean(Hb)
P(anaemia)
P(severe anaemia)
130
120
110
100
90
80%
60%
40%
20%
0%
6%
4%
2%
12%
1990
1995
2000
2005
2010

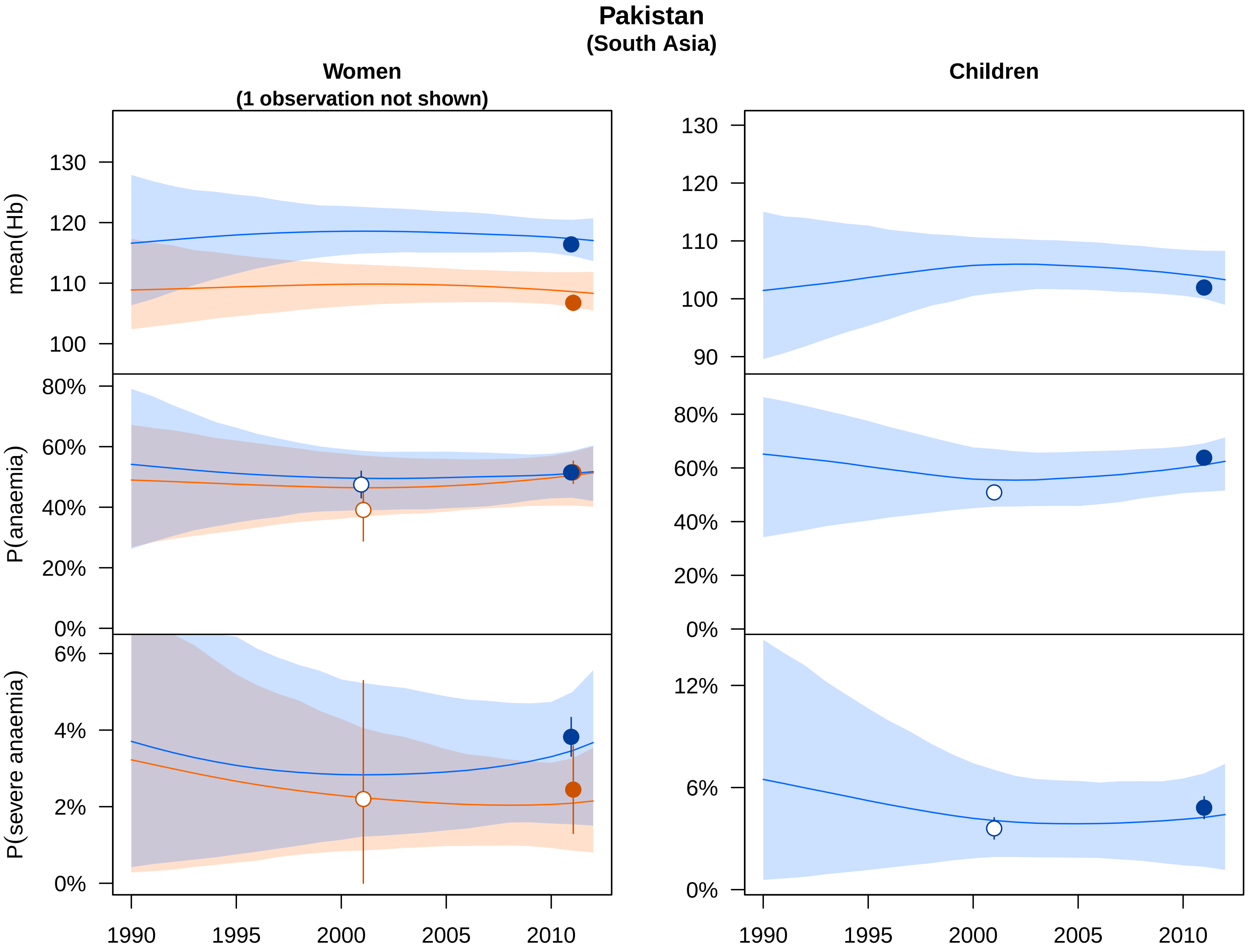
Pakistan
(South Asia)
Women
(1 observation not shown)
Children
mean(Hb)
P(anaemia)
P(severe anaemia)
130
120
110
100
90
80%
60%
40%
20%
0%
6%
4%
2%
12%
1990
1995
2000
2005
2010

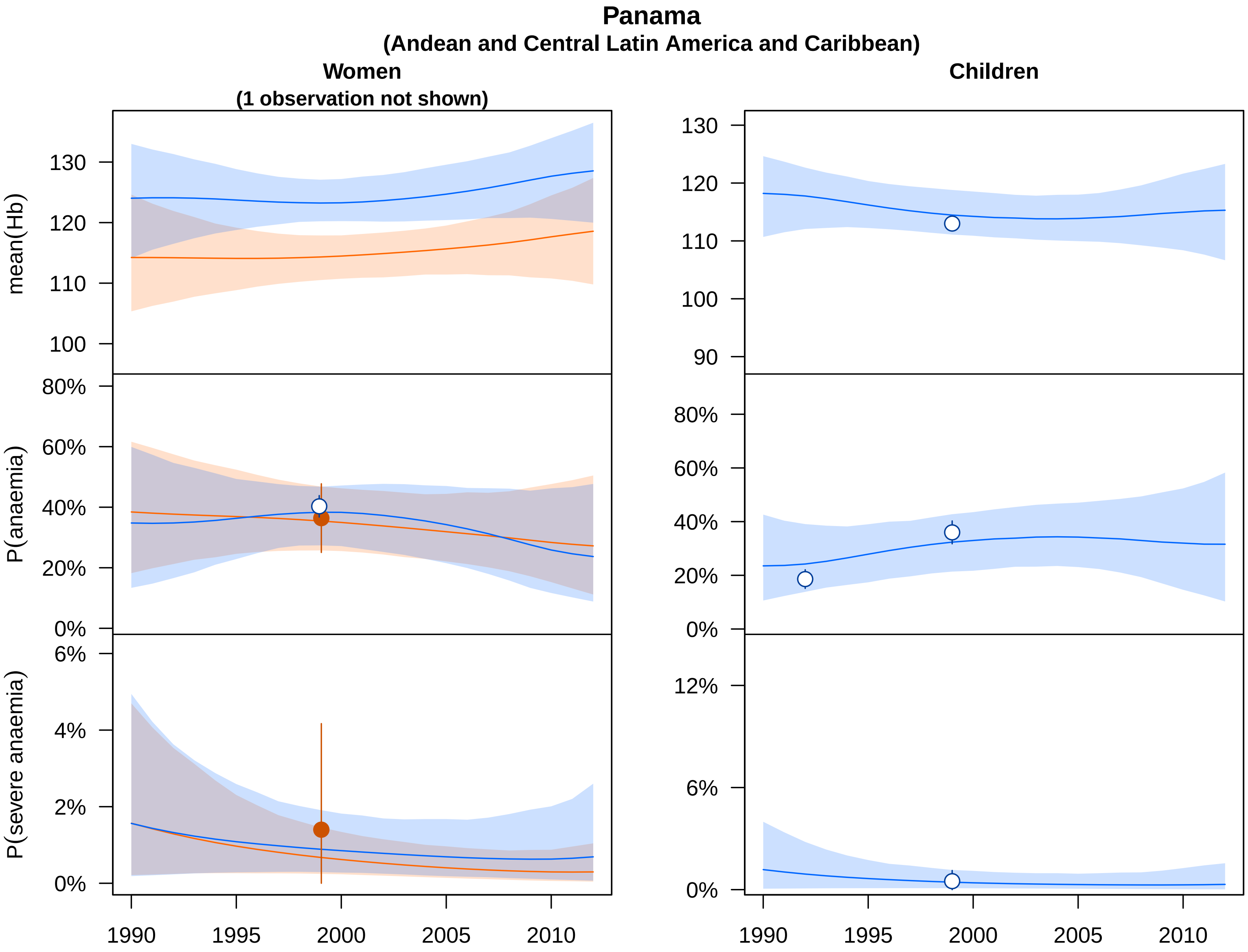
Panama
(Andean and Central Latin America and Caribbean)
Women
(1 observation not shown)
Children
mean(Hb)
P(anaemia)
P(severe anaemia)
130
120
110
100
90
80%
60%
40%
20%
0%
6%
4%
2%
12%
1990
1995
2000
2005
2010

# Papua New Guinea
## (Oceania)

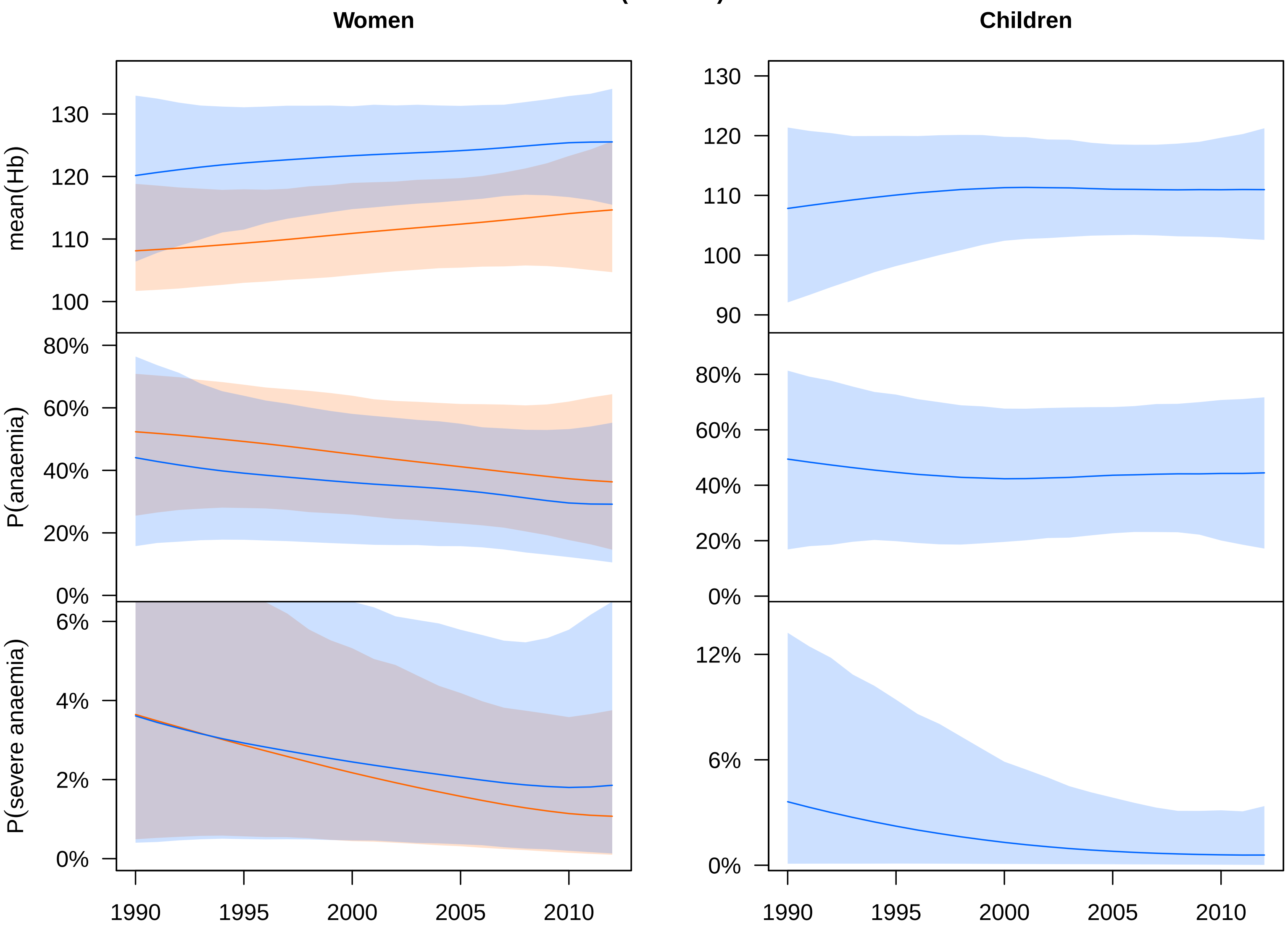

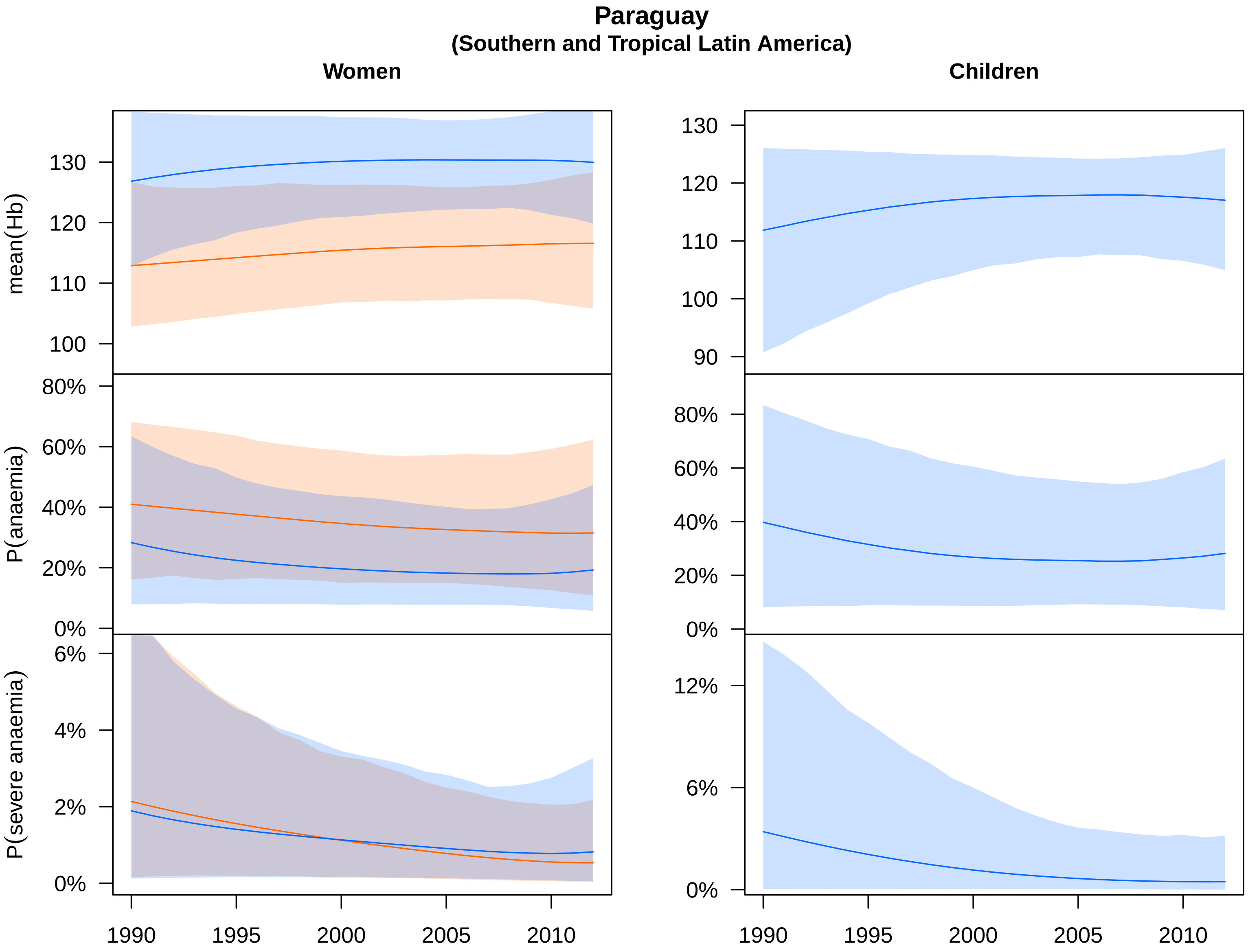

Paraguay
(Southern and Tropical Latin America)
Women
Children
mean(Hb)
P(anaemia)
P(severe anaemia)
100
110
120
130
90
0%
20%
40%
60%
80%
2%
4%
6%
12%
1990
1995
2000
2005
2010

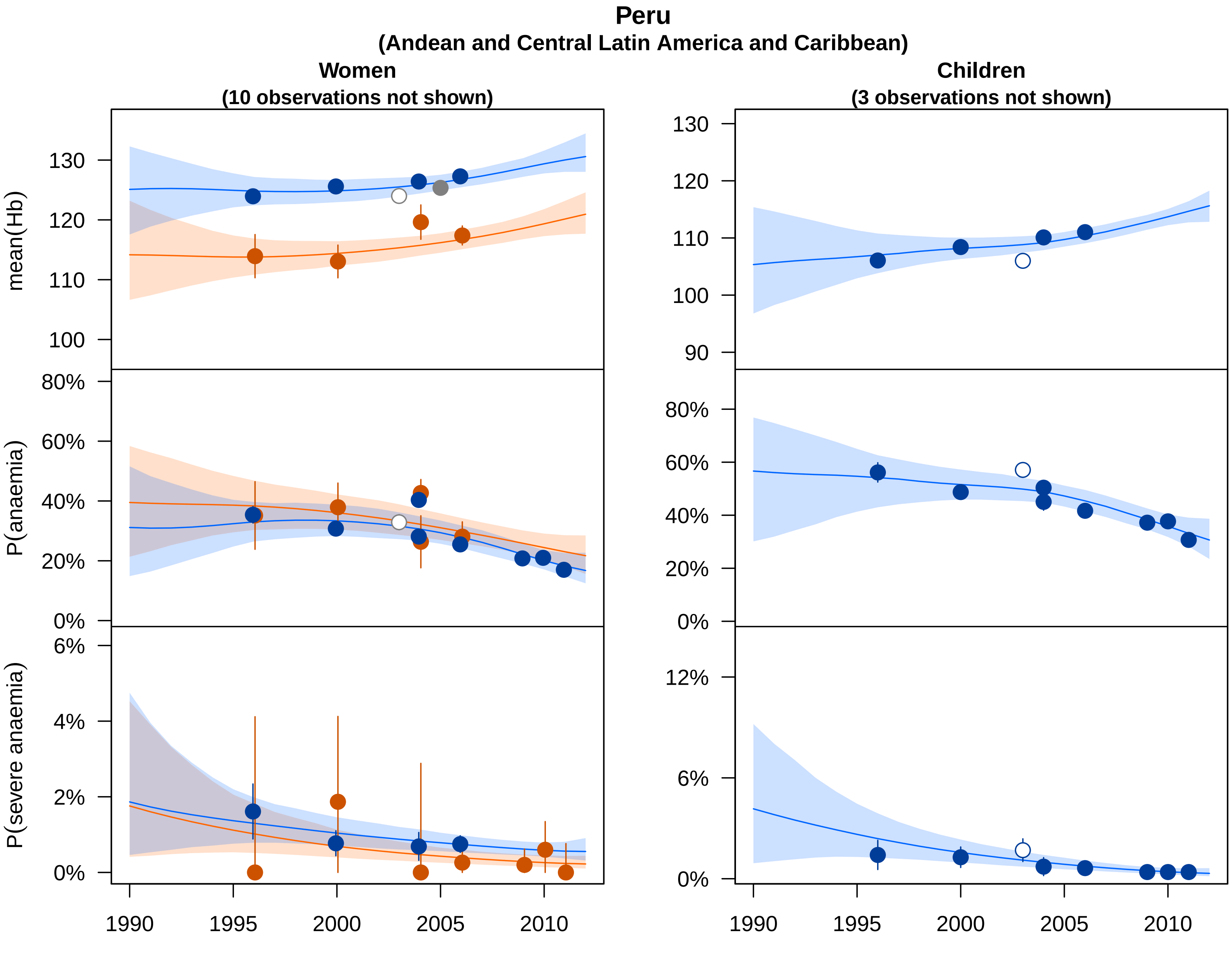
Peru
(Andean and Central Latin America and Caribbean)
Women
(10 observations not shown)
Children
(3 observations not shown)
mean(Hb)
P(anaemia)
P(severe anaemia)
130
120
110
100
90
80%
60%
40%
20%
0%
6%
4%
2%
12%
1990
1995
2000
2005
2010

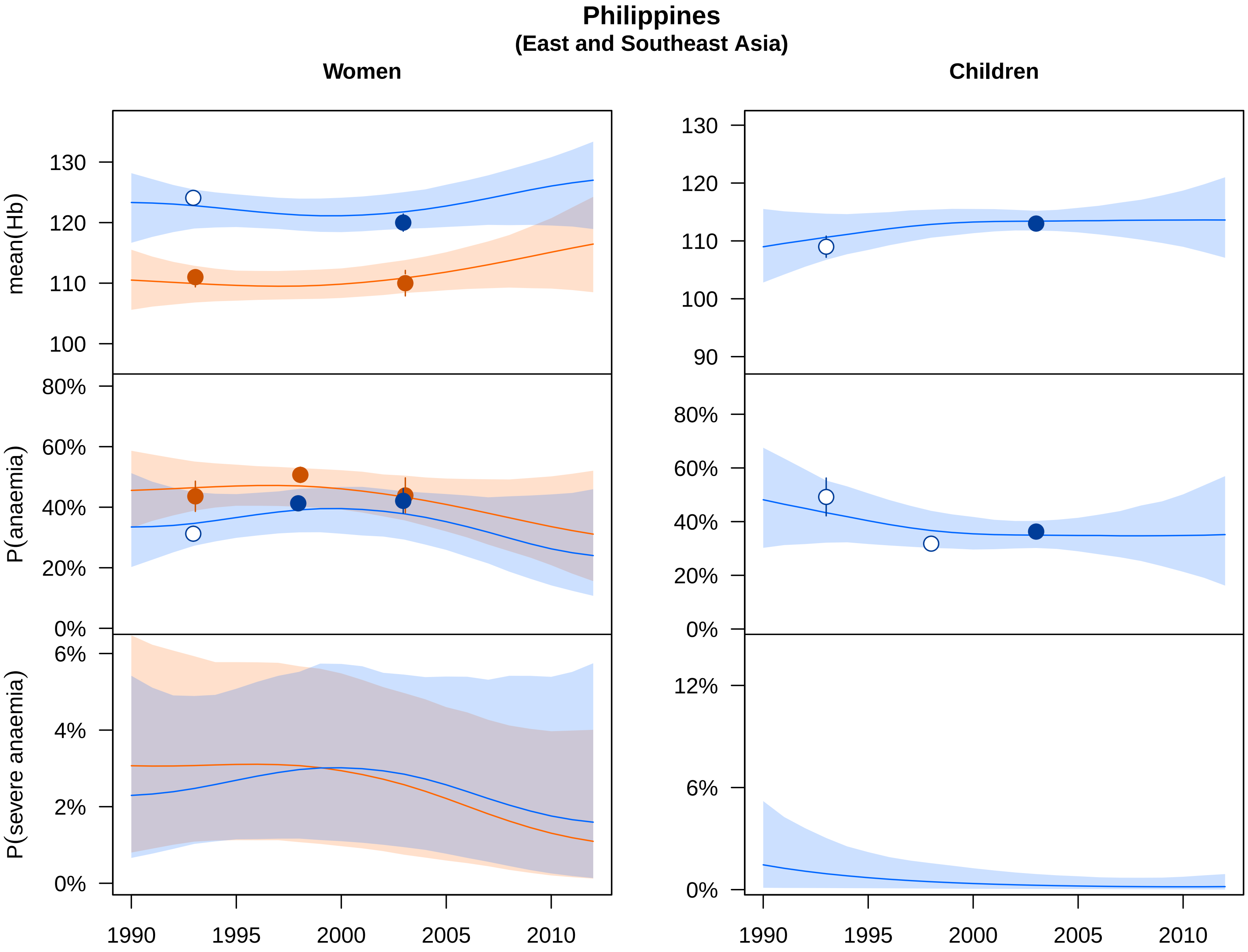
Philippines
(East and Southeast Asia)
Women
Children
mean(Hb)
P(anaemia)
P(severe anaemia)
130
120
110
100
90
80%
60%
40%
20%
0%
6%
4%
2%
12%
1990
1995
2000
2005
2010

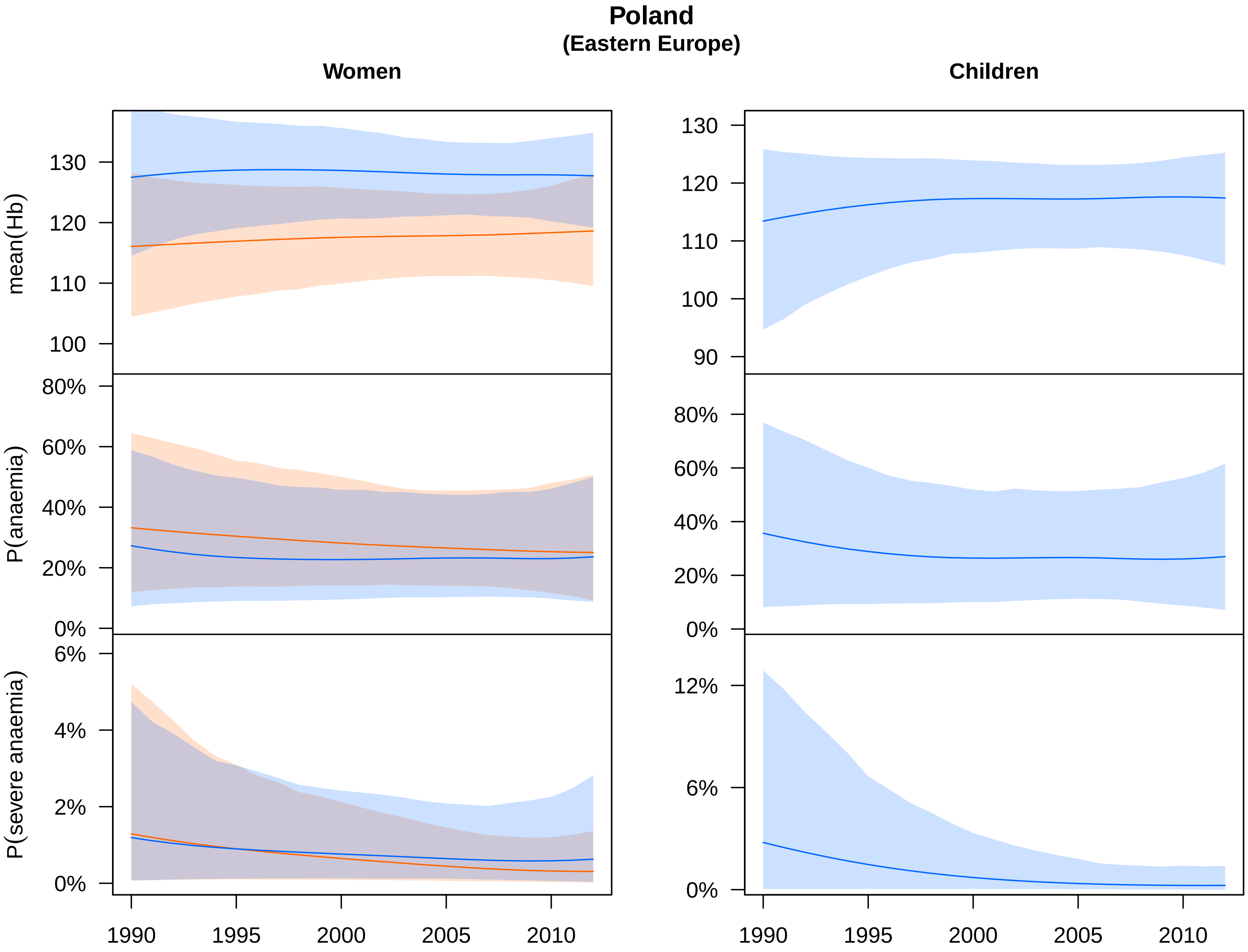

Poland
(Eastern Europe)
Women
Children
mean(Hb)
P(anaemia)
P(severe anaemia)
130
120
110
100
90
80%
60%
40%
20%
0%
6%
4%
2%
12%
1990
1995
2000
2005
2010

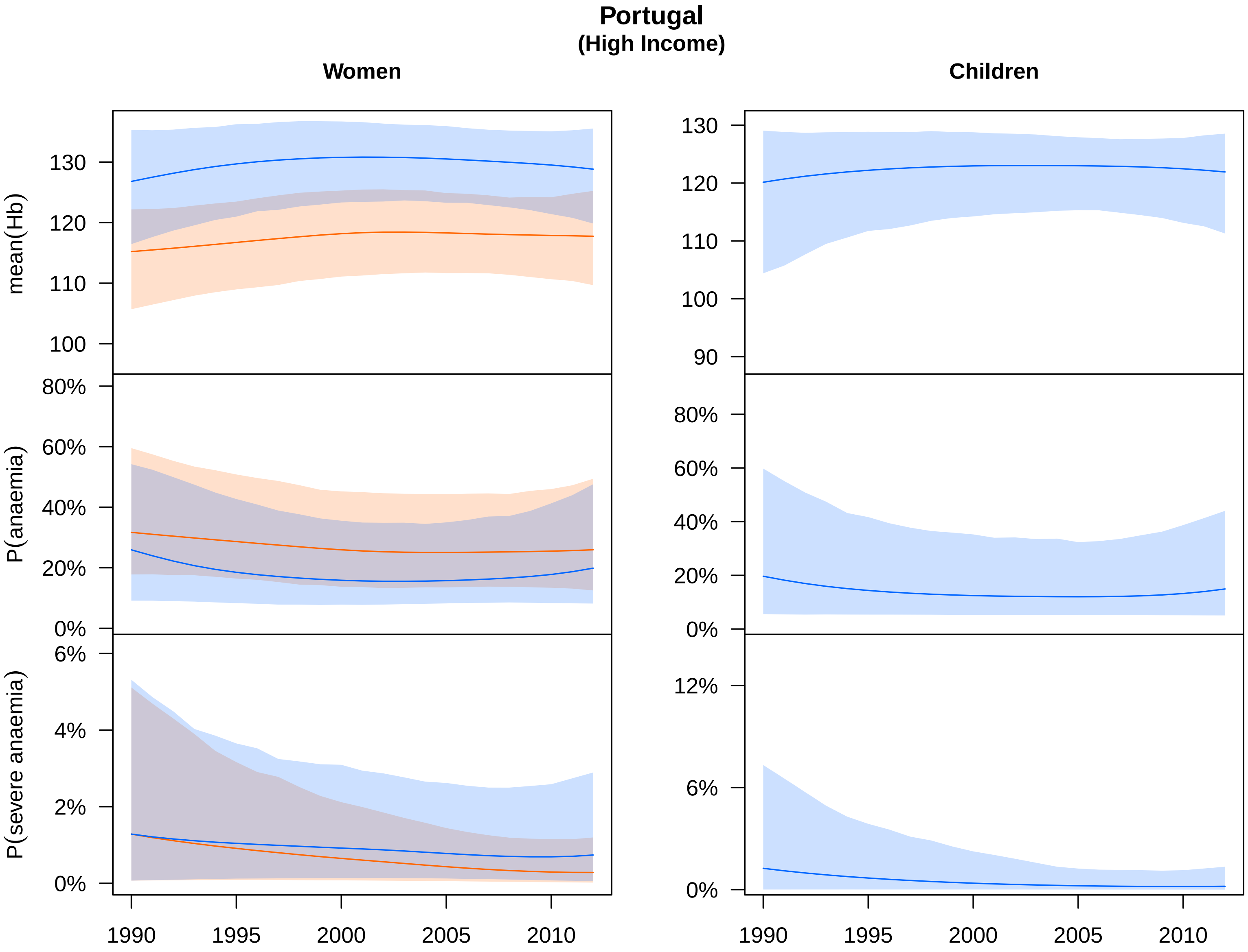
Portugal
(High Income)
Women
Children
mean(Hb)
P(anaemia)
P(severe anaemia)
130
120
110
100
90
80%
60%
40%
20%
0%
6%
4%
2%
12%
1990
1995
2000
2005
2010

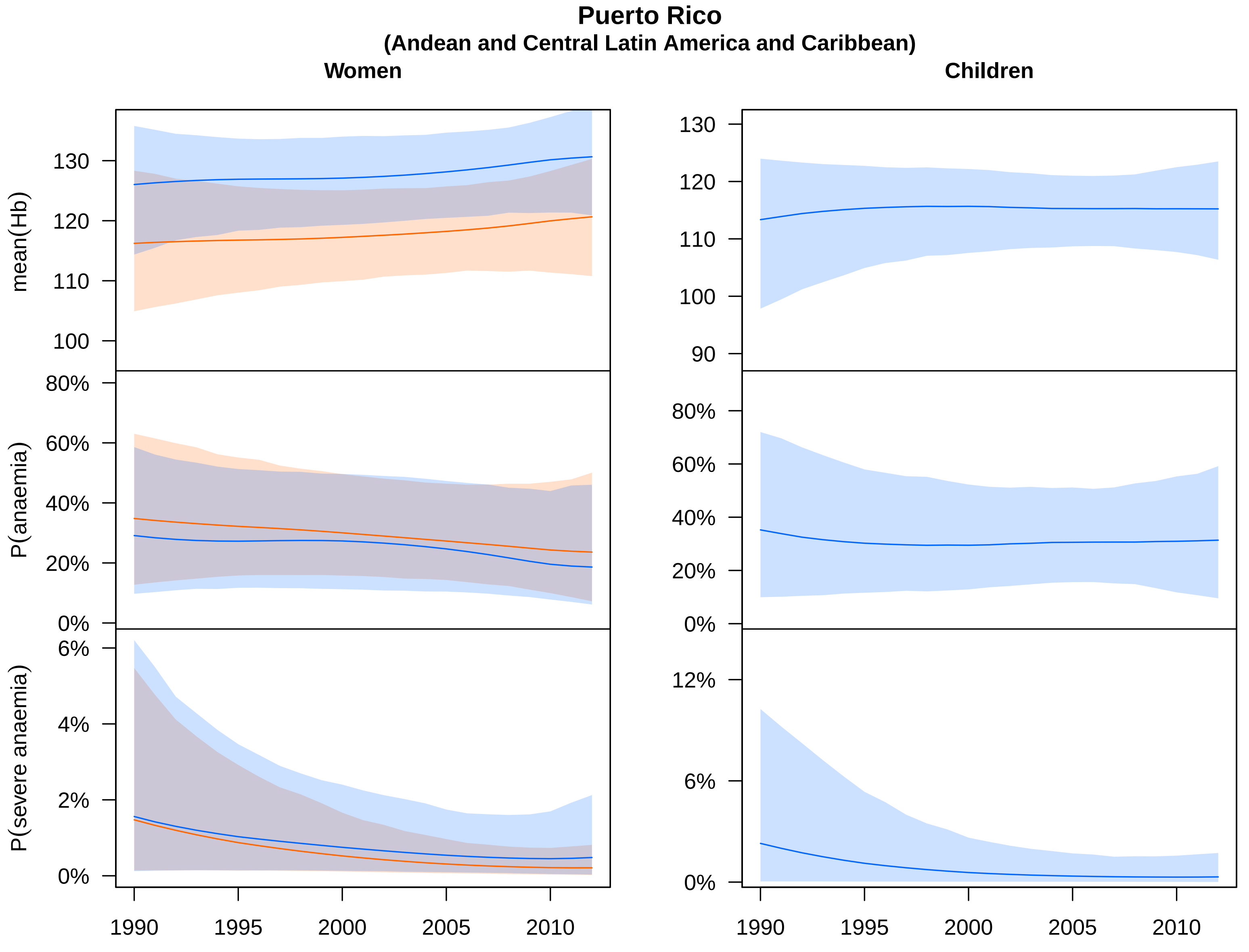
Puerto Rico
(Andean and Central Latin America and Caribbean)
Women
Children
mean(Hb)
P(anaemia)
P(severe anaemia)
130
120
110
100
90
80%
60%
40%
20%
0%
6%
4%
2%
12%
1990
1995
2000
2005
2010

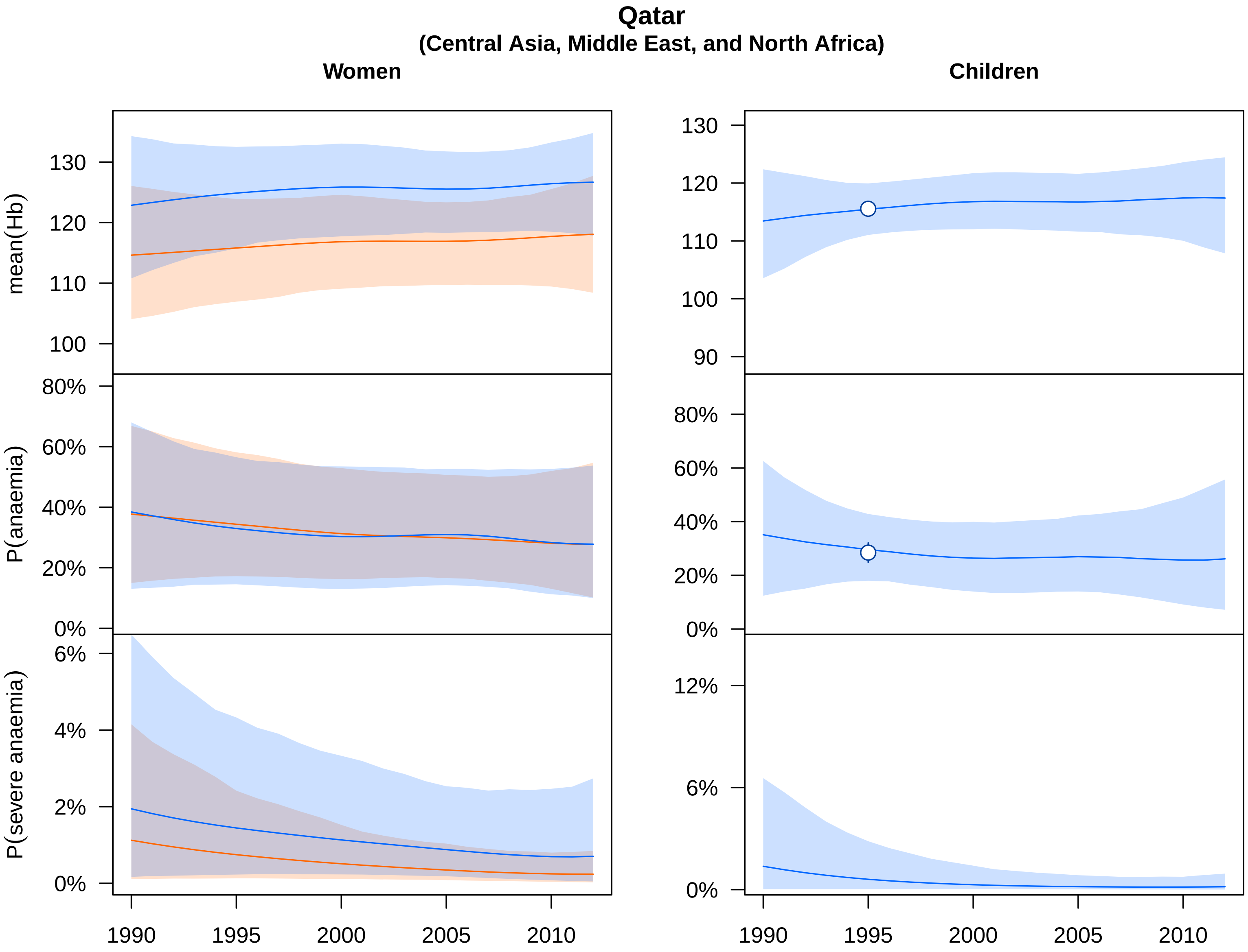
Qatar
(Central Asia, Middle East, and North Africa)
Women
Children
mean(Hb)
P(anaemia)
P(severe anaemia)
130
120
110
100
90
80%
60%
40%
20%
0%
6%
4%
2%
12%
1990
1995
2000
2005
2010

## Republic of Korea
### (High Income)

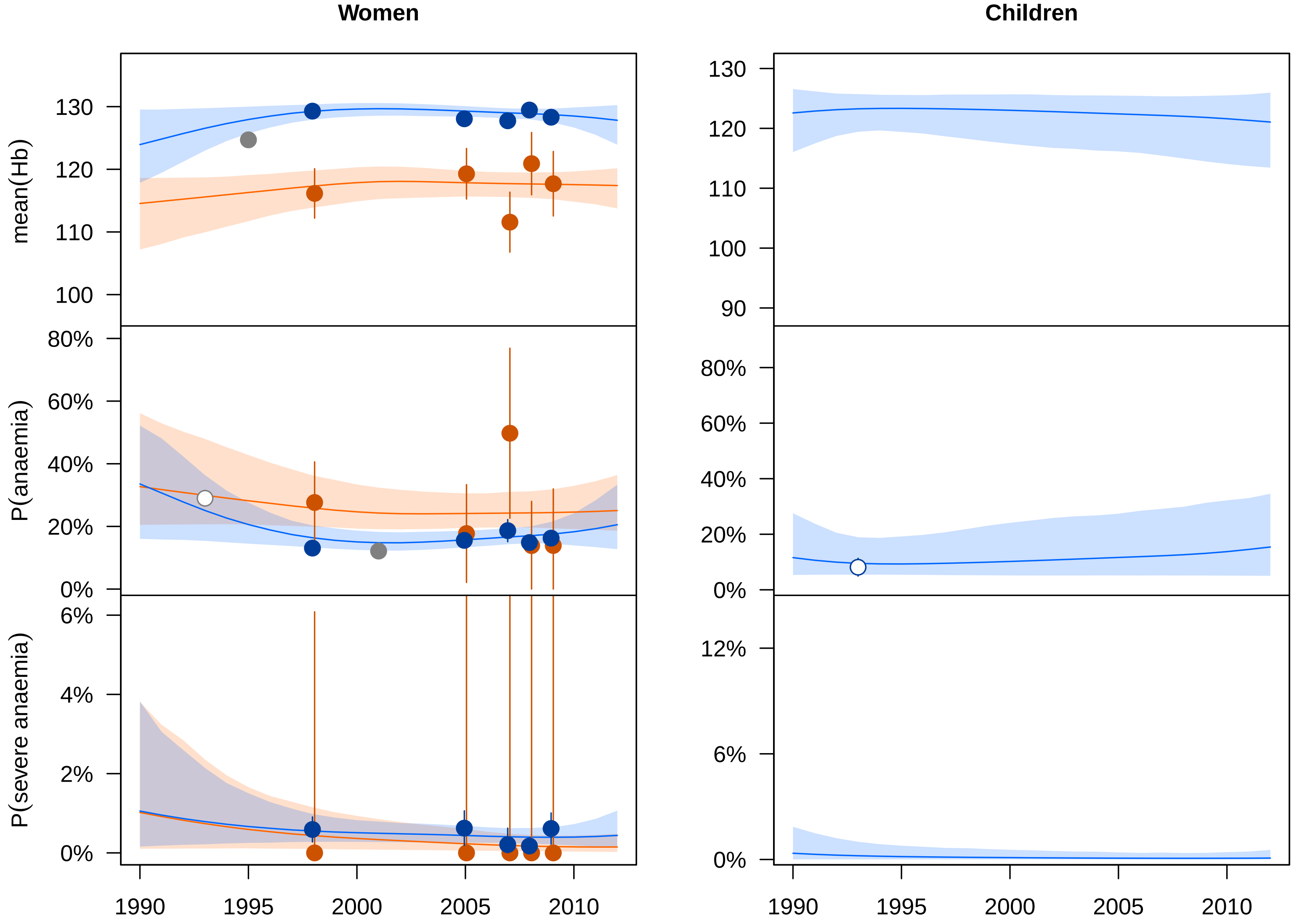

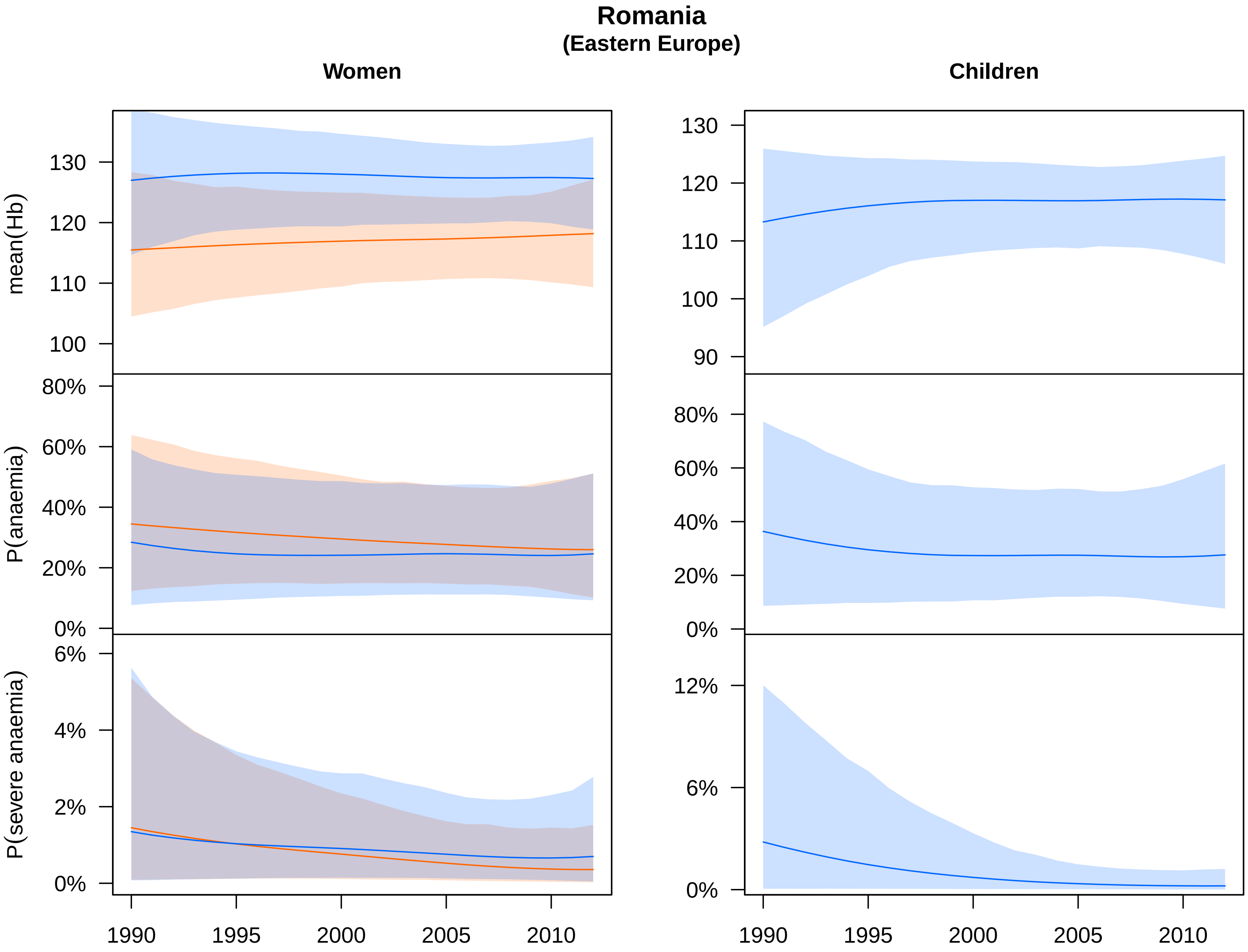
Romania
(Eastern Europe)
Women
Children
mean(Hb)
P(anaemia)
P(severe anaemia)
130
120
110
100
90
80%
60%
40%
20%
0%
6%
4%
2%
12%
1990
1995
2000
2005
2010

## Russian Federation
### (Eastern Europe)

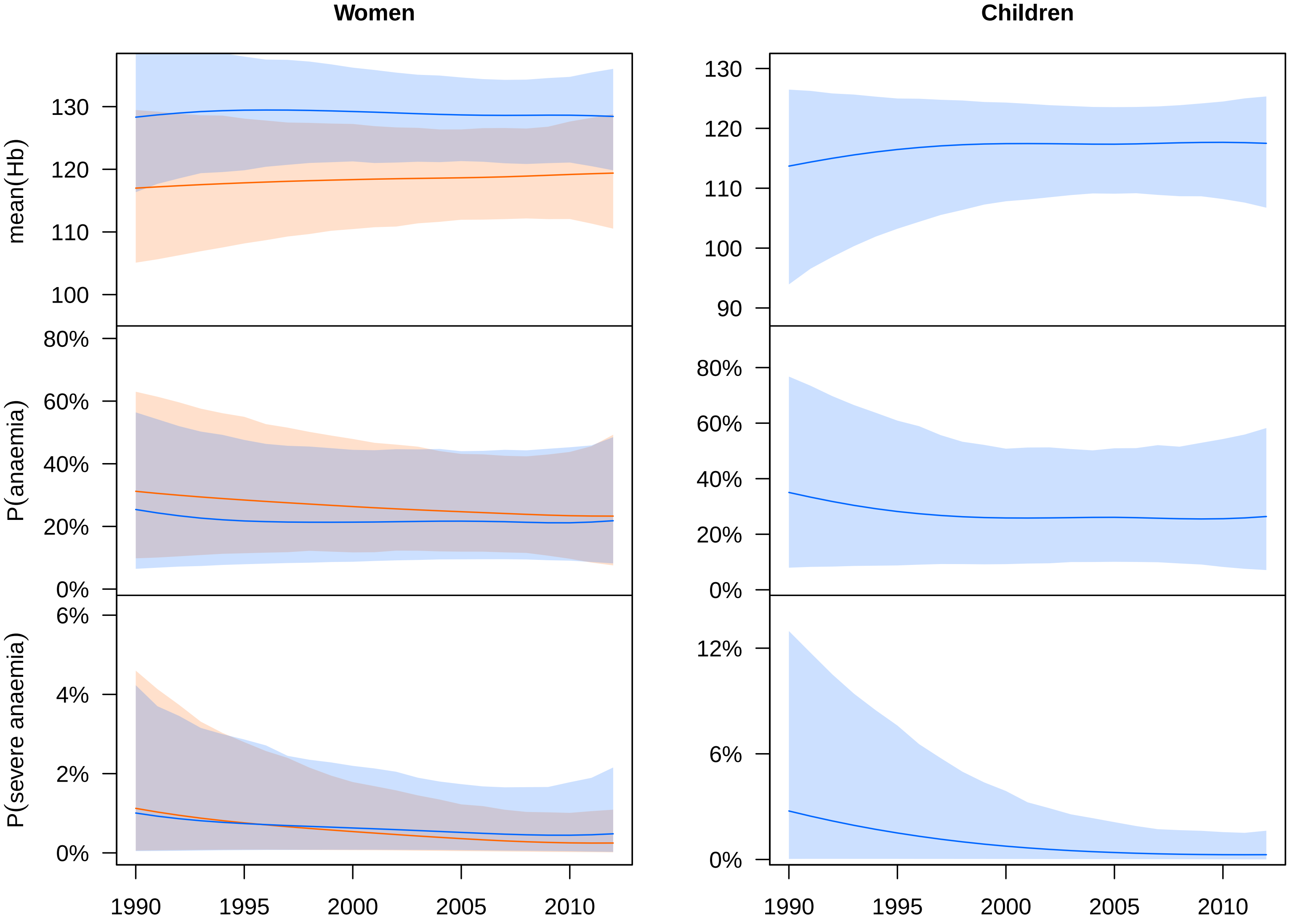

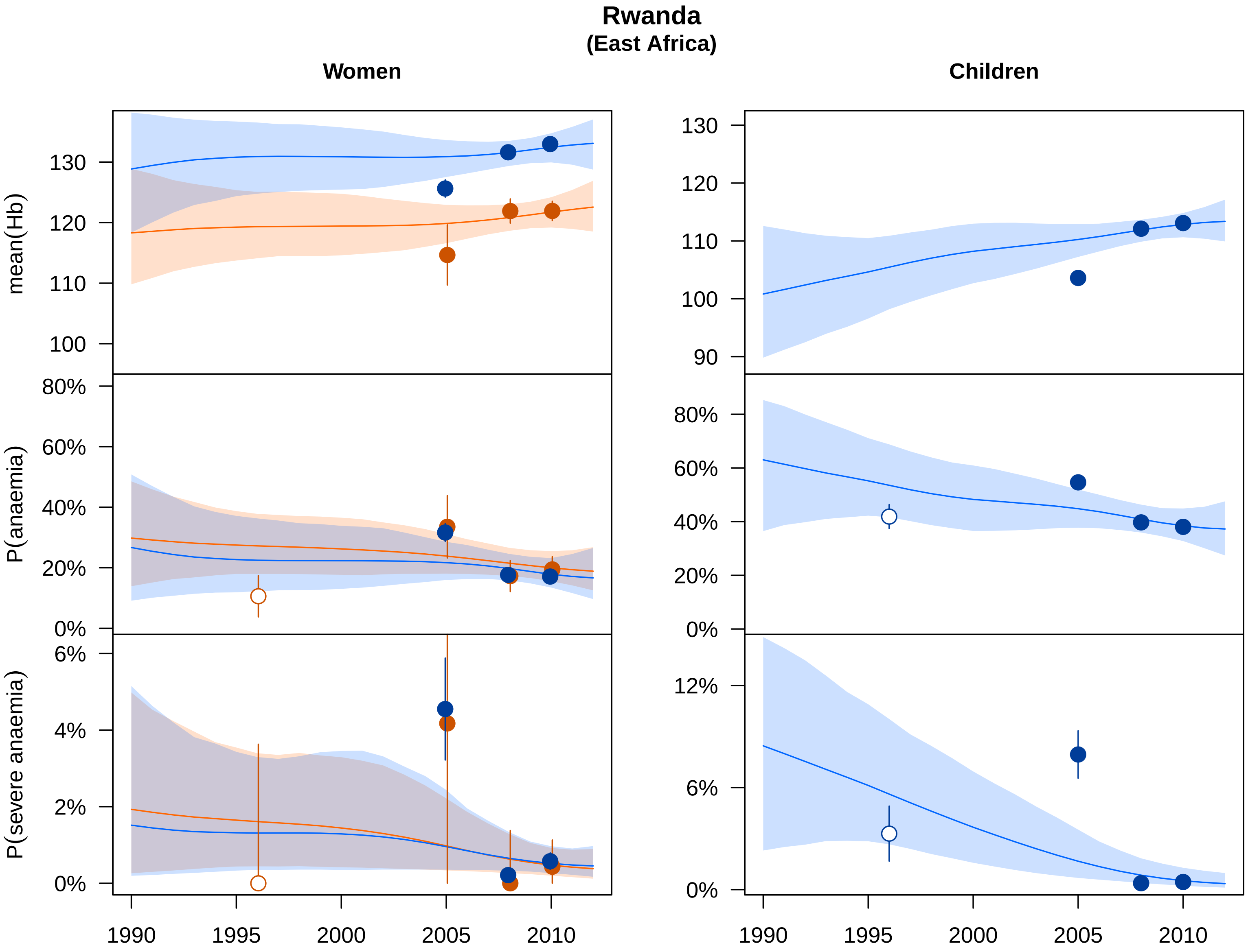

Rwanda
(East Africa)
Women
Children
mean(Hb)
P(anaemia)
P(severe anaemia)
130
120
110
100
90
80%
60%
40%
20%
0%
6%
4%
2%
12%
1990
1995
2000
2005
2010

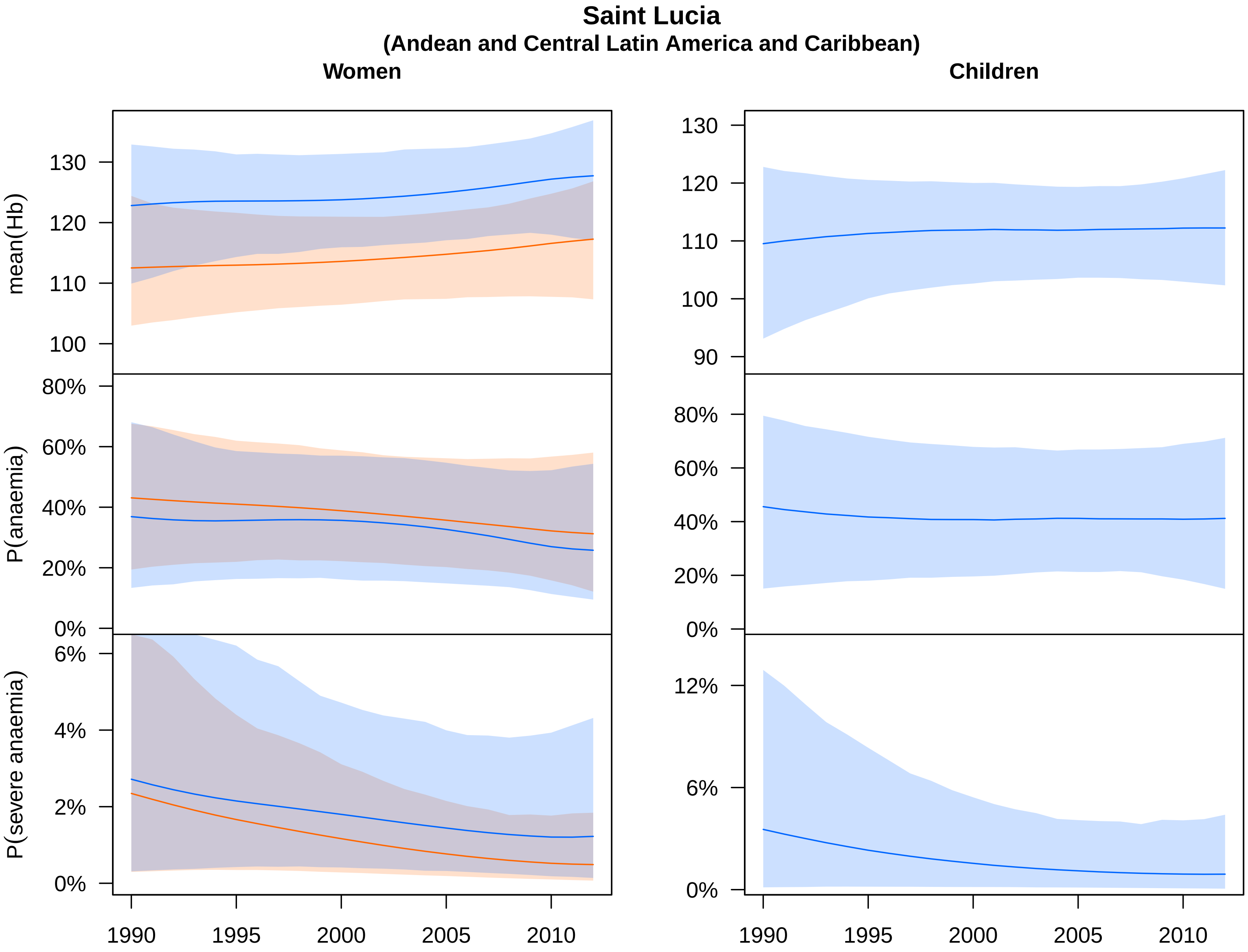
Saint Lucia
(Andean and Central Latin America and Caribbean)
Women
Children
mean(Hb)
P(anaemia)
P(severe anaemia)
130
120
110
100
90
80%
60%
40%
20%
0%
6%
4%
2%
12%
1990
1995
2000
2005
2010

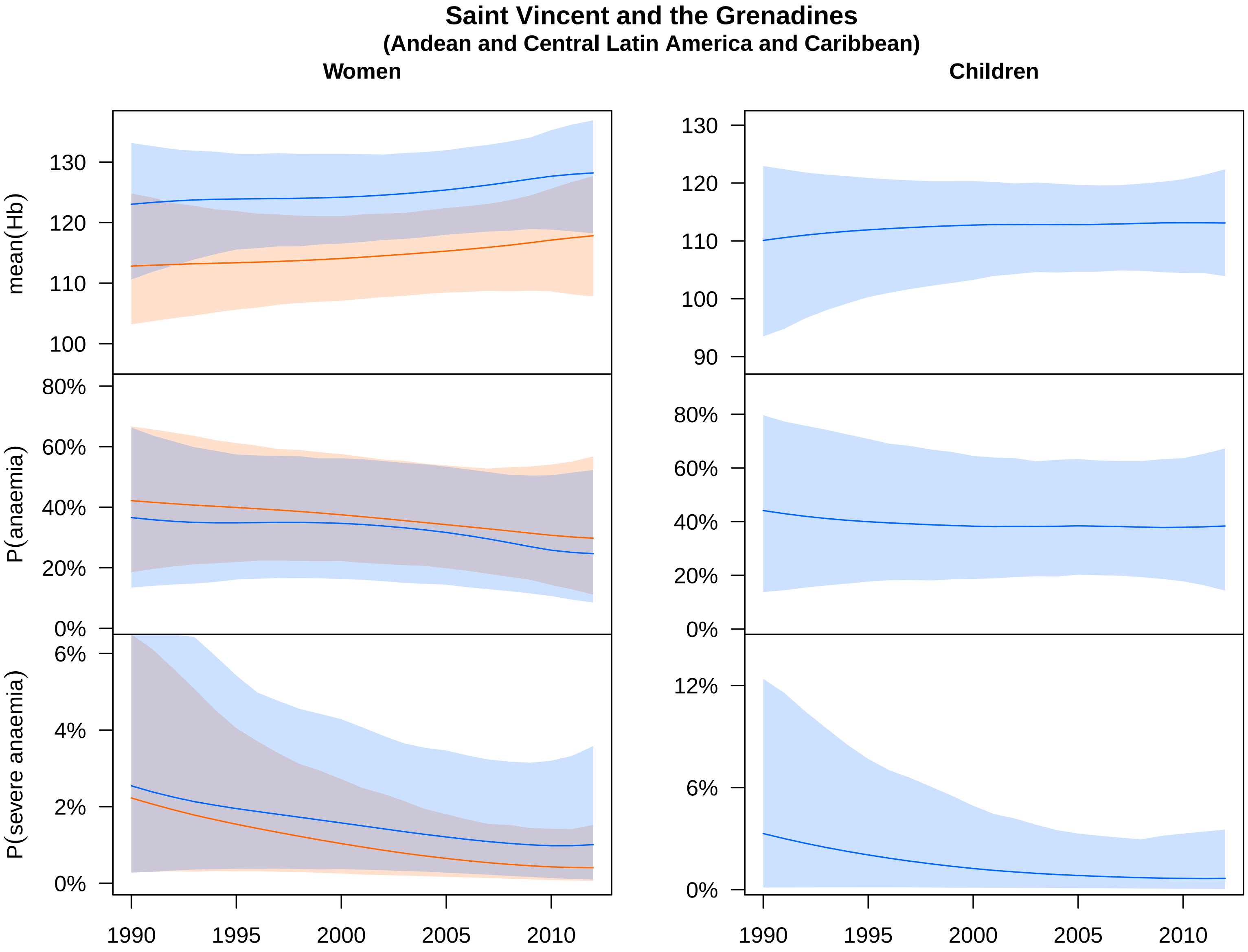
Saint Vincent and the Grenadines
(Andean and Central Latin America and Caribbean)
Women
Children
mean(Hb)
P(anaemia)
P(severe anaemia)
130
120
110
100
90
80%
60%
40%
20%
0%
6%
4%
2%
12%
1990
1995
2000
2005
2010

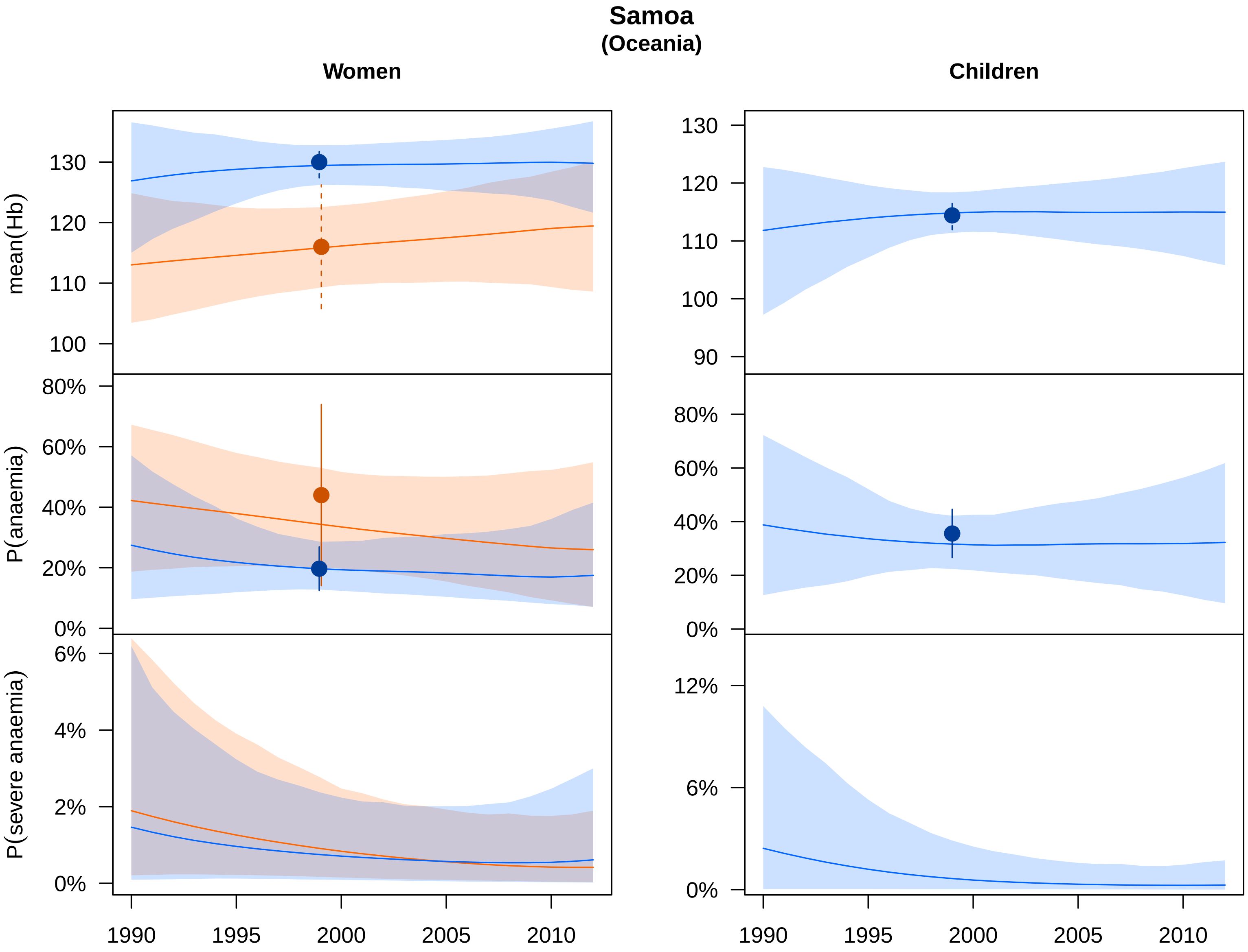
Samoa
(Oceania)
Women
Children
mean(Hb)
P(anaemia)
P(severe anaemia)
130
120
110
100
90
80%
60%
40%
20%
0%
6%
4%
2%
12%
1990
1995
2000
2005
2010

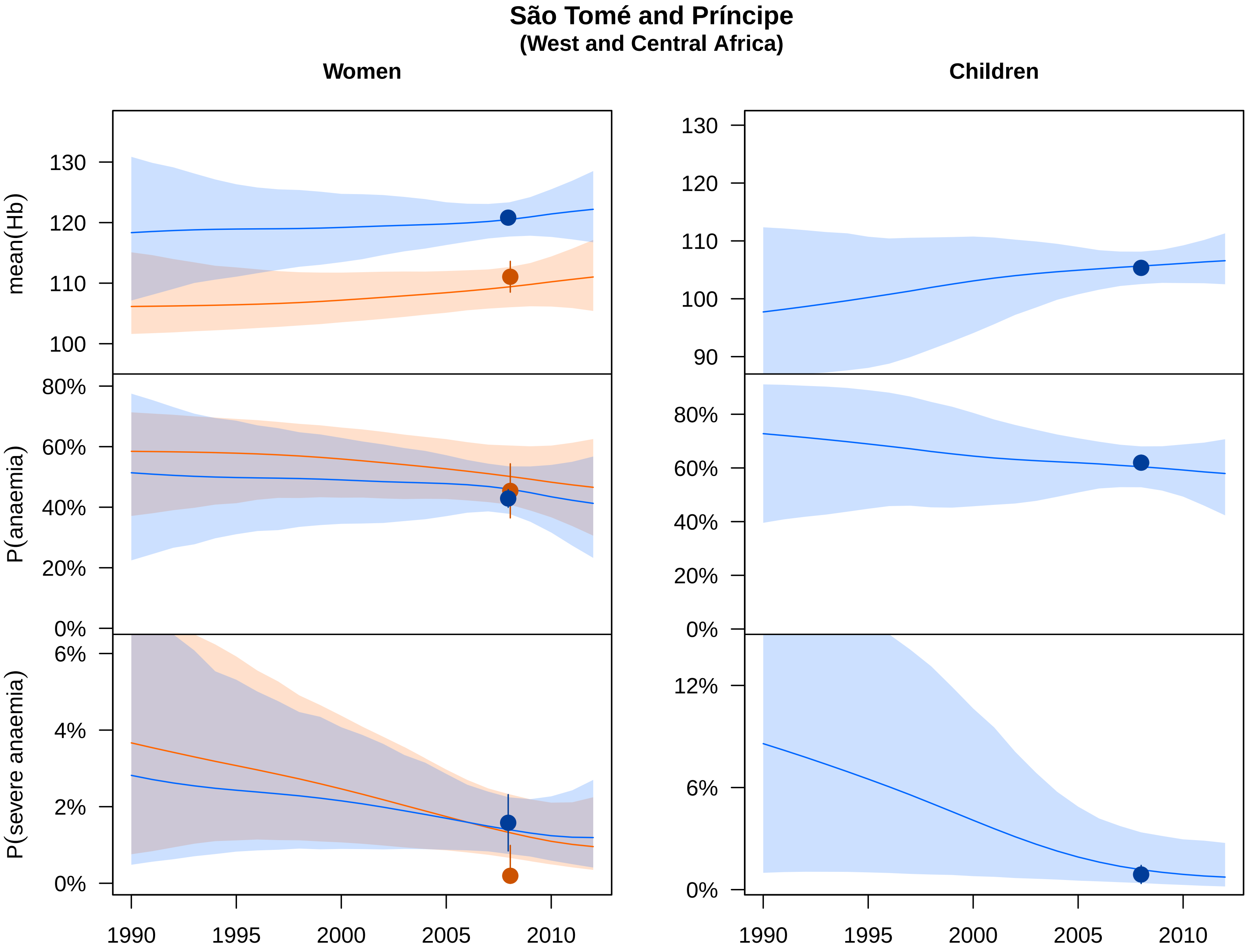
São Tomé and Príncipe
(West and Central Africa)
Women
Children
mean(Hb)
P(anaemia)
P(severe anaemia)
130
120
110
100
90
80%
60%
40%
20%
0%
6%
4%
2%
12%
1990
1995
2000
2005
2010

**Saudi Arabia**
**(Central Asia, Middle East, and North Africa)**

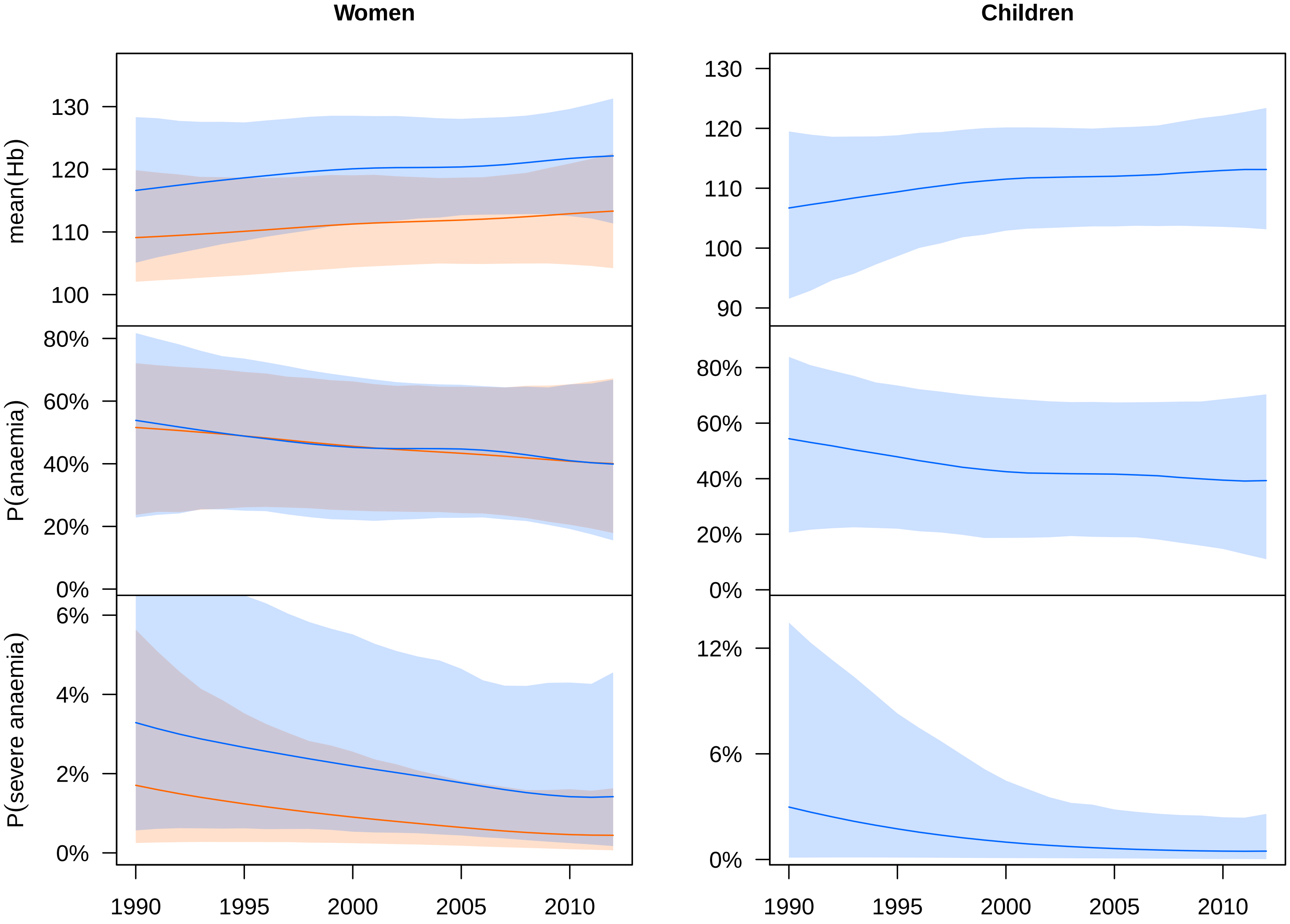

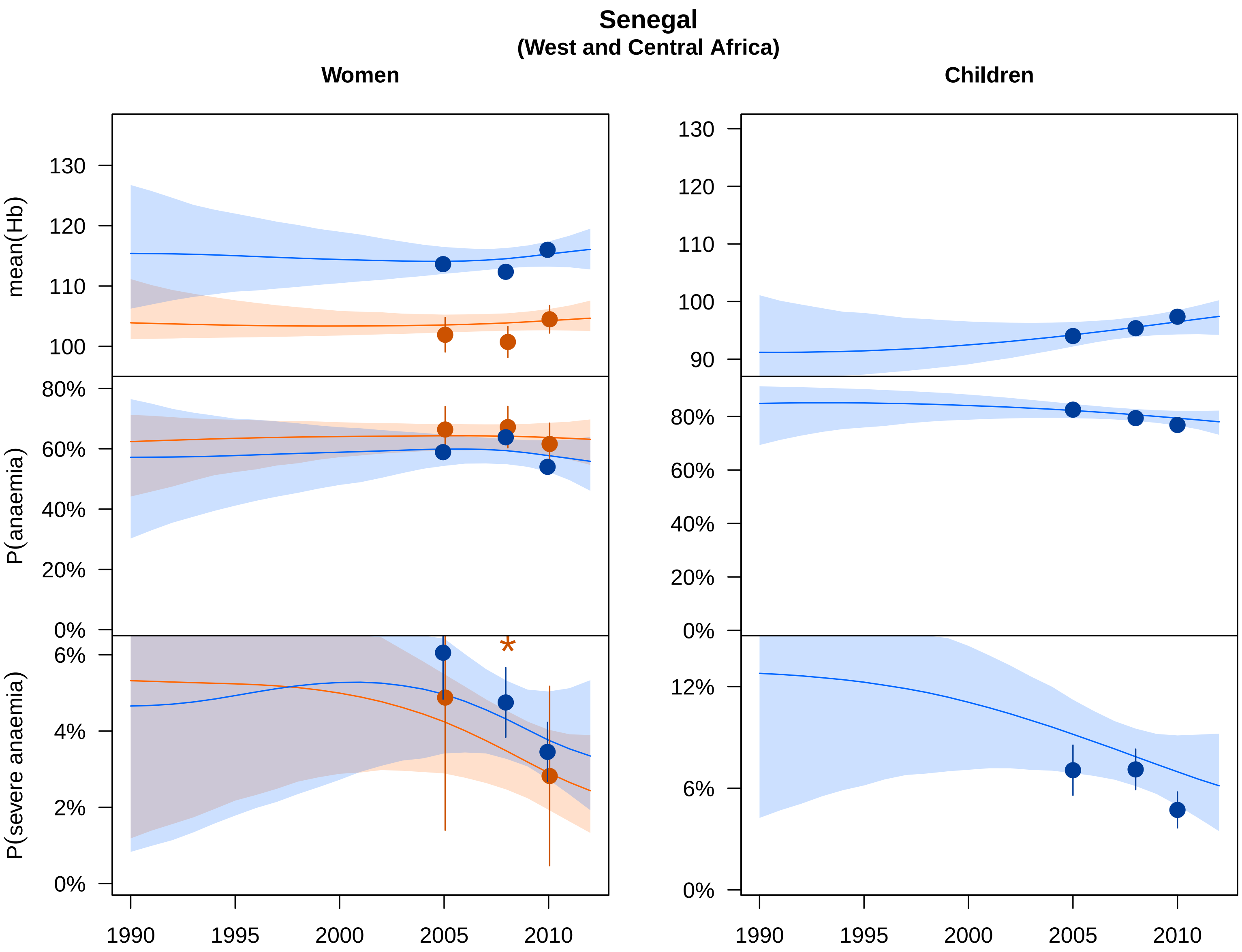

Senegal
(West and Central Africa)
Women
Children
mean(Hb)
P(anaemia)
P(severe anaemia)
130
120
110
100
90
80%
60%
40%
20%
0%
6%
4%
2%
12%
1990
1995
2000
2005
2010

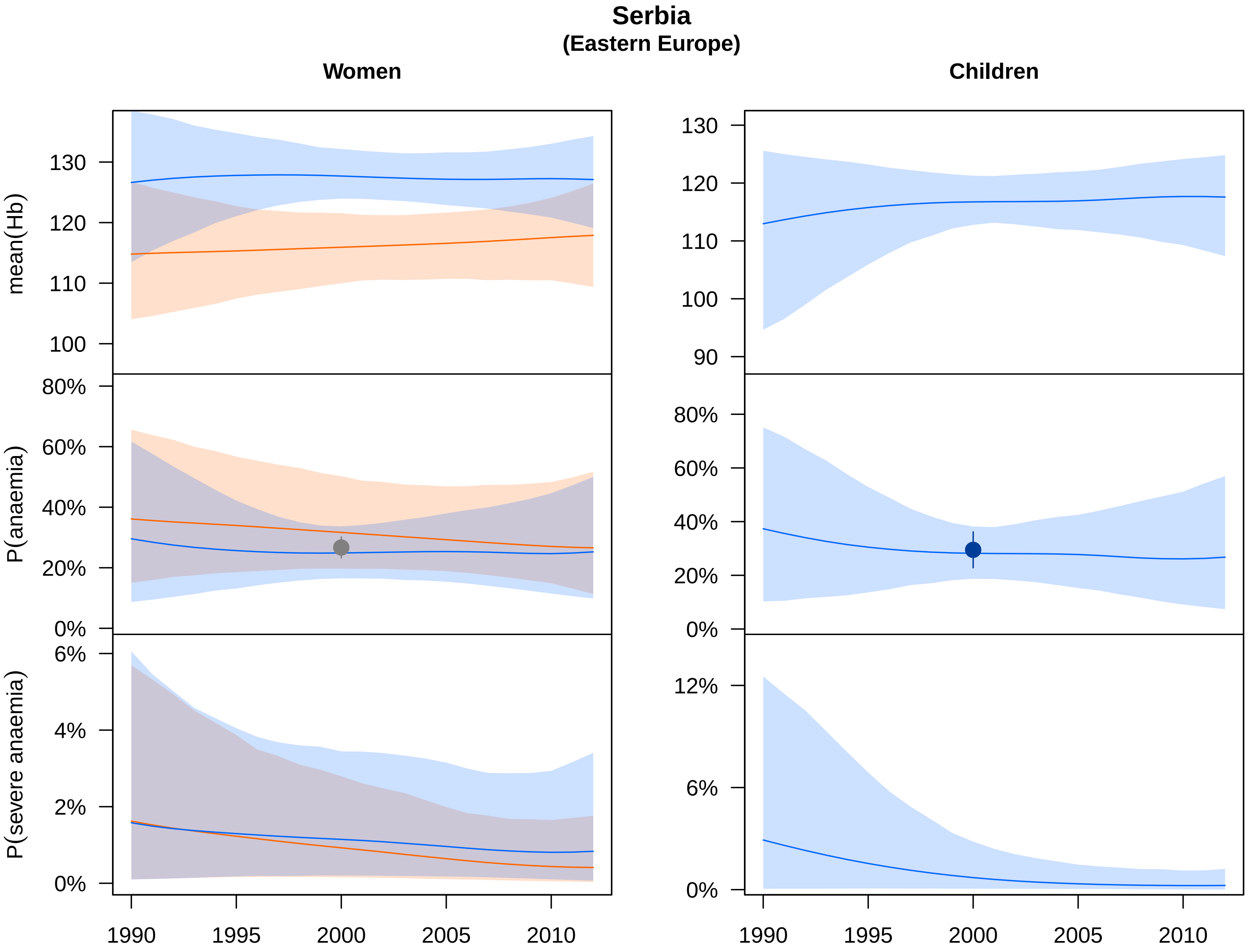
Serbia
(Eastern Europe)
Women
Children
mean(Hb)
P(anaemia)
P(severe anaemia)
130
120
110
100
90
80%
60%
40%
20%
0%
6%
4%
2%
12%
1990
1995
2000
2005
2010

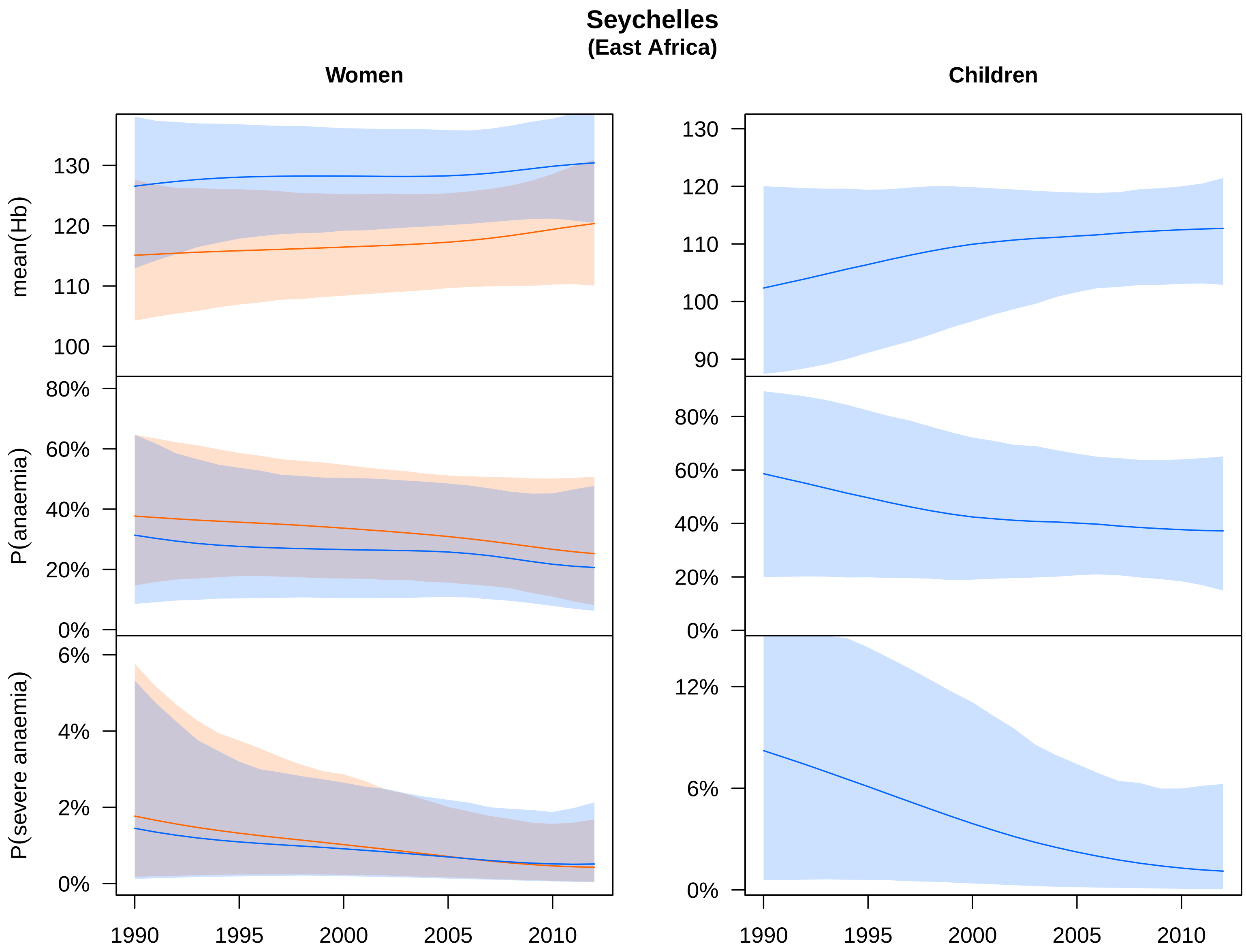

Seychelles
(East Africa)
Women
Children
mean(Hb)
P(anaemia)
P(severe anaemia)
130
120
110
100
90
80%
60%
40%
20%
0%
6%
4%
2%
12%
1990
1995
2000
2005
2010

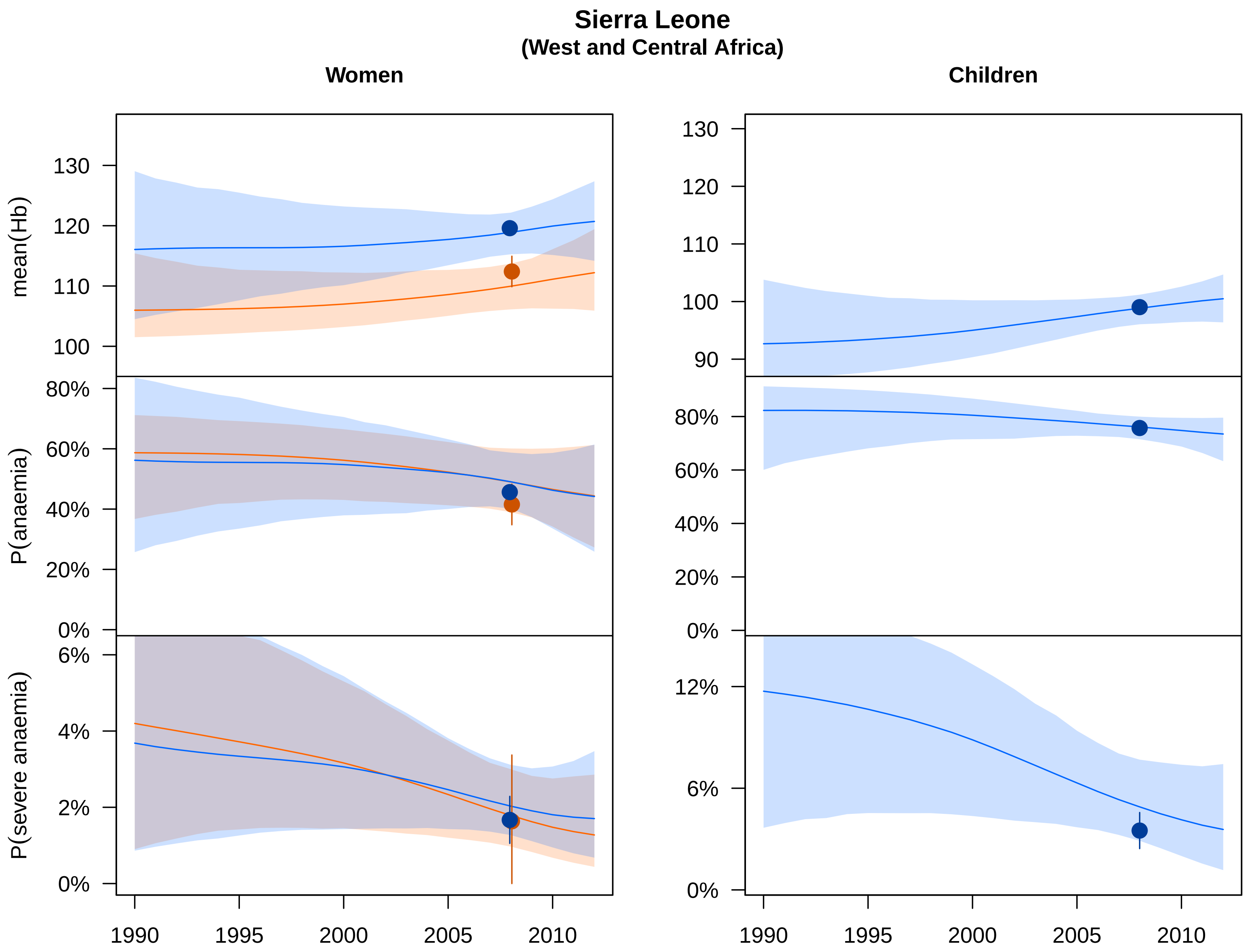
Sierra Leone
(West and Central Africa)
Women
Children
mean(Hb)
P(anaemia)
P(severe anaemia)
130
120
110
100
90
80%
60%
40%
20%
0%
6%
4%
2%
12%
1990
1995
2000
2005
2010

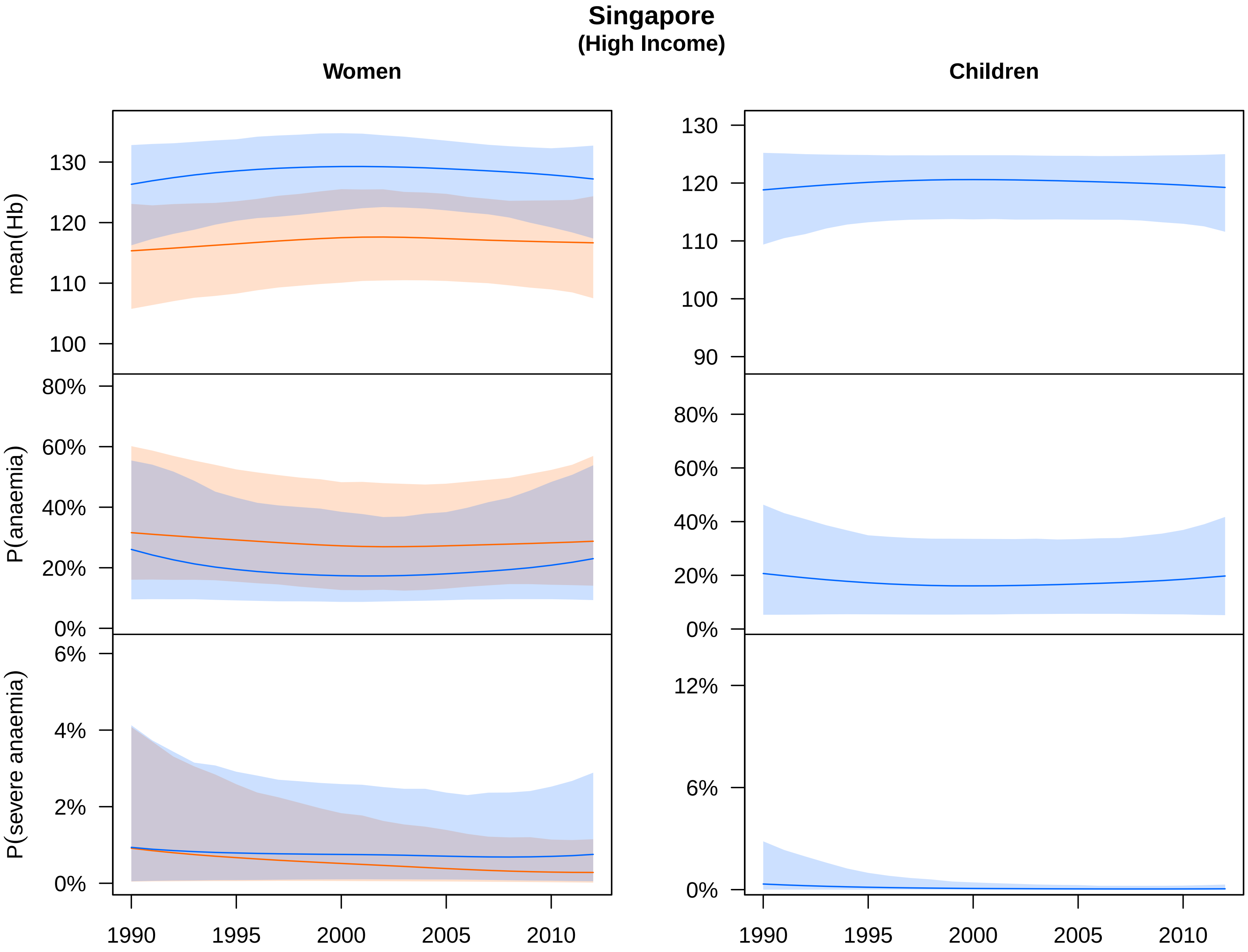
Singapore
(High Income)
Women
Children
mean(Hb)
P(anaemia)
P(severe anaemia)
130
120
110
100
90
80%
60%
40%
20%
0%
6%
4%
2%
12%
1990
1995
2000
2005
2010

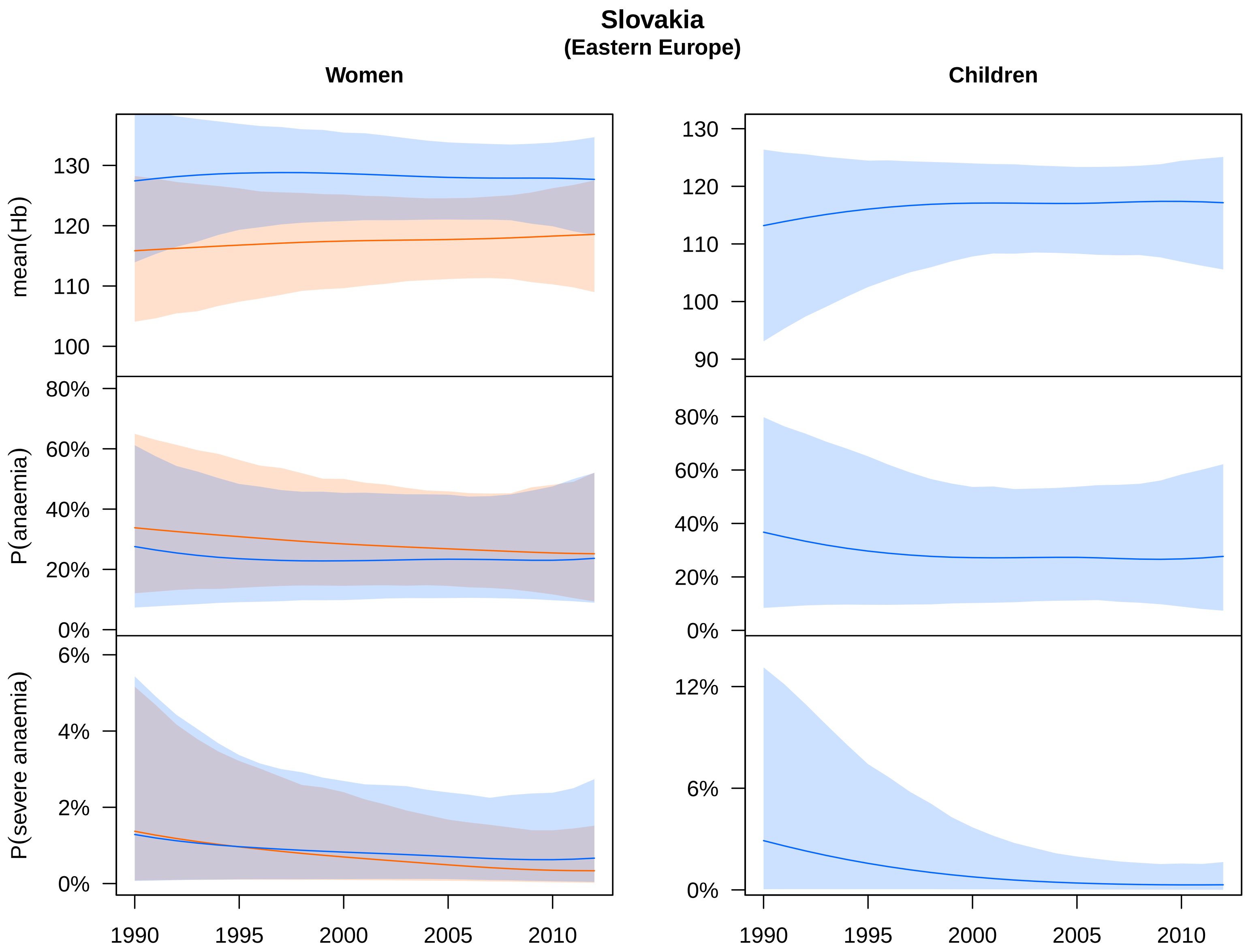

Slovakia
(Eastern Europe)
Women
Children
mean(Hb)
P(anaemia)
P(severe anaemia)
100
110
120
130
90
0%
20%
40%
60%
80%
2%
4%
6%
12%
1990
1995
2000
2005
2010

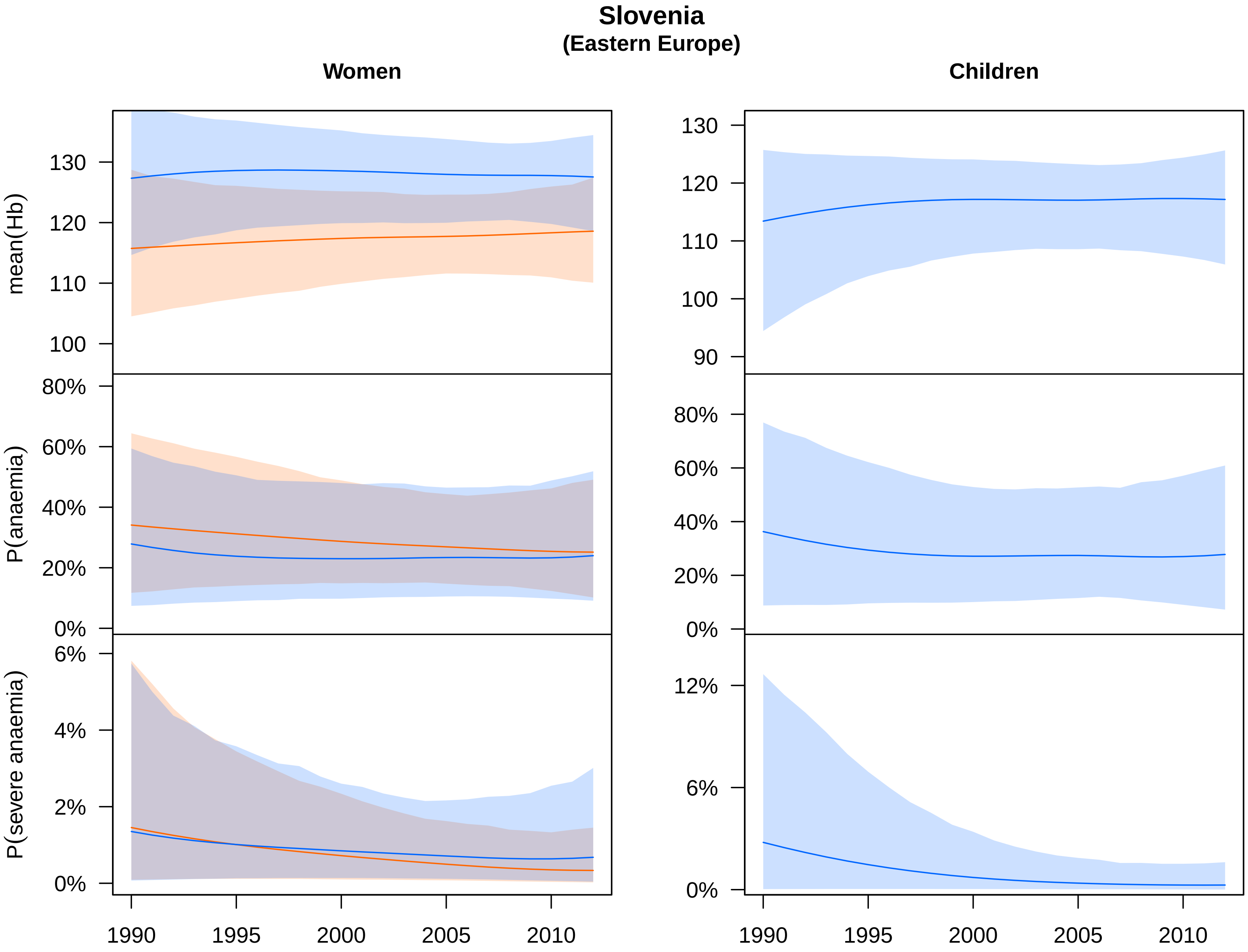
Slovenia
(Eastern Europe)
Women
Children
mean(Hb)
P(anaemia)
P(severe anaemia)
130
120
110
100
90
80%
60%
40%
20%
0%
6%
4%
2%
12%
1990
1995
2000
2005
2010

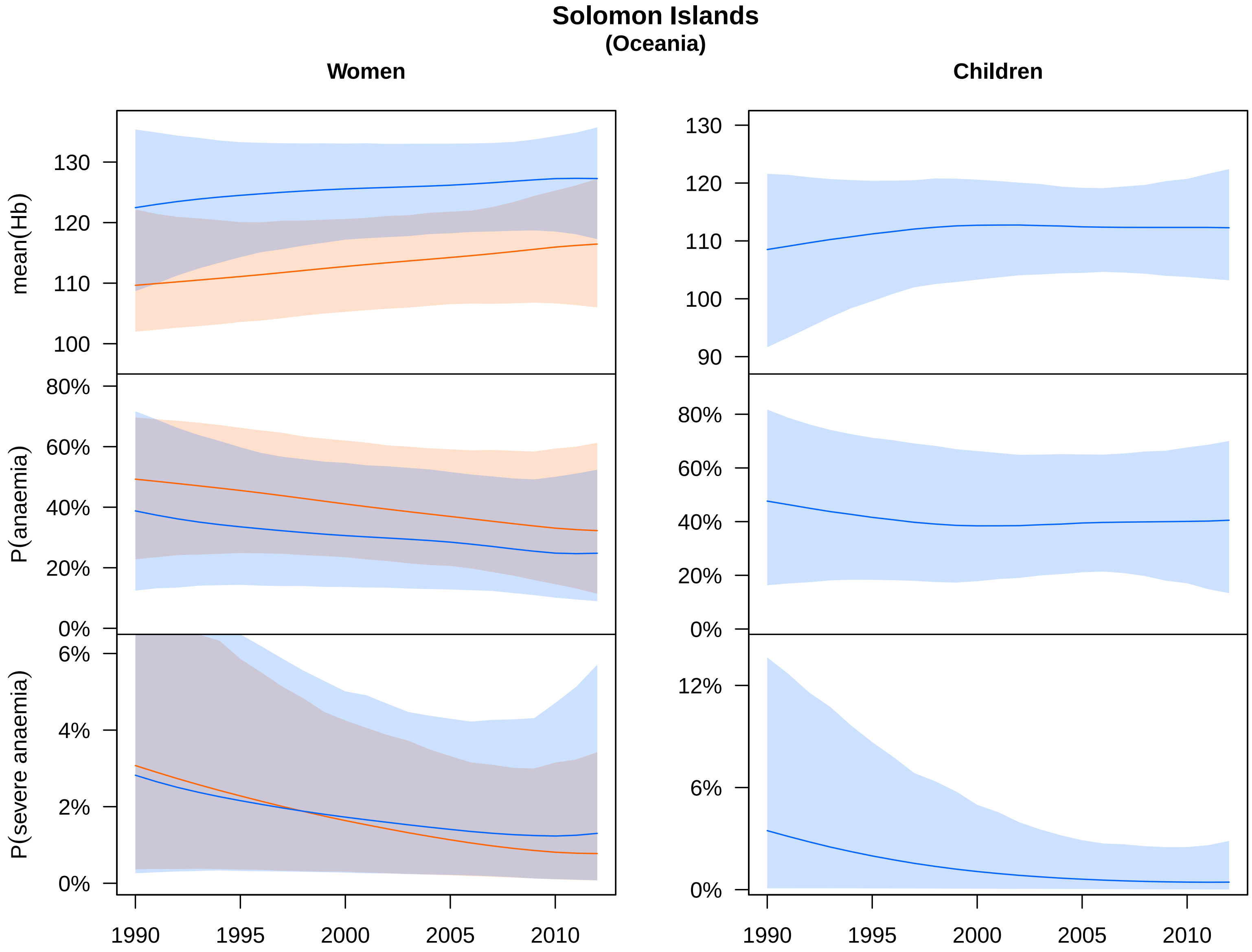

Solomon Islands
(Oceania)
Women
Children
mean(Hb)
P(anaemia)
P(severe anaemia)
130
120
110
100
90
80%
60%
40%
20%
0%
6%
4%
2%
12%
1990
1995
2000
2005
2010

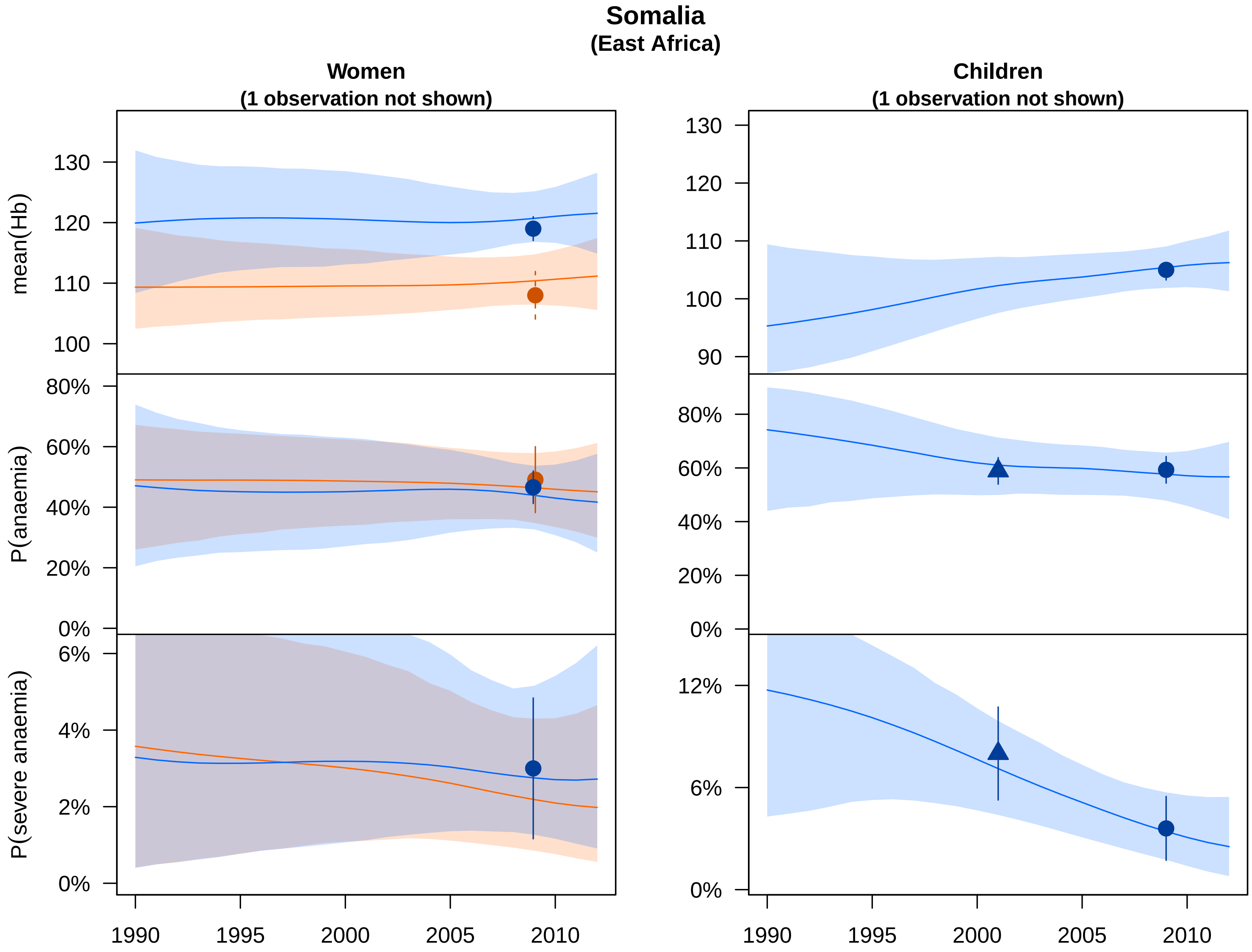
Somalia
(East Africa)
Women
(1 observation not shown)
Children
(1 observation not shown)
mean(Hb)
P(anaemia)
P(severe anaemia)
130
120
110
100
90
80%
60%
40%
20%
0%
6%
4%
2%
12%
1990
1995
2000
2005
2010

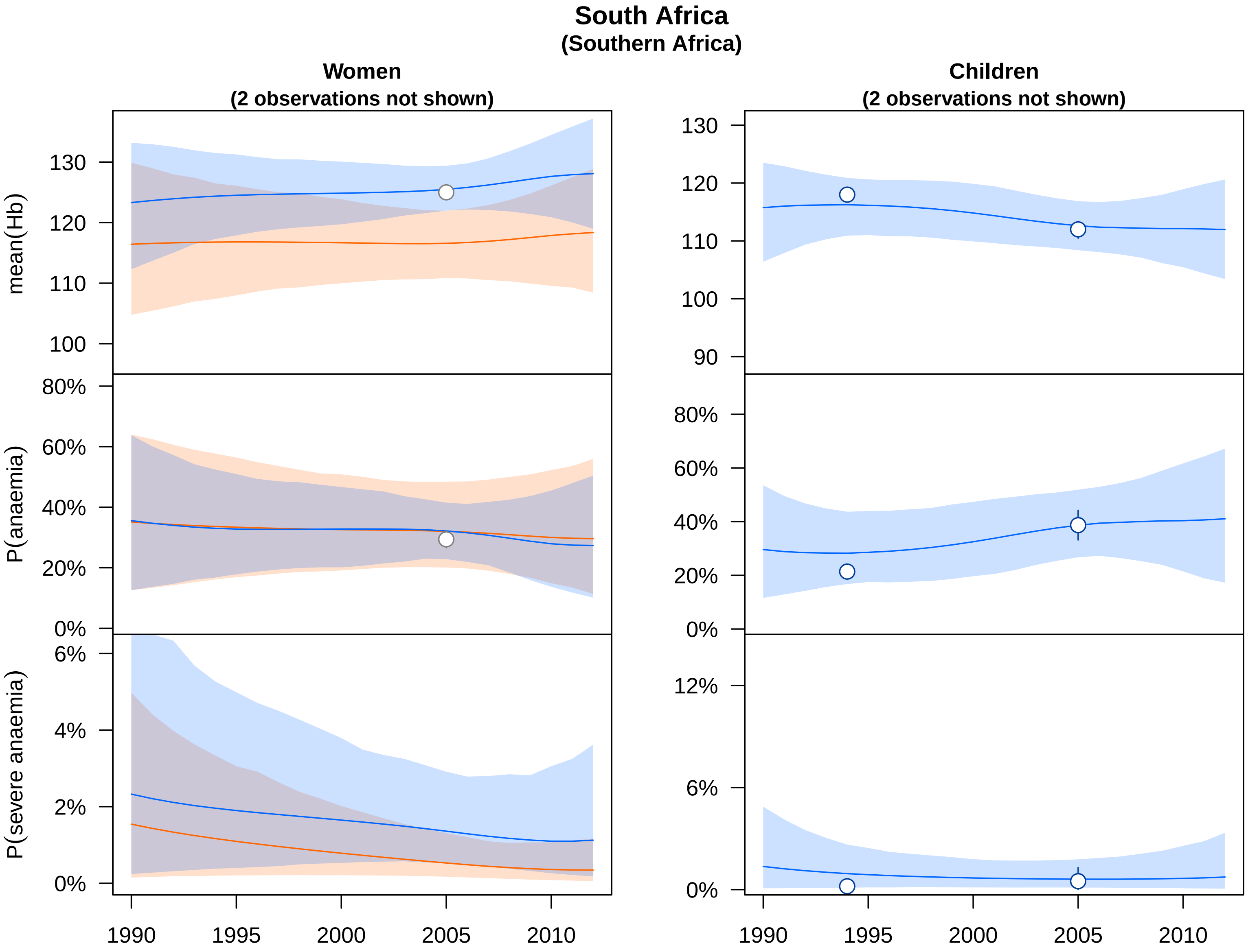
South Africa
(Southern Africa)
Women
(2 observations not shown)
Children
(2 observations not shown)
mean(Hb)
P(anaemia)
P(severe anaemia)
130
120
110
100
90
80%
60%
40%
20%
0%
6%
4%
2%
12%
1990
1995
2000
2005
2010

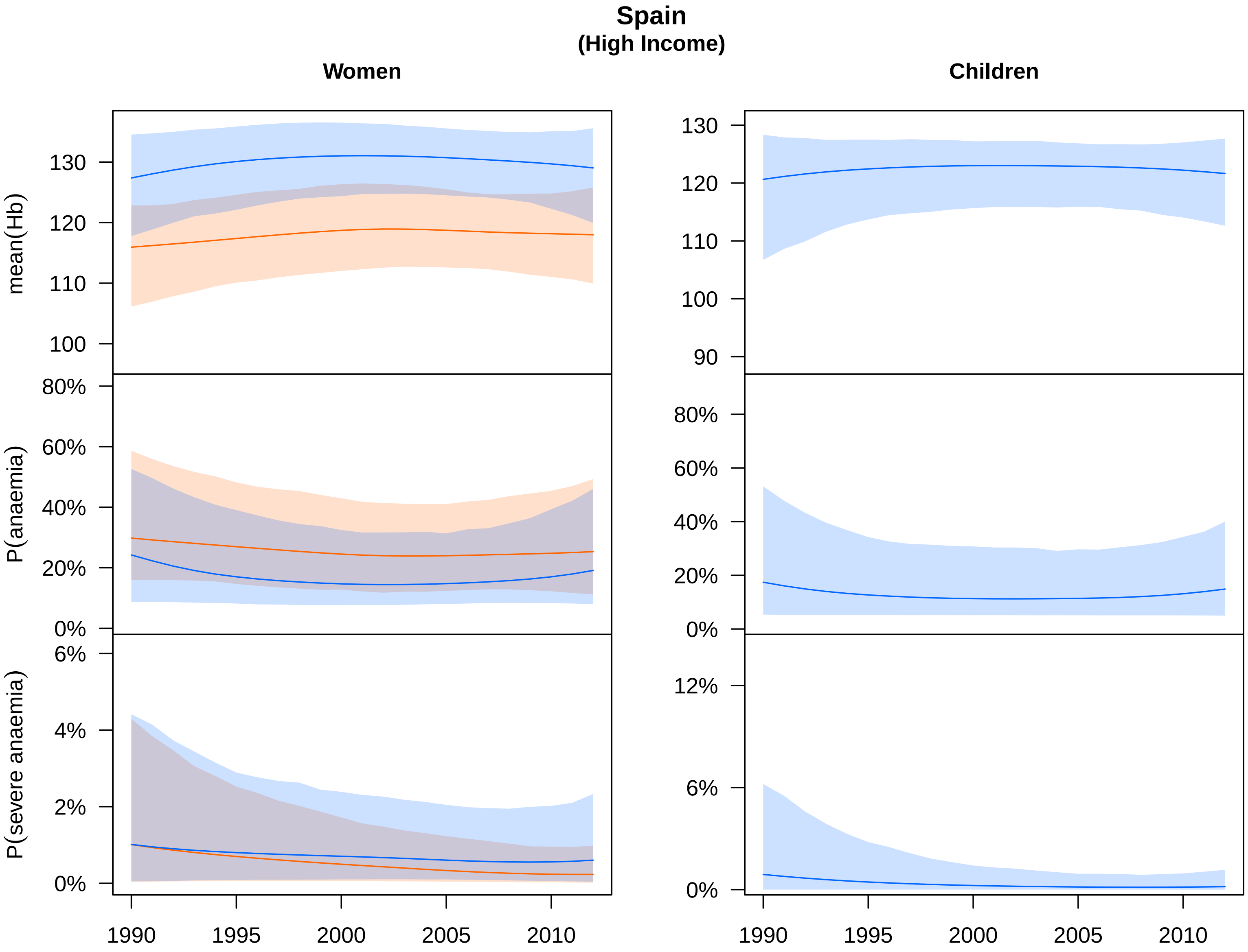
Spain
(High Income)
Women
Children
mean(Hb)
P(anaemia)
P(severe anaemia)
130
120
110
100
90
80%
60%
40%
20%
0%
6%
4%
2%
12%
1990
1995
2000
2005
2010

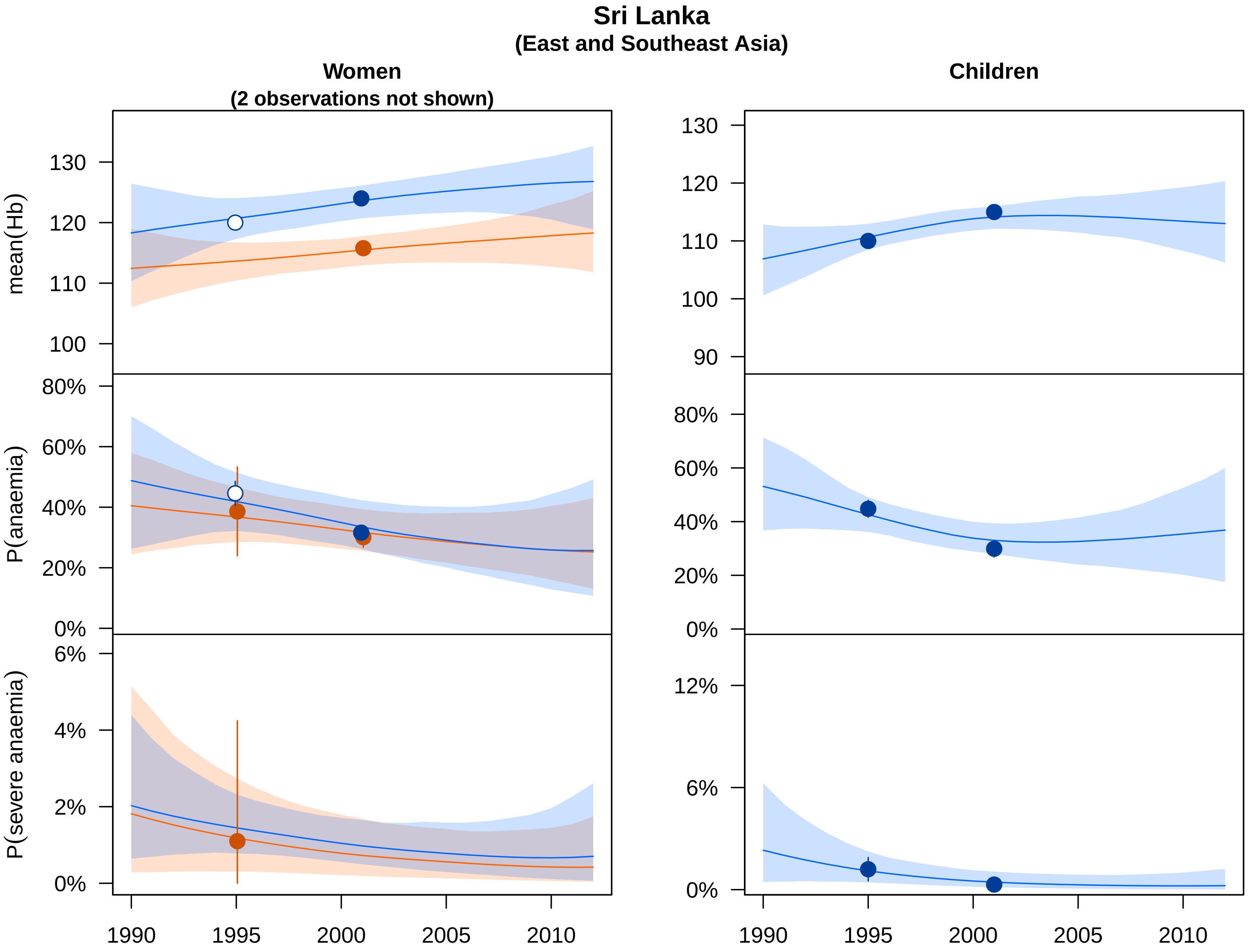
Sri Lanka
(East and Southeast Asia)
Women
(2 observations not shown)
Children
mean(Hb)
P(anaemia)
P(severe anaemia)
130
120
110
100
90
80%
60%
40%
20%
0%
6%
4%
2%
12%
1990
1995
2000
2005
2010

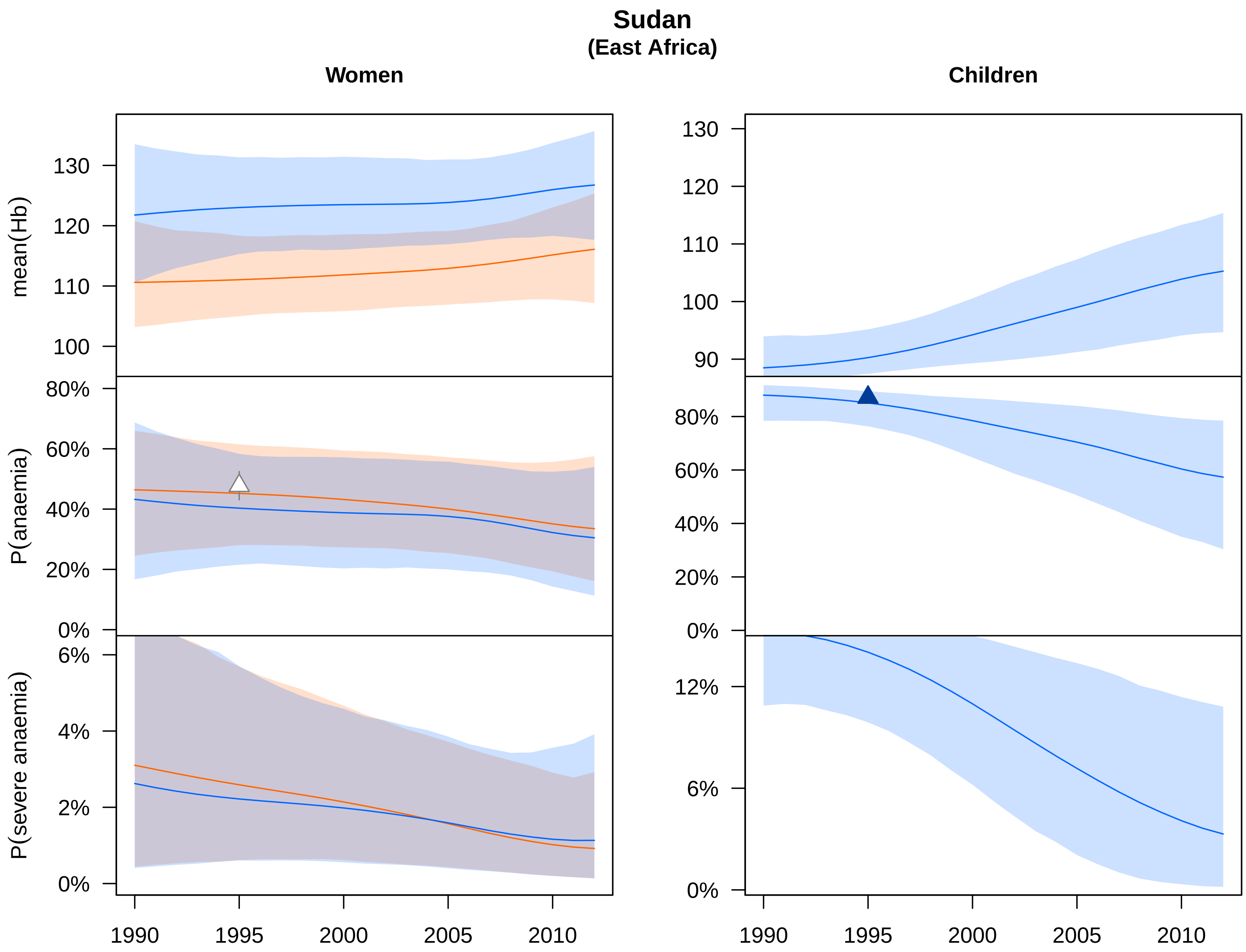

Sudan
(East Africa)
Women
Children
mean(Hb)
P(anaemia)
P(severe anaemia)
130
120
110
100
90
80%
60%
40%
20%
0%
6%
4%
2%
12%
1990
1995
2000
2005
2010

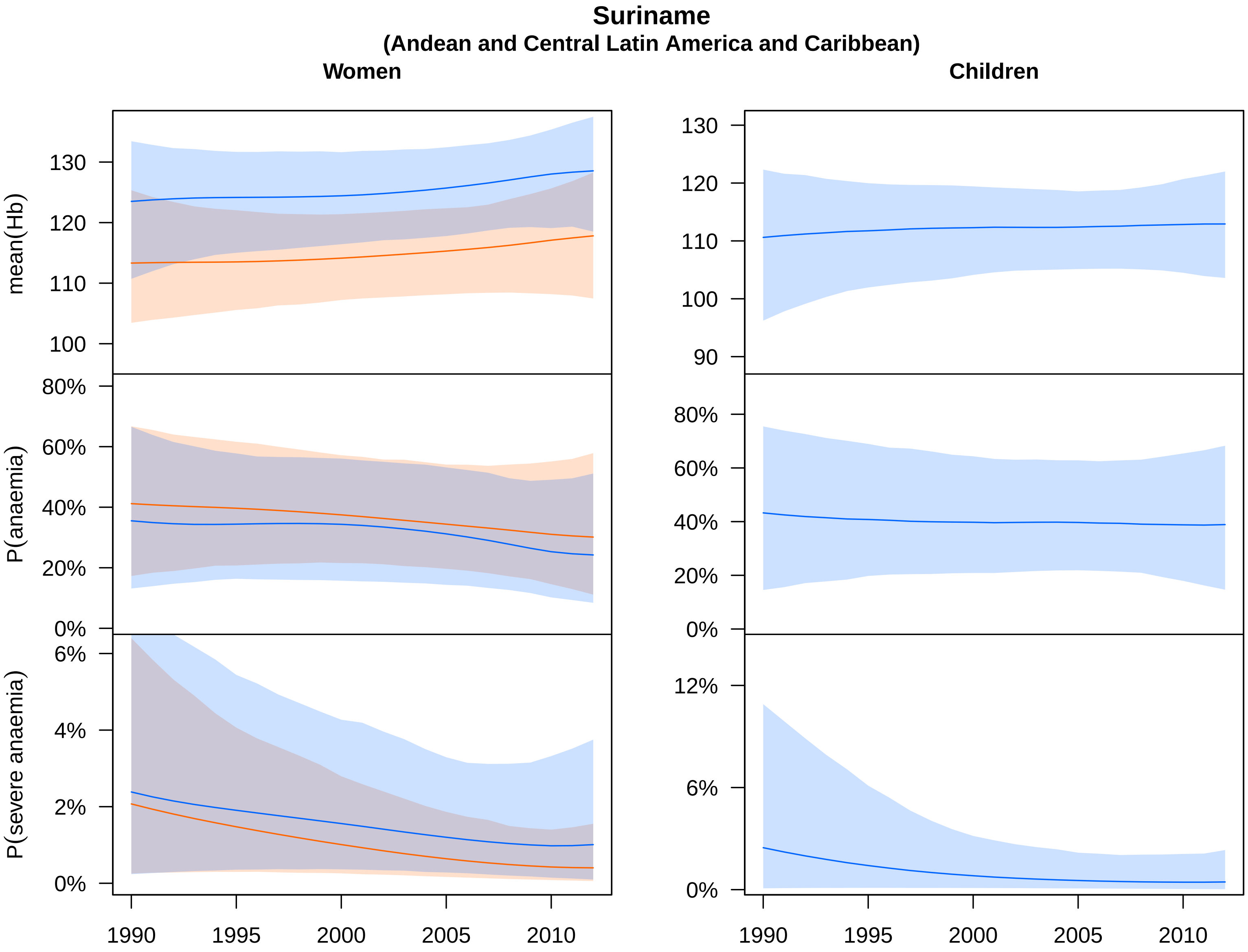

Suriname
(Andean and Central Latin America and Caribbean)
Women
Children
mean(Hb)
P(anaemia)
P(severe anaemia)
130
120
110
100
90
80%
60%
40%
20%
0%
6%
4%
2%
12%
1990
1995
2000
2005
2010

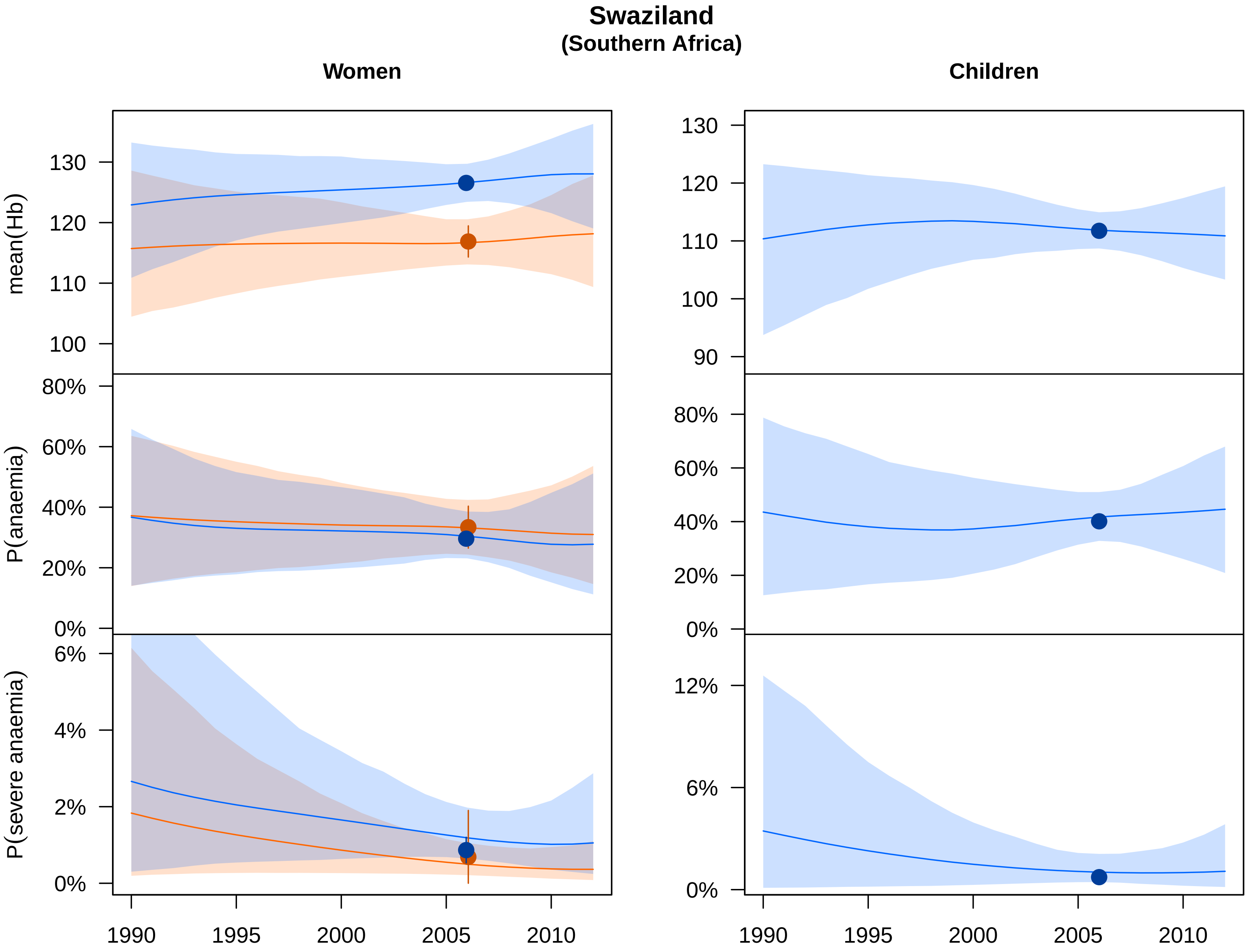

Swaziland
(Southern Africa)
Women
Children
mean(Hb)
P(anaemia)
P(severe anaemia)
130
120
110
100
90
80%
60%
40%
20%
0%
6%
4%
2%
12%
1990
1995
2000
2005
2010

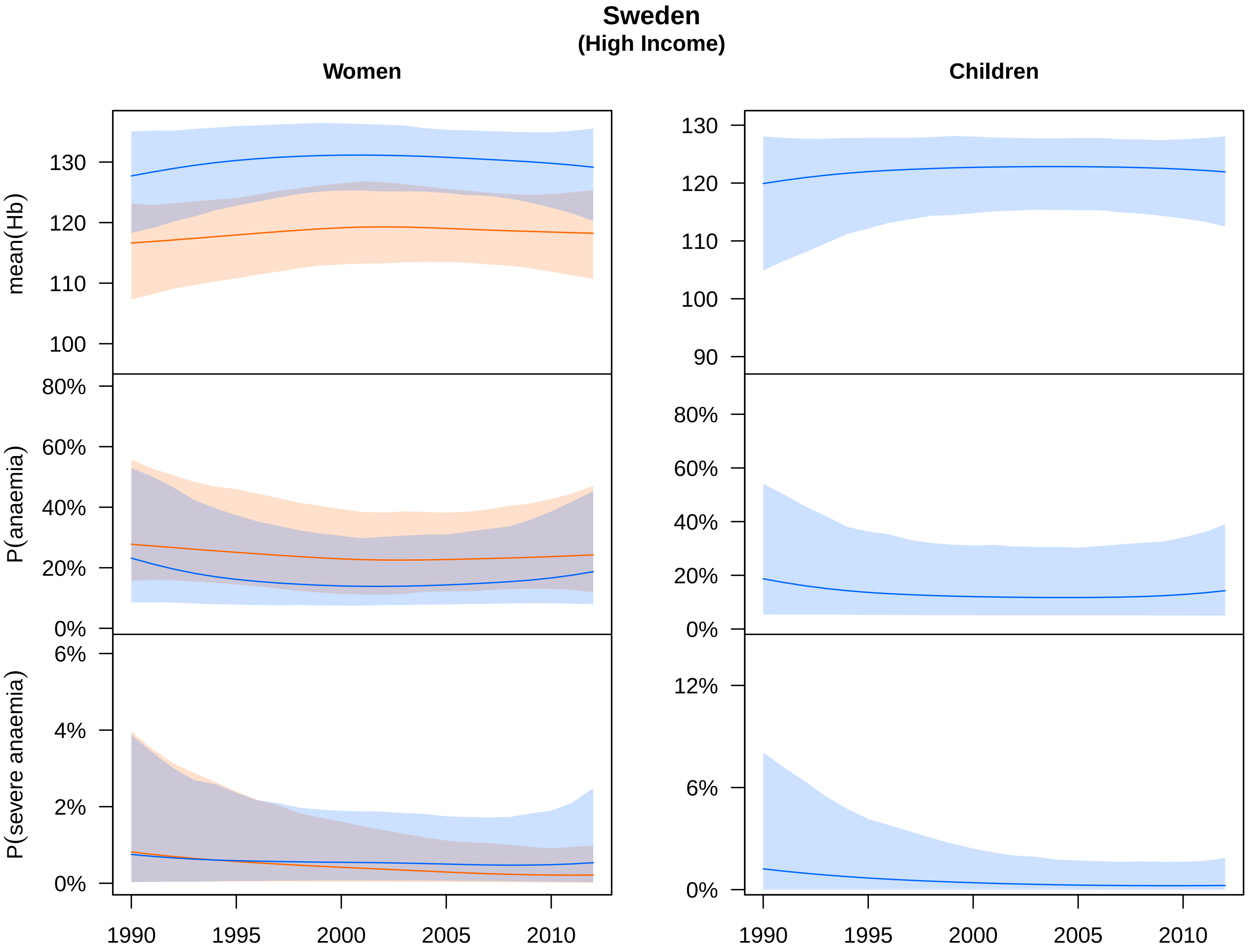
Sweden
(High Income)
Women
Children
mean(Hb)
P(anaemia)
P(severe anaemia)
130
120
110
100
90
80%
60%
40%
20%
0%
6%
4%
2%
12%
1990
1995
2000
2005
2010

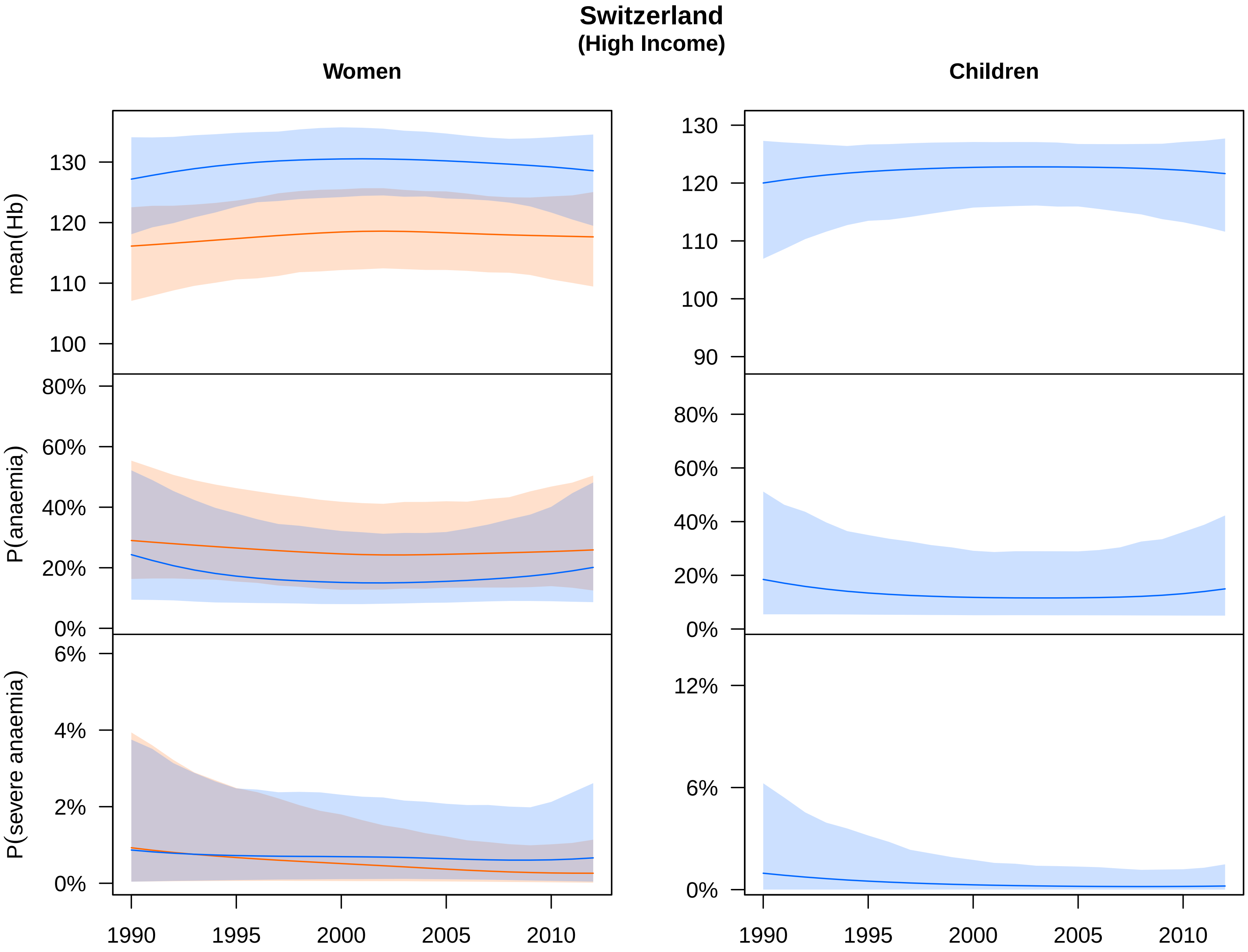
Switzerland
(High Income)
Women
Children
mean(Hb)
P(anaemia)
P(severe anaemia)
130
120
110
100
90
80%
60%
40%
20%
0%
6%
4%
2%
12%
1990
1995
2000
2005
2010

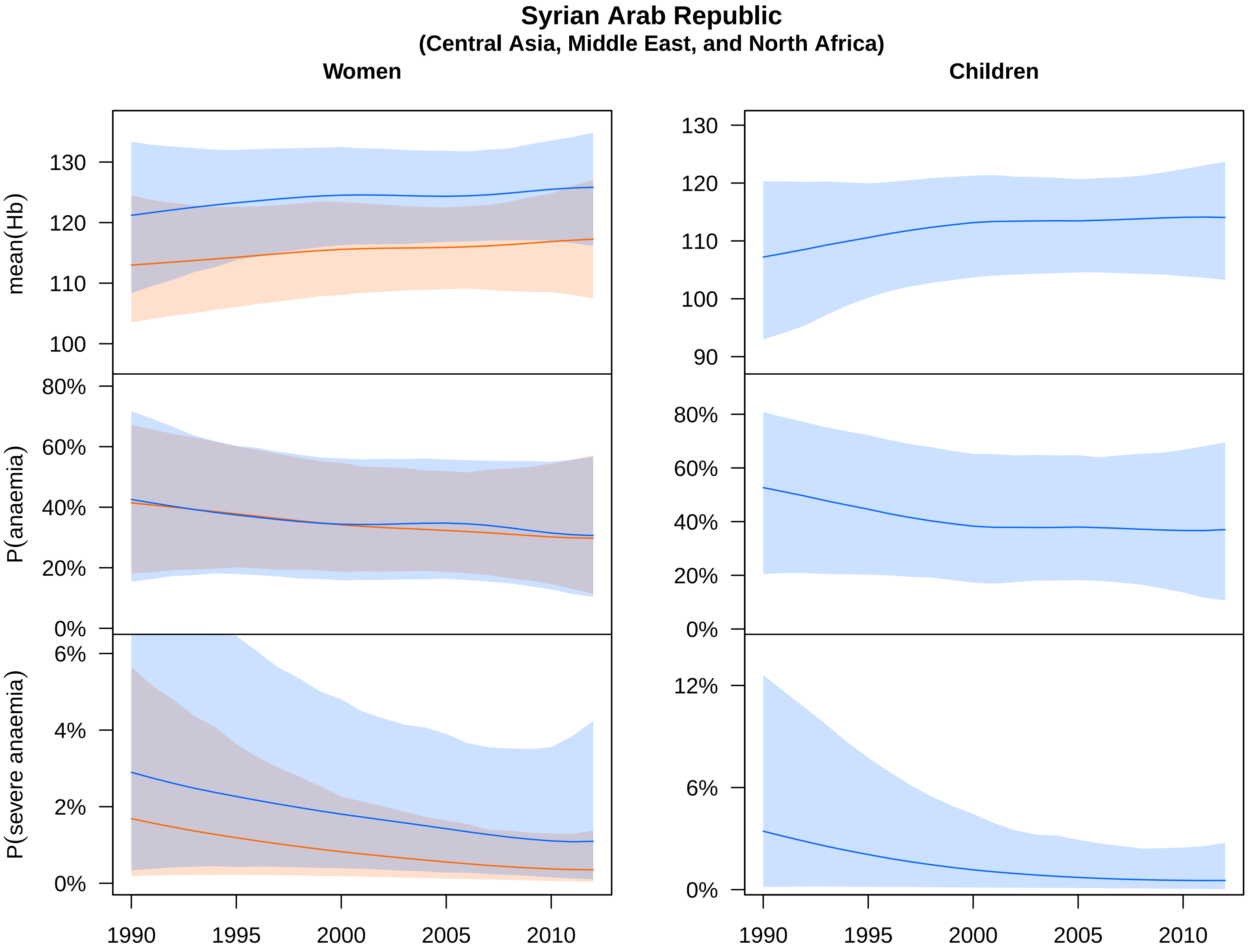

Syrian Arab Republic
(Central Asia, Middle East, and North Africa)
Women
Children
mean(Hb)
P(anaemia)
P(severe anaemia)
130
120
110
100
90
80%
60%
40%
20%
0%
6%
4%
2%
12%
1990
1995
2000
2005
2010

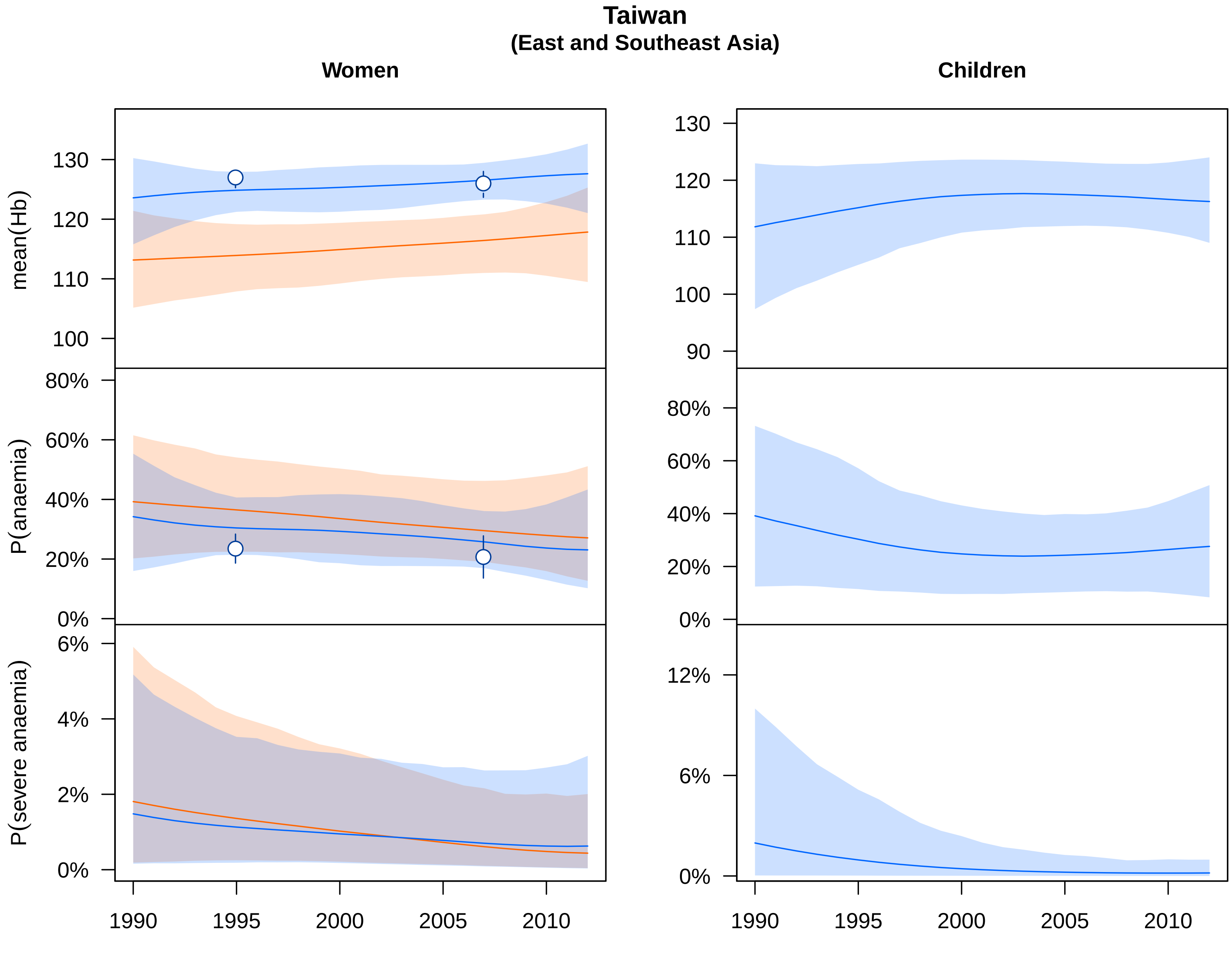
Taiwan
(East and Southeast Asia)
Women
Children
mean(Hb)
P(anaemia)
P(severe anaemia)
130
120
110
100
90
80%
60%
40%
20%
0%
6%
4%
2%
12%
1990
1995
2000
2005
2010

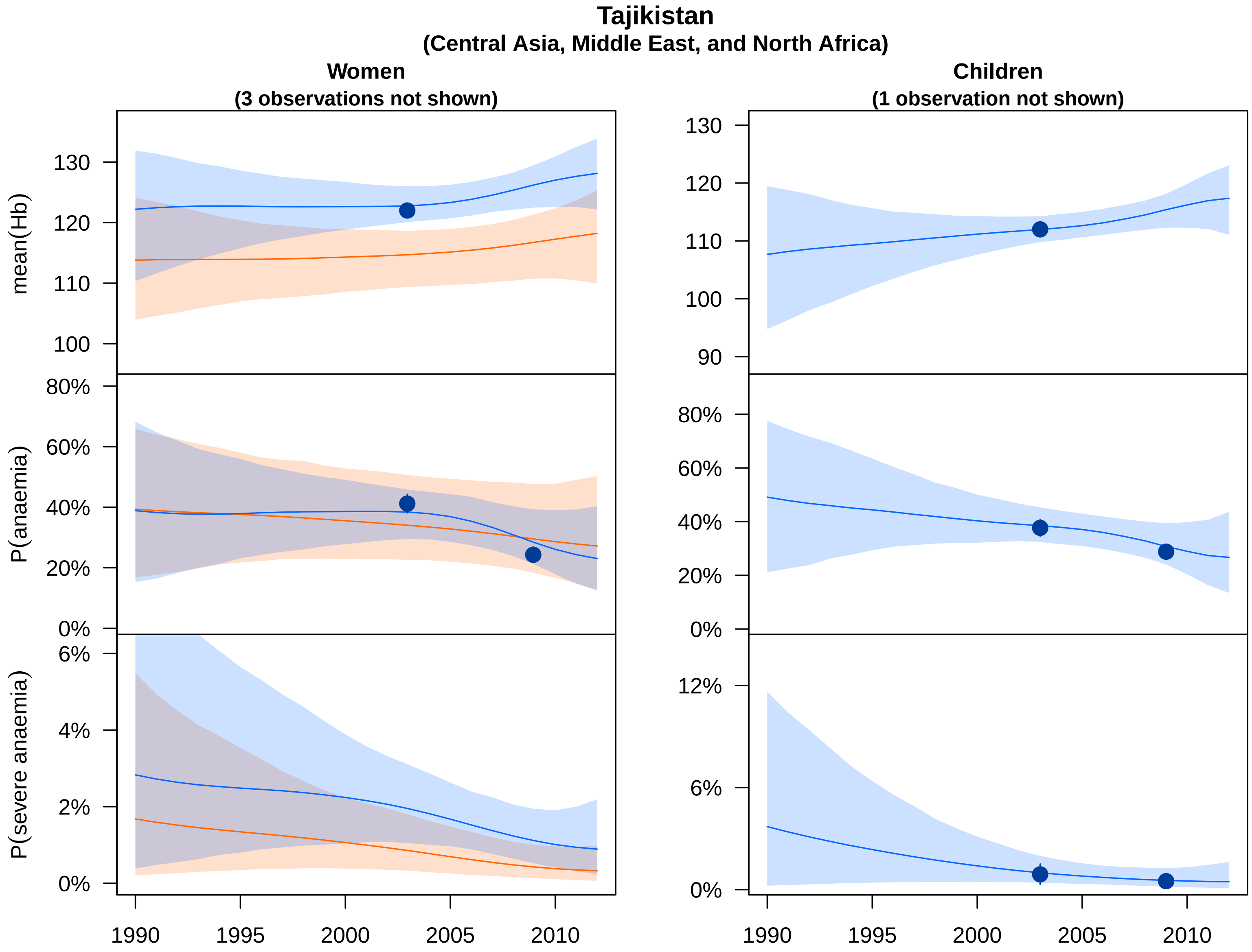
Tajikistan
(Central Asia, Middle East, and North Africa)
Women
(3 observations not shown)
Children
(1 observation not shown)
mean(Hb)
P(anaemia)
P(severe anaemia)
130
120
110
100
90
80%
60%
40%
20%
0%
6%
4%
2%
12%
1990
1995
2000
2005
2010

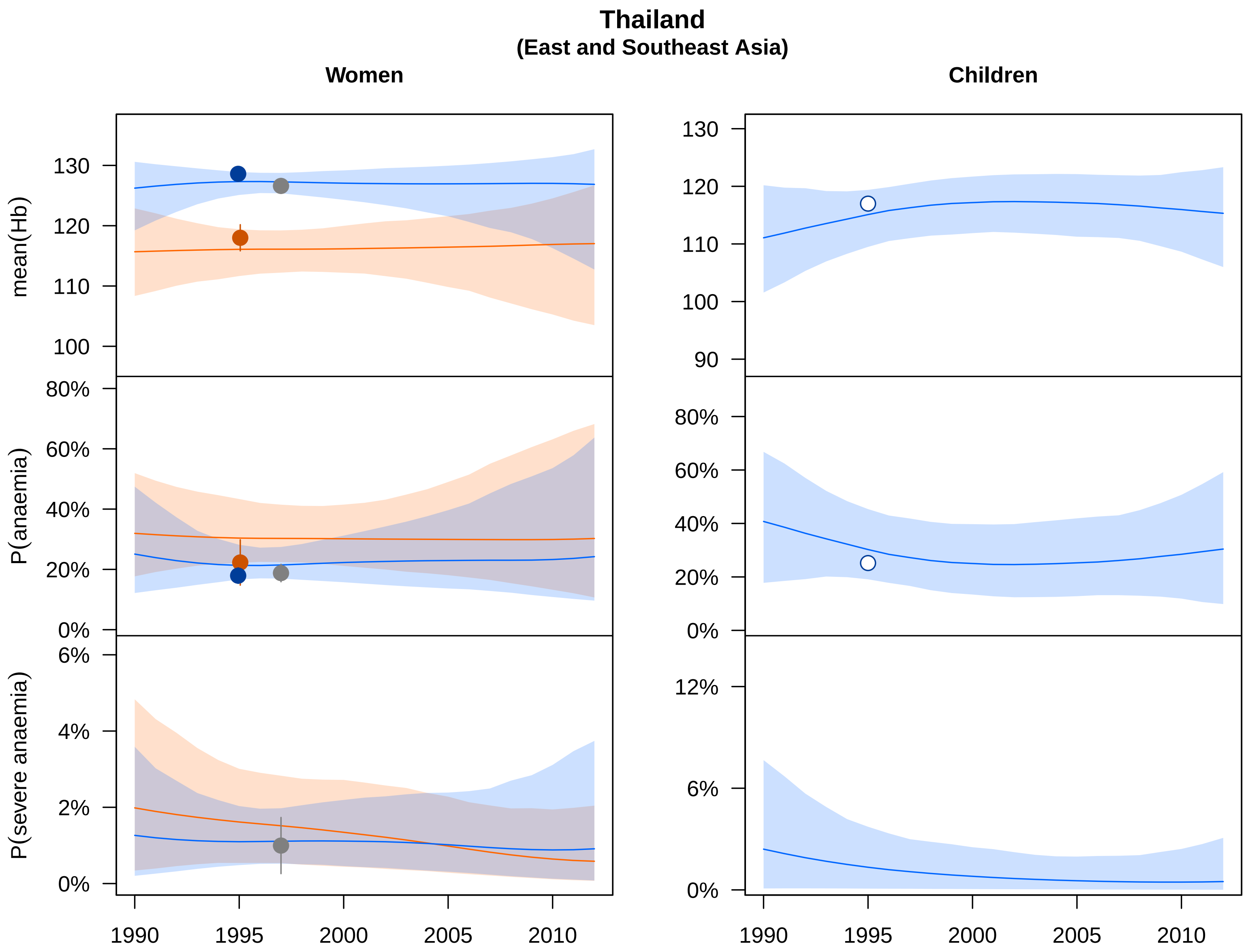
Thailand
(East and Southeast Asia)
Women
Children
mean(Hb)
P(anaemia)
P(severe anaemia)
130
120
110
100
90
80%
60%
40%
20%
0%
6%
4%
2%
12%
1990
1995
2000
2005
2010

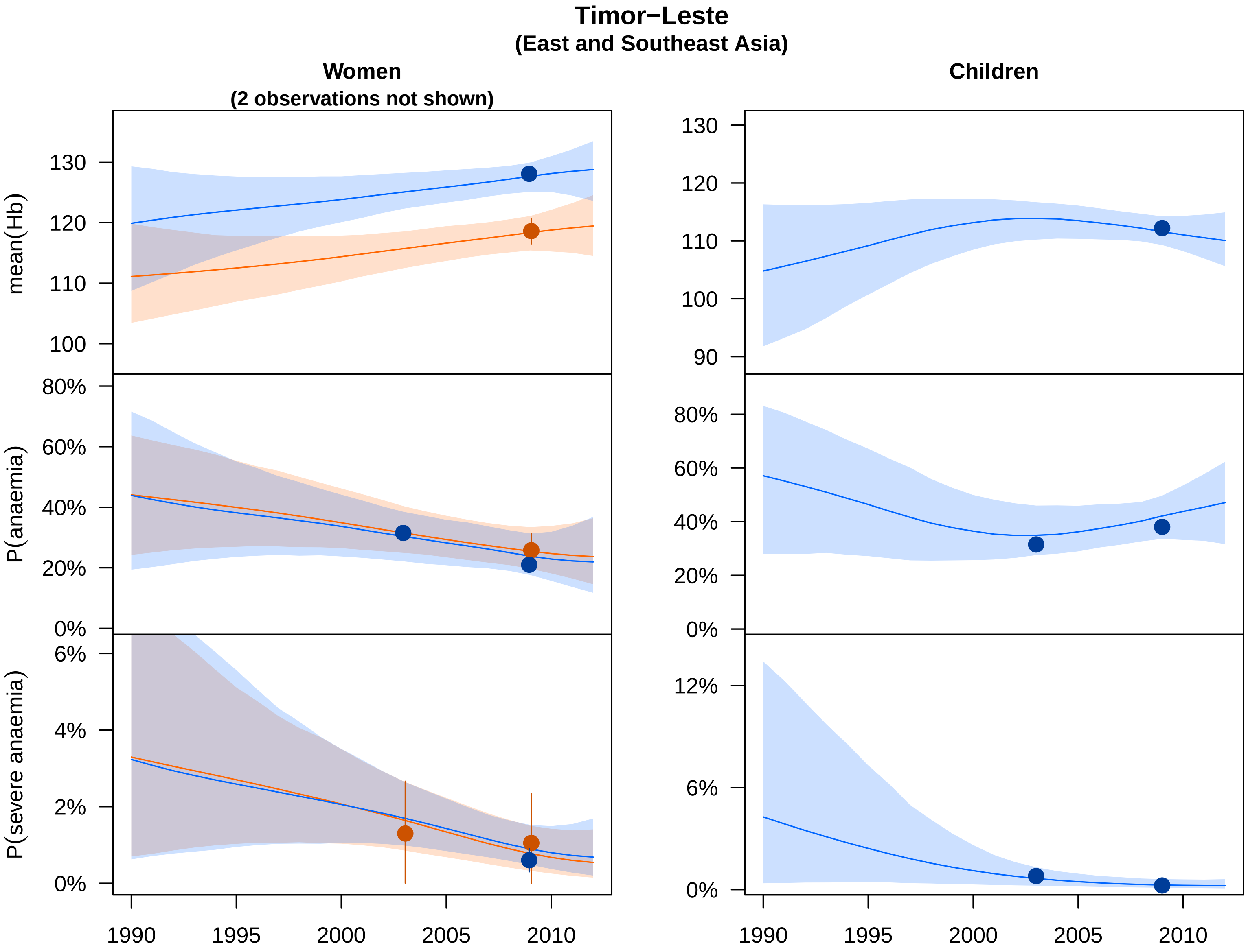
Timor−Leste
(East and Southeast Asia)
Women
(2 observations not shown)
Children
mean(Hb)
P(anaemia)
P(severe anaemia)
130
120
110
100
90
80%
60%
40%
20%
0%
6%
4%
2%
12%
1990
1995
2000
2005
2010

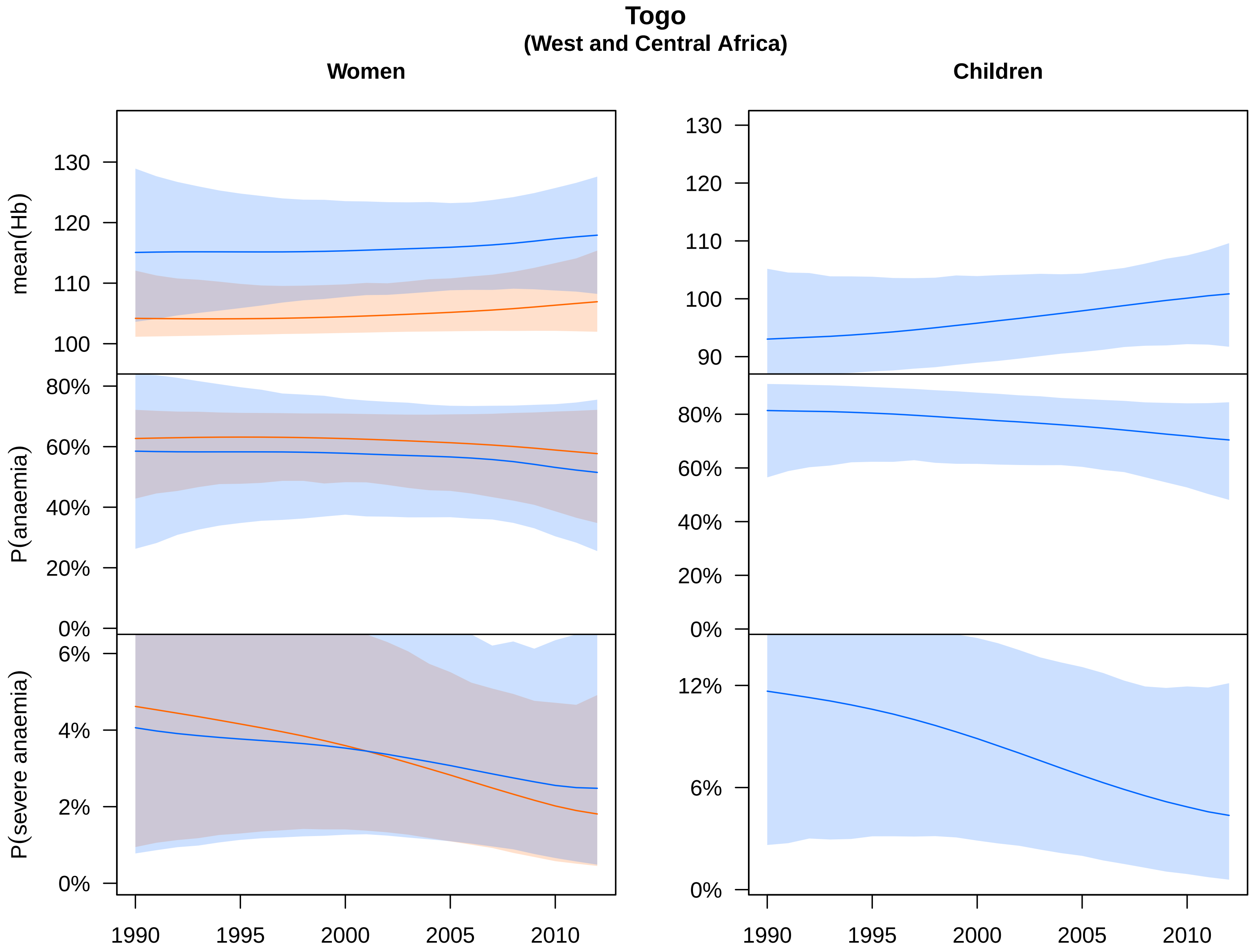
Togo
(West and Central Africa)
Women
Children
mean(Hb)
P(anaemia)
P(severe anaemia)
130
120
110
100
90
80%
60%
40%
20%
0%
6%
4%
2%
12%
1990
1995
2000
2005
2010

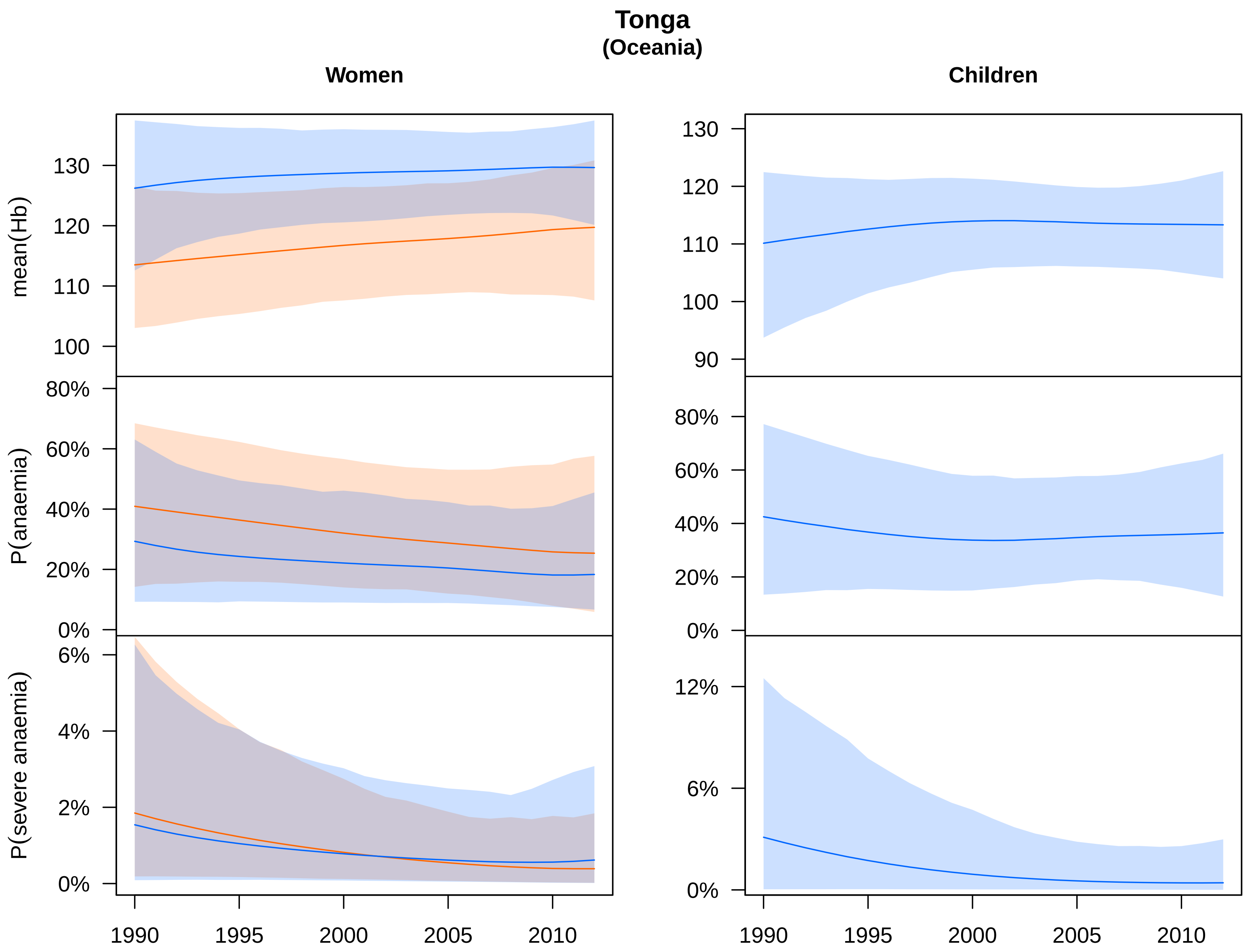
Tonga
(Oceania)
Women
Children
mean(Hb)
P(anaemia)
P(severe anaemia)
130
120
110
100
90
80%
60%
40%
20%
0%
6%
4%
2%
12%
1990
1995
2000
2005
2010

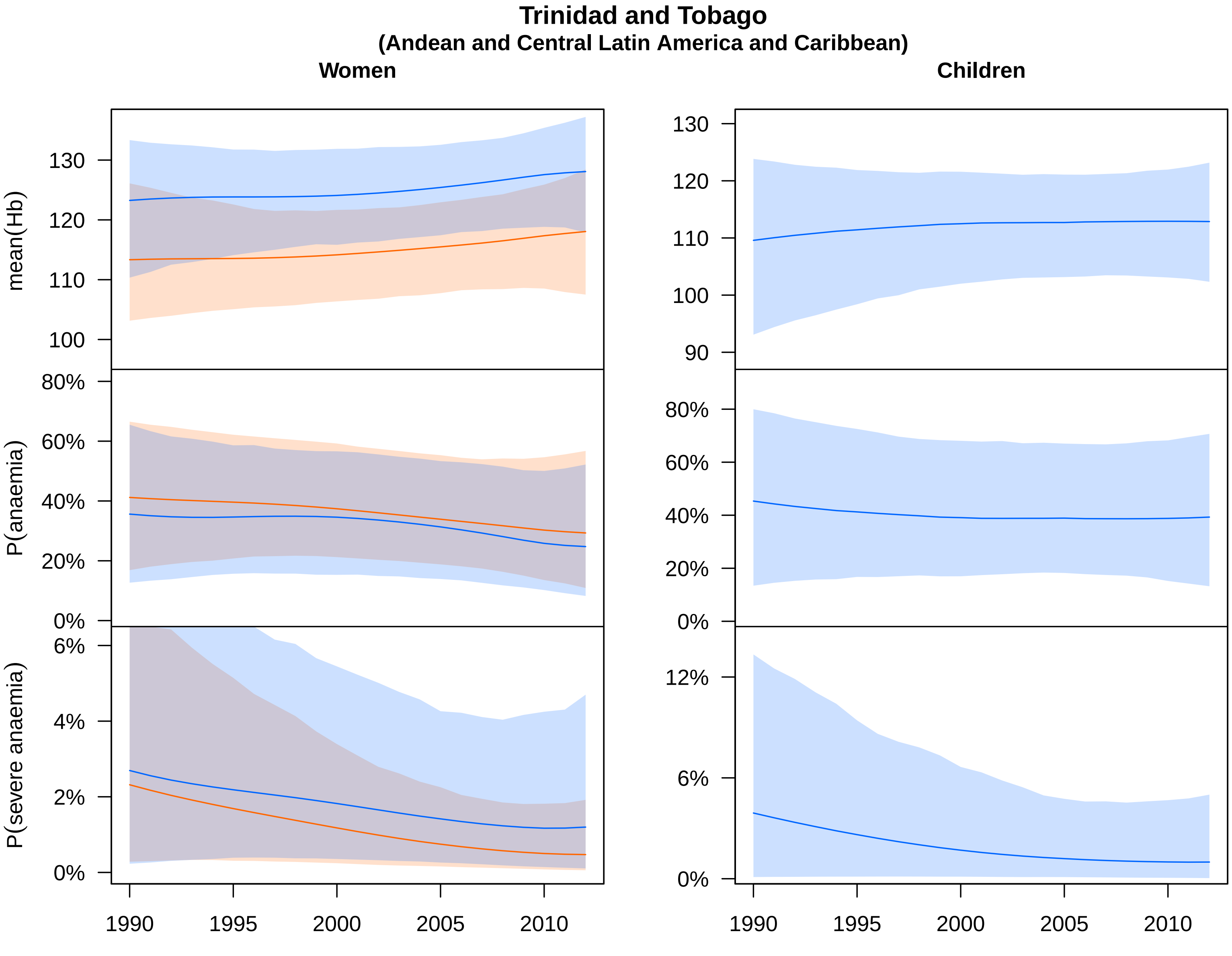
Trinidad and Tobago
(Andean and Central Latin America and Caribbean)
Women
Children
mean(Hb)
P(anaemia)
P(severe anaemia)
130
120
110
100
90
80%
60%
40%
20%
0%
6%
4%
2%
12%
1990
1995
2000
2005
2010

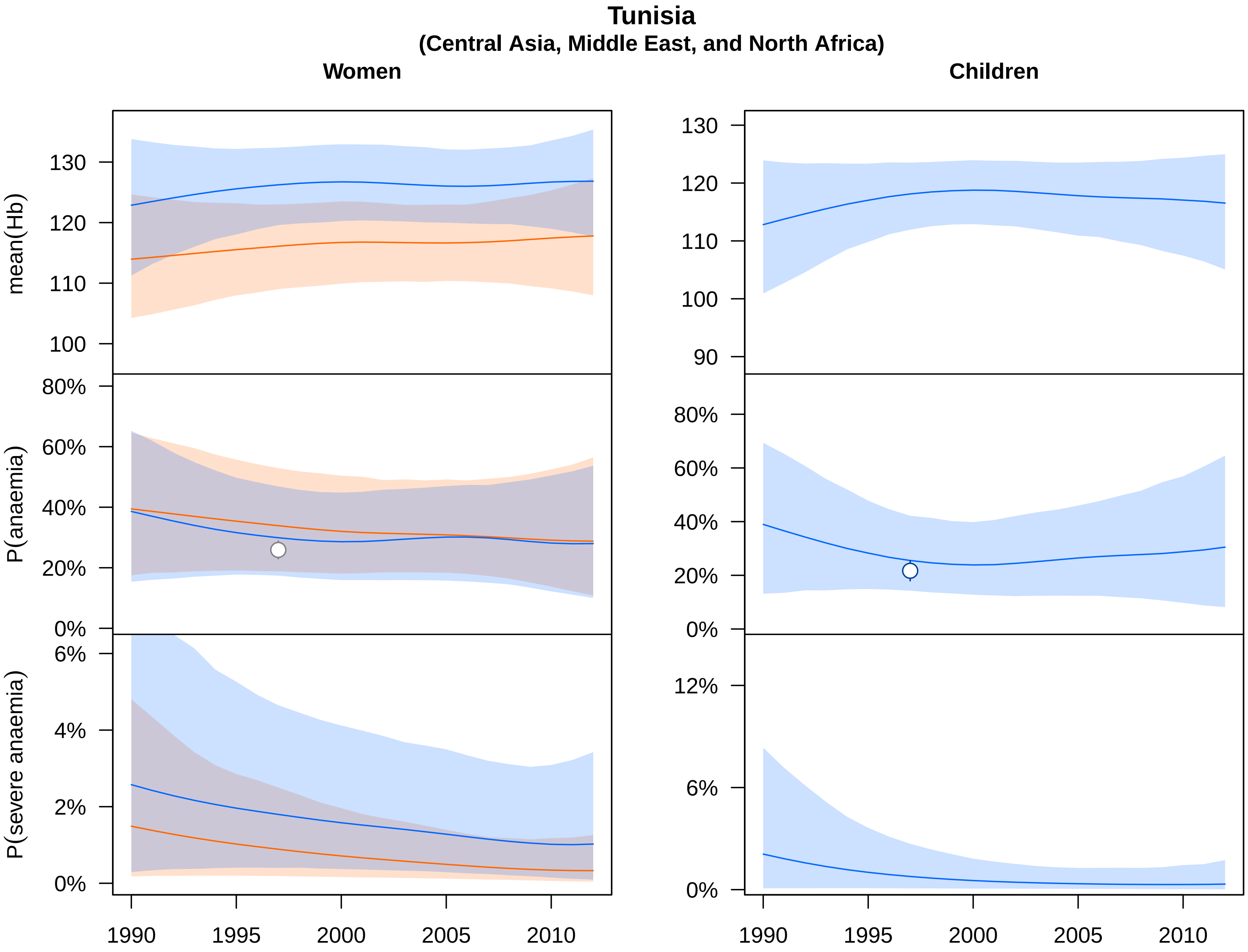
Tunisia
(Central Asia, Middle East, and North Africa)
Women
Children
mean(Hb)
P(anaemia)
P(severe anaemia)
130
120
110
100
90
80%
60%
40%
20%
0%
6%
4%
2%
12%
1990
1995
2000
2005
2010

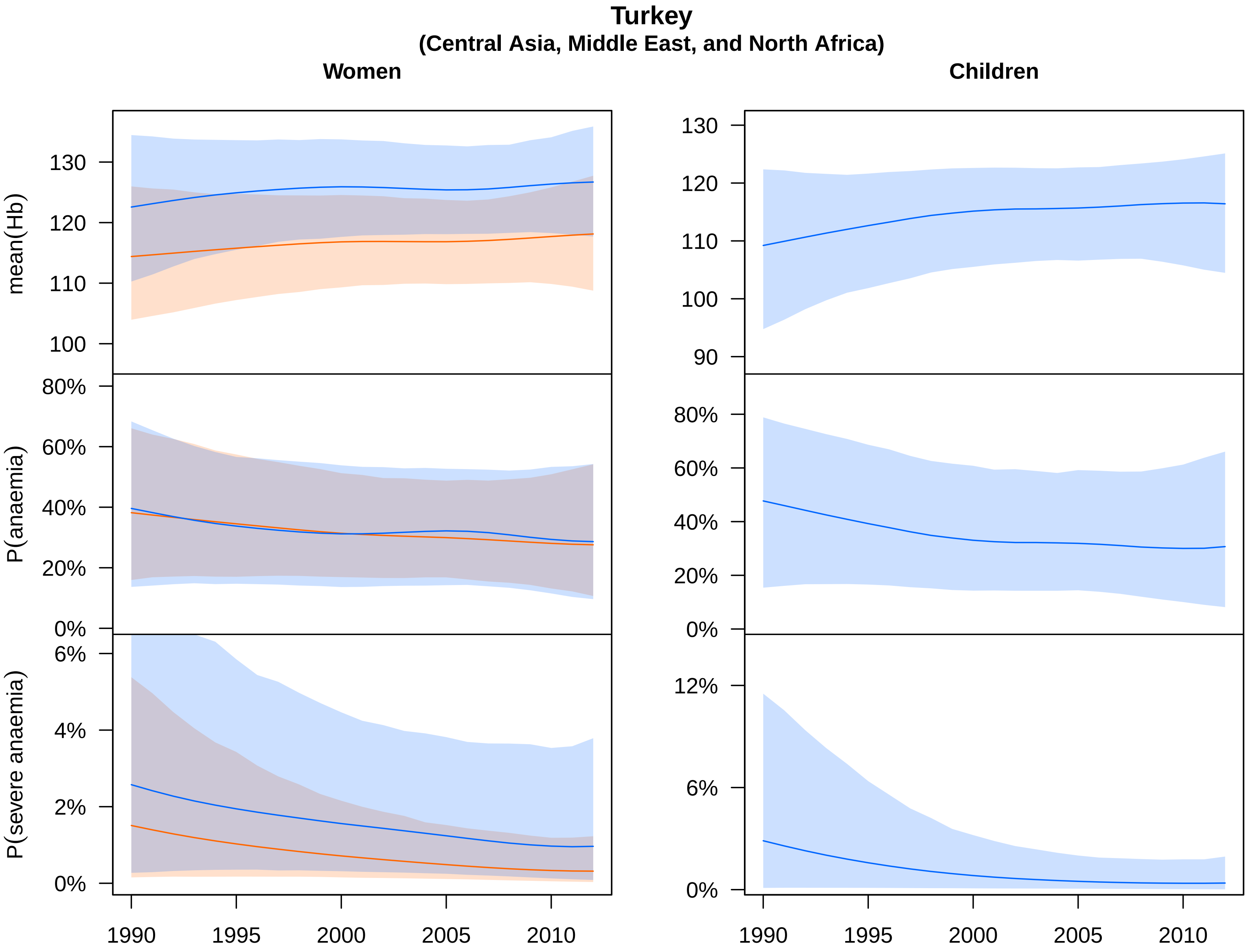
Turkey
(Central Asia, Middle East, and North Africa)
Women
Children
mean(Hb)
130
120
110
100
90
P(anaemia)
80%
60%
40%
20%
0%
P(severe anaemia)
6%
4%
2%
12%
1990
1995
2000
2005
2010

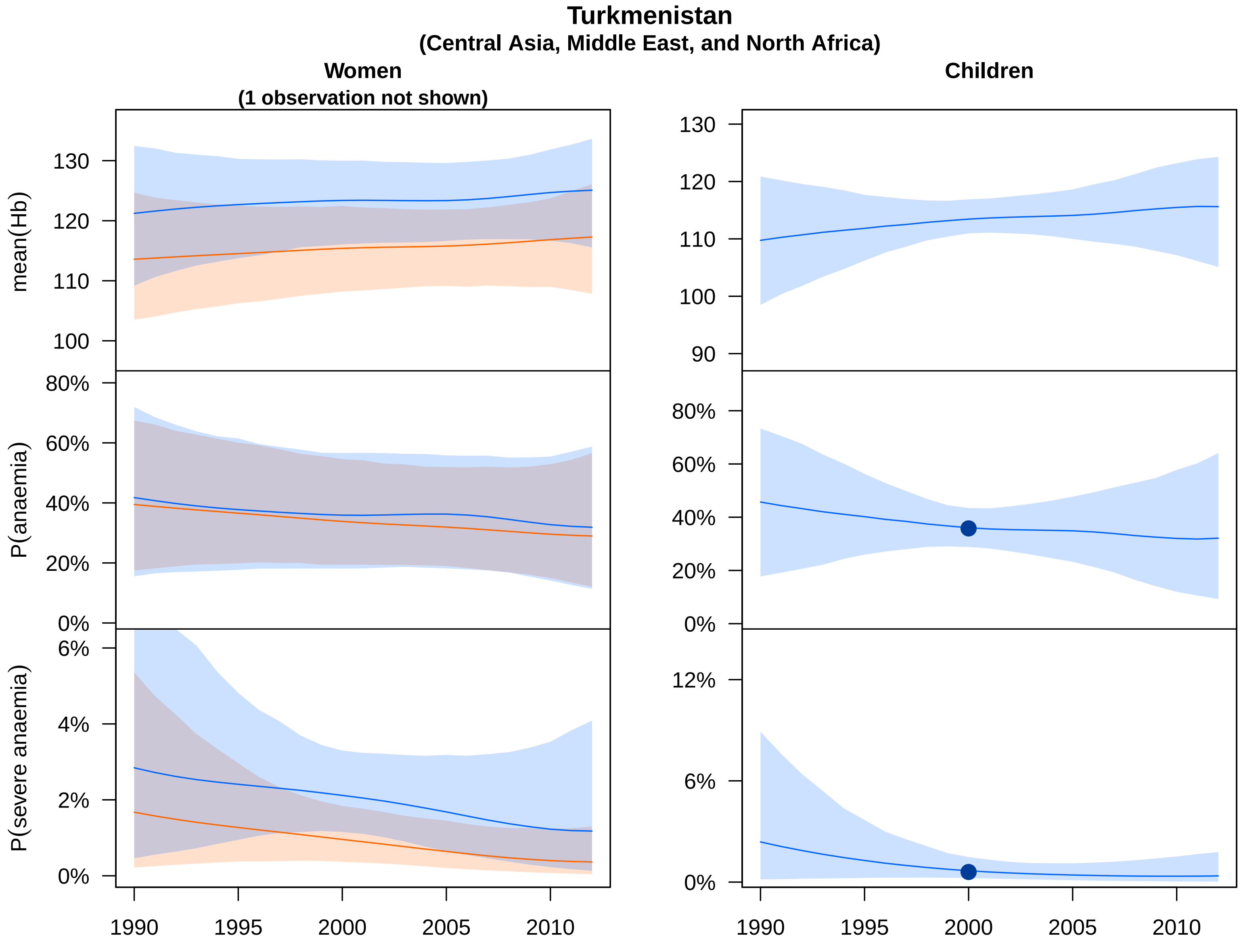
Turkmenistan
(Central Asia, Middle East, and North Africa)
Women
(1 observation not shown)
Children
mean(Hb)
P(anaemia)
P(severe anaemia)
130
120
110
100
90
80%
60%
40%
20%
0%
6%
4%
2%
12%
1990
1995
2000
2005
2010

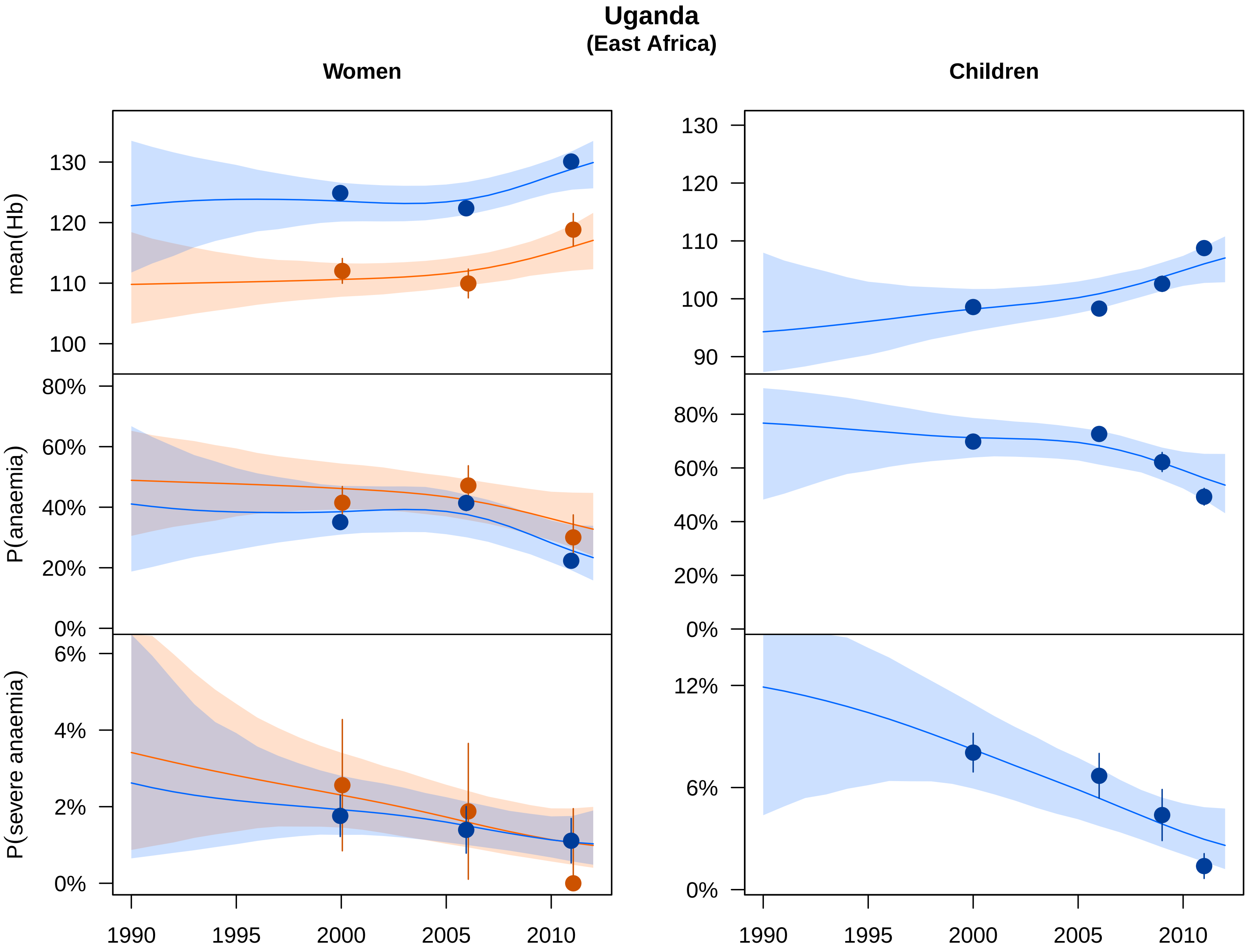
Uganda
(East Africa)
Women
Children
mean(Hb)
P(anaemia)
P(severe anaemia)
130
120
110
100
90
80%
60%
40%
20%
0%
6%
4%
2%
12%
1990
1995
2000
2005
2010

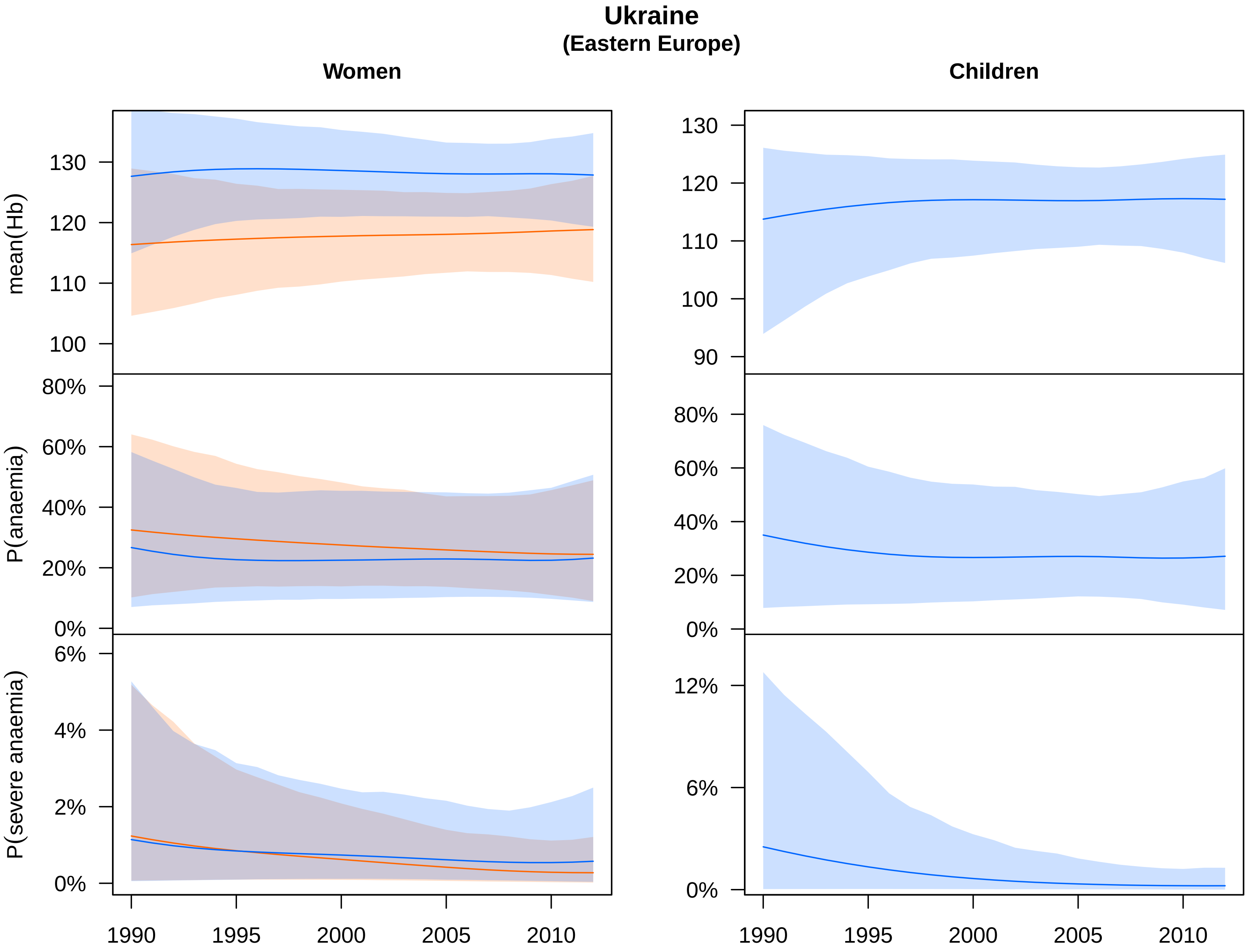

Ukraine
(Eastern Europe)
Women
Children
mean(Hb)
P(anaemia)
P(severe anaemia)
100
110
120
130
90
0%
20%
40%
60%
80%
2%
4%
6%
12%
1990
1995
2000
2005
2010

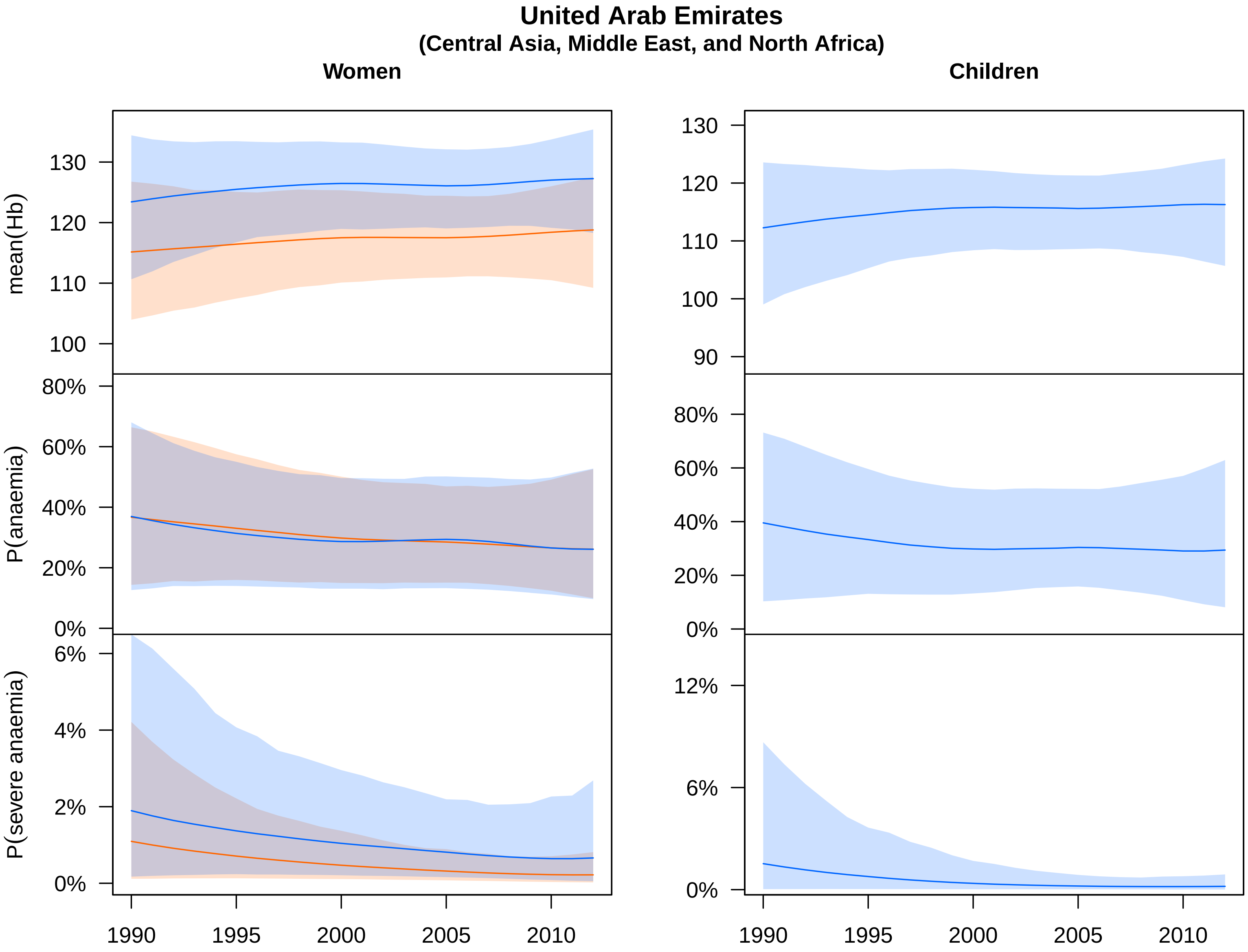

United Arab Emirates
(Central Asia, Middle East, and North Africa)
Women
Children
mean(Hb)
P(anaemia)
P(severe anaemia)
130
120
110
100
90
80%
60%
40%
20%
0%
6%
4%
2%
12%
1990
1995
2000
2005
2010

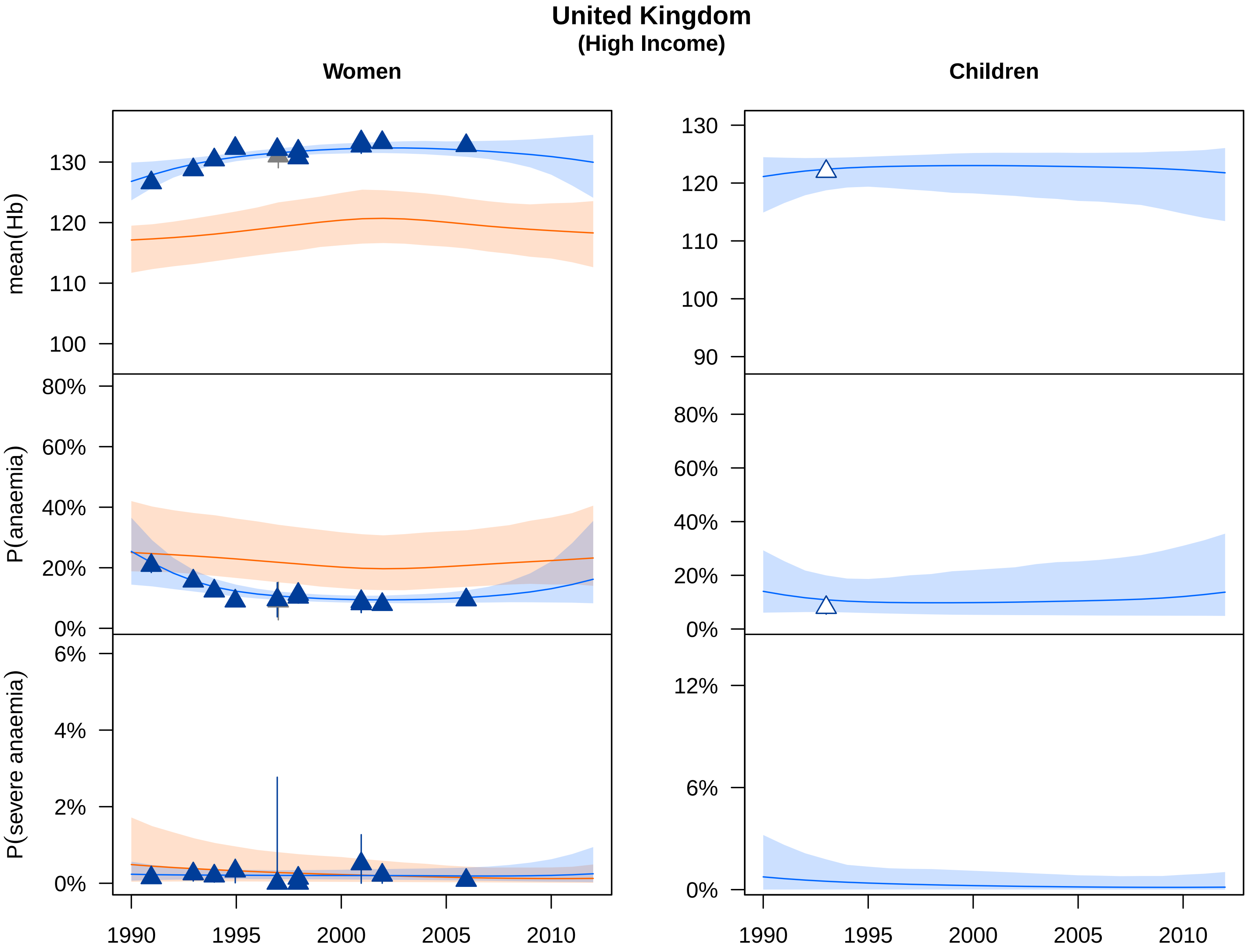
United Kingdom
(High Income)
Women
Children
mean(Hb)
P(anaemia)
P(severe anaemia)
130
120
110
100
90
80%
60%
40%
20%
0%
6%
4%
2%
12%
1990
1995
2000
2005
2010

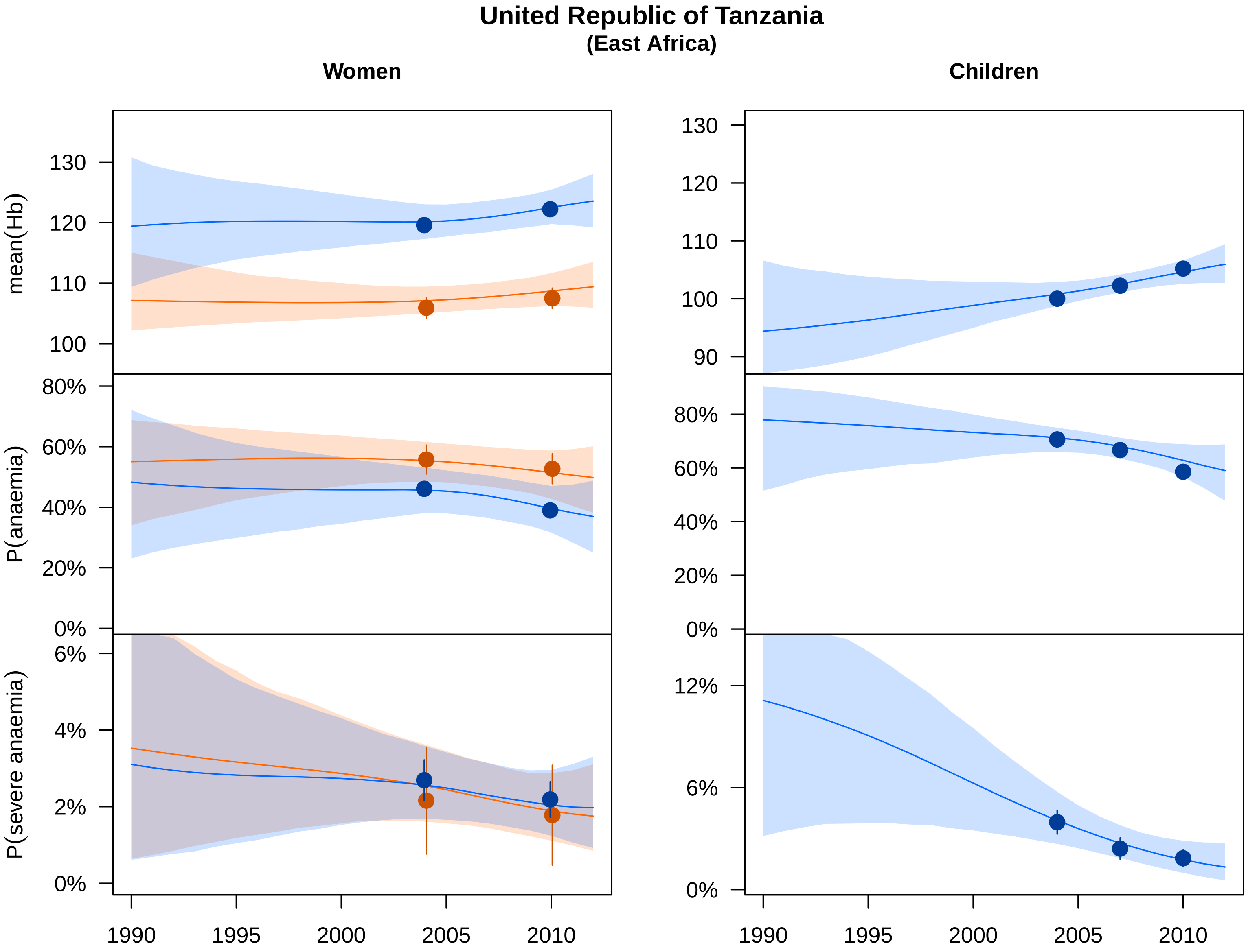

United Republic of Tanzania
(East Africa)
Women
Children
mean(Hb)
P(anaemia)
P(severe anaemia)
130
120
110
100
90
80%
60%
40%
20%
0%
6%
4%
2%
12%
1990
1995
2000
2005
2010

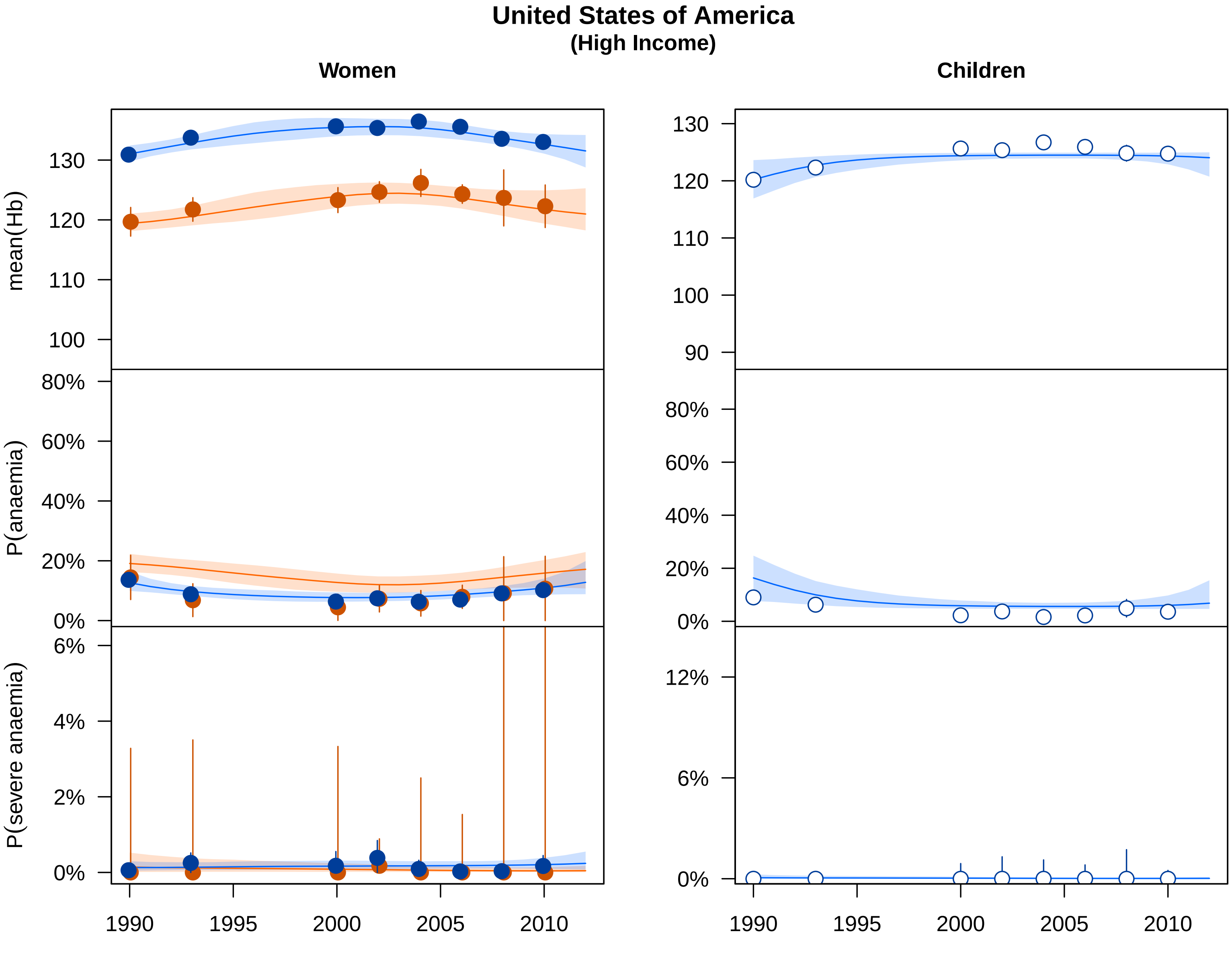
United States of America
(High Income)
Women
Children
mean(Hb)
P(anaemia)
P(severe anaemia)
130
120
110
100
90
80%
60%
40%
20%
0%
6%
4%
2%
12%
1990
1995
2000
2005
2010

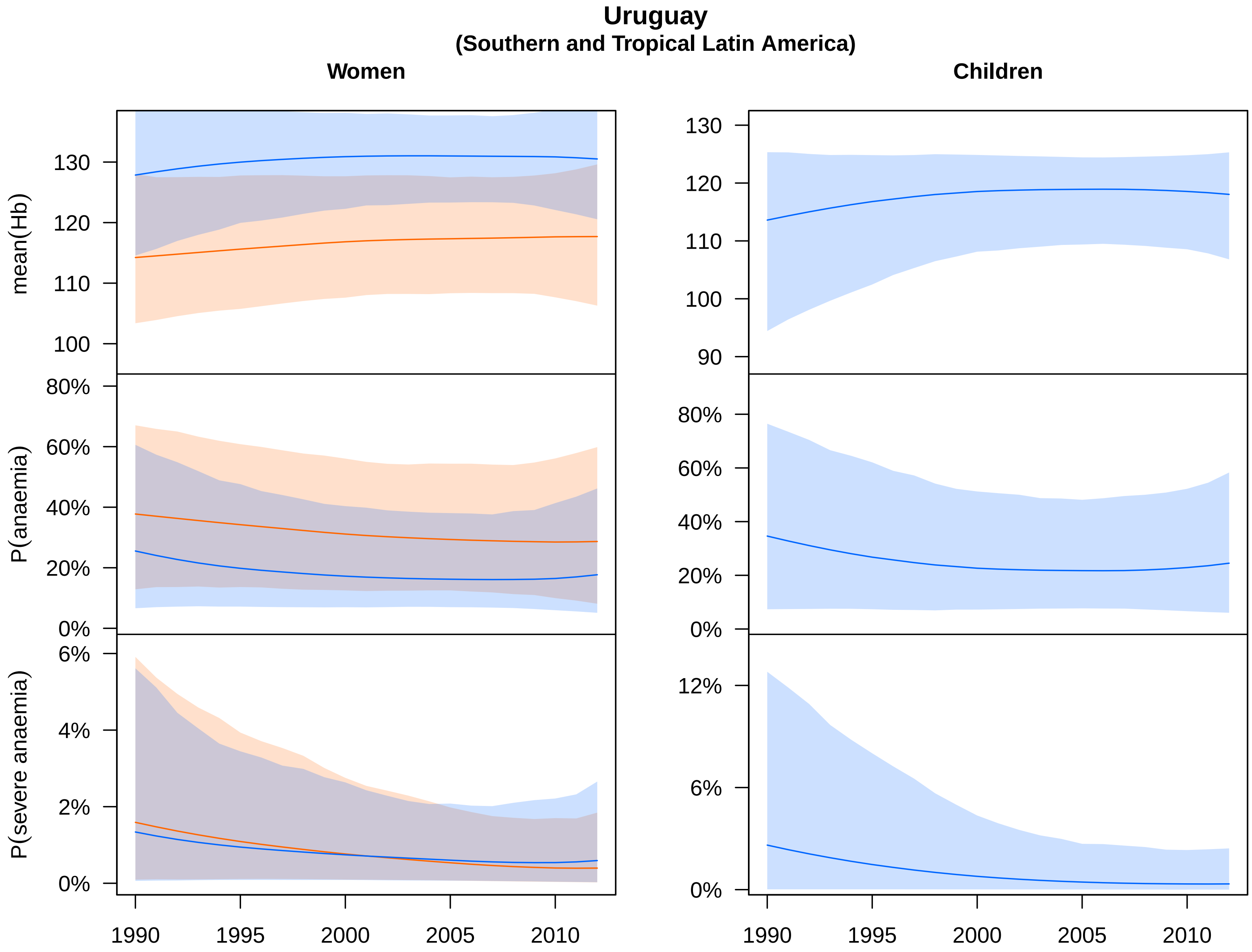

Uruguay
(Southern and Tropical Latin America)
Women
Children
mean(Hb)
P(anaemia)
P(severe anaemia)
130
120
110
100
90
80%
60%
40%
20%
0%
6%
4%
2%
12%
1990
1995
2000
2005
2010

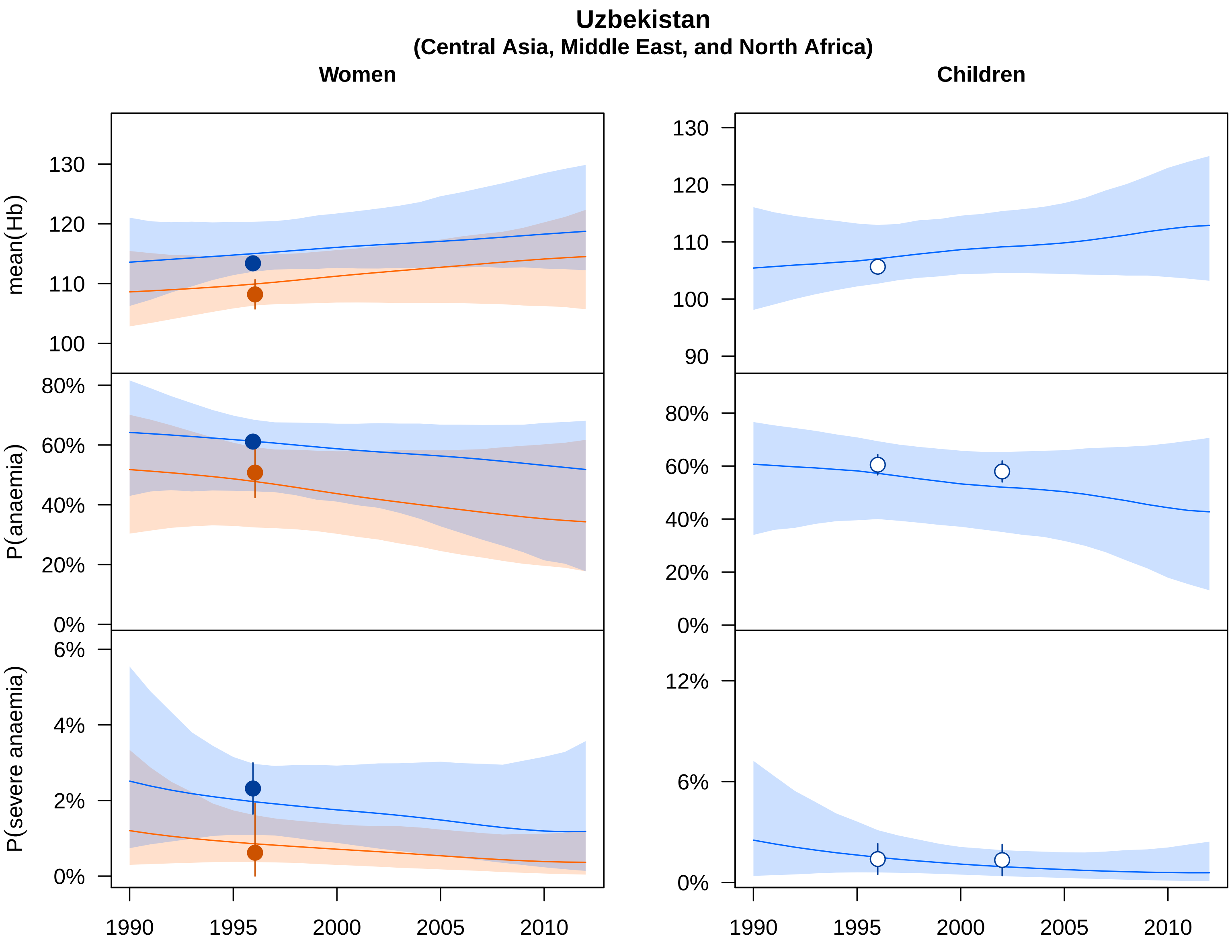
Uzbekistan
(Central Asia, Middle East, and North Africa)
Women
Children
mean(Hb)
P(anaemia)
P(severe anaemia)
130
120
110
100
90
80%
60%
40%
20%
0%
6%
4%
2%
12%
1990
1995
2000
2005
2010

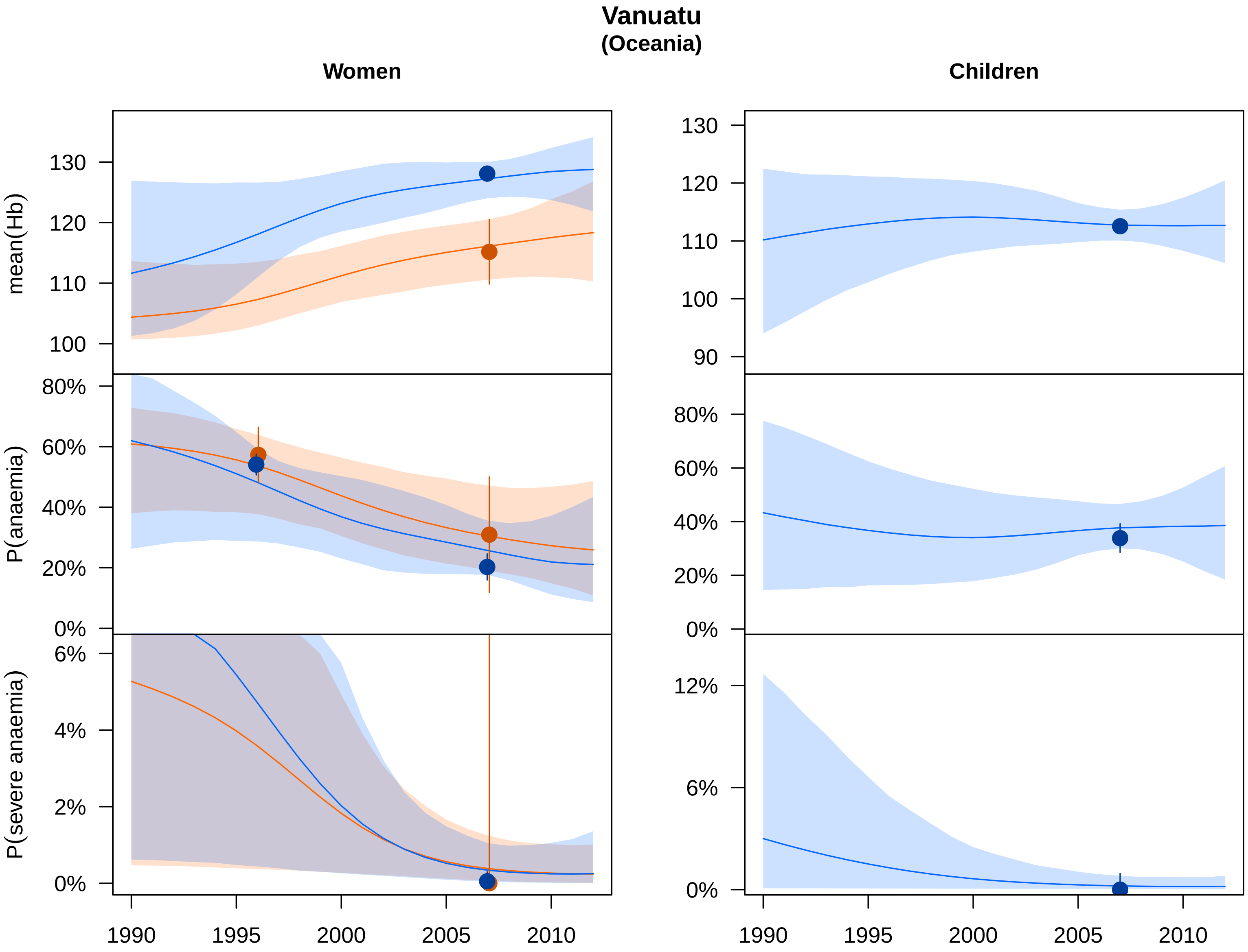

Vanuatu
(Oceania)
Women
Children
mean(Hb)
P(anaemia)
P(severe anaemia)
130
120
110
100
90
80%
60%
40%
20%
0%
6%
4%
2%
12%
1990
1995
2000
2005
2010

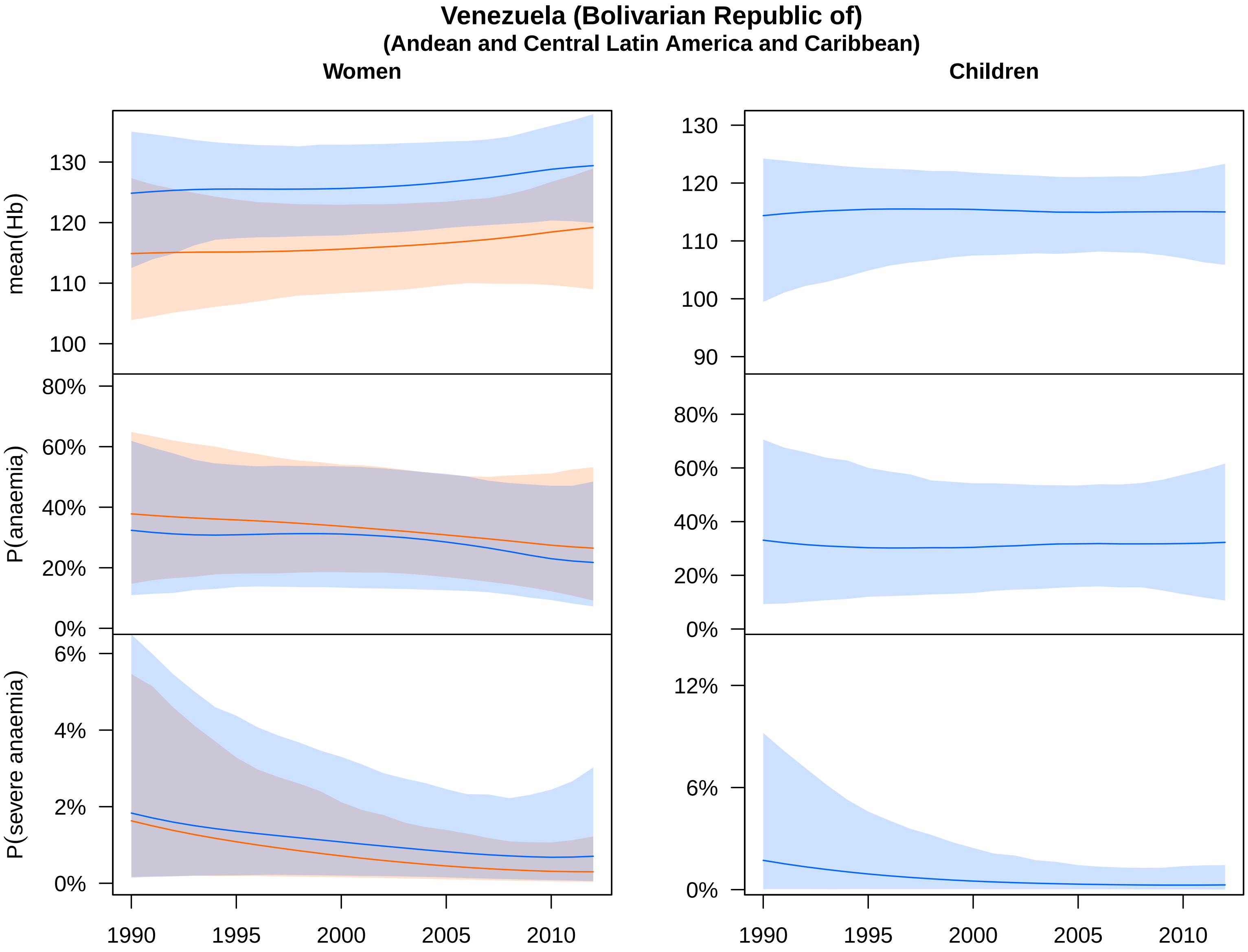
Venezuela (Bolivarian Republic of)
(Andean and Central Latin America and Caribbean)
Women
Children
mean(Hb)
P(anaemia)
P(severe anaemia)
100
110
120
130
90
0%
20%
40%
60%
80%
2%
4%
6%
12%
1990
1995
2000
2005
2010

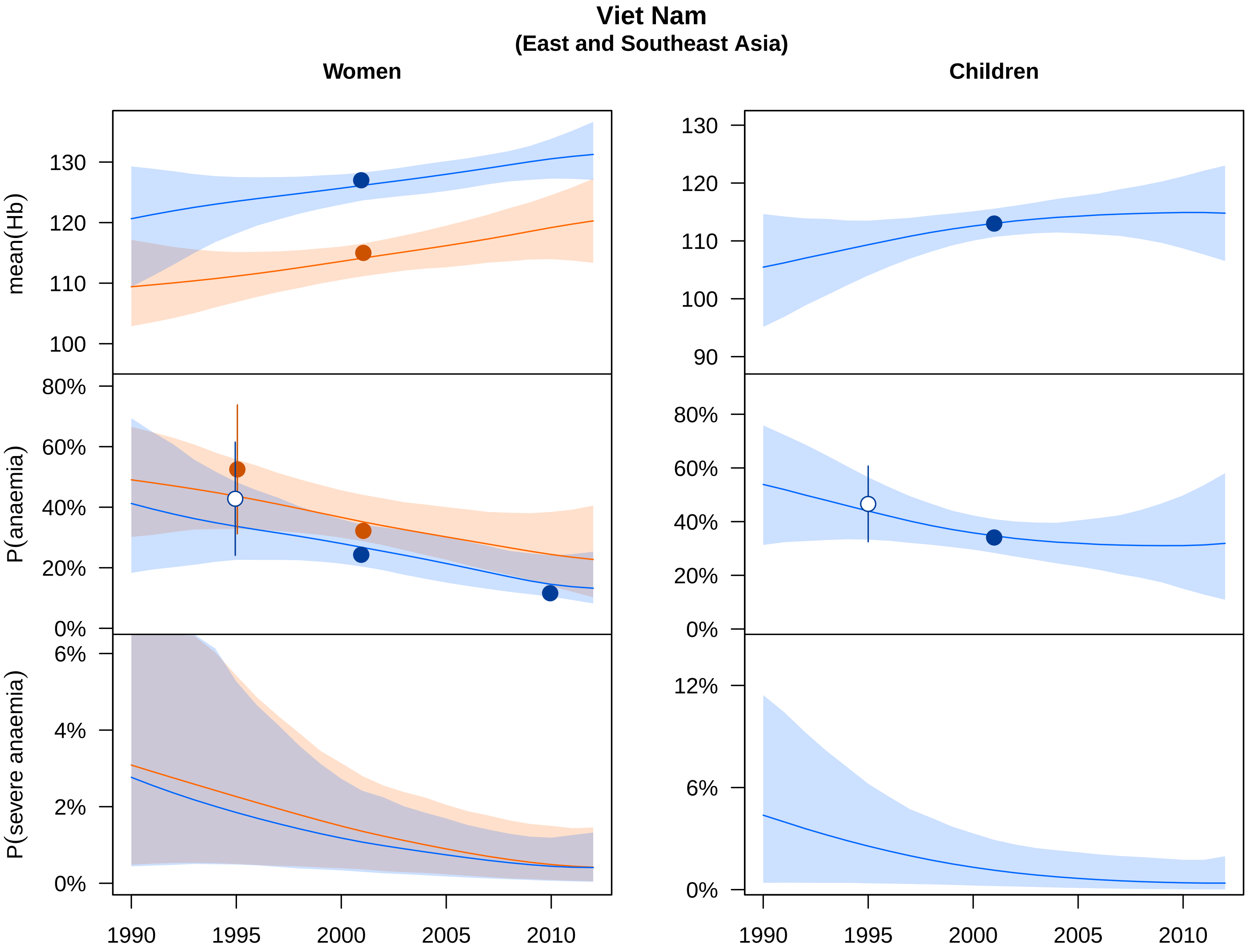

Viet Nam
(East and Southeast Asia)
Women
Children
mean(Hb)
P(anaemia)
P(severe anaemia)
130
120
110
100
90
80%
60%
40%
20%
0%
6%
4%
2%
12%
1990
1995
2000
2005
2010

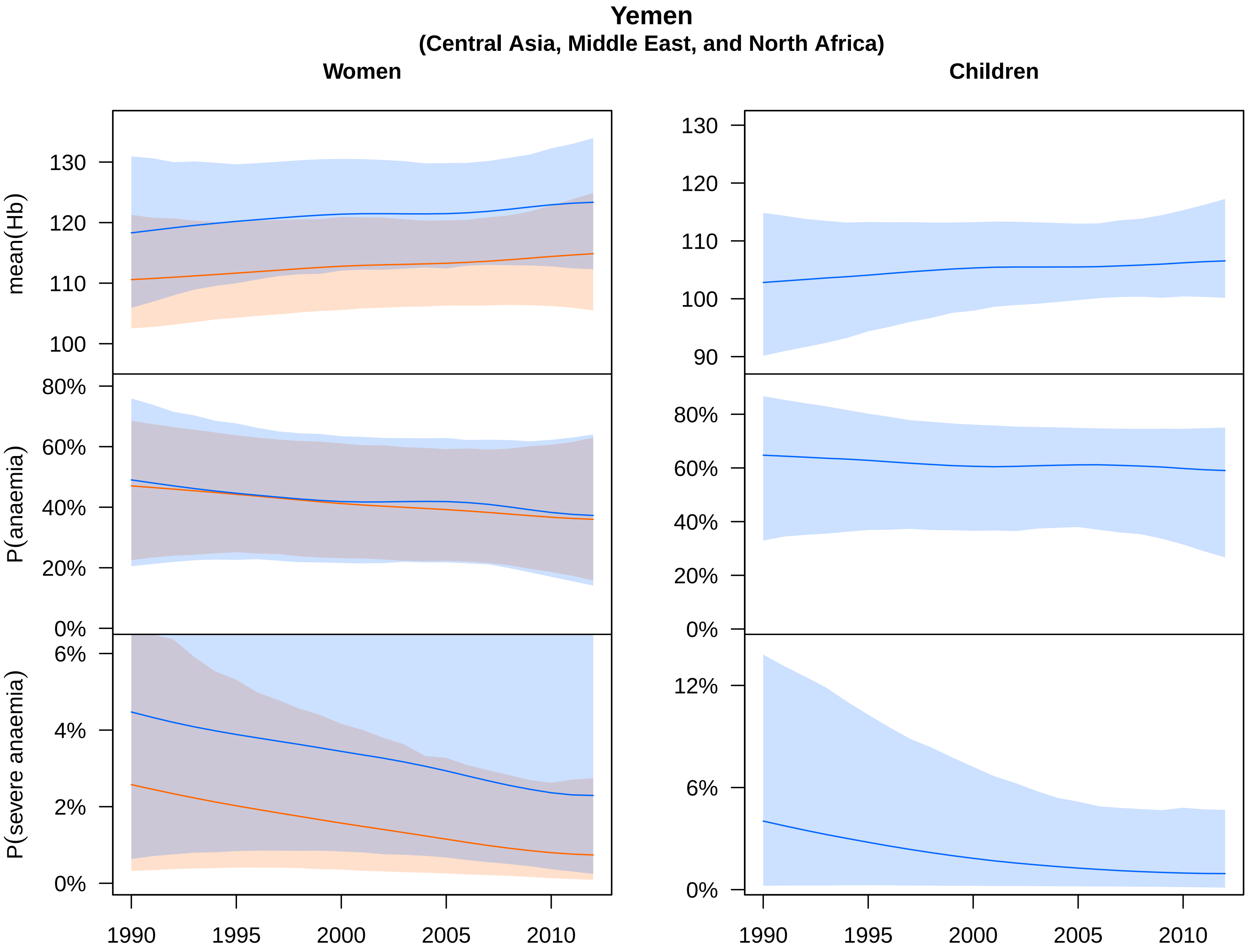
Yemen
(Central Asia, Middle East, and North Africa)
Women
Children
mean(Hb)
P(anaemia)
P(severe anaemia)
130
120
110
100
90
80%
60%
40%
20%
0%
6%
4%
2%
12%
1990
1995
2000
2005
2010

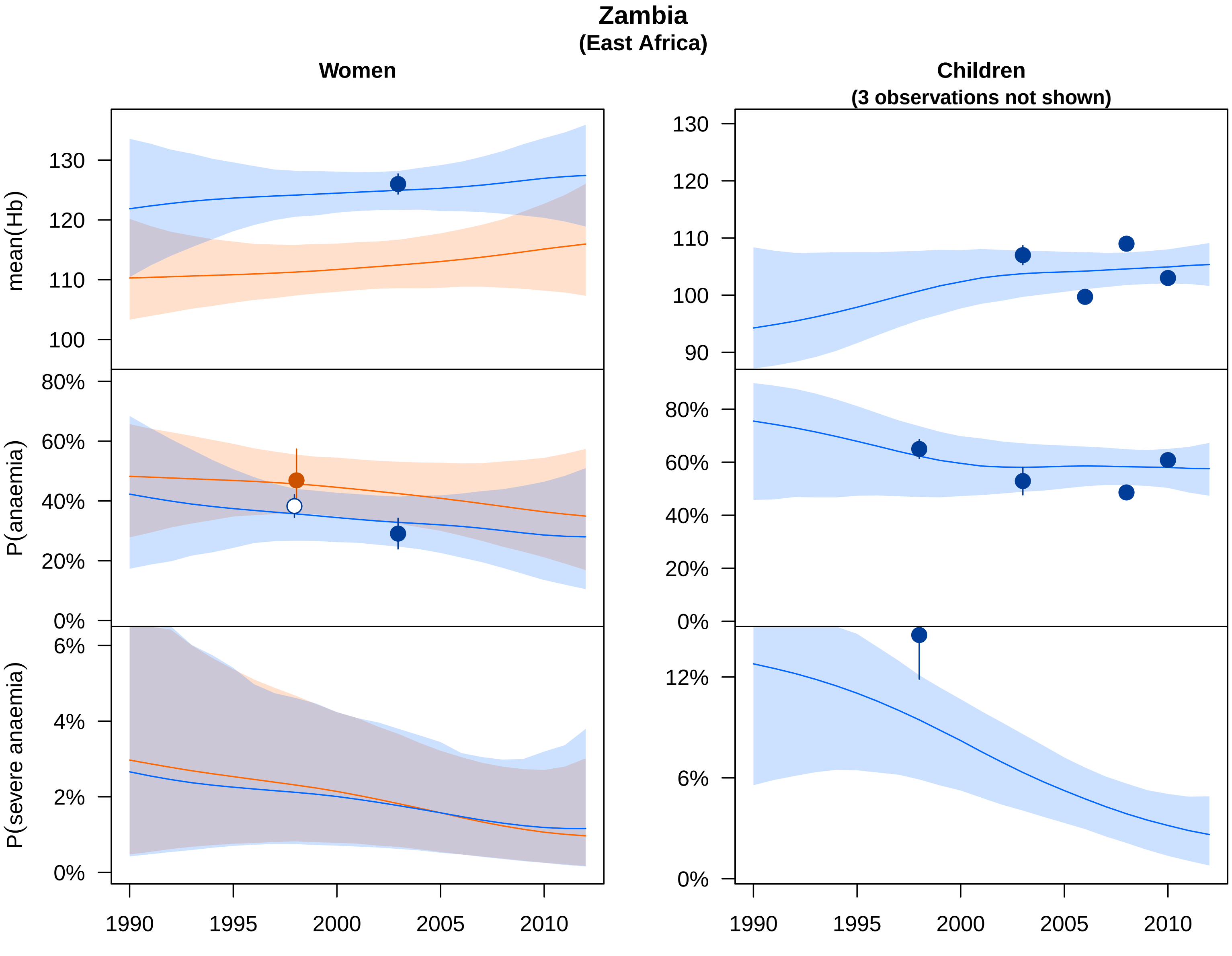

Zambia
(East Africa)
Women
Children
(3 observations not shown)
mean(Hb)
P(anaemia)
P(severe anaemia)
130
120
110
100
90
80%
60%
40%
20%
0%
6%
4%
2%
12%
1990
1995
2000
2005
2010

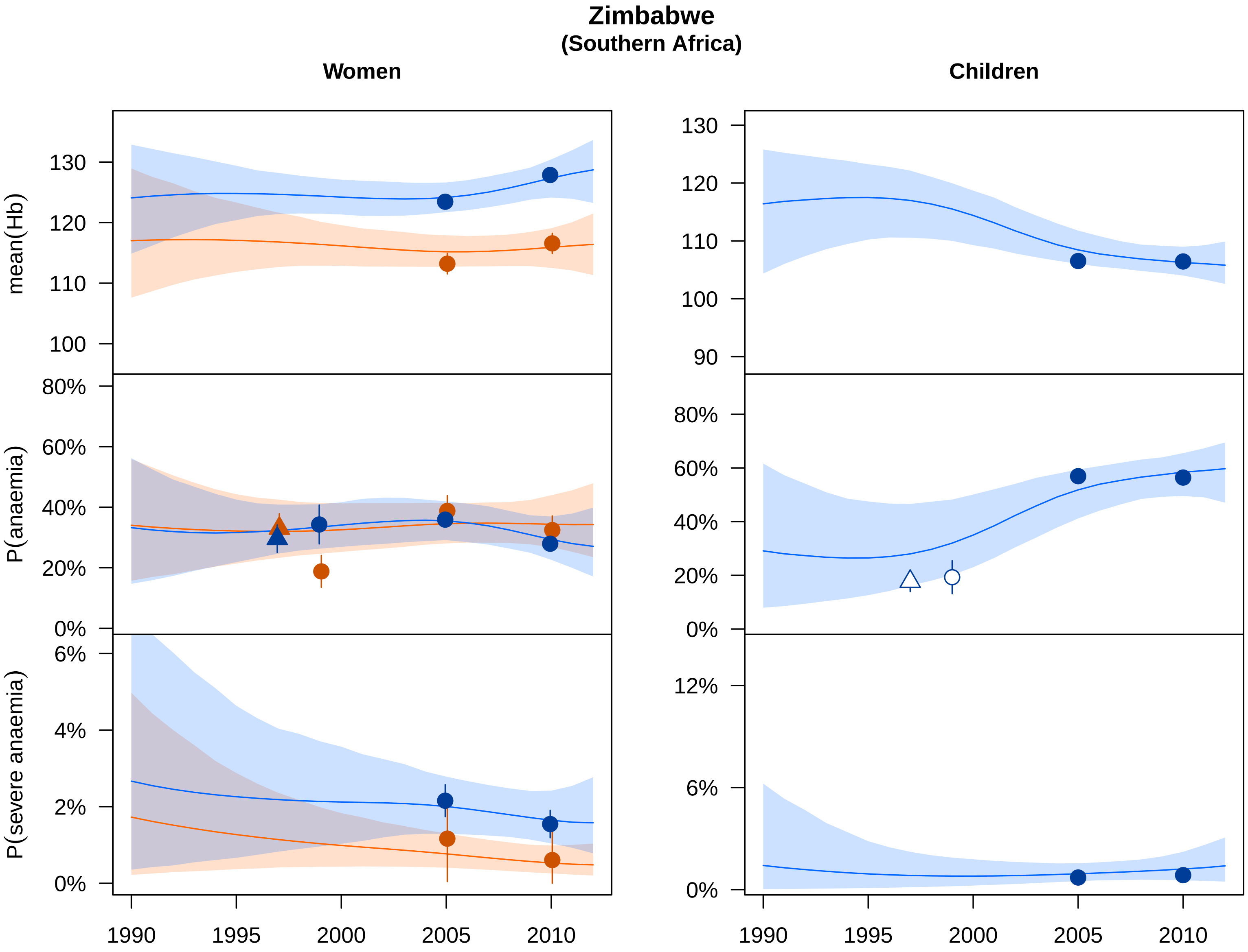
Zimbabwe
(Southern Africa)
Women
Children
mean(Hb)
P(anaemia)
P(severe anaemia)
130
120
110
100
90
80%
60%
40%
20%
0%
6%
4%
2%
12%
1990
1995
2000
2005
2010
